# Proceedings of Réanimation 2017, the French Intensive Care Society International Congress

**DOI:** 10.1186/s13613-016-0223-8

**Published:** 2017-01-10

**Authors:** Wulfran Bougouin, Eloi Marijon, Benjamin Planquette, Nicole Karam, Florence Dumas, David Celermajer, Daniel Jost, Lionel Lamhaut, Frankie Beganton, Alain Cariou, Guy Meyer, Xavier Jouven, Sudden Death Expertise Center, Côme Bureau, Julien Charpentier, Omar Ben Hadj Salem, Lucie Guillemet, Michel Arnaout, Alexis Ferre, Guillaume Geri, Nicolas Mongardon, Frédéric Pène, Jean-Daniel Chiche, Jean-Paul Mira, Guylaine Labro, François Belon, Vinh-Phuc Luu, Julien Chenet, Guillaume Besch, Marc Puyraveau, Gaël Piton, Gilles Capellier, Maëlle Martin, Jean-Baptiste Lascarrou, Aurélie Le Thuaut, Jean-Claude Lacherade, Laurent Martin-Lefèvre, Maud Fiancette, Isabelle Vinatier, Christine Lebert, Konstantinos Bachoumas, Aihem Yehia, Matthieu Henry-Laguarrigue, Gwenhaël Colin, Jean Reignier, Elodie Privat, Joséphine Escutnaire, Cyrielle Dumont, Valentine Baert, Christian Vilhelm, Hervé Hubert, Vincent Robert-Edan, Karim Lakhal, Andrew Quartin, Brian Hobbs, Cynthia Cely, Cynthia Bell, Tai Pham, Roland Schein, Yimin Geng, Chaan Ng, Stephan Ehrmann, Charlotte Salmon Gandonnière, Julie Boisramé-Helms, Olivier Le Tilly, Isabelle Benz De Bretagne, Emmanuelle Mercier, Julie Mankikian, Anne Bretagnol, Ferhat Meziani, Jean Michel Halimi, Chantal Barin Le Guellec, Stéphane Gaudry, David Hajage, Florence Tubach, Bertrand Pons, Eric Boulet, Alexandre Boyer, Guillaume Chevrel, Nicolas Lerolle, Dorothée Carpentier, Nicolas de Prost, Alexandre Lautrette, Julien Mayaux, Saad Nseir, Jean-Damien Ricard, Didier Dreyfuss, AKIKI Study Group, René Robert, Franscesco Garzotto, Eric Kipnis, Ciro Tetta, Claudio Ronco, David Schnell, DO-RE-MI-FA Group, Bourmaud Aurelie, Marie Reynaud, Christophe Clec’h, Mourad Benyamina, François Vincent, Christophe Mariat, Caroline Bornstain, Stephane Rouleau, Christophe Leroy, Yves Cohen, Jerome Morel, Matthieu Legrand, Jeremy Terreaux, Michaël Darmon, Marie Cantier, Adeline Morisot, Emmanuel Guérot, Emmanuel Canet, Etienne De Montmollin, Guillaume Voiriot, Mathilde Neuville, Jean-François Timsit, Romain Sonneville, ENCEPHALITICA Study Group, Abdallah Fayssoil, Tania Stojkovic, Anthony Behin, Adam Ogna, Frédéric Lofaso, Pascal Laforet, Karim Wahbi, Helene Prigent, Denis Duboc, David Orlikowski, Bruno Eymard, Djillali Annane, Loic Le Guennec, Clémentine Cholet, Nicolas Bréchot, Guillaume Hekimian, Sébastien Besset, Guillaume Lebreton, Ania Nieszkowska, Jean Louis Trouillet, Pascal Leprince, Alain Combes, Charles-Edouard Luyt, Marion Griton, Musa Sesay, Nadia Sibaï De Panthou, Thomas Bienvenu, Matthieu Biais, Karine Nouette-Gaulain, Guillaume Fossat, Florian Baudin, Cécile Coulanges, Sabrine Bobet, Arnaud Dupont, Léa Courtes, Dalila Benzekri, Toufik Kamel, Grégoire Muller, Nicolas Bercault, François Barbier, Isabelle Runge, Marie Skarzynski, Armelle Mathonnet, Thierry Boulain, Youenn Jouan, Noémie Teixera, Claire Hassen-Khodja, Antoine Guillon, Christophe Gaborit, Leslie Grammatico-Guillon, Cécile Rebière, Elie Azoulay, Benoit Misset, Stephane Ruckly, Maïté Garrouste-Orgeas, Nancy Kentish-Barnes, Jacques Duranteau, Marie Thuong, Liliane Joseph, Anne Renault, Olivier Lesieur, Anne-Gaelle Si Larbi, Gérald Viquesnel, Benjamin Zuber, Sophie Marque, Stanislas Kandelman, Nicolas Pichon, Bernard Floccard, Marion Galon, Sylvie Chevret, Nancy Kentish-Barnes, Valérie Seegers, Stéphane Legriel, Samir Jaber, Jean Yves Lefrant, Danielle Reuter, Olivier Guisset, Christophe Cracco, Amélie Seguin, Jacques Durand-Gasselin, Marine Thirion, Zoé Cohen-Solal, Hélène Foulgoc, Julien Rogier, Elsa Delobbe, Frédérique Schortgen, Pierre Asfar, Boisramé-Helms Julie, David Grimaldi, Grelon Fabien, Nadia Anguel, Lasocki Sigismond, Henry-Lagarrigue Matthieu, Frédéric Gonzalez, Legay François, Christophe Guitton, Maleka Schenck, Doise Jean-Marc, Peter Radermacher, for the HYPER2S Investigators and REVA Research Network, Nancy Kentish-Barnes, Joseph Nsiala Makunza, Mejeni Kamdem Nathalie, Akilimali Pierre, Kilembe Manzanza Adolphe, Rafael Mahieu, Thomas Reydel, Angéline Jamet, Nicolas Chudeau, Julien Huntzinger, Steven Grange, Anne Courte, Jérémie Lemarie, Sébastien Gibot, Julia Champey, Jean Dellamonica, Damien Du Cheyron, Damien Contou, Jean-Marc Tadié, Martin Cour, Gaetan Beduneau, Antoine Marchalot, Laurent Guérin, Sebastien Jochmans, Nicolas Terzi, Sebastien Preau, Christian Brun-Buisson, Armand Mekontso Dessap, for the Purpura Fulminans Study Group, Meryl Vedrenne-Cloquet, Sophie Breinig, Camille Jung, Maxime Brussieux, Marie-Odile Marcoux, Xavier Durrmeyer, Renaud Blondé, François Angoulvant, Jérôme Grasset, Jérôme Naudin, Stéphane Dauger, GFRUP RMEF, Solenn Remy, Karine Kolev-Descamp, Julie Demaret, Guillaume Monneret, Etienne Javouhey, Maryline Chomton, Michaël Sauthier, Emilie Vallieres, Philippe Jouvet, Guillaume Geslain, Isabelle Guellec, Jérôme Rambaud, Matthieu Schmidt, Peter Schellongowski, Amandine Dorget, Nicolo Patroniti, Fabio Silvio Taccone, Dinis Reis Miranda, Jean Reuter, Hélène Prodanovic, Marc Pierrot, Martin Balik, Sunghoon Park, REVA ECMOnet, Claude Guérin, Laurent Papazian, Reignier Jean, Louis Ayzac, Anderson Loundou, Jean-Marie Forel, Mehdi Mezidi, Mylène Aublanc, Sophie Perinel-Ragey, Floriane Lissonde, Aurore Louf-Durier, Romain Tapponnier, Hodane Yonis, Remi Coudroy, Jean-Pierre Frat, Florence Boissier, Arnaud W. Thille, Flore Richard, Hélène Le Gullou-Guillemette, Jonathan Fahri, Achille Kouatchet, Laetitia Bodet-Contentin, Denis Garot, Déborah Le Pennec, Laurent Vecellio, Elsa Tavernier, Pierre François Dequin, Jonathan Messika, Yolaine Martin, Natacha Maquigneau, Christelle Puechberty, Annabelle Stoclin, Serge Villard, Aline Dechanet, Audrey De Jong, Marion Monnin, Mehdi Girard, Gérald Chanques, Nicolas Molinari, Maxens Decavèle, Sébastien Campion, Roukia Ainsouya, Marie-Cécile Niérat, Mathieu Raux, Thomas Similowski, Alexandre Demoule, Keyvan Razazi, Martial Tchir, Faten May, Guillaume Carteaux, Rougevin-Baville Pauline, Andronikof Marc, Jean Pierre Bedos, Koukabi Mehrsa, Carole Mauger-Briche, François Mijon, Pierre Trouiller, Benjamin Sztrymf, Pierre Cretallaz, Romain Mermillod-Blondin, Dominique Savary, Ines Sedghiani, Hamdi Doghri, Asma Jendoubi, Dhekra Hamdi, Mohamed Ali Cherif, Youssef Zied El Hechmi, Jerbi Zouheir, Nicolas Persico, Francois Maltese, Cécile Ferrigno, Amandine Bablon, Cécile Marmillot, Antoine Roch, Ines Sedghiani, Grégory Papin, Marc Gainnier, Laurent Argaud, Adrie Christophe, Bertrand Souweine, Dany Goldgran-Toledano, Guillaume Marcotte, Anne Sylvie Dumenil, Schwebel Carole, Jerôme Cecchini, Samuel Tuffet, Muriel Fartoukh, Damien Roux, Martial Thyrault, Mekontso Dessap Armand, Simon Chauveau, Nadège Wesner, Laurence Monnier-Cholley, Naïke Bigé, Hafid Ait-Oufella, Bertrand Guidet, Vincent Dubée, Pierre Labroca, Jérémie Lemarié, Gérard Chiesa, Isabelle Laroyenne, Léo Borrini, Rémi Klotz, Quoc Phan Sy, Marie-Christine Cristina, Jean Paysant, Pierre Fillâtre, Arnaud Gacouin, Matthieu Revest, Pierre Tattevin, Erwan Flecher, Yves Le Tulzo, Matthieu Jamme, Fabrice Daviaud, Nathalie Marin, Michael Thy, Baptiste Duceau, Fanny Ardisson, Valade Sandrine, Marion Venot, Benoît Schlemmer, Lara Zafrani, Stéphanie Pons, Lenka Styfalova, Lila Bouadma, Aguila Radjou, Jordane Lebut, Bruno Mourvillier, Richard Dorent, Marie-Pierre Dilly, Patrick Nataf, Michel Wolff, Aëlle Le Gall, Simon Bourcier, Yacine Tandjaoui-Lambiotte, Vincent Das, Mikael Alves, Naïke Bigé, Chtara Kamilia, Ammar Rania, Najeh Baccouch, Olfa Turki, Hmida Chokri Ben, Mabrouk Bahloul, Mounir Bouaziz, Claire Dupuis, Anne Perozziello, Julien Letheulle, Marc Valette, Cécile Herrmann-Storck, Laura Crosby, Khalid Elkoun, Benjamin Madeux, Frédéric Martino, Hélène Migueres, Pascale Piednoir, Matthias Posch, Guillaume Thiery, Minh-Tu Huynh-Ky, Pierre Alexandre Bouchard, Jean-François Sarrazin, François Lellouche, Mai-Anh Nay, Brice Lortat-Jacob, Bertrand Rozec, Marion Colnot, Nicolas Belin, Loïc Barrot, Jean-Christophe Navellou, Cyrille Patry, Claire Chaignat, Melanie Claveau, Frédéric Claude, Cécile Aubron, Zoe Mcquilten, Michael Bailey, Jasmin Board, Heidi Buhr, Bruce Cartwright, Mark Dennis, Paul Forrest, Carol Hodgson, David Mcilroy, Deirdre Murphy, Lynnette Murray, Vincent Pellegrino, David Pilcher, Jayne Sheldrake, Huyen Tran, Shirley Vallance, Jamie Cooper, Camille Bombled, Charles Vidal, Dimitri Margetis, Julien Amour, Domien Coart, Jasperina Dubois, Tom Van Herpe, Dieter Mesotten, Sébastien Bailly, Jc Lucet, Alain Lepape, François L’hériteau, Martine Aupée, Caroline Bervas, Sandrine Boussat, Anne Berger-Carbonne, Anaïs Machut, Anne Savey, REA-RAISIN Study Group, Jean-Jacques Tudesq, Sandrine Valade, Lionel Galicier, Cédric De Bazelaire, Nicolas Munoz-Bongrand, Xavier Mignard, Lucie Biard, Djamel Mokart, Martine Nyunga, Fabrice Bruneel, Antoine Rabbat, Pierre Perez, Anne Pascale Meert, Dominique Benoit, Eric Mariotte, Franck Ehooman, Rebecca Hamidfar-Roy, Yannick Hourmant, Arnaud Mailloux, Alexandra Beurton, Jean-Louis Teboul, Valentina Girroto, Galarza Laura, Christian Richard, Xavier Monnet, Vincent Dubée, Hamid Merdji, Julien Dang, Gabriel Preda, Jean-Luc Baudel, Cyrielle Desnos, Michel Zeitouni, Ines Belaroussi, Antoine Parrot, Clarisse Blayau, Jean-Pierre Fulgencio, Christophe Quesnel, Vincent Labbe, Marc Pineton De Chambrun, François Beloncle, Sybille Merceron, Yannick Fedun, Bernard Lecomte, Jérôme Devaquet, Marc Puidupin, Bruno Verdière, Zahir Amoura, for the EurêClark Study Group, Constance Vuillard, Jais Xavier, Delphine Bourlier, Amar David, Sattler Caroline, Montani David, Simmoneau Gerald, Sitbon Olivier, Marc Humbert, Savale Laurent, Olivier Dujardin, Adrien Bouglé, Hamou Nora Ait, Joe Elie Salem, Najoua El-Helali, Zoé Coppere, Aude Gibelin, Clementine Taconet, Michel Djibre, Adel Maamar, Elen Colobert, Pierre Fillatre, Fabrice Uhel, Christophe Camus, Josquin Moraly, Redouane Dahoumane, Eric Maury, Boun Kim Tan, Vivier Emmanuel, Misslin Pauline, Parmeland Laurence, Poirié Philippe, Jean-Ralph Zahar, Haond Catherine, Pommier Christian, Ait-Bouziad Karim, Hocine Mounia, Témime Laura, Vero Hanitra Rasoldier, Guy Mager, Jean-Pierre Eraldi, Stéphanie Gelinotte, François Bougerol, Julien Dehay, Jean-Philippe Rigaud, Pierre Louis Declercq, Julien Michel, Nejla Aissa, Sandrine Henard, Philippe Guerci, Ichraq Latar, Bruno Levy, Nicolas Girerd, Antoine Kimmoun, Saousen Ben Abdallah, Sabrine Nakaa, Kmar Hraiech, Dhouha Ben Braiek, Ali Adhieb, Abdelwaheb M’ghirbi, Ali Ousji, Zeineb Hammouda, Fekri Abroug, Walid Sellami, Zied Hajjej, Walid Samoud, Iheb Labbene, Mustapha Ferjani, Fatma Kaaniche Medhioub, Rania Allela, Najla Ben Algia, Samar Cherif, Delphine Attia, Andrianjafy Herinjatovo, Xavier Laborne Francois, Med Aziz Bouhouri, Mohamed Taoufik Slaoui, A. Soufi, K. Khaleq, D. Hamoudi, A. Nsiri, R. Harrar, Eric Maury, Suzanne Goursaud, Maxime Gauberti, Paul-Emile Labeyrie, Thomas Gaberel, Véronique Agin, Eric Maubert, Denis Vivien, Clément Gakuba, Anwar Armel, Rchi Abdou, Samira Kalouch, Khalid Yaqini, Aziz Chlilek, Walid Sellami, Soumaya Ben Yedder, Alexandre Tonnelier, Fabien Hervé, Guillaume Halley, Jean-Luc Frances, Mickael Moriconi, Mathieu Saoli, Aude Garnero, Didier Demory, Jean Michel Arnal, Bertrand Canoville, Cédric Daubin, Jennifer Brunet, Hassen Ben Ghezala, Salah Snouda, Chiekh Imen Ben, Moez Kaddour, Islem Ouanes, Mahdi Marzouk, Françoise Haniez, Hélène Jaillet, Henri Maas, Pierre Andrivet, Christian Darné, François Viau, Hassen Ben Ghezala, Islem Ouanes, Laurence Dangers, Claire Montlahuc, Sébastien Perbet, Islem Ouanes, Zeineb Hamouda, Sabrine Nakee, Lamia Ouanes-Besbes, Khaoula Meddeb, Ahmed Khedher, Nesrine Sma, Jihene Ayachi, Messaouda Khelfa, Nesrine Fraj, Hend Ben Lakhal, Hedia Hammed, Raja Boukadida, Hajer Hafsa, Imed Chouchene, Mohamed Boussarsar, Braiek Dhouha Ben, Lamia Ouanes-Besbes, Kaoutar Benatti, A. Dafir, W. Aissaoui, W. Elallame, W. Haddad, R. Cherkab, C. Elkettani, L. Barrou, Zakaria Ait Hamou, Xavier Repessé, Cyril Charron, Alix Aubry, Alexis Paternot, Julien Maizel, Michel Slama, Antoine Vieillard-Baron, Ahlem Trifi, Sami Abdellatif, Meriem Fatnassi, Foued Daly, Rochdi Nasri, Khaoula Ben Ismail, Salah Ben Lakhal, Florian Bazalgette, Aurelien Daurat, Claire Roger, Laurent Muller, Denis Doyen, Rémi Plattier, Alexandre Robert, Hervé Hyvernat, Gilles Bernardin, Mathieu Jozwiak, Julia Gimenez, Pablo Mercado, François Depret, Najla Tilouch, Houda Mater, Ben Sik Ali Habiba, Oussama Jaoued, Rim Gharbi, Mohamed Fekih Hassen, Souheil Elatrous, Pierre Pasquier, Quentin Vuillemin, Jean-Vivien Schaal, Thibault Martinez, Sandrine Duron, Marion Trousselard, Pierre-Eric Schwartzbrod, Thomas Baugnon, Laurent Dupic, Caroline Duracher Gout, Laure De Saint Blanquat, Sylvie Séguret, Gaelle Le Ficher, Gilles Orliaguet, Philippe Hubert, Groupe Communication et Simulation en Pédiatrie, Naïke Bigé, Guillaume Leblanc, Raphael Briand, Lucas Brousse, Valentine Brunet, Léonard Chatelain, Dominique Prat, Frédéric Jacobs, Nadège Demars, Olfa Hamzaoui, Guy Moneger, Benjamin Sztrymf, Emilie Duburcq-Gury, Léa Satre-Buisson, Thibault Duburcq, Julien Poissy, Laurent Robriquet, Merce Jourdain, Thierry Sécheresse, Mattéo Miquet, Alexis Simond, Pascal Usseglio, Yamina Hamdaoui, Mohamed Boussarsar, Victoire Desailly, Patrick Brun, Pauline Iglesias, Jérémie Huet, Clémence Masseran, Antoine Claudon, Clément Ebeyer, Thomas Truong, Antoine Tesnière, Alexandre Mignon, Stéphane Gaudry, Dabor Resiere, Ruddy Valentino, Julien Fabre, Benoit Roze, Jean-Louis Ferge, Cyrille Charbatier, Sabia Marie, Michel Scholsser, Signate Aitsatou, Mathieu Raad, Andre Cabie, Hossein Mehdaoui, Clement Cousin, Christophe Rousseau, Jean-François Llitjos, Fanny Alby-Laurent, Julie Toubiana, Nadia Belaidouni, Marlène Cherruault, Jérome Tamburini, Didier Bouscary, Sarah Fert, Eugénie Delile, Emmanuel Besnier, David Coquerel, Rémi Nevière, Vincent Richard, Fabienne Tamion, Chaojie Wei, Huguette Louis, Schmitt Margaux, Albuisson Eliane, Orlowski Sophie, Antoine Kimmoun, Zakaria Riad, Marine Coroir, Bernard Rémy, Bombled Camille, Jeremie Joffre, Philippe Aegerter, Dejan Ilic, Marc Ginet, Caroline Pignard, Philippe Nguyen, Guillaume Mourey, Emmanuel Samain, Sebastien Pili-Floury, Romain Jouffroy, Caill Nicolas, Jean-Claude Alvarez, Maraffi Tomasso, Pascal Philippe, Jean-Herlé Raphalen, J. Baud Frédéric, Benoit Vivien, Carli Pierre, Frederic Baud, Hana Fredj, Youssef Blel, Nozha Brahmi, Hassen Ben Ghezala, Anne-Sophie Hanak, Isabelle Malissin, Joel Poupon, Patricia Risede, Lucie Chevillard, Bruno Megarbane, Manel Barghouth, Aymen M’rad, Marwa Ben Hmida, Hafedh Thabet, Hao Liang, Jacques Callebert, Camille Lagard, Bruno Megarbane, Sahar Habacha, Bassem Chatbri, Christophe Camillerapp, Laurence Labat, Marion Soichot, Pierre Garçon, Antoine Goury, Lamia Kerdjana, Sebastian Voicu, Nicolas Deye, Bruno Megarbane, Anwar Armel, Benqqa Anas, Mezgui Othman, S. Moumine, S. Kalouch, K. K. Yakini, A. Chlilek, Ahmed Hajji, Assaad Louati, Ammar Khaldi, Aida Borgi, Nargess Ghali, Asma Bouziri, Khaled Menif, Jaballah Najla Ben, Anwar Armel, Jeanne Brochon, Mihaela Dumitrescu, Sarah Thévenot, Jean-Pascal Saulnier, Khaled Husseini, Catherine Laland, Julie Cremniter, Anne Bousseau, Olivier Castel, Cassandra Brémaud-Csizmadia, Margot Diss, Aurélie Portefaix, Julien Berthiller, Yves Gillet, Nabil Tabet Aoul, Ali Douah, Zakaria Addou, Houari Youbi, Mohamed Moussati, Kamel Belhabiche, Souad Mir, Sanaa Abada, Zerhouni Amel, Nabil Aouffen, Zina Bouzit, Ahmed H. Grati, Gilles F. Dhonneur, Mohamed Boussarsar, Nicolas Lau, Ilham Mezhari, Nicolas Roucaud, Matthieu Le Meur, Rémi Paulet, Jean-Michel Coudray, Wahiba Imène Ghomari, Reda Boumlik, Vincent Peigne, Jean-Louis Daban, Mathieu Boutonnet, Bernard Lenoir, Hafiani Yassine, Cheikh Chaigar Mohamed, Allali Khalid, Moussaid Ihssan, Elyoussoufi Said, Salmi Said, Amira Ben Jazia, Jaziri Fatima, Skouri Wafa, Bennasr Maha, Ben Abdelghni Khaoula, Turki Sami, B. Abdallah Taeib, Fatma Kaaniche Medhioub, Virginie Rollet-Cohen, Philippe Sachs, Zied Merchaoui, Sylvain Renolleau, Mehdi Oualha, Maxime Eloi, Sandrine Jean, Maryne Demoulin, Cécile Valentin, Julia Guilbert, Hervé Walti, Ricardo Carbajal, Pierre-Louis Leger, Yasemin Karaca-Altintas, Astrid Botte, Julien Labreuche, Elodie Drumez, Patrick Devos, Franck Bour, Francis Leclerc, Ayari Ahmed, Menif khaled, Assaad Louati, Borgi Aida, Khaldi Ammar, Ghali Narjess, Hajji Ahmed, Bouziri Asma, Nejla Ben Jaballah, Pierre-Louis Leger, Julien Pansiot, Valérie Besson, Bruno Palmier, Olivier Baud, Bruno Cauli, Christiane Charriaut-Marlangue, Amélie Mansuy, Fabrice Michel, Stéphane Le Bel, Julia Boubnova, Fabrice Ughetto, Caroline Ovaert, Virginie Fouilloux, Olivier Paut, Matthias Jacquet-Lagrèze, Nicolas Tiebergien, Najib Hanna, Jean-Noël Evain, Florent Baudin, Sonia Courtil-Teyssedre, Dominique Bompard, Marc Lilot, Laurent Chardonal, Jean-Luc Fellahi, Claire Claverie, Guillaume Pouessel, Aimée Dorkenoo, Jean-Marie Renaudin, Mireille Eb, Antoine Deschildre, Stéphane Leteurtre, Hafiani Yassine, Belkadi Kamal, Oboukhlik Adil, Aalalam Ouafa, Moussaoui Mouhamed, Charkab Rachid, Barrou Lahoucine, Fahmi Dachraoui, Sabrine Nakkaa, Hammouda Zaineb, Dorra Mlika, Olfa Gloulou, Mohamed Boussarsar, Setti-Aouicha Zelmat, Djamila-Djahida Batouche, Belkacem Chaffi, Fatima Mazour, Nadia Benatta, Ines Fathallah, Rafaa Aloui, Aymen Zoubli, Nadia Kouraichi, Ines Fathallah, N. Kouraichi, Shireen Salem, Eric Vicaut, Bruno Megarbane, David Ambroise, Anne-Marie Loriot, Emmanuel Bourgogne, Bruno Megarbane, Hatem Ghadhoune, Guissouma Jihene, Insaf Trabelsi, Hend Allouche, Habib Brahmi, Mohamed Samet, Hatem El Ghord, Rodolphe Lebeau, Jean-Louis Laplanche, Nadia Benturquia, Bruno Megarbane, Youssef Blel, A. M’rad, Fatma Essafi, A. Benabderrahim, Romain Jouffroy, Dabor Resiere, Bruno Sanchez, Jocelyn Inamo, Bruno Megarbane, Djamila-Djahida Batouche, Amel Zerhouni, Kheira Tabeliouna, Amine Negadi, Zahia Mentouri, Fanny Le Gall, Jean-Luc Hanouz, Hervé Normand, Abdo Khoury, Fatimata Seydou Sall, Alban De Luca, Aurore Pugin, Lionel Pazart, Chrystelle Vidal, Franck Leroux, Abdo Khoury, Erwan L’Her, Nicolas Marjanovic, Abdo Khoury, Thibault Desmettre, Christophe Lambert, Sophie Perinel Ragey, Loredana Baboi, Jean-Etienne Bazin, Catherine Koffel, Gilles Dhonneur, Zina Bouzit, Larbi Bradai, Issam Ben Ayed, Fethi Aissa, Hakim Haouache, Yoann Marechal, Patrick Biston, Michael Piagnerelli, Perrine Bortolotti, Delphine Colling, Vincent Colas, Benoit Voisin, Florent Dewavrin, Thierry Onimus, Patrick Girardie, Fabienne Saulnier, Tomas Urbina, Yann Nguyen, Anne-Lise Druoton, Marc Soudant, Damien Barraud, Marie Conrad, Aurélie Cravoisy-Popovic, Lionel Nace, Pierre-Edouard Bollaert, Ruste Martin, Laurent Bitker, Jean-Christophe Richard, David Brossier, Isabelle Goyer, Christopher Marquis, Marie Lampin, Alain Duhamel, Hélène Béhal, Tahar Dhaoui, Véronique Godeffroy, Eve Devouge, Dominique Evrard, Florence Delepoulle, Sylvie Racoussot, Bruno Grandbastien, Marie Lampin, Claire Heilbronner, Emeline Roy, Alexandra Masson, Alice Hadchouel-Duvergé, Virginie Rigourd, Elise Delacroix, Isabelle Wroblewski, Isabelle Pin, Anne Ego, Valerie Payen, Thierry Debillon, Anne Millet, Julien Denot, Véronique Berthelot, Emilie Thueux, Marie Reymond, Alexandra De Larrard, Alain Amblard, Pierre-Louis Leger, Nabil Tabet Aoul, Virginie Lemiale, Johanna Oziel, Noelle Brule, Anne-Sophie Moreau, Takoua Marhbène, Salma Sellami, Amira Jamoussi, Samia Ayed, Emna Mhiri, Leila Slim, Jalila Ben Khelil, Mohamed Besbes, Sylvain Chawki, Aicha Hamdi, Magali Ciroldi, Alice Cottereau, Edouard Obadia, Yoann Zerbib, Claire Andrejak, Sylvie Ricome, Hervé Dupont, François Baudin, Pauline Dureau, Audrey Tanguy, Charlotte Arbelot, Hassen Kais Ben, Ahmed Charfeddine, Benjamin Granger, Lucile Laporte, Coralie Hermetet, Kais Regaieg, Rim Khemakhem, Hedi Chelly, Chaigar Mohammed Cheikh, Hamid Mountij, Kawtar Rghioui, Wafae Haddad, Rachid Cherkab, Houcine Barrou, Aitmouden Naima, Othmani M. bennani, Kais Regaieg, Ahmed Douib, Amal Samet, Pierre-Julien Cungi, Cédric Nguyen, Jean Cotte, Erwan D’aranda, Eric Meaudre, Jean-Phillipe Avaro, Mohamed Taoufik Slaoui, Amel Mokline, Imene Rahmani, Achraf Laajili, Helmi Amri, Lazheri Gharsallah, Bahija Gasri, Sofiene Tlaili, Rym Hammouda, Amen Allah Messadi

**Affiliations:** 1 Cardiologie, Hôpital Européen Georges-Pompidou, Rue Leblanc, Paris, France; 2 Usip, Hopital Europeen Georges-Pompidou, Paris, France; 3 Service d’accueil des urgences, Hôpital Cochin, Paris, France; 4 Réanimation médicale, Hôpital Cochin, Paris, France; 5 Cardiology, Sydney Medical School, Sydney, Australia; 6 Bspp, B.s.p.p., Paris, France; 7 Réanimation adulte, Hôpital Necker - Enfants Malades, Paris, France; 8 Paris descartes, Inserm U970, Paris, France; 9 Service de Pneumologie et Soins Intensifs, Hopital Europeen Georges-Pompidou, Paris, France; 10 Service de pneumologie et réanimation médicale, Pitié-Salpêtrière Hospital, Paris, France; 11 Service d’Anesthésie et des Réanimations Chirurgicales, Hôpital Henri Mondor, Créteil, France; 12 Service de réanimation médicale, Hôpital Cochin, Assistance Publique Hôpitaux de Paris, Paris, France; 13 Réanimation médicale, CHU de Besançon, Besançon, France; 14 Centre de méthodologie clinique, CHU de Besançon, Besançon, France; 15 Département d’anesthésie et de réanimation, CHU de Besançon, Besançon, France; 16 Réanimation chirurgicale, CHU de Besançon, Besançon, France; 17 Réanimation chirurgicale, CHU Jean Minjoz, Besançon, France; 18 Intensive Care, District Hospital Center, La Roche-Sur-Yon, France; 19 Clinical Research Unit, District Hospital Center, La Roche-Sur-Yon, France; 20 Réanimation polyvalente, Centre Hospitalier Départemental – site de La Roche-sur-Yon, La Roche-Sur-Yon, France; 21 Réanimation polyvalente, Hopital Les Oudaries, La Roche-Sur-Yon, France; 22 Réanimation, Centre Hospitalier Départemental – site de La Roche-sur-Yon, La Roche-Sur-Yon, France; 23 Medical Intensive Care Unit, University Hospital Center, Nantes, France; 24 Réanimation pédiatrique, C.H. Régional Universitaire de Lille (CHRU de Lille), Lille, France; 25 French National Out-of-Hospital Cardiac Arrest Registry (réac), Lille, France; 26 Public Health Department, Université Lille 2 – Faculté de Médecine Henri Warembourg, Loos, France; 27 Public Health Department, Université Lille 2 – Faculté de Médecine Henri Warembourg, Lille, France; 28 Service de réanimation chirurgicale, Hôpital Guillaume et René Laënnec, CHU de Nantes, Nantes, France; 29 Division of pulmonary, Critical Care, Allergy and Sleep Medicine, Department of Medicine, University of Miami School of Medicine, Miami, USA; 30 Department of Biostatistics, University of Texas M.D. Anderson Cancer Center, Houston, TX USA; 31 Division of Pediatric Nephrology and Hypertension, University of Texas Health Science Center-Houston, Houston, TX USA; 32 Réanimation médico-chirurgicale, Hôpital Tenon, Paris, France; 33 Research Medical Library, University of Texas M.D. Anderson Cancer Center, Houston, TX USA; 34 Department of Radiology, University of Texas M.D. Anderson Cancer Center, Houston, TX USA; 35 Réanimation polyvalenteCHRU Hôpitaux de Tours, Tours, France; 36 Réanimation médicale, Nouvel Hôpital Civil, CHU Strasbourg, Strasbourg, France; 37 Réanimation, CHU de Strasbourg, Strasbourg, France; 38 Biochimie et biologie moléculaire, CHRU Hôpitaux de Tours, Tours, France; 39 Réanimation médicale polyvalente, Hôpital de La Source, CHR Orléans, Orléans, France; 40 Néphrologie, transplantation rénale et hémodialyse, CHRU Hôpitaux de Tours, Tours, France; 41 Service de réanimation médico-chirurgicale, CHU Louis Mourier, Colombes, France; 42 Département d’épidémiologie et de recherche clinique, Hôpital Louis-Mourier - APHP, Colombes, France; 43 Département d’epidémiologie et recherche clinique, Unité de Recherche Clinique Paris Nord, Paris, France; 44 Réanimation - Grands Brulés, CHU Pointe à Pitre - Abymes, Pointe a Pitre, France; 45 Intensive care unit, Hospital Center Regional University, Pointe-a-Pitre, Guadeloupe; 46 Val d’oise, Hôpital René Dubos, Pontoise, France; 47 Réanimation médicale, Centre Hospitalier Universitaire de Bordeaux, Bordeaux, France; 48 Essonnes, C.H. Sud Francilien, Corbeil-Essonnes, France; 49 Réanimation médicale, Centre Hospitalier Universitaire d’Angers, Angers, France; 50 Réanimation médicale, Centre Hospitalier Universitaire Rouen, Rouen, France; 51 Réanimation Médicale, Hôpital Henri Mondor, Créteil, France; 52 Réanimation médicale, CHU Gabriel-Montpied, Clermont-Ferrand, France; 53 Réanimation médicale, Hôpital Pitié-Salpêtrière, Paris, France; 54 Centre de Réanimation, Centre Hospitalier Régional Universitaire de Lille, Lille, France; 55 Service de Réanimation Médico-Chirurgicale, CHU Louis Mourier, Colombes, France; 56 Inserm, iame, umr 1137, Université Paris Diderot, Sorbonne Paris Cité, Paris, France; 57 Réanimation polyvalente, Hôpital Louis-Mourier (AP-HP), Colombes Cedex, France; 58 Réanimation médico-chirurgicale, Hôpital Louis-Mourier - APHP, Colombes, France; 59 Service de Réanimation médicaleCHU de Poitiers, Poitiers, France; 60 Réanimation médicaleCHU de Poitiers, Poitiers, France; 61 International Renal Research Institute of Vicenza, San Bortolo Hospital, Vicenza, Italy; 62 Réanimation chirurgicale, Centre Hospitalier Régional Universitaire de Lille, Lille, France; 63 Research Extracorporeal Therapies, Fresenius Medical Care, Bad Homburg Vor Der Höhe, Germany; 64 Service de reanimation, Service de Réanimation polyvalente, Angoulême, France; 65 Département de santé publique, Institut de Cancérologie de la Loire Lucien Neuwirth, Saint-Priest-En-Jarez, France; 66 Réanimation polyvalente b, Hospital Center University De Saint-Étienne, Saint-Priest-En-Jarez, France; 67 Réanimation medico-chirurgicale, Hôpital Avicenne, Bobigny, France; 68 Anesthésie réanimation et traitement chirurgical des grands brûlés, APHP - Hopital Saint-Louis, Paris, France; 69 Réanimation polyvalente, Groupe Hospitalier Intercommunal Le Raincy-Montfermeil, Montfermeil, France; 70 Néphrologie, Centre Hospitalier Universitaire de Saint-Étienne, Saint-Étienne, France; 71 Service de réanimation, Hospital Center D’angoulême, Angoulême, France; 72 Réanimation médicale, CHU Saint-Etienne - Hôpital Nord, Saint-Étienne, France; 73 Service de réanimation médicale et infectieuse, Hôpital Bichat-Claude Bernard-APHP, Paris, France; 74 Centre mémoire, clinique rainier iii, Hospital Center Princesse Grace, Monaco, Monaco; 75 Réanimation médicale, Hopital Europeen Georges-Pompidou, Paris, France; 76 Service de réanimation médicale, Hôpital Saint-Louis (AP-HP), Paris, France; 77 Réanimation médicale, Hôpital Saint-Louis (AP-HP), Paris, France; 78 Réanimation, C.H. Général Saint Denis hôpital Delafontaine, Saint-Denis, France; 79 Service de réanimation médicale, Hôpital Tenon (AP-HP), Paris, France; 80 Réanimation médicale et infectieuse, Hôpital Bichat-Claude Bernard (AP-HP), Paris, France; 81 Service de réanimation médicale et infectieuse, Hôpital Bichat-Claude Bernard (AP-HP), Paris, France; 82 Réanimation médicale et infectieuse, Hôpital Bichat-Claude Bernard, Paris, France; 83 Neurologie et reanimation polyvalente, Institute De Myologie et CHU Raymond Poincaré, Garches, France; 84 Neurologie, Institute De Myologie, Paris, France; 85 Réanimation polyvalente et pole ventilation à domicile, CHU Raymond Poincare, Garches, France; 86 Service d’explorations fonctionnelles respiratoires, Hôpital Raymond-Poincaré (AP-HP), Garches, France; 87 Cardiologie, Hôpital Cochin, Paris, France; 88 Réanimation médico-chirurgicale, Hôpital Raymond-Poincaré, Garches, France; 89 Service de réanimation médicaleGroupe Hospitalier Pitié Salpêtrière, Paris, France; 90 Service de chirurgie thoracique et cardiovasculaire, Hôpital Universitaire La Pitié-Salpêtrière, Université Pierre et Marie Curie, IHU ICAN, Paris, France; 91 Groupe Hospitalier Pitié Salpêtrière, Paris, France; 92 Service de réanimation médicale, Groupe Hospitalier La Pitié-Salpêtrière, Institut de Cardiométabolisme et Nutrition, Assistance Publique Hôpitaux de Paris, Paris, France; 93 Neuro ICUCHU – Hôpitaux de Bordeaux, Bordeaux, France; 94 Service de pneumologie, Hôpital de La Source, CHR Orléans, Orléans, France; 95 Service de réanimation chirurgicale, Hôpital de La Source, CHR Orléans, Orléans, France; 96 Loiret, Hôpital Régional Orléans La Source, Orléans, France; 97 Réanimation médicale, Centre Hospitalier Régional d’Orléans, Orléans, France; 98 Service des urgences, CHRU Hôpitaux de Tours, Tours, France; 99 Service d’information médicale, épidémiologie et économie de la santé, CHRU Hôpitaux de Tours, Tours, France; 100 Service d’information médicale, d’épidémiologie et d’économie de la santé, CHU Bretonneau, Tours, France; 101 Réanimation, Fondation Hopital Saint Joseph, Paris, France; 102 Réanimation médicale, Hospital Center University, Rouen, France; 103 Réanimation polyvalente, Groupe Hospitalier Paris-Saint-Joseph, Paris, France; 104 Statistics Department, Outcomerea research group, Paris, France; 105 Reanimation, Hôpital Michallon, Grenoble, France; 106 Umr 1137, Faculté de Médecine Xavier Bichat, Paris, France; 107 Réanimation médicale, Assistance Publique Hôpitaux de Paris, Hôpital Saint Louis, Paris, France; 108 Réanimation chirurgicale, Hôpital Bicêtre, Le Kremlin-Bicêtre, France; 109 Val d’oise, CH René Dubos, Pontoise, France; 110 Réanimation médicale, CHU Brest, Brest, France; 111 Réanimation, Centre Hospitalier la Rochelle, La Rochelle, France; 112 Hospital Foch, 92151 Suresnes, France; 113 Réanimation chirurgicale, C.H.U de Caen, Caen, France; 114 Intensive Care Unit, Hospital Center De Versailles, Le Chesnay, France; 115 ICU, C.H. Sud Francilien, Corbeil-Essonnes, France; 116 Anesthésie réanimation, Hôpital Beaujon (AP-HP), Clichy, France; 117 Service de réanimation polyvalente, Centre Hospitalier Universitaire de Limoges, Limoges, France; 118 Département anesthésie-réanimation, Hôpital Édouard Herriot, Lyon, France; 119 Réanimation médicale, AP-HP, Hôpital Saint Louis, Paris, France; 120 Service de biostatistique et information médicale, Hôpital Saint-Louis, Paris, France; 121 Umr 669, Inserm, Paris, France; 122 Réanimation, Centre Hospitalier de Versailles, Le Chesnay, France; 123 DAR B, Hôpital Saint Eloi, Montpellier, France; 124 Réanimation chirurgicale, Hopital Carémeau, Nîmes, France; 125 Réanimation, Hopital Carémeau, Nîmes, France; 126 Réanimation médicale, Hôpital Saint-Louis, Paris, France; 127 Réanimation médicale-hôpital saint-andré, Centre Hospitalier Universitaire de Bordeaux, Bordeaux, France; 128 Réanimation médicale, Centre Hospitalier Universitaire de Caen, Caen, France; 129 Réanimation, Hôpital Sainte-Musse, Toulon, France; 130 Val d’oise, Centre Hospitalier Victor Dupouy (Argenteuil), Argenteuil, France; 131 33, CHU – Hôpitaux de Bordeaux, Bordeaux, France; 132 Réanimation médicale, Hôpital Henri-Mondor (AP-HP), Créteil Cedex, France; 133 Réanimation, C.H.U. d’Angers, Angers, France; 134 Réanimation médicale, Nouvel Hôpital Civil, Hôpitaux Universitaires de Strasbourg, Strasbourg, France; 135 Service de réanimation polyvalente, Centre Hospitalier de Versailles, Le Chesnay, France; 136 Réanimation, C.H. – Le Mans, Le Mans, France; 137 Réanimation médicale, CHU de Bicêtre, Le Kremlin Bicêtre, France; 138 Réanimation chirurgicale, C.H.U. d’Angers, Angers, France; 139 Réanimation polyvalente, Hospital Center Departmental De Vendée, La Roche-Sur-Yon, France; 140 Réanimation, C.H. de Saint Brieuc, Saint-Brieuc, France; 141 Réanimation médicale, C.H.U. Hôtel Dieu, Nantes, France; 142 Réanimation médicale, C.H.R.U. Hôpitaux Universitaires Strasbourg, Strasbourg, France; 143 Réanimation, C.H. Chalon sur Saône William Morey, Chalon-Sur-Saône, France; 144 Institut für anästhesiologische pathophysiologie und verfahrensentwicklung, Universitätsklinikum Ulm, Ulm, Germany; 145 Anesthésie-Réanimation, Cliniques Universitaires de Kinshasa, Kinshasa, Democratic Republic of the Congo; 146 Statistics, School of Public Health, Kinshasa, Democratic Republic of the Congo; 147 Anesthéthésie-réanimation, Cliniqes Universitaires de Kinshasa, Kinshasa, Democratic Republic of the Congo; 148 Réanimation médico-chirurgicale, C.H. - Le Mans, Le Mans, France; 149 Réanimation médicale, Centre hospitalier Bretagne Atlantique, Vannes, France; 150 Réanimation polyvalente, Centre Hospitalier Yves le Foll, Saint-Brieuc, France; 151 Réanimation médicale, Hôpital Central, C.H.U. de Nancy, Nancy, France; 152 Réanimation médicale, C.H.U. Grenoble, Grenoble, France; 153 Réanimation médicale, Centre Hospitalier Universitaire Archet, Nice, France; 154 Service de réanimation médicale, Hôpital Henri Mondor, Créteil, France; 155 Réanimation médicale, Centre Hospitalier Universitaire de Rennes, Rennes, France; 156 Service de maladies infectieuses et réanimation médicale, Centre Hospitalier Universitaire, Rennes, France; 157 Réanimation Médicale, Hospices Civils de Lyon - Groupement Hospitalier Edouard Herriot, Lyon, France; 158 Service de réanimation médicale, Centre Hospitalier de Dieppe, Dieppe, France; 159 Réanimation polyvalente, Centre Hospitalier de Dieppe, Dieppe, France; 160 Réanimation médicale, Hôpital Ambroise Paré (AP-HP), Boulogne-Billancourt, France; 161 Service de Réanimation, Centre Hospitalier Marc Jacquet, Melun, France; 162 Service de réanimation médicale, Clinique de Réanimation Médicale, Grenoble, France; 163 Réanimation médicale, Centre Hospitalier Régional Universitaire de Lille, Lille, France; 164 Service de réanimation néonatale, C.H. Intercommunal Créteil, Créteil, France; 165 Service de réanimation néonatale et pédiatrique, Hospital Center University Toulouse - Casselardit Ancely, Toulouse, France; 166 Centre de recherche clinique, C.H. Intercommunal Créteil, Créteil, France; 167 Mayotte, CHM, Mamoudzou, France; 168 Urgences pédiatriquesCHU Necker-Enfants Malades, Paris, France; 169 Iriseo, Iriseo - 3D Supports multimédia Interactifs, Saint-Victurnien, France; 170 Réanimation et surveillance continue pédiatriques, CHU Robert Debré, Paris, France; 171 Réanimation pédiatrique, Hôpital Femme Mère Enfant, Bron/Lyon, France; 172 Laboratoire d’immunologie cellulaire, Hospices Civils de Lyon - Groupement Hospitalier Edouard Herriot, Lyon, France; 173 Soins intensifs pédiatriques, CHU Sainte-Justine, Montréal, Canada; 174 Microbiology Department, CHU Sainte-Justine, Montréal, Canada; 175 Hôpital Robert-Debré (AP-HP), Paris, France; 176 Réanimation pédiatrique et néonatale, Hopital pour enfants Trousseau, Paris, France; 177 Service de réanimation médicale, Groupe Hospitalier Pitié-Salpêtrière, Paris, France; 178 Unit 13i2, Department of Medicine I/intensive care, Vienna, Austria; 179 Anestesia e raiamizione, Ospedale San Gerardo, Monza, Italy; 180 Service de Soins Intensifs, Hôpital Erasme, Brussels, Belgium; 181 Intensive Care, Erasmus University Medical Center, Rotterdam, Netherlands; 182 Unité de réanimation et de surveillance continueservice de pneumologie et réanimation médicale, Pitié-Salpêtrière Hospital, Paris, France; 183 Service de réanimation medicale, Hopital Universitaire d’Angers, Angers, France; 184 Department of Anaesthesia and Intensive Care, General University Hospital in Prague, Prague, Czech Republic; 185 Pulmonaryallergy and Critical Care, Hallym University Sacred Heart Hospital, Seoul, Republic of Korea; 186 Réanimation médicale, Hôpital de la Croix-Rousse, Lyon, France; 187 Service de réanimation-détresses respiratoires et infections sévères, Hôpital Nord, Marseille, France; 188 Réanimation DRIS, Hôpital Nord APHM, Marseille, France; 189 Réanimation médicale, CHU Hôtel-Dieu Nantes, Nantes, France; 190 C-clin, Hôpital Henry Gabrielle, Saint-Genis-Laval, France; 191 Unité de recherche de santé publique, Faculté de Médecine secteur Timone (Aix-Marseille Université), Marseille, France; 192 Réanimation médicale, Hospital Nord, Marseille, France; 193 Service de réanimation médicale, HEGP, CHU de Poitiers, Paris, France; 194 Réanimation Médicale, Hôpital Européen Georges-Pompidou (AP-HP), Paris, France; 195 Service de réanimation médicale et médecine hyperbare, Centre Hospitalier Universitaire d’Angers, Angers, France; 196 Département de biologie des agents infectieux et pharmaco-toxicologie, Centre Hospitalier Universitaire d’Angers, Angers, France; 197 Service des maladies du sang, Centre Hospitalier Universitaire d’Angers, Angers, France; 198 CEPR (Inserm U1100/EA 6305), aérosolthérapie et biomédicaments à visée respiratoire, Université François Rabelais, Tours, France; 199 Inserm cic 1415, CHRU Hôpitaux de Tours, Tours, France; 200 Réanimation-uscm, Gustave Roussy, Villejuif, France; 201 Unité de recherche clinique, Hôpital Bichat-Claude Bernard (AP-HP), Paris, France; 202 Dim, Hôpital La Colombière, Montpellier, France; 203 Inserm umr_s 1158 “neurophysiologie respiratoire expérimentale et clinique”, Pitié-Salpêtrière Hospital, Paris, France; 204 Département d’anesthésie-réanimation et umr_s 1158, Hôpital Universitaire Pitié-Salpêtrière, Paris, France; 205 Service de pneumologie et réanimation médicale, Groupe Hospitalier Pitié-Salpêtrière, Paris, France; 206 Réanimation polyvalente - surveillance continue, Ctre Hospitalier Intercommunal de Villeneuve Saint GeorgesLucie et Raymond AUBRAC, Villeneuve-Saint-Georges, France; 207 Réanimation polyvalente, Hôpital Antoine Béclère, Clamart, France; 208 Service d’accueil des urgences, Hôpital Antoine Béclère, Clamart, France; 209 Réanimation médico-chirurgicale, Centre Hospitalier de Versailles, Le Chesnay, France; 210 Service d’accueil des urgences, Centre Hospitalier de Versailles, Le Chesnay, France; 211 Service d’accueil des urgences, C.H. Intercommunal de Meulan-Les Mureaux, Meulan-En-Yvelines, France; 212 Réanimation polyvalente, C.H. Intercommunal de Meulan-Les Mureaux, Meulan-En-Yvelines, France; 213 Pôle sau - samu 74 - smur - réanimation, C.H. Annecy Genevois, Metz-Tessy, France; 214 Emergency and Intensive Care Department, hôpital habib thameur, Tunis, Tunisia; 215 Service d’accueil des urgences adultes, Hôpital Nord APHM, Marseille, France; 216 INSERM U1137 équipe 5, Université Paris Diderot, 75018 Paris, France; 217 Réanimation des urgences médicales, Hôpital de la Timone, Marseille, France; 218 Service de réanimation médicale, Hospices Civils de Lyon - Groupement Hospitalier Edouard Herriot, Lyon, France; 219 Hôpital Cochin, 75014 Paris, France; 220 Service de réanimation médicale, CHU Gabriel-Montpied, Clermont-Ferrand, France; 221 Réanimation polyvalente, Centre Hospitalier Général, Gonesse, France; 222 Reanimation medicale, Assistance Publique Hôpitaux de Paris, Paris, France; 223 Réanimation chirurgicale, Hôpital Antoine Béclère, Clamart, France; 224 38, C.H.U de Grenoble, C.H.U, La Tronche, La Tronche, France; 225 ICU, C.H. de Longjumeau, Longjumeau, France; 226 ICU, C.H. des Deux Vallées - site Longjumeau, Longjumeau, France; 227 Réanimation médicale, Hôpital Saint-Antoine, Paris, France; 228 Radiologie, Hôpital Saint-Antoine, Paris, France; 229 Service de Réanimation Médicale, Hôpital Saint-Antoine, Paris, France; 230 Service de rééducation et d’appareillage, Institut Robert Merle d’Aubigné, Valenton, France; 231 Service de médecine physique et de réadaptation, Centre Medico-Chirurgical de Readaptation des Massues, Lyon, France; 232 Service de médecine physique et de réadaptation, Hôpital d’Instruction des Armées Percy, Clamart, France; 233 Service de médecine physique et de réadaptation, Centre de médecine physique et de réadaptation de la tour de Gassies, Brugge, France; 234 Service de médecine physique et de réadaptation, Hôpital la renaissance sanitaire, Villiers-Saint-Denis, France; 235 Service de médecine physique et de réadaptation, Pôle Saint Hélier, Rennes, France; 236 Centre louis pierquin, Institut Régional de Médecine Physique et de Réadaptation, Nancy, France; 237 Service de chirurgie thoracique, cardiaque et vasculaire, Centre Hospitalier Universitaire, Rennes, France; 238 ICUREsearch, Paris, France; 239 Réanimation médicale et des maladies infectieuses, Hôpital Bichat-Claude Bernard, Paris, France; 240 Réanimation Médicale et Infectieuse, GH Bichat Claude Bernard, Paris, France; 241 Cardiologie, Hôpital Bichat-Claude Bernard (AP-HP), Paris, France; 242 Anesthésie réanimation chirurgie cardio-vasculaire, Hôpital Bichat-Claude Bernard (AP-HP), Paris, France; 243 Chirurgie cardiaque et vasculaire, Hôpital Bichat-Claude Bernard (AP-HP), Paris, France; 244 93, Hôpital Avicenne, Bobigny, France; 245 Réanimation polyvalente adulte, Centre Hospitalier Intercommunal André Grégoire, Montreuil, France; 246 Réanimation médicale, C.H. Intercommunal Poissy/Saint-Germain-en-Laye, Poissy Cedex, France; 247 Réanimation polyvalente, Faculté de médecine de Sfax, Sfax, Tunisia; 248 Réanimation polyvalente, CHU Habib Bourguiba, Sfax, Tunisia; 249 Réanimation médicale, CHU BICHAT, Paris, France; 250 Virology, Hospital Center Regional University, Pointe-a-Pitre, Guadeloupe; 251 Centre de recherche de l’iucpq, Institut Universitaire de Cardiologie et de Pneumologie de Québec, Québec, Canada; 252 Réanimation chirurgicale polyvalente, Hôpital Bichat-Claude Bernard (AP-HP), Paris, France; 253 Réanimation CTCV Transplantation thoracique, CHU de Nantes - Hôpital Nord Laennec, Saint-Herblain, France; 254 Réanimation médicaleBd Tanguy Prigent, Brest, France; 255 DEPM, Transfusion Research Unit, Monash University, Melbourne, Australia; 256 DEPM, Monash University, ANZIC-RC, Melbourne, Australia; 257 Intensive Care Unit, Alfred Hospital, Melbourne, Australia; 258 Intensive Care Unit, Royal Prince Alfred Hospital, Sydney, Australia; 259 Intensive Care Unit, Royal Prince Alfred Hospital, Camperdown, Australia; 260 Anesthesiology, Alfred Hospital, Prahran, Australia; 261 Haematology, Alfred Hospital, Prahran, Australia; 262 Département d’anesthésie et de réanimation, UMRS INSERM 1166, Hôpital Universitaire La Pitié-Salpêtrière, Université Pierre et Marie Curie, IHU ICAN, Paris, France; 263 Réanimation chirurgicale cardiovasculaire et thoracique, Groupe Hospitalier Pitié-Salpêtrière, Paris, France; 264 Service d’anesthésie et de réanimation, Institut de cardiologiepitié salpetrière hospital, Faculté de Médecine Pierre et Marie Curie, Paris, France; 265 Intensive care medicine, UZ Leuven, Louvain, Belgium; 266 Anesthesia & Intensive Care, Jessa Ziekenhuis, Campus Virga Jesse, Hasselt, Belgium; 267 Anaesthesiology & Intensive Care, Ziekenhuis Oost-Limburg a.v., Genk, Belgium; 268 Iame team 5, INSERM UMR 1137, Paris, France; 269 Equipe 11, Institut Albert Bonniot - Inserm U823, La Tronche, France; 270 Hygiène hospitalière, Hôpital Bichat-Claude Bernard (AP-HP), Paris, France; 271 Réanimation, Hospices Civils De Lyon, Lyon, France; 272 Médecine interne, Hôpital Bichat-Claude Bernard (AP-HP), Paris, France; 273 Hygiène hospitalière, C.H.U de Rennes, Rennes, France; 274 Pharmacie, CHU - Hôpitaux de Bordeaux, Bordeaux, France; 275 Réanimation, CHRU Nancy, Nancy, France; 276 DGOS, Ministère des Affaires sociales et de la Santé, Paris, France; 277 Cclin sud est, Hospices Civils De Lyon, Lyon, France; 278 Cclin, Hospices Civils De Lyon, Lyon, France; 279 Immuno-hématologie, Hôpital Saint-Louis (AP-HP), Paris, France; 280 Radiologie, Hôpital Saint-Louis, Paris, France; 281 Chirurgie viscérale, Hôpital Saint-Louis, Paris, France; 282 Reanimation médicale, Hôpital Saint-Louis (AP-HP), Paris, France; 283 Sbim, Assistance Publique Hôpitaux de Paris, Paris, France; 284 Réanimation, Institut Paoli-Calmettes, Marseille, France; 285 Réanimation polyvalente, Centre Hospitalier de Roubaix, Roubaix, France; 286 Réanimation pneumologique, Hôpital Cochin, Paris, France; 287 Réanimation polyvalente, C.H.U. de Nancy, Nancy, France; 288 Réanimation, Institut Jules Bordet, Brussels, Belgium; 289 Réanimation polyvalente, Hopital universitaire, Gand, Belgium; 290 Réanimation médicale, C.H.U., La Tronche, La Tronche, France; 291 Service de réanimation médicale, INSERM UMRS_999, université paris-sud, Hôpital de bicêtre, hôpitaux universitaires paris-sud, Assistance publique – Hôpitaux de Paris, Le Kremlin-Bicêtre, France; 292 Departement d’anesthésie-réanimation, Hôpital Maison Blanche, Reims Cedex, France; 293 Réanimation, Centre Hospitalier Général de Saint-Denis, Saint-Denis, France; 294 Réanimation médico-chirurgicale, Hôpital Tenon -APHP, Paris, France; 295 Département de réanimation médicale et de médecine hyperbare, C.H.U. d’Angers, Angers, France; 296 Service de réanimation médico-chirurgicale, Centre Hospitalier de Versailles, Le Chesnay, France; 297 Service de réanimation polyvalente, Centre Hospitalier Bretagne Atlantique, Vannes, France; 298 Service de réanimation polyvalente, Centre Hospitalier Notre-Dame de la Miséricorde, Ajaccio, France; 299 Service de réanimation polyvalente, Hospital Foch, Suresnes, France; 300 Service de réanimation polyvalente, Hôpital d’instruction des armées Desgenettes, Lyon, France; 301 Service de réanimation polyvalente, Centre Hospitalier Général de Saint-Denis, Saint-Denis, France; 302 Service de médecine interne, Groupe Hospitalier La Pitié-Salpêtrière, Institut IE3M, Assistance Publique Hôpitaux de Paris, Paris, France; 303 Hôpital Bicêtre (AP-HP), Le Kremlin-Bicêtre, France; 304 Val de marne, Hôpital Bicêtre (AP-HP), Le Kremlin-Bicêtre, France; 305 Service d’anesthésie et de réanimation, institut de cardiologie, Pitié-Salpêtrière Hospital, Paris, France; 306 Centre d’investigation clinique paris est 1421, département de pharmacologie, Hôpital Universitaire La Pitié-Salpêtrière, Université Pierre et Marie Curie, IHU ICAN, Paris, France; 307 Unité de microbiologie clinique et dosages des anti-infectieux, Groupe hospitalier Paris Saint-Joseph, Paris, France; 308 Microbiologie, Hôpital Saint-Antoine, Paris, France; 309 Réanimation Médicale, Hôpital Saint-Antoine, AP-HP, Paris, France; 310 Réanimation Polyvalente, Hôpital Saint Joseph Saint Luc, Lyon, France; 311 Pharmacie, Hôpital Saint Joseph Saint Luc, Lyon, France; 312 Biologie, Hôpital Saint Joseph Saint Luc, Lyon, France; 313 Département d’information médicale, Hôpital Saint Joseph Saint Luc, Lyon, France; 314 Unité de prévention et lutte contre les infections nosocomiales (uplin), CHU Angers, Angers, France; 315 Hygiène hospitalière, Hospices Civils de Lyon, Lyon, France; 316 Laboratoire mesurs, Conservatoire National des Arts et Métiers, Paris, France; 317 Microbiologie, Centre Hospitalier de Dieppe, Dieppe, France; 318 Service de réanimation médicale brabois, CHRU de Nancy, Vandœuvre-Lès-Nancy, France; 319 Service de microbiologie, CHRU de Nancy, Vandœuvre-Lès-Nancy, France; 320 Service de maladie infectieuse et tropicale, CHRU de Nancy, Vandœuvre-Lès-Nancy, France; 321 Service de réanimation chirurgicale brabois, CHRU de Nancy, Vandœuvre-Lès-Nancy, France; 322 Centre d’investigation clinique pierre drouin, CHRU de Nancy, Vandœuvre-Lès-Nancy, France; 323 Cardiologie - centre d’investigation clinique pierre drouin, CHRU de Nancy, Vandœuvre-Lès-Nancy, France; 324 Réanimation Médicale Brabois, Centre Hospitalier Universitaire de Nancy, Vandœuvre-Lès-Nancy, France; 325 Réanimation polyvalente, CHU Fattouma Bourguiba, Monastir, Tunisia; 326 Department of Critical Care Medicine and Anesthesiology, Military Hospital of Tunis, Tunis, Tunisia; 327 Faculté de médecine de Sfax, Sfax, Tunisia; 328 Hopital régional mahres, Faculté de médecine de Sfax, Sfax, Tunisia; 329 Intensive Care, hopital régional Gafsa, Sfax, Tunisia; 330 Intensive Care, hopital régional mahres, Sfax, Tunisia; 331 URGENCES, Centre Hospitalier Général de Longjumeau, Longjumeau, France; 332 Samu, C.H. Sud Francilien, Corbeil-Essonnes, France; 333 Reanimation des urgences chirurgicale, Chu Ibn Rochd, Casablanca, Morocco; 334 Calvados, Pôle Anesthésie-Réanimation C.H.U de Caen, Caen, France; 335 Calvados, INSERM U919, Cyceron, Caen, France; 336 Calvados, Neurochirurgie, C.H.U de Caen, Caen, France; 337 Anesthésie réanimationCHU Ibn Rochd Casa, Casablanca, Morocco; 338 Service de réanimation pédiatriqueChu Ibn Rochd, Casablanca, Morocco; 339 FinistereC.H. de Cornouaille, Quimper, France; 340 VAR, Hospital Sainte Musse, Toulon, France; 341 Réanimation polyvalente, Hôpital Sainte-Musse, Toulon, France; 342 Anesthésie, Centre Hospitalier Universitaire de Caen, Caen, France; 343 Réanimation Médicale, Hôpital Henri Mondor, Avenue du Maréchal de Lattre de Tassigny, Créteil, France; 344 Réanimation Médicale, Hopital regional zaghouan, faculté de médecine de Tunis, Zaghouan, Tunisia; 345 Teaching Department of Emergency and Intensive Care, Regional Hospital of Zaghouan, Zaghouan, Tunisia; 346 91, C.H. Bligny, Briis-Sous-Forges, France; 347 Réanimation Médicale, Teaching Department of Emergency and Intensive Care, Hospital of Zaghouan, Zaghouan, Tunisia; 348 Service de pneumologie et réanimation médicale, Groupe Hospitalier Pitié Salpêtrière, Paris, France; 349 Department of Informatics and Biostatistics, Saint Louis Hospital, Paris, France; 350 Service de réanimation adultes, C.H.U. Estaing, Clermont-Ferrand, France; 351 Réanimation Polyvalente, CHU Fatouma Bourguiba, Monastir, Tunisia; 352 Réanimation médicale, CHU Farhat Hached, Sousse, Tunisia; 353 Department of Intensive Care and Toxicology, Centre d’Assistance Médicale Urgente, Tunis, Tunisia; 354 Réanimation médicale, Research laboratory n° lr14es05, Faculty of medicine, CHU Farhat Hached, Sousse, Tunisia; 355 Réanimation médico-chirurgicale, Assistance Publique - Hôpitaux de Paris, Hôpital Ambroise Paré, Boulogne-Billancourt, France; 356 Réanimation médicale, Centre Hospitalier Universitaire, Amiens, France; 357 Réanimation médicale, Centre hospitalier universitaire la Rabta de Tunis, Tunis, Tunisia; 358 Réanimation, Chru De Nîmes, Nîmes, France; 359 Réanimation médicale, CHU Hôpital l’Archet 1, Route Saint-Antoine de Ginestière, Nice, France; 360 Réanimation médicale, Hôpitaux de l’Archet 1 et 2, Nice, France; 361 Service de réanimation, Hôpital l’Archet 2, Nice, France; 362 Réanimation Médicale, EPS Taher Sfar Mahdia, Mahdia, Tunisia; 363 Service de réanimation, Hôpital d’Instruction des Armées Percy, Clamart, France; 364 Antenne médicale, Centre médical des armées, Besançon, France; 365 Centre de traitement des brûlés, Hôpital d’Instruction des Armées Percy, Clamart, France; 366 Unité de recherche, Centre d’épidémiologie et de santé publique des armées, Marseille, France; 367 Département de neurosciences et contraintes opérationnelles, Institut de Recherche Biomédicale des Armées, Brétigny-Sur-Orge, France; 368 Réanimation neurochirurgicale pédiatrique, Hôpital Necker - Enfants Malades, Paris, France; 369 Réanimation et surveillance continue médico-chirurgicales, Hôpital Necker - Enfants Malades, Paris, France; 370 Réanimation polyvalente, Hospital Antoine Béclère, Clamart, France; 371 Pôle de réanimation, hôpital salengro, C.H.R.U. - Lille, Avenue Oscar Lambret, Lille, France; 372 73, Centre hospitalier Métropole Savoie, Chambéry, France; 373 Giga la vis, Institut des Hauts de Seine, Nanterre, France; 374 Service de pneumologie et réanimation, Hôpital Cochin, Paris, France; 375 Laboratoire ilumens, Universisté René Descartes - Paris V, Paris, France; 376 Intensive Care Unit, University Hospital of Martinique, Fort-De-France, France; 377 Centre Hospitalier Universitaire de Fort de France, Fort-De-France, Martinique France; 378 Cardiology Department, University Hospital of Martinique, Fort-De-France, France; 379 Réanimation Polyvalente, Centre Hospitalier Universitaire de Fort de France, Fort-De-France, Martinique; 380 Infectious and Tropical Diseases Department, University Hospital of Martinique, Fort-De-France, France; 381 Department of Neurology, University Hospital of Martinique, Fort-De-France, France; 382 Medical Intensive Care, Hôpital Cochin, Paris, France; 383 Unité 1016, 22 rue méchain, Institut National de la Santé et de la Recherche Médicale, Paris, France; 384 U1016, Institut National de la Santé et de la Recherche Médicale, 22 rue méchain, Paris, France; 385 Haematology, Hôpital Cochin, Paris, France; 386 INSERM U1096, Université de rouen, Rouen, France; 387 Pôle Réanimations Anesthésie SAMU, Hospital Center University Rouen, Rouen, France; 388 U1096, INSERM, Rouen, France; 389 Inserm u995 equipe 4, Université Lille 2, Lille, France; 390 Réanimation médicale, Hospital Center University Rouen, Rouen, France; 391 U1116, Faculté de Médecine de Nancy, Vandœuvre-Lès-Nancy, France; 392 Unité espri-biobase, CHRU Nancy, Vandœuvre-Lès-Nancy, France; 393 Service de biochimie, CHRU de Nancy, Vandœuvre-Lès-Nancy, France; 394 URC, Hospital Ambroise Paré, Boulogne-Billancourt, France; 395 Laboratoire d’hématologie, Hopital Robert Debré, Reims, France; 396 Laboratoire d’hémostase, cytologie et plateforme de biomonitoring, EFS Bourgogne Franche-Comté, Besancon, France; 397 Anesthésie Réanimation SAMUAPHP - CHRU Necker Enfants Malades, Paris, France; 398 Service de pharmacologie toxicologie, APHP - Hopital Raymond Poincaré, Paris, France; 399 Assistance Publique Hôpitaux de Paris, Paris, France; 400 Service de réanimation adulte, Hôpital Necker, Assistance Publique Hôpitaux de Paris, Paris, France; 401 Réanimation adulte - samu, Hôpital Necker - Enfants Malades, Paris, France; 402 SAMU de ParisRéanimation polyvalente, 75014 Paris, France; 403 Inserm u1144, Paris-Descartes University, Paris, France; 404 Department of Medical and Toxicological Critical Care, Lariboisière Hospital, Paris, France; 405 Laboratory of Toxicology, Lariboisière Hospital, Paris, France; 406 Department of Emergency, Centre d’Assistance Médicale Urgente, Tunis, Tunisia; 407 Departement of Intensive Care and Toxicology, Centre d’Assistance Médicale Urgente, Tunis, Tunisia; 408 Intensive Care Unit, Regional Hospital of Ben Arous, Tunis, Tunisia; 409 Peadiatric Intensive Care Unit, Children Hospital of Tunis, Tunis, Tunisia; 410 Unité de recherche ur12sp10, université tunis el manar, Peadiatric Intensive Care Unit, Children Hospital of Tunis, Tunis, Tunisia; 411 Neonatal and Paediatric Intensive Care, Chu de poitiers, Poitiers, France; 412 Infection Prevention Team, Chu de poitiers, Poitiers, France; 413 Laboratory of Bacteriology, Chu de poitiers, Poitiers, France; 414 PEDIATRIE, Hôpital Femme Mère Enfant, Bron, France; 415 Réanimation Pédiatrique, Groupement Hospitalier Est-Hôpital Femme Mère Enfant, Bron, France; 416 Réanimation pédiatrique, Hôpital Femme Mère Enfant, Bron, France; 417 Pôle information médicale evaluation recherche, equipe d’accueil 4129, hospices civils de Lyon, Lyon, France; 418 Réanimation pédiatrique canastel, Faculté de médecine d’Oran, Oran, Algeria; 419 Anesthésie réanimation pédiatrique, Etablissement hospitalier spécialisé en pédiatrie Canastel, Oran, Algeria; 420 Réanimation pédiatrique de Canastel d’oran, Departement de medecine d’Oran Algerie, Oran, Algeria; 421 Réanimation pédiatrique, EHS CANASTEL, Oran, Algeria; 422 Anesthésie-Réanimatiion, CHU Abdelkader Hassani, Sidi Bel Abbès, Algeria; 423 Urgences médico-chirurgicales, CHU Abdelkader Hassani, Sidi Bel Abbès, Algeria; 424 Réanimation, Centre hospitalier Métropole Savoie, Chambéry, France; 425 Réanimation, Hôpital d’Instruction des Armées Percy, Clamart, France; 426 Département d’anesthésie-réanimation, Hôpital d’Instruction des Armées Percy, Clamart, France; 427 Medical ICU, Hospital Abderrahmen Mami De Pneumo-Phtisiologie, Ariana, Tunisia; 428 Réanimation médicale, Hôpital Abderrahmen Mami, Ariana, Tunisia; 429 Réanimation et surveillance continue pédiatriques, Hôpital Necker, Rue de Sèvres, Paris, France; 430 Réanimation néonatale et pédiatrique, CHU Kremlin-bicêtre, Le Kremlin-Bicêtre, France; 431 Réanimation pédiatrique polyvalente, Hôpital Necker - Enfants Malades, Paris, France; 432 Unité u1141, INSERM-Hôpital Robert Debré, Paris, France; 433 Pediatric Intensive Care Unit, CHU Lille, 59000 Lille, France; 434 Department of Biostatistics, CHU Lille, 59000 Lille, France; 435 Univ. Lille, ea 2694 - santé publique : épidémiologie et qualité des soins, C.H. Régional Universitaire de Lille (CHRU de Lille), 59000 Lille, France; 436 Physioflow, Manatec Biomedical, 78300 Poissy, France; 437 Unité de recherche ur12sp10. université tunis el manar, Service de réanimation pédiatrique polyvalente, Hôpital d’enfants Béchir Hamza de Tunis, Tunis, Tunisia; 438 Réanimation polyvalente, Hospital Children, Tunis, Tunisia; 439 Inserm u1141, Hôpital Robert Debré, Paris, France; 440 Pharmacologie de la circulation cérébrale, INSERM EA4475, Faculté de Pharmacie, University Paris Descartes, Paris, France; 441 Inserm nps, Université UPMC, Paris, France; 442 Anesthésie-réanimation pédiatrique, Hopital de la Timone, Marseille, France; 443 Centre de référence des hernies diaphragmatiques, Hôpital de la Timone, Marseille, France; 444 Cardiologie pédiatrique, Hôpital de la Timone, Marseille, France; 445 Chirurgie cardiaque pédiatrique, Hôpital de la Timone, Marseille, France; 446 Anesthésie réanimation, Hôpital Louis Pradel, Bron, France; 447 Anesthésie, Hôpital Femme Mère Enfant, Bron, France; 448 Pédiatrie, Centre Hospitalier de Roubaix, Roubaix, France; 449 Department of allergology, Emile Durkheim Hospital, Épinal, France; 450 Department of epidemiology (cepidc), National Mortality Center, Le Kremlin-Bicêtre, France; 451 Pediatric Pulmonology and Allergy Department, C.H. Régional Universitaire de Lille (CHRU de Lille), Lille, France; 452 Pharmacy Department, CHU Farhat Hached, Sousse, Tunisia; 453 Anesthésie réanimation chirurgicale, EHU 1er Novembre, Oran, Algeria; 454 Réanimation pédiatrique, Centre Hospitalier et Universitaire d’Oran, Oran, Algeria; 455 Service de gynéco-obstétrique, EHS 1er Novembre, Oran, Algeria; 456 Anesthesie-réanimation chirurgicale, EHS 1ER NOVEMBRE, Oran, Algeria; 457 Cardiologie, Centre Hospitalier et Universitaire d’Oran, Oran, Algeria; 458 Department of Biostatistics, Lariboisière Hospital, Paris, France; 459 Laboratory of Biochemistry, HEGP, Paris, France; 460 Réanimation médicale bizerte, Faculté de médecine de Tunis, Bizerte, Tunisia; 461 Service de Réanimation Médicale et Toxicologique, CHU Lariboisière, Paris, France; 462 Reanimation, EHS CANASTEL, Oran, Algeria; 463 Service d’anesthésie réanimation, Centre Hospitalier Universitaire de Caen, Caen, France; 464 Service d’explorations fonctionnelles respiratoire, Centre Hospitalier Universitaire de Caen, Caen, France; 465 Emergency Medicine and Critical Care, CHU de Besançon, Besançon, France; 466 Inserm cic 1431, CHU de Besançon, Besançon, France; 467 Emergency Medicine and Critical Care, Hôpital jean Minjoz, Besançon, France; 468 Réanimation médicale, CHRU de Brest, Brest, France; 469 Urgences, CHU de Poitiers, Poitiers, France; 470 CHU Gabriel-Montpied, Clermont-Ferrand, France; 471 Anesthesia and Intensive Care Medicine, Centre Hospitalier Louis Pradel, Lyon, France; 472 Anesthesia and Intensive Care Medicine, CHU Henri Mondor, Créteil, France; 473 Reanimation polyvalente, Hôpital Civil Marie Curie, Charleroi, Belgium; 474 Réanimation Polyvalente, Centre Hospitalier De Valenciennes, Valenciennes, France; 475 Cic 1433 épidémiologie et clinique, C.H.U. de Nancy, Nancy, France; 476 Santé publique:épidémiologie et qualité des soinsservice de biostatistiques, CHRU de Lille, Lille, France; 477 Biostatistiques, C.H. Régional Universitaire de Lille (CHRU de Lille), Lille, France; 478 Pédiatrie, C.H. de Cambrai, Cambrai, France; 479 Pédiatrie, C.H. de Lens, Lens, France; 480 Pédiatrie, C.H. d ‘Arras, Arras, France; 481 Pédiatrie, C.H. de Boulogne-sur-Mer, Boulogne-Sur-Mer, France; 482 Pédiatrie, C.H. de Dunkerque, Dunkirk, France; 483 Pédiatrie, C.H. de Douai, Douai, France; 484 Santé publique:épidémiologie et qualité des soinsservice de gestion du risque infectieux, CHRU de Lille, Lille, France; 485 Service de réanimation et surveillance continue médico-chirurgicale pédiatrique, Hôpital Necker - Enfants Malades, Paris, France; 486 Service de pédiatrie générale, Hôpital Necker - Enfants Malades, Paris, France; 487 Pneumology, Hospital Necker, Paris, France; 488 Réanimation néonatale, Hôpital Necker - Enfants Malades, Paris, France; 489 Isère, Hôpital couple enfant, Grenoble, France; 490 Reanimation pediatrique, C.h.u., La Tronche, La Tronche, France; 491 Réanimation médicale, CHU, Nantes, France; 492 Microbiologie Laboratory, Hôpital Abderrahmen Mami, Ariana, Tunisia; 493 Service de Réanimation Pneumologique, CHU Amiens - Hôpital Sud, Amiens, France; 494 Service de réanimation polyvalente, Centre hospitalier intercommunal Robert Ballanger, Aulnay-Sous-Bois, France; 495 Réanimation cardio thoracique et vasculaire, CHU Amiens-Picardie, Amiens, France; 496 Réanimation chirurgicale, Cochin Port-Royal, Paris, France; 497 Département de biostatistiques, santé publique et information médicale, Pitié-Salpêtrière Hospital, Paris, France; 498 Réanimation chirurgicale polyvalente, Hôpital de la Pitié-Salpêtrière, Paris, France; 499 Service d’information médicaled’épidémiologie et d’économie de la santé, CHRU Hôpitaux de Tours, Tours, France; 500 ICU, CHU Habib Bourguiba, Sfax, Tunisia; 501 Reanimation polyvalente, CHU Habib Bourguiba, Sfax, Tunisia; 502 Medical Informatics Laboratory, Chu Ibn Rochd, Casablanca, Morocco; 503 Intensive Care Unit and Anesthesiology, Hôpital d’Instruction des Armées Sainte-Anne, Toulon, France; 504 Thoracic Surgery Department, Hôpital d’Instruction des Armées Sainte-Anne, Toulon, France; 505 Burn Care Department, Trauma and Burn Center, Tunis, Tunisia

## Physician abstracts

### Oral communications

#### O1 Pulmonary embolism related sudden cardiac arrest admitted alive at hospital: characteristics and outcomes

##### Wulfran Bougouin^1^, Eloi Marijon^1^, Benjamin Planquette^2^, Nicole Karam^1^, Florence Dumas^3^, David Celermajer^4^, Daniel Jost^5^, Lionel Lamhaut^6^, Frankie Beganton^7^, Alain Cariou^8^, Guy Meyer^9^, Xavier Jouven^10^, Sudden Death Expertise Center

###### ^1^Cardiologie, Hôpital Européen Georges-Pompidou, Rue Leblanc, Paris, France; ^2^Usip, Hopital Europeen Georges-Pompidou, Paris, France; ^3^Service d’accueil des urgences, Hôpital Cochin, Paris, France; ^4^Cardiology, Sydney medical school, Sydney, Australia; ^5^Bspp, B.s.p.p., Paris, France; ^6^Réanimation adulte, Hôpital Necker - Enfants Malades, Paris, France; ^7^Paris descartes, Inserm U970, Paris, France; ^8^Réanimation Médicale, Hôpital Cochin, Paris, France; ^9^Service de Pneumologie et Soins Intensifs, Hopital Europeen Georges-Pompidou, Paris, France; ^10^Cardiologie, Hôpital Européen Georges-Pompidou, Paris, France

####### **Correspondence:** Wulfran Bougouin - wulfran.bougouin@gmail.com

*Annals of Intensive Care* 2017, **7(Suppl 1)**:O1

**Introduction** Pulmonary embolism (PE) is a relatively common cardiovascular condition, occasionally and tragically manifesting as sudden cardiac arrest (SCA). The natural history of SCA complicating PE has been poorly evaluated. Guidelines suggest the consideration of thrombolytic therapy when PE-related SCA is suspected, despite the absence of evidence. In this study, we described the characteristics and management of PE-related SCA in a large regional registry.

**Patients and methods** In this prospective population-based study, we included all patients admitted at hospital alive after out-of-hospital SCA, in Paris and suburbs, France (6.6 million inhabits), from May 2011 to September 2015. Regarding PE, we collected risk factors, clinical decision rules (Wells rule and Geneva score) and diagnostic strategy.

**Results** Of 2926 patients hospitalized after SCA, 82 cases were diagnosed as PE-related SCA (2.8%, 95% CI 2.2–3.4). Independent factors associated with SCA due to PE were non-shockable initial rhythm (OR 12.4, 95% CI 4.9–31.0, *P* < 0.001), past history of thromboembolism (OR 10.4, 95% CI 5.6–19.4, *P* < 0.001), absence of known heart disease (OR 3.8, 95% CI 2.0–7.3, P < 0.001) and female sex (OR 1.9, 95% CI 1.2–3.0, P = 0.008). Considering non-shockable initial rhythm and previous thromboembolism as major predictors of PE, combination of those factors had a specificity for detection of PE-related SCA of 98% and a sensitivity of 23%, with a positive predictive value of 31% and a negative predictive value of 98% (Fig. 1) .

**Fig. 1:**
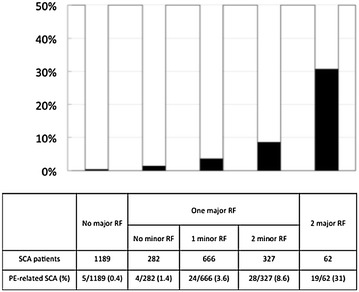
See text for description

Systemic thrombolysis was performed in 47 patients (57%). After adjustment, PE was associated with survival at discharge (OR 2.4, 95% CI 1.2–4.7, P = 0.001), compared with non-PE SCA. Finally, among patients hospitalized for PE-related SCA, only thrombolysis (OR 12.5, 95% CI 1.8–89.1, P = 0.01) and delay from CPR to ROSC < 20 min (OR 6.8, 95% CI 1.3–35.2, P = 0.02) were independently associated with survival to hospital discharge.

**Conclusion** In this population-based study, PE was not an unusual cause of SCA, and was associated with better survival, challenging the traditional view. Thrombolysis was associated with an increased survival in this population, reinforcing current guidelines.

**Competing interests** None.

#### O2 Eligibility for and feasibility of donation after circulatory death Maastricht III (DCD MIII) process in post-anoxic patients: a retrospective analysis

##### Côme Bureau^1^, Julien Charpentier^1^, Omar Ben Hadj Salem^1^, Lucie Guillemet^1^, Michel Arnaout^1^, Matthieu Jamme^1^, Alexis Ferre^1^, Guillaume Geri^1^, Florence Dumas^1^, Nicolas Mongardon^1^, Frédéric Pène^1^, Jean-Daniel Chiche^1^, Jean-Paul Mira^1^, Alain Cariou^1^

###### ^1^Réanimation médicale, Hôpital Cochin, Paris, France

####### **Correspondence:** Côme Bureau come.bureau@gmail.com

*Annals of Intensive Care* 2017, **7(Suppl 1)**:O2

**Introduction** Donation after circulatory death corresponds to the category III of the Maastricht classification (DCDM III) and may provide mostly kidney and liver transplants with good long-term function. Patients suffering from irreversible brain damages after cardiac arrest are commonly considered candidates for DCDMIII, but little is known regarding the proportion of these patients who could be eligible for this procedure. Using a cohort of post-cardiac arrest patients, our aim was to assess the rate of contra-indications for DCDMIII and to measure the delay between withdrawal of life sustaining treatments (LSTW) and the appearance of low values for common physiological parameters during the agonal phase, which may compromise the process by altering graft function.

**Patients and methods** Using the Cochin registry (Paris, France), we conducted a retrospective single-centre study from January 2007 to December 2014. We included all patients who died in ICU after LSTW decision because of post-anoxic brain damages. For each patient, we collected exclusion criteria for DCDMIII and the length of time between LSTW implementation and death. We also collected hemodynamic and respiratory parameters during the agonal phase.

**Results** We included 404 patients in the study, of whom 275 (68%) had at least one exclusion criteria for a DCDMIII process, mostly because of age >65 (190 patients). Other exclusion criteria were: multiple organ failure (n = 88), neoplastic diseases (n = 55, including 46 solid tumours), brain-dead state that occurred after LSTW decision (n = 18), unknown cause of the initial cardiac arrest (n = 13), chronic viral diseases (n = 13), uncontrolled sepsis (n = 4), occurrence of a new refractory cardiac arrest (n = 2), and judicial problems (n = 3).

The 130 potentially eligible patients for DCDMIII included 94 men (72%) with a mean age of 51 years (±7.7). At time of death after LSTW, the mean length of stay in ICU was 11.6 days (±6). The most common aetiology of cardiac arrest was acute myocardial ischemia (n = 59, 45%). LSTW consisted in terminal weaning of mechanical ventilation in 71 patients (55%), extubation in 12 patients (9%) and infusion of vasopressors was stopped in 3 patients (2%).

The average duration of the agonal phase (time between LSTW implementation and death) was 746 min (min) (±162) and this delay was >180 min in 92 patients (71%). After LSTW implementation, an oxygen transcutaneous saturation (SpO_2_) <70% occurred in 637 min (±545), a mean arterial pressure (MAP) <60 mmHg in 723 min (±586) and a systolic arterial pressure (SAP) <50 mmHg in 733 min (±596). The delay between SpO_2_ < 70% and death was 154 min (±262), and this delay was 59 min (±160) after MAP < 60 mmHg and 23 min (±134) after SAP < 50 mmHg.

**Conclusion** In this large cohort of brain damaged patients with LSTW decision, we observed that a high proportion of patients would not have been eligible for a DCDMIIII process. Even in those without contra-indication, the delay between LSTW implementation and the final circulatory arrest was not compatible with French national guidelines. Low values for arterial pressure and oxygenation persisted during a substantial part of time before final circulatory arrest. This information may help in refining the management of the DCDMIII process in this population.

**Competing interests** None.

#### O3 Evaluation of the prognostic value of the bispectral index (BIS) and suppression ratio (RS) among patients admitted to the ICU for cardiac arrest

##### Guylaine Labro^1^, Francois Belon^1^, Vinh-Phuc Luu^2^, Julien Chenet^3^, Guillaume Besch^4^, Marc Puyraveau^2^, Gaël Piton^1^, Gilles Capellier^1^

###### ^1^Réanimation médicale, CHU de Besançon, Besançon, France; ^2^Centre de méthodologie clinique, CHU de Besançon, Besançon, France; ^3^Département d’anesthésie et de réanimation, CHU de Besançon, Besançon, France; ^4^Réanimation chirurgicale, CHU de Besançon, Besançon, France

####### **Correspondence:** Guylaine Labro - guylainelabro@hotmail.fr

*Annals of Intensive Care* 2017, **7(Suppl 1)**:O3

**Introduction** Predicting the neurological outcome of patients admitted to the ICU after a cardiac arrest successfully resuscitated remains difficult [1]. The bispectral index (BIS) allows for the rapid and standardised assessment of the cortical function based upon eletroencephalogram analysis whereas the ratio of suppression (RS) is indicative of the absence of electrical activity of the brain. We aimed to evaluate the prognostic value of the BIS and the RS for predicting neurologic outcome after cardiac arrest.

**Patients and methods** This was a prospective, single center, observational study performed in a large regional University hospital. Adult patients admitted to the ICU for cardiac arrest between March 2012 and October 2014 were included in the study. The exclusion criteria was pregnancy. The BIS and the RS were collected as soon as possible after ICU admission. The patients were not included in the analysis if they died within 24 h, if they had a low signal quality [defined as high EMG artefacts (≥30 dB)], or if the monitoring of BIS started 24 h after ICU admission. The neurological outcome of the patients was based upon the cerebral performans category (CPC) calculated at 3 months. CPC score of 1 or 2 indicated good outcome, whereas CPC score of 3–5 indicated poor outcome.

**Results** During the study period 148 patients were admitted to our ICU for a cardiac arrest. The BIS and RS were monitored in 103 patients.17 patients were excluded (early death ≤24 h; low quality of signal; BIS and RS performed ≥24 h after ICU admission). Thus, 86 patients were enrolled in this study. The means age was 57.6 ± 16.8 years, 61 patients (70.9%) were male, the cardiac arrest was out-of-hospital in 63 patients (73.3%), hypoxia was the main cause of cardiac arrest (43%), 60 patients (70.6%) were treated with therapeutic hypothermia. At 3 month of follow-up, a total of 50 patients (58.1%) had died and 55 patients (63.9%) were classified as having a poor outcome. The mean duration from the return of spontaneous circulation (ROSC) to the BIS and RS measurements was 5.7 ± 3.0 h. The BIS values were significantly lower in patient with poor outcome compared with patients with a good outcome (5.9 ± 11.1 vs 37.1 ± 18.0, *p* < 0.0001). The RS values were significantly higher in patient with poor outcome group compared to those with good neurological outcome (85.9 ± 26.3 vs 18.4 ± 31.3, *p* < 0.0001). The BIS predicted poor outcome with a likelihood ratio of 23.8 and an area under the curve (AUC) of 0.918 [95% CI (0.839–0.966)]. The optimal sensitivity [78.4%, 95% CI (67.3–89.5)] and specificity [96.5%, 95% CI (89.8–100)] for neurological outcome prediction was obtained using a cut-off value of BIS < 5. The RS predicted poor outcome with a likehood ratio of 23.8 and an AUC of 0.936 [95% CI (0.862–0.977)]. The optimal sensitivity [78.0%, 95% CI (66.9–89.0)] and specificity [96.8%, 95% CI (90.8–100)] for neurological outcome prediction was obtained using a cut-off value of RS > 84. In multivariable logistic regression model, BIS or RS predicted poor outcome with an odds ratio of 65.0 [95% CI (6.1–689.2), *p* = 0.0005].

**Discussion** The results of this study using the EEG derived parameters BIS and RS confirm previous findings showing that they are linked to the neurological outcome of patients admitted to the ICU after a cardiac arrest [2]. In particular, a BIS < 5 and/or a RS > 84, measured at ICU admission, were both strongly associated with a poor neurological outcome at 3-months.

**Conclusion** BIS values may be used to predict long term neurological outcome of patients following cardiopulmonary resuscitation. The ability to accurately predict early non-recovery after cardiac arrest could facilitate discussions with families and limit use of ICU resources in futile cases. BIS and RS values measured at ICU admission might be considered as additional prognostic tools available for the intensivist.

**Competing interests** None.

**References**

1. Sandroni C. Neurological prognostication after cardiac arrest. Curr Opin Crit Care. 2015;21(3):209–14.
2. Seder David B. The bispectral index and suppression ratio are very early predictors of neurological outcome during therapeutic hypothermia after cardiac arrest. Int Care Med. 2010;36:281–88.

#### O4 Nutrition during targeted temperature management after cardiac arrest: observational study of neurological and infectious outcomes

##### Maëlle Martin^1^, Jean-Baptiste Lascarrou^1^, Aurélie Le Thuaut^2^, Jean-Claude Lacherade^1^, Laurent Martin-Lefèvre^1^, Maud Fiancette^1^, Isabelle Vinatier^1^, Christine Lebert^1^, Konstantinos Bachoumas^1^, Aihem Yehia^1^, Matthieu Henry-Laguarrigue^1^, Gwenhaël Colin^1^, Jean Reignier^3^

###### ^1^Intensive Care, District Hospital Center, La Roche-sur-Yon, France; ^2^Clinical Research Unit, District Hospital Center, La Roche-sur-Yon, France; ^3^Medical Intensive Care Unit, University Hospital Center, Nantes, France

####### **Correspondence:** Maëlle Martin - maellemart1@gmail.com

*Annals of Intensive Care* 2017, **7(Suppl 1)**:O4

**Introduction** Cardiac arrest represents one of the greatest medical challenges because of its terrible mortality, morbidity and cost. International guidelines for cardiopulmonary resuscitation led to survival and prognosis improvement, based on the Chain of Survival and the Advanced Life Support, including Targeted Temperature management (TTM) [1]. Although routinely used, guidelines do not provide detailed management of patients with TTM, especially how to adapt associated therapies such as sedation or nutrition. Guidelines encourage early nutrition for intensive care patients, within 24–48 h after admission [2]. Nevertheless, after cardiac arrest, early nutrition (EN) is disputed. Common post-cardiac arrest syndrome with circulatory failure, frequent diarrheic collapse; and supposed lower digestive tolerance in hypothermia do not encourage this practice.

This study first aims to determine if EN is associated with better neurological outcome for patient under TTM. Secondly, we evaluate nutritional tolerance in hypothermia.

**Patients and methods** We retrospectively included patients under TTM after cardiac arrest in a single mixed intensive care unit from January 2008 to December 2014. Patients fed within 48 h after admission (EN; enteral or parenteral) were compared to those fed after 48 h or not fed [delayed nutrition (DN); after rewarming] concerning neurological and infectious outcomes. Enteral nutrition was initiated at maximal caloric objective define at 20 kCal/kg/day until day 7, with isocaloric product. Incidence of vomiting and use of prokinetic drugs were recorded for enteral nutrition tolerance comparison between <36 and ≥36 °C feeding.

**Results** Among 203 patients under TTM at 33 °C after cardiac arrest, 142 were early fed. EN was associated with better neurological outcome assessed by Cerebral Performance Category (CPC) at 3 month (42.3 vs 18%; p = 0.001). After propensity adjustment, EN was still protective for good neurological outcome at 3 month (OR 3.1 [1.36–7.05]; p = 0.01). Comparison between EN and DN showed no difference for early-onset pneumonia (p = 0.4); ventilator-associated pneumonia (VAP) (p = 0.07), nosocomial urinary tract infection (p = 0.35), and nosocomial bacteraemia (p = 0.3). Considering death as competing-risk, VAP were not more frequent with EN (HR 1.07 [0.68–1.69], p = 0.76, Fig. 2). Prokinetic use and vomiting were not increased when nutrition was instituted at temperature <36 °C as compared as ≥36 °C (respectively, 27.2% at 36 °C vs 27.3%, p = 0.99; 34 vs 33%, p = 0.87).

**Fig. 2:**
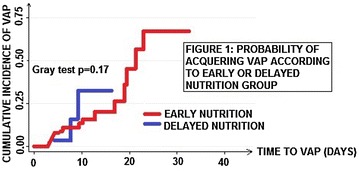
Probability of acquering VAP according to early or delayed nutrition group

**Conclusion** EN is associated with better neurological outcome during targeted temperature management. EN is not associated with more adverse event (infectious and poor nutrition tolerance) when instituted at temperature less than 36 °C.

**Competing interests** None.

**References**

1. Nolan JP, et al. European Resuscitation Council and European Society of Intensive Care medicine guidelines for post-resuscitation care 2015. Resuscitation. 2015;95:202–22.
2. Taylor BE et al. Guidelines for the Provision and Assessment of Nutrition Support Therapy in the Adult Critically Ill Patient: Society of Critical Care Medicine (SCCM) and American Society for Parenteral and Enteral Nutrition (A.S.P.E.N.). Crit Care Med. 2016;44(2):390–438.

#### O5 Description of the epidemiological characteristics and outcome of cardiac arrest in children: a French study

##### Elodie Privat^1^, Joséphine Escutnaire^2^, Cyrielle Dumont^3^, Valentine Baert^2^, Christian Vilhelm^4^, Stéphane Leteurtre^5^, Hervé Hubert^4^

###### ^1^Réanimation pédiatrique, C.H. Régional Universitaire de Lille (CHRU de Lille), Lille, France; ^2^French National Out-of-Hospital Cardiac Arrest Registry (réac), French National Out-of-Hospital Cardiac Arrest Registry (réac), Lille, France; ^3^Public Health Department, Université Lille 2 - Faculté de Médecine Henri Warembourg, Loos, France; ^4^Public Health Department, Université Lille 2 - Faculté de Médecine Henri Warembourg, Lille, France; ^5^Réanimation pédiatrique, Centre Hospitalier Régional Universitaire de Lille, Lille, France

####### **Correspondence**: Elodie Privat - elodie.privat@hotmail.fr

*Annals of Intensive Care* 2017, **7(Suppl 1)**:O5

**Introduction** Cardiac arrest in children has a very poor prognosis. Knowledge of its epidemiological characteristics is necessary to improve the patient care and survival. The French National Cardiac Arrest registry (RéAC), created in 2009, combines all epidemiological data on out-of-hospital cardiac arrests (OHCA). The objective of this study was to describe the epidemiological characteristics and outcomes of OHCA in children under 18 years old.

**Materials and methods** All patients under 18 years old victims of out-of-hospital cardiac arrest and registered in the French National Cardiac Arrest registry between July 2011 and September 2015 were included. Patients were divided into four groups: infants (under 1 year old), toddlers (1–4 years old), children (5–12 years old) and adolescents (13–17 years old). Variables related to anamnesis, basic cardiopulmonary resuscitation, specialized CPR and outcome of patients were collected.

**Results** Out of 42,960 registered cardiac arrest, 900 (2%) involved children under 18 years of age. Out of the 900 patients enrolled, 393 (44%) were less than 1 year old. The OHCA occurred mainly at home (67%). The percentage of traumatic cardiac arrest increased with age, reaching up to 49% in adolescents. Respiratory failure was the leading cause of cardiac arrest in toddlers and children (respectively 40 and 31%). Adolescents were more likely to have an initial shockable rhythm (8%) than other groups (p < 10^−3^). The intraosseous access was used in 33% of the children. Overall survival at 1 month was 8.3% (75/900) and 66.7% (50/75) of these patients had a favorable neurological prognosis. Outcomes description per age is described in Table 1.

**Table 1:** Outcome description of patients

Variables^a^	<1 year (n = 393)	1–4 years (n = 146)	5–12 years (n = 142)	13–17 years (n = 219)	p
ROSC	71 (18)	43 (30)	41 (29)	81 (37)	<10^−3^
Survival at hospital admission	68 (17)	52 (36)	43 (30)	85 (39)	<10^−3^
Survival at day 30	13 (3)	20 (14)	13 (9)	29 (13)	<10^−3^
CPC 1 or 2 at day 30	12 (3)	13 (9)	3 (2)	22 (10)	<10^−3^

*ROSC* return of spontaneous circulation, *CPC* cerebral performance category

**Conclusion** There were significant differences between the patient’s groups regarding the location, type of cardiac arrest, initial rate, and survival. An age group approach could be considered to improve care strategy and survival of cardiac arrest victims.

**Competing interests** None.

#### O6 Contrast-associated acute kidney injury (AKI) in the intensive care unit (ICU): systematic review and meta-analysis

##### Vincent Robert-Edan^1^, Karim Lakhal^1^, Andrew Quartin^2^, Brian Hobbs^3^, Cynthia Cely^2^, Cynthia Bell^4^, Tai Pham^5^, Roland Schein^2^, Yimin Geng^6^, Chaan Ng^7^, Stephan Ehrmann^8^

###### ^1^Service de réanimation chirurgicale, Hôpital Guillaume et René Laënnec, CHU de Nantes, Nantes, France; ^2^Division of Pulmonary, Critical Care, Allergy and Sleep Medicine, Department of Medicine, University of Miami School of Medicine, Miami, United States of America; ^3^Department of biostatistics, University of Texas M.D. Anderson Cancer Center, Houston, TX, United States of America; ^4^Division of Pediatric Nephrology and Hypertension, University of Texas Health Science Center-Houston, Houston, TX, United States of America; ^5^Réanimation médico-chirurgicale, Hôpital Tenon, Paris, France; ^6^Research Medical Library, University of Texas M.D. Anderson Cancer Center, Houston, TX, United States of America; ^7^Department of Radiology, University of Texas M.D. Anderson Cancer Center, Houston, TX, United States of America; ^8^Réanimation polyvalente, CHRU Hôpitaux de Tours, Tours, France

####### **Correspondence:** Vincent Robert-Edan - vincent.re@gmail.com

*Annals of Intensive Care* 2017, **7(Suppl 1)**:O6

**Introduction** Avoiding the use of iodinated contrast media (CM) is frequent, fearing it may contribute to AKI. The aim of this systematic review and meta-analysis was to quantify the risk of AKI attributable to CM in ICU patients.

**Materials and methods** A systematic review until December 31st 2015, through 5 databases, searched for studies evaluating intravascular administration of iodinated CM. Only controlled studies evaluating AKI following CM exposure in ICU patients matched to unexposed patients were included in the meta-analysis. Meta-analysis was performed on patient-level data using a hierarchical Bayesian nested mixed effects multiple logistic regression model. Bayesian methodology allows evaluating how evidence-based physicians would assess the AKI risk attributable to CM according to both their a priori belief and the presentation of the controlled studies identified in the systematic review. Two meta-analyses were performed with different a priori hypotheses. An objective meta-analysis modeled a neutral state of a priori belief (Odds Ratio [OR] of 1 with impartial distribution) yielding a posteriori OR distribution representative of data collected in controlled studies. A subjective meta-analysis modeled the common belief that CM increases the AKI risk, using an a priori OR of 1.37 based on uncontrolled studies holding clinical community consensus. We determined the minimum a priori relative effective sample size (RESS, representing the a priori strength of belief) needed to observe a significant a posteriori OR distribution: how much physicians have to be convinced a priori that CM increases AKI risk to maintain this belief after being confronted with the studies data.

**Results** Among 5696 references, 10 compared ICU patients receiving CM with an unexposed group and 4 performed risk adjustments for baseline AKI risk. Three studies used patient matching: overall, 280 CM patients were matched with 280 control patients. The resulting a posteriori OR did not reach statistical significance: with no prior assumption, there is no evidence that CM increases the risk of AKI in the ICU.

Using an a priori OR of 1.37 (subjective meta-analysis), the a posteriori distribution of the OR did not reach statistical significance except when modelling a very high a priori belief that CM causes AKI (minimum a priori RESS 4.8-folds higher than the RESS of the objective meta-analysis and 70-folds higher than a neutral objective a priori hypothesis).

**Conclusion** This systematic review and meta-analysis did not enlighten a risk of AKI attributable to iodinated CM in ICU patients.

**Competing interests** None.

#### O7 Iohexol clearance for exploring the link between glomerular filtration rate and acute kidney injury in patients with acute circulatory failure

##### Charlotte Salmon Gandonnière^1^, Julie Boisramé-Helms^2^, Olivier Le Tilly^3^, Isabelle Benz De Bretagne^3^, Emmanuelle Mercier^1^, Julie Mankikian^1^, Anne Bretagnol^4^, Ferhat Meziani^2^, Jean Michel Halimi^5^, Chantal Barin Le Guellec^3^, Stephan Ehrmann^1^, Clinical Research in Intensive Care and Sepsis (CRICS network)

###### ^1^Réanimation polyvalente, CHRU Hôpitaux de Tours, Tours, France; ^2^Réanimation médicale, Nouvel Hôpital Civil, CHU Strasbourg, Strasbourg, France; ^3^Biochimie et biologie moléculaire, CHRU Hôpitaux de Tours, Tours, France; ^4^Réanimation médicale polyvalente, Hôpital de La Source, CHR Orléans, Orléans, France; ^5^Néphrologie, transplantation rénale et hémodialyse, CHRU Hôpitaux de Tours, Tours, France

####### **Correspondence:** Charlotte Salmon Gandonnière - charlotte.salmon.gandonniere@gmail.com

*Annals of Intensive Care* 2017, **7(Suppl 1)**:O7

**Introduction** Acute kidney injury (AKI) is associated with significant morbidity and mortality, particularly in intensive care unit (ICU) patients. In stable patients, glomerular filtration rate (GFR), the best overall index of kidney function, can be estimated measuring the plasma clearance of an exogenous tracer such as iohexol [1]. There is no reliable method to assess GFR in unstable patients, as classical methods such as MDRD (modifications of diet in renal disease) and urinary clearance calculations have shown their limits [2]. The aim of this study was to determine GFR using iohexol plasma clearance (Cliox) at the initial phase of acute circulatory failure and to evaluate its association with subsequent development of AKI.

**Patients and methods** Multicentric study in 3 French ICUs. Patients suffering from acute circulatory failure were included within 12 h of ICU admission and administered intravenously a non-toxic dose of iohexol (5 mL; 300 mg/mL) followed by collection of 9 blood samples for iohexol plasma concentration determination (5 and 30 min, 1, 3, 6, 9, 12, 18 and 24 h). Iohexol concentrations were determined using high performance liquid phase chromatography and a three-compartment population pharmacokinetic model was implemented to calculate individual iohexol clearances.

**Results** 100 patients were included. Median age was 65 years (Q1: 55; Q3: 77), baseline MDRD 93 mL/min (73; 116) and SAPS II 59 (45; 75). Most patients were admitted for septic shock. We could calculate Cliox in 85 patients (85%). Failures to calculate Cliox included iodinate contrast media injection outside of the study and early renal replacement therapy. Median Cliox was 38 mL/min (19–58).

76 patients (76%) developed AKI according to the kidney disease, improving global outcome classification (KDIGO): 15 KDIGO 1, 30 KDIGO 2, 31 KDIGO 3. In 59 patients out of 92 for whom enough serum creatinine dosages were available (64%), serum creatinine decreased in the first 24 h of ICU stay, including 44 patients among the the 76 developing AKI (58%). Cliox was inversely related to the severity of AKI: median Cliox for KDIGO 0 patients was 68 mL/min (44; 77), 40 mL/min (30; 58) for KDIGO 1 patients, 36 mL/min (24; 52) for KDIGO 2 patients and 16 mL/min (9; 22) for KDIGO 3 patients. In 40 out of 82 patients (49%) the difference between MDRD (calculated from serum creatinine at the time of inclusion) and Cliox exceeded 20 mL/min, and in most cases (90%) MDRD overestimated estimated GFR (eGFR). For AKI patients, eGFR according to MDRD was >60 mL/min in 18 patients. According to MDRD, 8 patients had glomerular hyperfiltration defined by an eGFR > 130 mL/min. Only 4 patients had hyperfiltration according to Cliox. For the 2 patients with eGFR > 130 mL/min with both methods, one suffered denutrition; his Cliox was 138 mL/min, while MDRD was 281 mL/min. For the 6 patients with hyperfiltration according to MDRD and not Cliox, 4 had denutrition, 1 had a very low baseline serum creatinine (18 μmol/L), and one suffered morbid obesity. Four patients with eGFR > 130 mL/min according to MDRD had a maximum KDIGO score of 2.

**Discussion** Our study confirms that variations of serum creatinine are not a good marker of GFR. We hypothesize this to be related to the large amount of fluid infusion at the acute phase following ICU admission and the influence of nutritional factors. The MDRD formula tended to overestimate eGFR. Cliox may enable to overcome the limits of the MDRD formula at the acute phase of critical illness, as it seems not to be influenced by nutritional factors or fluid infusion, unlike creatinine variations.

**Conclusion** The close link between Cliox and AKI is very encouraging for the development of this method of eGFR assessment in critically ill patients. Cliox could be used for early determination of AKI risk and for drug dosage adaptation, as it is a better reflection of instantaneous GFR than MDRD.

**Competing interests** None.

**References**

1. Macedo E, Bouchard J, Soroko SH, Chertow GM, Himmelfarb J, Ikizler TA, et al. Fluid accumulation, recognition and staging of acute kidney injury in critically-ill patients. Crit Care Lond Engl. 2010;14(3):R82.
2. Bröchner-Mortensen J. A simple method for the determination of glomerular filtration rate. Scand J Clin Lab Invest. 1972;30(3):271–4.

#### O8 Effect of renal replacement therapy strategies in septic-shock patients with severe acute kidney injury: a post hoc analysis of a randomized controlled trial

##### Stéphane Gaudry^1^, David Hajage^2^, Frédérique Schortgen^3^, Laurent Martin-Lefèvre^4^, Florence Tubach^5^, Bertrand Pons^6^, Eric Boulet^7^, Alexandre Boyer^8^, Guillaume Chevrel^9^, Nicolas Lerolle^10^, Dorothée Carpentier^11^, Nicolas de Prost^3^, Alexandre Lautrette^12^, Anne Bretagnol^13^, Julien Mayaux^14^, Saad Nseir^15^, Jean-Damien Ricard^16^, Didier Dreyfuss^17^, AKIKI Study group

###### ^1^Service de réanimation médico-chirurgicale, CHU Louis Mourier, Colombes, Colombes, France; ^2^Département d’épidémiologie et de recherche clinique, Hôpital Louis-Mourier - APHP, Colombes, France; ^3^Réanimation médicale, Hôpital Henri Mondor, Créteil, France; ^4^Réanimation polyvalente, Centre Hospitalier Départemental - site de La Roche-sur-Yon, La Roche-sur-Yon, France; ^5^Département d’epidémiologie et recherche clinique, Unité de Recherche Clinique Paris Nord, Paris, France; ^6^Réanimation - Grands Brulés, CHU Pointe à Pitre - Abymes, POINTE A PITRE, France; ^7^Val d’oise, Hôpital René Dubos, Pontoise, France; ^8^Réanimation médicale, Centre Hospitalier Universitaire de Bordeaux, Bordeaux, France; ^9^Essonnes, C.H. Sud Francilien, Corbeil-Essonnes, France; ^10^Réanimation médicale, Centre Hospitalier Universitaire d’Angers, Angers, France; ^11^Réanimation médicale, Centre Hospitalier Universitaire Rouen, Rouen, France; ^12^Réanimation médicale, CHU Gabriel-Montpied, Clermont-Ferrand, France; ^13^Réanimation médicale polyvalente, Hôpital de La Source, CHR Orléans, Orléans, France; ^14^Réanimation médicale, Hôpital Pitié-Salpêtrière, Paris, France; ^15^Centre de Réanimation, Centre Hospitalier Régional Universitaire de Lille, Lille, France; ^16^Service de Réanimation Médico-Chirurgicale, CHU Louis Mourier, Colombes, France; ^17^Inserm, iame, umr 1137, Université Paris Diderot, Sorbonne Paris Cité, Paris, France

####### **Correspondence:** Stéphane Gaudry - stephanegaudry@gmail.com

*Annals of Intensive Care* 2017, **7(Suppl 1)**:O8

**Introduction** Acute kidney injury is particularly common in septic-shock patients and is associated with high mortality. The putative effect of renal replacement therapy (RRT) on the prognosis of such patients is highly debated; some advocating that outcome might improve owing to modulation of inflammation. We aimed to compare outcomes of septic-shock patients with severe acute kidney injury (stage 3 of KDIGO classification) treated with an early RRT strategy (all patients immediately received RRT) with those treated with a delayed RRT strategy (patients received late RRT or no RRT at all).

**Patients and methods** We did a post hoc subgroup analysis in a subset of septic-shock patients with severe acute kidney injury (stage 3 of KDIGO classification) from a multicenter randomized controlled trial. In the trial, patients from 31 intensive care were randomly assigned (1:1) to either an early or a delayed RRT initiation strategy. With the early strategy, RRT was initiated within 6 h after inclusion criteria were met. With the delayed strategy, RRT was started if at least one of the following criteria was met: severe hyperkalemia, metabolic acidosis, pulmonary edema, serum urea concentration greater than 40 mmol/l, or oliguria for more than 72 h after randomization. The primary outcome was overall survival at day 60.

**Results** Of the 413 septic-shock patients (on a total of 620 patients), 209 were managed with early strategy and 204 with delayed strategy. The Kaplan–Meier estimates of mortality at day 60 did not differ significantly between the early and delayed strategies; 101 deaths occurred among 209 patients in the early-strategy group (48.5%; 95% confidence interval [CI] 41.3–54.9), and 99 deaths occurred among 204 patients in the delayed-strategy group (48.5%, 95% CI 41.2–55.0; P = 0.97) (Fig. 3). A total of 97 patients (47.5%) in the delayed-strategy group did not receive renal-replacement therapy. The number of days RRT-free days was significantly higher in the delayed strategy group (21 [5–29] vs 17 [2–25], p < 0.001). Median length of stay in hospital did not differ significantly between groups (20 [8–39] vs 19 [7–40] days, p = 0.9).

**Fig. 3:**
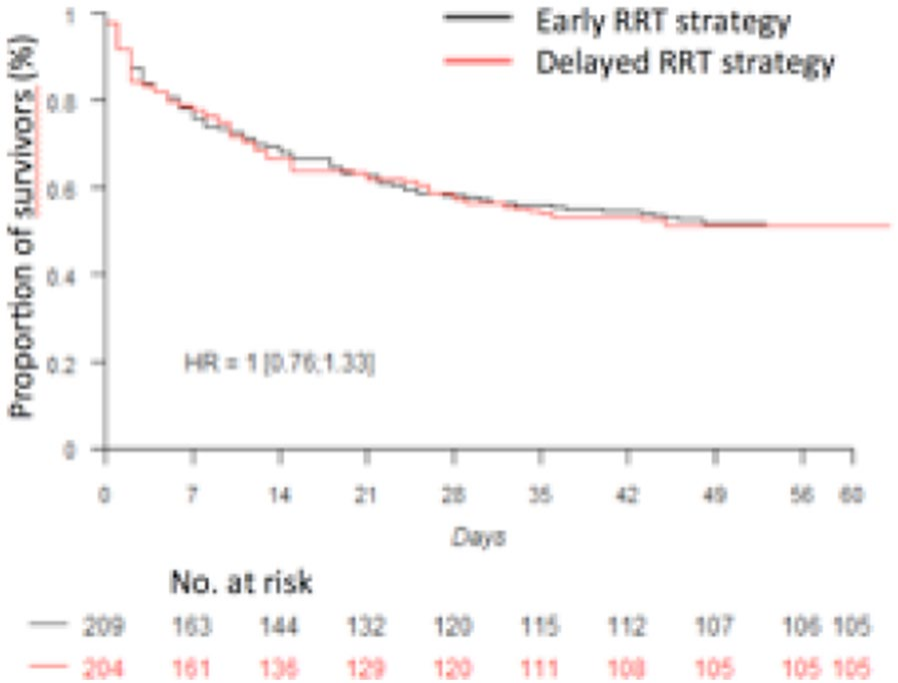
Probability of survival and timing of RRT initiation

**Conclusion** The timing of RRT in septic-shock patients with severe acute kidney injury did not significantly influence mortality. However, a conservative strategy avoided many unnecessary RRT sessions.

**Competing interests** None.

#### O9 Dose response multicentre investigation on fluid assessment (DOREMIFA) in critically ill patients: the French cohort

##### René Robert^1^, Franscesco Garzotto^2^, Eric Kipnis^3^, Ciro Tetta^4^, Claudio Ronco^2^, DO-RE-MI-FA Group

###### ^1^Service de Réanimation médicale, CHU de Poitiers, Poitiers, France; ^2^International Renal Research Institute of Vicenza, San Bortolo Hospital, Vicenza, Italy; ^3^Réanimation chirurgicale, Centre Hospitalier Régional Universitaire de Lille, Lille, France; ^4^Research extracorporeal therapies, Fresenius Medical Care, Bad Homburg vor der Höhe, Germany

####### **Correspondence:** René Robert - rene.robert@chu-poitiers.fr

*Annals of Intensive Care* 2017, **7(Suppl 1)**:O9

**Introduction** There is growing evidence that fluid accumulation beyond the correction of hypovolaemia is associated with increased morbidity, mortality and a longer hospital stay. We recently published a prospective cohort observational study “The Dose Response Multicentre Investigation on Fluid Assessment (DoReMIFA) in critically ill patients” aimed to investigate the impact of fluid balance and fluid accumulation on mortality for both AKI and (Non) N-AKI patiens as for those who receive renal replacement therapy (AKI-RRT).

Aim of the present is to assess the fluid administration of the French subgroup of patients.

**Patients and methods** We analysed 209 (12.05%) of the 1734 enrolled patients, from the 2 French ICUs. Fluid overload (FO) was defined as the ratio between cumulative fluid balance and the initial body weight, in percentage. Maximum fluid overload (MFO) referred to the peak value of FO during the entire ICU stay. TMFO represented the number of days between. ICU admission and day of MFO. Velocity of fluid accumulation was defined by Fluid overload slope (FOSL) as the MFO/TMFO ratio. A boxplots for the three groups (N-AKI, AKI and AKI-RRT) illustrated the MFO for both survivors and non-survivors during the ICU stay. A Kaplan–Meier analysis was performed to evaluate the time to death for the three groups (N-AKI, AKI and AKI-RRT). The time to death was evaluated by a Cox proportional hazard regression analysis.

**Results** 53% of patients had AKI (38% stage 1, 20% Stage 2, 42% Stage3). The Kaplan–Meier analysis including the first 28 days of ICU, highlighted a significant survival benefit for patients without AKI, in particular for longer ICU stay. The AKI and AKI-RRT group had, conversely form the entire study population, similar survival rates. In all cohorts as in N-AKI, AKI and AKI-RRT non-survivors had a higher MFO than survivors. Again, the AKI-RRT and AKI groups had similar levels of MFO, with a lower over-hydration for the RRT group. Cox regression analysis of the velocity of fluid accumulation showed that for every increase of one unit of the FOSL, the hazard of death increased significantly by a factor of 1.44. The hazard ratio decreased to 1.41 when adjusting for SAPS II score.

**Discussion** The fluid assessment in critically ill patients enrolled on the French ICUs confirm the findings of the Doremifa study. Fluid overload is strongly correlated with mortality at any degree. A lower degree of fluid overload for the AKI-RRT group, when compared with the AKI population, and a similar survival curve between the 2 groups, may suggest that CRRT has a protective effect. More analysis are needed to confirm this hypothesis, an early initiation of the treatment have to be also investigated.

**Conclusion** The velocity of fluid accumulation, as in the findings of the main study, contribute to worse patients outcome.

**Competing interests** Fresenius medical care: fees for travelling and hotel.

#### O10 Doppler-based renal resistive index in assessing renal dysfunction reversibility in ICU patients: results of a multicenter cohort study

##### David Schnell^1^, Bourmaud Aurelie^2^, Marie Reynaud^3^, Christophe Clec’h^4^, Julie Boisramé-Helms^5^, Mourad Benyamina^6^, François Vincent^7^, Alexandre Lautrette^8^, Christophe Mariat^9^, Caroline Bornstain^7^, Stephane Rouleau^10^, Christophe Leroy^8^, Yves Cohen^11^, Jerome Morel^3^, Matthieu Legrand^6^, Jeremy Terreaux^12^, Michaël Darmon^12^

###### ^1^Service de reanimation, Service de Réanimation polyvalente, Angoulême, France; ^2^Département de santé publique, Institut de Cancérologie de la Loire Lucien Neuwirth, Saint-Priest-en-Jarez, France; ^3^Réanimation polyvalente b, Hospital Center University De Saint-Étienne, Saint-Priest-en-Jarez, France; ^4^Réanimation medico-chirurgicale, Hopital Avicenne, Bobigny, France; ^5^Réanimation, CHU de Strasbourg, Strasbourg, France; ^6^Anesthésie réanimation et traitement chirurgical des grands brûlés, APHP - Hopital Saint-Louis, Paris, France; ^7^Réanimation polyvalente, Groupe Hospitalier Intercommunal Le Raincy-Montfermeil, Montfermeil, France; ^8^Réanimation médicale, CHU Gabriel-Montpied, Clermont-Ferrand, France; ^9^Néphrologie, Centre Hospitalier Universitaire de Saint-Étienne, Saint-Étienne, France; ^10^Service de réanimation, Hospital Center D’angoulême, Angoulême, France; ^11^Réanimation médico-chirurgicale, Hôpital Avicenne, Bobigny, France; ^12^Réanimation médicale, CHU Saint-Etienne - Hôpital Nord, Saint-Étienne, France

####### **Correspondence:** Michaël Darmon - michael.darmon@chu-st-etienne.fr

*Annals of Intensive Care* 2017, **7(Suppl 1)**:O10

**Introduction** Doppler-based renal resistive index (RI) measurement may hold promise in differentiating transient from persistent AKI in selected critically ill patients. Although several studies have suggested adequate performance in predicting short-term reversibility of AKI, most of these studies were performed in limited patient samples [1]. Additionally, a recent study has identified discrepant results regarding its diagnostic performance [2] suggesting confirmatory studies to be required.

The main objective of this study was to assess diagnostic performance of RI in predicting persistent AKI in critically ill patients. Secondary objectives were to assess diagnostic performance of semi-quantitative assessment of renal perfusion (SQP) using color-Doppler in predicting persistent AKI and performance of both tests in predicting needs for renal replacement therapy (RRT).

**Patients and methods** Prospective multicenter study performed in eight ICUs from December 2013 to April 2016. This study was declared to Clinicaltrial.gov.

Adult patients requiring mechanical ventilation were included in this study. Patients with mild to severe chronic kidney diseases, arrhythmia, or obstructive renal dysfunction were excluded from this study. Patients with hospital stay shorter than 72 h and in whom renal reversibility could not be assessed were secondarily excluded.

Acute kidney injury (AKI) was defined according to the KDIGO definition.

Transient AKI was defined as AKI with recovery within the first 3 days following inclusion.

Intra-renal RI was calculated as (peak systolic velocity − end-diastolic velocity)/peak systolic velocity.

SQP was assessed using a scale ranging from 0 (absence of renal perfusion) to 3 (renal vessels identifiable until the arcuate arteries in the entire field of view).

Results are reported as number (%), median (IQR) and area under curve (95% CI).

**Results** Overall, 371 patients were included. Median age was 76 years (66–89) and 236 patients were of male gender (63.6%). Most of the patients were admitted with medical conditions (n = 253; 68.2%) and 162 patients (43.7%) had sepsis at ICU admission. Median LOD score was of 8 (5–11) at study inclusion and 198 patients required vasopressors (53.4%).

Of the included patients, 253 (68.2%) had an AKI at study inclusion, including 158 patients (42.6%), 35 (9.4%) and 60 (16.2%) with AKI stage 1, 2 and 3 respectively. Doppler-based RI was obtained in 365 patients (98.4%), semi-quantitative assessment of renal perfusion in 367 (98.9%). Patients with AKI had a higher RI at ICU admission [0.70 (0.62–0.77) vs. 0.65 (0.59–0.70); P = 0.0001] and a lower SQP [2(1–3) vs. 2 (2–3); P = 0.0003].

Twenty patients were discharged before day 3 leaving 351 patients in the final analysis, including 118 (33.6%), 97 (27.6%) and 136 patients (38.7%) no AKI, transient AKI or persistent AKI respectively.

Resistive index at inclusion was of respectively 0.65 (0.59–0.70), 0.69 (0.62–0.77) and 0.71 (0.62–0.77) in patients without AKI, with transient AKI and with persistent AKI (P = 0.0005). Resistive index failed to demonstrate any interest in predicting persistent AKI (Area under ROC curve: 0.58; 95% CI 0.52–0.64).

Semi-quantitative assessment of renal perfusion was of respectively 2 (2–3), 2 (2–3), and 2 (1–3) in patients without AKI, with transient AKI and with persistent AKI (P = 0.002)]. Semi-quantitative assessment of renal perfusion failed to demonstrate any interest in predicting persistent AKI (Area under ROC curve: 0.59; 95% CI 0.52–0.65).

Overall, 46 patients (12.4%) required RRT during ICU stay. In these patients, RI and SQP were of respectively 0.75 (0.65–0.77) [vs. 0.67 (0.60–0.74) in non-RRT patients; P = 0.0003] and 2 (1–2) [vs. 2 (2–3) in non-RRT patients; P = 0.0003]. Both test displayed a poor performance in predicting subsequent renal replacement therapy at study inclusion [AUC ROC curve of respectively 0.67 (0.59–0.74) and 0.65 (0.57–0.73) for RI and semi-quantitative perfusion].

**Conclusion** Our results suggest that renal perfusion monitoring using Doppler-based resistive index or color-Doppler, although feasible in ICU setting, failed to predict short term AKI reversibility and displayed a poor performance in predicting needs for renal replacement therapy.

**Competing interests** None.

**References**

1. Ninet et al. J Crit Care. 2015.
2. Dewitte et al. Crit Care. 2012.

#### O11 Neurologic outcomes and adjunctive steroids in adults with severe tuberculous meningitis: the tuberculous meningitis in ICU (TBM in ICU) multicenter study

##### Marie Cantier^1^, Adeline Morisot^2^, Emmanuel Guérot^3^, Bruno Megarbane^4^, Keyvan Razazi^5^, Damien Contou^5^, Eric Mariotte^6^, Emmanuel Canet^6^, Etienne De Montmollin^7^, Vincent Dubée^8^, Eric Boulet^9^, Stéphane Gaudry^10^, Guillaume Voiriot^11^, Julien Mayaux^12^, Frédéric Pène^13^, Mathilde Neuville^1^, Jean-François Timsit^1^, Romain Sonneville^1^, ENCEPHALITICA Study Group

###### ^1^Service de réanimation médicale et infectieuse, Hôpital Bichat-Claude Bernard-APHP, Paris, France; ^2^Centre mémoire, clinique rainier iii, Hospital Center Princesse Grace, Monaco, Monaco; ^3^Réanimation médicale, Hopital Europeen Georges-Pompidou, Paris, France; ^4^Service de Réanimation Médicale et Toxicologique, CHU Lariboisière, Paris, France; ^5^Réanimation Médicale, Hôpital Henri Mondor, Créteil, France; ^6^Service de réanimation médicale, Hôpital Saint-Louis (AP-HP), Paris, France; ^7^Réanimation, C.H. Général Saint Denis hôpital Delafontaine, Saint-Denis, France; ^8^Réanimation médicale, Hôpital Saint-Antoine, Paris, France; ^9^Val d’oise, Hôpital René Dubos, Pontoise, France; ^10^Service de réanimation médico-chirurgicale, CHU Louis Mourier, Colombes, France; ^11^Service de réanimation médicale, Hôpital Tenon (AP-HP), Paris, France; ^12^Réanimation médicale, Hôpital Pitié-Salpêtrière, Paris, France; ^13^Réanimation Médicale, Hôpital Cochin, Paris, France

####### **Correspondence:** Marie Cantier - mariecantier@gmail.com

*Annals of Intensive Care* 2017, **7(Suppl 1)**:O11

**Introduction** Tuberculous meningitis (TBM) is frequently associated with neurological complications requiring admission in intensive care unit (ICU). Adjunctive steroids reduce mortality, but may have no effect on disabling neurologic deficits in survivors. Moreover, the benefit of steroids is controversial in immunocompromised patients and in patients with severely altered mental status at hospital presentation. We aimed to identify indicators of poor functional outcome in adult patients with severe forms of TBM requiring ICU admission. In particular, we investigated the effect of adjunctive steroids on functional outcomes and 1-year mortality.

**Patients and methods** We conducted a retrospective cohort study (January 1st 2004 to June 15th 2016) in consecutive TBM cases admitted to the medical ICUs of 12 university-affiliated hospitals, located in the Paris area, France. We used multivariate logistic regression analysis to identify factors associated with a poor functional outcome. A poor functional outcome was defined as a score of 3–6 on the modified Rankin scale (mRS) 90 days after ICU admission. In a secondary analysis, we used Cox proportional hazards modeling to evaluate the risks of death at 1 year. Data are presented in median (interquartile range) or numbers (percentage).

**Results** A total of 95 patients were studied, including 44 (46%) immunocompromised patients. At ICU admission, the Glasgow Coma Scale score was 11 (8–14), 64 (67%) patients had Medical Research Council (MRC) grade 3 illness, and 66 (69%) required invasive mechanical ventilation. Brain MRI was performed in 79/95 (83%) cases. Antituberculous therapy was administered to 90/95 (95%) patients (5 died before treatment could be started), and 76/90 (84%) patients received adjunctive steroids. The duration of ICU stay was 10 (4–24) days.

A poor functional outcome was reported for 55/90 (61%) patients at 90 days (5 patients were lost to follow-up). The following factors were independently associated with a poor outcome at 90 days: older age (per 1-year increment, adjusted odds ratio (aOR): 1.04, 95% CI 1.0–1.08, p = 0.035), cerebrospinal fluid (CSF) protein level >1.9 g/L (aOR: 8.85, 95% CI 2.49–39.64, p = 0.002), hydrocephalus on MRI (aOR: 8.6, 95% CI 2.05–46.5, p = 0.006). By contrast, the use of adjunctive steroids had a protective effect (aOR: 0.15, 95% CI 0.02–0.87, p = 0.045).

The Kaplan–Meier estimated 1-year mortality was 51% (0.39–0.61). The following factors were independently associated with mortality: CSF protein levels >1.9 g/L [adjusted relative risk (aRR): 2.47, 95% CI 1.17–5.25, p = 0.018], hydrocephalus on MRI (aRR: 3.45, 95% CI 1.50–7.91, p = 0.003), brain infarction on MRI (aRR: 2.40, 95% CI 0.99–5.81, p = 0.051). The use of adjunctive steroids had a protective effect on 1-year mortality (aRR = 0.16, 95% CI 0.05–0.45, p = 0.0006).

**Conclusion** Despite antituberculous therapy and supportive care, severe forms of TBM are characterized by a poor outcome in more than 50% of cases. Elevated CSF protein levels, hydrocephalus and brain infarction on MRI at ICU admission represent major indicators of poor outcome. Our data suggest that use of adjunctive steroids is associated with reduced disability and mortality, irrespective of immune status and severity of illness. We conclude that adjunctive steroids may benefit to all patients with a suspicion of TBM admitted to the ICU.

**Competing interests** None.

#### O12 Value of diaphragmatic echography for predicting respiratory insufficiency in 123 patients with neuromuscular disorders

##### Abdallah Fayssoil^1^, Tania Stojkovic^2^, Anthony Behin^2^, Adam Ogna^3^, Frédéric Lofaso^4^, Pascal Laforet^2^, Karim Wahbi^2^, Helene Prigent^4^, Denis Duboc^5^, David Orlikowski^3^, Bruno Eymard^2^, Djillali Annane^6^

###### ^1^Neurologie et reanimation polyvalente, Institute De Myologie et CHU Raymond Poincaré, Garches, France; ^2^Neurologie, Institute De Myologie, Paris, France; ^3^Réanimation polyvalente et pole ventilation à domicile, CHU Raymond Poincare, Garches, France; ^4^Service d’explorations fonctionnelles respiratoires, Hôpital Raymond-Poincaré (AP-HP), Garches, France; ^5^Cardiologie, Hôpital Cochin, Paris, France; ^6^Réanimation médico-chirurgicale, Hôpital Raymond-Poincaré, Garches, France

####### **Correspondence:** Abdallah Fayssoil - fayssoil2000@yahoo.fr

*Annals of Intensive Care* 2017, **7(Suppl 1)**:O12

**Introduction** Diaphragm is the main muscle of ventilation. Diaphragmatic echography is a non invasive tool used in Intensive Care Unit. We sought to compare diaphramatic echography and spirometric function in neuromuscular disorders.

**Patients and methods** We included retrospectively patients with neuromuscular disorders followed in 2 neuromuscular centers (Pitié and Garches, France). Ultrasonographic (US) analysis of the right and the left hemidiaphragm, using M-mode, was performed during quiet breathing and deep breathing. For the right hemidiaphragmatic analysis, the transducer was placed on the anterior subcostal region at the right mid-clavicular line. For the left hemidiaphragmatic analysis, the transducer was placed on the anterior subcostal region between the anterior and midaxillary lines.

**Results** 123 patients with muscular dystrophies (DM1, PROMM, DMD, BMD, LGMD, FSH, mitochondriopathies and other myopathies) were included in our study. Mean age was 39 ± 14 years. Mean Walton score was 2.6 ± 2. Mean forced vital capacity (FVC) was 74 ± 28% of predicted value and mean PCO_2_ was 42 ± 6 mmHg. Mean right hemidiaphragmatic excursion (RHDE) during quite breathing was 14.5 ± 6 mm and 44.7 ± 22 mm during deep breathing. Mean left hemidiaphragmatic excursion (LHDE) during quite breathing was 14.3 ± 6 mm and 34 ± 22 mm during deep breathing. FCV was significantly correlated with RHDE during quite breathing (r = 0.55, p < 0.001), with LHDE during quite breathing (r = 0.57, p < 0.001), with RHDE (r = 0.71, p < 0.001) and LHDE (r = 0.78, p < 0.001) during deep breathing. RHDE during deep breathing predicts a FVC < 60% with AUC at 0.86. RHDE during deep exhalation predicts a peak expiratoty flow <270 l/min with AUC at 0.78.

**Conclusion** Diaphragmatic echography may predict respiratory insufficiency in neuromuscular disorders.

**Competing interests** None.

#### O13 Brain injury during veno-arterial extracorporeal membrane oxygenation

##### Loic Le Guennec^1^, Clémentine Cholet^1^, Matthieu Schmidt^1^, Nicolas Bréchot^1^, Guillaume Hekimian^1^, Sébastien Besset^1^, Guillaume Lebreton^2^, Ania Nieszkowska^1^, Jean Louis Trouillet^1^, Pascal Leprince^2^, Alain Combes^1^, Charles-Edouard Luyt^1^

###### ^1^Service de réanimation médicale, Groupe Hospitalier Pitié Salpêtrière, Paris, France; ^2^Service de chirurgie cardiaque, Groupe Hospitalier Pitié Salpêtrière, Paris, France

####### **Correspondence:** Loic Le Guennec - loic.leguennec@yahoo.fr

*Annals of Intensive Care* 2017, **7(Suppl 1)**:O13

**Introduction** Veno-arterial extracorporeal membrane oxygenation (VA-ECMO) is used to provide cardiac support in patient suffering refractory cardiogenic shock. Its use is increasing and associated with neurological complications, mainly cerebrovascular. The frequency of those events and their impact on patients are not well described. We therefore study the epidemiology, risk factors and impact of cerebral complications occurring in VA-ECMO patients.

**Patients and methods** Observational study conducted in a tertiary referral center (2006–2014) on patients developing a neurological complication (ischemic stroke or intracranial bleeding) while on VA-ECMO versus those who did not.

**Results** The main clinical characteristics of the patients are reported in Table 2. Among 873 consecutive patients who had received VA-ECMO, 72 developed cerebral complications on ECMO: ischemic stroke in 47 (5%) with and cerebral bleeding in 25 (3%), occurring after a median [IQR) of 11 [7–21.5] and 6 [5–11] days of ECMO support, respectively.

**Table 2:** Admission characteristics, hemostasis disorders during VA-ECMO and patient outcomes according to brain damage or not

Characteristic	No brain damage		Patients with brain damage
	(*n* *=* *800*)	Ischemic stroke (*n* *=* *47*)	Cerebral bleeding (n = 25)
Age (years)	50 ± 15	50 ± 16	48 ± 18
Male sex [n (%)]	568 (71)	32 (68)	12 (48)
SAPS II	72 [54–85]	70 [61–86]	74 [57–90]
SOFA score	5 [3–14]	5 [3–8]	3 [3–7]
Biologic values (J1 ECMO)			
Lactates (mmol/L)	6 [2.7–11.1]	5 [2.9–10.1]	4 [4.0–10.0]
PT (%)	45 [30–62]	54 [34–65.8]	47 [27.5–54]
ACT	1.6 [1.2–2.3]	1.4 [1.1–1.8]	1.8 [1.4–3]
Fibrinogen (g/L)	3.0 [2.0–5.0]	3.0 [2.4–5.0]	4.0 [1.7–4.0]
Platelets (×10^9^/L)	155 [96–215]	163 [100–246]	86 [56.5–164]
Type of ECMO			
Peripheral	698 (87)	35 (74)	20 (80)
Switch to central ECMO	38 (5)	2 (4)	4 (16)
Central	107 (13)	12 (26)	5 (25)
Intra-aortic balloon pump	265 (33)	16 (34)	5 (25)

Results are expressed as mean ± SD, number (%) or median [27th–75th percentile interquartile range]

No specific risk factor of ischemic stroke was found in univariable analysis except body mass index >26 (OR 2.16, 95% CI 1.15–4.05). Hematological failure (defined as platelets <50 000/mL) at ECMO initiation (OR CI 3.64, 95% 1.30–10.21) and platelets <20,000/mL during ICU stay (OR 3.01, 95% CI 1.22–7.40) were significantly associated with cerebral bleeding in univariable analysis. Age, comorbidities, renal replacement therapy, and intra-aortic balloon pump use were not associated with neurological complications. Twenty-three (49%) patients with ischemic stroke and 21 (84%) with intracranial bleeding died versus 385 (48%) of patients without brain injury.

**Conclusion** Neurological events occurred frequently in patients on VA-ECMO. Ischemic stroke is the most frequent, occurs late during ECMO support and does not seem to be associated with higher mortality than patients without brain injury. Cerebral bleeding occurs early and is associated with high mortality rate. Low platelets count at ECMO initiation and during ECMO support are associated with cerebral bleeding.

**Competing interests** None.

#### O14 Does heart rate variability predict clinical outcome of patients with subarachnoid hemorrhage in the neurointensive care unit?

##### Marion Griton^1^, Musa Sesay^1^, Nadia Sibaï De Panthou^1^, Thomas Bienvenu^1^, Matthieu Biais^1^, Karine Nouette-Gaulain^1^

###### ^1^Neuro ICU, CHU - Hôpitaux de Bordeaux, Bordeaux, France

####### **Correspondence:** Marion Griton - marion.griton@chu-bordeaux.fr

*Annals of Intensive Care* 2017, **7(Suppl 1)**:O14

**Introduction** Patients with subarachnoid hemorrhage (SAH), admitted to the neurointensive care unit (NICU) are exposed to complications including rebleeding, vasospasm, hydrocephalus, pain and sepsis. The autonomic nervous system (ANS) is a warning system which can be assessed noninvasively by heart rate variability (HRV). Many reports have shown a relationship between HRV and outcome in myocardial infarction, stroke and renal insufficiency. To our knowledge very few papers have addressed this issue in the NICU. The aim of this study was to check whether HRV could predict outcome in patients with SAH admitted to the NICU.

**Patients and methods** Following Institutional Review Board approval, patients with SAH, admitted to the NICU were included in this prospective monocentric study. Those with persistent arrhythmia, cardiac pacing or younger than 18 years were excluded. All subjects were assessed everyday starting from their arrival at NICU, i.e. day 2 after SAH, to day 7. HRV was measured between 2 and 4 p.m. during 10-min. HRV was achieved by connecting a computer to the electrocardiogram (ECG) monitor. Online power spectrum was calculated from the ECG R–R interval using the maximum entropy method (MemCalc™, Suwa Trust, Japan). Low (LF: 0.04–0.15 Hz) and high frequency (HF: 0.15–0.4 Hz) spectra were associated with sympathetic (Σ) and vagal activities respectively. Entropy and coefficient of variation of RR intervals (CVVR) were also measured.

Concomitantly, we noted demographic, hemodynamic, respiratory and comorbidity data. The severity of SAH was classified using the five points World Federation of Neurological Surgeons (WFNS) score (were 1 = less severe and 5 = very severe) and the four point Fisher scale (where 1 = less severe and 4 = very severe). Outcome at discharge was assessed by the modified Rankin scale, a poor outcome was defined by either death or Rankin score of 4–5 which means severe disability and inability to walk without assistance Outcome was defined as good or poor based on the absence or presence of Rankin score of 4–5 and/death. Univariate and multiple logistic regression models were applied on these data to determine the predictive (s) factor (s) of poor outcome.

**Results** The inclusion criteria were fulfilled by 125 patients, but complete data was only available in 53 of them. Among them, five died before discharge, 10 were discharged with a Rankin score of 4 or 5. The most significant modifications of our study parameters were observed on day 2 after SAH. These observations are summarized in Table 3. Consequently, on day 2 after hemorrhage, LF (p = 0.02), VLF (p = 0.03), entropy (p < 0.001) and CVVR (p < 0.01) were significantly decreased in patients with poor prognosis. This trend was sustained The multivariate logistic regression model revealed an odds ratio [95% confidence interval] for LF = 0.997 [0.995–1.000]; Entropy = 0.919 [0.0.872–0968]; VLF = 0.997 [0.995–1.000] and CVVR 0.586 [0.388–0.885] considered as significant predictors of poor outcome.

**Table 3:** Univariate analysis of data in the study population

	Good outcome	Poor outcome	p value
No of patients (%)	38 (72)	15 (28)	
Mean age (years)	56.2 ± 11.7	59.6 ± 12.1	0.004
Female sex	23 (60)	9 (60)	0.86
WFNS IV–V	10 (26)	7 (47)	<0.001
Fisher grade 4	18 (47)	9 (60)	<0.001
HRV on day 2			
LF (ms)	519 ± 528	171 ± 385	0.02
HF (ms)	513 ± 863	224 ± 505	0.24
Entropy	50.3 ± 12.0	34.3 ± 16.4	<0.001
LF/HF	1.97 ± 1.41	1.87 ± 2.27	0.85
VLF (ms)	1471 ± 2152	184 ± 364	0.02
CVVR (%)	4.18 ± 2.01	2.25 ± 2.31	0.006

**Discussion** These results corroborate previous reports showing that a decrease in HRV, particularly the sympathetic pathway, at admission, is associated with poor outcome (1).The mechanisms are not clearly known. Ischemic or hemorrhagic lesions had been observed in the hypothalamus of patients with poor outcome after SAH, which is the predominant modulator of autonomic system (2).

**Conclusion** This study suggests that early assessment of HRV in the NICU could predict outcome in patients with SAH. A decrease in HRV at admission of patients was significantly associated with poor outcome. Further studies are required to confirm this finding.

**Competing interests** None.

**References**

1. Brain J Neurol. 1963; 86: 301–14.
2. Am J Emerg Med. 2012; 30: 651–56.

#### O15 Electrical muscle stimulation and bicycling combined to early standard rehabilitation versus early standard rehabilitation alone: impact on global muscle strength at ICU discharge—an open-label, single-centre, assessor-blinded randomised trial

##### Guillaume Fossat^1^, Florian Baudin^1^, Cécile Coulanges^2^, Sabrine Bobet^1^, Arnaud Dupont^3^, Léa Courtes^1^, Anne Bretagnol^1^, Dalila Benzekri^1^, Toufik Kamel^1^, Grégoire Muller^1^, Nicolas Bercault^1^, François Barbier^1^, Isabelle Runge^1^, Mai-Anh Nay^1^, Marie Skarzynski^1^, Armelle Mathonnet^1^, Thierry Boulain^1^

###### ^1^Réanimation médicale polyvalente, Hôpital de La Source, CHR Orléans, Orléans, France; ^2^Service de pneumologie, Hôpital de La Source, CHR Orléans, Orléans, France; ^3^Service de réanimation chirurgicale, Hôpital de La Source, CHR Orléans, Orléans, France

####### **Correspondence:** Thierry Boulain - thierry.boulain@chr-orleans.fr

*Annals of Intensive Care* 2017, **7(Suppl 1)**:O15.

**Introduction** Early Standard Rehabilitation (ESR), first passive and then passive/active, is recommended for critically ill patients in whom it reduces the duration of mechanical ventilation (MV), improves functional status, muscle strength and quality of life after hospital discharge. The early addition of leg bicycling on a cyclo-ergometer and of electrical muscle stimulation (EMS) is now part of common practice in the ICU. Whether it can preserve or improve muscle strength and further increase the beneficial effects of ESR is little known.

**Patients and methods** Single-centre, randomised study comparing the effects of the combination of early and daily leg bicycling + EMS of the quadriceps + ESR (intervention group) versus ESR alone (usual care group) on the global muscle strength assessed by the MRC score at ICU discharge by a physiotherapist blinded to the randomization group (NCT02185989). All consecutive patients were potentially eligible if they were deemed to need more than 72 h of care in ICU. Main non-inclusion criteria were resuscitated cardiac arrest, presence of pacemaker or implantable defibrillator, acute cerebral disease requiring deep sedation for at least 72 h, known neuromuscular disease, and amputation of a lower limb. Randomization was stratified by sex, MV or not at study entry, and day of admission (Thursday/Friday vs other days). The interventions were applied right from Day 1 (within 72 h of admission), 5 days/week. Protocoled ESR consisted of daily multistep program (from 10 passive mobilisations of each joint in comatose patients to passive/active muscle work, transfer to chair, standing and walking, depending on patient’s level of wakefulness/cooperation). In the intervention group, 30 min passive/active leg bicycling (even in bed-ridden patients) and 54 min EMS of the quadriceps were performed 5 days/week in addition to ESR, according to pre-established programs.

**Results** From July 2014 to June 2016, 314 patients were included (as planned per protocol) and 313 were analysable (1 consent withdrawal): 155 in usual care group and 158 in intervention group. Among the whole population, ICU mortality was 18%, SAPSII 46 ± 18, admission SOFA 8 (IQR 6; 12), patients treated with MV 85%. Clinical characteristics at study entry were similar between groups. *Primary endpoint*: 124 and 121 patients upon the 131 and 125 ICU survivors in usual care and intervention groups respectively, could be assessed for the primary endpoint. The discharge MRC score was 53 (IQR 44; 60) and 51 (44; 58) in usual care and intervention groups, respectively (P = 0.86), and was also not different between groups in patients under MV at time of study entry: 52 (IQR: 44; 58) (n = 95) and 49 (43; 57) (n = 89) (P = 0.26). *Secondary endpoints*: There was no between-group difference in discharge functional status as assessed by the *ICU mobility scale* in the whole population (P = 0.54) (P = 0.64 in patients under MV at study), or by the change in the Katz index from inclusion to discharge (P = 0.39) (P = 0.47 in patients under MV at study entry), or in the day-28 ventilator-free days [21 days (17; 22) vs 20 days (18; 21); P = 0.34]. The thickness of the *rectus femoris* muscle, assessed by echography at inclusion and discharge in survivors, showed a lower decline in the intervention group: −1.8 mm (−4.4; −0.2) vs −0.7 mm (−1.5; −0.25); P = 0.009. The impact on delirium occurrence in ICU is still under analysis. Data concerning physical and mental status at 6 months are not fully available yet.

*Safety:* We observed no serious adverse event related to the studied interventions.

**Conclusion** Although safe and resulting in lower decline in muscle thickness as observed on echography (not blinded assessment), the addition of daily leg bicycling and EMS to ESR did not result in higher global muscle strength as assessed by the MRC score (blinded assessment) at ICU discharge in a mixed and heterogeneous population of critically ill patients. Exploratory subgroup analyses are underway and perhaps will help to identify subsets of patients in whom the studied intervention might be beneficial and might deserve further investigations.

**Competing interests** None.

#### O16 Post-intensive care syndrome: a population-based observational study of healthcare use

##### Youenn Jouan^1^, Noémie Teixera^2^, Claire Hassen-Khodja^3^, Antoine Guillon^1^, Christophe Gaborit^3^, Leslie Grammatico-Guillon^3^, Stephan Ehrmann^1^

###### ^1^Réanimation polyvalente, CHRU Hôpitaux de Tours, Tours, France; ^2^Service des urgences, CHRU Hôpitaux de Tours, Tours, France; ^3^Service d’information médicale, épidémiologie et économie de la santé, CHRU Hôpitaux de Tours, Tours, France

####### **Correspondence:** Youenn Jouan - youenn.jouan@gmail.com

*Annals of Intensive Care* 2017, **7(Suppl 1)**:O16

**Introduction** Intensive care unit (ICU) admission is known to lead, among survivors, to numerous and persistent disabilities and impairments after discharge, forming the “post intensive care syndrome” (PICS). However, PICS consequences in term of healthcare use is less studied. Recent data tend to demonstrate that ICU survivors have increased healthcare use after ICU. However, little is known about healthcare use for the most severely ill patients, which are supposed to be at higher risk of PICS, and, to our knowledge, no epidemiological data are available in France.

**Patients and methods** We conducted a retrospective multicenter study using comprehensive administrative hospital discharge databases of the *Centre Val de Loire region, France* (2.5 millions of inhabitants). Based on an ICD-10 algorithm, we included all adult patients admitted in an ICU for septic shock or acute respiratory distress syndrome (ARDS) during 2011 and invasively ventilated at least 5 days. Performance of the selection algorithm was validated through review of a subsample of medical charts. Comorbidities were also extracted from ICD-10 coding and reported using a scoring system derived from Charlson Comorbidity Index. Healthcare use and comorbidities were analyzed 2 years before (pre-ICU period) and 2 years after ICU (post-ICU period).

**Results** 552 patients were selected, of which 249 (45%) died during the hospital stay. Among the 303 survivors, 293 (97%) had complete data required for analysis and none was lost for follow up. Mean ± SD age was 61 ± 14 years, SAPS2 49 ± 17 and median ventilation duration was 10 days (Q1 = 7; Q3 = 20). Regarding chronic comorbidities during the pre-ICU period, cardiac disease was reported for 26% of the patients, respiratory disease for 16%, kidney disease for 13%, and hepatic disease for 12%.

Healthcare resources utilization analysis during the pre-ICU period revealed that 58% of the patients required hospitalization, 54% ambulatory care, 57% emergency admissions and 10% rehabilitation facilities. Twenty-three percent of the patients had no healthcare use.

During the post-ICU period, the 2-year mortality rate was 15%. Healthcare resources utilization was significantly increased during the post-ICU period compared to the pre-ICU period for hospitalizations (72%, p < 0.001), ambulatory care (73%, p < 0.001) and rehabilitation facilities (54%, p < 0.001). No patient had no healthcare use. Regarding chronic comorbidities, cardiac, respiratory and renal diseases were significantly more frequent compared to the pre-ICU period (respectively 32, 27, and 21%, p < 0.001 for the three conditions). Time trend analysis of the healthcare use in the post-ICU period revealed that the first 9 months were at high healthcare use (essentially hospitalizations and rehabilitation facilities), and emergency admissions tended to increase at the end of the 2-years follow-up.

**Discussion** Patients admitted to ICU for acute respiratory distress syndrome and septic shock frequently have a significant healthcare resources utilization during the 2 years before. The 2 years following admission is characterized by a more important healthcare use, together with a significant increase in comorbidities.

**Conclusion** Our study highlights the epidemiological impact of PICS at the population level in a French region, underpinning observational and interventional research within and beyond the ICU.

**Competing interests** None.

#### O17 A pilot study of 6-months evaluation of social, psychological, financial and emotional consequences of an ICU stay in survivors critically ill patients

##### Cécile Rebière^1^, Elie Azoulay^2^, Benoit Misset^3^, Stephane Ruckly^4^, Jean-François Timsit^5^, Maïté Garrouste-Orgeas^1^

###### ^1^Réanimation, Fondation Hopital Saint Joseph, Paris, France; ^2^Réanimation médicale, Hôpital Saint-Louis, Paris, France; ^3^Réanimation médicale, Hospital Center University, Rouen, France; ^4^Statistics Department, Outcomerea Research Group, Paris, France; ^5^Réanimation médicale et infectieuse, Hôpital Bichat-Claude Bernard, Paris, France

####### **Correspondence:** Maïté Garrouste-Orgeas - mgarrouste@hpsj.fr

*Annals of Intensive Care* 2017, **7(Suppl 1)**:O17

**Introduction** Consequences of hospitalization in critically ill patients have been recognized for several years with physical, cognitive and psychological consequences published under the denomination of the post intensive care syndrome which will become a new challenge for intensivists. The impact of social, personal and financial consequences has been less reported. The primary objective of this pilot study is to report the social, financial, psychological, physical and emotional consequences in a group of critically ill patients compared to a group of patients never hospitalized in ICU. The second objective is to investigate patient’s perceptions to better understand their memories of their hospitalization through a qualitative approach.

**Patients and methods** We designed a case control study in three ICUs belonging to the Outcomerea research group (July 2014–May 2015). Case patients were adult patients ventilated for more than 48 h. We excluded patients not speaking or understanding French and patients who denied participation. Clinical and demographics characteristics of the cases were extracted from the Outcomerea database. They were interviewed 6-months after ICU discharge. Control patients, matched on age and sex and never hospitalized in ICU, were interviewed face-to-face during an hospital consultation. All patients completed the same questionnaires in a random order, exploring emotional and post traumatic-stress syndrome (Impact of Event Scale-revised, cut-off >22), self-sufficiency in daily activities (activity of daily Living, ADL), quality of life (first question of the SF-36), and questions about their place of living, of working and financial conditions, need of psychological help and marital status. We used a phenomenological approach to report patient’s perceptions.

**Results** Of the 96 eligible patients, 20 (20.8%) died at 6 months, 39 (40.6%) were excluded and 37 were entered in the analysis and compared to 37 control patients. Characteristics of the case patients were: age (median: 65, range: 47–73), 64% male, SAPS II (51, 37–64), ICU stay (12 days, 8–19), hospital stay (29 days, 22–41). The median IES-R score was significantly higher in cases (14, 8–31) vs control (6, 3–10), p < 0.01. IES-R > 22 was found in 13 (35.1%) cases patients versus 1 (2.7%) of control patients (p < 0.01). Activities of daily living without help were significantly most often performed in control versus cases for bathing (n = 37, 100% vs n = 33, 89%, p = 0.04) and continence (n = 37, 100% vs n = 33, 89% p = 0.04). Perception of the quality of life was not significantly different between cases and controls but increasing quality of life was much important in cases (n = 15, 40.6%) versus control (3, 8.1%), p < 0.01. See Table 4.

**Table 4:** Functional and personal changes in cases and controls

Variables	Cases n = 37	Control n = 37	p value
Return home within 1 month	17 (45.9%)	37 (100%)	<0.01
Need of help at home	22 (59.4%)	5 (13.5%)	<0.05
Change in working conditions	12 (32.4%)	3 (8.1%)	<0.05
Change in financial conditions	10 (27%)	7 (18.9%)	0.6
Psychologist help	17 (45.9%)	5 (13.5%)	<0.05
Use of medications	9 (24.3%)	8 (21.6%)	1
Change in marital status	2 (5.4%)	3 (8.1%)	1
Eating disorders	8 (21.6%)	5 (13.5%)	0.54
Disorder of sexual life	12 (32.4%)	7 (18.9%)	0.3

Three themes were found in the qualitative analysis: the ICU stay seen as a traumatized period, a period without memories and support from families and friends. Their representations in the verbatims were 13 (35.1%), 13 (35.1%) and 3 (8.1%).

**Conclusion** This pilot study reported substantial neuropsychological and functional alterations related to the ICU stay and emphasized the need for better estimating these modifications in a multicenter study. Addressing these consequences adds to the role of intensivists for elaborating prevention programs and promoting post intensive care syndrome to non ICU practitioners to collaborate together for the best future of ICU patients.

**Competing interests** None.

**References**

1. Harvey MA, Davidson JE. Postintensive care syndrome: right care, right now and…later. Crit Care Med. 2016;44:381–5.
2. Needham DM, Davidson J, Cohen H, Hopkins RO, Weinert C, Wunsch H, et al. Improving long-term outcomes after discharge from intensive care unit: report from a stakeholders’ conference. Crit Care Med. 2012;40:502–9.

#### O18 To understand or not to understand brain death: impact on grief symptoms in relatives who experienced organ donation request

##### Nancy Kentish-Barnes^1^, Jacques Duranteau^2^, Julien Charpentier^3^, Marie Thuong^4^, Liliane Joseph^2^, Laurent Martin-Lefèvre^5^, Anne Renault^6^, Olivier Lesieur^7^, Anne-Gaelle Si Larbi^8^, Gérald Viquesnel^9^, Benjamin Zuber^10^, Sophie Marque^11^, Stanislas Kandelman^12^, Nicolas Pichon^13^, Bernard Floccard^14^, Marion Galon^15^, Sylvie Chevret^16^, Elie Azoulay^17^

###### ^1^Réanimation médicale, Assistance Publique Hôpitaux de Paris, Hôpital Saint Louis, Paris, France; ^2^Réanimation chirurgicale, Hôpital Bicêtre, Le Kremlin-Bicêtre, France; ^3^Réanimation Médicale, Hôpital Cochin, Paris, France; ^4^Val d ‘oise, CH René Dubos, Pontoise, France; ^5^Réanimation polyvalente, Centre Hospitalier Départemental - site de La Roche-sur-Yon, La Roche-sur-Yon, France; ^6^Réanimation médicale, CHU Brest, Brest, France; ^7^Réanimation, Centre Hospitalier la Rochelle, La Rochelle, France; ^8^92151, Hospital Foch, Suresnes, France; ^9^Réanimation chirurgicale, C.H.U de Caen, Caen, France; ^10^Intensive care unit, Hospital Center De Versailles, Le Chesnay, France; ^11^ICU, C.H. Sud Francilien, Corbeil-Essonnes, France; ^12^Anesthésie réanimation, Hôpital Beaujon (AP-HP), Clichy, France; ^13^Service de réanimation polyvalente, Centre Hospitalier Universitaire de Limoges, Limoges, France; ^14^Département anesthésie-réanimation, Hôpital Édouard Herriot, Lyon, France; ^15^Réanimation médicale, AP-HP Hôpital Saint Louis, Paris, France, France; ^16^Service de biostatistique et information médicale, Hôpital Saint-Louis, Paris, France; ^17^Réanimation médicale, Hôpital Saint-Louis, Paris, France

####### **Correspondence:** Nancy Kentish-Barnes - nancy.kentish@aphp.fr

*Annals of Intensive Care* 2017, **7(Suppl 1)**:O18

**Introduction** In the ICU context, in the case of organ donation, patients’ relatives are at the centre of the decision process: within a limited time frame, they will be told that the patient is brain dead and will be asked to consider organ donation. Qualitative studies have put forward that understanding brain death facilitates decision-making and impacts on the final decision (donation vs non donation). However the impact of understanding brain death on relatives’ grieving process has never been evaluated. In this study, we searched for correlation between semi-quantitative answers to questions related to understanding of brain death and experience of the process in a questionnaire completed by relatives 1 month after the patient’s death and post-traumatic related symptoms (PTSD) and complicated grief.

**Patients and methods** This is an ancillary study of a larger prospective, observational study in 28 ICUs in France that aimed to compare grief symptoms of relatives of donor patients versus relatives of non-donor patients. For each brain dead patient, the relative who served as the surrogate was included at time of organ donation discussion. Relatives were assessed at 3 time points during a telephone interview: at 1 month, to complete a questionnaire regarding their experience in the ICU and description of the organ donation request and procedure, including understanding of brain death; at 3 months to complete the Hospital Anxiety and Depression Scale (HADS) and the Impact of Event Scale-Revised (IES-R) for PTSD symptoms; at 9 months, to complete the IES-R and the Inventory of Complicated Grief (ICG).

**Results** 202 relatives were included in the study. At 1 month after the patient’s death, 79.2% of relatives completed the questionnaire, at 3 months 70.3% completed the HADS and the IES-R and at 9 months 61.4% completed the IES-R and the ICG.

One month after the death, 35% of relatives declared having difficulties in understanding brain death and 32% experienced decision-making as difficult. Results show that experience of the decision making process impacts on relatives’ well-being. At 3 months, compared to relatives who did not find the decision difficult, those who did find it difficult more often presented significant PTSD symptoms (40.54 vs 65.88%, p = 0.016). At 9 months, compared to relatives who understood brain death, those who did not understand brain death had higher global ICG score [23 (12.5–36.5) vs 36 (28–43.75), p = 0.010] and more often presented complicated grief symptoms (46.15 vs 75%, p = 0.026). There was a trend in increased prevalence of PTSD related symptoms with 60% in the group of relatives who did not understand brain death versus 47.2% in the group that did, but this was not significant (p = 0.33).

**Discussion** Results show that difficulty experienced during organ donation discussion and decision impacts on relatives’ well-being in the months that follow the patient’s death. Support to relatives should be proposed in this context. Interestingly, understanding of brain is a key component of relatives’ experience: on top of possibly impacting on the decision itself, it significantly impacts on relatives’ grieving process 9 months after the patient’s death. Promoting better understanding of brain death, proposing clearer explanations, by using various media, may improve both relatives’ understanding and well-being.

**Conclusion** Our study shows that understanding of brain death is a key component of relatives’ experience that significantly impacts on the grieving process. Efforts should be made to improve relatives’ understanding of brain death.

**Competing interests** None.

#### O19 Impact of ICU end-of-life care on relatives’ grief symptoms

##### Nancy Kentish-Barnes^1^, Valérie Seegers^2^, Stéphane Legriel^3^, Alain Cariou^4^, Samir Jaber^5^, Jean Yves Lefrant^6^, Bernard Floccard^7^, Anne Renault^8^, Isabelle Vinatier^9^, Armelle Mathonnet^10^, Danielle Reuter^11^, Olivier Guisset^12^, Christophe Cracco^13^, Amélie Seguin^14^, Jacques Durand-Gasselin^15^, Marine Thirion^16^, Zoé Cohen-Solal^1^, Elie Azoulay^11^

###### ^1^Réanimation médicale, Assistance Publique Hôpitaux de Paris, Hôpital Saint Louis, Paris, France; ^2^Umr 669, Inserm, Paris, France; ^3^Réanimation, Centre Hospitalier de Versailles, Le Chesnay, France; ^4^Réanimation Médicale, Hôpital Cochin, Paris, France; ^5^DAR B, Hôpital Saint Eloi, Montpellier, France; ^6^Réanimation chirurgicale, Hopital Carémeau, Nîmes, France; ^7^Département anesthésie-réanimation, Hôpital Édouard Herriot, Lyon, France; ^8^Réanimation médicale, CHU Brest, Brest, France; ^9^Réanimation polyvalente, Hopital Les Oudaries, La Roche-sur-Yon, France; ^10^Réanimation médicale, Centre Hospitalier Régional d’Orléans, Orléans, France; ^11^Réanimation médicale, Hôpital Saint-Louis, Paris, France; ^12^Réanimation médicale-hôpital saint-andré, Centre Hospitalier Universitaire de Bordeaux, Bordeaux, France; ^13^Service de reanimation, Service de Réanimation polyvalente, Angoulême, France; ^14^Réanimation médicale, Centre Hospitalier Universitaire de Caen, Caen, France; ^15^Réanimation, Hôpital Sainte-Musse, Toulon, France; ^16^Val d’oise, Centre hospitalier Victor Dupouy (Argenteuil), Argenteuil, France

####### **Correspondence:** Nancy Kentish-Barnes - nancy.kentish@aphp.fr

*Annals of Intensive Care* 2017, **7(Suppl 1)**:O19

**Introduction** Relatives of patients who die in the ICU experience a considerable burden of harm such as symptoms of anxiety and depression, posttraumatic stress disorder (PTSD) symptoms and quality-of-life alterations. Improving the quality of dying and death is recognized as a priority. Nevertheless, specific data are needed to understand what specific aspects of ICU care affect the relatives’ grieving process. This study aims at providing information on potential links between anxiety/depression, PTSD related symptoms, complicated grief and components of ICU end-of-life care in order to specify ICU practices that may affect the risk of developing these symptoms.

**Patients and methods** This is an ancillary study of the CAESAR study—a prospective, observational study in 41 ICUs in France. Eligible patients were adults who died after at least 48 h in the ICU. For each patient, the relative who served as the surrogate was included at time of death. Relatives were assessed 21 days then 3, 6, and 12 months after the death during a telephone interview. At 21 days they completed the CAESAR scale; at 3 months, they completed the Hospital Anxiety and Depression Scale (HADS) and the Impact of Event Scale-Revised (IES-R) and at 6 and 12 months they completed the IES-R and the Inventory of Complicated Grief. In this study, we searched for correlation between semi-quantitative answers to CAESAR questions (and not scores) and outcomes.

**Results** 475 patients and their relatives were included. Response rates were 90.5, 81.3, 59.4 and 45.2%, at day-21, 3, 6 months and at 1 year, respectively.

5 domains are associated with significant increased risk of developing ICU burden (p ≤ 0.05 for each variable).

1. *Quality of care and symptom control.* Perception that pain was not under control and that the patient had difficulties in breathing is associated with increased risk of developing anxiety and depression at 3 months, PTSD related symptoms at 6 and 12 months, complicated grief at 6 months. Dissatisfaction with quality of care is associated with increased risk of developing anxiety and depression at 3 months, PTSD related symptoms at 3 months, complicated grief at 12 months.
2. *Quality of communication.* Dissatisfaction with communication with either doctors or nurses is associated with increased risk of developing anxiety and depression at 3 months, PTSD related symptoms at 3 and 6 months, complicated grief at 6 months.
3. *Kindness.* Perception that the team was not kind enough is associated with increased risk of developing anxiety and depression at 3 months, PTSD related symptoms at 3, 6 and 12 months.
4. *Preparation for death*. Relatives who were not informed that the patient was dying, who were unable to express important things or to say goodbye were more at risk of developing anxiety and depression at 3 months, PTSD related symptoms at 3, 6 and 12 months and complicated grief at 6 and 12 months.
5. *Presence at time of death* is associated with increased risk of developing PTSD related symptoms and complicated grief at 6 and 12 months.

**Discussion** Relatives are sensitive to interaction between the ICU team and themselves as well as between the team and the patient. Quality of communication (both verbal and non verbal) and support, as well as preparation for the death, are key components of relatives’ experience that impact on grief symptoms in the months that follow the patient’s death.

**Conclusion** Quality of care and support during the dying process are at the heart of the relatives’ experience. This study puts forward practices that may be improved in order to promote both palliative care and family centered care in the ICU and, *in fine*, decrease grief symptoms in bereaved relatives.

**Competing interests** None.

#### O20 Complicated grief after organ donation of brain-dead patients: evaluation of the donors’relatives 6 months after the death

##### Hélène Foulgoc^1^, Julien Rogier^1^, Elsa Delobbe^1^

###### ^1^33, CHU - Hôpitaux de Bordeaux, Bordeaux, France

####### **Correspondence:** Hélène Foulgoc - helene_f@hotmail.fr

*Annals of Intensive Care* 2017, **7(Suppl 1)**:O20

**Introduction** In 2015, 1824 organ donations were performed in France. We already know that complicated grief after the death of a relative in the intensive care unit is frequent but the population of organ donors’relatives hasn’t been studied. Currently, there doesn’t exist any sector to orientate donors’ families. The purpose of our study is to investigate the complicated grief donors’ relatives, its associated diseases and risk factors.

**Patients and methods** Prospective, single-center, observational study. During the first meeting with the transplantation coordination team of our hospital, it was proposed to the organ donors’ relatives to participate. The relatives who had accepted received the ICG, IDS-SR and PCLS questionnaires at 1, 3 and 6 months after the death. The primary endpoint was the presence of complicated grief, defined by an ICG > 25, among the relatives of organ donors after 6 months from the death. The secondary end points were the presence of major depressive disorders (IDS-SR ≥ 15) and post-traumatic stress disorders (PTSD) (PCLS ≥ 44) at 6 months and the analysis of complicated grief‘s risk factors: about the deceased person (age, sex, cause of the death, place of the death, time between the hospital’s entrance and the death) and about the relative (sex, age, relationship with the donor, occupational category, PCLS ≥ 44 and IDS-SR ≥ 15 6 months after the death).

**Results** From December 2014 to January 2016, 81 donors’ relatives were included. An average of 3.24 relatives per donor was included. 16 of the 29 relatives who responded at 6 months had an ICG > 25. The prevalence of complicated grief is 55.2% [95 CI = 37–73]. The prevalence of major depressive disorder and PTSD are respectively 72% [95 CI = 55.7–88.3] and 31% [95 CI = 14.2–47.8] (Table 1). No variable were significantly associated with a complicated grief at 6 months from the death.

**Discussion** Even if only 36% of the relatives completed the scales at 6 months, this study shows a high risk of complicated grief among donor’s relatives. But we can see that the prevalence of complicated grief in this population is closed to the prevalence find after the death of a relative in the intensive care unit. Because of the lake of responses at 6 months, we can’t conclude on the risk factors.

**Conclusion** 55.2% of donor’s relatives developed a complicated grief 6 months after the death. The establishment of dedicated channels to orientate these bereaved persons seems important. Further studies should be conducted to define better the complicated grief’s risk factors following an organ donation.

**Competing interests** None.

**References**

1. Prigerson HG, Maciejewski PK, Reynolds CF, Bierhals AJ, Newsom JT, Fasiczka A, et al. Inventory of complicated grief: a scale to measure maladaptive symptoms of loss. Psychiatry Res. 1995;59(1–2):65–79.
2. Kentish-Barnes N, Chaize M, Seegers V, Legriel S, Cariou A, Jaber S, et al. Complicated grief after death of a relative in the intensive care unit. Eur Respir J. 2015.

#### O21 Impact of fluid-induced hyperchloremia on acid base balance and outcomes in septic shock: post hoc analysis of the “Hyper2S”study

##### Frédérique Schortgen^1^, Pierre Asfar^2^, Boisramé-Helms Julie^3^, Julien Charpentier^4^, Emmanuel Guérot^5^, Bruno Megarbane^6^, David Grimaldi^7^, Grelon Fabien^8^, Nadia Anguel^9^, Lasocki Sigismond^10^, Henry-Lagarrigue Matthieu^11^, Frédéric Gonzalez^12^, Legay François^13^, Christophe Guitton^14^, Maleka Schenck^15^, Doise Jean-Marc^16^, Didier Dreyfuss^17^, Peter Radermacher^18^, for the HYPER2S Investigators and REVA Research Network

###### ^1^Réanimation médicale, Hôpital Henri-Mondor (AP-HP), Créteil Cedex, France; ^2^Réanimation, C.H.U. d’Angers, Angers, France; ^3^Réanimation médicale, Nouvel Hôpital Civil, Hôpitaux Universitaires de Strasbourg, Strasbourg, France; ^4^Réanimation Médicale, Hôpital Cochin, Paris, France; ^5^Réanimation médicale, Hopital Europeen Georges-Pompidou, Paris, France; ^6^Service de Réanimation Médicale et Toxicologique, CHU Lariboisière, Paris, France; ^7^Service de réanimation polyvalente, Centre Hospitalier de Versailles, Le Chesnay, France; ^8^Réanimation, C.H. - Le Mans, Le Mans, France; ^9^Réanimation médicale, CHU de Bicêtre, Le Kremlin Bicêtre, France; ^10^Réanimation chirurgicale, C.H.U. d’Angers, Angers, France; ^11^Réanimation polyvalente, Hospital Center Departmental De Vendée, La Roche-sur-Yon, France; ^12^Réanimation medico-chirurgicale, hopital avicenne, Bobigny, France; ^13^Réanimation, C.H. de Saint Brieuc, Saint-Brieuc, France; ^14^Réanimation médicale, C.H.U. Hôtel Dieu, Nantes, France; ^15^Réanimation médicale, C.H.R.U. Hôpitaux Universitaires Strasbourg, Strasbourg, France; ^16^Réanimation, C.H. Chalon sur Saône William Morey, Chalon-sur-Saône, France; ^17^Réanimation polyvalente, Hôpital Louis-Mourier (AP-HP), Colombes cedex, France; ^18^Institut für anästhesiologische pathophysiologie und verfahrensentwicklung, Universitätsklinikum Ulm, Ulm, Germany

####### **Correspondence:** Frédérique Schortgen - frederique.schortgen@aphp.fr

*Annals of Intensive Care* 2017, **7(Suppl 1)**:O21

**Introduction** The harmfulness of fluid-induced hyperchloremia (H-Cl) remains debated. Large randomized trial showed that chloride-rich crystalloids did not worsen outcome [1]. The volume of fluids, however, was limited and the incidence of H-Cl was not recorded.

**Patients and methods** In a post hoc analysis of the database of the RCT “Hyper2S”, a study comparing normal to 3% hypertonic saline for 72 h in 434 patients with septic shock, we assessed the incidence and the impact of H-Cl (≥110 mmol/L) on adverse events and mortality. Cl, pH, bicarbonate (Bic) and lactate (Lac) were recorded at H0-12-24-72. Episodes of hyperlactatemia (H-Lac > 2 mmol/L), metabolic acidosis (pH < 7.35 + Bic < 22) either H-Cl (pH < 7.35 + Bic < 22 + Cl ≥ 110 + Lact ≤ 2) or H-Lac (pH < 7.35 + Bic < 22 + Lact > 2) were recorded. Acute kidney injury (AKI) was defined by doubling creatinine or dialysis.

**Results** 413 patients without missing data were analysed. H-Cl and HCl-acidosis were recorded in 257 (62%) and 77 (19%) patients, respectively, H-Lact and H-Lact-acidosis in 294 (71%) and 209 (51%) patients, respectively. Baseline severity scores were similar in patients with and without H-Cl but vasopressor dose was higher in patients with H-Cl (Table 5). The chloride load was significantly higher in patients with H-Cl (Table 5). Both, H-CL acidosis and H-Lac acidosis were more frequent in patients with than without H-Cl episode (Table 5). H-Cl was not associated with AKI or mortality (Table 5). The mortality of patients who experienced H-Cl acidosis (25%) was similar to patients who never experienced metabolic acidosis (28%), p = 0.65.

**Table 5:** Characteristics and outcome of patients with and without H-Cl (results are number and % of patients and medians IQR)

	No H-Cl (n = 156)	H-Cl (n = 257)	P
Characteristics at inclusion			
SAPS II (points)	55 (46–63)	56 (48–65)	0.65
SOFA (points)	10 (8–12)	10 (8–12)	0.31
Vasopressor dose (µg/kg/min)	0.32 (0.20–0.66)	0.44 (0.23–0.80)	0.04
Serum creatinine (µmol/L)	137 (79–208)	132 (80–191)	0.31
Evolution from H0 to H72			
Volume of fluid resuscitation (L)	1.4 (0.6–2.3)	2.2 (1.1–3.9)	<0.001
Chloride load (all fluids) (mmol)	287 (129–491)	690 (293–1127)	<0.001
Mean pH	7.36 (7.29–7.40)	7.32 (7.26–7.37)	<0.001
≥1 episode metab. acidosis [n (%)]	82 (53)	204 (79)	<0.001
≥1 episode H-Cl acidosis [n (%)]	NA	77 (30)	–
≥1 episode H-Lact acidosis [n (%)]	41 (26)	149 (58)	<0.001
Outcomes			
AKI [n (%)]	96 (64)	146 (58)	0.42
D28 mortality [n (%)]	60 (38)	95 (37)	0.76

**Conclusion** H-Cl is frequent in septic shock patients resuscitated with chloride-rich fluids but does not increase AKI or mortality. H-Lact is more frequent in patients with H-Cl, which is an important bias for the interpretation of the origin of acidosis and attributable mortality.

**Competing interests** None.

**Reference**

1. Young P. JAMA. 2015;314:1701–10.

#### O22 Grief symptoms in relatives of brain dead patients: comparison of relatives of donor and non-donor patients

##### Nancy Kentish-Barnes^1^, Jacques Duranteau^2^, Julien Charpentier^3^, Marie Thuong^4^, Liliane Joseph^2^, Laurent Martin-Lefèvre^5^, Anne Renault^6^, Anne-Gaelle Si Larbi^7^, Olivier Lesieur^8^, Gérald Viquesnel^9^, Benjamin Zuber^10^, Sophie Marque^11^, Stanislas Kandelman^12^, Nicolas Pichon^13^, Bernard Floccard^14^, Marion Galon^15^, Sylvie Chevret^16^, Elie Azoulay^17^

###### ^1^Réanimation médicale, Assistance Publique Hôpitaux de Paris, Hôpital Saint Louis, Paris, France; ^2^Réanimation chirurgicale, Hôpital Bicêtre, Le Kremlin-Bicêtre, France; ^3^Réanimation Médicale, Hôpital Cochin, Paris, France; ^4^Val d ‘oise, CH René Dubos, Pontoise, France; ^5^Réanimation polyvalente, Centre Hospitalier Départemental - site de La Roche-sur-Yon, La Roche-sur-Yon, France; ^6^Réanimation médicale, CHU Brest, Brest, France; ^7^92151, Hospital Foch, Suresnes, France; ^8^Réanimation, Centre Hospitalier la Rochelle, La Rochelle, France; ^9^Réanimation chirurgicale, C.H.U de Caen, Caen, France; ^10^Intensive Care Unit, Hospital Center De Versailles, Le Chesnay, France; ^11^ICU, C.H. Sud Francilien, Corbeil-Essonnes, France; ^12^Anesthésie réanimation, Hôpital Beaujon (AP-HP), Clichy, France; ^13^Service de réanimation polyvalente, Centre Hospitalier Universitaire de Limoges, Limoges, France; ^14^Département anesthésie-réanimation, Hôpital Édouard Herriot, Lyon, France; ^15^Réanimation médicale, AP-HP Hôpital Saint Louis, Paris, France, France; ^16^Service de biostatistique et information médicale, Hôpital Saint-Louis, Paris, France; ^17^Réanimation médicale, Hôpital Saint-Louis, Paris, France

####### **Correspondence:** Nancy Kentish-Barnes - nancy.kentish@aphp.fr

*Annals of Intensive Care* 2017, **7(Suppl 1)**:O22

**Introduction** Long after the death of a loved one, end-of-life decisions can remain with the living and have been implicated in post ICU burden. In the case of organ donation, the relatives are at the centre of the decision process: within a limited time frame, they will be told that the patient is brain dead and will be asked to consider organ donation. Attention has been focused on how relatives make the decision to donate or not to donate the patient’s organs but only very few studies have focused on the impact of organ donation decision making on their psychological well-being during the months that follow the patient’s death. The goal of this study was to describe the grieving process of relatives who were approached about organ donation in the context of brain death and to compare grief symptoms of relatives of donor patients (DPs) versus relatives of non-donor patients (NDPs).

**Patients and methods** We conducted a prospective, observational study in 28 ICUs in France. For each brain dead patient, the relative who served as the surrogate was included at time of organ donation discussion. Relatives were assessed at 3 time points during a telephone interview: At 1 month, to complete a questionnaire regarding their experience in the ICU; at 3 months to complete the Hospital Anxiety and Depression Scale (HADS) and the Impact of Event Scale-Revised (IES-R) for PTSD symptoms; at 9 months, to complete the IES-R and the Inventory of Complicated Grief (ICG). The primary outcome measure was the IES-R (at 3 and 9 months).

**Results** 202 relatives were included in the study among which 158 were relatives of DPs and 44 of NDPs. At 1 month after the patient’s death, 79.2% of relatives completed the questionnaire, at 3 months 70.3% completed the HADS and the IES-R and at 9 months 61.4% completed the IES-R and the ICG.

Relatives’ experience of ICU and decision-making varies between the 2 groups. Relatives of NDPs are less satisfied with communication with ICU team than relatives of DPs (26.6 vs 8%, p = 0.021). Relatives of NDPs were more often shocked by organ donation request than relatives of DPs (64.52 vs 19%, p < 0.0001). During organ donation discussions, relatives of NDPs more often declared an absence of support from the ICU team (19.35 vs 1.59%, p = 0.0008) and more often felt under pressure (41.94 vs 7.14%, p < 0.0001).

At 3 months, there was no difference in the IES-R score between relatives of DPs (31 [21–41]) and NDPs (31.5 [11.25–34]) (p = 0.29). Similarly there were no differences in HADS score (13 [9–20] and 13.5 [8.25–20] respectively, p = 1.00) and anxiety and depression subscores. At 9 months, there was no difference in the IES-R score between relatives of DP (26 [12.75–38]) and NDP (33.5 [21.25–43.25]) (p = 0.17). Similarly there were no differences in ICG score (25.5 [14.37–25] and 32.5 [17.43–25] respectively, p = 0.11).

**Discussion** Relatives of DPs and NDPs have different experience of quality of communication and quality of support during the patient’s ICU stay. More than the decision itself, quality of the organ donation process impacts on relatives’ grief symptoms.

**Conclusion** The decision (donation vs no donation) has no impact on grief symptoms in the months following the patient’s death. However, experience of the request and of the decision itself, as well as quality of communication and support, are elements that effect relatives’ experience and grieving process.

**Competing interests** None.

#### O23 Implementation and impact of the surviving sepsis campaign protocol: results of a quasi-experimental study in Democratic Republic of Congo (DRC)

##### Joseph Nsiala Makunza^1^, Mejeni Kamdem Nathalie^1^, Akilimali Pierre^2^, Kilembe Manzanza Adolphe^3^

###### ^1^Anesthésie-Réanimation, Cliniques universitaires de Kinshasa, Kinshasa, Democratic Republic of the Congo; ^2^Statistics, School Of Public Health, Kinshasa, Democratic Republic of the Congo; ^3^Anesthéthésie-réanimation, Cliniqes Universitaires de Kinshasa, Kinshasa, Democratic Republic of the Congo

####### **Correspondence:** Joseph Nsiala Makunza - mnsiala78@gmail.com

*Annals of Intensive Care* 2017, **7(Suppl 1)**:O23

**Introduction** In developed countries, the application of the “Surviving Sepsis Campaign” (SSC) protocols in the management of sepsis and septic shock has been associated with increased survival [1].

In the context of a fragile health system, the application of these protocols may be expected to prove to be difficult to undertake and the impact may also be expected to vary [2].

**Patients and methods** We conducted a prospective, *quasi*-*experimental before and after study* in the university hospital of Kinshasa (DRC) comparing 33 consecutive patients in septic shock treated according to usual care versus 39 patients treated according to a protocol type EGDT from 1st February 2014 to 31st July 2014 (pre- protocol phase) and 1st September 2014 to 28th February 2015 (post- protocol phase). Between the 2 phases, we have drawn and implemented a local protocol based on SSC recommendations. A kit consisting of central venous catheters, devices capable of measuring central venous pressure, flow regulator (Dosiflow R) and lactate reader was available.

In the absence of electrically driven syringe pumps, catecholamines were administered by continuous perfusion using a flow regulating device (Dosiflow). We drew up calculating tables based on a standard dilution and a variable administration rate controlled by the flow regulating device, thus permitting precise and constant dose administration in millilitres/hour (ml/h).

The main outcome measures were: the rate of compliance with 6-h sepsis care bundles and the hospital mortality until J30. The Student’s and U Mann–Whitney tests were used to compare quantitative variables, and the chi-square test to compare qualitative variables. Kaplan–Meier survival curves were used and the differences between the two curves were analyzed using the log rank test.

This study received the approval of ethics committee and was registered 23th January 2014 under number ESP/CE/053/14.

**Results** Baseline characteristics of patients were similar in both two groups. Lung infection was the main source of septic shock and antibiotics administration delay was less than 3 h in the two groups. In contrast, blood cultures and blood lactate were performed only in patients of the EGDT group.

Patients of the EGDT group received more IV fluids (+1226 ml on average), more catecholamines (+24, 9%) and were more often transfused (+13, 7%) than the patients treated in the habitual fashion. Protocol compliance was substantially improved, passing from 0 to 50%. The absolute risk reduction of mortality was 17% (100 vs 83%; p = 0.0037) when all the therapeutic measures had been employed.

The Kaplan–Meier survival curves showed that the patients in the pre-protocol group had a significantly greater risk of dying compared with those in the post-protocol group. The average length of stay was 2.2j vs 5.2j p = 0.0037.

**Discussion** Our results in terms of compliance with the sepsis bundle were satisfying given that even in highly developed countries the compliances rates are often below 50% [3]. Ignorance of the very existence of these recommendations and logistical factors are often quoted as the reasons why compliance is so poor

The positive impact that we observed on mortality was found in similar studies [4].

Other more recent randomized controlled studies have not found significant differences but this may be explained by the similarity of treatment protocols in the two groups [5].

Our study is coherent according to current literature but there exist certain limits which might be expected in this kind of study.

Our research was implemented in only one centre and these results cannot be extrapolated to all sub-Saharan Africa, nor even to our own country. As in all before/after studies, comparison with a historic control group is liable to introduce a bias based on confusion. We sought to minimize this bias however by choosing a short study period (12 months) with the same therapeutic team during the entire study.

**Conclusion** Despite a difficult socio-economic context, the implementation of a local protocol based on the recommendations of the SSC was associated with improved outcome in septic shock patients in our hospital compared to usual care.

We thus intend to continue and to widen the application of this protocol in an effort to evaluate if this positive effect will be observed in a greater number of patients.

**Competing interests** None.

**References**

1. Levy MM, Dellinger RP, Townsend SR, Linde-Zwirble WT, Marshall JC, Bion J, et al. The Surviving Sepsis Campaign: results of an international guideline-based performance improvement program targeting severe sepsis. Crit Care Med. 2010;38(2):367–74.
2. Baelani I, Jochberger S, Laimer T, Otieno D, Kabutu J, Wilson I, Baker T, Dünser MW. Availability of critical care resources to treat patients with severe sepsis or septic shock in Africa: a self-reported, continent-wide survey of anaesthesia providers. Crit Care. 2011;15(1):R10.
3. Rhodes A, Phillips G, Beale R, Cecconi M, Chiche JD, De Backer D, et al. The Surviving Sepsis Campaign bundles and outcome: results from the International Multicentre Prevalence Study on Sepsis (the IMPreSS study). Intensive CareMed. 2015; 41(9):1620–8.
4. Levy MM, Rhodes A, Phillips GS, Townsend SR, Schorr CA, Beale R, et al. Surviving Sepsis Campaign: association between performance metrics and outcomes in a 7.5-year study. Crit Care Med. 2014;40:1623–33 5.
5. Angus DC, Barnato AE, Bell D, Bellomo R, Chong CR, Coats TJ, Davies A, et al. A systematic review and meta-analysis of early goal-directed therapy for septic shock: the ARISE, ProCESS and ProMISe Investigators. Intensive Care Med. 2015; 41(9):1549–60.

#### O24 Characteristics and 1-year prognosis of tetanus patients admitted to the ICU

##### Rafael Mahieu^1^, Thomas Reydel^1^, Adel Maamar^2^, Angéline Jamet^3^, Nicolas Chudeau^4^, Julien Huntzinger^5^, Steven Grange^6^, Anne Courte^7^, Stephan Ehrmann^8^, Jérémie Lemarie^9^, Sébastien Gibot^9^, Michaël Darmon^10^, Christophe Guitton^11^, Julia Champey^12^, Jean Dellamonica^13^, Ferhat Meziani^14^, Damien Du Cheyron^15^, Nicolas Lerolle^1^

###### ^1^Réanimation médicale, Centre Hospitalier Universitaire d’Angers, Angers, France; ^2^Réanimation médicale, Centre hospitalier universitaire de Rennes, Rennes, France; ^3^Réanimation médicale, CHU de Poitiers, Poitiers, France; ^4^Réanimation médico-chirurgicale, C.H. - Le Mans, Le Mans, France; ^5^Réanimation médicale, Centre hospitalier Bretagne Atlantique, Vannes, France; ^6^Réanimation médicale, Centre Hospitalier Universitaire Rouen, Rouen, France; ^7^Réanimation polyvalente, Centre Hospitalier Yves le Foll, Saint-Brieuc, France; ^8^Réanimation polyvalente, CHRU Hôpitaux de Tours, Tours, France; ^9^Réanimation médicale, hôpital central, C.H.U. de Nancy, Nancy, France; ^10^Réanimation Médicale, CHU Saint-Etienne - Hôpital Nord, Saint-Étienne, France; ^11^Réanimation médicale, C.H.U. Hôtel Dieu, Nantes, France; ^12^Réanimation médicale, C.H.U. Grenoble, Grenoble, France; ^13^Réanimation médicale, Centre Hospitalier Universitaire Archet, Nice, France; ^14^Réanimation médicale, Nouvel Hôpital Civil, CHU Strasbourg, Strasbourg, France; ^15^Réanimation médicale, Centre Hospitalier Universitaire de Caen, Caen, France

####### **Correspondence:** Rafael Mahieu - rafael.mahieu@gmail.com

*Annals of Intensive Care* 2017, **7(Suppl 1)**:O24

**Introduction** Despite being a fully preventable infectious disease, tetanus is still responsible for about 500,000 death worldwide. In developed countries, the incidence is strongly associated with a lack of vaccination coverage in the elderly. In the USA or in France, people over 65 year-old have a twice to tenfold increase in annual incidence of tetanus versus younger patients [1]. Considering the long lasting effects of tetanus toxin up to 6 weeks, elderly people admitted in intensive care units (ICU) are of particular risk of complications. Prognostics factors are well known in developing countries with a mortality rate above 20% but clinical data in developed countries are missing. We conducted a multi-center retrospective study in France on 90-day and 1-year mortality in patients with tetanus admitted in ICU.

**Patients and methods** This study was conducted over 15 French ICUs. All adults patients admitted for tetanus from January 2000 to December 2014 were included. Data were retrospectively collected from medical files. Long-term vital outcome was obtained by interrogating town hall registries.

**Results** Seventy patients were recruited over the study period. Median age was 80 years [IQ range 73–84], 86% were women. Median Charlson comorbidity index score was 4 [3–5] and Knaus chronic health score status was distributed equally between category A or B (50% each). All patients had trismus and 56% had generalized form at presentation. The median incubation period was 10 days [IQR 8–14]. Mechanical ventilation (MV) was performed in 90% of patients for a median duration of 36 days [IQR 26–46]. Median SAPS II score at ICU admission was 33 [IQR 26–40], corresponding to an predicted hospital mortality rate of 14%. Median length of stay in ICU was 41 days [IQR 24–53]. Ninety-day and one-year mortality rates were 13% (n = 9) and 16% (n = 11) respectively. Kaplan–Meier survival curve is presented Fig. 4.

**Fig. 4:**
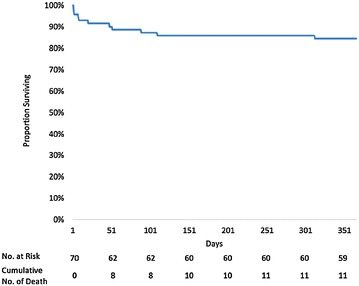
Overall survival of tetanus patients admitted in ICU

Death typically occurred within the first week (55%) due of severe arrhythmia at admission or during the fourth ICU week (withdrawal of life sustaining therapies in 3 patients or multiple organ dysfunction syndrome due to nosocomial infections in 2). Ventilator-associated pneumoniae incidence was 15 episodes per 1000 ventilation-days (total of 2234 ventilator-days observed) corresponding to 48% of ICU patients receiving MV.

Mortality was associated with older age (83 [81–85] versus 79 [73–84] years, p = 0.06) and baclofen use (intrathecal or intravenous, 4/9 non survivors vs. 8/61 in survivors, p = 0.04). Shorter incubation period (under the median delay of 10 days), generalized tetanus, wound debridement (performed in 11 patients) and higher SAPSII (above 30) were significantly associated with longer duration of MV.

**Discussion** In-hospital mortality rate was low and consistent with SAPS II estimation [2], despite long-term mechanical ventilation in an elderly population. Surprisingly, baclofen use was associated with an increased risk of death and may be linked to drug-related adverse events. Association of wound debridement with MV duration may either be related to higher bacterial inoculum in larger wound or to per-procedure toxin release.

**Conclusion** Long-term follow-up for tetanus-related ICU admission highlights the favorable outcome of this elderly population despite very prolonged MV and frequent infectious complications.

**Competing interests** None.

**References**

1. Centers for Disease Control and Prevention (CDC). Tetanus surveillance—United States, 2001–2008. MMWR Morb Mortal Wkly Rep. 2011;60(12):365–9.
2. Le Gall JR, Lemeshow S, Saulnier F. A new Simplified Acute Physiology Score (SAPS II) based on a European/North American multicenter study. JAMA. 1993;270(24):2957–63.

#### O25 The clinical spectrum of purpura fulminans in adult patients: a national multicenter retrospective study of 306 patients

##### Damien Contou^1^, Romain Sonneville^2^, Gwenhaël Colin^3^, Remi Coudroy^4^, Frédéric Pène^5^, Jean-Marc Tadié^6^, Martin Cour^7^, Gaetan Beduneau^8^, Antoine Marchalot^9^, Laurent Guérin^10^, Sebastien Jochmans^11^, Stephan Ehrmann^12^, Nicolas Terzi^13^, Sebastien Preau^14^, François Barbier^15^, Christian Brun-Buisson^1^, Armand Mekontso Dessap^1^, Nicolas de Prost^1^, for the Purpura Fulminans Study Group

###### ^1^Réanimation Médicale, Hôpital Henri Mondor, Créteil, France; ^2^Service de réanimation médicale et infectieuse, Hôpital Bichat-Claude Bernard-APHP, Paris, France; ^3^Réanimation polyvalente, Centre Hospitalier Départemental - site de La Roche-sur-Yon, La Roche-sur-Yon, France; ^4^Réanimation médicale, CHU de Poitiers, Poitiers, France; ^5^Réanimation Médicale, Hôpital Cochin, Paris, France; ^6^Réanimation médicale, Centre Hospitalier Universitaire de Rennes, Rennes, France; ^7^Réanimation Médicale, Hospices Civils de Lyon - Groupement Hospitalier Edouard Herriot, Lyon, France; ^8^Réanimation médicale, Centre Hospitalier Universitaire Rouen, Rouen, France; ^9^Service de réanimation médicale, Centre Hospitalier de Dieppe, Dieppe, France; ^10^Réanimation médicale, Hôpital Ambroise Paré (AP-HP), Boulogne-Billancourt, France; ^11^Service de Réanimation, Centre Hospitalier Marc Jacquet, Melun, France; ^12^Réanimation polyvalente, CHRU Hôpitaux de Tours, Tours, France; ^13^Service de réanimation médicale, Clinique de Réanimation Médicale, Grenoble, France; ^14^Réanimation médicale, Centre Hospitalier Régional Universitaire de Lille, Lille, France; ^15^Loiret, Hôpital Régional Orléans La Source, Orléans, France

####### **Correspondence:** Damien Contou - damien.contou@aphp.fr

*Annals of Intensive Care* 2017, **7(Suppl 1)**:O25

**Introduction** The available data on *Purpura Fulminans* in adult patients are scarce, old and mainly limited to patients with meningococcal infections. Our aims were:

1. to describe the clinical features.
2. to identify predictive factors of in-ICU mortality.
3. to compare the presentation and outcomes between causative micro-organisms.
4. to report the rate of limb amputation and identify predictive factors for limb amputation in adult patients admitted in intensive care unit for an infectious *Purpura Fulminans*.

**Patients and methods** We performed a 16-year national multicenter retrospective study in 56 ICUs in France from 2000 to 2016. Infectious PF was defined by the association of a sudden and extensive purpura, whatever its causative microorganism, together with the need for vasopressor support. Patients with a noninfectious purpura or with a purpura in a context of infectious endocarditis were excluded from the study.

**Results** (1) *Clinical features upon ICU admission* A total of 306 patients were included in the study for an incidence of 0.35 patients per year and per center. Patients were young (median age 34 years [21–53]) and had no previously known comorbidity in 69% of cases. Symptoms before ICU admission included fever (77%), digestive symptoms (61%), headache (45%), myalgia (25%) and lower limb pain (21%), for which 16% of the patients consumed non-steroidal anti-inflammatory drugs before admission. Before ICU admission, 77% of patients had a purpura notified and 75% received a parenteral β-lactam antibiotic. A successful resuscitation of an out-of-hospital cardiac arrest was recorded for 5% of patients. Mean coma Glasgow score at ICU admission was 15 [13–15] and 20% of patients had a neck stiffness. A lumbar puncture was performed in 56% of patients and showed a meningitis (pleocytosis > 10/mm^3^) in 45% of them. A bacteremia was documented in 66% of cases (n = 202/306) and a positive cerebrospinal fluid culture was obtained for 51% of lumbar punctures performed (n = 85/171). In all, the two predominantly identified microorganisms were *Neisseria meningitidis* (n = 195/306, 63%), mainly serogroup B (39%) and C (34%), and *Streptococcus pneumoniae* (n = 67/306, 22%).

(2) *Patients’ outcomes* In-ICU mortality was 41% (n = 126/306). Compared to ICU non-survivors, ICU survivors were younger (29 vs. 43 years, p < 0.0001), had more frequently received a β-lactam antibiotic before ICU admission (83 vs. 64%, p < 0.0001), had more frequent neck stiffness (26 vs. 12%, p = 0.004) and cytological meningitis (51 vs. 31%, p = 0.029) and were more frequently documented with *Neisseria meningitidis* (69 vs. 56%, p = 0.04). Age (OR 1.02/year, 95% CI [1.01–1.04]; p = 0.002) and SOFA score (OR 1.44/point, 95% CI [1.30–1.58]; p < 0.0001) upon ICU admission were the only variables independently associated with ICU mortality.

(3) *Impact of the identified microorganism* As compared with others, patients with pneumococcal PF were older (p < 0.0001), had more frequent asplenia (48 vs. 2%, p < 0.0001), higher SAPS 2 and SOFA scores together with a higher ICU mortality (52 vs. 36%, p = 0.04), and required more frequent limb amputation (31 vs. 9%, p < 0.0001).

(4) *Surviving patients requiring amputations* 22% of ICU survivors (n = 39/180) eventually required amputation during their ICU stay with a median of 3 [2–4] limbs amputated. Among ICU survivors (n = 180/306, 59%), those who were amputated (n = 39/180, 22%) were older (42 vs. 26 years, p = 0.016) and presented with higher SOFA and SAPS 2 scores, lower platelets counts (38 vs. 87.10^3^ mm^−3^, p < 0.0001), more severe kidney injury and higher arterial lactate (7 vs. 5 mmol/L, p < 0.001) and creatine kinase (856 vs. 159 IU/L, p < 0.001) levels than those who were not amputated. By multivariable regression analysis, the following risk factors for limb amputation were identified among ICU survivors: SOFA score upon ICU admission (OR 1.34/point, 95% CI [1.14–1.59]; p < 0.0001), *Streptococcus pneumoniae* PF (OR 5.05, 95% CI [1.73–14.74]; p = 0.003), and platelets transfusion (OR 6.06, 95% CI [2.20–16.73]; p < 0.0001).

**Conclusion** *Purpura Fulminans* is a rare disease mainly affecting young healthy patients. Most patients had an extensive purpura identified before ICU admission and those receiving antibiotics before ICU admission had a lower mortality than others. *Neisseria meningitidis* and *Streptococcus pneumoniae* were the main micro-organisms identified. The overall ICU mortality was high and limb amputations were needed in almost one quarter of ICU survivors. Patients with *Streptococcus pneumonia* PF had a poorer outcome with a higher ICU mortality and a higher risk of limb amputation.

**Competing interests** None.

**References**

1. Vincent J-L, Nadel S, Kutsogiannis DJ, Gibney RTN, Yan SB, Wyss VL, et al. Drotrecogin alfa (activated) in patients with severe sepsis presenting with purpura fulminans, meningitis, or meningococcal disease: a retrospective analysis of patients enrolled in recent clinical studies. Crit Care Lond Engl. 2005;9(4):R331–43.
2. Giraud T, Dhainaut J-F, Schremmer B, Regnier B, Desjars P, Loirat P, et al. Adult overwhelming meningococcal purpura: a study of 35 cases, 1977–1989. Arch Intern Med. 1991;151(2):310–6.

#### O26 Cerebral NIRS profiles during premedication for neonatal intubation

##### Meryl Vedrenne-Cloquet^1^, Sophie Breinig^2^, Camille Jung^3^, Maxime Brussieux^3^, Marie-Odile Marcoux^2^, Xavier Durrmeyer^1^

###### ^1^Service de réanimation néonatale, C.H. Intercommunal Créteil, Créteil, France; ^2^Service de réanimation néonatale et pédiatrique, Hospital Center University Toulouse - Casselardit Ancely, Toulouse, France; ^3^Centre de recherche clinique, C.H. Intercommunal Créteil, Créteil, France

####### **Correspondence:** Meryl Vedrenne-Cloquet - meryl_vedrenne@yahoo.fr

*Annals of Intensive Care* 2017, **7(Suppl 1)**:O26

**Introduction** To date, there is no consensus on which anesthetic protocol should be used before neonatal intubation. As a result, awake intubation, although strongly discouraged by current recommendations, is still common in neonates, especially in France. Many experts consider Propofol, a short-acting anesthetic agent, an appropriate drug for premedication before neonatal intubation. Propofol is known to decrease systemic vascular resistance leading to low arterial blood pressure, which consequently raises concerns about its hemodynamic tolerance. However in neonates, low mean arterial blood pressure (MABP) is poorly correlated with low systemic or cerebral blood flow. NIRS (Near InfraRed Spectroscopy) allows cerebral tissue oxygenation monitoring, reflecting cerebral blood flow.

The aim of our study was to compare cerebral oxygenation profiles between a combination of a synthetic opioid plus a muscle-blocker and propofol, used as premedication prior to neonatal endotracheal intubation.

**Patients and methods** Observational prospective study, conducted in 2 of the 8 centers participating in a randomized, controlled, double-blind, multicenter trial (PRETTINEO study, ClinicalTrial.gov identifier NCT01490580). Patients were randomly assigned (1:1) between “atropine-propofol” and “atropine-atracurium-sufentanil” before elective or semi-urgent intubation in the neonatal intensive care unit. Randomization was stratified on weight and center. Exclusion criteria included low blood pressure defined as a MABP (in mmHG) below gestational age (in weeks). Physiological parameters, including pulse oxymetry (SpO_2_) and regional cerebral oxygen saturation (rScO_2_), were collected 1 min prior to induction of anesthesia, and then up to 60 min after. To investigate the balance between oxygen delivery and consumption, cerebral fractional tissue oxygen extraction (FTOE) was calculated as FTOE = (SpO_2_ − rScO_2_)/SpO_2_. The analyzed parameters included changes in rScO_2_ and FTOE over time and their relative change from baseline value in both treatment groups.

**Results** From March to August, 2016, 65 neonates were assessed for eligibility. Among them, 28 were finally included in this ancillary study. Their mean (SD) gestational age at birth was 32 (5) weeks. Median [IQR] age and mean (SD) weight at the time of intubation were 0.5 [0.2–5.4] days and 1886 (900) g respectively. At the time this abstract was conceived, data validation had not been completed. Results will be available at the time of the meeting.

**Discussion** To our knowledge, only two studies to date have investigated the cerebral hemodynamic effects of propofol in neonates. Both reported persistent low MABP after a propofol bolus but a very short decrease in rScO_2_. Only one studied propofol before intubation and suggested that low rScO_2_ could be attributable to low SpO_2_ during the procedure, but not to low MABP (Smits et al., J Pediatr, 2016). None of them compared propofol to another anesthetic protocol. Our study is thus the first to compare cerebral oxygenation between two currently acceptable regimens of anesthesia for premedication before neonatal intubation.

**Conclusion** This observational study is expected to provide useful information about cerebral oxygenation in neonates during intubation and to disentangle the effects of the drugs from the effects of the procedure. We also expect this study will en courage Neonatologists to avoid awake intubation.

**Competing interests** None.

**References**

1. Vanderhaegen J, Naulaers G, Van Huffel S, Vanhole C, Allegaert K. Cerebral and systemic hemodynamic effects of intravenous bolus administration of propofol in neonates. Neonatology. 2010;98(1):57–63.
2. Smits A, Thewissen L, Caicedo A, Naulaers G, Allegaert K. Propofol dose-finding to reach optimal effect for (semi-)elective intubation in neonates. J Pediatr. 2016; pii: S0022-3476(16)30651-5. doi:10.1016/j.jpeds.2016.07.049 **(Epub ahead of print)**.

#### O27 A prospective multicentric study of severe cutaneous infections in pediatric intensive care: the SCIPIC cohort

##### Renaud Blondé^1^, François Angoulvant^2^, Jérôme Grasset^3^, Jérôme Naudin^4^, Stéphane Dauger^4^, GFRUP, RMEF

###### ^1^Mayotte, CHM, Mamoudzou, France; ^2^Urgences pédiatriques, CHU Necker-Enfants Malades, Paris, France, France; ^3^Iriseo, Iriseo - 3D Supports multimédia Interactifs, Saint-Victurnien, France; ^4^Réanimation et surveillance continue pédiatriques, CHU Robert Debré, Paris, France

####### **Correspondence:** Stéphane Dauger - stephane.dauger@aphp.fr

*Annals of Intensive Care* 2017, **7(Suppl 1)**:O27

**Introduction** Cellulitis (CEL) and Necrotizing Fasciitis (NF) are life-threatening skin infections much more frequent in adults than in children. Therefore, despite major differences in epidemiology, microbiology, and outcome, clinical guidelines for management are directly adapted from adult ICU experience. We designed a prospective multicentric cohort study to describe the clinical course of CEL/NF requiring admission to PICU.

**Patients and methods** This study was approved by the IRB Paris Nord and the French Data Protection Authority (CCTIRS/CNIL). After parents’ consent was obtained, thirty centers (France, French Polynesia, Mayotte and La Réunion Islands, French Caraibs Islands, Switzerland, Belgium, Canada, Netherlands, Norway) included on a secure-dedicated-website all patients aged from 28 days to 18 years and admitted to PICU for a suspected or confirmed CEL/NF with association of the three following signs: (i) area of skin inflammation, (ii) rapid evolution or skin necrosis, (iii) variation in core temperature (>38.5 °C or <36 °C). Premature infants (<37 weeks of gestation) and patients with Stevens Johnson syndrome, Purpura Fulminans or Hereditary Angioedema were excluded. We collected all data concerning past medical history, first clinical signs, PICU stay, antibiotherapy, microbiology (Staphylococcus and Streptococcus species were send to French Reference Centers), radiological diagnosis, surgery and outcome 1 year after discharge. Data are presented as mean ± SD according to their Gaussian distribution.

**Results** 50 patients (age: 5.9 ± 5.2 months and weight: 24.2 ± 18.1 kg) were included from October 2011 to April 2016. The diagnosis was suspected 3.5 ± 2.7 days after a known risk factor (46%; surgery: 12; NSAIDs: 10; varicella: 9; cancer: 4). Only two patients had travelled abroad. Skin lesions observed on 10.6 ± 16% of body surface were erythema (28), necrosis (13) and bulla (10) and were located on face (17), legs/feet (16), abdomen/pelvis (14), arms/hands (13) and trunk (10). On admission, mortality and organ dysfunction scores were as follow: PRISM = 9.3 ± 7.5; PIM2 = 12.6 ± 23.3; PELOD D1 = 12.6 ± 3.2 and POPC = 1.6 ± 1.0. Main biological tests showed: hemoglobin: 10.0 ± 2.2 g/dl; neutrophils: 10320 ± 8740/mm^3^; platelets: 197700 ± 140764 mm^3^; CRP: 189 ± 122 mg/l; PCT: 69.3 ± 75.3 mg/l; fibrinogen: 5.2 ± 2.2 g/l; ASAT: 108 ± 166 UI/l and ALAT: 61 ± 82 UI/l; protidemia: 53 ± 13 g/l. 28 patients (56%) showed low blood pressure for age including 14 with oliguria, needing fluid challenges (45 ± 26 ml/kg) and vasopressors for 3 ± 1 days. 29 patients (68%) were ventilated invasively for 8.2 ± 10.5 days, with PEEP = 7±3 cmH_2_O and FIO_2_ = 58 ± 27% during the first 3 days. 80% of patients received continuous intravenous analgesia (morphine and benzodiazepines). Half of patients were transfused with blood products and also a half received albumin. While MRI was performed in only 7 cases, CT scan and ultrasound were performed to confirm diagnosis in 23 cases (46%, 14 on day 1) and in 21 cases (42%, 15 on day 1), respectively. 28 patients (56%) were operated. One bacterial strain alone was identified in 36 cases (72%) and at least two in eight cases (16%), including *Staphylococcus aureus* (17), *GAβH*–*S* (13), *Escherichia coli* (5), *Pseudomonas aeruginosa* (4). Antibiotics used were penicillin G (26), clindamycin (21), 3rd generation cephalosporin (12) and rifampicin (9). A third of patients received immunoglobulins and five children (10%) received hyperbaric oxygen (22 sessions). Length of stay in PICU was 12.5 ± 14.7 days. Three patients (6%) died and only two (4%) were readmitted to PICU because of failure directly link to skin lesion. POPC score on discharge was 1.9 ± 1.3. Long term follow-up at 1 year is ongoing.

**Discussion** This study reports the largest prospective multicentric international cohort of pediatric skin infections needing PICU admission to date. Surgery was the first risk factor reported in our cohort, before varicella. Severe circulatory and respiratory failures on admission in a context of deep biological inflammatory syndrome required aggressive treatments during the first 3 days. Lower part of the body was more frequently involved than reported before in childhood. The diagnosis was quickly suspected and confirmed mainly by ultrasound and CT scan, with the use of MRI still very rare. More than a half of patients have been operated after classical treatments associating immunoglobulins and antitoxin-antibiotics. Infection appeared mainly monobacterial as usually described in children but gram-negative strains are emerging. Mortality was still low and sequella were rare.

**Conclusion** Cellulitis (CEL) and Necrotizing Fasciitis (NF) are still rare in childhood, with low mortality and few sequella if aggressive treatment including surgery is emergently performed. However, it seems that epidemiology has changed during the last decade in high-income countries. Statistical analysis of this database is ongoing to identify predictors of surgical requirements and to describe more precisely bacterial strains involved.

**Competing interests** None.

**References**

1. de Prost N, Sbidian E, Chosidow O, Brun-Buisson C, Amathieu R; Henri Mondor Hospital Necrotizing Fasciitis Group. Management of necrotizing soft tissue infections in the intensive care unit: results of an international survey. Intensive Care Med. 2015;41(8):1506–8.
2. Endorf FW, Garrison MM, Klein MB, Richardson A, Rivara FP. Characteristics, therapies, and outcome of children with necrotizing soft tissue infections. Pediatr Infect Dis J. 2012;31(3):221–3.

#### O28 Immunosuppression induced by septic shock in children: a prospective observational study before a multicenter therapeutic trial

##### Solenn Remy^1^, Karine Kolev-Descamp^1^, Julie Demaret^2^, Guillaume Monneret^2^, Etienne Javouhey^1^

###### ^1^Réanimation pédiatrique, Hôpital Femme Mère Enfant, Bron/Lyon, France; ^2^Laboratoire d’immunologie cellulaire, Hospices Civils de Lyon - Groupement Hospitalier Edouard Herriot, Lyon, France

####### **Correspondence:** Solenn Remy - solenn.remy@hotmail.fr

*Annals of Intensive Care* 2017, **7(Suppl 1)**:O28

**Introduction** Immunosuppression induced by sepsis is well described in adults. Therapeutic trials with immunomodulatory treatments are already underway, using HLA-DR expression on monocytes (mHLA-DR) or lymphopenia as biomarkers of immunosuppression. Pediatric patients with Septic Shock (SS) have been much less studied. Thus, the main objective of this study was to explore post-sepsis immunoparalysis in a pediatric cohort study. Both sides of cellular immunity were assessed: innate immunity (mHLA-DR) and adaptive immunity (lymphocyte subsets). We also wanted to obtain normal values of mHLA-DR in healthy children, according to age.

**Materials and methods** We performed a single-center prospective study with children under 18 years-old, admitted in Pediatric Intensive Care Unit for SS (“Surviving Sepsis Campaign” criteria), between September 2014 and July 2016. We recruited controls from healthy children hospitalized for an elective surgery, without any criteria of infection. mHLA-DR, total lymphocyte count, and lymphocyte sub-populations’ proportions (CD4^+^ and CD8^+^ T cells, regulatory T cells, NK cells, and B cells) were determined by flow cytometry. Samples were analyzed at Day 1, 3 and 7, after sepsis onset. Clinical data were collected prospectively, especially severity scores [PIM2, PELOD2 and Cumulative Vasopressor Index (CVI)], and secondary nosocomial infection occurrence.

**Results** 30 controls and 26 patients were recruited. mHLA-DR in healthy children presented no variation according to age, and was similar to healthy adults. At each time points, mHLA-DR in SS group was decreased, comparing with controls. The medians of mHLA-DR were respectively 6.066 IQR [3.737–16.310], 6.308 IQR [3.185–8.965] and 9.323 IQR [6.384–12.738], versus 29.668 ab/c IQR [24.335–39.199] in the control group (*p* < 0.0001 at D1, D3 and D7; Mann–Whitney). mHLA-DR at D3 was significantly correlated with the Cumulative Vasopressor Index (Spearman’s correlation coefficient r = −0.50; *p* = 0.031). Patients secondarily infected presented a lower mHLA-DR at D3 than patients without secondary infection: respectively 4398 ab/c [2437–6212] versus 8474 ab/c [5904–10844] (*p* = 0.022, Student t test) (Fig. 5).

**Fig. 5:**
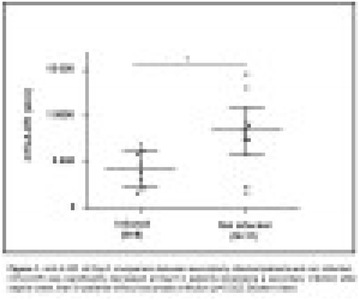
See text for description

Total lymphocytes and CD4 T cells were decreased at D1 and D3. This lymphopenia corrected between D3 and D7. NK cells were decreased at each time points. B lymphocytes were decreased comparing to controls at D1 and D7 (not D3), but much less pronounced than T cells. Regulatory T cells percentage was initially comparable to controls. Then, a gradual increase was observed, which became statistically significant at D7, compared to controls (*p* = 0.0058; Mann–Whitney). No significant difference was observed between patients with or without secondary infections, according to any lymphocyte subsets.

Concerning clinical data, patients contracting a secondary infection stayed significantly longer at the hospital than others: respectively 33.5 days [19.5–58.0] versus 14.0 [7.5–21.0] (*p* = 0.0094; Mann–Whitney).

**Conclusion** For the first time, we reported that healthy children presented mHLA-DR values with no variation according to age, and similar to those seen in adults. Innate immunity was similarly altered in children with Septic Shock than in adult patients. Adaptive immunity seemed to be less impaired by the shock than in adults. Further analyses will be conducted to complete these findings as functional tests on T cells. As secondary infections considerably increase the length of stay at the hospital, this report paves the way for a multicenter therapeutic trial, in order to evaluate immunostimulatory treatment in children with Septic Shock.

**Competing interests** None.

**References**

1. Hotchkiss RS, Monneret G, Payen D. Sepsis-induced immunosuppression: from cellular dysfunctions to immunotherapy. Nat Rev Immunol. 2013;862–74.
2. Hall MW, Knatz NL, Vetterly C, Tomarello S, Wewers MD, Volk HD, Carcillo JA. Immunoparalysis and nosocomial infection in children with multiple organ dysfunction syndrome. Intensive Care Med. 2011;37:525–32.

#### O29 Ventilator associated pneumonia and ventilator associated events in pediatric intensive care

##### Maryline Chomton^1^, David Brossier^1^, Michaël Sauthier^1^, Emilie Vallieres^2^, Philippe Jouvet^1^

###### ^1^Soins intensifs pédiatriques, CHU Sainte-Justine, Montréal, Canada; ^2^Microbiology department, CHU Sainte-Justine, Montréal, Canada

####### **Correspondence:** Maryline Chomton - chomton.m@gmail.com

*Annals of Intensive Care* 2017, **7(Suppl 1)**:O29

**Introduction** Ventilator associated pneumonia (VAP) is the second cause of nosocomial infection in pediatric intensive care. Recent modifications of the CDC criteria for VAP diagnosis included VAP diagnostic criteria for children [1] and criteria to identify ventilator associated events (VAE) in adults [2] that are not validated for children. The purposes of this study were to determine retrospectively the incidence, risk factors and management of VAP using the new CDC definition and to study the validity of adult VAE diagnostic criteria in critically ill children.

**Patients and methods** We conducted a single center retrospective study in the pediatric intensive care unit (PICU) of Sainte-Justine Hospital, a medical and surgical PICU including cardiac surgery patients. All patients invasively mechanically ventilated (IMV) >48 h between November 2013 and November 2015 were included. Patient records were analyzed for VAP diagnosis, risk factors, management and VAE. Statistical analysis included median and range calculation for descriptive data and children with and without VAP were compared using a Mann–Whitney test with a significant level: p < 0.05.

**Results** Of 688 invasively ventilated patients, 287 were included in our study (IMV > 48 h). Thirty patients met radiologic and clinical VAP CDC criteria. VAP rate was 10.4% and VAP incidence was 7/1000 invasive ventilation days. Mortality rate was higher in the VAP group but in the VAP group was not statistically significant [16.0 vs 9.7% respectively, (p = 0.23)]. VAP occurred early in IMV course (4 days). The main technique to identify the pathogen was tracheal aspiration with semi quantitative culture, the most common VAP organisms identified were Gram Negative bacteria (60%) with predominance of *Haemophilus influenza and Pseudomonas aeruginosa* with sensitive profile and antibiotherapy used was in accordance with guidelines. Median duration of ventilation (15 vs 6 days, p < 0.001) and PICU stay (19 vs 9 days, p < 0.001) increased significantly in children with VAP versus no VAP. Univariate analysis showed that age, reintubation, ARDS and continuous enteral feeding were risk factors for VAP. Among the thirty patients with VAP, 17 had adult CDC VAE’s criteria (sensitivity = 56%).

**Discussion** The following points are in agreement with VAP literature. Using VAE criteria could constitute a faster, more objective method to screen VAP. However, sensitivity should be improved by adapting VAE criteria to children.

**Conclusion** Using the CDC updated VAP definition for children, VAP incidence is similar to adults. Adult VAE cannot be used to screen prospectively VAP in children. Specific pediatric VAE needs to be developed and validated.

**Competing interests** None.

**References**

1. CDC. Pneumonia (ventilator associated (VAP) and non ventilator pneumonia (PNEU)) Events;2015.
2. CDC. Ventilator associated events;2015.

#### O30 Incidence and risk factors of ventilator associated pneumonia in neonatal intensive care unit

##### Guillaume Geslain^1^, Isabelle Guellec^2^, Jérôme Rambaud^2^

###### ^1^Hôpital Robert-Debré (AP-HP), Paris, France; ^2^Réanimation pédiatrique et néonatale, Hopital pour enfants Trousseau, Paris, France

####### **Correspondence:** Guillaume Geslain - guillaumegeslain@hotmail.fr

*Annals of Intensive Care* 2017, **7(Suppl 1)**:O30.

**Introduction** Ventilator associated pneumonia (VAP) is defined as a lung infection occurring after 48 h of mechanical ventilation. Ventilator associated pneumonia incidence, complications and mortality are well known in critically ill adults but probably under diagnosed in critically ill neonates. The main objective of our study was to evaluate the incidence of VAP in neonatal intensive care units, evaluate associated mortality and morbidity and find potential risk factors.

**Patients and methods** This is a prospective, observational, single-center conducted in neonatal intensive care unit of Armand Trousseau hospital from 01/11/2014 to 31/10/15. All infants aged 28 days or less hospitalized in the service are included.

**Results** 381 patients were enrolled including 327 intubated patients. 17 of 327 patients intubated presented VAP. The incidence of VAP was 4.05 per 1000 days of hospitalization with an incidence rate of 8.78 per 1 000 days of invasive ventilation. The average age at diagnosis of VAP was 21.47 ± 13.02 days for an average duration of invasive ventilation of 15.23 ± 11.48 days. After VAP, invasive ventilation time is prolonged to 25.71 ± 20.90 days (OR 1.19 [1.12–1.27]). The nosocomial infection rate was significantly higher in the VAP group (p < 0.001) with 9 on 17 patients in VAP group (52.94%) versus 21 on 310 patients (6.77%). The occurrence of VAP was significantly associated with higher mortality (OR 4.46 [1.32–14.94]) and an increase in invasive ventilation times (p < 0.001) and non-invasive (p < 0.001) and hospital stay (p < 0.001). There is a significant difference in the duration of invasive ventilation before VAP [average 15.23 ± 11.48 (1–35)] compared to patients without VAP [mean 4.84 ± 4.92 (1–28), p < 0.001]. Patients with a birth weight less than or equal to 1000 grams are associated with risk of VAP (OR 4.31 [1.38–13.39]) in multivariate analysis contrary to the term. Intubated patients with a balloon are associated with risk of VAP (adjusted OR 4.03 [1.14–14.26]) and a Snappe-II score above 16 also (OR 4.98 [1.40–17.67]).

**Conclusion** We managed one of the larger neonatal study for VAP in critically ill neonates. These pneumonias remained frequent in critically ill neonate and are associated with a higher mortality and morbidity. Patients with a birth weight less than 1000 g seem to be particularly vulnerable.

**Competing interests** None.

#### O31 Extracorporeal life support for acute respiratory failure in immunocompromised patients: an international multicenter retrospective study (The IDEA study)

##### Matthieu Schmidt^1^, Peter Schellongowski^2^, Amandine Dorget^1^, Nicolo Patroniti^3^, Fabio Silvio Taccone^4^, Dinis Reis Miranda^5^, Jean Reuter^6^, Hélène Prodanovic^7^, Romain Sonneville^6^, Marc Pierrot^8^, Martin Balik^9^, Sunghoon Park^10^, Alain Combes^11^, REVA, ECMOnet

###### ^1^Service de réanimation médicale, Groupe Hospitalier Pitié-Salpêtrière, Paris, France; ^2^Unit 13i2, Department of Medicine I/intensive care, Vienna, Austria; ^3^Anestesia e raiamizione, Ospedale San Gerardo, Monza, Italy; ^4^Service de Soins Intensifs, Hôpital Erasme, Bruxelles, Belgium; ^5^Intensive care, Erasmus University Medical Center, Rotterdam, Netherlands; ^6^Service de réanimation médicale et infectieuse, Hôpital Bichat-Claude Bernard-APHP, Paris, France; ^7^Unité de réanimation et de surveillance continue, service de pneumologie et réanimation médicale, Pitié-Salpêtrière Hospital, Paris, France; ^8^Service de réanimation medicale, Hopital Universitaire d’Angers, Angers, France; ^9^Dept of anaesthesia and intensive care, General University Hospital in Prague, Prague, Czech Republic; ^10^Pulmonary, Allergy and Critical Care, Hallym University Sacred Heart Hospital, Seoul, Republic of Korea; ^11^Service de Réanimation Médicale, Groupe Hospitalier Pitié Salpêtrière, Paris, France

####### **Correspondence:** Matthieu Schmidt - matthieuschmidt@yahoo.fr

*Annals of Intensive Care* 2017, **7(Suppl 1)**:O31

**Introduction** The proportion of immunocompromised patients with extracorporeal life support (ECLS)-treated severe ARDS varies from 5 to 31% in recent cohorts. To date, very few data on ECMO use and its associated outcomes are available on this population. Our aims were to (1) describe the clinical features, (2) compare the outcomes between causative immunocompromised status, (3) identify predictive pre-ECLS factors of 6-month mortality, and (4) report the rate of ECLS-related complications.

**Patients and methods** We performed an international multicenter retrospective study in 10 ICUs from 2008 to 2014. Immunocompromised status was defined by hematologic malignancies, solid tumor, solid organ transplant, human immunodeficiency virus (HIV), or long term or high dose glucosteroids or immunosuppressant use. Inclusion criteria were immunocompromised patient with acute respiratory failure rescued by extracorporeal membrane oxygenation or (ECMO) or extracorporeal CO_2_ removal (ECCO2R).

**Results** 1. A total of 225 patients (age 48.6 ± 15.1 years; APACHE II 26.4 ± 9.0) were included in the study (30% hematologic malignancies, 28% long-term corticosteroids or immunosuppressant use, 18% solid tumor, 16% solid organ transplant, and 9% HIV). ECLS was initiated for severe ARDS, moderate ARDS, or chronic end-stage respiratory in 190(84%), 14(6%), and 19(8%) patients, respectively. Main ARDS etiologies were bacterial pneumonia (30%), viral pneumonia (18%), and specific lung involvement (12%). Refractory hypoxemia (73%) was the main indication for ECLS with lowest pre-ECLS PaO_2_/FiO_2_ at 63 (51–87) mmHg. Venovenous ECMO and ECCO2R were initiated in 199 (88%) and 15(7%) patients, respectively. Pre-ECLS tidal volume was 5.7(4.7–6.4) mL/kg, with a positive end-expiratory pressure at 10(8–13) cmH_2_O and a plateau pressure at 32(30–35) cmH_2_O. Interval between mechanical ventilation onset and cannulation was 2(1–8) days.

2. Six-month mortality of patients with hematologic malignancies, long-term corticosteroids or immunosuppressant use, solid tumor, solid organ transplant, and HIV were 76, 60, 80, 51, and 71%, respectively (log-rank test, p = 0.02). Cumulative survival at 6 months were lower for patients with hematologic malignancies versus others (log-rank test, p = 0.003) whereas those with solid organ transplant exhibited higher cumulative survival at 6 months (log-rank test, p = 0.02).

3. One hundred and three patients (46%) were successfully weaned from ECLS. In-ICU and 6-month post ICU discharge survival were 37% (n = 83/225) and 32% (n = 72/225), respectively. Compared to patients who died within 6 months after ICU admission, 6-month survivors were younger (46 vs. 49 years, p = 0.05), had more frequently a newly diagnosed immunocompromised status (39 vs. 17%, p = 0.0003), were more frequently patients with solid organ transplant (24 vs 12%, p = 0.02, had a higher pre-ECMO hemoglobin (9.4 vs 8.8 g/dL) and platelet counts (160 vs 112 × 103/µL; p = 0.008), and exhibited lower mechanical ventilation-ECMO onset interval (1[0–5] vs 3[1–9] days, p = 0.002). Age (OR 1.02/year, 95% CI [1.002–1.04], p = 0.035), solid organ transplant (0.38 [0.17–0.85], p = 0.019), newly diagnosed immunocompromised status (0.32 [0.16–0.65], p = 0.002), platelet count ≥200,000 × 10^3^/µL (0.33[0.15–0.72], p = 0.005) and delay from mechanical ventilation initiation to ECMO cannulation >7 days (3.23 [1.42–7.34], p = 0.005) were independently associated with 6-month mortality.

4. Eighty-two (36%) patients had at least one ECMO-related major bleeding event (oro-nasal bleeding 10%; hemothorax 7%; cerebral bleeding 7%), which was less frequent with patients alive at 6-months. One hundred and four (46%) and 20 (9%) patients reported ventilator associated pneumonia and cannula infection, respectively, with no impact on 6-month survival.

**Conclusion** Six-month survival of ECLS-treated severe ARDS in immunocompromised patients appears low, especially for patients with hematologic malignancies and solid tumor. However, young age, solid organ transplant, newly diagnosed immunocompromised status and rapid decision to start ECLS seem associated with a better prognosis.

**Competing interests** None.

#### O32 Driving pressure is a significant predictor of mortality in the acurasys and proseva randomized controlled trials in ARDS patients

##### Claude Guérin^1^, Laurent Papazian^2^, Reignier Jean^3^, Louis Ayzac^4^, Anderson Loundou^5^, Jean-Marie Forel^6^

###### ^1^Réanimation médicale, Hôpital de la Croix-Rousse, Lyon, France; ^2^Service de réanimation-détresses respiratoires et infections sévères, Hôpital Nord, Marseille, France; ^3^Réanimation médicale, CHU Hôtel-Dieu Nantes, Nantes, France; ^4^C-clin, Hôpital Henry Gabrielle, Saint-Genis-Laval, France; ^5^Unité de recherche de santé publique, Faculté de Médecine secteur Timone (Aix-Marseille Université), Marseille, France; ^6^Réanimation médicale, Hospital Nord, Marseille, France

####### **Correspondence:** Claude Guérin - claude.guerin@chu-lyon.fr

*Annals of Intensive Care* 2017, **7(Suppl 1)**:O32

**Introduction** Driving pressure (ΔP) across the respiratory system has been suggested as the strongest predictor of hospital mortality in ARDS patients. We wonder whether this result may be due to the wide range of tidal volume (VT) and PEEP used across the trials included and whether a strict control of them would minimize the role of ΔP as predictor. Therefore, we investigated ΔP in two trials in which lung protective mechanical ventilation was applied to ARDS patients. Our working hypothesis was that ΔP was a risk factor just like compliance (Crs) or Plateau pressure (Pplat) of the respiratory system.

**Patients and methods** ARDS patients included in the Acurasys and Proseva trials previously reported were used. Both had near inclusion criteria (notably PaO_2_/FIO_2_ < 150 mm Hg and PEEP ≥ 5 cm H_2_O) and similar lung protective mechanical ventilation (in particular VT 6 ml/kg predicted body weight and PEEP/FIO_2_ table). Both found survival benefit in the experimental group. SOFA, continuous neuromuscular blocking agent (NMBA) infusion, prone position, combined use of NMBA and prone position, pH, PaCO_2_, PaO_2_/FIO_2_, lactate, breathing frequency, VT, PEEP, Pplat, Crs and ΔP were recorded at day 1 after inclusion together with gender, age and SAPSII at the time of admission and compared between survivors and nonsurvivors at day 90. Cox proportional hazard models were used with covariates significantly different between survivors and non survivors at the threshold of 0.20 and mortality at day 90 as dependent variable. Due to the obvious colinearity between ΔP, Crs and Pplat we performed the following analyses. First we made a specific Cox model for each of them. Second, we developed three Cox models in which we used the above variables by couples (Pplat-ΔP; Crs-ΔP and Crs-Pplat). We made the following assumptions: if both variables in the couple lacked significance in the second model, the same information was carried by each component of the couple; if one or both variables kept significant correlation each brought significant and distinct information; if significant correlation was kept for one of the variables in the couple and lost for the other the former would be more informative than the latter.

**Results** Both trials enrolled 805 patients of who 787 had data available at day 1 of who 533 survived and 254 did not. In the univariate analysis, ΔP averaged 13.7 ± 3.7 and 12.8 ± 3.7 cmH_2_O (P = 0.002) in nonsurvivors and survivors, respectively. In each single Cox model, Hazard ratios (HR) were 1.05 (1.02–1.08) (P = 0.005), 1.04 (1.01–1.08) and 0.985 (0.972–0.999) (P = 0.023) for ΔP, Pplat and Crs, respectively. PEEP and VT were not significant risk factors in any model. In the model with ΔP and Pplat used together, each of them kept significance [HR 1.31 (1.07–1.61) and 1.13 (1.02–1.26)]. In the model with ΔP and Crs both lost significance and the same was true in the model using Pplat and Crs.

**Discussion** ΔP was a significant predictor with a 5% increase in mortality per each cmH_2_O increment of ΔP, a result similar to that found by Amato et al. However, Pplat conveys the same information. This is likely due to the fact that VT and PEEP were similar in each group of both trials and not different between survivors and non survivors.

**Conclusion** When strict lung protective mechanical ventilation is applied to ARDS patients ΔP, Crs and Pplat were risk factors of mortality. ΔP and Pplat bring distinct and significant information.

**Competing interests** None.

#### O33 Impact of PEEP and body inclination in the supine and prone positions on esophageal pressure in ARDS patients

##### Mehdi Mezidi^1^, Mylène Aublanc^1^, Sophie Perinel-Ragey^1^, Floriane Lissonde^1^, Aurore Louf-Durier^1^, Romain Tapponnier^1^, Hodane Yonis^1^, Zakaria Riad^1^, Jean-Christophe Richard^1^, Claude Guérin^1^

###### ^1^Réanimation Médicale, Hôpital de la Croix-Rousse, Lyon, France

####### **Correspondence:** Mehdi Mezidi - mehdi.mezidi@gmail.com

*Annals of Intensive Care* 2017, **7(Suppl 1)**:O33

**Introduction** Prone positioning (PP) for long sessions in association with use of low tidal volume and cisatracurium infusion have been shown survival benefit in ARDS patients. Little is known about evaluation of PEEP setting based on end-expiratory transpulmonary pressure at zero flow (PEEPtotL) in PP. We hypothesized that esophageal pressure (Pes) was lower in PP than in supine position due the relief of the weight of the mediastinum. Hence, the PEEP level to achieve positive PEEPtotL should be lower in PP. Furthermore, the inclination of the body should also influence the measurements of Pes. The main objective of the study was to evaluate the variation of end-expiratory esophageal pressure at zero flow (PEEPtotes) in PP and in supine position, each at two body inclinations, and the subsequent effects on PEEPtotL and transpulmonary driving pressure (ΔPL).

**Materials and methods** A prospective interventional physiologic study was performed in patients with severe ARDS (PaO_2_/FIO_2_ < 150) and requiring PP. Pes was measured with an esophageal balloon. Transpulmonary pressure (Ptp) was computed as the difference between airway pressure and Pes. DPL was computed as the difference in Ptp at the end of inspiration and at the end of expiration at zero flow. Chest wall elastance (Est,cw) was calculated as the ratio of end-inspiratory Pes minus PEEPtotes divided by tidal volume. End-expiratory lung volume (EELV) was measured by nitrogen wash-out method. Thorax angulation was 30° and 0° in supine position (SP) and 0° and 15° in PP. From PEEP set according to the low PEEP/FIO_2_ table of the ARMA trial (1), PEEP level was further adjusted to achieve PEEPtotL close to 3 cmH_2_O. Measurements were done in supine position and after 1 h in PP.

**Results** Twenty patients were included. PEEPtotes did not vary significantly between SP(30°) and PP(0° or 15°). However, opposite variations were found according to thorax angulation in PP: a rise of 1.9 (SD 1.7) cmH_2_O in PP(0°), p = 0.005 and a drop in PP(15°) of 2.2 (SD 3.2) cmH_2_O, p = 0.06. In a complementary analysis, PEEPtotes was studied in four positions [SP(30°), SP(0°), PP(0°) and PP(15°)] and followed an inversed U-shape pattern with mean values of 8.6 (SD 3.2), 12.8 (SD 2.7), 10.2 (SD 3.3) and 2.6 (SD 2.1) cmH_2_O, respectively. These differences were statistically significant (Holm adjusted *p* value for multiple comparisons <0.05) except between SP(30°) and PP(15°). As a consequence, PEEPtotL rose of 2.6 cmH_2_O between SP(0°) and PP(0°). With postural variations, EELV was significantly altered. Hence, PEEPtotL at PP(0°) was computed at the EELV in SP(0°), allowing to eliminate the impact of the change of EELV between postures: 77% of PEEPtotL changes were due to change of posture per se [+3.7 (SD 2.6) cmH_2_O, p < 0.05]. Between SP(30°) and PP(0°), posture effect on PEEPtotL variation was +2.7 (SD 4.5) cmH_2_O, *p* = 0.055. A linear mixed model disclosed that rise of PEEPtotL was explained by PP for an amount of 2.7 (SD 0.44) cmH_2_O and by thorax angulation for 0.13 (SD 0.02) cmH_2_O per degree (p < 10^−6^).

PP was associated with lower ΔPL [−1.0 cmH_2_0 (SD 1.3), *P* = 0.004]. Chest wall elastance did not change between 30° supine and 0°PP [10.4 (SD 3) vs. 8.5 (SD 3.2) L/cmH_2_O, *P* = 0.85].

**Discussion** Variation of thorax angulation significantly alters Pes and Ptp values. Splitting the variation of Pes into those due to change in either EELV or posture allows a better understanding of the impact of PP on Pes.

**Conclusion** PP(0°) was associated with Pes rise as compared with SP(30°) and Pes drop when compared with SP(0°). However, when analyzing PEEPtotL variation due only to posture change (and not EELV change), a rise of almost 3 cmH_2_O of PEEPtotL was seen in PP(0°) compared to SP(0°) or SP(30°). Clinicians might therefore consider lowering PEEP level in PP.

**Competing interests** None.

**Reference**

1. The Acute Respiratory Distress Syndrome Network. Ventilation with lower tidal volumes as compared with traditional tidal volumes for acute lung injury and the acute respiratory distress syndrome. The Acute Respiratory Distress Syndrome Network. N Engl J Med. 2000;342(18):1301–8.

#### O34 Can we consider criteria for acute respiratory distress syndrome (ARDS) in patients breathing spontaneously?

##### Remi Coudroy^1^, Jean-Pierre Frat^1^, Florence Boissier^1^, Damien Contou^2^, René Robert^1^, Arnaud W Thille^1^

###### ^1^Réanimation médicale, CHU de Poitiers, Poitiers, France; ^2^Réanimation Médicale, Hôpital Henri Mondor, Créteil, France

####### **Correspondence:** Remi Coudroy - remi.coudroy@chu-poitiers.fr

*Annals of Intensive Care* 2017, **7(Suppl 1)**:O34

**Introduction** According to the Berlin definition for ARDS, the degree of hypoxemia must be assessed under either invasive or noninvasive ventilation (NIV). In a recent large international survey, about 15% of ARDS were diagnosed while treated with NIV. However, NIV is debated in hypoxemic patients with acute respiratory failure. We aimed to assess whether the use of NIV is really needed to diagnose ARDS in patients with spontaneous breathing under oxygen.

**Patients and methods** We included all ICU patients treated first with standard oxygen and then with NIV for non-hypercapnic acute respiratory failure from 2 prospective studies [1, 2]. PaO_2_/FiO_2_ was assessed at ICU admission under oxygen with an easily bedside calculated FiO_2_, and under NIV after 1 h of initiation and within the first 24 h. Severity of hypoxemia was considered as mild when PaO_2_/FiO_2_ ranged from 201 to 300, moderate from 101 to 200 and severe when ≤100 mmHg.

**Results** Among the 219 patients with acute respiratory failure treated with NIV, 172 (79%) fulfilled ARDS criteria within the first 24 h following ICU admission. ARDS was classified as mild in 14%, moderate in 53%, and severe in 33% of cases. The overall rates of intubation and ICU mortality were 58 and 30% respectively.

When considering the 155 patients with bilateral infiltrates on chest X-ray and PaO_2_/FiO_2_ ≤ 300 mmHg under oxygen, 87% (n = 135) had ARDS criteria after 1 h of NIV and 95% (n = 148) within the first 24 h. ICU-mortality according to the severity of hypoxemia did not differ under standard oxygen or NIV: 29 versus 20% in mild (p = 0.55), 22 versus 30% in moderate (p = 0.29), and 45 versus 43% in those with severe hypoxemia (p > 0.99).

**Conclusion** Almost all patients with bilateral infiltrates and PaO_2_/FiO_2_ ≤ 300 mmHg under oxygen standard meet ARDS criteria after NIV initiation. Mechanical ventilation does not seem necessary to diagnose ARDS in patients with spontaneous breathing.

**Competing interests** None.

**References**

1. Frat JP, Thille AW, Mercat A, Girault C, Ragot S, Perbet S, et al. High-flow oxygen through nasal cannula in acute hypoxemic respiratory failure. N Engl J Med. 2015;372(23):2185–96.
2. Thille AW, Contou D, Fragnoli C, Cordoba-Izquierdo A, Boissier F, Brun-Buisson C. Non-invasive ventilation for acute hypoxemic respiratory failure: intubation rate and risk factors. Crit Care. 2013;17(6):R269.

#### O35 Prognosis factors of severe influenza in ICU and introduction delay of oseltamivir

##### Flore Richard^1^, Rafael Mahieu^2^, Hélène Le Gullou-Guillemette^3^, Nicolas Chudeau^4^, Jonathan Fahri^5^, Achille Kouatchet^1^

###### ^1^Service de réanimation médicale et médecine hyperbare, Centre Hospitalier Universitaire d’Angers, Angers, France; ^2^Réanimation médicale, Centre Hospitalier Universitaire d’Angers, Angers, France; ^3^Département de biologie des agents infectieux et pharmaco-toxicologie, Centre Hospitalier Universitaire d’Angers, Angers, France; ^4^Réanimation médico-chirurgicale, C.H. - Le Mans, Le Mans, France; ^5^Service des maladies du sang, Centre Hospitalier Universitaire d’Angers, Angers, France

####### **Correspondence:** Achille Kouatchet - ackouatchet@chu-angers.fr

*Annals of Intensive Care* 2017, **7(Suppl 1)**:O35

**Introduction** Influenza infection has a major impact on ICU hospitalizations during epidemic season, mainly affecting the elderly with exacerbation of their comorbidities.

To identify bad prognosis factors in severe influenza and the impact of delayed antiviral therapy among ICU patients infected with influenza.

**Patients and methods** We conducted a retrospective, observational study in Angers CHU medical ICU, from November 2009 to April 2015. Influenza infection was confirmed by Immunofluorescence. Demographic datas, comorbid conditions, antiviral treatment and time elapsed between ICU admission and treatment, clinical outcome, bacterial infection associated, type of virus, antibiotic, ventilation, renal injury, use of vasopressive drugs were recorded. Risk factors for death were analyzed by backward stepwise logistic regression.

**Results** The study population consisted of 85 patients whom mean age was 63 years old. 80% had at least one comorbidity. Influenza A infected 71% of our patients and type B 28%. [iz1] Most of them was admitted for respiratory distress and 59% were under invasive ventilation. The rate of death was 25% at day 28. 40% were under vasopressive drug. 84% received NAI among whom 76% received it in the first 2 days after ICU admission. Risk factors for death were, shock at the admission, immunosuppressive conditions and late administration of NAI. patients receiving NAI more than 48 h after ICU admission had a higher risk of death.

A multivariate analysis was performed. Shock at the admission was associated with mortality (OR −2.38: 95% CI 0.03–0.045) and time elapsed from ICU to [RM1] NAI less than 3 days was associated with survival at 28 day (OR 1.68: 95% CI 1.30–22.11).

See Table 6.

**Table 6:** See text for description

	Death d28	Alive d28	p
Baseline characteristics			
Age	66.1 ± 12.8	61.9 ± 17.2	0.3
Sex (M)	12 (54.5)	28 (45.2)	0.45
BMI	25.5 ± 5.3	28.1 ± 8.6	0.2
Corticosteroids in 10th days before ICU admission	5 (23.8)	5 (7.9)	0.052
Charlson score	3.2 ± 3.4	2.1 ± 2.5	0.16
IGS II	49.1 ± 20.8	36.5 ± 15.5	0.0039
Clinical presentation			
Fever (>38 °C)	18 (85.7)	40 (63.5)	0.056
Body aches	1 (4.8)	16 (25.4)	0.085
Initial influenza diagnostic test			0.77
Rapid diagnosis	8 (38.1)	26 (41.3)	
Immunofluorescence	11 (52.4)	28 (44.4)	
PCR	2 (9.5)	9 (14.3)	
Clinical complications			
Shock	18 (85.7)	16 (25.4)	<0.0001
ARDS	11 (52.4)	18 (28.6)	0.047
Acute renal failure	12 (57.1)	13 (20.6)	0.0015
Secondary bacterial infection	13 (61.9)	22 (34.9)	0.03
Details of antiviral treatment			
ICU to Neuraminh delay <3 days	9 (42.9)	45 (71.4)	0.018

**Conclusion** Cases of severely ill suspected influenza infections in epidemic period may benefit as soon as they are admitted in ICU from antiviral therapy. Our results add to the existing observational datas on the effectiveness of starting antiviral treatment with Neuraminidase Inhibitors as soon as Influenza is clinically suspected, even if suspicion appears at ICU admission and with delay from symptoms onset.

**Competing interests** None.

#### O36 Salbutamol nebulization during non-invasive ventilation in exacerbated COPD patient

##### Laetitia Bodet-Contentin^1^, Antoine Guillon^1^, Thierry Boulain^2^, Jean-Pierre Frat^3^, Denis Garot^1^, Déborah Le Pennec^4^, Laurent Vecellio^4^, Elsa Tavernier^5^, Pierre François Dequin^1^

###### ^1^Réanimation polyvalente, CHRU Hôpitaux de Tours, Tours, France; ^2^Réanimation médicale polyvalente, Hôpital de La Source, CHR Orléans, Orléans, France; ^3^Réanimation Médicale, CHU de Poitiers, Poitiers, France; ^4^Cepr (inserm u1100/ea 6305), aérosolthérapie et biomédicaments à visée respiratoire, Université François Rabelais, Tours, France; ^5^Inserm cic 1415, CHRU Hôpitaux de Tours, Tours, France

####### **Correspondence:** Laetitia Bodet-Contentin - laetitia.contentin@univ-tours.fr

*Annals of Intensive Care* 2017, **7(Suppl 1)**:O36

**Introduction** Non invasive ventilation (NIV) is a widely used technique to treat hypercapnic acute respiratory failure (ARF) in Chronic Obstructive Pulmonary Disease (COPD) patients. Nebulization of beta 2 agonists is recommended to treat COPD exacerbations. Nebulizing beta 2 agonist on NIV circuits could be potentially helpful but had been studied only in stable COPD patients. Our aim was to compare effectiveness of salbutamol and placebo nebulized in NIV circuit in patients admitted to the intensive care unit (ICU) for exacerbation of COPD. Aerosols were generated by a vibrating mesh nebulizer, whose optimal place in the circuit (just after the Y-piece) was determined by a preliminary bench study.

**Patients and methods** We conducted a double-blinded trial comparing salbutamol and placebo delivered in a random order as two aerosols separated by 60 min, during NIV, in 43 patients admitted to the ICU for ARF. A spirometry was obtained at several points before and after nebulizations, as were clinical and biological safety parameters.

**Results** Forced expiratory volume in one second (FEV1) increased significantly from baseline to 40 min after the end of salbutamol nebulization, when compared to placebo (+5.6 vs. −8.9%, p = 0.04). At the same time, potassium level decreased slightly in the placebo arm (p < 0.01), when glucose and lactate levels increased (NS). Symmetric changes were observed in the salbutamol arm after the second nebulization (+13.7 vs. −8.9%, p < 0.01). Nebulization was well tolerated in all patients.

**Conclusion** Placing the nebulizer immediately after the Y-piece is appropriate to deliver aerosols in NIV circuit and compatible with crimped circuit. Salbutamol nebulization is well tolerated during NIV and increase significantly FEV1 when compared to placebo. If this physiological effect is associated with clinical benefit remains to demonstrate.

**Competing interests** I, the undersigned, certify that the BANNISTER clinical trial «Beta Agonist Nebulization in Non Invasively ventilated Chronic Obstructive Pulmonary Disease (COPD) patients: Safety and Therapeutic Efficacy Range» , was funded by Association pour la Promotion à Tours de la Réanimation Médicale and the bench study by Inserm. The nebulizers were provided by Aerogen; the company was not involved in study design, data exploitation or drafting of the manuscript.

**References**

1. Calvert LD, Jackson JM, White JA, Barry PW, Kinnear WJ, O’Callaghan C. Enhanced delivery of nebulised salbutamol during non-invasive ventilation. J Pharm Pharmacol. 2006;58(11):1553–7.
2. Nava S, Karakurt S, Rampulla C, Braschi A, Fanfulla F. Salbutamol delivery during non-invasive mechanical ventilation in patients with chronic obstructive pulmonary disease: a randomized, controlled study. Intensive Care Med. 2001;27(10):1627–35.

#### O37 Effect of a musical intervention on tolerance and efficacy of non-invasive ventilation: the “MUSique pour l’Insuffisance Respiratoire Aigue—MUS-IRA” randomized controlled trial

##### Jonathan Messika^1^, Yolaine Martin^2^, Natacha Maquigneau^3^, Matthieu Henry-Laguarrigue^4^, Christelle Puechberty^5^, Annabelle Stoclin^5^, Serge Villard^2^, Aline Dechanet^6^, Didier Dreyfuss^2^, David Hajage^7^, Jean-Damien Ricard^8^

###### ^1^Service de réanimation médico-chirurgicale, CHU Louis Mourier, Colombes, Colombes, France; ^2^Réanimation médico-chirurgicale, Hôpital Louis-Mourier - APHP, Colombes, France; ^3^Réanimation, Centre Hospitalier Départemental - site de La Roche-sur-Yon, La Roche-sur-Yon, France, La Roche-sur-Yon, France; ^4^Réanimation, Centre Hospitalier Départemental - site de La Roche-sur-Yon, La Roche-sur-Yon, France; ^5^Réanimation-uscm, Gustave Roussy, Villejuif, France; ^6^Unité de recherche clinique, Hôpital Bichat-Claude Bernard (AP-HP), Paris, France; ^7^Département d’épidémiologie et de recherche clinique, Hôpital Louis-Mourier - APHP, Colombes, France; ^8^Service de Réanimation Médico-Chirurgicale, CHU Louis Mourier, Colombes, France

####### **Correspondence:** Jonathan Messika - jonathan.messika@aphp.fr

*Annals of Intensive Care* 2017, **7(Suppl 1)**:O37

**Introduction** One of major determinants of non-invasive ventilation (NIV) success is its tolerance. Numerous strategies have been assessed to improve this tolerance, such as pharmacological sedation or sophrology. Music therapy has been showed to be effective in healthcare settings, in particular in ICU invasively ventilated subjects [1]. We therefore investigated the effect of a musical session provided by a trained caregiver on NIV tolerance and efficacy in ICU patients with acute respiratory failure.

**Patients and methods** MUS-IRA is a randomized 3-centers, 3-arm open-label trial (PHRIP 2013). Subjects included were adult acute respiratory failure patients for whom the physician in charge considers NIV as indicated with a level of consciousness allowing a benefit from the musical intervention (Glasgow Coma Scale >11). Non inclusion criteria were contra-indication to NIV; severe hearing impairment; withdrawal of life sustaining therapies with expected survival of less than 48 h; subjects included in another trial dealing with acute respiratory failure. NIV was conducted in the same fashion in the three arms of randomization. The “musical intervention” subjects received a 30-min L-type music session with the MUSIC CARE© software (2), with a sleeping mask concealing the eyes. The “sensory deprivation” group had the sleeping mask and the insulating around-ear headphone, during a 30-min period. The “NIV alone” group had their NIV conducted as it is usually in our ICUs.

The main objective was to determine if a musical intervention improved NIV tolerance, measured by respiratory comfort, and ventilation parameters at 30 min of NIV in comparison to conventional care. The respiratory comfort was assessed by a nurse or nurse assistant blinded to the treatment arm with a numeric visual scale (from 0 to 10) at the initiation (T0), after 30 min (T30), and at different time points of each NIV session. The primary endpoint was the change in respiratory comfort at initiation and after 30 min of the first NIV session. The secondary endpoints were NIV failure and the percentage of patients requiring anxiolytics or sedative during NIV sessions. The comparison performed in a pre-specified fashion was between “musical intervention” group and “NIV-only” group. A total of 99 subjects had to be randomized in order to show a 2-unit difference in respiratory comfort between two groups. This number was extended to 114 because of missing data on the primary endpoint.

**Results** Among the 114 subjects randomized (May 2015 to May 2016—“musical intervention” group n = 37; “sensory deprivation” group n = 38, “NIV-only” group n = 39), median age was 67 years (60–74), and 63 were men (55.3%). Mean baseline respiratory comfort for “musical intervention”, “sensory deprivation” and “NIV-only” was 4.34 ± 3.01, 4.24 ± 2.59, 3.89 ± 3.03 respectively for the first NIV session (p = 0.74). Mean change in respiratory comfort between T0 and T30 for “musical intervention”, “sensory deprivation” and “NIV-only” was respectively 0.54 ± 3.57; 0.55 ± 2.33; 0.66 ± 2.9. The comparison between “musical intervention” and NIV-alone group yielded a p-value of 0.91. NIV failure during ICU stay was evidenced in 16.2%, 10.5%, 12.8% (“musical intervention”, “sensory deprivation” and “NIV-only”, respectively, p = 0.74). No subjects required the administration of anxiolytics or sedatives to cope with the first NIV session in all groups.

**Discussion** Our study failed to evidence a significant effect of a musical intervention on the reduction of respiratory discomfort during the first NIV session of acute respiratory failure ICU patients. One may hypothesize that the improvement provided by NIV (which patients received in all three groups) was such that it outweighed and/or masked a potential effect of the musical intervention. Further analysis are planned to investigate the respiratory comfort and the evolution of physiological parameters during the subsequent sessions.

**Conclusion** Applying a musical intervention early in the course of a treatment by NIV during acute respiratory failure did not modify respiratory comfort. Further research is warranted.

**Competing interests** None.

**References**

1. Chlan LL, Weinert CR, Heiderscheit A, Tracy MF, Skaar DJ, Guttormson JL, et al. Effects of patient-directed music intervention on anxiety and sedative exposure in critically ill patients receiving mechanical ventilatory support: a randomized clinical trial. JAMA. 2013;309(22):2335–44.
2. Guetin S GP Blayac JP, Eledjam JJ. Une nouvelle technique contrôlée de musicothérapie dans la prise en charge des douleurs viscérales aiguës et chroniques. Doul Analg. 2005;(1):19–25.

#### O38 Apnoeic oxygenation via high-flow nasal oxygen combined with non-invasive ventilation preoxygenation for intubation in hypoxaemic patients in intensive care unit: the randomised OPTINIV study

##### Audrey De Jong^1^, Marion Monnin^1^, Mehdi Girard^1^, Gérald Chanques^1^, Nicolas Molinari^2^, Samir Jaber^1^

###### ^1^DAR B, Hôpital Saint Eloi, Montpellier, France; ^2^Dim, Hôpital La Colombière, Montpellier, France

####### **Correspondence:** Audrey De Jong - audreydejong@hotmail.fr

*Annals of Intensive Care* 2017, **7(Suppl 1)**:O38

**Introduction** Tracheal intubation in the intensive care unit (ICU) is associated with severe life-threatening complications including severe hypoxemia [1]. Preoxygenation before intubation has been recommended in order to decrease such complications. Noninvasive ventilation (NIV)-assisted preoxygenation allows increased oxygen saturation during intubation procedure [2]. However, the NIV-mask has to be taken off after preoxygenation to allow the passage of the tube through the mouth. High-flow nasal cannula oxygen (HFNC) has a potential of apneic oxygenation during the apnea period following the preoxygenation with NIV. We hypothesized that application of HFNC combined with NIV was more effective at reducing oxygen desaturation during the intubation procedure compared to NIV alone for preoxygenation in hypoxemic ICU patients with acute respiratory failure.

**Patients and methods** We did this randomised, controlled, single-center trial with assessor-blinded outcome assessment in patients admitted to ICU. Hypoxaemic patients requiring orotracheal intubation for respiratory failure were randomised to receive preoxygenation using HFNC [flow = 60 L/min, fraction of inspired-oxygen (FIO_2_) = 100%] combined with NIV (pressure support = 10 cmH_2_O, positive end-expiratory pressure = 5 cmH_2_O, FiO_2_ = 100%) in the interventional-group or using NIV alone in the reference-group (Fig. 6). The primary outcome was the minimal oxygen saturation (SpO_2_) during the intubation procedure. Secondary outcomes were intubation-related complications and ICU-mortality. The OPTINIV trial was registered with ClinicalTrials.gov, number NCT02530957.

**Fig. 6:**
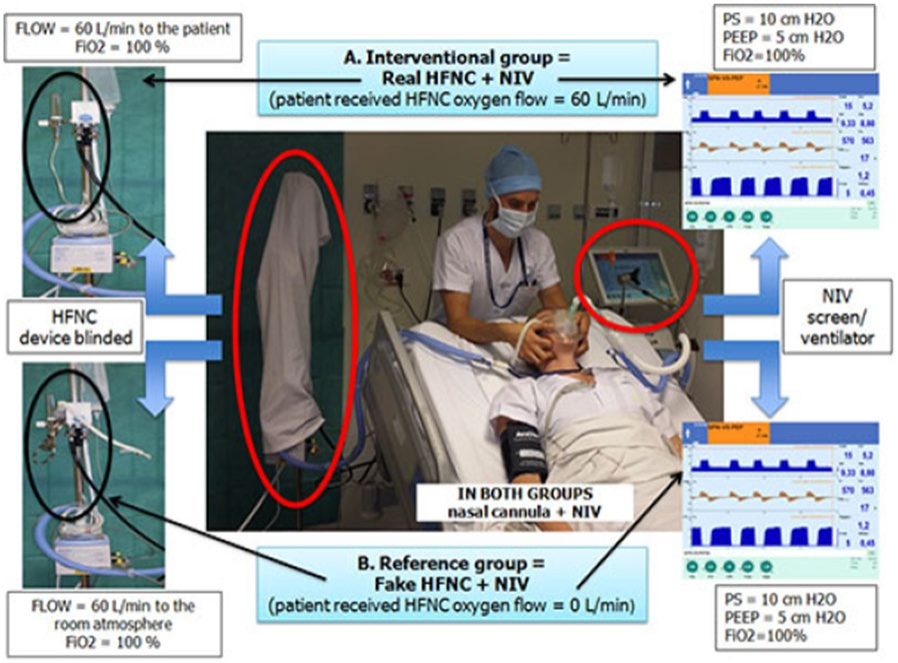
Blinding sequence of the OPTINIV study

**Results** Between July-2015 and February-2016, we randomly assigned 25 and 24 patients in the interventional and reference groups. During the intubation procedure, median minimal SpO_2_ values were significantly higher in the interventional-group when compared with the reference-group (100 [95–100] vs 96 [92–99]%, *p* = 0.029). After exclusion from analysis of two patients for protocol violation, no (0%) patients in the interventional-group and five (21%) patients in the reference-group had a SpO_2_ below 80% (*p* = 0.050). We recorded no significant difference in intubation-related complications and ICU-mortality between groups.

**Conclusion** A novel strategy of preoxygenation adding apnoeic high-flow oxygen to NIV to perform orotracheal intubation in hypoxaemic patients is more effective at reducing oxygen desaturation in comparison to the reference-method using NIV alone.

**Competing interests** None.

**References**

1. De Jong A, Molinari N, Terzi N, Mongardon N, Arnal JM, Guitton C, Allaouchiche B, Paugam-Burtz C, Constantin JM, Lefrant JY et al. Early identification of patients at risk for difficult intubation in the intensive care unit: development and validation of the MACOCHA score in a multicenter cohort study. Am J Respir Crit Care Med. 2013; 187(8):832–9.
2. Baillard C, Fosse JP, Sebbane M, Chanques G, Vincent F, Courouble P, Cohen Y, Eledjam JJ, Adnet F, Jaber S. Noninvasive ventilation improves preoxygenation before intubation of hypoxic patients. Am J Respir Crit Care Med. 2006;174(2):171–7.

#### O39 Feasibility and validity of an observational scale as a surrogate of dyspnea in non-communicating intubated patients in the intensive care unit (ICU)

##### Maxens Decavèle^1^, Côme Bureau^1^, Sébastien Campion^2^, Roukia Ainsouya^2^, Julien Mayaux^1^, Hélène Prodanovic^1^, Marie-Cécile Niérat^2^, Mathieu Raux^3^, Thomas Similowski^1^, Alexandre Demoule^1^

###### ^1^Service de pneumologie et réanimation médicale, Pitié-Salpêtrière Hospital, Paris, France; ^2^Inserm umr_s 1158 “neurophysiologie respiratoire expérimentale et clinique”, Pitié-Salpêtrière Hospital, Paris, France; ^3^Département d’anesthésie-réanimation et umr_s 1158, Hôpital Universitaire Pitié-Salpêtrière, Paris, France

####### **Correspondence:** Maxens Decavèle - maxencesar@hotmail.fr

*Annals of Intensive Care* 2017, **7(Suppl 1)**:O39

**Introduction** Dyspnea is common and severe in intensive care unit (ICU) patients. In these patients, dyspnea is associated with a poorer outcome. The measurement of dyspnea involves a self-assessment by the patient with a visual analogic scale (VAS-Dyspnea), which by definition requires a certain level of communication. However, many intubated critically-ill patients are unable to reach this level of communication, which makes very difficult the assessment of dyspnea.

Recently, our team has developed, a behavioral score named IC-RDOS [1], which is reliable to predict severe dyspnea in ICU patients. This score has been only validated in non-intubated conscious ICU patients.

The objective of our study was to validate this score in intubated communicating and non-communicating (NC) patients. IC-RDOS was compared to VAS-Dyspnea in communicating patients and to electrophysiologic markers of dyspnea such as surface electromyographic (EMG) activity of extra diaphragmatic inspiratory muscles [2], in NC patients.

**Materials and methods** Between February and August 2016, invasively mechanically ventilated patients with clinical respiratory discomfort were included. The IC-RDOS and EMG activity of the Parasternal and Alae Nasi were measured before (baseline, BL) and after one or two interventions aiming at reducing dyspnea. These interventions consisted in an optimization of ventilator settings (Opt) followed, if necessary, by the injection of opioids. The VAS-Dyspnea was only recorded in communicating patients. Non-communicating state was defined by the impossibility to perform a VAS-Dyspnea. This work was supported by the “Fond de dotation Recherche en Santé Respiratoire” and the “Fondation du Souffle”.

**Results** 35 patients (age 67 [56–76], SAPS II 51 [35–61]; med [IQR]) were included; 17 were NC. Among the 18 communicating patients we observed a strong correlation between the IC-RDOS and the VAS-Dyspnea (rho = 0.545, p = 0.038). Among NC patients, IC-RDOS decreased from 6.2 [4.3–6.6] at BL to 4.4 [2.7–6.3] (p = 0.286) and to 4.3 [2.9–4.8] (p = 0.024) after Opt and opioids injection, respectively. Among NC patients, we observed concomitantly, a significant diminution of the EMG activity (EMGAUC) of the Alae Nasi compared to to BL (−38% [p = 0.003] and −64% [p = 0.008] after Opt and morphine injection respectively) and a significant diminution of the EMGAUC of the Parasternal relative to BL (−65% [p = 0.039]) after morphine injection. Among NC patients, we found a significant positive correlation between IC-RDOS and Parasternal EMGAUC (rho = 0.538, p = 0.060).

**Conclusion** IC-RDOS is a reliable surrogate of dyspnea in communicating and non-communicating intubated ICU patients.

**Competing interests** None.

**References**

1. Persichini R, Gay F, Schmidt M, Mayaux J, Demoule A, Morélot-Panzini C, Similowski T. Diagnostic accuracy of respiratory distress observation scales as surrogates of dyspnea self-report in intensive care unit patients. Anesthesiology. 2015;123(4):830–7.
2. Schmidt M, Kindler F, Gottfried SB, Raux M, Hug F, Similowski T, Demoule A. Dyspnea and surface inspiratory electromyograms in mechanically ventilated patients. Intensive Care Med. 2013;39(8):1368–76.

#### O40 Prevalence and impact of pleural effusion during weaning from mechanical ventilation: a prospective multicenter study

##### Keyvan Razazi^1^, Florence Boissier^2^, Mathilde Neuville^3^, Sebastien Jochmans^4^, Martial Tchir^5^, Faten May^1^, Guillaume Carteaux^1^, Nicolas de Prost^1^, Armand Mekontso Dessap^1^

###### ^1^Réanimation Médicale, Hôpital Henri Mondor, Créteil, France; ^2^Service de réanimation médicale, CHU de Poitiers, Paris hegp service de réanimation médicale, France; ^3^Service de réanimation médicale et infectieuse, Hôpital Bichat-Claude Bernard-APHP, Paris, France; ^4^Service de Réanimation, Centre Hospitalier Marc Jacquet, Melun, France; ^5^Réanimation polyvalente - surveillance continue, Ctre Hospitalier Intercommunal de Villeneuve Saint Georges, Lucie et Raymond AUBRAC, Villeneuve-Saint-Georges, France

####### **Correspondence:** Keyvan Razazi - keyvan.razazi@aphp.fr

*Annals of Intensive Care* 2017, **7(Suppl 1)**:O40

**Introduction** Pleural effusions are common in critically ill patients. Bedside lung ultrasonography (LU) outperformes chest radiograph for the diagnosis of pleural effusion. Pleural effusion drainage improves oxygenation and respiratory mechanics of patients under mechanical ventilation. However, the role of pleural effusion during the weaning process is unclear.

**Patients and methods** We performed a prospective multicenter study in six ICUs in France. We used ultrasonography to screen for pleural effusion during the weaning process. Weaning failure was defined as failure of spontaneous breathing trial (SBT) or extubation. Patients were included the day of a first weaning test after at least 24 h of mechanical ventilation. We assessed factors associated with pleural effusion and its evolution.

**Results** Two hundred and forty nine patients were included. Median duration of mechanical ventilation was 4 [2–8] days before a first SBT. Forty-seven patients (19%) failed a first SBT and 31 (15%) had extubation failure (reintubation or death within the 7 days following extubation). Pleural effusion was detected in 139 patients (56%) the day of SBT. Most of pleural effusions were homogeneously anechoic (98%) and associated with pulmonary condensation or atelectasis (70%). Interpleural distance was higher among patients with left ventricular diastolic dysfunction, cancer history, ARDS or dialysis before the first SBT. A higher interpleural distance was associated with SBT failure and weaning failure. Patient with weaning failure had more often large pleural effusion (34 vs 12%, p < 0.01). Among patient failing the first SBT and followed up during the weaning process, either diuretic nor fluid balance change altered the interpleural distance within 24–48 h.

**Conclusion** Pleural effusion is frequent during the weaning process and is associated with weaning failure. Further studies are needed to test whether a strategy aimed at draining large pleural effusions has the potential to decrease duration of weaning process.

**Competing interests** None.

#### O41 Characterization of emergency physicians’ recourse to intensive care unit physicians: a prospective multicenter study

##### Rougevin-Baville Pauline^1^, Andronikof Marc^2^, Jean Pierre Bedos^3^, Stéphane Legriel^4^, Koukabi Mehrsa^5^, Carole Mauger-Briche^6^, François Mijon^7^, Pierre Trouiller^1^, Benjamin Sztrymf^1^

###### ^1^Réanimation polyvalente, Hôpital Antoine Béclère, Clamart, France; ^2^Service d’accueil des urgences, Hôpital Antoine Béclère, Clamart, France; ^3^Réanimation médico-chirurgicale, Centre Hospitalier de Versailles, Le Chesnay, France; ^4^Réanimation, Centre Hospitalier de Versailles, Le Chesnay, France; ^5^Service d’accueil des urgences, Centre Hospitalier de Versailles, Le Chesnay, France; ^6^Service d’accueil des urgences, C.H. Intercommunal de Meulan-Les Mureaux, Meulan-en-Yvelines, France; ^7^Réanimation polyvalente, C.H. Intercommunal de Meulan-Les Mureaux, Meulan-en-Yvelines, France

####### **Correspondence:** Benjamin Sztrymf - benjamin.sztrymf@aphp.fr

*Annals of Intensive Care* 2017, **7(Suppl 1)**:O41

**Introduction** Recourses to the intensive care units (ICU) physicians by the emergency department (ED) physicians are frequent, estimated around once a day. Nevertheless, these recourses are inherently unpredictable. A good relationship seems very important for an adequate care of the patients and a rational use of hospital resources. The collaboration between these two entities remains poorly described. Therefore, we aimed to characterize those recourses.

**Patients and methods** This is prospective observational multicenter study conducted during 1 month (march 2016) in the ED and ICU of three hospitals of Paris suburb: Hôpital Antoine Béclère (398 beds university hospital), Hôpital André Mignot (702 beds) and Centre hospitalier intercommunal de Meulan les Mureaux (517 beds). We first conducted a preliminary survey describing the baseline evaluation of those recourses, with a questionnaire distributed to all physicians in the participating units. Therefater, we did a prospective work to characterize the reality of theses recourses. A questionnaire was fulfilled at each call, by the ED and the ICU physicians, blinded from his colleague. Finally, to evaluate the “non recourse” too, we recorded the characteristics of ICU patients admitted from another ward of the hospital, within seven days after triage in the ED.

**Results** Preliminary survey: 40 emergency physicians and 24 intensivists answered (response rate: 87%). Intensivists declared that generally they estimate that the recourse is justified (87% of answers), in an appropriate timing (72%), and that they generally agree with the ED’s physician about the emergency level (92%).

- Recourse characterization: during the study period, there were 111 recourses among the 12 674 patients admitted in the ED. We had bilateral data in 50% of cases. The mean recourse frequency was 1.2/day, representing 1/114 patients admitted in the ED, with a great variation between hospitals: from 1/71 to 1/158 patients. 50% of these recourses occurred during daytime (8 AM to 6 PM).

Intensivist went to the ED in 81% of cases. For the remaining cases, a direct admission was decided in 40% of cases and a phone advice was given in 60%. The mean time of arrival to the ED was 10 min and the mean time spent in the ED was 32 min. Life sustaining treatment limitation was a frequent recourse’s reason (9.4%).

There was a non significant trend toward an estimation of higher severity by emergency physicians (37.3 vs 19.8%, p = 0.19).

The exchange’s climate was rated excellent or good in 81% of cases. It was more often “neutral” when an ED’s resident was calling (45 vs 5.7%, p = 0.048). In those cases (8% of recourses) intensivists complained about an insufficient supervision of the resident.

Main demand of the ED physician was an ICU admission (80%), and disagreement occurred in 36% of cases. Eventually, 55% of patients were admitted in ICU.

“Non recourse” involved 20 patients. It was estimated according to their file that 7 of them (40%) harbored severity signs at the initial ED evaluation, but the intensivist wasn’t solicited. ICU admission was denied for one patient despite intensivist’s evaluation and evidence of severity.

**Discussion** The ICU physicians seem to be solicited by the ED physicians roughly once a day. There is some “non-recourse” with delayed admission in ICU which could be deleterious for the patient.

The climate of medical exchanges is usually good, although this appreciation may be downgraded by a resident’s call.

Except for the Glasgow coma scale (which was suggested in our questionnaire), no severity or prognostic score was used as objective assessment of severity.

Life sustaining treatment limitation is a frequent reason for ICU recourse. These complex situations require a collegial discussion and recourses were estimated justified by the intensivists.

**Conclusion** Recourses to ICU physicians by ED physicians seem to be adapted and the medical exchanges happen in good relationship conditions. The frequency of these recourses is very variable among hospitals. ED physicians are mainly asking for an admission in ICU. Our work seems to point out some improvement ways, such as the use of severity scores, and a better supervision of the residents when asking for an intensivist’s evaluation.

**Competing interests** None.

#### O42 Analysis of invasive mechanical ventilation as managed by French pre-hospital emergency physicians

##### Pierre Cretallaz^1^, Romain Mermillod-Blondin^1^, Jean-Christophe Richard^1^, Dominique Savary^1^

###### ^1^Pôle sau - samu 74 - smur - réanimation, C.H. Annecy Genevois, Metz-Tessy, France

####### **Correspondence:** Pierre Cretallaz - pierre.cretallaz@gmail.com

*Annals of Intensive Care* 2017, **7(Suppl 1)**:O42

**Introduction** If French activity in emergency departments is similar to that of other countries, the concept of pre-hospital medicalization remains specific. Invasive mechanical ventilation (IMV) is a common practice in French pre-hospitals; however, few data is available.

**Materials and methods** An epidemiological study was conducted at two French pre-hospital departments throughout 2015 and 5 months of 2016. Eligible patients were those receiving IMV at pre-hospitals and aged >18 years. The aim was to define pre-hospital IMV practices and determine their consequences on mechanical and blood gases.

**Results** Eighty patients were included with 71.3% being men and middle-aged 55 years. J28 survival was 60%.

The aetiologies of intubation were 38.8% traumatic, 16.5% of cardiac origin, and 12.5% of brain vascular origin.

The preferred mode of ventilation was assist-control volume-control (98.75%). The median tidal volume was 6.9 ml/kg of predicted body weight (PBW; interquartile range (IQR) 6.4–7.7 ml/kg PBW) with a range of 5.1–10.4 ml/kg PBW. Lung protective volume ventilation was employed in 70.7% of patients (6–8 ml/kg PBW).

The median PEEP was 5 mmHg (IQR 5–5 mmHg). 34% of patients had a change of PEEP’s level compared to factory settings. The average respiratory rate was 15 cycles/min (SD 14–16 cycles/min).

The median FiO_2_ was 100% (IQR 60–100%), with 58% of patients exposed to FiO_2_ = 100%. In all, 56% of patients had hyperoxemia gases (PaO_2_: 126 mmHg, IQR 96–307 mmHg) and 56% hypercapnia gases (PaCO_2_: 46.3 mmHg, IQR 40.1–52.9 mmHg). Overall, 46.3% of patients needing brain protection (brain injury, stroke) had hypercapnia gases.

**Discussion** Lung-protective ventilation is not defined strictly by tidal volume between 6–8 ml/kg PBW, but is a strategy that includes PEEP, respiratory frequency, and FiO_2_ (1).

This study suggests infrequent use of PEEP titration in favour of delivery of a high level of oxygen and infrequent utilisation of lung-protective ventilation in French pre-hospital medicalization.

ARDS is a syndrome that should be prevented by generalization of a lung-protective ventilation strategy for all of patients (2).

**Conclusion** French pre-hospital invasive mechanical ventilation must be optimized with a full strategy of lung-protective ventilation with regards to tidal volume, pressure, oxygenation or capnia.

A comparative study following a guided ventilation strategy is currently underway to determine if a ventilatory protocol would produce better results than the free will of the emergency physician.

**Competing interests** None.

**References**

1. Fuller BM, Mohr NM, Hotchkiss RS, Kollef MH. Reducing the burden of acute respiratory distress syndrome: the case for early intervention and the potential role of the emergency department. Shock.2014;41 (5):378–87.
2. Levin MA, McCormick PJ, Lin HM, Hosseinian L, Fischer GW. Low intraoperative tidal volume ventilation with minimum PEEP is associated with increased mortality. BR J Anaesth. 2014;113(1):97–108.

#### O43 Intra hospital transport’s complications: incidence and risk factors

##### Ines Sedghiani^1^, Hamdi Doghri^2^, Asma Jendoubi^1^, Dhekra Hamdi^1^, Mohamed Ali Cherif^2^, Youssef Zied El Hechmi^2^, Jerbi Zouheir^2^

###### ^1^Emergency and Intensive Care Department, Hôpital Habib Thameur, Tunis, Tunisia; ^2^Emergency and Intensive Care Department, Hopital Habib Thameur, Tunis, Tunisia

####### **Correspondence:** Ines Sedghiani - sedghiani.ines@gmail.com

*Annals of Intensive Care* 2017, **7(Suppl 1)**:O43

**Introduction** Caring for patients during intra hospital transport (IHT) is a high-risk activity. Adverse events during transport are frequent and may have significant consequences for the patient. The aim of this study was to assess the incidence of complications occurring during the IHT and to analyze the causes of such complications.

**Patients and methods** We prospectively describe IHT from the emergency department, realized from January 2016 to March 2016. Were included in the study IHT of compromised patients for whom critical care monitoring was needed and emergency physician is required. Clinical characteristics of patient’s departure and technical equipments (mechanical ventilation, drugs) were noted. Complications were defined as follows: patient related problems (desaturation, haemodynamic instability, arrhythmia, extubation, acute change in mental status, death) and ventilator related problems (breakdown or defect of the material).

**Results** During the inclusion period, 102 IHT were carried out. The IHT were realized for imaging procedure in 41 cases and for transferring patients to the intensive care unit in 24 cases and to the other wards in 37 cases. The median IHT duration was 15 min [10–30]. Twenty patients (19%) were mechanically ventilated. The majority of IHT (60%) were performed by the night shift emergency team. The incidence of complications was 44% (45 patients). Most events were related to haemodynamic instability in 25 cases, desaturation in 22 cases, agitation in 14 cases and cardiac arrest in 2 cases and one death. Therapeutic interventions were volume resuscitation in 13 cases, optimization of sedation in 12 cases, vasopressor managment in 12 patients and cardiopulmonary resuscitation in 3 cases.

The occurrence of complications during transport was significantly increased in mechanically ventilated patients (p = 0.009), especially with inspiratory oxygen fraction >0.5 (p = 0.00), sedation before transport (p = 0.001), vasopressor requirement before transport (p = 0.03) and with the night shift team (p = 0.007). Sedation and mechanical ventilation were the independent risk factors of IHT complications.

**Conclusion** This study confirms that the intrahospital transport of compromised patients leads to a significant number of complications. This finding emphasises the need of improving medical skills during IHT.

**Competing interests** None.

#### O44 Influence of shift duration on cognitive performance of emergency practitioners: a prospective cross-sectional study

##### Nicolas Persico^1^, Francois Maltese^2^, Cécile Ferrigno^1^, Amandine Bablon^1^, Cécile Marmillot^1^, Laurent Papazian^2^, Antoine Roch^2^

###### ^1^Service d’accueil des urgences adultes, Hôpital Nord APHM, Marseille, France; ^2^Réanimation DRIS, Hôpital Nord APHM, Marseille, France

####### **Correspondence:** Nicolas Persico - nicolas.persico@ap-hm.fr

*Annals of Intensive Care* 2017, **7(Suppl 1)**:O44

**Introduction** Shift work including night work is responsible for sleep deprivation and tiredness. The relationship between tiredness and the risk of medical errors is now commonly accepted. The main objective of this study was to evaluate the cognitive performance of emergency practitioners after night shift of 14 h and after work shift of 24 h and to compare them with tests performed after a rest night at home.

**Materials and methods** We conducted an observational, prospective, single-center, randomized and cross-over comparative study. Emergency practitioners (staff physicians and residents) were eligible if they worked at least one night from 6:30 p.m. to 8:30 a.m. (14 h) and a day followed by consecutive night from 8:30 a.m. to 8:30 a.m. (24 h) within three consecutive months. Emergency practitioners participated to three cognitive assessments separated by at least 7 days: after a night of rest, after a night shift of 14 h and after a work shift of 24 h (including a night shift). The evaluation after night rest took place at least 3 days after the previous night shift. Three assessments were randomly assigned via sealed envelopes within a period of 3 months for each participant. Each participant was his own control and was evaluated by the same examiner. A psychologist formed four voluntary examiners to assess cognitive performance. Psycho-cognitive assessment began with self-evaluation of tiredness, attention, mood and lack of sleep (visual scales). Then an examiner evaluated participant’s cognitive performance according to the Wechsler Adult Intelligence Scale and the Wisconsin Card Sorting Test. Four cognitive skills were assessed: speed of processing information, working memory capacity, cognitive flexibility and perceptual reasoning. To test our main hypothesis, we performed an analysis of variance with repeated measures.

**Results** Forty emergency practitioners were included: 19 staff physicians and 21 residents. A staff physician and a resident declined to participate. Staff physicians were 36.2 ± 7.1 years old and residents were 26.8 ± 1.0 years old (*p* < 0.001). Average number of night shift per month was 4.4 ± 1.1 for staff physicians and 3.2 ± 0.6 for residents (*p* < 0.001). Amount of sleep, phone wakes up and stand ups were not different among staff physicians and residents. For all participants, no cognitive capacity was significantly altered after a night shift of 14 h when compared with performance after a night of rest. Conversely, three cognitive abilities were impaired after a work shift of 24 h when compared with performance after a night of rest: speed of processing information (11.2 ± 2.7 vs 12.4 ± 3.2; *p* < 0.003), working memory capacity (10.1 ± 2.9 vs 11.6 ± 3.0; *p* < 0.001) and perceptual reasoning (8.4 ± 2.7 vs 10.6 ± 2.8; *p* < 0.001). In absolute percentage, those performances were 10 to 20% lower after work shift of 24 h than after night of rest. Only cognitive flexibility scores were not significantly different in this condition. Working memory capacity was the only cognitive ability significantly altered after a work shift of 24 h versus a night shift of 14 h (10.1 ± 2.9 vs 11.6 ± 3.0; *p* < 0.01) whereas other cognitive abilities were all lower after a work shift of 24 h but without significant difference. Regarding the influence of medical experience, cognitive performance of staff physicians compared to residents were not statistically different after a night shift of 14 h and after a work shift of 24 h. There was no significant correlation between self and hetero assessment.

**Conclusion** The cognitive abilities of emergency practitioners were significantly altered following a work shift of 24 h whereas they were not significantly different from rest condition after a night shift of 14 h. Limiting 24 h shift work for emergency department should be considered and further evaluated.

**Competing interests** None.

#### O45 In hospital medical emergency calls: epidemiology and patients outcomes

##### Ines Sedghiani^1^, Hamdi Doghri^2^, Dhekra Hamdi^1^, Mohamed Ali Cherif^2^, Youssef Zied El Hechmi^2^, Jerbi Zouheir^2^

###### ^1^Emergency and intensive care department, hôpital habib thameur, Tunis, Tunisia; ^2^Emergency and intensive care department, Hopital Habib Thameur, Tunis, Tunisia

####### **Correspondence:** Ines Sedghiani - sedghiani.ines@gmail.com

*Annals of Intensive Care* 2017, **7(Suppl 1)**:O45

**Introduction** Medical emergency team is called to provide acute care for compromised patients outside intensive care unit. While waiting for his arrival, medical ward’s physicians must initiate patient rescue.

The aim of this study was to describe clinical characteristics and outcomes of patients who experience medical emergency team calls and to evaluate initial care provided in medical specialty wards.

**Patients and methods** It’s a two-months prospective study (03/2016–04/2016) including all in hospital emergency calls. These calls were given by medical wards teams (dermatology, Gastroenterology, internal medicine, dermatology, cardiology and radiology) to the emergency physician when a deterioration in their patient’s condition was documented. Emergency and intensive care department emergency calls were excluded.

**Results** There were a total of 51 calls for 40 patients, principally hospitalized in internal medicine ward (21) and cardiology ward (14). Emergency calls were given by a physician in 47 cases and a nurse in 4 cases, during the night in 32 cases (62%). The greater number of calls was received Monday (13) and the week- end (11). Cardiac arrest (7), respiratory distress (28) and hemodynamic instability (16) were the reason’s call. The median emergency team arrival time was 10 min. The majority of patients (88%) required acute care for on average 35 min spent time. The vital signs were monitored and adequate therapy was initiated by the attending physician for 17 (33%) patients. Cardiovascular support was necessary for 24 (47%) patients, respiratory assistance was necessary for 3 patients. A transfer for the intensive care unit was indicated for 16 patients (31%) and delayed for 13 patients because of a lack of beds. In hospital mortality was 23% (n = 12). In hospital cardiac arrest and coma (Glasgow coma scale ≤8) were the principal prognostic factors. Delayed emergency time arrival was not associated with a greater mortality.

**Conclusion** In hospital emergencies were associated with an increased mortality. A better recognition of the instable patients may improve their prognostic.

**Competing interests** None.

#### O46 A new approach of sepsis heterogeneity

##### Grégory Papin^1^, Sébastien Bailly^2^, Claire Dupuis^1^, Stephane Ruckly^3^, Marc Gainnier^4^, Laurent Argaud^5^, Elie Azoulay^6^, Adrie Christophe^7^, Bertrand Souweine^8^, Dany Goldgran-Toledano^9^, Guillaume Marcotte^10^, Jean-Marie Forel^11^, Romain Sonneville^12^, Anne Sylvie Dumenil^13^, Michaël Darmon^14^, Maïté Garrouste-Orgeas^15^, Schwebel Carole^16^, Jean-François Timsit^17^

###### ^1^75018, INSERM U1137 équipe 5, Université Paris Diderot, Paris, France; ^2^Equipe 11, Institut Albert Bonniot - Inserm U823, La Tronche, France; ^3^Reanimation, Hôpital Michallon, Grenoble, France; ^4^Réanimation des urgences médicales, Hôpital de la Timone, Marseille, France; ^5^Réanimation Médicale, Hospices Civils de Lyon - Groupement Hospitalier Edouard Herriot, Lyon, France; ^6^Réanimation médicale, Hôpital Saint-Louis, Paris, France; ^7^75014, Hôpital Cochin, Paris, France; ^8^Réanimation médicale, CHU Gabriel-Montpied, Clermont-Ferrand, France; ^9^Réanimation polyvalente, Centre Hospitalier Général, Gonesse, France; ^10^Reanimation medicale, Assistance Publique Hôpitaux de Paris, Paris, France; ^11^Réanimation DRIS, Hôpital Nord APHM, Marseille, France; ^12^Service de réanimation médicale et infectieuse, Hôpital Bichat-Claude Bernard-APHP, Paris, France; ^13^Réanimation chirurgicale, Hôpital Antoine Béclère, Clamart, France; ^14^Réanimation Médicale, CHU Saint-Etienne - Hôpital Nord, Saint-Étienne, France; ^15^Réanimation, Fondation Hopital Saint Joseph, Paris, France; ^16^38, C.H.U de Grenoble C.H.U, La Tronche, France; ^17^Réanimation médicale et infectieuse, Hôpital Bichat-Claude Bernard, Paris, France

####### **Correspondence:** Grégory Papin - grego.papin@gmail.com

*Annals of Intensive Care* 2017, **7(Suppl 1)**:O46.

**Introduction** Reducing the heterogeneity of patients included in clinical trials for sepsis is essential. The aim of this study was to identify more homogeneous clusters of patients with sepsis and septic shock according to their initial clinical and biological characteristics.

**Patients and methods** All patients admitted for sepsis or septic shock, according to the new Sepsis 3.0 definition, were included from a national prospective multicenter ICU cohort. A first test set of patients was used in an unsupervised clustering to build clusters independently of patient’s prognosis. After description of mains characteristics of each cluster, 3 years mortality was compared with log rank test. A binary tree was built to assign new patients into cluster and evaluated using the validation set.

**Results** The test set included 4050 (67%) patients admitted for a first episode of sepsis. Six distinct clusters were identified (Fig. 7). Three years mortality was 21% [16–26%] for COPD exacerbation cluster, 26% [24–28%] for pulmonary sepsis cluster, 37% [33–40%] for surgical sepsis cluster, 27% [19–34%] for meningo-encephalitis cluster, 47% [41–52%] for immunocompromised patients cluster and 47% [44–50%] for chronic diseases cluster (p < .001). Binary tree identified 6 discriminant variables to assign patients into clusters: lung infection, surgical admission, bronchial infection, meningeal infection, hematological malignancy and chronic heart failure. Identical patient profiles were found in the validation set.

**Fig. 7:**
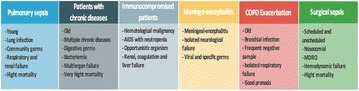
See text for description

**Conclusion** Six clusters of ICU patients admitted for sepsis or sepsis shock were identified. All clusters contained patients who met the new definitions of sepsis. Despite this, these clusters have a very high heterogeneity prognosis. Considering these clusters may improve homogeneity of patient’s enrolled in future clinical trials.

**Competing interests** None.

#### O47 Antimicrobial strategy for severe community-acquired Legionnaires’ disease: a multicentre retrospective observational study

##### Jerôme Cecchini^1^, Samuel Tuffet^1^, Romain Sonneville^2^, Muriel Fartoukh^3^, Julien Mayaux^4^, Damien Roux^5^, Achille Kouatchet^6^, Florence Boissier^7^, Martial Tchir^8^, Martial Thyrault^9^, Eric Maury^10^, Sebastien Jochmans^11^, Mekontso Dessap Armand^12^, Christian Brun-Buisson^1^, Nicolas de Prost^1^

###### ^1^Réanimation Médicale, Hôpital Henri Mondor, Créteil, France; ^2^Service de réanimation médicale et infectieuse, Hôpital Bichat-Claude Bernard-APHP, Paris, France; ^3^Réanimation médico-chirurgicale, Hôpital Tenon, Paris, France; ^4^Réanimation médicale, Hôpital Pitié-Salpêtrière, Paris, France; ^5^Réanimation médico-chirurgicale, Hôpital Louis-Mourier - APHP, Colombes, France; ^6^Service de Réanimation médicale et Médecine hyperbare, Centre Hospitalier Universitaire d’Angers, Angers, France; ^7^Réanimation Médicale, Hôpital Européen Georges-Pompidou (AP-HP), Paris, France; ^8^Réanimation polyvalente - surveillance continue, Ctre Hospitalier Intercommunal de Villeneuve Saint Georges, Lucie et Raymond AUBRAC, Villeneuve-Saint-Georges, France; ^9^Icu, C.H. de Longjumeau, Longjumeau, France; ^10^Réanimation Médicale, Hôpital Saint-Antoine, AP-HP, Paris, France; ^11^Service de Réanimation, Centre Hospitalier Marc Jacquet, Melun, France; ^12^Réanimation médicale, Hôpital Henri-Mondor (AP-HP), Créteil Cedex, France

####### **Correspondence:** Jerôme Cecchini - jerome.cecchini@aphp.fr

*Annals of Intensive Care* 2017, **7(Suppl 1)**:O47

**Introduction** Legionnaires’ disease (LD) is an important cause of community-acquired pneumonia. A high mortality rate has been reported in the most severe LD cases, for which the optimal antimicrobial regimen remains controversial.

**Patients and methods** This is a retrospective, multicentre observational study including patients admitted to ten intensive care units (ICU) for severe LD over a 10-year period. Patients were stratified according to the antibiotic strategy administered: (1) fluoroquinolones versus no fluoroquinolones and (2) monotherapy versus combination therapy. To evaluate the effect of antimicrobial strategy on ICU mortality, multivariable Cox model and propensity score analyses were used.

**Results** 211 patients with LD requiring ICU admission were included. A fluoroquinolone-based and a combination therapy were administered to 159 and 123 patients, respectively. 146 patients (69%) developed an acute respiratory distress syndrome, 111 (53%) a shock, 56 (27%) an acute renal failure that required renal replacement therapy, and 54 (26%) died in the ICU. In-ICU mortality was lower in the fluoroquinolone group as compared to the no-fluoroquinolone group (21 vs. 39%, p = 0.01), as well as in the combination therapy group as compared to the monotherapy group (20 vs. 34%, p = 0.02). In multivariable analysis with covariate adjustment including a propensity score for fluoroquinolone treatment, a fluoroquinolone-based therapy, but not a combination therapy, was independently associated with a reduced risk of mortality (HR 0.4, 95% CI [0.2–0.7], p = 0.002) (Table 7).

**Table 7:** Univariable and multivariable Cox model of factors associated with day-60 mortality in patients (n = 211) with severe Legionnaires’ disease admitted in the ICU

	Univariable analysis	Multivariable analysis
	HR (95% CI), p value	HR (95% CI), p value
Combination therapy		
No	1	I/NR
Yes	0.56 [0.32–0.97], p = 0.04	
Fluoroquinolone therapy		
No	1	1
Yes	0.38 [0.21–0.68], p = 0.001	0.39 (0.22–0.71), p = 0.002
Age > 60 years		
No	1	1
Yes	2.74 [1.37–5.50], p = 0.004	3.22 (1.49–6.97), p = 0.003
Smoking		
No	1	1
Yes	0.33 [0.17–0.64], p = 0.001	0.53 (0.26–1.08), p = 0.08
LODS (per point)	1.34 [1.23–1.46], p < 0.0001	1.40 (1.27–1.54), p < 0.0001
Propensity score^a^ (per point)	0.23 [0.09–0.57], p = 0.002	I/NR

*I/NR* included not retained, *LODS* Logistic Organ Dysfunction Score, *HR* hazard ratio

^a^Propensity score of fluoroquinolone-based treatment

**Discussion** Our results, which suggest a beneficial effect on mortality of a fluoroquinolone-based therapy in patients with LD who required ICU admission, are consistent with previous studies showing a non-significant trend in favor of fluoroquinolone use in patients hospitalized for LD [1]. The limited number of patients in our study precluded assessing the individual effects of distinct molecules within the fluoroquinolone class.

**Conclusion** Patients with severe LD receiving a fluoroquinolone-based antimicrobial regimen in the early course of the management in ICU had a lower in-ICU mortality, which persisted after adjusting on significant covariates. A combination therapy did not provide significant mortality benefit in the current study.

**Competing interests** None.

**Reference**

1. Burdet C, Lepeule R, Duval X, et al. Quinolones versus macrolides in the treatment of legionellosis: a systematic review and meta-analysis. J Antimicrob Chemother 2014;9:2354–60.

#### O48 Necrotizing pneumonia in ICU: clinical, microbiological and radiological characteristics

##### Simon Chauveau^1^, Nadège Wesner^1^, Laurence Monnier-Cholley^2^, Naïke Bigé^1^, Jeremie Joffre^3^, Eric Maury^4^, Hafid Ait-Oufella^1^, Bertrand Guidet^1^, Vincent Dubée^5^

###### ^1^Réanimation médicale, Hôpital Saint-Antoine, Paris, France; ^2^Radiologie, Hôpital Saint-Antoine, Paris, France; ^3^Service de reanimation médicale, Hôpital Saint-Antoine, Paris, France; ^4^Réanimation Médicale, Hôpital Saint-Antoine, AP-HP, Paris, France; ^5^Service de Réanimation Médicale, Hôpital Saint-Antoine, Paris, France

####### **Correspondence:** Vincent Dubée - vdubee@gmail.com

*Annals of Intensive Care* 2017, **7(Suppl 1)**:O48

**Introduction** Data on necrotizing pneumonia in ICU are scarce. This potentially devastating condition is generally thought to be due to highly virulent bacteria such as Panton-Valentine leukocidin-secreting *Staphylococcus aureus* or to slowly-growing organisms such as mycobacteria and other Actinomycetales. The goal of this study was to describe the clinical, microbiological and radiological features of NP in intensive care unit.

**Patients and methods** Monocenter restrospective study. All patients hospitalized between January 1st 2009 and July 1st 2016 with an ICD-10-CM code for pneumonia (J1X) in an 18-bed medical ICU were included. All chest X-rays and CT-scans obtained during the stay were screened in order to select patients with NP, which was defined as a new cavity or a zone with no contrast enhancement within a pulmonary consolidation. Diagnosis was confirmed by a senior radiologist. Medical records of included patients were reviewed to describe clinical and radiological presentation, demographic characteristics, microbiological results and outcome.

**Results** Among 1009 screened pts, a definitive diagnosis of NP was made in 37 pts (3.7%). Median age was 57 [interquartile range 48–69] years, 68% were males, median SAPS2 was 43. Half of the pts were immunocompromised due to hematologic malignancy (7 pts), solid tumor (7 pts), immunosuppressive therapy (2 pts) or HIV infection (2 pts). Eight pts had chronic respiratory disease, 8 had diabetes, 11 were alcoholic.

Pneumonia was community-acquired in 21 pts, including 3 pts with aspiration pneumonia. The remaining NP were nosocomial diseases, including 5 pts with ventilator-associated pneumonia. In 4 pts, pneumonia was considered to be acquired by hematogenous spread, including 1 patient with endocarditis.

Most pts had severe hypoxemia, with a median minimum PaO_2_/FiO_2_ ratio of 153 [102–203] mmHg. Twenty-three pts were intubated, and median duration of mechanical ventilation was 4 days. Eighteen pts developed septic shock, and 3 had renal replacement therapy. In-ICU mortality was 32% (12 pts).

Chest CT-scan was performed in all pts but 1. One or more cavity was observed in 33 pts, whereas lung necrosis without excavation was present in 4 pts. Most pts (25) had more than one lesion, involving more than 1 pulmonary lobe in 22 pts and both lungs in 15 pts. Median size of the largest lesion was 42 [24–78] mm.

Broncho-alveolar lavage was performed in 25 pts, and bronchial aspirate in 18 pts. One or more pathogen was isolated in 29 pts; in 4 pts, the final diagnosis was non-infectious (neoplasia in 3 pts and 1 crack lung), and all samples were sterile in 4 additional pts. Fifteen pts had polymicrobial infection. Overall, the most frequently isolated pathogen was *Pseudomonas aeruginosa* (9 pts), followed by *Staphylococcus aureus* (7 pts) and fungi (7 pts). *Klebsiella pneumoniae* and other Enterobacteriaceae were isolated in 5 and 6 pts, respectively. All cases involving *P. aeruginosa* were diagnosed in immunocompromised patients. In contrast, most patients with *S. aureus* infection (6/7) and all patients with *K. pneumoniae* infection were immunocompetent.

Among pts with proven infection, none underwent surgical treatment, but percutaneous drainage was performed in 3 pts.

**Conclusion** NP was rarely observed in our ICU. In immunocompromised patients, *P. aeruginosa* and fungi were the most frequently isolated pathogens, whereas highly virulent bacteria such as *S. aureus* and *K. pneumoniae* were more frequently encountered in immunocompetent patients.

**Competing interests** None.

#### O49 Epidemiology and long-term outcome after severe symmetric peripheral gangrene

##### Pierre Labroca^1^, Jérémie Lemarié^1^, Gérard Chiesa^2^, Isabelle Laroyenne^3^, Léo Borrini^4^, Rémi Klotz^5^, Quoc Phan Sy^6^, Marie-Christine Cristina^7^, Pierre-Edouard Bollaert^1^, Jean Paysant^8^

###### ^1^Réanimation médicale, hôpital central, C.H.U. de Nancy, Nancy, France; ^2^Service de rééducation et d’appareillage, Institut Robert Merle d’Aubigné, Valenton, France; ^3^Service de médecine physique et de réadaptation, Centre Medico-Chirurgical de Readaptation des Massues, Lyon, France; ^4^Service de médecine physique et de réadaptation, Hôpital d’Instruction des Armées Percy, Clamart, France; ^5^Service de médecine physique et de réadaptation, centre de médecine physique et de réadaptation de la tour de Gassies, Bruges, France; ^6^Service de médecine physique et de réadaptation, Hôpital la renaissance sanitaire, Villiers-Saint-Denis, France; ^7^Service de médecine physique et de réadaptation, Pôle Saint Hélier, Rennes, France; ^8^Centre louis pierquin, Institut Régional de Médecine Physique et de Réadaptation, Nancy, France

####### **Correspondence:** Jérémie Lemarié - j.lemarie@chru-nancy.fr

*Annals of Intensive Care* 2017, **7(Suppl 1)**:O49

**Introduction** Symmetric peripheral gangrene (SPG) is a rare but severe complication of septic shock often leading to multiple amputations. Epidemiology of SPG and long-term outcome remain poorly known. Amputations are serious offense to body integrity but can benefit from rehabilitation and prosthetics. Our objectives were to describe epidemiology of SPG and to assess health-related quality of life (HRQOL) once rehabilitation was achieved.

**Patients and methods** A prospective and retrospective, multicentric study was performed. Adult patients hospitalised between 2005 and 2015 were included. They must have undergone at least two major amputations (whatever the level) and been discharged in a specialised rehabilitation center. HRQOL was assessed with generic scale EQ-5D-3L by phone call. Epidemiologic data were extracted from hospitalization reports.

**Results** Nine centres on 13 participated, 30 patients were recruited and medical letters were available for 25 of them. SPR was observed in a majority of female (60%), aged around 50 yo. Mean intensive care unit (ICU) length of stay was 39 (± 21) days. Infectious agents were in majority Gram positive cocci (64%), but Escherichia Coli took an important part (20%), similar to Meningococcus (16%). All patients were amputated of the two lower limbs and 80% were quadruple amputees. HRQOL estimated wit EQ index was inferior to the French reference. However, patients rated themselves their health state as similar to the reference and even superior to the reference French value before SPG, using visual analogue scale (VAS). Main decrease in VAS was 22 points (95% CI 13–31). Intense pain due to phantom pain was the main factor of impaired EQ index. Painkillers use was statistically dependant of antidepressants use. All patient except one said they would be willing to be treated again for SPG.

**Conclusion** Symmetric peripheral gangrene is mainly due to Gram positive Cocci but also Escherichia Coli. It leads to severe amputations with impaired HRQOL which could be improved by better analgesic strategies. However patients consider themselves as being in good health and would be willing to be treated again. This should be taken into account before withdrawing life-sustaining therapies.

**Competing interests** None.

#### O50 Factors associated with positive valve culture in endocarditis and its impact on outcomes

##### Pierre Fillâtre^1^, Arnaud Gacouin^1^, Matthieu Revest^1^, Adel Maamar^1^, Pierre Tattevin^1^, Erwan Flecher^2^, Nicolas Lerolle^3^, Yves Le Tulzo^1^, Jean-Marc Tadié^1^

###### ^1^Service de maladies infectieuses et réanimation médicale, Centre Hospitalier Universitaire, Rennes, France; ^2^Service de chirurgie thoracique, cardiaque et vasculaire, Centre Hospitalier Universitaire, Rennes, France; ^3^Réanimation médicale, Centre Hospitalier Universitaire d’Angers, Angers, France

####### **Correspondence:** Pierre Fillâtre - pierre.fillatre@chu-rennes.fr

*Annals of Intensive Care* 2017, **7(Suppl 1)**:O50

**Introduction** Early surgical intervention in bacterial infective endocarditis seems to increase survival compared with conservative management or delayed surgery [1]. However, in order to decrease the bacterial inoculum on valves, European society of cardiology guidelines suggest, in cases without emergency, to postpone surgery to allow 1 or 2 weeks of antibiotic treatment [2]. We looked for valve culture in a population of endocarditis in intensive care unit and we aimed to identify associated factor for positive valve culture and to study outcomes of those patients.

**Patients and methods** Retrospective study between 2002 and 2016 for all consecutive adult patients who underwent cardiac surgery during the acute phase of definite left sided infectious endocarditis (Duke Criteria) in two referral centers and requiring intensive care unit (ICU) hospitalization. The following variables were recorded: demographic characteristics, echography findings before surgery (vegetation size, abscess…), cardiac timing surgery between the first adequate antimicrobial therapy and surgery and was classified as <7 days, 7–13 days and more than 14 days. Continuous variables were expressed as median and quartiles range and were compared by non-parametric tests (Mann–Whitney U-test). Proportions were compared by a χ^2^ test. A descendant stepwise logistic regression analysis was performed to determine the variables independently associated with positive valve culture. The study was approved by the Rennes institutional review board (N°12–70).

**Results** During the study period, 165 patients underwent cardiac surgery. Among them, 13 had endocarditis without microbiological documentation and 4 had no information about valve culture. Finally, 148 patients were included in the study with a median age of 65 years [53–73], a majority of men (74%) and obesity in 23 cases (16%). In most cases, it was a native valve endocarditis (n = 100, 68%) with a predominance of methicillin susceptible *Staphylococcus aureus* (n = 50, 34%) and a majority of acute endocarditis began less than 1 month (n = 102, 69%). In 95 cases (64%), the vegetation size was measured >10 mm and abscess was noted in 74 cases (50%). Time between adequate antimicrobial therapy and surgery was 14 days [5–26] and this latter was performed because of unstable hemodynamic condition in 102 cases (69%). Valve culture was positive in 46 cases (31%). Extra corporel circulation time was not significantly different in case of positive valve culture (120′ [97–173] vs 123′ [90–159]). There was only 2 cases of new surgery for valve disinsertion and 12 for tamponade.

The only factor independently associated with positive valve culture was the surgery performed in the week following the beginning of adequate antimicrobial therapy (OR 8.16, p < 0.001) and there was a strong tendency for larger vegetations >10 mm (OR 2.39 but p = 0.066). Performing surgery in the second week was not significantly associated with positive valve culture. In our study, positive valve culture was not associated with death or post-surgical complications except for acute respiratory distress syndrome (ARDS) which was more frequent in case of positive valve culture (37 vs 16%, p = 0.008). Overall in hospital mortality was 23%. The mortality of patients for which surgery was performed in the first week was 16/50 (32%) versus 18/96 (18%) but this tendency was not significant (p = 0.156).

**Discussion** Performing early surgery was independently associated with positive valve culture which may be explained by the too short time of antimicrobial therapy for reduction of bacterial inoculum. However, valve surgery in patients with positive valve culture was not associated with death or post-surgery complications except ARDS and clinicians must be aware of this risk.

**Conclusion** Our results suggest that valve cultures are more frequently positive when surgery is performed before 7 days of adequate antimicrobial therapy. We did not find that positive valve culture was associated with worst ICU outcomes except for ARDS.

**Competing interests** None.

**References**

1. Anantha Narayanan M, Mahfood Haddad T, Kalil AC, Kanmanthareddy A, Suri RM, Mansour G, et al. Early versus late surgical intervention or medical management for infective endocarditis: a systematic review and meta-analysis. Heart Br Card Soc. 2016;102(12):950–7.
2. Habib G, Lancellotti P, Antunes MJ, Bongiorni MG, Casalta J-P, Del Zotti F, et al. 2015 ESC Guidelines for the management of infective endocarditis: The Task Force for the Management of Infective Endocarditis of the European Society of Cardiology (ESC) Endorsed by: European Association for Cardio-Thoracic Surgery (EACTS), the European Association of Nuclear Medicine (EANM). Eur Heart J. 2015;36(44):3075–128.

#### O51 Time course of septic shock in immunocompromised and non-immunocompromised patients

##### Matthieu Jamme^1^, Fabrice Daviaud^1^, Julien Charpentier^1^, Nathalie Marin^1^, Yannick Hourmant^1^, Michael Thy^2^, Jean-Paul Mira^1^, Frédéric Pène^1^

###### ^1^Réanimation médicale, Hôpital Cochin, Paris, France; ^2^Réanimation médicale, Hopital Cochin, Paris, France

####### **Correspondence:** Matthieu Jamme - mat.jamme@gmail.com

*Annals of Intensive Care* 2017, **7(Suppl 1)**:O51

**Introduction** The outcome of septic shock has undoubtedly improved over the last two decades, both in immunocomptetent and immunocompromised patients. The combination of anti-infective treatments and aggressive organ failure supports often allows stabilisation of the clinical condition, but patients become then exposed to intensive care unit (ICU)-acquired infectious and non-infectious complications that significantly impact on prognosis. Whereas immunosuppression is commonly viewed as a major risk factor of death in septic shock, whether it is associated with an increased incidence of ICU-acquired complications is unclear. The present study aimed at addressing the course of septic shock across clinically significant subgroups of patients with and without immunosuppressive conditions.

**Patients and methods** This was a 8-year (2008–2015) monocenter retrospective study performed in a 24-bed medical ICU. All consecutive adult patients diagnosed for septic shock within the first 48 h of intensive care unit (ICU) admission were included. Septic shock was defined as a microbiologically proven or clinically suspected infection, associated with acute circulatory failure requiring vasopressors despite adequate fluid filling. Patients were considered either as non-immunocompromised or immunocompromised, this latter subgroup being categorized in three subgroups according to the underlying immunosuppressive condition: solid neoplasm, hematological malignancy and HIV- or drug-induced cellular immunosuppression. Furthermore, administration of intravenous chemotherapy during the last 3 months and leuconeutropenia defined by leucocyte count <1000/mm^3^ and/or neutrophil count <500/mm^3^ were collected as well. Survival status was assessed at ICU and hospital discharge. We focused on the most likely infectious, hemorrhagic and ischemic complications occurring after the first 48 h in the ICU.

The impact of the underlying immune status on 3-day and late mortality was assessed using a cause-specific proportional hazard model. To assess the impact of immune status on ICU complications, we performed two competing risk analysis model (Fine-Gray and cause-specific proportional hazard model). A landmark analysis with the choice of a 3-day fixed landmark point was performed to correct for the substantial immortal time bias. The SAPS2 was used to adjust for admission severity.

**Results** Eight hundred one patients were included. Their median age was 59 [57–79] years. The main source of infection was the lung in 49.6%. About 38% of patients had underlying immunosuppressive conditions, distributed into solid neoplasms (15.2%), hematological malignancy (13.2%) and non malignant immunosuppression (9.6%). The overall in-ICU and in-hospital mortality rates were 37.3% and 41.3%, respectively. With respect to the immune status, immunocompromised patients displayed worse outcome than immunocompetent ones, those with solid neoplasms having the highest risk of death. 113 patients died within the first 3 days, leaving 633 patients at risk of ICU-acquired complications. Among the 3-day survivors, the crude incidence rates of ICU-acquired infectious, ischemic and hemorrhagic complications were 27, 11 and 9%, respectively. The incidence of secondary infections was similar across the immunocompetent and the three immunocompromised subgroups of patients. In contrast, non malignant immunosuppression was associated with an increased risk of ischemic complications while hematological malignancies were associated with an increased risk of bleeding.

**Conclusion** We herein show that patients with septic shock display various clinical courses in relation with their underlying immune status. The burden of ICU-acquired complications in septic shock calls for every efforts of prevention and early detection, in order to improve the overall outcome.

**Competing interests** None.

#### O52 Epidemiology, treatment and outcome of patient with neutropenic enterocolitis admitted in ICU: a retrospective study

##### Baptiste Duceau^1^, Emmanuel Canet^1^, Virginie Lemiale^1^, Eric Mariotte^1^, Fanny Ardisson^1^, Valade Sandrine^1^, Marion Venot^1^, Benoît Schlemmer^1^, Elie Azoulay^1^, Lara Zafrani^1^

###### ^1^Réanimation médicale, Hôpital Saint-Louis (AP-HP), Paris, France

####### **Correspondence:** Baptiste Duceau - bduceau@gmail.com

*Annals of Intensive Care* 2017, **7(Suppl 1)**:O52

**Introduction** Neutropenic Enterocolitis (NE) occurs in about 5.3% [1] of patients hospitalized for hematological malignancies receiving chemotherapy. Data in critically ill patients with NE are scarce. The goal of this study is to describe the population of patient with NE admitted in ICU.

**Patients and methods** This retrospective study was performed in a 12-beds medical ICU between January 2010 and August 2016. All neutropenic patients hospitalized in the ICU with a diagnosis of enteritis and/or colitis were screened. Patients with a differential diagnosis (clostridium colitis, viral colitis, inflammatory enterocolitis, mesenteric ischemia, Graft-Versus-Host disease) were excluded. Univariate analysis between radiological findings, microbial documentation and outcomes were made with Fisher exact test (Software R v3.2.2).

**Results** We included 54 patients [male n = 34 (63%)], aged of 53 [31–63] years. The underlying malignancies were acute leukemia in 26 (48.1%) patients, lymphoma in 19 (35.2%) patients, and solid tumor cancer in 7 (12.9%) patients. Two patients had idiopathic medullar aplasia. Median SAPSII and SOFA scores at admission in ICU were respectively 53 [44–66] and 10 [8–12]. Main reason of admission was sepsis [n = 49 (91%)] with an increase in the Sepsis-related SOFA of 2 points or more for 51 (94%) patients. During ICU stay, 29 (54%) patients required mechanical ventilation, 37 (69%) patients required vasopressors and 13 (24%) patients required renal replacement therapy. Respective ICU and hospital mortality were 37 and 46.3%. Respective ICU and hospital median length-of-stay were 5.5 [3–15.75] and 39 [27–55] days.

NE occurred mainly after chemotherapy regimens containing cytarabine n = 36 (66.7%) and anthracyclines drugs n = 26 (48.1%). Symptoms appeared after a median of 8 [5–11] days after chemotherapy. Median duration of aplasia was 15.5 [10–22] days. Main symptoms consisted in diarrhea [n = 50 (92.6%)], abdominal pain [n = 47 (87%)], oral mucositis [n = 36 (66.7%)], paralytic ileus [n = 17 (31.5%)], nausea and vomiting [n = 14 (25.9%)]. Digestive hemorrhage occurred in 9 (16.7%) patients. Almost all patient had abdominal CT-scan (94.4%). The digestive inflammation involved ascending colon [n = 41 (75.9%)], transverse colon [n = 25 (46.3%)], descending colon or sigmoid [n = 25 (46.3%)], and small intestine [n = 12 (22.2%)]. A diffuse involvement (more than one segment) occurred in n = 32 (59.3%) patients. The other frequent signs were a peritoneal effusion n = 37 (68.5%) and intra-abdominal fat infiltration n = 19 (35.2%). Pneumoperitonea (n = 2) and parietal pneumatosis (n = 1) were rare.

Antibiotic therapy consisted in beta lactam (carbapenems [n = 31 (57.4%)], ureidopenicillins and inhibitors [n = 18 (33.3%)], cefepime [n = 4 (7.4%)], ceftazidime [n = 1 (1.9%)] combined with aminosids [n = 41 (75.9%)], vancomycin [n = 44 (81.5%)], and ornidazole [n = 34 (63%)]. Antifungal therapy was used for 33 (61.1%) patients. There was 33 (61%) bacterial and 8 (14.8%) fungal documented infections. Empirical antimicrobial therapy was effective in respectively 31 and 3 cases. Most frequent pathogens were *Escherichia coli* (n = 10), *Enterococcus faecium* n = 8, *Enterobacter cloacae* (n = 7), *Klebsiella pneumoniae* (n = 6), *Pseudomonas aeruginosa* (n = 3), *Candida spp* (n = 5). Infection was polymicrobial for n = 16 (29.6%). Involvement of small intestine including terminal ileum was associated with fungal infections (p = 0.01). ICU mortality was associated with fungal infections (p = 0.003) and polymicrobial infections (p = 0.03). Five patients underwent surgery (2 perforations, 1 digestive hemorrhage, 1 abscess, 1 negative laparotomy), with a ICU mortality of 60%.

**Conclusion** NE is a life threatening complication in patients with malignancies who receive intensive chemotherapy. NE is associated with high mortality rates, especially in patients with fungal infections. Antifungal therapy should be systematically discussed in NE patients with small intestine involvement.

**Competing interests** None.

**Reference**

1. Gorschlüter M, et al. Neutropenic enterocolitis in adults: systematic analysis of evidence quality. Eur J Haematol. 2005;75(1):1–13.

#### O53 Early infectious complications following heart transplantation

##### Stéphanie Pons^1^, Romain Sonneville^1^, Lenka Styfalova^2^, Lila Bouadma^1^, Mathilde Neuville^1^, Eric Mariotte^1^, Aguila Radjou^1^, Jordane Lebut^1^, Bruno Mourvillier^1^, Richard Dorent^3^, Marie-Pierre Dilly^4^, Patrick Nataf^5^, Michel Wolff^1^, Jean-François Timsit^1^

###### ^1^Réanimation médicale et infectieuse, Hôpital Bichat-Claude Bernard (AP-HP), Paris, France; ^2^Icuresearch, ICUREsearch, Paris, France; ^3^Cardiologie, Hôpital Bichat-Claude Bernard (AP-HP), Paris, France; ^4^Anesthésie réanimation chirurgie cardio-vasculaire, Hôpital Bichat-Claude Bernard (AP-HP), Paris, France; ^5^Chirurgie cardiaque et vasculaire, Hôpital Bichat-Claude Bernard (AP-HP), Paris, France

####### **Correspondence:** Stéphanie Pons - pons.stephanie0@gmail.com

*Annals of Intensive Care* 2017, **7(Suppl 1)**:O53

**Introduction** Heart transplantation is the reference treatment of end-stage heart failure. National priority heart transplantation for severe acute heart failure has increased, leading to a change in the population of heart-transplant recipients (older patients with more comorbidities). Infectious complication is one of the biggest concerns after solid organ transplantation, with high rates of morbidity and mortality. The impact of early infectious complications has never been assessed in a large cohort of ICU patients. We aimed to determine the characteristics, the determinants and the impact of infectious complications after heart transplantation.

**Patients and methods** We retrospectively studied all consecutive heart-transplant recipients from Bichat university hospital, between January 1st, 2011 and June 30th, 2015. All infectious complications that occurred within 6 months after transplantation were considered for analysis. The early post-operative period was defined as the first 8 days following heart transplantation. The primary endpoint was the rate of infectious complications at 6 months. We used multivariate logistic regression to identify independent risk factors for infections in the ICU. The impact of infectious complications on mortality at 3 months was determined by Cox regression analysis. Data are presented as median [interquartile range] or number (percentage).

**Results** One hundred and thirteen patients (53 years [40–62], male n = 86 (76.1%)) were included. At the time of heart transplantation, 65 (57.5%) patients were hospitalized in ICU for severe acute heart failure and 28 (24.8%) patients were under extracorporeal membrane oxygenation (ECMO) support. The SOFA score on the day before heart transplantation was 4 [1–6]. A cytomegalovirus (CMV) mismatch was found in 15 (14%) recipients. Twenty-two (19%) patients had a multidrug-resistant bacteria (MRB) carriage before transplantation, and six (5.3%) acquired one during the first week in the ICU. There were 213 infectious complications within 6 months after heart transplantation in 90 (80.5%) patients. The first bacterial infection occurred in ICU for 66 (58.4%) patients. Pneumonia was the most frequent infection, accounting for 46.5% of the cases. Sixty-five pneumonia were diagnosed during the ICU stay, and 46 out of 65 (70%) within the early post-operative period. By contrast, bloodstream infections (n = 31, 14.5%) and Scarpa infections (n = 22, 10.3%) were as frequent in the early post-operative period than after day eight. Sixty-three patients (55.8%) developed at least one infection due to Gram-negative bacilli, including 24 patients (21.2%) with an infection due to *Pseudomonas aeruginosa*. Thirty-five patients (31%) had a Gram positive cocci infection. Multi-resistant bacteria were responsible for infection in twenty-one (18.6%) patients. Within 6 months following heart transplantation, 44 (38.9%) patients had a viral complication, usually occurring after the early post-operative period and before day 30 [n = 32 (28.3%)]. Fungal infection was found in 16 (14.2%) patients, including seven invasive aspergillosis, most of them diagnosed after day 30. In univariate analysis, we found that ECMO following heart transplantation (n = 10 (29.4%) vs n = 62 (49%) p < 0.01), day 1 SOFA score (8 [7–10] vs 9.5 [8–11] p = 0.01) and mechanical ventilation duration (3 [1–8] vs 9 [5–15] p < 0.01) were associated with infectious complications in ICU. After adjustment, ECMO following heart transplantation was identified as the only independent risk factor for bacterial infection in the ICU (Odds Ratio: 3.1, 95% confidence interval 1.4–6.9, p = 0.006). Bacterial infection in ICU was associated with a longer stay in ICU (20 [13–29] days versus 12 [8–17] days, p < 0.001), but not with ICU mortality. Ninety-two patients (81.4%) were alive 6 months after the transplantation, and 11 out of the 21 deaths (52.3%) were caused by an infectious complication. In an adjusted Cox model, the third not-viral infection was significantly associated with death at 3 months post transplantation (adjusted Hazard Ratio: 6.2, 95% confidence interval 1.2–31, p = 0.02).

**Conclusion** This study confirms the high rate of early infectious complications after heart transplantation. ECMO following transplantation is an independent factor associated with bacterial infections. Bacterial infection in ICU was associated with a longer stay in ICU. The risk of death at 3 months after transplantation increased dramatically with the third episode of “not-viral infection”.

**Competing interests** None.

#### O54 Clinical course and prognosis of severe cryptococcosis in the intensive care unit: a retrospective multicenter study

##### Aëlle Le Gall^1^, Mathilde Neuville^2^, Simon Bourcier^3^, Julien Mayaux^4^, Damien Contou^5^, Yacine Tandjaoui-Lambiotte^6^, Vincent Das^7^, Benjamin Zuber^8^, Stéphane Gaudry^9^, Guillaume Voiriot^10^, Mikael Alves^11^, Eric Maury^1^, Naïke Bigé^1^

###### ^1^Réanimation médicale, Hôpital Saint-Antoine, Paris, France; ^2^Service de réanimation médicale et infectieuse, Hôpital Bichat-Claude Bernard-APHP, Paris, France; ^3^Réanimation médicale, Hôpital Cochin, Paris, France; ^4^Réanimation médicale, Hôpital Pitié-Salpêtrière, Paris, France; ^5^Réanimation Médicale, Hôpital Henri Mondor, Créteil, France; ^6^93, Hôpital Avicenne, Bobigny, France; ^7^Réanimation polyvalente adulte, Centre Hospitalier Intercommunal André Grégoire, Montreuil, France; ^8^Intensive care unit, Hospital Center De Versailles, Le Chesnay, France; ^9^Service de réanimation médico-chirurgicale, CHU Louis Mourier, Colombes, Colombes, France; ^10^Réanimation médico-chirurgicale, Hôpital Tenon, Paris, France; ^11^Réanimation médicale, C.H. Intercommunal Poissy/Saint-Germain-en-Laye, Poissy Cedex, France

####### **Correspondence:** Naïke Bigé - naikebige@gmail.com

*Annals of Intensive Care* 2017, **7(Suppl 1)**:O54

**Introduction** Cryptococcosis is a well-known opportunistic infection, especially in HIV-infected patients with profound immunosuppression. However, data regarding presentation and prognosis of severe forms of cryptococcosis requiring intensive care unit (ICU) admission are scarce.

**Patients and methods** We performed a retrospective multicenter study including all patients admitted for cryptococcosis from January 1998 to March 2016 to one of the 11 participating ICUs. Admissions were identified through a systematic review of ICUs databases using ICD-10 code B45. Diagnosis was confirmed either by cryptococcal antigen detection in serum, cerebrospinal fluid, bronchoalveolar lavage or urine, or by the identification of *Cryptococcus* in the culture of a specimen of any site or by histology. Qualitative and quantitative values are expressed as number and percentage, and median and interquartile range, respectively. Comparisons between ICU-survivors and –non survivors were performed using Fisher’s exact test and Mann–Whitney test for qualitative and quantitative variables, respectively. A p-value <0.05 was considered to be significant.

**Results** Seventy-three patients (age 44 [37; 52] years, 58 (79.5%) male, SAPS II: 42 [31; 59], SOFA score at day one: 4 [1;7]) were included. Twenty-five (34.2%) of them reported previous opportunistic infection. HIV infection was the leading cause of immunosuppression (n = 58 (79.5%), median CD4 count: 12 [6; 40]/mm^3^) and was newly diagnosed in 30 (51.7%) patients. The remaining patients broke as follows: immunosuppressive therapy for auto-immune disease (n = 6) or solid organ transplantation (n = 4) or nephrotic syndrome (n = 1), cirrhosis (n = 1), primary hypogammaglobulinemia (n = 2) and diabetes mellitus as the only underlying comorbidity (n = 1). Impaired consciousness (68.5%) was the first reason for ICU admission, followed by acute respiratory failure (21.9%), shock (8.2%) and acute renal failure (1.4%). Time from first symptoms to diagnosis was 7 [3; 21] days, and was 9 [4;25] days from first symptoms to ICU admission.

Central nervous system was the most frequently infected site [n = 62 (84.9%)] with meningo-encephalitis (n = 60 patients) and brain abscesses (n = 8). Lung was the second more frequent site of infection (n = 14). Other localisations included skin, heart, urinary tract, pleura and spleen. In 23 patients (31.5%), at least two sites were infected. Fungemia was observed in 17 patients (23.2%).

Invasive mechanical ventilation was required in 34 (46.6%) patients, vasopressors in 18 (24.7%) and renal replacement therapy in 10 (13.7%). Median ICU length of stay was 4 [3;12] days. ICU mortality was 41.1%.

Factors associated with ICU death were: infection of at least 2 sites (OR 5.1 [1.8; 15.2], p = 0.0038), SAPS II and SOFA score at day one (p < 0.0001), need for invasive mechanical ventilation (OR 18.9 [5.6;63.3], p < 0.0001), need for vasopressors (OR 41.3 [10.3;769.6], p < 0.0001) and renal replacement therapy (OR 7.5 [1.5;38.2], p = 0.013). Neither HIV status (p = 0.77) nor previous opportunistic infection (p = 0.80) was associated with ICU mortality.

**Discussion** Because of the limited number of non-HIV-infected patients, the study was not powerful enough to analyze clinical presentation according to HIV status.

**Conclusion** Severe forms of cryptococcosis requiring ICU admission are mostly observed among HIV-infected patients. However, one in five patients has another cause of immunosuppression, principally immunosuppressive therapy for auto-immune disease or solid organ transplantation. Central nervous system is the most common site infected. ICU mortality is high and associated with disseminated disease and need for organ supports. HIV status does not influence prognosis.

**Competing interests** None.

#### O55 Fungemia caused by Yarrowia lipolytica in ICU (about 55 cases)

##### Chtara Kamilia^1^, Ammar Rania^1^, Najeh Baccouch^2^, Kais Regaieg^3^, Olfa Turki^1^, Hmida Chokri Ben^1^, Mabrouk Bahloul^1^, Mounir Bouaziz^1^

###### ^1^Réanimation polyvalente, Faculté de médecine de Sfax, Sfax, Tunisia; ^2^Réanimation polyvalente, CHU Habib Bourguiba, Sfax, Tunisia; ^3^Reanimation polyvalente, CHU Habib Bourguiba, Sfax, Tunisia

####### **Correspondence:** Chtara Kamilia - kamilia.chtaraelaoud@gmail.com

*Annals of Intensive Care* 2017, **7(Suppl 1)**:O55

**Introduction** Rare opportunistic (non-Candida, non-Cryptococcus) yeast bloodstream infections (ROYBSIs) are rare, even in intensive care unit ICU. Candida lipolytica candidemia is a rare but an emerging pathogenic yeast infection in humans. It can gain access to the bloodstream through intravascular catheterization, especially through central venous catheters in immunocompromised or critically ill patients during hospitalization.

**Patients and methods** We retrospectively reviewed all episodes of unusual cases of Y. lipolytica fungemia occurred between October 2012 and June 2014 in the intensive care unit (ICU) of the UH Habib Bourguiba Sfax Tunisia. Underlying diseases, predisposing factors, laboratory data, and outcome were analyzed.

**Results** During this period, 55 cases of Y. lipolytica septicemia were diagnosed. There were 46 men and 9 women (sexratio = 5). The median age was 44.8 ± 19.8 years. The SAPS II was 40, 22 ± 12, 85 points. The catheterization (96%), mechanical ventilation (96%), the broadspectrum antibiotics (89%) and the prolonged hospitalization in ICU (78%) were the main risk factors. Patients were hospitalized in ICU, mostly, for polytraumatism (60%), acute respiratory failure (14.5%), and post-operative complications (9%). Fever unresponsive to broadspectrum antibacterial therapy was the predominant sign of infection (69%). Candida score was 1.85 ± 0.97 point. Pittet index was realized in 30 patients (54.5%). It was positive in 11 patients (36.6%). Preemptive treatment was indicated in 10 patients and was based on intravenous amphotericin B (50%) and fluconazole (50%).

The delay of fungemia was 18.73 ± 12.4 days. In our study, the most Y. lipolytica isolates are susceptible to fluconazole (73%) and amphotericin B (99%). The mortality rate in ICU was 45.5%.

Moreover, comparison between survivors and deceased showed that factors associated with deaths were: high SAPS II score on ICU admission, high SOFA score on the day of fungemia, a kidney failure and the presence of shock.

**Conclusion** In conclusion, although non-Candida fungemia is a rare clinical entity, it can develop in immunocompromised patients, and result in poor outcome.

**Competing interests** None.

#### O56 Septic shock in France from 2009 to 2014: incidence, outcome, and associated costs of care

##### Claire Dupuis^1^, Lila Bouadma^2^, Stephane Ruckly^3^, Anne Perozziello^3^, Bruno Mourvillier^4^, Sébastien Bailly^5^, Romain Sonneville^6^, Jean-François Timsit^7^

###### ^1^Réanimation médicale, CHU BICHAT, Paris, France; ^2^Réanimation médicale et des maladies infectieuses, Hôpital Bichat-Claude Bernard, Paris, France; ^3^Umr 1137, Faculté de Médecine Xavier Bichat, Paris, France; ^4^Réanimation Médicale et Infectieuse, GH Bichat Claude Bernard, Paris, France; ^5^Equipe 11, Institut Albert Bonniot - Inserm U823, La Tronche, France; ^6^Service de réanimation médicale et infectieuse, Hôpital Bichat-Claude Bernard-APHP, Paris, France; ^7^Réanimation médicale et infectieuse, Hôpital Bichat-Claude Bernard, Paris, France

####### **Correspondence:** Claire Dupuis - cdup83@gmail.com

*Annals of Intensive Care* 2017, **7(Suppl 1)**:O56

**Introduction** Despite decades of research and significant improvement in medical management, septic shock remains a major health care burden and a leading cause of death worldwide. The aim of this study is to determine recent national trends in occurrence of septic shock (SC) and SC-related deaths and costs.

**Patients and methods** We analysed the occurrence of sepsis from 2009 to 2014, in adult patients, using the national French hospital Database PMSI (Programme de Médicalisation des Systèmes d’Information). This database collects annually, for each hospital stay in France, a uniform hospital discharge record including diagnostic codes according to the International Classification of diseases, Tenth revision, Clinical Modification (ICD-10th-CM) and procedure codes according to the CCAM (Classification Commune des Actes Médicaux).

Cases were identified from discharge records in the PMSI database. Septic shock was defined by a combination of a principal or accompanying diagnosis of infection (ICD-10thCM) plus a diagnosis code for septic shock (R572, R578, R579) or the procedure code EQLAFF03 (infusion of vasopressors). Charlson index were determined using diagnosis codes as previously described [1].

Costs were determined according to the National Reference Costs (ENC = Etude Nationale des Couts). Temporal trend were assessed by calculating the value of the entire variable by year and compared using linear regression variables or Cochran Armitage Trend Test for continuous and categorical variables respectively.

**Results** A total of 25,444,627 adults aged ≥ 18 years were hospitalized in France during 2009–2014. Among them we identified 419,597 (10.3%) patients with SC. There was an annualized increase in the incidence of SC from 87 to 122/100,000 inhabitants p < 0.001). Only 25% of the cases were hospitalized in an affiliated university centre. Sepsis was more frequent among men (62.8%) than among women (37.2%). Overall the SAPS2 score was (median [interquartile range = IQR] 49 [36–65]. Charlson comorbidities index decreased significantly over time (p < 0.001). 58.6% of the cases were considered as medical. Nearly all the patients were admitted from the emergency department (75.9%) or from wards (20.1%). About 80% of the patients were hospitalized at least 1 day in Intensive Care Units and of note this percentage decreased consistently over time from 85.6 to 79.7%. Concomitantly admission at least 1 day in intermediate care unit (from 24.9% to 33.6%) or in specialized wards such as onco-hematology (from 0.6% to 3.1%) increased over time. Overall, the main comorbidities reported were congestive heart failure (27.6%), cerebro-vascular disease (8.7%), Chronic Pulmonary Disease (15%), dementia (2.9%), renal disease (11.3%), diabetes without (6.3%) and with complications (5%) and cancer (16.9%), mild liver disease (8.5%). The main sources of sepsis were pulmonary (39.2%), abdominal (12%), urinary (15.6%), and cutaneous (3.2%), cardiac (3.1%), neurological (1.1%), and osteo-articular (1.6%). Gram-negative bacteria were the predominant organisms causing sepsis (from 27.6% to 38.8%) but gram-positive bacteria were reported most commonly in each subsequent year as well (from 20.8% to 26.4%). The length of ICU and hospital stays were 9 [3–19] and 18 [8–35] days respectively and both decreased over time (p < 0.001). Mortality rates slightly decreased over the 6-year period (from 41.7% to 38.7%). However the in-hospital deaths related to sepsis increased over time from 433 to 571/10,000 deaths in France (p < 0.001). The average costs per case were 14,453€ [7460–25,041] decreasing over time from 15,291 [7637–26,136] to 14,171 [7593–24,194].

**Conclusion** SC incidence increased in France. Nearly 75% of the cases were managed in non-affiliated university centre. Although the overall mortality rates in patients with SC is slightly decreasing in France, the number of deaths related to SC in France is growing.

Results must be interpreted with caution because of the limits related to administrative databases. However, the trends are comparable to data from other countries.

**Competing interests** None.

**Reference**

1. Quan H, Sundararajan V, Halfon P, Fong A, Burnand B, Luthi J-C, et al. Coding algorithms for defining comorbidities in ICD-9-CM and ICD-10 administrative data. Med Care. 2005;43(11):1130–9.

#### O57 Severe complications of Zika virus infection during the 2016 outbreak in Guadeloupe

##### Julien Letheulle^1^, Marc Valette^1^, Cécile Herrmann-Storck^2^, Laura Crosby^1^, Khalid Elkoun^1^, Benjamin Madeux^1^, Frédéric Martino^1^, Hélène Migueres^1^, Pascale Piednoir^1^, Bertrand Pons^1^, Matthias Posch^1^, Guillaume Thiery^1^

###### ^1^Intensive care unit, Hospital Center Regional University, Pointe-a-Pitre, Guadeloupe; ^2^Virology, Hospital Center Regional University, Pointe-a-Pitre, Guadeloupe

####### **Correspondence:** Julien Letheulle - julienletheulle@yahoo.fr

*Annals of Intensive Care* 2017, **7(Suppl 1)**:O57

**Introduction** Since 2015, Zika virus (ZIKV) infection is spreading across South and Central America. The outbreak reached the Caribbean in the early 2016. The aim of this study is to describe the characteristics and outcome of patients with severe complications of ZIKV requiring intensive care unit (ICU) admission.

**Patients and methods** We performed a prospective observational study in the ICU in the University Hospital of Guadeloupe from May 15th to September 15th 2016. All patients with an acute ZIKV infection confirmed either by Real Time PCR (RT-PCR) or IgM detection in the serum were included. In patients presenting with an acute neurologic disease, extensive laboratory testing, CSF analysis, brain MRI and electromyography testing were performed according to a predefined protocol, in order to rule out others diagnosis. Patients with Guillain-Barré syndrome (GBS) were treated with intravenous immunoglobulins 0.4 mg/kg/day during 5 days.

Qualitative data are presented in percentage and quantitative data in medial [interquartile range].

**Results** During the study period, 19 patients with proven ZIKV infection were admitted to the ICU: 10 (53%) men, median age 59 [37–73] years, SAPS-2 30 [21–37]. Main comorbidities were chronic hypertension (47%) and diabetes (32%). One patient (5%) was immunocompromised and 6 (32%) had no comorbidities.

Among the 19 patients, 15 (79%) were admitted for an acute neurological disease, namely GBS in 10 (53%) patients and encephalitis in 5 (26%) patients. The remaining 4 (21%) patients were admitted for COPD exacerbation, lupus exacerbation, myasthenia gravis and metformine intoxication complicated with auto-immune hepatitis.

Clinical symptoms of ZIKV infection such as fever, arthralgia, cutaneous rash, conjunctivitis were identified in 11 (58%) patients. The time between symptoms onset and ICU admission was 7 [5–10] days. Among the 15 patients with neurological diseases, the time between the onset of neurological symptoms and ICU admission was 3 [2–5] days. ZIVK RT-PCR was positive in urine in 15 (79%) patients, in blood in 2 (11%) patients and in CSF in 1 (5%) patient. Serum IgM were detected in 10 (53%) patients.

CSF was obtained in the 15 patients with neurological diseases. For the 10 patients with GBS, white cell count was 3 [1–5]/mm^3^, protein level was 1.4 [0.8–1.9] g/L, glucose level 4.8 [4.3–5.3] mmol/l. For the 5 patients with encephalitis, white cell count was 65 [24–180]/mm^3^, protein level was 0.6 [0.4–1.1] g/L, glucose level 5.5 [4.1–6.1] mmol/l.

Brain MRI abnormalities were observed in 4 (21%) patients, and myelitis was observed in one patient.

Fourteen patients (79%) had mechanically ventilation for median length of 12 [6–22] days. Among them 4 (21%) were tracheotomised because of prolonged mechanical ventilation. Six (32%) patients received vaso-active drugs, and 2 (11%) required renal replacement therapy. ICU length of stay was 12 [7–25] days. Five (26%) patients stayed more than 30 days in the ICU: 3 patients with GBS, 1 patient with encephalitis, 1 other patient. Three (16%) patients died in the ICU.

**Conclusion** As previously reported, ZIKV infection is responsible for neurological complications [1]. In particular, the GBS has been observed in a previous epidemic in French Polynesia [2]. Our data confirmed the association between ZIKV infection and GBS, as the annual incidence of GBS in our ICU is usually 3–5.

Interestingly, several cases of encephalitis were observed, which has not been reported previously.

These are preliminary data. As the outbreak is still ongoing in the Caribbean, we can expect more cases and more precise data within the next months.

**Competing interests** None.

**References**

1. Broutet N, Krauer F, Riesen M, et al. Zika virus as a cause of neurologic disorders. N Engl J Med. 2016;374(16):1506–9.
2. Cao-Lormeau VM, Blake A, Mons S, et al. Guillain-Barré Syndrome outbreak associated with Zika virus infection in French Polynesia: a case–control study. Lancet 2016;387:1531–9.

#### O59 Closed-loop adjustment of oxygen flowrate with FreeO_2_ in patients with acute coronary syndrome, comparison of two SpO_2_ target and manual adjustment: a randomized controlled study

##### Minh-Tu Huynh-Ky^1^, Pierre Alexandre Bouchard^1^, Erwan L’Her^2^, Jean-François Sarrazin^1^, François Lellouche^1^

###### ^1^Centre de recherche de l’iucpq, Institut Universitaire de Cardiologie et de Pneumologie de Québec, Québec, Canada; ^2^Réanimation médicale, CHRU de Brest, Brest, France

####### **Correspondence:** François Lellouche - francois.lellouche@criucpq.ulaval.ca

*Annals of Intensive Care* 2017, **7(Suppl 1)**:O59

**Introduction** Supplemental oxygen has been used in the management of patients with acute coronary disease and investigated for more than a century [1]. The rational for a systematic oxygen supply in patients with acute coronary syndrome is based on a limited number of data. The links between hypoxia and ischemic or arrhythmic electrocardiographic abnormalities have been known for a long time. The risks associated with hyperoxia have been well described in physiological studies (increased coronary resistances, decreased coronary blood flow). A recent large RCT demonstrated that liberal oxygen administration during the acute phase of myocardial infarction may increase the level of cardiac enzyme and the infarct size [2].

FreeO_2_ is a recently developed device that automatically titrates oxygen flowrate with the aim to maintain constant the SpO_2_, around the target set by the physician. We evaluated two SpO_2_ targets using automated oxygen titration with FreeO_2_ during acute coronary syndrome and evaluated oxygenation delivered with usual manual oxygen therapy. Our hypotheses were that high rates of hypoxemia and hyperoxia would occur with manual titration, and would be reduced with automated oxygen titration.

**Patients and methods** We conducted a pilot randomized controlled, single blind monocentric study to evaluate oxygen therapy administration at the acute phase of acute coronary syndrome. Patients with acute coronary syndrome were included (based on AHA criteria). Severe COPD patients were excluded. Patients were randomized one of the three arms: Automated oxygen titration with FreeO_2_ at two different SpO_2_ targets (92 and 96%) and manual administration of oxygen.

The study lasted a maximum of 24 h for each patient, including one night. All patients were continuously monitored with FreeO_2_ (set in recording mode in the manual group), continuous cardiac telemetry was performed for all patients, cardiac enzymes were collected as per usual care.

The primary outcome was the frequency of desaturation (SpO_2_ < 90% for 30 s). The secondary outcomes were the frequency of arrhythmias, the rate of tachycardia episodes and the level of cardiac enzymes in patients with acute coronary disease.

**Results** Sixty patients were included in the study, the mean age was 63 ± 12 years, 73% of the patients were men. The average recording time was 11.5 ± 2.8 h. Preliminary data are presented here.

–21 patients were included in the control group (manual adjustment of oxygen therapy).

–20 patients were included in the FreeO_2_ group with SpO_2_ target = 92%.

–19 patients were included in the FreeO_2_ group with SpO_2_ target = 97% (including one case of missing data due to technical issues).

Primary end point (rate of hypoxemia): The percentage of time with hypoxemia (SpO_2_ < 90%) was 4% of the recording time (equivalent to 30 min) in the control group, 1.2% of the recording time (equivalent to 9 min) in the FreeO_2_ (92%) group and 0.4% of the recording time (equivalent to 2.5 min) in the FreeO_2_ (97%) group (manual vs. 92%, p = 0.58; manual vs. 97%, p = 0.001; 92 vs. 97%, p = 0.006).

57% of the patients in the control group, 30% in the FreeO_2_ (92%) and 22% in the FreeO_2_ (97%) experienced desaturation below 90% for more than 30 s (manual vs. 92%, p = 0.08, manual vs. 97%, p = 0.027).

The rate of severe hypoxemia (SpO_2_ < 80 and 85%) were reduced with FreeO_2_ (97%).

The rates of ventricular extrasystoles were 43, 35, 6%, p < 0.001 and atrial extrasystoles were 28, 20 and 17%, p = 0.15 for manual titration, FreeO_2_ (92%) and FreeO_2_ (97%) respectively.

The mean maximal heart rate was 96 ± 15, 86 ± 10 and 82 ± 13, p = 0.018 for manual titration, FreeO_2_ (92%) and FreeO_2_ (97%) respectively.

There was no differences in hospital length of stay between the groups and the mortality was 0% in all groups. Other data are under analysis.

**Conclusion** Automated oxygen titration with FreeO_2_ with SpO_2_ target set at 97% reduced the rate of desaturation and severe desaturation in patients managed for acute coronary syndrome.

Automated oxygen titration with FreeO_2_ with SpO_2_ target set at 97% reduced the rate of ventricular extrasystoles.

SpO_2_ target set at 92% with FreeO_2_ may not be sufficient in this population.

With manual titration the risk of hypoxemia and severe hypoxemia was increased, however, the rate of significant hyperoxia was not increased in this study and could not be evaluated.

Additional data are required to evaluate intermediate levels of oxygenation target and potential impact in this population.

**Competing interests** Co-founder of Oxynov company that develops the FreeO_2_ system.

**References**

1. Steele C. Severe angina pectoris relieved by oxygen inhalations. BMJ. 1900;2:1568
2. Stub D, et al. Air versus oxygen in ST-segment-elevation myocardial infarction. Circulation. 2015;131(24):2143–50.

#### O60 Changes in end-tidal carbon dioxide as a surrogate for cardiac output changes outperform heart rate-, blood pressure- and femoral Doppler-derived indices during fluid challenge

##### Karim Lakhal^1^, Mai-Anh Nay^2^, Toufik Kamel^2^, Brice Lortat-Jacob^3^, Stephan Ehrmann^4^, Bertrand Rozec^5^, Thierry Boulain^2^

###### ^1^Service de réanimation chirurgicale, Hôpital Guillaume et René Laënnec, CHU de Nantes, Nantes, France; ^2^Réanimation médicale polyvalente, Hôpital de La Source, CHR Orléans, Orléans, France; ^3^Réanimation chirurgicale polyvalente, Hôpital Bichat-Claude Bernard (AP-HP), Paris, France; ^4^Réanimation polyvalente, CHRU Hôpitaux de Tours, Tours, France; ^5^Réanimation CTCV Transplantation thoracique, CHU de Nantes - Hôpital Nord Laennec, Saint-Herblain, France

####### **Correspondence:** Mai-Anh Nay - vannavy@free.fr

*Annals of Intensive Care* 2017, **7(Suppl 1)**:O60

**Introduction** During acute circulatory failure, volume expansion (VE) aims at increasing cardiac output (CO). However, CO is seldom measured to manage VE [1]. Increase in systolic, mean and pulse arterial blood pressure (ΔveSBP, ΔveMBP and ΔvePP) or decrease in heart rate (ΔveHR) are often used as surrogates for VE-induced increase in CO (ΔveCO) despite their poor performance.

VE-induced changes in end-tidal carbon dioxide (ΔveEtCO_2_) could be a surrogate for ΔveCO. Indeed, EtCO_2_, the amount of exhaled carbon dioxide (CO_2_), depends on CO_2_ production by body tissues, its delivery by CO and its elimination by alveolar ventilation. If, during VE, alveolar ventilation is kept unchanged, i.e., during fully controlled ventilation, and if we hypothesize that CO_2_ production only mildly changes, then ΔveEtCO_2_ may reflect ΔveCO [2].

Other appealing surrogates were also scarcely evaluated: Doppler measurements of VE-induced increase in femoral artery flow (ΔveFemFlow) or, provided the absence of inspiratory efforts and arrhythmia, VE-induced decrease in respiratory pulse pressure variation (ΔvePPV).

The objective of this study was to compare ΔveEtCO_2_, ΔveFemFlow, ΔveSBP, ΔveMBP, ΔvePP and, when applicable, ΔvePPV and ΔveHR as surrogates for ΔveCO for the identification of patients who responded/did not respond to VE.

**Patients and methods** Adult patients were prospectively included if (1) they already had an arterial line, (2) they were receiving controlled mechanical ventilation, (3) their blood pressure (BP) was stable over 5 min, (4) the attending physician prescribed a VE and (5) presence of at least one criterion suggesting acute circulatory failure: hypotension (invasive systolic BP < 90 mmHg and/or mean BP < 65 mmHg), oliguria (<0.5 ml kg h^−1^) considered to be related to circulatory failure, arterial lactate >2.5 mmol L^−1^, skin mottling, or vasopressor and/or inotropic drug infusion.

CO was measured by trans-thoracic echocardiography and was the average of 2 sets of 3 (5 in case of arrhythmia) consecutive measurements started from the velocity time interval of highest magnitude. EtCO_2_, displayed on the ventilator, was collected at a glance, once at each study phase.

Femoral velocity time interval was measured with a 5-MHz linear echographic probe to quantify flow. Other definitions were tested for femoral flow: magnitude (peak) of the femoral pulse wave.

The ability to identify patients responding or not to VE (ΔveCO ≥ 15%) was evaluated by calculation of the areas under the receiver operating characteristic curves (AUCROCs).

**Results** In 109 patients included, poor thoracic insonation prevented CO measurements in 22 (20%). One patient was excluded because of change in minute ventilation >0.2 L/min during the study protocol.

In the 86 remaining patients, the AUCROC for ΔveEtCO_2_ was 0.82 [0.73–0.90], significantly higher than for ΔvePP, ΔveSBP, ΔveMBP and ΔveFemFlow, whatever its definition (AUCROC 0.61–0.65, all p < 0.05). A ΔveEtCO_2_ > 1 mmHg had good positive (5.0 [2.6–9.8]) and fair negative (0.29 [0.2–0.5]) likelihood ratios. Arrhythmia was of little impact on the reliability of ΔveEtCO_2_: the 16 patients with arrhythmia had similar relationship between ΔveEtCO_2_ and ΔveCO than regular rhythm patients (r^2^ = 0.23 in both subgroups).

In 60 patients with no arrhythmia, ΔveEtCO_2_ (AUCROC = 0.84 [0.72–0.92]) outperformed ΔveHR (AUCROC = 0.52 [0.39–0.66], p < 0.05) and tended to outperform ΔvePPV (AUCROC = 0.73 [0.60–0.84], p = 0.21). In the 45 patients with no arrhythmia and receiving a tidal volume <8 ml/kg of ideal body weight, ΔveEtCO_2_ was of significantly better performance than ΔvePPV: AUCROC = 0.86 [0.72–0.95] vs. 0.66 [0.49–0.80], p = 0.02.

**Conclusion** ΔveEtCO_2_ outperformed ΔvePP, ΔveSBP, ΔveMBP, ΔveFemFlow and ΔveHR, and, in case of protective ventilation and/or arrhythmia, also outperformed ΔvePPV. ΔVEEtCO_2_ > 1 mmHg indicates that the patient is very likely to have responded to VE.

**Competing interests** None.

**References**

1. Boulain T, Boisrame-Helms J, Ehrmann S, Lascarrou JB, Bougle A, Chiche A, Lakhal K, Gaudry S, Perbet S, Desachy A, Cabasson S, Geneau I, Courouble P, Clavieras N, Massanet PL, Bellec F, Falquet Y, Reminiac F, Vignon P, Dequin PF, Meziani F. Volume expansion in the first 4 days of shock: a prospective multicentre study in 19 French intensive care units. Intensive Care Med. 2015;41:248–56
2. Jacquet-Lagreze M, Baudin F, David JS, Fellahi JL, Hu PB, Lilot M, Piriou V. End-tidal carbon dioxide variation after a 100- and a 500-ml fluid challenge to assess fluid responsiveness. Ann Intensive Care. 2016;6:37

#### O61 Intra-abdominal hypertension among critically ill patients requiring extracorporeal life support for refractory cardiac arrest

##### Marion Colnot^1^, François Belon^1^, Nicolas Belin^1^, Loïc Barrot^1^, Jean-Christophe Navellou^1^, Cyrille Patry^1^, Guylaine Labro^1^, Claire Chaignat^1^, Melanie Claveau^1^, Frédéric Claude^1^, Gilles Capellier^1^, Gaël Piton^1^

###### ^1^Réanimation médicale, CHU de Besançon, Besançon, France

####### **Correspondence:** Marion Colnot - marion.colnot@hotmail.fr

*Annals of Intensive Care* 2017, **7(Suppl 1)**:O61

**Introduction** Patient admitted to the ICU for refractory cardiac arrest can currently be managed using peripheral veno-arterial extracorporeal life support (ECLS) [1]. Because of impaired venous in-flow, occurrence of intra-abdominal hypertension (IAH), that is, intra-vesical pressure (IVP) above 12 mmHg), can compromise ECLS efficiency [2]. We hypothesized that prompt diagnosis and treatment of IAH among patients under ECLS for refractory cardiac arrest may improve ECLS performance and clinical outcome. We aimed to describe critically ill patients requiring ECLS for refractory cardiac arrest and presenting with IAH.

**Patients and methods** This was a retrospective study of patients admitted to the ICU of the Besançon University Hospital, between January 2010 and August 2016, who develop IAH under peripheral veno-arterial ECLS for refractory cardiac arrest. We collected data about diagnosis and treatment of IAH, ECLS performance, and patient outcome. Descriptive analysis was performed. Quantitative variables were expressed as median [interquartile range] and qualitative variables as number (percentage).

**Results** During the study period, 202 patients (4% of the patients) were admitted to our ICU with femoral veno-arterial ECLS. Indication of ECLS was refractory cardiac arrest in 97 patients (48%), and among them, 28 (29%) developed IAH. Data were available for 27 patients who were included in the analysis. Maximum IVP was 22 [17–25.5] mmHg, which was reached 12 [5–19.5] h after ECLS onset. IVP was above 20 mmHg in 56% of cases. Curarization was initiated in 22 patients (81%). Ten patients (45%) required additional rescue laparotomy which was performed 2.5 [2–5] h after the diagnosis of abdominal compartment syndrome. ECLS parameters during maximum IAH and their improvement after efficient treatment were presented in Fig. 8. Clinically, there was a transient improvement of hemodynamic status after treatment, but hepatic and renal failures persisted. In our cohort, in-ICU mortality reached 100%. Deaths occurred after a median duration of ECLS of 32 [12–69] h, and because of refractory multi-organ failure in 82% of cases.

**Fig. 8:**
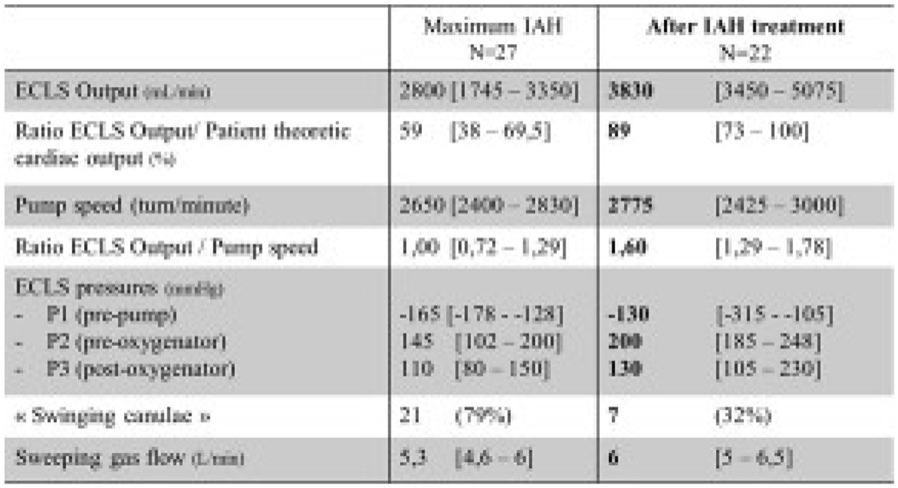
Comparison of ECLS parameters during IAH and after efficient treatment of IAH

**Conclusion** Among patients under ECLS for refractory cardiac arrest, IAH is frequent, occurs early, and is associated with an extremely poor prognosis. IAH treatment is associated with an improvement of ECLS outflow and hemodynamic status. IAH among patients presenting with refractory cardiac arrest might be a strong prognostic factor *per se*. It’s not excluded that early and efficient treatment of IAH, before occurrence of abdominal compartment syndrome, might improve the overall prognosis of critically ill patients under ECLS for cardiac arrest.

**Competing interests** None.

**References**

1. Shekar et al. Mechanical circulatory support in the new era: an overview. Crit Care 2016;20:66.
2. Maj et al. Abdominal compartment syndrome during extracorporeal membrane oxygenation. J Cardiothorac Vasc Anesth. 2012;26(5):890–2.

#### O63 Low-dose heparin versus full systemic anticoagulation in critically ill patients undergoing extracorporeal membrane oxygenation: the HELP-ECMO pilot randomised controlled study

##### Cécile Aubron^1^, Zoe Mcquilten^2^, Michael Bailey^3^, Jasmin Board^4^, Heidi Buhr^5^, Bruce Cartwright^6^, Mark Dennis^7^, Paul Forrest^6^, Carol Hodgson^4^, David Mcilroy^8^, Deirdre Murphy^4^, Lynnette Murray^3^, Vincent Pellegrino^4^, David Pilcher^4^, Jayne Sheldrake^4^, Huyen Tran^9^, Shirley Vallance^4^, Jamie Cooper^3^

###### ^1^Réanimation médicale, Bd Tanguy Prigent, Brest, France; ^2^Depm, monash university, Transfusion Research Unit, Melbourne, Australia; ^3^Depm, monash university, ANZIC-RC, Melbourne, Australia; ^4^Intensive care unit, Alfred Hospital, Melbourne, Australia; ^5^Intensive care unit, Royal Prince Alfred Hospital, Sydney, Australia; ^6^Intensive care, Royal Prince Alfred Hospital, Sydney, Australia; ^7^Intensive care unit, Royal Prince Alfred Hospital, Camperdown, Australia; ^8^Anesthesiology, Alfred Hospital, Prahran, Australia; ^9^Haematology, Alfred Hospital, Prahran, Australia

####### **Correspondence:** Cécile Aubron - cecile.aubron@chu-brest.fr

*Annals of Intensive Care* 2017, **7(Suppl 1)**:O63

**Introduction** Variable intensity systemic anticoagulation with unfractionated heparin is routinely used in patients undergoing extra corporeal membrane oxygenation (ECMO) to offset the increase risk of thrombosis in this population. Bleeding remains the most frequent complications and is independently associated with worse outcomes. Therefore, determining optimal anticoagulation to prevent thrombosis whilst minimising bleeding in adults on ECMO is a priority. This pilot study aims to evaluate the feasibility of allocating patients on ECMO in two different anticoagulation patterns group resulting in a difference in anti-coagulation level.

**Patients and methods** This is a randomised, controlled, unblinded pilot trial at two intensive care units. We enrolled critically ill patients who required ECMO (venous–venous [VV] or venous-arterial [VA]). We randomly assigned patients to therapeutic anticoagulation with heparin (target activated partial thromboplastin time [aPTT] between 50 and 70 s) or low dose heparin (12,000 units/24 h aiming for aPTT <45 s). Paired aPTT and anti-Xa assays were taken at a minimum once a day. The primary endpoints for feasibility were difference in mean heparin dose, aPTT and anti-Xa levels. All primary outcomes were log-transformed prior to analysis and reported as geometric means (95% CI) with overall differences determined using Repeat measures ANOVA.

**Results** Between May 2014 and March 2016, 31 patients who underwent ECMO (9 VA and 22 VV) were enrolled; 16 were randomised to low dose and 15 to therapeutic dose heparin. The groups were similar in age (mean 41 years [SD 16.8] vs 43 [SD 17.6] p = 0.75), gender (68 vs 80% male, p = 0.47), type of ECMO (31 vs 27% VA, p = 0.78) and severity sepsis-related organ failure assessment score (mean 10 [SD 3.6] vs 10 [SD 3.3], p = 1.0). The mean duration of ECMO support was 9.33 days (SD 5.97) in the low dose and 9.79 days (SD 4.77) in the therapeutic dose group (p = 0.82). For the primary outcomes, there was a significant difference in the daily mean aPTT (48.1 [95% CI 43.5–53.3] vs 56.2 [95% CI 50.7–62.3], p = 0.03), daily mean anti-Xa (0.11 [95% CI 0.07–0.18] vs 0.30 [IQR 0.19–0.46], p = 0.003) and daily mean heparin dose (11,784 units [95% CI 8693–15,972] vs 22,050 [IQR 16,262–29,899], p = 0.004) in the low dose compared to therapeutic group. There was no difference in thrombotic and bleeding complications between study groups.

**Conclusion** In this pilot trial, administration of a low dose heparin protocol was feasible, and resulted in a significant difference in mean heparin dose administered and daily aPTT and anti-Xa levels between groups. Our findings support the feasibility of a larger study to evaluate the safety and efficacy of low-dose anticoagulation compared with therapeutic heparin with regard to thrombotic and bleeding events in patients receiving ECMO.

**Competing interests** None.

#### O64 Ventilator-associated pneumonia in patients assisted by veno-arterial extracorporeal membrane oxygenation: epidemiology and risk factors of treatment failure

##### Camille Bombled^1^, Adrien Bouglé^1^, Marine Coroir^1^, Charles Vidal^1^, Dimitri Margetis^1^, Guillaume Lebreton^2^, Julien Amour^1^

###### ^1^Département d’anesthésie et de réanimation, hôpital universitaire pitié salpêtrière, Université Pierre et Marie Curie, UMRS INSERM 1166, IHU ICAN, Paris, France; ^2^Service de chirurgie thoracique et cardiovasculaire, Hôpital Universitaire Pitié Salpêtrière, Université Pierre et Marie Curie, Paris, France

####### **Correspondence:** Camille Bombled - cam.bombled@gmail.com

*Annals of Intensive Care* 2017, **7(Suppl 1)**:O64

**Introduction** Ventilator-associated pneumonia (VAP) is a frequent complication in Intensive Care Unit (ICU) patients.

In the specific case of patients treated with Veno-Arterial Extracorporeal Life Support (VA-ECLS), epidemiology and risk factor of VAP treatment failure (VAP-TF) have been incompletely investigated. The objective of this study was to describe the epidemiology of VAP and investigate the risk factors of treatment failure (VAP-TF) in a large cohort of ICU patients treated with VA-ECLS.

**Patients and methods** All patients treated with a VA-ECLS between January 2013 and December 2014 were systematically included in the SARIC database. We retrospectively investigated all patients who developed a VAP during VA-ECLS. Diagnosis of VAP was confirmed by a positive quantitative culture of a respiratory sample. VAP-TF was defined as composite of death attributable to pneumonia and relapse within 28 days of the first episode. Incidence, risk factors and impact on outcomes were analyzed.

To describe the population, Chi square test (categorical variables), Student t-test and Man Whitney tests (continuous variables) were used for statistical analysis. Statistical significance was defined as p < 0.05. Stepwise logistic regression was subsequently led to analyze risk factors for VAP treatment failure, with a threshold of p < 0.1 for inclusion in the model.

**Results** At all, 162 patients underwent ECMO support for >48 h. During the VA-ECLS support, 86 (53%) patients developed a VAP. The ICU-mortality was 57%. The median time to onset of VAP was 3 days after the beginning of mechanical ventilation. The main pathogens identified were Gram-negative Bacilli, *Pseudomonas aeruginosa* or enterobacteriaceae being involved in 80% of cases.

VAP-TF occurred in 28 (32.6%) patients. The *Pseudomonas aeruginosa* infection was the only risk factor for VAP-FT identified, odds ratio (OR) 3.7 (95% CI [1.4–10.6]; p = 0.02). The Renal Replacement Therapy (RRT) trended to increase the risk of VAP-TF, OR 3.4 (95% CI [1.0–12.0]; p = 0.05). In the subgroup of patients with *Pseudomonas aeruginosa* pneumonia (PA-VAP), neither the duration of antibiotic therapy nor the use of a combination of antibiotics was associated with VAP-TF. Changes in clinical or biochemical parameters from the VAP diagnosis (day 0) to day 14, such as leukocytes, procalcitonin, temperature, blood oxygenation, VA-ECLS flow or pump rotational speed, were not able to predict VAP-T. A VAP relapse was associated with an increased of duration of mechanical ventilation, VA-ECLS support duration and length of ICU or hospital stay. With this sample size, mortality was not impacted by VAP.

**Discussion** In this large cohort, VAP occurs early during VA-ECLS support while identified pathogens are those known to be involved for late-onset VAP. It could be explained by a very co-morbid status of patients in cardiac surgery ICU. VAP-TF occurs in 30% of patients, especially in PA-VAP while no guideline exists for the adequate antibiotic regimen and duration in patients treated with VA-ECLS. In addition, renal replacement therapy trends to increase VAP-TF. Antibiotics pharmacokinetics variations related to VA-ECLS and/or RRT support may contribute to this poor efficacy of VAP treatment.

**Conclusion** VAP in patients treated with VA-ECLS is associated with an important morbidity, and infection by *Pseudomonas aeruginosa* appears as a strong risk factor of treatment failure. Further studies seem necessary to precise the best antibiotic management in these patients.

**Competing interests** None.

**References**

1. Shekar K. The combined effects of extracorporeal membrane oxygenation and renal replacement therapy on meropenem pharmacokinetics: a matched cohort study. Crit Care. 2014;18(6):565.
2. Schmidt M, Bréchot N, Hariri S, Guiguet M, Luyt CE, Makri R, et al. Nosocomial infections in adult cardiogenic shock patients supported by venoarterial extracorporeal membrane oxygenation. Clin Infect Dis. 2012;55(12):1633–41.

#### O67 Development and validation of a computer algorithm to detect nosocomial infections in critically ill patients

##### Domien Coart^1^, Jasperina Dubois^2^, Tom Van Herpe^1^, Dieter Mesotten^3^

###### ^1^Intensive Care Medicine, UZ Leuven, Leuven, Belgium; ^2^Anesthesia & Intensive Care, Jessa Ziekenhuis, Campus Virga Jesse, Hasselt, Belgium; ^3^Anaesthesiology & Intensive Care, Ziekenhuis Oost-Limburg a.v., Genk, Belgium

####### **Correspondence:** Dieter Mesotten - dieter.mesotten@gmail.com

*Annals of Intensive Care* 2017, **7(Suppl 1)**:O67

**Introduction** ICU-acquired infections are common in critically ill patients and result in prolonged ICU-stay and increased healthcare costs. Moreover, the necessary antibiotic treatment may lead to a selection of multiresistant bacteria. Therefore, health authorities mandate the registration of specific nosocomial infections such as ventilator-associated pneumonia and catheter-related blood stream infections and wound infections. Unfortunately, there is no unified definition of nosocomial infection and its incidence depends on the screening intensity.

We therefore hypothesized that a computer algorithm using the prescribed antibiotic treatments may be an objective alternative for the detection of nosocomial infections.

**Patients and methods** As a gold standard, infections were scored by an infectious disease specialist.

The Java algorithm uses the following inputs: patient identifier, admission/discharge day, antibiotic name/dose/route of administration, administration days of antibiotics. The algorithm distinguishes infections on admission and nosocomial infections.

The algorithm was trained and validated in data sets of critically ill adults (ICU) and children (PICU) admitted to the ICU of the University Hospitals Leuven between 2012 and 2015.

**Results** In the training sets 314/335 PICU-patients and 352/383 ICU-patients received antibiotics. Gold standard scoring yielded a nosocomial infections rate of 11% in the PICU and 16% in the ICU-population. When the criterion of at least 2 days of antibiotics beyond the prophylactic spectrum, the algorithm yielded a nosocomial infection rate of 23% (PICU) and 21% (ICU). A True Positive Rate of 0.917, a False Positive Rate of 0.084, a Positive Predictive Value 0.671 and a Negative Predictive Value of 0.983 were achieved.

In the validation set only adult data were used. 384/408 patients received antibiotics. 55 had a nosocomial infection and 91 an infection on admission by gold standard measurement. Here a True Positive Rate of 0.873, a False Positive Rate of 0.067, a Positive Predictive Value 0.705 and a Negative Predictive Value of 0.976 were obtained. For scoring infections on admission the algorithm had a True Positive Rate of 0.785, a False Positive Rate of 0.058, a Positive Predictive Value 0.843 and a Negative Predictive Value of 0.917.

**Discussion** Sensitivity (TPR) and specificity (1-FPR) are comparable and acceptable around 0.85–0.90 for an algorithm that scores nosocomial infections with limited input variables. In its current settings it could serve as elimination tool for patients who definitely do not have a nosocomial infection. Infectious disease specialists can then focus on remaining patient files for scoring nosocomial infections.

Sensitivity needs to be increased while maintaining specificity for actual use in an environment with varying antibiotic prescription practices. The algorithm should also be further validated in lager datasets for different ICUs.

**Conclusion** In this proof-of-concept study, a computer algorithm was shown to be an acceptable alternative to trained infectious disease specialists in scoring nosocomial infections.

**Competing interests** None.

#### O68 Temporal trends in ICU-acquired bacteremia due to *Staphylococcus aureus* annual incidence in a French national ICU network

##### Sébastien Bailly^1^, Jc Lucet^2^, Alain Lepape^3^, François L’hériteau^4^, Martine Aupée^5^, Caroline Bervas^6^, Sandrine Boussat^7^, Anne Berger-Carbonne^8^, Anaïs Machut^9^, Anne Savey^10^, Jean-François Timsit^11^, REA-RAISIN study group

###### ^1^Iame team 5, INSERM UMR 1137, Paris, France; ^2^Hygiène hospitalière, Hôpital Bichat-Claude Bernard (AP-HP), Paris, France; ^3^Réanimation, Hospices Civils De Lyon, Lyon, France; ^4^Médecine interne, Hôpital Bichat-Claude Bernard (AP-HP), Paris, France; ^5^Hygiène hospitalière, C.H.U de Rennes, Rennes, France; ^6^Pharmacie, CHU - Hôpitaux de Bordeaux, Bordeaux, France; ^7^Réanimation, CHRU Nancy, Nancy, France; ^8^Dgos, Ministère des Affaires sociales et de la Santé, Paris, France; ^9^Cclin sud est, Hospices Civils De Lyon, Lyon, France; ^10^Cclin, Hospices Civils De Lyon, Lyon, France; ^11^Réanimation médicale et infectieuse, Hôpital Bichat-Claude Bernard, Paris, France

####### **Correspondence:** Sébastien Bailly - sbailly@chu-grenoble.fr

*Annals of Intensive Care* 2017, **7(Suppl 1)**:O68

**Introduction** *Staphylococcus aureus* (SA) is the most common isolated Gram-positive organism responsible for infection in ICU settings. The objective of this study was to assess in a French national network of ICU surveillance, the incidence of SA ICU-acquired bloodstream infection (BSI) overall and according to resistance profiles, either methicilin-susceptible (MSSA) and methicilin-resistant SA (MRSA), and the temporal trends of the incidence over 10 years, adjusted for patients’ case mix and ICUs’ characteristics.

**Materials and methods** Data from 2005 to 2014 were used. The incidence of BSI due to SA was assessed as the yearly ratio of ICU-acquired (>48 h.) SA BSI per 10,000 ICU patients. Only the first SA BSI occurred during one ICU stay for a patient was taken into account. Univariable autoregressive models were performed to assess the temporal trend in the evolution of the annual incidence during the study period. Multivariable autoregressive model were performed to adjust on case-mix and other centers’ characteristics with a significant annual trend selected in the univariable analysis.

**Results** Of 265,035 patients included from an annual median number of 158 participating ICUs (144 in 2005, 213 in 2014), 9553 (3.6%) had an ICU-acquired BSI, and 1476 (15%) of them had at least one SA BSI (56 per 10,000 ICU patients). One-third of SA BSI (n = 491, 33%) was MRSA and 907 (67%) were MSSA. There was a significant decrease of annual incidence for all SA BSI from 2005 to 2014 (64 to 48/10,000 ICU patients; −1.8/10,000 ICU patients per year; p = 0.02). The raw annual incidence of MRSA BSI decreased significantly from 34 to 11/10,000 ICU patients between 2005 and 2014 (−2.21/10,000 per year; p = 0.001). There was no significant change in the incidence of MSSA from 28 to 35/10,000 between 2005 and 2014 (+0.35/10,000 per year; p = 0.53) (Fig. 9). By adjusting on annual ICUs variables significant in the multivariable model (percent of medical-surgical ICU, public hospital, percentage of patients with antibiotic therapy at ICU admission, central venous catheter exposure, medical admission and annual median of age) there was no significant change in the overall incidence of SA BSI, but a significant decrease of MRSA (p = 0.02). The temporal trends were similar in all French regions.

**Fig. 9:**
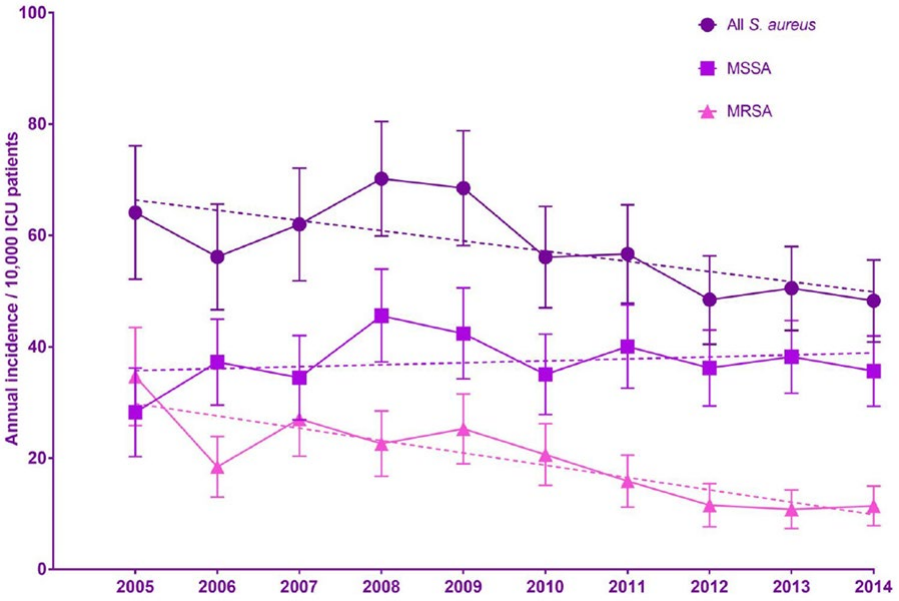
Annual incidence of ICU-acquired bacteremia due to *S. aureus* BSI, overall and according to resistance profiles from 2005 to 2014

**Conclusion** After adjustment on center variables, there was neither significant change in the overall incidence of *S. aureus* BSI nor in the incidence of MSSA BSI in ICU. However, the incidence of MRSA BSI decreased significantly. Moreover MSSA BSI did not significantly increase. MRSA rate trends in the ICUs participating to the network (about half the French ICUs) are in line with national MRSA trends.

**Competing interests** None.

#### O69 Diagnostic strategy in critically ill patients with hemophagocytic lymphohistiocytosis

##### Jean-Jacques Tudesq^1^, Sandrine Valade^1^, Lara Zafrani^1^, Lionel Galicier^2^, Cédric De Bazelaire^3^, Nicolas Munoz-Bongrand^4^, Emmanuel Canet^1^, Fanny Ardisson^1^, Virginie Lemiale^1^, Elie Azoulay^1^, Eric Mariotte^1^

###### ^1^Réanimation médicale, Hôpital Saint-Louis, Paris, France; ^2^Immuno-hématologie, Hôpital Saint-Louis, Paris, France; ^3^Radiologie, Hôpital Saint-Louis, Paris, France; ^4^Chirurgie viscérale, Hôpital Saint-Louis, Paris, France

####### **Correspondence:** Jean-Jacques Tudesq - jj.tudesq@gmail.com

*Annals of Intensive Care* 2017, **7(Suppl 1)**:O69

**Introduction** Hemophagocytic lymphohistiocytosis (HLH) is a life threatening condition characterized by NK/T cells/macrophage overactivation resulting in a cytokine storm with multiorgan dysfunction. In adults, HLH is mostly secondary to cancers (mainly hematological neoplasms), infections or auto-immune diseases.

HLH-etiology can be difficult to assess, especially in the critical care setting. Clinical and biological symptoms of HLH, its etiology and underlying immune deficiency being often mixed. Yet, targeting HLH-etiology is part of the ICU management in which supporting organ failure, early etoposide and empirical anti-infectious agents are routine. In this work, we assessed diagnostic yield of tissue biopsies to identify HLH-etiology.

**Patients and methods** This single center retrospective study was conducted between 2007 and 2016. Medical records of all consecutive patients admitted with a HLH diagnosis (defined using HLH 2004 criteria) were reviewed. The performance of each diagnostic procedure was established. Rstudio software was used for analysis.

**Results** Over the study period, 142 patients (45 women, 32%; median age 47 years [37–57]) were recorded. Acute respiratory failure was the main reason for ICU admission (40 cases, 28%). Ninety-six patients (68%) had a known immune deficiency (HIV 36%, other 32%). Median SOFA at admission was 7 [5–10]. All patients met at least 5/8 HLH criteria. HLH-etiology was identified in 137 patients (96%): malignancy in 102 (74%), infection in 28 (20%), autoimmune disease in 7 (5%). Life sustaining therapy was needed in 80 patients (56%): mechanical ventilation (52%), vasopressors (44%), or renal replacement therapy (37%). Hospital mortality was 42%, with no difference between patients with or without identified HLH-etiology.

Bone marrow smears were performed in the ICU in 77 cases and were mostly useful to contribute to HLH diagnosis (83% hemophagocytosis). However, only 23 (30%) identified HLH-etiology. Among tissues that were sampled, the highest and the lowest diagnostic yields were provided by spleen resection and liver biopsy, respectively (7/8 [87.5%] splenectomies and 11/30 [37%] liver biopsies allowed to establish a definite diagnosis).

Figure 10 displays respective contributions of other tissue examination. Interestingly, the best feasibility/contribution ratio was achieved by minimally invasive lymph node biopsy. Namely, among 97 patients with clinically relevant lymph nodes, 57 (59%) could be explored under echography or CT, and diagnostic yield was 74%.

**Fig. 10:**
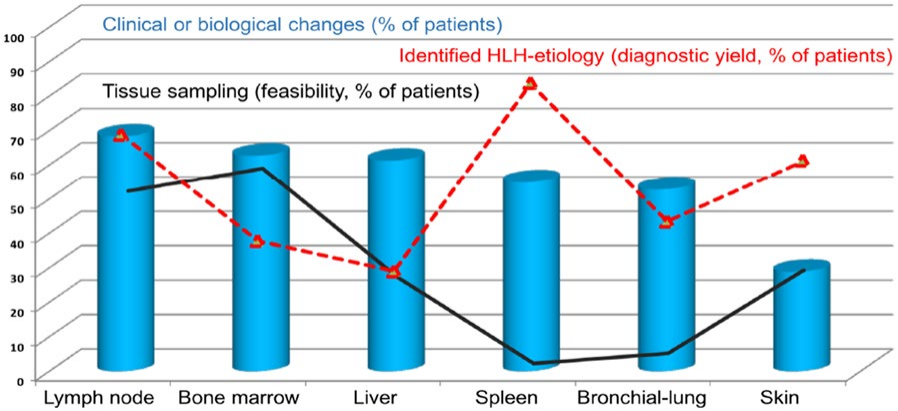
Diagnostic yield of tissue biopsies in patients with hemophagocytic lymphohistyocytosis

Severe adverse events included two cases of reversible hemorrhagic shocks, one following transjugular liver biopsy and one after splenectomy. No post-procedure infection was recorded.

**Discussion** Identifying HLH-etiology is a mandatory step for short and long term recovery. Moreover, when HLH patients are critically ill, identifying and targeting HLH etiology is the cornerstone of ICU management. However, in patients with multi-organ dysfunction, clinicians need guidance to understand which procedure is likely to identify HLH-etiology. In patients with HLH, pancytopenia or spleen enlargement are the rule. However, other organ involvement has to be sought in each specific case.

Even if splenectomy achieves the highest diagnostic yields, minimally invasive lymph node biopsy or skin biopsy are of value based on their feasibility/diagnostic yield ratio. Only few complications were identified in this work and minimally invasive procedures seem to be safe.

**Conclusion** This study provides important guidance to establish HLH-etiology. Timing for identifying HLH-etiology has nevertheless not been associated with mortality. Studies to assess whether targeting HLH-etiology in the initial management are warranted.

**Competing interests** None.

#### O70 Granulocyte colony-stimulating factor-induced neutropenia recovery and respiratory status deterioration in critically ill patients with hematologic malignancies

##### Xavier Mignard^1^, Lara Zafrani^2^, Lucie Biard^3^, Djamel Mokart^4^, Frédéric Pène^5^, Achille Kouatchet^6^, Julien Mayaux^7^, François Vincent^8^, Martine Nyunga^9^, Fabrice Bruneel^10^, Antoine Rabbat^11^, Christine Lebert^12^, Pierre Perez^13^, Anne Pascale Meert^14^, Dominique Benoit^15^, Michaël Darmon^16^, Elie Azoulay^2^, Virginie Lemiale^2^

###### ^1^Reanimation médicale, Hôpital Saint-Louis (AP-HP), Paris, France; ^2^Réanimation médicale, Hôpital Saint-Louis, Paris, France; ^3^Sbim, Assistance Publique Hôpitaux de Paris, Paris, France; ^4^Réanimation, Institut Paoli-Calmettes, Marseille, France; ^5^Réanimation Médicale, Hôpital Cochin, Paris, France; ^6^Service de Réanimation médicale et Médecine hyperbare, Centre Hospitalier Universitaire d’Angers, Angers, France; ^7^Réanimation médicale, Hôpital Pitié-Salpêtrière, Paris, France; ^8^Réanimation polyvalente, Groupe Hospitalier Intercommunal Le Raincy-Montfermeil, Montfermeil, France; ^9^Réanimation polyvalente, Centre Hospitalier de Roubaix, Roubaix, France; ^10^Réanimation médico-chirurgicale, Centre Hospitalier de Versailles, Le Chesnay, France; ^11^Réanimation pneumologique, Hôpital Cochin, Paris, France; ^12^Réanimation, Centre Hospitalier Départemental - site de La Roche-sur-Yon, La Roche-sur-Yon, France; ^13^Réanimation polyvalente, C.H.U. de Nancy, Nancy, France; ^14^Réanimation, Institut Jules Bordet, Bruxelles, Belgium; ^15^Réanimation polyvalente, Hopital universitaire, Gand, Belgium; ^16^Réanimation Médicale, CHU Saint-Etienne - Hôpital Nord, Saint-Étienne, France

####### **Correspondence:** Xavier Mignard - xavier.mignard@gmail.com

*Annals of Intensive Care* 2017, **7(Suppl 1)**:O70

**Introduction** In patients with haematological malignancies, neutropenia recovery is a situation where respiratory status may deteriorate. It has been previously demonstrated that granulocyte colony-stimulating factor (G-CSF) administration increases the risk of lung injury. ICU physicians are reluctant to use G-CSF in neutropenic patients with respiratory symptoms. However, data supporting this strategy are limited, consisting mainly of case series, retrospective data and experimental data. The aim of this study is to evaluate the impact of G-CSF administration on respiratory status in neutropenic patients with haematological malignancies admitted in ICU.

**Patients and methods** TRIAL-OH is a prospective, multicenter observational study that included 1011 patients with hematological malignancies who required ICU admission in 2010–2011 in 17 French and Belgian centers [1]. 288 patients who were neutropenic at ICU admission were included. The main endpoint was respiratory status deterioration at day 7, defined as any increase in respiratory SOFA score or death within the first 7 days of ICU stay. Using a propensity score (PS) based on the probability of receiving G-CSF during the first 48 h of ICU stay, we estimated the association between GCSF administration and respiratory function at day 7 on the matched sample, using a logistic regression model, adjusted on respiratory SOFA score at admission.

**Results** 288 neutropenic patients were included in the study. 201 (70%) did not receive G-CSF during the first 48 h of ICU stay. 87 (30%) received G-CSF at day 1 or day 2. 142 patients were selected by PS-matching. 57 (40%) were male, the median age was 58 (46–65) years. The most frequent malignancy was acute leukemia in 60 (42%) patients. The median SOFA score at admission was 6 (4–9). The respiratory SOFA score at admission was 0 for 103 patients (73%), 1–2 for 18 patients (12%) and 3–4 for 24 patients (15%). 11 (8%) patients had an invasive pulmonary aspergillosis.

After propensity score matching (71 patients/group), there was no significant association between G-CSF administration and respiratory status deterioration during the following 7 days (OR 1.08; 95% CI 0.55–2.13; p = 0.83), even though neutropenic patients who received G-CSF had better neutrophil recovery at day 7. 70 (49%) patients died or showed an increase of the respiratory SOFA score. Among them, 37 (53%) received G-CSF and 33 (47%) did not. Additional sensitivity analysis in patients admitted for acute respiratory failure showed similar results (OR 0.96; 95% CI 0.36–2.55; p = 0.93).

**Discussion** This is the largest study focusing on the association between G-CSF administration and respiratory status in critically ill neutropenic patients. Although the benefit of G-CSF in terms of mortality has never been demonstrated in this population, our data do not confirm G-CSF related pulmonary toxicity.

**Conclusion** In this study, G-CSF administration was not associated with deterioration of respiratory status in critically ill neutropenic patients with haematological malignancy.

**Competing interests** None.

**Reference**

1. Azoulay E, et al. J Clin Oncol. 2013;JCO.2012.47.2365.

#### O71 Rituximab-related complications in severe thrombotic thrombocytopenic purpura patients

##### Eric Mariotte^1^, Lara Zafrani^1^, Lionel Galicier^2^, Marion Venot^1^, Valade Sandrine^1^, Fanny Ardisson^1^, Virginie Lemiale^1^, Emmanuel Canet^1^, Benoît Schlemmer^1^, Elie Azoulay^1^

###### ^1^Réanimation médicale, Hôpital Saint-Louis (AP-HP), Paris, France; ^2^ Immuno-hématologie, Hôpital Saint-Louis (AP-HP), Paris, France

####### **Correspondence:** Eric Mariotte - eric.mariotte@aphp.fr

*Annals of Intensive Care* 2017, **7(Suppl 1)**:O71

**Introduction** Short term prognosis of thrombotic microangiopathies (TMA) has improved over the last decades with a mortality rate smoothing out to 10%. Main advances include management guidelines, improved understanding of the pathophysiology of most TMA syndromes and the development of targeted treatments. Acquired thrombotic thrombocytopenic purpura (TTP) in adults, an element of the TMA syndromes, is caused by a severe ADAMTS13 activity deficiency chiefly through anti-ADAMTS13 auto-antibodies. Rituximab, a chimeric anti-CD20 monoclonal antibody, has shown effectiveness for the treatment of refractory or recurrent TTP. In this study we focused on the short and long term complications of Rituximab administration in critically ill patients with severe TTP.

**Patients and methods** All TMA patients requiring admission to St Louis hospital ICU from 2006 to 2015 were enrolled in the study. Clinical, biological and follow-up data were retrieved from the patients’ medical chart and the hospital’s electronic database. Specific complications of TMA-therapies were specifically sought. Data are presented as numbers (%) and medians (interquartile range). Groups were compared using non-parametric statistical tests (Chi square test or Mann–Whitney).

**Results** During the study period, 124 critically ill TMA patients were recruited. There were 79 (64%) females, aged 44 years old (32–55). The TMA etiology included TTP in 81 (65%) cases, HUS in 24 (19%) and other TMA in 19 (15%) cases. Among the 81 TTP patients, 58 (72%) were females aged 42 years old (27–53). At diagnosis 66 (83%) patients presented with neurological symptoms, 50 (62%) with cardiac anomalies (whether clinical, EKG or biological anomalies), 46 (56%) with renal dysfunction, 28 (35%) with digestive symptoms. Day-1 hemoglobin was 7.4 g/dl (6.2–8.7), platelet rate was 11 G/l (8–19), creatinine was 96 µmol/l (73–148) and LDH rate was 1880 U/l (1410–2638). ADAMTS13 activity was undetectable in all patients, with auto-antibodies in 61/70 (87%). Day-1 SOFA was 6 (5–9) with 23 (28%) patients requiring mechanical ventilation, 12 (15%) requiring extra-renal epuration and 14 (17%) requiring vasopressors throughout the ICU stay. All patients received daily plasma-exchange (PEX) therapy, 78 (96%) corticosteroids, 55 (69%) 2 (1–2) anti-hypertensive drugs, 70 (86%) low dose Aspirin, and 62 (77%) received anticoagulation. Other therapies were administered in half the patients (n = 39) including Rituximab in 33 (41%) cases, pulse steroids in 23 (28%), Vincristine in 13 (16%), and other therapies in 11 (14%) (splenectomy, cyclophosphamide, twice daily PEX and vinblastine). ICU stay was 8 days (5–16) and 5 patients (6%) died in the ICU. The 33 patients receiving Rituximab in the ICU did not exert any benefit in terms of ICU or long term mortality. Rituximab administration was not associated with significant increase of ICU-acquired infections (9/33 (27%) vs 8/48 (17%), p = 0.2). No adverse reaction was noted following Rituximab infusion during the ICU period. Follow up was 1126 days (271–2001) and 3 additional patient died during follow-up. Eleven (14%) patients had TMA recurrence within 585 days (125–858). There was a trend toward a longer time to recurrence in Rituximab-treated patients (1475 days (427–2530) vs 136 days (80–749), p = 0.09). In addition, 22 TTP patients subsequently received Rituximab during follow-up either for TTP clinical recurrence or as maintenance therapy for persistent or recurrent ADAMTS13 undetectable activity. Long term complications potentially attributable to Rituximab included transient decrease in IgM levels (3 patients), serum disease in 1 patient, mild cognitive dysfunction in 1 patient and asymptomatic hepatitis B virus reactivation in 1 patient.

**Discussion** Rituximab, when used in patients with severe/refractory TTP, seems safe at short and long term. This apparently low complication rate may be due to the relatively low Rituximab dosage used as compared to regimen applied to patients with lymphoma. High dose steroids might also have been protective against acute cytokinic reactions. Last, the retrospective nature of this study might also have undermined transient or mild Rituximab-related toxicity.

**Conclusion** In critically ill patients with severe/refractory TTP, safety profile of Rituximab is good. Studies to assess its efficacy as early adjunctive therapy in non-refractory TTP patients are warranted.

**Competing interests** None.

#### O72 Long term health related quality of life in critically ill patients with hematological malignancies

##### Franck Ehooman^1^, Lara Zafrani^2^, Djamel Mokart^3^, Frédéric Pène^4^, Achille Kouatchet^5^, Julien Mayaux^6^, François Vincent^7^, Martine Nyunga^8^, Fabrice Bruneel^9^, Antoine Rabbat^10^, Christine Lebert^11^, Pierre Perez^12^, Anne Pascale Meert^13^, Dominique Benoit^14^, Rebecca Hamidfar-Roy^15^, Michaël Darmon^16^, Elie Azoulay^2^, Virginie Lemiale^2^

###### ^1^Réanimation médicale, Hôpital Saint-Louis (AP-HP), Paris, France; ^2^Réanimation médicale, Hôpital Saint-Louis, Paris, France; ^3^Réanimation, Institut Paoli-Calmettes, Marseille, France; ^4^Réanimation Médicale, Hôpital Cochin, Paris, France; ^5^Service de Réanimation médicale et Médecine hyperbare, Centre Hospitalier Universitaire d’Angers, Angers, France; ^6^Réanimation médicale, Hôpital Pitié-Salpêtrière, Paris, France; ^7^Réanimation polyvalente, Groupe Hospitalier Intercommunal Le Raincy-Montfermeil, Montfermeil, France; ^8^Réanimation polyvalente, Centre Hospitalier de Roubaix, Roubaix, France; ^9^Réanimation médico-chirurgicale, Centre Hospitalier de Versailles, Le Chesnay, France; ^10^Réanimation pneumologique, Hôpital Cochin, Paris, France; ^11^Réanimation, Centre Hospitalier Départemental - site de La Roche-sur-Yon, La Roche-sur-Yon, France; ^12^Réanimation polyvalente, C.H.U. de Nancy, Nancy, France; ^13^Réanimation, Institut Jules Bordet, Bruxelles, Belgium; ^14^Réanimation polyvalente, Hopital universitaire, Gand, Belgium; ^15^Réanimation médicale, C.h.u., La Tronche, France; ^16^Réanimation Médicale, CHU Saint-Etienne - Hôpital Nord, Saint-Étienne, France

####### **Correspondence:** Franck Ehooman - franck.ehooman@aphp.fr

*Annals of Intensive Care* 2017, **7(Suppl 1)**:O72

**Introduction** Outcome of critically ill patients with hematological malignancies has substantially improved over the last decade. However, data regarding the long term Quality of Life (QOL) of these patients are scarce. The purpose of this study is to assess post ICU burden and health related QOL, and to identify risk factors for long term QOL impairment.

**Patients and methods** TRIAL-OH is a prospective, multicenter observational study that included 1011 patients with hematological malignancies who required ICU admission in 2010–2011 in 17 French and Belgian centers. Ninety days and 1 year after ICU discharge, HRQOL was was determined by applying the interview form of the short-form 36 questionnaire (SF-36), which measures HRQOL in 8 separate dimensions (0 = worst health state, 100 = best health state). Psychological distress symptoms were evaluated using the Hospital Anxiety Depression Score (HADS) and the Impact of Event Scale (IES). All datas were collected prospectively

**Results** Two hundred and seventy-nine patients were evaluated at 3 months and 117 patients were evaluated at 1 year after ICU discharge.

At 3 months global median Physical (PS) and Mental scores (MS) were respectively 51.6 [48.0–55.3] and 49.7 [46.1–53.2], and at 1 year median PS and MS were respectively 63.8 [59.9–67.6] (p = 0.92) and 56.3 [52.5–60.0] (p = 0.05).

Physical functioning, general health, social functioning, mental health did not significantly change between 3 months and 1 year. Role Limitation due to physical problems (0.0 [0–6.2] vs 25.0 [17.7–32.3], p = 0.0008) and Vitality Score (40.0 [36.1–43.9] vs 46.7 [43.3–50.0] p = 0.018) significantly improved at 1 year. Role limitation due to emotional problems (72.4 [93.4–106.6] vs 66.7 [59.2–74.1], p = 0.001) and bodily pain (70.0 [64.2–75.8] vs 67.5 [63.0–72.0] were significantly worse at 1 year. At 3 months, 49 (17.5%) patients had an IES score at 20 and 139 (49.5%) patients had a HADS at 8

**Discussion** This multicentric study is the first to assess long term QOL in a large cohort of critically ill patients with hematological malignancies. Significant improvement in terms of short term mortality in this population do not fully reflect the impact of ICU stay. Prompt recognition of risk factors for impaired QOL and adapted therapy for anxiodepressive symptoms may improve their outcome

**Conclusion** Critically ill patients with hematological malignancies have impaired QOL at 3 months and recovery is incomplete 1 year after ICU discharge. Long term QOL should be taken into account in the management of these patients.

**Competing interests** None.

#### O73 Should we admit patients with hematological malignancies earlier to the ICU?

##### Yannick Hourmant^1^, Arnaud Mailloux^1^, Pierre Perez^2^, Djamel Mokart^3^, Virginie Lemiale^1^, Frédéric Pène^4^, Achille Kouatchet^5^, Julien Mayaux^6^, François Vincent^7^, Fabrice Bruneel^8^, Martine Nyunga^9^, Antoine Rabbat^10^, Christine Lebert^11^, Anne Pascale Meert^12^, Dominique Benoit^13^, Rebecca Hamidfar-Roy^14^, Sylvie Chevret^15^, Elie Azoulay^1^

###### ^1^Réanimation médicale, Hôpital Saint-Louis, Paris, France; ^2^Réanimation polyvalente, C.H.U. de Nancy, Nancy, France; ^3^Réanimation, Institut Paoli-Calmettes, Marseille, France; ^4^Réanimation Médicale, Hôpital Cochin, Paris, France; ^5^Service de Réanimation médicale et Médecine hyperbare, Centre Hospitalier Universitaire d’Angers, Angers, France; ^6^Réanimation médicale, Hôpital Pitié-Salpêtrière, Paris, France; ^7^Réanimation polyvalente, Groupe Hospitalier Intercommunal Le Raincy-Montfermeil, Montfermeil, France; ^8^Réanimation médico-chirurgicale, Centre Hospitalier de Versailles, Le Chesnay, France; ^9^Réanimation polyvalente, Centre Hospitalier de Roubaix, Roubaix, France; ^10^Réanimation pneumologique, Hôpital Cochin, Paris, France; ^11^Réanimation, Centre Hospitalier Départemental - site de La Roche-sur-Yon, La Roche-sur-Yon, France; ^12^Réanimation, Institut Jules Bordet, Bruxelles, Belgium; ^13^Réanimation polyvalente, Hopital universitaire, Gand, Belgium; ^14^Réanimation médicale, C.h.u., La Tronche, France; ^15^Service de biostatistique et information médicale, Hôpital Saint-Louis, Paris, France

####### **Correspondence:** Yannick Hourmant - yannick.hmt@gmail.com

*Annals of Intensive Care* 2017, **7(Suppl 1)**:O73

**Introduction** Over the last two decades, advances in hematology and critical care management have translated into improved outcomes for critically ill hematological patients. Concerns have been raised about delayed admission to the ICU, however, the strength of evidence remains weak.

**Patients and methods** In a substudy from Trialoh led by the GRRR-OH and published in 2013, we sought to assess how time from hospital to ICU admission is associated with hospital mortality. Data were collected from 17 ICUs from France and Belgium between January 2010 and May 2011. We used non parametric Wilcoxon tests and Fisher exact tests for baseline univariate comparisons across groups, p-value equal or below 0.05 were considered statistical significant. Regression logistic models were used to summarize predictive information for ICU mortality.

**Results** Based on non-parametric estimation, the cutoff value of 3 days for time between hospital and ICU admission was best associated with mortality (OR 1.66 (1.28–2.15), P = 0.0001). Among the 1007 included patients, 592 were admitted before day-3 and 415 after day-3. Non-Hodgkin’s lymphoma, acute myeloid leukemia, and myeloma represented about 70% of admissions in the two groups. Patients admitted earlier were older [62 (50–71) vs. 59 (48–67), p = 0.01], more frequently women (35 vs. 45%, p = 0.003), had higher Charlson scores [4 (3–6) vs. 3 (2–5), P = 0.007 mostly from diabetes, cardiovascular diseases and chronic kidney diseases], but less altered performance status 1 (0–2) vs. 2 (1–3), p < 0.0001). Early admitted patients, were more frequently admitted to the ICU from the ED/SAMU (43 vs. 2%, p < 0.0001) and less frequently enrolled in a hematology trial (23 vs. 33%, p = 0.0004). Patients admitted before day-3, more frequently carried a newly diagnosed malignancy (28.69 vs. 15.74%; p < 0.0001) and were less frequently acute myeloid leukemia patients (23 vs. 33%, p = 0.0009), recipients of hematopoietic stem cell transplants (both for autologous (18.16 vs. 11.53%, p = 0.004) and allogeneic (20.29 vs. 10.34%; p < 0.001)) or patients not in remission (36 vs. 46%, p < 0.0001). Delayed ICU admission occurred more frequently in patients with acute respiratory failure (70.3 vs. 57.2%, p < 0.0001) or coagulation disorders (23.7 vs. 16%, p = 0.003). Patients admitted after day-3, developed more frequently multiple organ failure (59.5 vs. 51%, p = 0.009).

By multivariable analysis with mortality as the outcome variable, admission after day-3 tended to be associated with mortality but did not reach significance when adjusted on patient’s characteristics and processes of care. An ongoing analysis to adjust on severity at ICU admission will be communicated in January.

**Conclusion** Patients admitted to the ICU more than 3 days after hospital admission have an increased mortality. As one-third of these patients were admitted after an initial refusal, these results identify the typology of patients for whom delayed ICU admission impacts on outcomes. Interventional studies are warranted.

**Competing interests** None.

#### O74 The effects of passive leg raising can be detected by the plethysmographic oxygen saturation signal

##### Alexandra Beurton^1^, Jean-Louis Teboul^1^, Valentina Girroto^1^, Galarza Laura^1^, Christian Richard^1^, Xavier Monnet^1^

###### ^1^Service de réanimation médicale, inserm umr s_999, université paris-sud, Hôpital de bicêtre, hôpitaux universitaires paris-sud, Assistance publique – Hôpitaux de Paris, Le Kremlin-Bicêtre, France, France

####### **Correspondence:** Alexandra Beurton - alex.beurton@gmail.com

*Annals of Intensive Care* 2017, **7(Suppl 1)**:O74

**Introduction** Volume expansion is aimed at increasing cardiac output. Nevertheless, a cardiac output monitoring technique is not always available. The perfusion index (PI) (Masimo Corp., Irvine, CA, USA), is automatically calculated from the plethysmographic waveform of oxygen saturation as the ratio of the pulsatile fraction, caused by blood flow, and the non-pulsatile fraction. We hypothesized that PI could be proportional to stroke volume and that it could detect changes in cardiac output during passive leg raising (PLR) and volume expansion (VE).

**Patients and methods** We included patients for which a PLR test was planned. We measured the changes in cardiac index (CI, PiCCO2 device, Pulsion Medical Systems, Munich, Germany) and PI before and during the PLR tests. If a VE (500 mL of saline infusion over 10 min) was performed, we also measured its effects on CI and PI.

**Results** Fifty-five PLR tests were performed in 30 patients. One case was excluded because of a poor oxygen saturation signal. Norepinephrine was administered in all cases at a mean dose of 1.6 ± 1.0 mg/h. The PLR test was positive (increase in CI ≥ 10%) in 26 “preload responsive” cases and negative in 29 “preload unresponsive” cases. During PLR test, in preload responsive cases, CI significantly increased by 30 ± 15% and PI significantly increased by 79 ± 45%. During PLR test, in preload unresponsive cases, neither CI nor PI changed significantly. PI was able to detect a positive PLR test with good accuracy (area under the receiver operating characteristic curve: 0.99 (95% confidence interval 0.90–1.00, p < 0.001). If PI increased >23%, a positive response to PLR could be diagnosed with a sensitivity of 100% (84–100%) and a specificity of 90% (74–98%). Volume expansion was administered in 15 cases with a positive PLR test. Taking into account both PLR and volume expansions, the changes in CI and PI (27 ± 11% and 73 ± 47%, respectively) were correlated (r = 0.73, p < 0.001). The PI value at baseline was <2 in 15 patients, but the ability of PI to track changes in CI was not poorer in these patients than in the other ones.

**Conclusion** The results of this preliminary study are that the perfusion index using pulse oximetry seems to accurately reflect changes in CI during PLR test and volume expansion. This could be a reliable way to assess preload responsiveness in a totally non-invasive way.

**Competing interests** JLT and XM are members of the Medical Advisory Board of Pulsion Medical Systems. JLT gave lectures for Masimo. The authors have no other conflicts of interest to declare.

#### O75 Exploration of tissue perfusion parameters around tracheal intubation procedure and mechanical ventilation initiation

##### Vincent Dubée^1^, Jeremie Joffre^2^, Naïke Bigé^3^, Simon Bourcier^3^, Hamid Merdji^1^, Julien Dang^1^, Gabriel Preda^1^, Jean-Luc Baudel^3^, Eric Maury^3^, Bertrand Guidet^3^, Hafid Ait-Oufella^3^

###### ^1^Service de Réanimation Médicale, Hôpital Saint-Antoine, Paris, France; ^2^Service de reanimation médicale, Hôpital Saint-Antoine, Paris, France; ^3^Réanimation médicale, Hôpital Saint-Antoine, Paris, France

####### **Correspondence:** Vincent Dubée - vdubee@gmail.com

*Annals of Intensive Care* 2017, **7(Suppl 1)**:O75

**Introduction** Mechanical ventilation is a common organ support therapy in critically ill patients. Mechanical ventilation initiation could have several hemodynamic consequences, ranging from tachycardia to life-threatening collapse. The objectives of this study were (*i*) to describe the microcirculatory and macrocirculatory changes following emergency tracheal intubation (TI) and (*ii*) to identify predictors of hemodynamic instability.

**Patients and methods** Prospective observational monocenter study conducted in a 18-bed medical ICU. Consecutive patients requiring tracheal intubation were eligible for the study. Data collection included general demographic parameters and comorbidities, indication for tracheal intubation and presence of sepsis. Global hemodynamic parameters (blood pressure, heart rate, cardiac index) and tissue perfusion parameters (mottling score, capillary refill time [CRT], toe-to-rectal gradient temperature) were recorded before, 5 min and 2 h after TI. Cardiac function parameters (left ventricular ejection fraction, right ventricular to left ventricular diameter ratio, presence of paradoxical septum), arterial blood gas and arterial lactate level were measured before and 2 h after TI. Hemodynamic instability requiring intervention (HII) was defined as any hemodynamic status degradation requiring intravenous fluid resuscitation (≥500 ml) or the introduction of vasopressors or an increase in vasopressor dose ≥20%. Difficulty of tracheal intubation was assessed using the Intubation Difficulty Scale.

**Results** Seventy-seven patients were included (male gender 61%) with a median age of 69 [interquartile range 56–81] years. Median SOFA score and SAPS2 were 7 [4–10] and 51 [41–64], respectively. Indication for tracheal intubation was hypoxemia (44%), hypercapnia (14%), and coma (32%). Most of the patients had sepsis (64%), including septic shock patients (22%).

Tracheal intubation had no significant impact on cardiac index, heart rate and tissue hypoperfusion, whereas median mean arterial pressure decreased from 82 [71–93] mmHg before intubation to 73 [66–84] mmHg 2 h after TI (P = 0.005, Wilcoxon signed rank test).

HII occurred in 38 patients (48%). Univariate comparison (Fisher’s exact test for discrete variables and Mann–Whitney U test for continuous variables) of these patients with those who did not experience HII indicated that male gender (P = 0.035), intubation for hypoxemia (P = 0.006), administration of norepinephrine before TI (P = 0.002), sepsis (P < 0.0001), higher SOFA score (P = 0.0005), higher SAPS2 (P = 0.006), mottling score ≥3 before TI (P = 0.046), higher baseline serum lactate level (P = 0.038) and higher baseline knee CRT (P = 0.029) were predictors for HII. In contrast, cardiac index, mean arterial pressure, baseline temperature gradient, index CRT before TI and Intubation Difficulty Scale were not associated with HII.

Sepsis patients had a relative risk (RR) of HII of 2.8 [95% confidence interval 1.8–4.4]. Among sepsis patients, a mottling score ≥3 was associated with a RR of HII of 9.8 [1.3–73.4], serum lactate level with a RR of 2.6 [1.2–5.6], and intubation for hypoxemia with a RR of 2.1 [1.2–3.8], as compared with the entire study population.

**Conclusion** In a non-selected ICU population, half of the patients required fluid resuscitation or vasoactive drugs in the 2 h following TI. Sepsis was the strongest predictor of hemodynamic instability. Tissue hypoperfusion parameters, especially mottling score, identified a subpopulation of septic patients with a high risk of hemodynamic instability. In contrast, global hemodynamic parameters such as cardiac index before TI did not predict bad hemodynamic tolerance of the procedure.

**Competing interests** None.

#### O76 The GRACE risk score in critically ill patients with sepsis and a suspected myocardial infarction

##### Cyrielle Desnos^1^, Michel Zeitouni^1^, Ines Belaroussi^1^, Michel Djibre^1^, Antoine Parrot^1^, Clarisse Blayau^1^, Jean-Pierre Fulgencio^1^, Christophe Quesnel^1^, Guillaume Voiriot^1^, Muriel Fartoukh^1^, Vincent Labbe^1^

###### ^1^Réanimation médico-chirurgicale, Hôpital Tenon, Paris, France

####### **Correspondence:** Vincent Labbe - vinclabbe@gmail.com

*Annals of Intensive Care* 2017, **7(Suppl 1)**:O76

**Introduction** Elevations of cardiac troponin values, suggesting a myocardial necrosis, are common in critically ill patients with sepsis and are associated with a poor prognosis. In this context, myocardial ischaemia is one of the main underlying mechanisms leading to necrosis of myocardial cells defining the myocardial infarction (MI). However identifying which septic patients with suspected myocardial infarction are at risk of mortality and major cardiac events is a clinical challenge [1]. Clinical evaluation may lack sufficient precision, leading to inappropriate medication and discharge. It is uncertain whether risk scores derived from cardiologic populations apply in this context. We aimed to assess whether the «Global Registry of Acute Coronary Events» [2] score (GRACE) may predict mortality in the intensive care unit (ICU) for septic patients with a suspected MI.

**Patients and methods** We conducted a prospective monocenter observational study from June 2012 to August 2016 in the medico-surgical ICU of Tenon Hospital, Paris, France. All patients with a suspected MI [significant cardiac troponin elevation with at least one of the following: symptoms of ischaemia, new significant ST-segment or T wave changes at electrocardiogram, acute left ventricular systolic dysfunction (1)] on ICU admission or during their ICU stay, were screened. Patients admitted for sepsis were included. The primary endpoint was to assess the performance of the GRACE score to predict ICU mortality. The secondary endpoints were to describe the respective occurrence of major cardiovascular events (stroke, cardiac arrest and reinfarction), major bleedings and cardiogenic shock during ICU stay.

**Results** During the study period, 238 out of 3774 patients (6.3%) had a suspected MI. Among them, 122 (51%) were admitted for sepsis (75 men, 66 years [IQR 25–75: 56–76], 80 with invasive ventilation, 78 with septic shock, median Simplified Acute Physiology Score II 50 [37–62]). Principal sources of infection were pulmonary (60%), urinary (19%), and abdominal (16%). The pathogen was identified in 78 patients (64%). 75% of suspected myocardial infarction occurred at admission or during the first day after admission (median time: 0 day [IQR 0–0]) either on ST-elevation (n = 23) or non ST elevation (n = 99). At the time of suspected MI, the median Sepsis-related Organ Failure Assessment Score was 8 [5–12]. The ICU length of stay was 8.5 days [4–15]. The ICU mortality rate was 0.30 (95% CI 0.22–0.39). At the time of suspected MI, the GRACE score was higher in the non-survivor group (201 [160–226]), as compared with the survivor group (176 [149–211]; p = 0.053). A cut-off value of 200 was associated with ICU mortality (OR 2.53, 95% CI 1.14–5.61; p = 0.022). The symptoms of ischaemia (13 vs 8%, p = 0.441), the level of high sensitivity cardiac troponin peak (1093 pg/mL [381–4054] vs 1248 pg/mL [554–2973], p = 0.66), and the left ventricular ejection fraction at echocardiography (29% [20–41] vs 35% [20–50], p = 0.21) were similar between non-survivors and survivors. The electrocardiogram ST-segment or T wave changes were less frequent in the non survivors than in the survivors (50 vs 69%, p = 0.041). Major cardiovascular complications, major bleedings, and cardiogenic shock occurred more frequently in the non-survivors than in the survivors (19 vs 4%, p = 0.012; 16 vs 7%, p = 0.086; 76 vs 36%, p < 0.001; respectively).

**Discussion** Critically ill septic patients with suspected MI had a high risk of cardiovascular events and bleeding. In this population, the GRACE scoring system was more accurate for predicting ICU death than symptoms of ischemia, troponin elevation, and left ventricular dysfunction. The systematic application of a validated risk score to septic ICU patients with a suspected MI could enable an early appropriate management, and provide a more reliable basis for adequate discharge and follow-up. These findings require validation in larger prospective studies.

**Conclusion** The GRACE risk score could be a promising score to help for predicting death in septic ICU patients with a suspected myocardial infarction.

**Competing interests** None.

**References**

1. Thygesen K, Alpert JS, Jaffe AS, Simoons ML, Chaitman BR, White HD, et al. Third universal definition of myocardial infarction. Eur Heart J. 2012;33(20):2551–67.
2. Elbarouni B, Goodman SG, Yan RT, Welsh RC, Kornder JM, Deyoung JP, et al. Validation of the Global Registry of Acute Coronary Event (GRACE) risk score for in-hospital mortality in patients with acute coronary syndrome in Canada. Am Heart J. 2009;158(3):392–9.

#### O77 Systemic capillary leak syndrome severe attacks admitted in intensive care unit

##### Marc Pineton De Chambrun^1^, Charles-Edouard Luyt^1^, François Beloncle^2^, Sybille Merceron^3^, Yannick Fedun^4^, Bernard Lecomte^5^, Jérôme Devaquet^6^, Nicolas Terzi^7^, Romain Sonneville^8^, Damien Contou^9^, Marc Puidupin^10^, Bruno Verdière^11^, Bertrand Souweine^12^, Jean-Paul Mira^13^, Elie Azoulay^14^, Laurent Argaud^15^, Alain Combes^1^, Zahir Amoura^16^, for the EurêClark study group

###### ^1^Service de réanimation médicale, Groupe Hospitalier La Pitié-Salpêtrière, Institut de Cardiométabolisme et Nutrition, Assistance Publique Hôpitaux de Paris, Paris, France; ^2^Département de réanimation médicale et de médecine hyperbare, C.H.U. d’Angers, Angers, France; ^3^Service de réanimation médico-chirurgicale, Centre Hospitalier de Versailles, Le Chesnay, France; ^4^Service de réanimation polyvalente, Centre Hospitalier Bretagne Atlantique, Vannes, France; ^5^Service de réanimation polyvalente, Centre Hospitalier Notre-Dame de la Miséricorde, Ajaccio, France; ^6^Service de réanimation polyvalente, Hospital Foch, Suresnes, France; ^7^Service de réanimation médicale, Clinique de Réanimation Médicale, Grenoble, France; ^8^Service de réanimation médicale et infectieuse, Hôpital Bichat-Claude Bernard, Assistance Publique Hôpitaux de Paris, Paris, France; ^9^Service de réanimation médicale, Hôpital Henri Mondor, Créteil, France; ^10^Service de réanimation polyvalente, Hôpital d’instruction des armées Desgenettes, Lyon, France; ^11^Service de réanimation polyvalente, Centre Hospitalier Général de Saint-Denis, Saint-Denis, France; ^12^Service de réanimation médicale, CHU Gabriel-Montpied, Clermont-Ferrand, France; ^13^Service de réanimation médicale, Hôpital Cochin, Assistance Publique Hôpitaux de Paris, Paris, France; ^14^Service de réanimation médicale, Hôpital Saint-Louis, Assistance Publique Hôpitaux de Paris, Paris, France; ^15^Service de réanimation médicale, Hospices Civils de Lyon - Groupement Hospitalier Edouard Herriot, Lyon, France; ^16^Service de médecine interne, Groupe Hospitalier La Pitié-Salpêtrière, Institut IE3 M, Assistance Publique Hôpitaux de Paris, Paris, France

####### **Correspondence:** Marc Pineton De Chambrun - marc.dechambrun@gmail.com

*Annals of Intensive Care* 2017, **7(Suppl 1)**:O77

**Introduction** Systemic Capillary Leak Syndrome (SCLS) is a rare disease characterized by recurrent life-threatening attacks of capillary hyper permeability in the presence of a monoclonal gammopathy (MG). During acute episodes, the leak of fluid and proteins from the intravascular compartment to the interstitium results in clinical signs of both acute hypovolemia and interstitial edema. Biological profile is pathognomonic with marked hemoconcentration and paradoxal hypoproteinemia. There are few data available about natural history and prognosis of severe SCLS attacks. The objectives of this study were to precisely describe natural history, outcome and mortality associated factors in severe SCLS attack.

**Patients and methods** Multicenter retrospective analysis of data from the European Clarkson registry (EurêClark). Criteria to retain SCLS’s diagnostic were; presence of a MG; ≥1 typical attack with clinical manifestations of hypovolemia and capillary leak; hemoconcentration with paradoxal hypo protidemia; exclusion of secondary capillary leak syndrome causes. Patients with severe attacks admitted in ICU were identified in EurêClark registry. Physician were contacted and offered to include the attack using a pre-established case report form. Categorical variables are expressed: n (%) and continuous variable: mean ± SD or median [IQR].

**Results** Between May 1992 and February 2016, 59 attacks in 37 patients were included. Sex ratio was 1.05 with an age of 51 ± 11.4 years. In ten patients, more than one attack was included. Thirty-four (92%) patients had an IgG MG with Kappa light chain in 20 (59%) patients. A trigger of the attack could be found in 34 (58%) patients with flu-like syndrome being the most frequent (89%). SAPS II score at admission was 54 [38–67] and SOFA score 6 [3–9]. Admission heart rate was 128 ± 21 bpm, admission arterial systolic, mean and diastolic blood pressure were respectively 75 [55–94], 60 [44–70] and 45 [36–60] mmHg. Frequent clinical manifestations at admission were; unaltered consciousness despite profound arterial hypotension (83%), asthenia (78%), faintness (64%), nausea and vomiting (53%), edema (51%), dyspnea (46%), marbles (42%), myalgia (39%) and abdominal pain (36%). Admission hemoglobin, protidemia, serum creatinine and arterial lactate were respectively 20.2 [17.9–22] g/dL, 50 [36.5–58.5] g/L, 176 [121–244.5] µmol/L and 4.6 [3.3–6.5] mmol/L. Five patients underwent phlebotomy for mistaken hyper viscosity syndrome and 57 (97%) received fluid therapy with a cumulated volume of 4.5 [2.8–10.6] L over 1 [1–2.5] days. Norepinephrine was administered in 28 (47.5%) patients, epinephrine in 10 (17%) and corticosteroids in 19 (32%). Twenty-two (37%) patients required mechanical ventilation and 18 (30.5%) underwent renal replacement therapy. Fifteen patients (25%) were treated with intravenous immunoglobulins (IgIV) during the attack. Compartment syndrome occurred in 12 (32%) patients and 11 (30%) died in ICU. In univariable analysis (over 37 unique patients) main factors associated with mortality were SAPS II (p = 0.006) and SOFA score (0.005), neurological dysfunction (neuro SOFA score ≥3, p = 0.003), high ICU maximum weight (p = 0.008), high cumulated volume of fluid therapy (p = 0.017), mechanical ventilation (p < 0.0001) and renal replacement therapy (p = 0.002) but not treatment with IgIV (p = 0.12). Multivariable analysis retained a SOFA > 10 (OR 10.4 [1.1–91], p = 0.04) and a cumulated volume of fluid therapy >8 L (OR 16.4 [1.2–230], p = 0.038) as independent factors of mortality. Treatment with IgIV (OR 11.5 [0.85–155], p = 0.066) was not independently associated with mortality.

**Conclusion** Our study presents the first large cohort of SCLC attacks admitted to ICU. Compartment syndrome was a particularly frequent complication and mortality over 59 attacks was 18.6%. High cumulated volume of fluid therapy seems to be associated with poorer outcome. There was a trend toward mortality in patients treated with IgIV during the attacks and such treatment should be used with caution.

**Competing interests** None.

#### O78 Characteristics and outcome of patients with precapillary pulmonary hypertension and admitted to intensive care unit for acute right heart failure

##### Constance Vuillard^1^, Jais Xavier^2^, Delphine Bourlier^2^, Amar David^2^, Sattler Caroline^2^, Montani David^2^, Simmoneau Gerald^2^, Sitbon Olivier^2^, Marc Humbert^2^, Savale Laurent^2^

###### ^1^Hôpital Bicêtre (AP-HP), Le Kremlin-Bicêtre, France; ^2^Val de marne, Hôpital Bicêtre (AP-HP), Le Kremlin-Bicêtre, France

####### **Correspondence:** Constance Vuillard - cvuillard@hotmail.com

*Annals of Intensive Care* 2017, **7(Suppl 1)**:O78

**Introduction** Acute right heart failure (RHF) remains the leading cause of death in patients with precapillary pulmonary hypertension (PH). However, no recent data on this life-threatening condition are available since the progress in severe PH management, the changes in lung graft allocation rules and the development of extracorporeal life support as bridge to transplantation.

**Patients and methods** The aim of this prospective, observational, single-centre study was to determine the characteristics and survival of patients suffering from pulmonary arterial hypertension (PAH), pulmonary veno-occlusive disease (PVOD) or chronic thromboembolic PH (CTEPH) and admitted to intensive care unit (ICU) for acute RHF. Risk factors associated with an increased likelihood of 1-month mortality were identified.

**Results** From March 2015 to April 2016, 83 patients (median age 59 years, female 53%, PAH 59%) were included in the study. Precapillary PH was diagnosed at the same time as acute RHF in 32.5% of cases. All patients were treated with intravenous diuretics. Catecholamines were initiated in 57 (68.5%) patients (dobutamine alone in 36% of cases and dobutamine + noradrenaline in 32.5% cases). Urgent lung transplantation was performed in 11 patients with PAH or PVOD including 4 after extracorporeal life support initiation. Urgent endarteriectomy was performed in 9 patients with CTEPH. Overall mortality rate in ICU, at 1 and 3 months was respectively 18, 20 and 28%. Mortality rate at 3 months of patients <50 years, 50 to 65 years and >65 years and treated with catecholamines was 7, 36 and 52% respectively. Risk factors for death at 1 month were: known PH (HR 4; p = 0.05), catecholamines initiation (HR 5; p = 0.03), identification of a triggering factor (HR 2.8; p = 0.04), average blood pressure at third day (HR 0.9; p = 0.001), weight loss in the first 3 days (HR 9; p = 0.01), urea at admission (HR 1.2; p < 0.01) and third day (HR 1.1; p = 0.003), creatinine at admission (HR 1; p = 0.05) and third day (HR 1.1; p = 0.047), BNP (HR 1; p = 0.002) and natremia (HR 0.9; p = 0.037) at third day.

**Conclusion** In the past 10 years, the phenotype of PH patients hospitalized in ICU for acute RHF has changed with a median age significantly higher in comparison with previous studie1. The risk of death at short-term remains high especially in patients treated with cathecholamine (35%). However, recent progress in lung graft allocation rules and management of acute RHF dramatically reduced the risk of death in young patients candidate for transplantation.

**Competing interests** None.

### Short presentations

#### P1 PHARMECMO: a pilot pharmacokinetic study of antibiotics in patients assisted by extracorporeal life support

##### Olivier Dujardin^1^, Adrien Bouglé^1^, Hamou Nora Ait^1^, Charles Vidal^1^, Guillaume Lebreton^2^, Joe Elie Salem^3^, Najoua El-Helali^4^, Julien Amour^1^

###### ^1^Département d’anesthésie et de réanimation, umrs inserm 1166, Hôpital Universitaire La Pitié-Salpêtrière, Université Pierre et Marie Curie, IHU ICAN, Paris, France; ^2^Service de chirurgie thoracique et cardiovasculaire, Hôpital Universitaire La Pitié-Salpêtrière, Université Pierre et Marie Curie, IHU ICAN, Paris, France; ^3^Centre d’investigation clinique paris est 1421, département de pharmacologie, Hôpital Universitaire La Pitié-Salpêtrière, Université Pierre et Marie Curie, IHU ICAN, Paris, France; ^4^Unité de microbiologie clinique et dosages des anti-infectieux, Groupe hospitalier Paris Saint-Joseph, Paris, France

####### **Correspondence:** Adrien Bouglé - adrien.bougle@aphp.fr

*Annals of Intensive Care* 2017, **7(Suppl 1)**:P1

**Introduction** Optimization of antibiotic therapy for extracorporeal Life Support (ECLS) patients remains a pharmacological challenge. Clinical studies suggest that individualized dosing strategies and therapeutic drug monitoring could facilitate the achievement of adequate antibiotic concentration. The objective of this pilot study was to observe the pharmacokinetic characteristics of commonly used antibiotics in intensive care unit (ICU) for patients treated with ECLS.

**Patients and methods** The PHARMECMO study was a pilot, prospective, pharmacokinetic study, conducted in ICU cardiac surgery. All patients treated with ECLS, with known or suspected sepsis and receiving antibiotic therapy, were eligible for inclusion. The concentration of each antibiotic was measured by a combination of liquid chromatography and mass spectrometry from blood samples. For intermittent administration of antibiotic, two successive measures were performed, under steady state conditions, both at 50% (CT50) and 100% (Cmin) of the dosing interval.

**Results** Forty-five eligible patients were enrolled for 68 inclusions allowing 114 analysed samples during 1 year. The median age was 63 years [interquartile range (IQR) 58–67], 84.4% of inclusions were male, with a median weight of 74.5 kg (IQR 70–86.75). Among the 68 inclusions, 39.7% received continuous veno-venous hemofiltration. ECLS therapy was veno-venous (n = 2), peripheral venoarterial (n = 51) or central venoarterial (n = 15). The most frequent causes of infection were pneumonia (n = 36), infection of femoral triangle (n = 5) and catheter-associated infection (n = 5). Of the pathogens identified, *Pseudomonas aeruginosa* was the most frequent (n = 20).

The main studied antibiotics were piperacillin-tazobactam (n = 17), cefotaxime (n = 12), imipenem (n = 10) and amikacin (n = 6). For the association piperacillin-tazobactam, the median CT50 was 87.00 mg/L (IQR 57.93–158.71) and the median Cmin 61.24 mg/L (IQR 44.92–90.51) for a dose of 4 g four times a day, with a MIC target of 16 mg/L. For cefotaxime, median concentrations were respectively, CT50 and Cmin of 64.69 mg/L (IQR 20.17–97.52) and 28.61 mg/L (IQR 7.28–42.12) for a MIC target of 1 mg/L and a median dose of 7 g per day. Regarding imipenem, at a dose of 1 g three times a day, the median concentrations were respectively, CT50 and Cmin, of 7.30 mg/L (IQR 4.07–14.59) and 3.28 mg/L (IQR 1.84–5.43) for a MIC target of 4 mg/L. Only one patient had a CT50 greater than 4 MIC, and 60% of measured Cmin were under the MIC. Finally, for amikacin, the median Cmax was 51.14 mg/L (IQR 32.36–78.26) at a median dose of 24.3 mg/kg, for a target between 60 and 80 mg/L.

**Conclusion** These preliminary data suggest that therapeutic drug monitoring could optimize the achievement of pharmacokinetic objectives associated with an effective antibiotic therapy. These data also suggest that, in most patients, the recommended doses of imipenem at 1 g three times a day and aminoglycoside at 20 to 25 mg/kg, do not respect the pK objectives reported in the literature.

**Competing interests** None.

#### P2 Variation in screen and isolate policy for multidrug-resistant bacteria (MDRB): a national survey in French adult ICUs

##### Zoé Coppere^1^, Guillaume Voiriot^1^, Aude Gibelin^1^, Clementine Taconet^1^, Vincent Labbe^1^, Muriel Fartoukh^1^, Michel Djibre^1^

###### ^1^Réanimation médico-chirurgicale, Hôpital Tenon -APHP, Paris, France

####### **Correspondence:** Michel Djibre - michel.djibre@aphp.fr

*Annals of Intensive Care* 2017, **7(Suppl 1)**:P2

**Introduction** The control of health-care associated infections and multidrug-resistant bacteria (MDRB) is a public health priority. The MDRB prevalence rate has dramatically increased, mainly extended-spectrum beta-lactamase producing Enterobacteriaceae (ESBLE). The aim of this study is to describe and to analyse the different screen and isolate policies regarding MDRB in French adult ICUs.

**Patients and methods** This is an observational, descriptive, multi-center online survey performed in French adult ICUs. The questionnaire included 63 questions divided into 4 parts: characteristics of the unit, MDRB screening policy, policy regarding contact precautions and ecology of the unit.

**Results** From April 2015 to June 2016, 73 of 301 French adult ICUs responded to the survey (24% response rate). A screening upon admission was performed in 96% of cases, mostly in a systematic way for at least one MDRB (78%). Screening was commonly used for ESBLE (98.6%), methicillin-resistant *Staphylococcus aureus* (89.8%) and, to a lesser extent, imipenem resistant *Acinetobacter baumanii* (47%). A targeted screening upon admission of highly resistant bacteria (HRB) was performed on high-risk patients in 86.3% of cases. A periodic MDRB screening during the ICU stay was performed in 83% of cases usually weekly (90%). Preemptive isolation of patients on ICU admission was performed in 82% of cases, and mostly in a targeted way (71%). Participants varied on gown and gloves wearing from standard precautions to consolidated additional contact precautions. About 23% of units used one or several specific decontamination methods. Imported and acquired MDRB rates >10% were noted in 44% and 27% of cases, respectively. Almost half of the units (48%) had already experienced a MDRB epidemic situation in the 3 preceding years.

**Discussion** French adult ICUs vary significantly in their MDRB screening and isolate approach, and about 10 combinations were encountered in the survey. The different approaches practiced were not always in agreement with the 2009 national guidelines for the prevention of MDRB transmission. Very few ICUs proceeded without screening and isolation at admission.

**Conclusion** Substantial variations exist in French ICUs practices regarding MDRB screening and isolation. The growing impact of imported and acquired MDRB rates and the frequency of epidemic situations in the ICUs may induce to reconsider and clarify the recommendations for prevention of “cross-transmission” of the French Society of Hospital Hygiene.

**Competing interests** None.

#### P3 Impact of routine decontamination on *Pseudomonas aeruginosa* acquired infections and antimicrobial susceptibility in an ICU

##### Adel Maamar^1^, Elen Colobert^1^, Pierre Fillatre^1^, Fabrice Uhel^1^, Yves Le Tulzo^1^, Christophe Camus^1^

###### ^1^Réanimation médicale, Centre hospitalier universitaire de Rennes, Rennes, France

####### **Correspondence:** Adel Maamar - adel.maamar@chu-rennes.fr

*Annals of Intensive Care* 2017, **7(Suppl 1)**:P3

**Introduction** Selective decontamination with various regimens using topical antimicrobials has been reported to reduce acquired infections (AIs) and mortality in the ICU. The efficacy of decontamination on the prevention of *P. aeruginosa* AIs is controversial [1] and potential impact on the emergence of antimicrobial-resistant strains is a concern. We assessed the incidence of *P. aeruginosa* AIs (PAAIs) with the routine use of a multiple decontamination regimen with oropharyngeal and digestive tobramycin/colistin/amphotericin B and nasal mupirocin/chlorhexidine body wash over 5 years with a special attention to antimicrobial resistance.

**Patients and methods** This was an observational single center study of all patients admitted to an ICU 2008–2012 (study population). Decontamination was given for the period of intubation and standard care otherwise. PAAIs were prospectively recorded. Rates of PAAIs (proportion of patients) were first compared between the study period and a 4-year pre-intervention period (2003–2006). During study, trends were analyzed by year using a logistic regression model. Categories were compared by Chi square test or Fisher’s exact test when appropriate. Continuous variables were expressed by median (25th–75th percentile) and compared using non-parametric test.er the text

**Results** Of the 5250 patients admitted to the ICU during the 5-year study period, 69 (1.3%) acquired 77 episodes of PA infection (vs 112 of 3603 patients [3.1%] during the pre-intervention period, *p* < 0.001). The incidence of PAAIs declined over time (OR 0.81 [0.68–0.96], p = 0.02). The proportion of patients who had clinical samples positive for PA at admission was 1.3% (70/5250) and did not vary with time (OR 1.03 [0.87–1.22], *p* = 0.75). Baseline characteristics of PAAIs patients were: SAPS II 55 (44–68), SOFA score 9 (7–12), prior antimicrobials exposition (number of molecules): 4 (2–5). AIs sites (n = 80) were respiratory (n = 52, ventilator-associated in 50), bloodstream (n = 15), genitourinary (n = 5), abdominal (n = 4) and other (n = 4). The delay of onset after admission was 13 days (7–22). Prior decontamination duration was 10 days (5–18). The susceptibility rate to 10 antimicrobials of the 80 isolates tested was as follows: ticarcillin (66.3%); piperacillin/tazobactam (82.5%); ceftazidime (93.8%); imipenem (52.5%); gentamicin (57.5%); tobramycin (97.5%); amikacin (96.3%); ciprofloxacin (67.5%]; fosfomycin (61.3%); colistin (98.8%). Susceptibility to all antimicrobials remained unchanged with time except for fosfomycin (susceptibility rate increased with time, OR 1.59 [1.05–2.39], *p* = 0.03). Antimicrobial therapy of 77 episodes consisted of a β-lactam agent (89.6%), ciprofloxacin (36.3%), aminoglycoside (31.1%). 6 episodes were not treated.

The mortality rate in ICU was similar in the patients with PAAIs (30.4%) and in those (n = 166) who acquired AIs not due to PA (31.3%, adjusted odds ratio = 0.84 [0.44–1.57], *p* = 0.58). The ICU mortality rate differed according to the site of the first episode: bloodstream 5/12 (41.6%); lung 16/46 (34.8%); other 0/11 (*p* = 0.03). In the patients with bloodstream or respiratory PAAIs, in multivariate analysis, SAPS II (per one unit increase OR 1.05 [1.00–1.10]) and non-susceptibility to piperacillin/tazobactam (OR 5.46 [1.04–27.77]) were independent risk factors for death in ICU (both *p* < 0.05). Aminoglycoside combination therapy was not associated with a higher cure rate (41%, vs no aminoglycoside 63%; *p* = 0.12).

**Discussion** Although all-cause AIs are declining in our ICU and the incidence rate has become low [2], PA remains the most common agent of AIs. 65% of PAAIs were ventilator-associated pneumonia. PAAIs were not associated with a higher ICU mortality rate than AIs due to other organisms.

**Conclusion** With the routine use of a decontamination regimen, AIs involving PA were controlled as well with no increase in antimicrobial resistance.

**Competing interests** None.

**References**

1. Hurley JC. Lack of impact of selective digestive decontamination on *Pseudomonas aeruginosa* ventilator-associated pneumonia: benchmarking the evidence base. J Antimicrob Chemother 2011;66:1365–73.
2. Camus C, Sauvadet E, Tavenard A, Piau C, Uhel F, Bouju P, Letheulle J, Dollo G, Gacouin A, Lavoué S, Le Tulzo Y. Decline of multidrug-resistant Gram negative infections with the routine use of a multiple decontamination regimen in ICU. J Infect 2016;73:200–9.

#### P4 Penicillin G susceptibility among *Staphylococcus aureus* is not so infrequent: a retrospective study

##### Josquin Moraly^1^, Redouane Dahoumane^2^, Vincent Dubée^1^, Gabriel Preda^1^, Jean-Luc Baudel^1^, Jeremie Joffre^3^, Naïke Bigé^1^, Hafid Ait-Oufella^1^, Eric Maury^1^

###### ^1^Réanimation médicale, Hôpital Saint-Antoine, Paris, France; ^2^Microbiologie, Hôpital Saint-Antoine, Paris, France; ^3^Service de reanimation médicale, Hôpital Saint-Antoine, Paris, France

####### **Correspondence:** Eric Maury - ejhmaury@gmail.com

*Annals of Intensive Care* 2017, **7(Suppl 1)**:P4

**Introduction** The incidence of resistance of *Staphylococcus aureus* to methicillin has decreased in France during the past decade leading to an increase use of methicillin and antistaphylococcal cephalosporins. It is usually stated that about 5% of methicillin-susceptible *Staphylococcus aureus* (MSSA) isolates are sensitive to penicillin G and penicillin G is unfrequently used to treat *Staphylococcus aureus* related infection. The aim of this retrospective observational study was to assess the rate of susceptibility to penicillin G among clinical *Staphylococcus aureus* strains isolated from ICU patients.

**Patients and methods** For a 10 years period, methicillin and penicillin susceptibilities of all *Staphylococcus aureus* strains isolated from clinical samples were analyzed. Repeat episodes and screening samples were excluded. Demographic data (age, sex), SAPSII score and in-ICU survival were recorded and analyzed according to penicillin G susceptibility.

**Results** From 01/2006 to 12/2015, 584 *Staphylococcus aureus* strains were isolated, 136 of whom being obtained from blood cultures, 327 from respiratory samples, 25 from urines 8 from arthritis. One hundred and three (17.6%) were susceptible to penicillin. Among all strains of *Staphylococcus aureus* isolated, the annual incidence of penicillin -susceptible strains varied from 5 to 24%. Over this period, global methicillin-resistant strains incidence was 15% with annual rate ranging from 4 to 31%. No difference was observed between age, sex of patients, SAPSII score or prognosis according to penicillin-G susceptibility (Table 8).

**Table 8:** Age, sex ratio, SAPSII score and in-ICU survival according to PeniG susceptibility

	All strains	Peni G-S	Peni G-R	P
	N = 584	N = 103	N = 481	
Age	64 ± 17	64 ± 18	66 ± 17	NS
Male	58%	58%	60%	NS
SAPSII	48 ± 21	49 ± 19	48 ± 2	NS
Survival	70%	70%	69%	NS

**Conclusion** This retrospective study suggests that penicillin susceptibility among clinical isolates of *Staphylococcus aureus* is not negligible and more frequent than the classical 5%. Microbiological laboratories should continue to test penicillin on *Staphylococcus aureus* strains. In this era of oxacillin shortage, penicillin G could be an appealing alternative in about 20% of cases.

**Competing interests** None.

#### P5 Impact of changing third-generation cephalosporin policy use on multiple-drug resistant bacteria nosocomial infections rates

##### Boun Kim Tan^1^, Vivier Emmanuel^1^, Misslin Pauline^2^, Parmeland Laurence^3^, Poirié Philippe^4^, Jean-Ralph Zahar^5^, Haond Catherine^6^, Pommier Christian^1^, Ait-Bouziad Karim^7^, Hocine Mounia^7^, Témime Laura^7^

###### ^1^Réanimation Polyvalente, Hôpital Saint Joseph Saint Luc, Lyon, France; ^2^Pharmacie, Hôpital Saint Joseph Saint Luc, Lyon, France; ^3^Biologie, Hôpital Saint Joseph Saint Luc, Lyon, France; ^4^Département d’information médicale, Hôpital Saint Joseph Saint Luc, Lyon, France; ^5^Unité de prévention et lutte contre les infections nosocomiales (uplin), CHU Angers, Angers, France; ^6^Hygiène hospitalière, Hospices Civils de Lyon, Lyon, France; ^7^Laboratoire mesurs, Conservatoire National des Arts et Métiers, Paris, France

####### **Correspondence:** Boun Kim Tan - tanbounkim@yahoo.fr

*Annals of Intensive Care* 2017, **7(Suppl 1)**:P5

**Introduction** Multiple-drug resistant bacteria carriage in general population and nosocomial infections are increasing in France and Europa even efficience of hands hygiene and antibiotic stewardship to control major hospital pathogens. Otherwise, antibiotics use have different consequences on bowel bacteria selection pressure. High biliary excretion of ceftriaxone and its long acting properties are responsible for a higher selection pressure in comparison with cefotaxime, an antibiotic drug with the same microbial spectre, lower biliary excretion, shorter action and a negligible effect on the bowel microflora.

**Patients and methods** Consequently, a new antibiotic policy was implemented in a 350-bed general hospital in Lyon (France) with the promotion of cefotaxim use instead of ceftriaxone from 1st January 2014 after an educational campaign in December 2013.

Hospital-acquired (HA) bloodstream meticillin-resistant *Staphylococcus aureus* (MRSA), third-generation cephalosporin-resistant Enterobacteriaceae (3GCRE), *Candida* infections and *Clostridium difficile* colitis cases per month/1000 patient-beds were identified and reviewed throughout our 12 beds-intensive care unit (ICU).

Monthly consumption of several antibiotics was monitored in defined daily doses (DDDs) per 1000 patient-occupied bed-days (1000 pt-bds) 24 months before (2012–2013) until 24 months after new antibiotic policy introduction (2014–2015).

**Results** Physicians have quickly followed new antibiotic policy and average monthly consumption of ceftriaxone reduced by 82% (from 119.6 to 26.6 DDDs/1000 pt-bds, p < 0.001) and cefotaxime increased in 40% (from 27.7 to 68.8 DDDs/1000 pt-bds, p < 0.001) between the first and final 24 months of the study in ICU.

Over the same periods, HA bloodstream infections rates were stable between the period before and after intervention for 3GCRE (1.71 vs 1.10 cases/1000 pt-bds, p = 0.33), MRSA (0.24 vs 0.08 cases/1000 pt-bds, p = 0.41), *Candida* (2.09 vs 2.76 cases/1000 pt-bds, p = 0.45). And HA *C. difficile* infections were similar between the two periods (0.60 vs 0.67 cases/1000 pt-bds for the second period, p = 0.82) in ICU.

Hand-hygiene compliance with alcoholic formulation (7.9 vs 10.31 ml per patient-days, p = 0.12) and consumption of all antibiotics (3936 vs 4520 DDDs/1000 pt-bds, p = 0.12) haven’t significantly changed before and after new antibiotic policy in our ICU and hospital.

**Discussion** While multiple-drug resistant bacteria nosocomial infections dramatically increase in France and Europa, trends in bloodstream 3GCRE, MRSA, *Candida* infections and *Clostridium difficile* colitis rates were stable in our ICU 2 years after introduction of a new antibiotic policy for third-generation cephalosporins. And our results seem different to some previous studies because only boodstream infections were considered in order to affirm multiple-drug resistant bacteria infections. But this method induced lower HA infections rates. Our results have to be analyzed after adjustment with multiple-drug resistant bacteria carriage at admission in ICU between the two periods. About 20% of patients carriers of multiple-drug resistant bacteria will be infected by the same bacteria during hospitalization indeed. A Poisson regression model (adjusted for overdispersion) will be used with log occupied bed-days as an offset. Linear trend terms were used to assess temporal changes. The main hypothesis was to see whether the trend in HA infections rates following antibiotic policy change was the same as the trend before the intervention. Poisson regression will be again used to assess which of the antibiotics was the better predictor of HA bloodstream 3GCRE, MRSA, *Candida* infections and *Clostridium difficile* colitis cases.

**Conclusion** Hospital new antibiotic policy promoting cefotaxime use instead of ceftriaxone didn’t seem to impact hospital-acquired bloodstream meticillin-resistant *Staphylococcus aureus*, third-generation cephalosporin-resistant Enterobacteriaceae and *Candida* infections and *Clostridium difficile* colitis rates after 2 years-monitoring in our intensive care unit. Further investigations are needed to precise the impact of dramatic increase in third-generation cephalosporin-resistant Enterobacteriaceae carriage on our results and the best predictors of these hospital-acquired infections.

**Competing interests** None.

**References**

1. Bräutigam HH, Knothe H, Rangoonwala R. Drugs. 1988;35 Suppl 2:163–8
2. Dancer SJ, Kirkpatrick P, Corcoran DS, Christison F, Farmer D, Robertson C. Int J Antimicrob Agents. 2013;41(2):137–42

#### P6 Assessment of antistaphylococcal antibiotics in intensive care: a 9-years monocentric retrospective analysis

##### Vero Hanitra Rasoldier^1^, Guy Mager^2^, Jean-Pierre Eraldi^1^, Stéphanie Gelinotte^1^, François Bougerol^1^, Julien Dehay^1^, Antoine Marchalot^1^, Jean-Philippe Rigaud^1^, Pierre Louis Declercq^1^

###### ^1^Réanimation polyvalente, Centre Hospitalier de Dieppe, Dieppe, France; ^2^Microbiologie, Centre Hospitalier de Dieppe, Dieppe, France

####### **Correspondence:** Vero Hanitra Rasoldier - vrasoldier@gmail.com

*Annals of Intensive Care* 2017, **7(Suppl 1)**:P6

**Introduction** Staphylococcal infections affect almost 30% of patients in intensive care units (ICU) in Western Europe (19.6% of *Staphylococcus aureus* (SA) infections and 11.2% of coagulase-negative *Staphylococcus* (CNS) infections). These infections are associated with increased risks of mortality, and increased prevalence of meticillin-resistance. During the last decade, new active antibiotics against meticillin-resistant *Staphylococcus* (MRS), linezolid and daptomycin appeared which changed the management of these infections. The objective of this study is to characterize patients infected with SA and CNS and assess prescribing habits concerning with antistaphylococcal antibiotics in ICU, 10 years after the advent of the new anti-MRS.

**Patients and methods** It is an observational retrospective study in a medical and surgical ICU. All patients with at least one SA or CSN-documented and treated infection between January 1st, 2006 and February 1st, 2015 have been included. A staphylococcal infection was defined by clinical, biological and microbiological criteria (based on thresholds of clinical guidelines). Antistaphylococcal antibiotic treatment was considered to be appropriate if at least one of the antibiotics used was effective against the germ according to the antibiogram. A descriptive analysis of the results was conducted.

**Results** Sixty-seven patients had at least one staphylococcal infection, corresponding to 74 infectious events including 63 SA infections (85%) and 11 CNS infections (15%). The average age of patients was 62 ± 15 years with a majority of men (62%) with numerous comorbidities (average Charlson of 4 ± 2). Average IGS and SOFA scores at 24 h were 60 ± 22 and 11 ± 5 respectively. There were mainly low respiratory infections (46 of 74, 62%) then 12 endocarditis and septicemias (16%) including 4 intravenous device infections. There were 47 nosocomial infections (64%) and the overall prevalence of MRS was 23.8% including 34% among nosocomial infections and only 4% among community-acquired infections. An empiric antibiotic therapy was prescribed in 89% of cases, and active against *Staphylococcus* spp. in 92% of cases. The empiric antibiotic therapy was effective against MRS for 48% of community-acquired and 43% of nosocomial infections. After the antibiogram reception, penicillin M was used for 46% of meticillin-sensitive staphylococcal infections, followed by amoxicillin-clavulanic acid (22%), and then vancomycin and linezolid (10% each). Vancomycin was the first used antibiotic in MRS infections (77%) with a vancomycin serum concentration at 48 h of 18 ± 8 mg/l, followed by linezolid (15%), mainly for the treatment of low respiratory infections. Daptomycin has never been used. Mortality rate at 28 and 90 days was 79% and 87% respectively, and the average length of stay in ICU was 15 days ([5; 27]).

**Conclusion** *Staphylococcus* spp. is essentially responsible for low respiratory infections, septicemias and infectious endocarditis, mostly nosocomial. These infections concern severely ill patients, with a low survival rate, without any causal link proved in this study. Empiric anti-MRS antibiotic therapy must be improved, particularly in a nosocomial condition. Vancomycin is still the first anti-MRS used 10 years after the provision of linezolid in France. Easier to use, empiric prescription of linezolid for nosocomial lung disease could help to improve patients’ management.

**Competing interests** None.

#### P7 Antibiotic therapy against methicillin-resistant staphylococcus aureus is inappropriate in ventilator-associated pneumonia with shock without prior methicillin-resistant staphylococcus aureus colonization

##### Julien Michel^1^, Nejla Aissa^2^, Sandrine Henard^3^, Philippe Guerci^4^, Ichraq Latar^5^, Bruno Levy^1^, Nicolas Girerd^6^, Antoine Kimmoun^7^

###### ^1^Service de réanimation médicale brabois, CHRU de Nancy, Vandœuvre-lès-Nancy, France; ^2^Service de microbiologie, CHRU de Nancy, Vandœuvre-lès-Nancy, France, France; ^3^Service de maladie infectieuse et tropicale, CHRU de Nancy, Vandœuvre-lès-Nancy, France; ^4^Service de réanimation chirurgicale brabois, CHRU de Nancy, Vandœuvre-lès-Nancy, France; ^5^Centre d’investigation clinique pierre drouin, CHRU de Nancy, Vandœuvre-lès-Nancy, France; ^6^Cardiologie - centre d’investigation clinique pierre drouin, CHRU de Nancy, Vandœuvre-lès-Nancy, France; ^7^Réanimation Médicale Brabois, Centre Hospitalier Universitaire de Nancy, Vandœuvre-lès-Nancy, France

####### **Correspondence:** Antoine Kimmoun - akimmoun@gmail.com

*Annals of Intensive Care* 2017, **7(Suppl 1)**:P7

**Introduction** Ventilator-associated pneumonia (VAP) is the leading cause of nosocomial infections in intensive care units. In case of VAP with shock, most recent international guidelines still recommend an early and broad-spectrum empiric antibiotic therapy focused on any infection due to Gram negative bacillus, including extended-spectrum β-lactamase–producing enterobacteriaceae (ESBL-EB), and MRSA. However, in recent years, there has been a change in the bacterial ecology, particularly a lower MRSA incidence. This study aims to demonstrate that, in patients with VAP and septic shock, an empiric antibiotic therapy with anti-MRSA activity without prior MRSA colonization is inappropriate.

**Patients and methods** Retrospective cohort study of patients with documented VAP in two intensive care units (ICU). All patients admitted to our ICU’s between January 2010 and December 2014 were included with the following criteria (1) mechanical ventilation for more than 48 h; (2) quantitatively significant culture of lower airways; (3) CPIS > 6. At admission and weekly thereafter, in all patients, was routinely performed multidrug-resistant bacterial species colonization by rectal or nasal swab collection.

**Results** Among 3629 patients under mechanical ventilation hospitalized in our ICU’s, 284 (7.8%) had a confirmed VAP, 172 (60.6%) with septic shock and 112 (39.4%) without. In the septic shock VAP group, 11 patients (6.4%) presented a VAP caused by MRSA. Among them, 10 were colonized by MRSA before VAP occurrence. No infection or colonization by MRSA were found in non septic shock VAP group. Interestingly, among the 284 patients with confirmed VAP, 141 (49.6%) (109 in the septic shock VAP group vs 32 without shock) were treated by anti-MRSA antibiotherapy (vancomycin or linezolid).

**Conclusion** MRSA colonization screening at admission and weekly thereafter is essential to avoid useless anti MRSA antibiotherapy in VAP patients with septic shock.

**Competing interests** None.

#### P8 Systematic screening of multidrug resistant bacteria in a Tunisian ICU

##### Saousen Ben Abdallah^1^, Sabrine Nakaa^1^, Kmar Hraiech^1^, Dhouha Ben Braiek^1^, Ali Adhieb^1^, Abdelwaheb M’ghirbi^1^, Ali Ousji^1^, Zeineb Hammouda^1^, Islem Ouanes^1^, Fahmi Dachraoui^1^, Lamia Ouanes-Besbes^1^, Fekri Abroug^1^

###### ^1^Réanimation polyvalente, CHU Fattouma Bourguiba, Monastir, Tunisia

####### **Correspondence:** Saousen Ben Abdallah - benabdallahsaoussen82@gmail.com

*Annals of Intensive Care* 2017, **7(Suppl 1)**:P8

**Introduction** Emergence of multidrug resistant (MDR) bacteria is a real problem worldwide and becoming increasingly important. The aim of this study was to describe the frequency and patterns of MDR bacteria following the implement of a systematic screening process at ICU admission in a Tunisian ICU.

**Patients and methods** Between 1st March 2016 and 31^st^ August 2016, consecutive patients admitted to our ICU following more than 24 h length of stay in another hospital ward, systematic screened for MDR bacteria. Specimens were recovered from nasal, axillary and rectal swabs. Micro-organisms cultures were made onchromogenic media.

**Results** During the study period 127 patients were admitted to the ICU, 32 of them were transferred from other wards and fulfilled the inclusion criteria. Colonization with MDR bacteria was present in 11 patients (34.3%) (median age 64 [IQR 39–75], 62% males, mechanical ventilation in 59.4%). 72% of patients were referred from the emergency department. During the study period 127 patients were admitted to the ICU, 32 of them were transferred from other wards and fulfilled the inclusion criteria. Colonization with MDR bacteria was present in 11 patients (34.3%) (median age 64 [IQR 39–75], 62% males, mechanical ventilation in 59.4%). 72% of patients were referred from the emergency department. During the study period 127 patients were admitted to the ICU, 32 of them were transferred from other wards and fulfilled the inclusion criteria. Colonization with MDR bacteria was present in 11 patients (34.3%) (median age 64 [IQR 39–75], 62% males, mechanical ventilation in 59.4%). 72% of patients were referred from the emergency department.

100% of the MDR bacteria were extended-spectrum beta-lactamase-producing enterobacteriacae (Table 9).

**Table 9:** Micro-organisms isolated with systematic (MDR) bacteria screening

	***N*** **=** ***11***
*Escherichia coli* [n (%)]	4 (36.3%)
*Klebsiella pneumoniae* [n (%)]	4 (36.3%)
*Klebsiella oxytoca* [n (%)]	1 (9%)
*Citrobacter freundii* [n (%)]	2 (18.1%)

No meticillin resistant staphylococcus aureus, nor carbapenemase producing pseudomonas aeruginosa or acinetobacter baumanii were isolated.

**Conclusion** MDR bacteria (100% extended-spectrum beta-lactamase-producing enterobacteriacae) is present in more than one-third patients transferred from the emergency department. The risk factors forMDR bacteria acquisition and a strategy to contain this phenomenon are needed in our hospital.

**Competing interests** None.

#### P9 Vancomycin-resistant enterococcus faecium bacteraemia in an intensive care unit: incidence and risk factors

##### Walid Sellami^1^, Zied Hajjej^1^, Walid Samoud^1^, Iheb Labbene^1^, Mustapha Ferjani^1^

###### ^1^Department of critical care medicine and anesthesiology, Military Hospital of Tunis, Tunis, Tunisia

####### **Correspondence:** Walid Sellami - drsellamiwalid@yahoo.fr

*Annals of Intensive Care* 2017, **7(Suppl 1)**:P9

**Introduction** Vancomycin-resistant *Enterococcus faecium* (VREF) is currently one of the most important etiologies of nosocomial infections in critically ill patients. In the continuum of VREF infections, bacteremia is of special interest, given that overall mortality rates may reach values higher than 60% with an attributable mortality of around 40%. The aim of our study was to determine incidence and risk factors associated with VREF bacteremia in an intensive care unit (ICU).

**Patients and methods** A retrospective case–control study was performed in the ICU of an university hospital in Tunisia from March 2008 to march 2016. Cases were defined as septic patients with VREF isolated from a blood culture (VREF group). Blood isolates were identified according to standard techniques and Vitek2 (bioMerieux).

VREF was defined as an *Enterococcus faecium* isolate with an MIC of vancomycin ≥32 μg/mL by the Etest (bioMerieux) according to the standards of the Clinical and Laboratory Standards Institute (CLSI).Control patients were randomly drawn from 65 hospitalized patients with vancomycin-susceptible *Enterococcus faecium* isolated from a blood culture (VSEF group). Medical records of the patients were reviewed. If patients developed several episodes of VREF bacteremia during the study period, only the first episode was investigated.

**Results** A total of 20 case patients (4.89 per 1000 admissions) and 20 control patients with at least one positive *E. faecium* blood culture were identified. The demographic and clinical characteristics of the case and control groups were similar, except for mean duration of length of stay, with values being significantly greater in the case group (66 ± 12 vs 24 ± 8, p < 0.001). In 14 cases, VREF bloodstream infections were related to intraabdominal infections; six cases had catheter-related VREF bloodstream infections. Mortality among these bacteremic patients did not differ significantly between those with VREF (14%) and those with VSEF (10%) isolates (p = 0.36). In the univariate analysis, the significant risk factors for VREF bloodstream infections included diabetes mellitus, arterial hypertension, end-stage renal disease, prior exposure to immunosuppressive agents notably corticosteroids, prior receipt of vancomycin before VREF identification and a prolonged length of stay in ICU (Table 10).

**Table 10:** Risk factors for VREF by univariate analysis

	EFRV (n = 20)	EFRV (n = 20)	p
Diabète	15	0	0.006
HTA	15	0	0.006
I Rénale C	15	5	0.03
Corticothérapie	17	5	0.04
Prise de Vanco	15	2	0.048

**Discussion** Enterococcus is the third most common cause of nosocomial bloodstream infection. Vancomycin-resistant Enterococcus (VRE) is an important problem in Europe, USA, and Latin America and has been isolated in many other countries. Infections due to VRE have been shown to be associated with significant in-hospital mortality and morbidity. Despite the fact that 80- 90% of clinical isolates of enterococci are E. faecalis, most VRE are E. faecium [1]. Similarly, in our institution, the vast majority of VRE bacteremia cases are caused by E. faecium.

**Conclusion** The incidence of VREF bloodstream infections was high compared with literature data.

Several risk factors have been identified and they should be considered in infection control practice to prevent VREF infection or colonization and to reduce they duration. This association has been previously suggested only in studies that have been limited by small numbers of patients and a failure to perform multivariate analysis. The retrospective analysis of a relative small cohort of patients is the major limitation of our study considering that we cannot be certain that we have identified all potential confounding factors.

**Competing interests** None.

**Reference**

1. Murray BE et al. N Engl J Med. 2000;9,342(10):710–21

#### P10 Convulsive crisis of the adult in the emergency: epidemiological and clinical characteristics

##### Fatma Kaaniche Medhioub^1^, Rania Allela^2^, Najla Ben Algia^3^, Samar Cherif^4^

###### ^1^Faculté de médecine de Sfax, Sfax, Tunisia; ^2^Hopital régional mahres, Faculté de médecine de Sfax, Sfax, Tunisia; ^3^Intensive care, hopital régional Gafsa, Sfax, Tunisia; ^4^Intensive care, hopital régional mahres, Sfax, Tunisia

####### **Correspondence:** Fatma Kaaniche Medhioub - fatma_kaaniche@yahoo.fr

*Annals of Intensive Care* 2017, **7(Suppl 1)**:P10

**Introduction** The convulsive crisis is a symptom indicating a hyper synchronous discharge of a more or less extensive portion of the cerebral cortex. It is a frequent disease in the emergency (0.3 to 1.2%) [1]. The aim of our study was to report the epidemiological, clinical, therapeutic and evolutionary aspects of the convulsive crisis in the emergency.

**Patients and methods** Prospective study extending on year (01/01/2015 - 31/12/2015), including all patients aged over 18 consulting in the emergency for convulsive crisis.

**Results** We included 41 patients. The average age was 45 ± 19 years with a sex ratio of 1.5. The convulsive crisis occurred at a known epileptic in 53.6% of cases. The average duration of crisis was 10 ± 4 min. The average time of consultation was 54 ± 21 min. Generalized tonic–clonic seizures were observed in 30 patients. Partial seizures in 11 patients. The status epilepticus has been reported in 12 patients (29.2%). Discontinuation of antiepileptic therapy was noted in 20 cases (48.7%), intoxication in 6 cases (14.6%), hypoglycemia in 5 cases (12.1%), head trauma in 5 cases (12.1%), accident cerebrovascular in 3 cases (7.3%) and preeclampsia in 2 cases (4.8%). The convulsive crisis was accompanied by a post-critical deficit in 6 patients (14.6%) and a head injury in 2 patients (4.8%). A brain imaging was indicated in 18 patients (43.9%). Benzodiazepines were prescribed in 78% of cases and barbiturates in 24.3% of cases. The use of invasive mechanical ventilation was reported in 12 patients (29.2%). Thirteen patients were hospitalized in intensive care, 12 in neurology and 6 in medicine department. There were no deaths.

**Conclusion** The management of convulsive crisis in the emergency is multidisciplinary. It is based on a good mastery of the definitions and recommendations on this subject.

**Competing interests** None.

**Reference**

1. Dunn MJ, Breen DP, Davenport RJ, Gary AJ. Early management of adults with an uncomplicated first generalised seizure. Emerg Med J. 2005;22:237–42.

#### P11 Admission in stroke units (SUs): For which patient if there is only one bed left?

##### Delphine Attia^1^, Andrianjafy Herinjatovo^1^, Xavier Laborne Francois^2^

###### ^1^URGENCES, Centre Hospitalier Général de Longjumeau, Longjumeau, France; ^2^Samu, C.H. Sud Francilien, Corbeil-Essonnes, France

####### **Correspondence:** Delphine Attia - delphine@attia.com

*Annals of Intensive Care* 2017, **7(Suppl 1)**:P11

**Introduction** Stroke is a frequent and serious disease. Stroke units (SUs) have demonstrated efficiency in reducing death and disability after stroke. In France, public health autorities’s reports recommended that every patient presenting with stroke symptoms should be admitted in SUs. But beds in these units are still insufficient, so triage seems unavoidable. We aimed to explore admission criteria in SUs in case there is only one bed left. In this situation, is the time window allowed thrombolysis the main criteria for neurologists to accept a patient?

**Patients and methods** This was a postal and e-mail survey that took place from January 1st to July 31st 2016. An anonymous questionnaire was sent to the 164 neurologists who usually work in the 21 SUs in the region of Ile de France. The survey was in two parts: the first one encompassed questions about the physicians themselves (age, professional status, current job tenure), and, then, in the second part we presented different clinical cases including the type of stroke (ischemic, hemorrhagic, or transient ischemic attack), age ranges of the patients, and different time slots during nyctohemeral period. Finally, data were collected in July 2016.

Results were expressed as absolute numbers and percentages, and for statistical analyses, we used the Fisher’s exact test from the on-line software BiostaTGV.

**Results** One hundred four questionnaires were completed, representing 63% of all. Majority of physicians were hospital practitioner (53%), and 56% have been working in SUs for more than 5 years. We asked to neurologists if they already refused a patient when it stay one bed in their unit, to keep it for a patient who could be eligible for thrombolysis, and the answer was positive in 64%.

There was no significant difference in accepting or denying regardless of the type of stroke or the different time slot. Concerning the age ranges, we found a significant relationship only with ischemic stroke (p = 0.014), as the oldest patients above 90 years old were more refused than the youngest.

**Discussion** Our study has several limitations, and probably the main is the risk of declarative biases due to descriptions of practices that differ from those encountered in real life. A more rigorous methodologic analysis, including a prospective study following patients suffering from stroke, would perhaps more reflect the reality.

**Conclusion** In case there is one bed left in their SUs, neurologists seem to accept most patients, even if thrombolysis is off limits. Only the age ranges may influence their decision if the patient suffers from ischemic stroke. More studies are necessary to confirm or not these results.

**Competing interests** None.

#### P12 Assessment of hemorrhagic stroke’s management in intensive care unit

Med Aziz Bouhouri^1^, Mohamed Taoufik Slaoui^1^, A. Soufi, K. Khaleq^1^, D. Hamoudi^1^, A. Nsiri^1^, R. Harrar^1^

##### ^1^Reanimation des urgences chirurgicale, chu ibn rochd, Casablanca, Morocco

###### **Correspondence:** Mohamed Taoufik Slaoui - dr.t.slaoui@gmail.com

####### *Annals of Intensive Care* 2017, **7(Suppl 1)**:P12

**Introduction** The management of hemorrhagic stroke (AVCH), considered the second cause of death in the world, has in recent years major changes. Early diagnosis and optimization of Early and specialized load have also shown their interest on the prognosis of patients

**Purpose** Through this analytical study, we emphasize the quality of the management of the hemorrhagic stroke.

**Materials and methods** We realized a retrospective study about 50 patients hospitalized at the resuscitation department of the surgical emergencies at the UHC Casablanca during a period of 4 years from January 2012 to December 2015. It included 31 men and 19 women, the meanagewas51 ± 15 yearsold. The epidemiological, clinical, paraclinical, therapeutic, and evolutive data were collected and gathered on a card then entered and analyzed on Epi Info software.

**Results** 50 patients were collected whose average age is 52 plus or minus 15 years male predominance is noted.

Hypertension is the most common risk factor for 41% followed by diabetes with a rate of 26%.

In our study, the recruitment rate was highest in autumn period 33.33%.

The mode of early neurological symptoms was brutal in 74% of 37 patients.

On admission clinical examination showed impaired consciousness with a Glasgow score ≤10 33 patients or 66%. Hemiplegia is the main focal neurological signs met the most common site of cerebral hemorrhage is lobar.

Brain CT showed an intra-parenchymal hemorrhage in 40 patients or 79% of cases with a predominance of the parietal location (23%). A pure subarachnoid hemorrhage was noted in 4 patients 10% of cases. 46% of patients had ventricular flooding.

Mechanical ventilation was required in 37 patients or 74%. Antihypertensive therapy was prescribed in 30 patients or 59%. Antiepileptic therapy was indicated in all patients.

The surgical indication was raised in 22 patients or 44%. 14 patients died after surgery.

The outcome was favorable in 14 patients or 28%, and negative in 36 patients or 72% of cases.

**Conclusion** In the literature, various predictive factors of mortality are observed. The prognosis of the hemorrhagic stroke in the units of intensive cares is still dark, hence the importance to develop specialized centres in order to improve the management of the patients who suffer from this affection.

**Competing interests** None.

#### P13 Optic nerve sheath diameter assessment: it depends of the probe

##### Jean-Luc Baudel^1^, Vincent Dubée^1^, Gabriel Preda^1^, Jeremie Joffre^2^, Naïke Bigé^1^, Hafid Ait-Oufella^1^, Eric Maury^1^

###### ^1^Réanimation médicale, Hôpital Saint-Antoine, Paris, France; ^2^Service de reanimation médicale, Hôpital Saint-Antoine, Paris, France

####### **Correspondence:** Eric Maury - ejhmaury@gmail.com

*Annals of Intensive Care* 2017, **7(Suppl 1)**:P13

**Introduction** Optic nerve sheath diameter (ONSD) is an easy to obtain parameter, which has been associated to absolute value and variation of intracranial pressure. However, the normal value of this parameter (observed in patients without raised intracranial pressure) remains debatable (from 5 to 5.9 mm) highlighting the importance measurement precision. Is this pilot observational study, we aimed to compare the ONSD values obtained with two different US device.

**Patients and methods** During a 2-month period, all consecutive patients recovering from acute disease, orientated, spontaneously breathing, without vasocative support were deemed to be included. Age sex, height, weight, BMI, SAPSII, myopia and other relevant ophtlamologic diseases were recoreded. After oral consent, ONSD of both right and left eyes were measured with two distinct US devices: Vivid 7 (GE, equipped with a 8 MHz (4–11 MHz) microconvex probe and a M Turbo, (Sonosite, Bothwell, MA) equipped with a HFL38 probe (6–11 MHz). Patients characteristics are expressed as qualitative or quantitative non parametric value as appropriate. The ONSD values obtained with the two devices were compared using Wilcoxon matched-paired test. The bias between the two measurements was expressed according to the Bland–Altman method.

**Results** During the study period, 25 patients (age: 59 years [50–66], SAPSII: 47[30–64], weight 69 kg [52–86], height 168 cm [158–175]; BMI 23 [20–29], myopia 3/25) were studied. Measurements, performed by a senior intensivist skilled in ultrasonography, were always possible with acceptable discomfort. Maximum values observed were 7.9 mm. ONSD values were similar between right and left eyes with the Vivid7 device (5.8 mms 5.7 mm, p = .9) and with MTurbo (4.6 vs 4.7 mm, p = .7) but were significantly greater with the Vivid device (p = .002 and p = .003 for right and left eye respectively). The values were significantly correlated (Spearman coeff r = 0.39 p = .003). No correlation was observed between ONSD values and height, weight or BMI. Myopia was not associated with greater values. The bias (difference mean) 1.16 m provided limits of agreement (±2SD) [−0.0589462, 0.291990) which included all except 3 patients.

**Discussion** In this small unselected medical ICU patients population with no neurological injury and unlikely to have raised intracranial pressure, the two US devices provided statistically different values with higher values with one of the devices.

**Conclusion** The ONSD is an easy to obtain parameter but depends on the US devices, which provides significantly different values.

**Competing interests** None.

#### P14 Is there a relevant experimental model of subarachnoid hemorrhage? Systematic review of preclinical literature

##### Suzanne Goursaud^1^, Maxime Gauberti^2^, Paul-Emile Labeyrie^2^, Thomas Gaberel^3^, Véronique Agin^2^, Eric Maubert^2^, Denis Vivien^2^, Clément Gakuba^1^

###### ^1^Calvados, Pôle Anesthésie-Réanimation C.H.U de Caen, Caen, France; ^2^Calvados, INSERM U919, Cyceron, Caen, France; ^3^Calvados, Neurochirurgie, C.H.U de Caen, Caen, France

####### **Correspondence:** Suzanne Goursaud - goursaud.suzanne@wanadoo.fr

*Annals of Intensive Care* 2017, **7(Suppl 1)**:P14

**Introduction** Delayed cerebral ischemia (DCI) is the main cause of disability after subarachnoid hemorrhage (SAH) but its pathophysiology remains unclear. We currently have no effective treatment to reduce the incidence of DCI. Study of pathophysiology has long suffered from a lack of consensus definition of DCI. It is only recently that experts have proposed that in observational studies and clinical trials aiming to investigate strategies to prevent DCI, the 2 main outcome measures should be: (1) cerebral infarction identified on CT scan or magnetic resonance imaging or proven at autopsy, after exclusion of procedure-related infarctions; and (2) functional outcome [1]. Currently, experimental studies must therefore meet this new definition. The aim of our study is to identify the extent preclinical studies using this new definition of DCI.

**Materials and methods** Systematic review from PUBMED using key words. Consulting additional bibliographic databases including gray literature, scientific meeting abstracts and review of the bibliography of selected articles completes the research. Inclusion criteria: (1) description and/or modification of an SAH model in rats or mice; (2) study of cerebral vasospasm and/or DCI. This research is performed in accordance with the PRISMA recommendations [2].

**Results** Seventy-one publications from 47 teams are included. 11 teams are responsible for 77% of publications. 8 different methods are used for induction of SAH. The most used model is the direct blood injection into the *cisterna magna.* Vasospasm is studied by brain imaging (n = 18) mainly in the first 72 h and histology (n = 32) until the tenth day. A positive diagnosis of vasospasm is placed in histology in 28 of 32 studies. Cerebral ischemia is sought in 24 publications including one in cerebral imaging. Neurological deficit is wanted by sensorimotor tests in 13 publications. Three models use animals with comorbidities (hypertension, obesity and chronic inflammation). No model uses female population. Among the 10 most cited studies, none of them is studying cerebral ischemia by imaging.

**Discussion** Differences with human pathology of SAH are raised about the pathophysiology leading to SAH and its anatomoclinical characteristics. In particular, there is an over-representation of models of posterior circulation. Similarly, study of vasospasm remains the most studied criteria to the detriment of the ischemia and neurological deficit, even though it is the first cause of disability after SAH (Table 11). Furthermore, we can note an over-estimation of the incidence of vasospasm in the included publications compared to the clinical epidemiological data. Study methods of vasospasm and cerebral ischemia may be criticized.

**Table 11:** Table of ten most cited studies

Studies	Models	Vasospasm	Delayed cerebral ischemia		
			Imaging	Histology	Behavioral study
Bederson (1995)	Endovascular perforation	NA	NA	NA	No focal neurological deficit
Bederson (1998)	Endovascular perforation	Yes	NA	NA	NA
Delgado (1985)	Direct injection	Yes	NA	NA	NA
Solomon (1985)	Direct injection	NA	NA	NA	NA
Prunell (2003)	Direct injection	NA	NA	Yes	Yes
Jackowski (1990)	Direct injection	Yes	NA	NA	NA
Sugawara (2008)	Endovascular perforation	Yes	NA	NA	Yes
Veelken (1995)	Endovascular perforation	NA	NA	Yes	NA
Gules (2002)	Direct injection	Yes	NA	NA	No focal neurological deficit
Doczi (1986)	Direct injection	NA	NA	NA	NA

*NA* not available

**Conclusion** Currently we don’t have a murine model of SAH meeting the diagnostic DCI criteria retained in humans. The development of such a model in agreement with the pathophysiological data known to humans remains a relevant objective with the aim of translational research more efficient.

**Competing interests** None.

**References**

1. Stroke. 2010;41(10):2391–5.
2. Ann Intern Med. 2014;151(2):264–9.

#### P16 Brain death in pediatric intensive care: department’s experience

##### Anwar Armel^1^, Rchi Abdou^1^, Samira Kalouch^2^, Khalid Yaqini^2^, Aziz Chlilek^2^

###### ^1^Anesthésie réanimation, CHU Ibn Rochd, Casablanca, Morocco; ^2^Service de réanimation pédiatrique, Chu Ibn Rochd, Casablanca, Morocco

####### **Correspondence:** Anwar Armel - armelanwar@gmail.com

*Annals of Intensive Care* 2017, **7(Suppl 1)**:P16

**Introduction** Pediatric transplants suffer from organs shortage. Children’s potential organs donors should be enhanced by awareness of the healthcare team and their families, given the socio-economic impact of the transplant. Our study’s aim was to evaluate our potential organs donors from deceased patients in pediatric intensive care during 2015.

**Materials and methods** We led a retrospective study in pediatric intensive care unit of UHC Ibn Rochd child hospital Abderahim Elharouchi of Casablanca, over 1 year from 1 January 2015 until 31 December 2015. Were Included all patients in clinical brain death. Our study excluded newborns.

Statistical analysis used the epi-info test with significance level P ˂0.05.

**Results** 25 cases were collected, 12% of deaths during 2015, with average age of 7, 32 ± 6, sex ratio was M/W 1.5, the average weight of our population was 34, 72 ± 32 kg, reason for admission was head injury following an PRA for 8 cases, cranial traumatism following defenestration for 2 cases, stroke for 10 cases, and epilepsy for 6 cases.

The Glasgow Average on admission was 6.88 ± 3, mean hospital duration was 6.75 days. All patients were ventilated on admission, use of vasoactive drugs were needed for 20 patients.

Brain death diagnosis was made by two intensive anesthetist doctors for each patient looking for brain death clinical signs. Cerebral angiography was made for 10 patients. The procedure of organ donation has been discussed in 17 cases, parents have accepted donation in two cases, refusal of both parents in 9 cases, refusal of one parent in 6 cases. In 8 cases the patient died before starting the procedure.

We successfully removed both kidneys and liver for our first donor and just one kidney and cornea for the second one.

**Discussion** In front of persisting imbalance between the needs and the number of grafts, removal of pediatric organs should be enhanced by optimizing children potential donors and their families management. It is important to encourage the involvement of a health care team and humanly participate with the coordination teams to deal with parents and dare to seek their consent to donate.

**Conclusion** This study shows out a significant pool of potential organ donors in pediatric intensive care. Hence the need to lead out activities within our society to make of them effective donors.

**Competing interests** None.

#### P18 Interest of lung ultrasound in an intensive care unit

##### Walid Sellami^1^, Zied Hajjej^1^, Soumaya Ben Yedder^1^, Iheb Labbene^1^, Mustapha Ferjani^1^

###### ^1^Department of critical care medicine and anesthesiology, Military Hospital of Tunis, Tunis, Tunisia

####### **Correspondence:** Walid Sellami - drsellamiwalid@yahoo.fr

*Annals of Intensive Care* 2017, **7(Suppl 1)**:P18

**Introduction** Interest in bedside lung ultrasound in the intensive care unit has exploded in recent years [1]. The aim of this study was to evaluate the contribution of lung ultrasound in an intensive care unit and its place as a diagnostic tool and therapeutic implication.

**Patients and methods** Retrospective study extending over 6 months. It included ICU patients who underwent lung ultrasound.

**Results** Forty patients underwent a lung ultrasound due to various respiratory etiologies: 60% patients for acute respiratory failure, 30% for ultrasound-guided pleural punctures, 20% for exploration of a white lung on chest radiograph. Ultrasonography consists in exploration the chest over the six regions. Of the 24 (60%) acute respiratory failure, the review found: 15 (62.5%) fluid pleural effusions, 2 (8%) pneumothorax, 4 (16%) alveolar-interstitial syndrome and 3 (13.5%) pulmonary condensation. Clinical and gasometric improvement was observed in 70% of patients. Lung ultrasound has allowed a change in management in 43% of patients and has provided new information in 72% of cases and led to successful ultra sound guided pleural drainage.

**Discussion** Lung ultrasound has allowed a major improvement of routine practice in intensive care unit. Once the appropriate equipment and training acquired, lung ultrasound has advantages: Safety, speed, acuity, reduced costs and increased patient comfort

**Conclusion** Responding immediately to questions for which only sophisticated approaches were used, lung ultrasound untangles these daily problems in the emergency and intensive care.

**Competing interests** None.

**Reference**

1. Rouby JJ. Intensive Care Med.2000;26:1046–56.

#### P19 How do we set assist control ventilation? Tidal volumes and relations to predicted body weight

##### Alexandre Tonnelier^1^, Fabien Hervé^1^, Guillaume Halley^1^, Jean-Luc Frances^1^, Mickael Moriconi^1^

###### ^1^finistere, C.H. de Cornouaille, Quimper, France

####### **Correspondence:** Alexandre Tonnelier - a.tonnelier@ch-cornouaille.fr

*Annals of Intensive Care* 2017, **7(Suppl 1)**:P19

**Introduction** The use of large tidal volume ventilation can be deleterious and lead to Ventilator Induced Lung Injury. It has been suggested that Lung-protective ventilation using low tidal volume should be used in all critically ill patients. We conducted a study to evaluate the tidal volumes related to body weight prescribed during Assist Control Mechanical Ventilation (ACV) in our Unit and to determine the proportion of patients receiving Low Tidal Volume Ventilation (LTVV).

**Patients and methods** Retrospective study conducted in a single 12 bed adult intensive care unit (ICU). Patients requiring mechanical ventilation admitted from January to June 2014 were included. Medical files were reviewed to determine the minimal and maximal tidal volumes related to predicted body weight (Vt min and Vt max) delivered during Assist Control Ventilation. LTVV was defined as Vt ≤ 8 ml/kg of predicted body weight (PBW).

**Results** We included 129 patients (63.5% men) with a median age of 64 years (IQR 52.5–71), a mean SAPS II Score of 46 ± 16 and a median length of mechanical ventilation of 6 days (IQR 2–14.5). Patients were predominantly non-surgical (77.5%) and 18.6% had a diagnosis of ARDS. Mean height was 168.4 ± 9.3 cm, actual and predicted body weight were 75.5 ± 21.7 kg and 63.1 ± 9.8 kg. Median Vt max was 7.9 ml/kg (IQR 7.3–8.9) and 72 patients (56%) had permanent LTVV (Vt max ≤ 8 ml/kg). Median Vt min was 7.4 ml/kg (IQR 6.9–8.2). Women had Vt max and Vt min significantly higher than men [median respectively 9.4 (IQR 8.3–9.8) vs 7.4 ml/kg (IQR 7–8) and 8.4 (IQR 7.5–9.3) vs 7.2 ml/kg (IQR 6.7–7.6); p < 0.0001] (Fig. 11). Only 17% of women had permanent LTVV versus 78% of men.

**Fig. 11:**
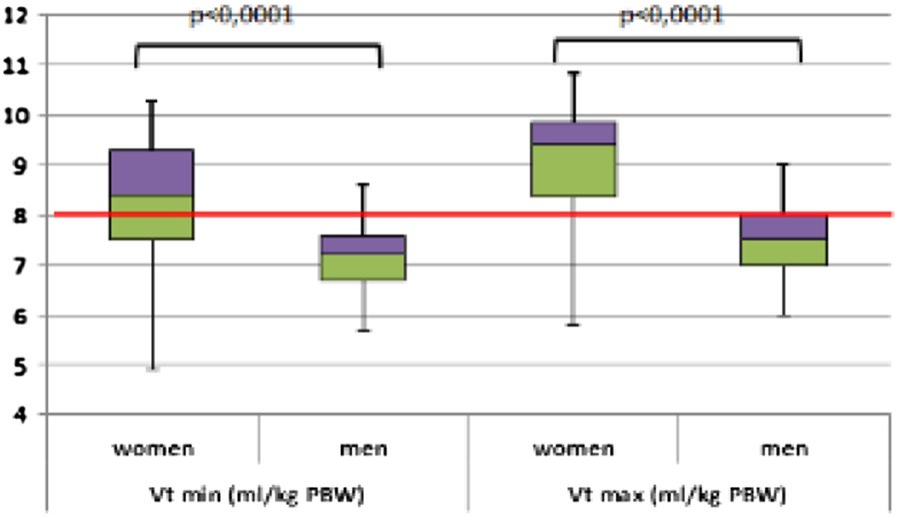
Box-plot of tidal volumes according to gender

**Discussion** Even though the benefit of LTVV applied for all ICU patients is still to be demonstrated, our results show that women are exposed to much more “aggressive” mechanical ventilation with potentially harmful consequences. LTVV seems to be achievable for all intensive care patients since 78% of men admitted in our unit are ventilated with maximal Vt ≤ 8 ml/kg of predicted body weight.

**Conclusion** A large proportion of patients in our unit are ventilated with LTVV but Mechanical Ventilation could be another example of gender discrimination. Particular attention should be paid when setting tidal volume for women in ICU.

**Competing interests** None.

**References**

1. Santamaria JD, Tobin AE, Reid DA, others. Do we practise low tidal-volume ventilation in the intensive care unit? A 14-year audit. Crit Care Resusc. 2015;17(2):108.
2. Lellouche F, Lipes J. Prophylactic protective ventilation: lower tidal volumes for all critically ill patients? Intensive Care Med. 2013;39(1):6–15.

#### P20 Respiratory mechanics of passive mechanically ventilated ICU patients

##### Mathieu Saoli^1^, Aude Garnero^2^, Didier Demory^3^, Jean Michel Arnal^3^

###### ^1^VAR, Hospital Sainte Musse, Toulon, France; ^2^Var, Hopital Sainte Musse, Toulon, France; ^3^Réanimation polyvalente, Hôpital Sainte-Musse, Toulon, France

####### **Correspondence:** Mathieu Saoli - mathieu.saoli@gmail.com

*Annals of Intensive Care* 2017, **7(Suppl 1)**:P20

**Introduction** Bench test studies are performed to evaluate or compare ventilators. Setting of respiratory mechanics of the lung model should simulate realistic conditions [1]. However, there is no standard for respiratory mechanics properties for normal lung, COPD, and ARDS. Consequently, bench studies use different respiratory mechanics variable which have a big impact on results [1]. This prospective observational study measured respiratory mechanics of passive ventilated ICU patients in order to provide realistic values for bench test studies.

**Patients and methods** This study was performed in adult mixed ICU of Sainte Musse hospital in Toulon between May 2015 and September 2016. Patients deeply sedated and passively ventilated were included. Exclusion criteria were mixed lung condition, presence of chest tube, prone position, and severe hemodynamic impairment. Patients were classified according to standard definition in: normal lungs, COPD or ARDS. Patients were ventilated in INTELLIVENT-ASV^®^ mode and positioned in semi-recumbent. Respiratory mechanics variables were measured once per patient during the first 24 h after ICU admission together with arterial blood gases. Airway pressure and flow was measured by a proximal pneumotachograph. Static compliance (CSTAT) and inspiratory resistances (RINS) were measured using the least square fitting method [2]. CSTAT was also calculated as the ratio between tidal volume and driving pressure. Expiratory time constant (RCEXP) was measured as the ratio between volume and flow at 75% of maximum expiratory flow and calculated as the product of CSTAT MEAS and RINS. Anova was performed to compare results between each lung condition. Data are presented as median [25th–75th quartiles].

**Results** Two hundred fifty-seven patients were included: age: 66 [54–74] years; BMI: 25 [22–28] kg/m^2^, SAPS II: 56 [44–66]. The main results are shown in Table 12.

**Table 12:** See text for description

	Normal lung (n = 95)	All ARDS (n = 131)	Mild ARDS (n = 35)	Moderate ARDS (n = 64)	Severe ARDS (n = 32)	COPD (n = 28)	P ANOVA
CSTAT MEAS (mL/cmH_2_O)	46 (42–59)	34 (29–45)	37 (30–46)	35 (29–43)	33 (29–43)	50 (37–70)	<.001
CSTAT CALC (mL/cmH_2_O)	52 (43–64)	40 (32–49)	41 (32–51)	41 (33–49)	38 (30–45)	56 (42–72)	<.001
RINS (cmH_2_O s/L)	13 (11–15)	12 (10–14)	11 (9–13)	12 (10–15)	11 (9–12)	22 (16–33)	<.001
RCEXP MEAS (s)	0.60 (0.52–0.72)	0.47 (0.41–0.57)	0.46 (0.41–0.54)	0.47 (0.39–0.57)	0.45 (0.38–0.57)	1.04 (0.65–1.82)	<.001
RCEXP CALC (s)	0.62 (0.51–0.80)	0.41 (0.32–0.55)	0.44 (0.35–0.57)	0.43 (0.30–0.58)	0.37 (0.30–0.51)	1.17 (0.69–1.70)	<.001

**Conclusion** This study reports clinically-based values of respiratory mechanics for passive mechanically ventilated ICU patients. These results should help bench studies design to simulate realistic conditions.

**Competing interests** None.

**References**

1. Chatburn R. Simulation studies for device evaluation. Respir Care 2014;59(4):e61–6.
2. Iotti GA, Braschi A, Brunner JX, Smits T, Olivei M, Palo A, Veronesi R. Respiratory mechanics by least squares fitting in mechanically ventilated patients: applications during paralysis and during pressure support ventilation. Intensive Care Med 1995;21:406–13.

#### P21 Usefulness of a respiratory comfort scale in titration of the NAVA (Neurally Adjusted Ventilatory Assist) level for patients under spontaneous mechanical ventilation: A physiological study

##### Bertrand Canoville^1^, Cédric Daubin^1^, Jennifer Brunet^2^, Damien Du Cheyron^1^, Frédéric Lofaso^3^, Nicolas Terzi^4^

###### ^1^Réanimation médicale, Centre Hospitalier Universitaire de Caen, Caen, France; ^2^Anesthésie, Centre Hospitalier Universitaire de Caen, Caen, France; ^3^Service d’explorations fonctionnelles respiratoires, Hôpital Raymond-Poincaré (AP-HP), Garches, France; ^4^Service de réanimation médicale, Clinique de Réanimation Médicale, Grenoble, France

####### **Correspondence:** Bertrand Canoville - canoville-b@chu-caen.fr

*Annals of Intensive Care* 2017, **7(Suppl 1)**:P21

**Introduction** Although Neurally Adjusted Ventilatory Assist (NAVA) is currently used in many intensive care units, the best method for setting the optimal level of assistance in this proportional ventilatory mode is not yet established. In order to determine a method based on aiming the best respiratory comfort of the patient, the authors studied the impact of several levels of assist in NAVA, on the respiratory behaviour and comfort, of patients undergoing weaning of the mechanical ventilation.

**Patients and methods** This study was single-center prospective, open, randomised, of current care. Patients staying in a critical care unit, undergoing invasive mechanical ventilation for an acute respiratory failure, and recovering spontaneous ventilation, were included. For every patient, an optimal level of assistance in pressure-support ventilation was determined and called AI 100, with which an equivalent level of assistance in NAVA called NAVA 100 was set. From this level, six other NAVA levels were determined, by increase or decrease of 25% stepwise, respectively called NAVA 25, NAVA 50, NAVA 75, NAVA 125, NAVA 150 and NAVA 175, and applied in a randomized order. For every situation, after obtaining a stability of the end-tidal carbon dioxide pressure (ETCO2), pressure, volume, flow, and electromyographic activity of the diaphragm (Edi) were recorded during 5 min. An arterial gazometry was performed, and respiratory comfort was assessed by using the dyspnea numeric scale, the Multi-dimensional Dyspnea Profile (MDP) by Banzett, and the Respiratory Distress Observational Scale (RDOS). All parameters were compared between ventilatory modes, by using variance analysis ANOVA.

**Results** Ten patients were included. Between NAVA levels, no difference was observed regarding tidal volume (Vt, p = 0.143) and arterial carbon dioxide pressure (PaCO_2_, p = 0.141). The Ti/Ttotneu ratio and AUC Editot (the area under curve of Edi superior to trigger) raised significantly for NAVA 25, the lowest NAVA level, respectively p = 0.005 and p < 0.001. The dyspnea numeric scale and MDP by Banzett showed no significant difference along the NAVA levels, respectively p = 0.412 and p = 0.107. However, RDOS score was significantly higher for the low levels of assistance NAVA 25 and NAVA 50; p < 0.001.

**Conclusion** With variation of the NAVA level, our patients showed a respiratory behaviour similar to the findings described previously in the litterature. The level of assistance did not seem to influence the patient’s feeling of dyspnea, despite an obvious raising of the ventilatory demand. However, Ti/Ttotneu ratio, AUC Editot and RDOS clinical score appear as potential useful tools to detect under-assistance issues in patients undergoing NAVA.

**Competing interests** None.

#### P22 Usefulness of full outline of unresponsiveness score to predict extubation failure in intubated critically-ill patients

##### Hassen Ben Ghezala^1^, Salah Snouda^2^, Chiekh Imen Ben^3^, Moez Kaddour^2^

###### ^1^Réanimation Médicale, Hôpital Henri Mondor, Avenue du Maréchal de Lattre de Tassigny, Créteil, France, Créteil, France; ^2^Réanimation Médicale, Hopital regional zaghouan, faculté de médecine de Tunis, Zaghouan, Tunisia; ^3^Teaching department of emergency and intensive care, Regional hospital of Zaghouan, Zaghouan, Tunisia

####### **Correspondence:** Hassen Ben Ghezala - hassen.ghezala@gmail.com

*Annals of Intensive Care* 2017, **7(Suppl 1)**:P22

**Introduction** The aim of the study is to assess the usefulness of the Full Outline of Unresponsiveness (FOUR) score in predicting extubation failure in critically-ill intubated patients admitted with disturbed level of conscious in comparison with the Glasgow Coma Scale (GCS).

**Materials and methods** All intubated critically-ill patients with disturbed level of consciousness were assessed using both the FOUR score and the GCS. The FOUR score and the GCS were compared regarding their predictive value for successful extubation at 14 days after intubation as a primary outcome measure. The 28-day mortality and the neurological outcome at 3 months were used as secondary outcome measures.

**Results** Eighty-six patients were included. Median age was 63 [50–77] years. Sex-ratio (M/F) was 1.46. Median GCS was 7 [3–10] while median FOUR score was 8.5 [2.3–11]. A GCS ≤ 7 predicted the extubation failure at 14 days after intubation with a sensitivity of 88.5% and specificity of 68.3% (Youden index = 0.57 95% CI [0.35–.7]) whereas a FOUR score <10 predicted the same outcome with a sensitivity of 80.8% and a specificity of 81.7% (Youden index = 0.62 95% CI [0.44–0.77]). The AUC was significantly higher with the FOUR score than with GCS (respectively 0.867 95% CI [0790–0.944] and 0.832 95% CI [0.741–0.923]; p = 0.014). Both scores had similar accuracy for predicting 28-day mortality and neurological outcome at 3 months.

**Conclusion** The FOUR score is superior to the GCS for prediction of successful extubation of intubated critically-ill patients.

**Competing interests** None.

#### P23 T-tube or pressure support ventilation for the spontaneous breathing trial

##### Islem Ouanes^1^, Mahdi Marzouk^1^, Dhouha Ben Braiek^1^, Saousen Ben Abdallah^1^, Zeineb Hammouda^1^, Fahmi Dachraoui^1^, Kmar Hraiech^1^, Sabrine Nakaa^1^, Ali Ousji^1^, Ali Adhieb^1^, Abdelwaheb M’ghirbi^1^, Lamia Ouanes-Besbes^1^, Fekri Abroug^1^

###### ^1^Réanimation polyvalente, CHU Fattouma Bourguiba, Monastir, Tunisia

####### **Correspondence:** Islem Ouanes - ouanes.islem@gmail.com

*Annals of Intensive Care* 2017, **7(Suppl 1)**:P23

**Introduction** Spontaneous breathing trial (SBT) is used to test patient’s readiness for separation from the ventilator. The most appropriate method for SBT (t-tube vs pressure support ventilation, PSV) is still a matter of debate. The aim of the present study was to assess SBT and patients outcomes with these two methods in a Tunisian ICU.

**Patients and methods** This was a cohort study including patients consecutively admitted between 1st July 2014 and 31st August 2016 and ventilated more than 48 h, who underwent their first SBT according to clinical criteria. SBT was performed in our ICU with T-tube before March 2016 and with PSV since then. Clinical characteristics and outcomes were compared between both groups. A multivariate analysis with adjustment on variables significantly different at baseline was made for the prediction of extubation success.

**Results** During the study period 84 patients under went first SBT (55 patients with T-tube and 29 with PSV). Table 13 summarizes patients characteristics and outcomes.

**Table 13:** Characteristics and outcomes in the two SBT modalities groups before adjustment

	PSV (n = 29)	T-tube (n = 55)	*p*
Age (med [IQR])	67 [48–74]	55 [36–67]	*0.044*
Gender (M/F)	21/8	32/23	0.240
SAPS III (med [IQR])	75 [66.5–80.5]	67 [60–77]	*0.021*
SOFA (med [IQR])	8 [7–10]	7 [5–10]	0.172
Reason for mechanical ventilation			
ARF on CRF [n (%)]	14 (48.3)	16 (29.1)	0.101
ARF de novo [n (%)]	8 (27.6)	17 (31)	
Septic shock [n (%)]	5 (17.2)	6 (11)	
Neurologic [n (%)]	2 (6.9)	16 (29.1)	
Ventilation duration before 1st SBT (days) med [IQR]	5 [3–8]	5 [3–8]	0.854
SBT outcome			
SBT duration, med [IQR]	60 [30–80]	60 [30–90]	0.489
SBT success [n (%)]	20 (68.9)	41 (74.5)	0.614
Reintubation [n (%)]	3 (10.3)	5 (9.1)	0.852
ICU LOS (days) (med [IQR])	16 [8.5–27.5]	12 [7–30]	0.486
ICU mortality [n (%)]	6 (20.7)	10 (18.2)	0.778

Multivariate analysis showed no relation between SBT modality and extubation success (extubation without reintubation in the following 48 h) (OR 0.89, 95% CI 0.33–2.38). We also matched 28 patients in PSV group with 28 others from T-tube group (on age, gender and SAPS III), and observed that the rate extubation success was not different (53.6% T-tube vs 57.1% PSV, p = 1.000). ICU mortality was significantly higher among reintubated patients than in successfully extubated patients (75 vs 11.3%, p < 0.001).

**Conclusion** In our cohort, SBT with PSV or T-tube provided comparable SBT results and clinical outcomes.

**Competing interests** None.

#### P25 First-year experience of a French mechanical ventilation weaning centre

##### Françoise Haniez^1^, Hélène Jaillet^1^, Henri Maas^1^, Pierre Andrivet^1^, Christian Darné^1^, François Viau^1^

###### ^1^91, C.H. Bligny, Briis-sous-Forges, France

####### **Correspondence:** Françoise Haniez - f.haniez@chbligny.fr

*Annals of Intensive Care* 2017, **7(Suppl 1)**:P25

**Introduction** Economic pressure to maximize resource utilization has resulted in the creation of post-ICU care centers dedicated to patients with weaning failure.

**Patients and methods** Prospective observational cohort study of patients admitted to a French mechanical ventilation centre located in Essonne; first year experience (January 2015 to December 2015). The 12-bed unit is staffed by nurses with special pulmonary and ventilation expertise, and it features 24-h respiratory therapy supervision and non invasive monitoring (that is ventilator alarms) with signal outputs at each bedside. Pulmonary and critical care specialists are in charge of patients’ treatments and weaning plans. Dietitians and physical therapists are all part of the care team.

**Participants** 106 patients admitted with a median age of 66.9 years; 60% were male.

70 patients (66%) had tracheostomy before they were transferred in our unit, 54 of them with prolonged mechanical ventilation;

27 patients (25.5%) had non invasive ventilation;

9 patients (8.5%) were dependent on high oxygen flow (>6 l/mn) without mechanical ventilation. All patients came from the ICU.

The duration of ICU stay preceding admission was 236 days.

**Results** The duration of total unit stay was 29 days (6–74 days). The mean IGS score at admission was 29. The mortality rate was 4.7% (3 patients with fibrosis dependent on high oxygen flow, 2 patients—1 with polymetastatic cancer—with tracheostomy and 24 h/24 ventilator support). 16 patients (15.1%) with worsening conditions returned to the ICU. Among the 85 remaining patients (80.2%), 46 were transferred to a rehabilitation centre, 10 were transferred in acute care hospital in step-down unit, 29 were discharged directly to home.

Weaning success was observed in 3 patients with NIV at admission and 24 were educated to self use of NIV.

Of the 16 patients without mechanical ventilation at admission only 3 patients with tracheostomy were discharged with long-term tracheostomy.

Of the 54 patients with invasive mechanical ventilation and tracheostomy at admission:

- 30 were weaned and decannulated
- 7 were discharged with long-term tracheostomy without ventilation
- 14 with long-term tracheostomy were only partially weaned from invasive ventilation
- 1 with tracheostomy and 24/24 ventilator support.

Of the patients who were dependent on high oxygen flow (>6 l/mn) at admission 4 were weaned from high oxygen flow at discharge.

**Discussion** The current data demonstrate that, in our centre, most of the patients with weaning failure can be successfully weaned. Despite a prolonged ICU stay before referral, weaning outcomes were similar to those reported in other countries and organizational models.

**Conclusion** Patients with weaning failure should be considered for transfer to a specialist weaning centre which can demonstrate favourable short-term outcome.

**Competing interests** None.

**Reference**

1. Mifsud Bonnici D, Sanctuary T, Warren A, et al. Prospective observational cohort study of patients with weaning failure admitted to a specialist weaning, rehabilitation and home mechanical ventilation centre. BMJ Open. 2016;6:e010025. doi:10.1136/bmjopen-2015-010025.

#### P26 COPD patients with acute exacerbation in a Tunisian intensive care unit: What is our practice?

##### Hassen Ben Ghezala^1^, Salah Snouda^2^, Chiekh Imen Ben^3^, Moez Kaddour^2^

###### ^1^Réanimation Médicale, Teaching Department of Emergency and Intensive Care, Hospital of Zaghouan, Zaghouan, Tunisia, Créteil, France; ^2^Réanimation Médicale, Hopital regional zaghouan, faculté de médecine de Tunis, Zaghouan, Tunisia; ^3^Teaching department of emergency and intensive care, Regional hospital of Zaghouan, Zaghouan, Tunisia

####### **Correspondence:** Hassen Ben Ghezala - hassen.ghezala@gmail.com

*Annals of Intensive Care* 2017, **7(Suppl 1)**:P26

**Introduction** Chronic Obstructive Pulmonary Disease (COPD) exacerbations are now the third leading cause of mortality all over the world and in Tunisia. There still are many controversies in their management and mainly the indications of antibiotic treatment and corticotherapy. In Tunisia and despite many valuable studies, only few studies were focused on the “Tunisian” daily practice in case of acute and severe exacerbations of COPD. Thus; we decided to perform this study. We aimed to describe the clinical characteristics of our patients, the blood gas exchange findings, the medical treatment, the ventilator support and to analyze their outcome.

**Materials and methods** It was a Single-center retrospective study performed in the teaching department of emergency and intensive care medicine in Zaghouan in Tunisia conducted between 1st January 2015 and 31th December 2015. All patients admitted in our ICU for acute exacerbation of COPD were enrolled in the study. Anamnestic characteristics, primary diagnosis, mode of mechanical ventilation were noted. The results of laboratory tests, blood gas data and the outcome of patients were also collected.

**Results** 40 patients were included in the study. The mean age was 68 ± 11 years with a sex ratio of 4 (32 males and 8 females). The mean SAPS II score was 35 ± 10 with an APACHE II mean score of 22 ± 5. The most common cause of COPD in our patients was tobacco smoke with a mean consumption of 32 ± 10 pack-year history of smoking. The most frequent COPD condition in our patients was chronic bronchitis (n = 29; 72.5%). Six patients received home oxygen therapy. Three patients had lung emphysema and one patient had both restrictive and obstructive syndrome. At admission, the mean Glasgow coma scale score was 12 ± 3. The mean Ph was 7.21 ± 0.13 with a mean bicarbonate level of 32 ± 10 mEq/l. Lung infection was the most common cause of exacerbation (n = 26; 65%). The other recorded causes were: pulmonary edema (n = 3), pulmonary embolism (n = 2) and pleural effusion (n = 5). Twenty-two patients (55%) received systemic corticotherapy and 33 patients received antibiotic therapy. Sixteen patients (40%) did not receive bronchodilator medicines. Fifteen patients (37%) required noninvasive ventilation (NIV). Eighteen patients (45%) was intubated and required invasive mechanical ventilation. Ventilatory support was not necessary in the other seven patients (17.5%). The mean duration of hospital stay in our ICU was 111 ± 98 h. Sixteen patients died in our ICU with a mortality rate of 40%. The most frequent causes of death were septic shock (n = 5), acute respiratory distress syndrome and multiorgan failure (n = 2). Four patients were transferred and twenty patients were discharged from hospital. Only SAPS II was independently associated with poor outcome (p < 0.001).

**Conclusion** Our patients may be classified severe COPD according to GOLD recommendations. There is a big gap between our daily practice and the international recommendations. We still have a widespread use of corticotherapy and antibiotic therapy. We need effective interventions to implement existing evidence-based guidelines into daily practice in exacerbations of COPD.

**Competing interests** None.

#### P27 Serum bicarbonate levels change during ICU stay for hypercapnic COPD exacerbation and impact on long-term survival

##### Islem Ouanes^1^, Saousen Ben Abdallah^1^, Kmar Hraiech^1^, Ali Ousji^1^, Sabrine Nakaa^1^, Ali Adhieb^1^, Dhouha Ben Braiek^1^, Abdelwaheb M’ghirbi^1^, Zeineb Hammouda^1^, Fahmi Dachraoui^1^, Fekri Abroug^1^, Lamia Ouanes-Besbes^1^

###### ^1^Réanimation polyvalente, CHU Fattouma Bourguiba, Monastir, Tunisia

####### **Correspondence:** Islem Ouanes - ouanes.islem@gmail.com

*Annals of Intensive Care* 2017, **7(Suppl 1)**:P27

**Introduction** Acute exacerbation of chronic obstructive pulmonary disease (AECOPD) is linked to morbidity and mortality increase; high bicarbonate levels are linked to chronic hypercapnia and hypoventilation in this population. The consequences of bicarbonate levels change during ICU stay are not well studied. The aim of the present study is to investigate the association between bicarbonate concentration change during ICU stay for AECOPD and long-term survival.

**Patients and methods** In a prospectively collected database including consecutive patients admitted between 2000 and 2012 for hypercapnic COPD exacerbation in our ICU, we calculated bicarbonate concentration change during ICU stay as follows: ΔHCO^3−^ = [HCO^3−^] at admission − [HCO^3−^] at discharge. Patients were split into 3 groups:

- Group 1: Decreased bicarbonate levels: ΔHCO^3−^ ≤ −5 mmol/l,
- Group 2: Small change in bicarbonate levels: −5 < ΔHCO^3−^ < +5 mmol/l,
- Group 3: Increased bicarbonate levels: ΔHCO^3−^ ≥ +5 mmol/l.

Long-term patient’s status (surviving or deceased) was checked by consulting the register of civil status.

**Results** During the study period 440 patients were consecutively admitted for hypercapnic COPD exacerbation (84.5% males, median age: 68 years, median pH at admission: 7.28 and median HCO^3−^: 30.6, NIV was the first ventilation modality in 65% and survival at ICU discharge: 84.3%). Blood gas analyses (at ICU admission and discharge), and long-term vital status were available in 239 patients (survival at a median follow-up of 7 years was 31.2%). Bicarbonate levels change were inversely associated with the long-term survival (Table 14; Fig. 12).

**Table 14:** Bicarbonate levels change and long-term survival

	ΔHCO^3−^ ≤ −5 (n = 53)	−5 < ΔHCO^3−^ < +5 (n = 142)	ΔHCO^3−^ ≥ +5 (n = 44)	p
ΔHCO^3−^ [med (IQR)]	−8.2 (−11.4;−6.05)	0 (−2.1; 2.05)	7.2 (6.2; 9.8)	<0.001
Long-term survival [n (%)]	26 (49.1)	57 (40.1)	10 (22.7)	0.027

**Fig. 12:**
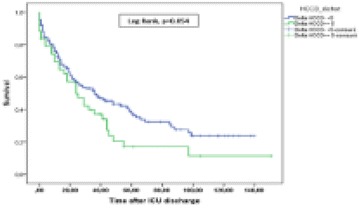
Kaplan Meier curve, bicarbonate levels change and long-term survival

**Conclusion** Our study suggests that bicarbonate levels change during ICU stay for hypercapnic COPD exacerbation could be considered as prognostic factor.

**Competing interests** None.

#### P28 Dyspnea in patients with acute respiratory failure requiring non invasive ventilation in the ICU: prevalence, factors and prognosis—a prospective cohort study

##### Laurence Dangers^1^, Claire Montlahuc^2^, Achille Kouatchet^3^, Samir Jaber^4^, Ferhat Meziani^5^, Sébastien Perbet^6^, Sylvie Chevret^7^, Elie Azoulay^8^, Alexandre Demoule^9^

###### ^1^Service de pneumologie et réanimation médicale, Groupe Hospitalier Pitié Salpêtrière, Paris, France; ^2^Department of informatics and biostatistics, Saint Louis Hospital, Paris, France; ^3^Service de Réanimation médicale et Médecine hyperbare, Centre Hospitalier Universitaire d’Angers, Angers, France; ^4^DAR B, Hôpital Saint Eloi, Montpellier, France; ^5^Réanimation médicale, Nouvel Hôpital Civil, CHU Strasbourg, Strasbourg, France; ^6^Service de réanimation adultes, C.H.U. Estaing, Clermont-Ferrand, France; ^7^Service de biostatistique et information médicale, Hôpital Saint-Louis, Paris, France; ^8^Réanimation médicale, Hôpital Saint-Louis, Paris, France; ^9^Service de pneumologie et réanimation médicale, Groupe Hospitalier Pitié-Salpêtrière, Paris, France

####### **Correspondence:** Laurence Dangers - laudangers@gmail.com

*Annals of Intensive Care* 2017, **7(Suppl 1)**:P28

**Introduction** Dyspnea is frequent and intense in intubated patients. It is one of the worse experiences of the intensive care unit (ICU) stay and is associated with adverse outcomes. Despite the increasing use of non invasive ventilation (NIV) as the cornerstone therapy of acute respiratory failure (ARF), little attention has been given to that threatening sensation.

The objectives were: (1) To quantify the prevalence and intensity of dyspnea in patients receiving NIV for ARF, at admission and in response to NIV; (2) To examine the factors associated with dyspnea; (3) To investigate the impact of dyspnea on NIV success or failure and on the quality of life and post-ICU burden.

**Patients and methods** Second analysis of a prospective observational cohort study in patients who received NIV for ARF in 54 ICUs in France and Belgium, in 2010/2011. Dyspnea measurement was assessed with a modified Borg scale at admission and in response to the first NIV session. Patients with a dyspnea intensity <4 (defined as light to moderate) were compared to those with dyspnea intensity ≥4 defined (moderate to severe).

**Results** Among the 426 patients included the median dyspnea on admission was 4 [3–5] and decreased to 3 [2–4] in response to the first NIV session (p = 0.001). Moderate to severe dyspnea in response to NIV was independently associated with severity as assessed by the SOFA (OR 1.09, p = 0.023), anxiety (OR 1.84, p = 0.009), leaks (OR 2.15, p = 0.002) and poor NIV tolerance (OR 2.01, p = 0.012). In addition, moderate to severe dyspnea was an independent risk factor of NIV failure (OR 2.24, p = 0.001). Finally, dyspnea was associated with higher length of stay and mortality (7 vs. 18%, p = 0.001) but was not associated with higher post ICU burden and quality of life.

**Conclusion** In patients receiving NIV, dyspnea is frequent, intense and exposes patients to a higher risk of NIV failure. NIV is associated with poorer outcome.

**Competing interests** None.

#### P29 Prevalence of anemia in COPD exacerbations of and impact on short-term prognosis

##### Islem Ouanes^1^, Ali Adhieb^1^, Sabrine Nakaa^1^, Kmar Hraiech^1^, Dhouha Ben Braiek^1^, Zeineb Hammouda^1^, Saousen Ben Abdallah^1^, Fahmi Dachraoui^1^, Ali Ousji^1^, Abdelwaheb M’ghirbi^1^, Lamia Ouanes-Besbes^1^, Fekri Abroug^1^

###### ^1^Réanimation polyvalente, CHU Fattouma Bourguiba, Monastir, Tunisia

####### **Correspondence:** Islem Ouanes - ouanes.islem@gmail.com

*Annals of Intensive Care* 2017, **7(Suppl 1)**:P29

**Introduction** COPD patients have often polyglobulia because of associated hypoxemia especially in patients at the stage of chronic respiratory failure. Little is known about the prevalence of anemia and its impact on prognosis in patients with severe AECOPD admitted to ICU. The aim of this study was to determine the prevalence of anemia in AECOPD patients and its impact on prognosis.

**Patients and methods** In a prospectively collected database including consecutive patients admitted between 2007 and 2015 for AECOPD in our ICU, we retrospectively assessed hemoglobin levels at ICU admission. Anemia was defined according to WHO criteria: Hb < 13 g/dl in males; Hb < 12 g/dl in females. Continuous variables expressed as median (25–75 percentiles interquartile ranges, IQR) and compared with the Mann–Whitney test.

**Results** The cohort included 210 patients (median age 67, median pH 7.30, 87.6% males, NIV as first ventilator mode in 86.2%). Anemia was observed in 77 of the 210 patients (36.6%). Median haemoglobin levels were at 10.7 and 14.5 g/dl, in patients with and without anemia, respectively, Table 15 summarizes characteristics and clinical outcomes in the two study groups.

**Table 15:** Characteristics and clinical outcomes in patients with and without anemia

	Anemia− (n = 133)	Anemia+ (n = 77)	p
Age [med (IQR)]	64 (57–72)	71 (65–75)	<0.001
Male [n (%)]	117 (88)	67 (87)	0.831
Time course of COPD (years) [med (IQR)]	7 (4–12)	8.5 (4.2–15)	0.212
pH [med (IQR)]	7.30 (7.26–7.34)	7.30 (7.27–7.35)	0.205
HCO^3−^ (µmol/L) [med (IQR)]	32.8 (28–36)	31.8 (27.7–35.5)	0.749
Creat (µmol/L) [med (IQR)]	83 (66–91)	101 (78.2–133.5)	<0.001
GFR (MDRD) [mL/min/1.73 m^2^, med (IQR)]	83 (70.1–104.5)	58.1 (42.1–76.1)	<0.001
NIV [n (%)]	114 (85.7)	67 (87)	0.839
Diabetes [n (%)]	22 (16.5)	15 (15.5)	0.580
Hypertension [n (%)]	33 (24.8)	24 (31.2)	0.337
Heart failure [n (%)]	13 (9.8)	10 (13)	0.497
Ventilation duration (days) [med (IQR)]	8 (5–11.5)	7 (4–10)	0.298
LOS (days) [med (IQR)]	10 (7–14)	9 (6–11.5)	0.138
ICU mortality [n (%)]	17 (12.8)	8 (10.6)	0.665

In multivariate analysis anemia was not identified as independent factor associated with ICU mortality.

**Conclusion** Anemia was observed in one-third of patients admitted to our ICU for severe AECOPD, and was not associated with ICU mortality.

**Competing interests** None.

#### P30 Performance of RV/LV ratio measured at exacerbation in predicting long-term mortality in severe COPD patients

##### Zeineb Hamouda^1^, Kmar Hraiech^1^, Sabrine Nakee^1^, Abdelwaheb M’ghirbi^1^, Saousen Ben Abdallah^1^, Islem Ouanes^1^, Fahmi Dachraoui^1^, Fekri Abroug^1^, Lamia Ouanes-Besbes^1^

###### ^1^Réanimation Polyvalente, CHU Fatouma Bourguiba, Monastir, Tunisia

####### **Correspondence:** Lamia Ouanes-Besbes - lamia.besbes@rns.tn

*Annals of Intensive Care* 2017, **7(Suppl 1)**:P30

**Introduction** The identification of specific prognostic factors that predict long-term mortality in COPD patients is paramount for the management of these patients. The objective of this study was to evaluate the performance of the right ventricle (RV)/left ventricle (LV) ratio measured at exacerbation requiring mechanical ventilation in predicting the long-term prognosis in patients with severe COPD.

**Patients and methods** Prospective collection of data on demographics and echocardiography in COPD patients hospitalized in the Intensive Care Unit for hypercapnic decompensation between December 2010 and March 2013. Recorded variables are: age, sex, BMI, medical history, clinical characteristics of the index exacerbation, and blood gas, biological, echocardiographic variables and the mortality at 6 years.

**Results** During the study period 108 were included in this study. The average age was 69 years (range: 43–92 years), 68% were male; the average SAPSII was 27 and average BMI was 26.8 kg/m^2^. The Mean PaCO_2_: 8.68 ± 1.69 kPa, and pH at admission was: 7.30 ± 0.04. All patients were ventilated with NIV initially. RV was frequently enlarged with a mean value of RV/LV at admission: 0.75 (±0.16). Overall mortality at 6 years was 35.5%. The ROC curve depicting the performance of RV/LV in predicting long term mortality is shown in Fig. 13 (AUC: 0.9, a cut-off of 0.7 had the best operative characteristics).

**Fig. 13:**
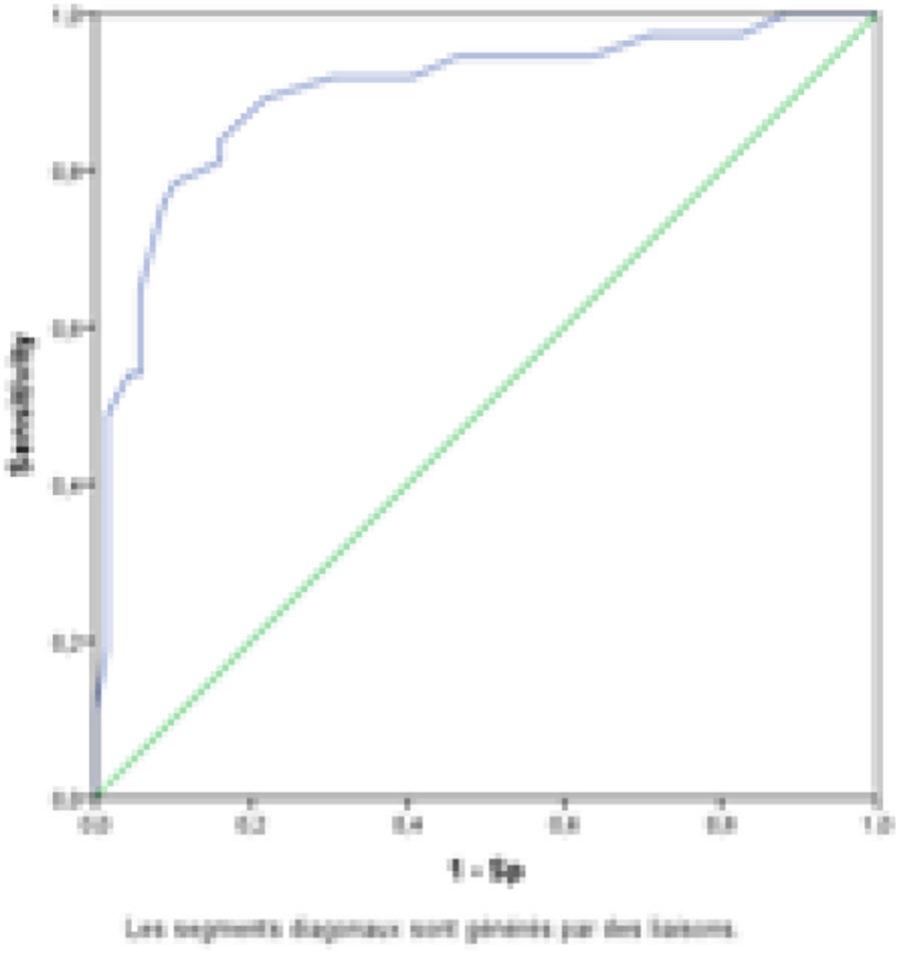
See text for description

**Conclusion** RV/LV ratio seems a good indicator of long term prognosis in patients with severe COPD

**Competing interests** None.

#### P31 Serum uric acid (SUA) as a predictor of mortality and future exacerbations of patients hospitalized for severe acute exacerbation of COPD

##### Khaoula Meddeb^1^, Ahmed Khedher^1^, Nesrine Sma^1^, Jihene Ayachi^1^, Messaouda Khelfa^1^, Nesrine Fraj^1^, Hend Ben Lakhal^1^, Hedia Hammed^1^, Raja Boukadida^1^, Hajer Hafsa^1^, Imed Chouchene^1^, Mohamed Boussarsar^2^

^1^Réanimation médicale, CHU Farhat Hached, Sousse, Tunisia; ^2^Réanimation médicale, chu farhat hached. Research laboratory n° lr14es05. Faculty of medicine, CHU Farhat Hached, Sousse, Tunisia

###### **Correspondence:** Mohamed Boussarsar - hamadi.boussarsar@gmail.com

####### *Annals of Intensive Care* 2017, **7(Suppl 1)**:P31

**Introduction** Severe acute exacerbation of COPD (AECOPD) reduces quality of life and represents a major burden for health care systems. SUA is well known to increase during hypoxia and systemic inflammation. This final product of purine degradation is one of biomarkers showing promise in the prediction of outcomes in AECOPD [1].

The aim of this study was to assess the possible role of SUA and SUA/creatinine ratio as biomarkers for the prediction of mortality and future exacerbations of patients hospitalized in intensive care unit (ICU) for severe AECOPD.

**Patients and methods** SUA levels were measured in 59 consecutive eligible patients on ICU admission for severe AECOPD between April 2015 and April 2016. Clinical and functional characteristics were compared between patients with levels below and above the median values of SUA and SUA/creatinine ratio. The primary end-point was all-cause mortality at 30 and 90 days. Secondary outcomes included duration of ICU stay, duration of mechanical ventilation and the number of AECOPD in the 6 months period after ICU discharge.

**Results** On ICU admission, age was 69 ± 11 years; SAPS II, 30 ± 9; SOFA score, was 5.0 ± 2.9. Median [IQR] pH was 7.31 [7.28–7.33]; PaCO_2_, 57 [53–69] mmHg; PaO_2_, 100 [73–203] mmHg; PaO_2_/FIO_2_, 239 ± 101. 52.5% had NIV with 45% NIV failure. Overall 71.2% had invasive mechanical ventilation and 59.3% received vasopressors.

Mean SUA was 342 ± 90 µmol/L; SUA/creatinine ratio, 4.5 ± 2.1. SUA levels were higher in patients presenting acute heart failure on admission (p = 0.035).

In addition, SUA/creatinine ratio levels were higher in patients having extended evolution time prior to ICU admission (p = 0.05) and those having high SOFA score (p = 0.05).

SUA levels were not associated with increased risk for AECOPD in the 6 months period after ICU discharge.

High SUA was an independent factor associated to circulatory failure in multivariate Cox regression analysis (HR = 12.32, p = 0.037).

In multivariate Cox regression analysis, high SUA/creatinine ratio was an independent predictor of 30-day mortality (HR = 15.03, p = 0.032), and it was also independently associated with acute heart failure (HR = 7.08, p = 0.046).

**Discussion** SUA is considered as a useful biomarker in the identification of high-risk patients admitted for mild AECOPD [1–2]. The present study suggests a role of SUA in the prediction of mortality in severe AECOPD associated with heart failure or shock requiring ICU admission

**Conclusion** High SUA was only independently associated to circulatory failure among patients with severe AECOPD. High SUA/creatinine ratio on ICU admission was associated with acute heart failure and was an independent predictor of 30-day mortality in ICU patients with severe AECOPD.

**Competing interests** None.

**References**

1. Guérin C, Burle J-F. Early rehabilitation in ICU is possible. Réanimation. 2015;24:S371–S378. doi:10.1007/s13546-014-1005-7.
2. Sricharoenchai T, Parker AM, Zanni JM, Nelliot A, Dinglas VD, Needham DM. Safety of physical therapy interventions in critically ill patients: a single center prospective evaluation of 1110 ICU admissions. J Crit Care. 2013. doi:10.1016/j.jcrc.2013.12.012.

#### P32 Long-term home non-invasive ventilation (NIV) following hypercapnic respiratory failure requiring mechanical ventilation: results of 10 years practice intensive care unit of Monastir

##### Zeineb Hamouda^1^, Sabrine Nakee^1^, Abdelwaheb M’ghirbi^1^, Kmar Hraiech^1^, Braiek Dhouha Ben^1^, Saousen Ben Abdallah^1^, Fahmi Dachraoui^1^, Islem Ouanes^1^, Fekri Abroug^1^, Lamia Ouanes-Besbes^1^

###### ^1^Réanimation Polyvalente, CHU Fatouma Bourguiba, Monastir, Tunisia

####### **Correspondence:** Lamia Ouanes-Besbes - lamia.besbes@rns.tn

*Annals of Intensive Care* 2017, **7(Suppl 1)**:P32

**Introduction** Long-term home NIV is still a matter of debate in patients with obstructive pulmonary disease. Recent studies showed a positive effects on exacerbations frequency and less obvious effects on the survival rate. The objective of this study is to assess the impact of long-term NIV in the long term in patients with persistent hypercapnia at the end of ICU admission for hypercapnic respiratory failure.

**Patients and methods** Prospective collection of data in patients hospitalized in the intensive care unit for acute hypercapnic respiratory failure requiring mechanical ventilation between October 2005 and October 2015. Patients with persistent hypercapnia and a diagnosis of COPD, obesity-hypoventilation syndrome, or obstructive sleep apnea had the following recorded data: age, sex, BMI, echocardiographic data, apnea/hypopnea index in the case a polygraphy was performed, long-term NIV duration and observance, the quality of life and the outcome with emphasis on frequency of exacerbations and survival.

**Results** During the study period, 110 patients received long-term NIV including 28 COPD, 45 OSA and 37 overlap syndrome. The average age was 65 ± 11 years, BMI: 27. The main comorbidities were: hypertension, diabetes and ischemic heart disease. The baseline pH was 7.38, baseline PaCO_2_:55 mm Hg. The mean daytime duration of NIV use: 5–6 h/day. The use of NIV in the long term is associated with a significant reduction in PaCO_2_, improved quality of life, and exacerbations’ frequency. Overall mortality at 10 years was 33%.

**Conclusion** This study shows the feasibility of NIV in long-term care of patients with persistent hypercapnia following ICU hospitalization for hypercapnic respiratory failure. It also suggests beneficial effects on PaCO_2_ levels, quality of life and the frequency of exacerbations.

**Competing interests** None.

#### P33 Incidence of thrombo embolic events on central venous catheters and predictor factors of its income

##### Kaoutar Benatti^1^, A Dafir^1^, W Aissaoui^1^, W Elallame^1^, W Haddad^1^, R Cherkab^1^, C Elkettani^1^, L Barrou^1^

###### ^1^Anesthésie réanimation, CHU Ibn Rochd, Casablanca, Morocco

####### **Correspondence:** Kaoutar Benatti - benattikaoutar@hotmail.fr

*Annals of Intensive Care* 2017, **7(Suppl 1)**:P33

**Introduction** Central venous catheters constitute invasive devices that are important for patients in intensive care units. They expose to multiple complications such as: infectious, traumatic and thrombo embolic complications [1].

The aim of our study is to look for the incidence of thrombo embolic events and to target its predictive factors.

**Materials and methods** It’s a prospective study lead in the intensive care unit department during 3 months.

Patients included: Every patient admitted in our department needing a central venous catheter for therapeutic use.

Exclusion criteria: Patient admitted with a central venous catheter (CVC), a long term CVC (for dialysis), Presence of thrombo embolic event before catheterization.

All CVC were placed with the help of echoguidance.

Each day, we realized a Doppler on the catheter estimating: permeability, Doppler velocity, the presence of thrombus. If this last was detected, we evaluate its length, mobility and adherence to the vein. The entire limb was explored through Doppler echography.

Patients were randomized into 2 groups: with and without thrombus.

Data was exploited by SPSS software by univariate then multivariate analysis.

**Results** 122 catheters were analysed, thrombus incidence was 24.5%.

The comparison between the two groups is mentioned in Table 16.

**Table 16:** See text for description

	Thrombus+	Thrombus−	OR (95% CI)	p
Age (ans)	49.9	48.6	–	0.757
Sex (n, %)				
Male	23 (25.6)	67 (74.4)	1.23 (0.47–3.21)	0.678
Female	7 (21.9)	25 (78.1)		
Neoplasy (n, %)	15 (50.0)	28 (30.1)	2.32 (1.00–5.39)	0.047
Complication (n, %) (slough or sepsis)	26 (86.7)	50 (53.8)	5.59 (1.81–17.28)	0.001
Anticoagulant (n, %)	15 (50.0)	55 (59.1)	0.69 (0.30–1.58)	0.379
PAR (n, %)	28 (93.3)	62 (66.7)	7.00 (1.56–31.31)	0.004
VAD (n, %)	16 (53.3)	35 (37.6)	1.89 (0.82–4.35)	0.129
Type of catheter (n, %)				
Double	5 (16.7)	47 (50.5)	1	0.001
Triple	25 (83.3)	46 (49.5)	5.11 (1.80–14.49)	

*ATB* antibiotics, *PAR* parenteral feeding, *VAD* Vaso active drugs

The thrombus was mobile in 83% with a mean length of 4.1 mm.

**Conclusion** Thrombo embolic events on CVC are frequent, varying from 8% to 25% of cases and depend on the site.

The Doppler helps us to diagnose it quickly and to prevent heavy complications.

In our study, predictive factors were: the neoplasy (p = 0.045), parenteral feeding and the use of triple lumen catheter.

**Competing interests** None.

**Reference**

1. Parienti JJ, et al. Intravascular complications of central venous catheterization by insertion site. N Engl J Med. 2015;373(13):1220–9.

#### P34 The effects of volume expansion on cardiac output cannot be detected by the changes in heart rate

##### Zakaria Ait Hamou^1^, Jean-Louis Teboul^1^, Nadia Anguel^1^, Christian Richard^1^, Xavier Monnet^1^

###### ^1^Réanimation médicale, CHU de Bicêtre, Le Kremlin Bicêtre, France

####### **Correspondence:** Zakaria Ait Hamou - aithamou-zakaria@hotmail.fr

*Annals of Intensive Care* 2017, **7(Suppl 1)**:P34

**Introduction** The primary goal of volume expansion in acute circulatory failure is to increase cardiac output. Nevertheless, techniques that measure cardiac output are not available in every patient. Changes in heart rate could be used instead of cardiac output, since increases in cardiac output induce a decrease in the reflex sympathetic stimulation that may be reflected by significant decreases in heart rate. Our goal was to test whether the changes in heart rate could be used to differentiate positive and negative response to volume expansion.

**Materials and methods** We measured heart rate and cardiac output before and after volume expansion (500 mL saline over 10–30 min) performed in patients with acute circulatory failure. We tested the ability of changes in heart rate to detect fluid responsiveness, defined by an increase in cardiac output by more than 15% with volume expansion.

**Results** We analysed 542 volume expansions performed in 242 patients (age 63 ± 14 years old, SAPSII 63 ± 13). The origin of shock was septic in 375 (69.2%) cases, cardiogenic in 24 (4.4%) cases, hypovolemic in 114 (21%) cases, vasoplegic without sepsis in 21 (3.9%) cases and unknown in 8 (1.5%) cases. Cardiac index increased from 3.0 ± 1.1 L/min/m^2^ to 3.7 ± 1.3 L/min/m^2^ (p < 0.01) and heart rate decreased from 96 ± 35 beats/min to 95 ± 31 beats/min (p < 0.01). Cardiac index increased ≥15% in 302 (56%) cases, ≥30% in 142 (26%) cases and ≥50% in 61 (11%) cases. Changes in heart rate were not able to detect increases in cardiac index, neither ≥15%, nor ≥30%, nor ≥50% (area under the Receiver Operating Characteristic curves not different from 0.5). Nevertheless, if heart rate decreased ≥20%, which occurred in 15 cases only, an increase in cardiac index ≥30% could be predicted with a sensitivity of 1% (95% confidence interval (CI) 0–5%) and a positive predictive value of 25% (3–65%)%, but with a specificity of 98% (97–99%) and a negative predictive value of 74% (70–78%). Also, if heart rate decreased ≥20%, an increase in cardiac index ≥50% could be predicted with a sensitivity of 2% (0–9%) and a positive predictive value of 1% (1–1%), but with a specificity of 99% (97–99%) and a negative predictive value of 90% (86–92%).

**Conclusion** Except for changes of very large amplitude, decreases in heart rate cannot reliably detect a positive response of cardiac output to volume expansion. Changes in heart rate only allow the detection of large changes in cardiac output.

**Competing interests** None.

#### P35 Effect of positive end-expiratory pressure and tidal ventilation on the mean systemic filling pressure

##### Xavier Repessé^1^, Cyril Charron^1^, Alix Aubry^2^, Alexis Paternot^2^, Julien Maizel^3^, Michel Slama^3^, Antoine Vieillard-Baron^1^

###### ^1^Réanimation médico-chirurgicale, Assistance Publique - Hôpitaux de Paris, Hôpital Ambroise Paré, Boulogne-Billancourt, France; ^2^Réanimation médico-chirurgicale, Hôpital Ambroise Paré (AP-HP), Boulogne-Billancourt, France; ^3^Réanimation médicale, Centre Hospitalier Universitaire, Amiens, France

####### **Correspondence:** Xavier Repessé - xavier.repesse@aphp.fr

*Annals of Intensive Care* 2017, **7(Suppl 1)**:P35

**Introduction** Mean systemic filling pressure (MSFP) defines the pressure measured in the veno-arterial circulatory system when the cardiac output is nil. Representing the upstream pressure of the venous return gradient, it is considered as a major hemodynamic parameter. Because of the difficulties of its measurement, it is not routinely used in clinical practice but some authors proposed to use heart–lung interaction under mechanical ventilation for its estimation. Our objective was to demonstrate how MSFP is modified by positive pressure ventilation.

**Patients and methods** We conducted a bi-centric non interventional, prospective and observational study to measure the pressure encountered in the circulatory system at the time of death in critically-ill patients on an arterial catheter and a central venous catheter. We included 112 patients with previously inserted catheters and mechanical ventilation with a positive end-expiratory pressure (PEEP) at the time of death. After calibration and supine positioning, arterial and venous pressures were recorded in five conditions: at end-expiration and end-inspiration with and without PEEP and finally after disconnection from the ventilator.

**Results** Pressure measured on arterial and central venous catheters did not differ, both representing MSFP. Both arterial and central venous pressures increase with tidal ventilation and with PEEP (Fig. 14). The position of the arterial or the venous central catheters did not have any impact on these results.

**Fig. 14:**
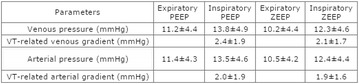
Effect of tidal ventilation and positive expiratory-end pressure one MSFP measured on central venous catheter and arterial catheter

**Conclusion** MSFP measured both on arterial and central venous catheters is altered by mechanical ventilation, with a tidal volume and PEEP effect, questioning the use of cardio-pulmonary interactions under mechanical ventilation for its extrapolation.

**Competing interests** None.

#### P36 Invasive monitoring in intensive units: diagnostic, therapeutic and prognostic benefits

##### Ahlem Trifi^1^, Sami Abdellatif^1^, Meriem Fatnassi^1^, Foued Daly^1^, Rochdi Nasri^1^, Khaoula Ben Ismail^1^, Salah Ben Lakhal^1^

###### ^1^Réanimation médicale, centre hospitalier universitaire la Rabta de Tunis, Tunis, Tunisia

####### **Correspondence:** Ahlem Trifi - ahlem.ahurabta@gmail.com

*Annals of Intensive Care* 2017, **7(Suppl 1)**:P36

**Introduction** Hemodynamic monitoring is an indubitable device in the diagnostic and therapeutic management in critically ill patients. The pulmonary artery catheter (PAC), since 1970, is the gold standard of recommended devices due to the diversity and relevance of provided parameters. Pulse contour cardiac output (PiCCO) represents an interesting alternative to the PAC. However invasiveness and delicacy in the collection and interpretation of data make that the risk/benefit ratio of these techniques is to be worrying.

Our purposes were to assess the diagnostic, therapeutic and prognostic utility of invasive monitoring by PAC and PiCCO compared to non-invasive or less common tools.

**Patients and methods** Evaluative retrospective study comparing two arms of ICU patients with invasive versus non-invasive hemodynamic monitoring. Were included, the ICU patients who had hemodynamic and/or respiratory failure and how required the use of hemodynamic exploration. Exclusion criteria were age less than 18 years and the lack of data on the medical record. Outcomes: diagnostic utility was judged on the consistency and quality of data interpretation, therapeutic usefulness on the therapeutic changes and favourable response following these therapeutic adjustments during the 1st 24 h of monitoring, and prognostic assessment was judged on the ventilation period, length of stay and mortality.

**Results** 131 cases of hemodynamic monitoring were included (PAC/PiCCO: n = 71 and common tools: n = 60). The superiority of the invasive monitoring was objectified with the consistency of interpretation of the collected parameters (91.5 vs 78.4%, p = 0.044) and therapeutic changes derived by interpretation (83 vs 66.7%, p = 0.041). No benefit found on the clinical improvement following the modifications guided by monitoring (50 vs 57%, p = 0.72) and (31 vs 47%, p = 0.074) for the hemodynamic and respiratory responses respectively. The duration of ventilation and stay-length did not differ with those of the standard group. A higher mortality was found with age >50 years (OR 1.54, 95% CI [1.05–2.27], p = 0.02) and SAPS II score >35 (OR 1.8, 95% CI [1.09–3.35], p = 0.049).

**Conclusion** the diagnostic value and therapeutic changes guided by monitoring were significantly in favour of PAC/PiCCO technical. No benefits on hemodynamic and respiratory efficiency following these changes with a higher mortality. The invasive techniques keep their interest in intricate situations and where the contribution of non-invasive tools, mainly the echocardiography, is insufficient.

**Competing interests** None.

#### P37 Prognostic value and time course evolution of left ventricular global longitudinal strain in septic shock, preliminary results of a prospective study

##### Florian Bazalgette^1^, Aurelien Daurat^1^, Claire Roger^1^, Laurent Muller^1^, Jean Yves Lefrant^2^

###### ^1^Réanimation, CHRU De Nîmes, Nîmes, France; ^2^Réanimation, Hopital Carémeau, Nîmes, France

####### **Correspondence:** Florian Bazalgette - flobazal@hotmail.fr

*Annals of Intensive Care* 2017, **7(Suppl 1)**:P37

**Introduction** Septic cardiomyopathy is commonly encountered in patients with septic shock. Most studies suggest no correlation between left ventricular ejection fraction (LVEF) and mortality in patients with septic shock. In the other hand, the initial left ventricle global longitudinal strain (LVGLS) seems to be a better prognostic factor. However its time course evolution remains to be precisely defined.

The present study aims to describe the evolution during the first days of the septic shock and to establish the prognostic value of this novel parameter in critically ill patients.

**Materials and methods** A prospective observational single center study was performed in the ICU.

After approval of the local ethics committee, all patient admitted to the ICU for septic shock without known pre-existing heart disease were eligible. In these preliminary results, 13 of the 100 planned patient were included and analysed.

Echocardiography was performed on the first day, and repeated daily during ICU stay until norepinephrin was stopped. LVEF and LVGLS were acquired in apical two-chamber, four-chamber and long-axis views. Patients were divided into two groups: survivors and non survivors.

**Results** Of the first 13 patients included mortality in the ICU was 23%. Left ventricular ejection fraction at admission (LVEF) was not significantly different among the survivors and non survivors (respectively 53% ± 12 vs 28% ± 9; p = 0.089). Initial LVGLS was lower (which indicates better function) in survivors as compared to non survivors (−13% ± 12 vs −6% ± 0.5; p = 0.044). Time course evolution of LVGLS is displayed in Fig. 15.

**Fig. 15:**
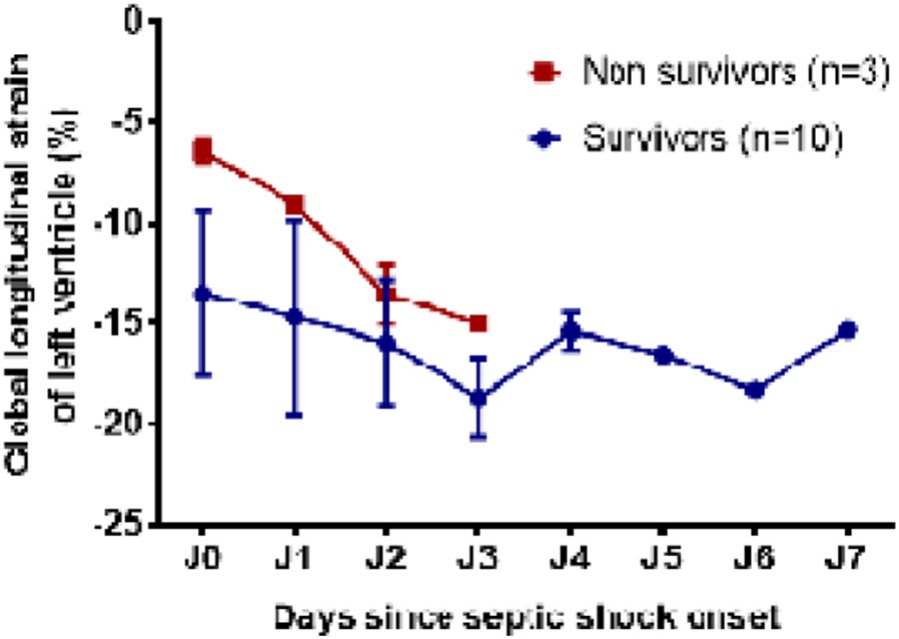
Time course evolution of LVGLS

**Conclusion** The preliminary results of the present study suggest that LVGLS has a better prognostic value than LVEF in the initial stage of septic shock.

**Competing interests** None.

#### P38 Prevalence, clinical characteristics and outcomes of left ventricular diastolic dysfunction in the intensive care unit

##### Denis Doyen^1^, Rémi Plattier^1^, Alexandre Robert^2^, Hervé Hyvernat^1^, Jean Dellamonica^3^, Gilles Bernardin^4^

###### ^1^Réanimation médicale, CHU Hôpital l’Archet 1, Route Saint-Antoine de Ginestière, Nice, France; ^2^Réanimation médicale, Hôpitaux de l’Archet 1 et 2, Nice, France; ^3^Réanimation médicale, Centre Hospitalier Universitaire Archet, Nice, France; ^4^Service de réanimation, Hôpital l’Archet 2, Nice, France

####### **Correspondence:** Denis Doyen - doyen.d@chu-nice.fr

*Annals of Intensive Care* 2017, **7(Suppl 1)**:P38

**Introduction** Left ventricular diastolic dysfunction (LVDD) represents about 50% of the causes of heart failure [1]. However little is known about LVDD in the intensive care unit (ICU) setting. The purpose of our study was to determine the prevalence, clinical characteristics and outcome of LVDD in the ICU.

**Materials and methods** We performed a prospective monocentric study in a medical ICU. Between august 2014 and December 2014, for all consecutively admitted patients, transthoracic echocardiography was realized during the first 24 h after ICU admission. LVDD was established according 2 definitions: 1 complete definition based on American Society of Echocardiography criteria [2], and 1 simplified definition.

**Results** LVDD prevalence was 61.4% (102/166) according to the complete definition and 56.0% (93/166) according to the simplified definition. LVVD patients were older (66.5 vs 55 years, p < 0.0001). Male sex predominated (67.6 vs 48.4%, p < 0.0001). LVVD was more associated with cardiogenic pulmonary oedema, diuretics use, and new onset of atrial fibrillation, whether the full definition (25.5 vs 0%, p < 0.0001; 52.9 vs 34.4%, p < 0.0001; and 27.5 vs 14.1%, p = 0.044 respectively) or the simplified definition (28 vs 0%, p < 0.0001; 53.8 vs 35.6%, p = 0.002; and 29 vs 13.7%, p = 0.018 respectively). Unlike the full definition, LVDD patients detected with the simplified definition were associated with more important intra-hospital mortality (28 vs 12.5%, p = 0.016).

**Discussion** LVDD prevalence found in our study is similar as in previous works in the cardiology setting. Higher incidences of atrial fibrillation and cardiogenic pulmonary oedema in the LVDD patients can be explained by a more important left atrial dilation, and a higher incidence of other cardiomyopathies in the LVDD cohort.

**Conclusion** LVDD is a frequent abnormality in the ICU setting, associated with an important morbi-mortality. LVDD identification should alert the physician for an higher risk of new onset of atrial fibrillation and cardiogenic pulmonary oedema.

**Competing interests** None.

**References**

1. Ponikowski P, Voors AA, Anker SD, Bueno H, Cleland JG, Coats AJ, et al. 2016 ESC Guidelines for the diagnosis and treatment of acute and chronic heart failure: The Task Force for the diagnosis and treatment of acute and chronic heart failure of the European Society of Cardiology (ESC) Developed with the special contribution of the Heart Failure Association (HFA) of the ESC. Eur Heart J. 2016;37(27):2129–200.
2. Nagueh SF, Smiseth OA, Appleton CP, Byrd BF, 3rd, Dokainish H, Edvardsen T, et al. Recommendations for the Evaluation of Left Ventricular Diastolic Function by Echocardiography: An Update from the American Society of Echocardiography and the European Association of Cardiovascular Imaging. J Am Soc Echocardiogr. 2016;29(4):277–314.

#### P39 Precision of measurements with transthoracic echocardiography in critically ill patients

##### Mathieu Jozwiak^1^, Julia Gimenez^1^, Jean-Louis Teboul^1^, Pablo Mercado^1^, François Depret^1^, Christian Richard^1^, Xavier Monnet^1^

###### ^1^Service de réanimation médicale, inserm umr s_999, université paris-sud, Hôpital de bicêtre, hôpitaux universitaires paris-sud, Assistance publique – Hôpitaux de Paris, Le Kremlin-Bicêtre, France, France

####### **Correspondence:** Mathieu Jozwiak - mathieu.jozwiak@aphp.fr

*Annals of Intensive Care* 2017, **7(Suppl 1)**:P39

**Introduction** We wanted to determine the precision of echocardiographic measurements performed in critically ill patients.

**Patients and methods** We included 100 hemodynamically stable patients (age 67 ± 16 years, SAPSII 52 ± 19, 54% mechanically ventilated, 16% with atrial fibrillation and 34% under norepinephrine). Three successive echocardiography examinations were performed by two different operators with a national echocardiographic diploma, the first and the third by one operator and the second one by the other one. Within each examination, three measurements were performed for each variable without moving the probe from the patient at the end of expiration and averaged. For every echocardiographic variable, we calculated the precision and the least significant change (LSC), i.e. the minimal change in variable that could be trusted to be significant.

**Results** When calculated from the two examinations performed by the same operator, the precision of an echocardiographic examination was 10 ± 9% for velocity time integral (VTI). It is exactly equal to the limit that is usually regarded as acceptable for measures estimating cardiac output. For LV ejection fraction (LVEF), early peak velocity of transmitral flow (E) at pulsed Doppler, early diastolic peak velocity of the lateral mitral annulus (e’) at Tissue Doppler Imaging and LV end-diastolic area (LVEDA), the precision was 7 ± 5%, 8 ± 9%, 17 ± 16% and 10 ± 8%, respectively. In this condition also, LSC of VTI was14 ± 13%. For LVEF, E, e’ and LVEDA, LSC was 10 ± 8%, 12 ± 12%, 24 ± 23% and 15 ± 11%, respectively. When calculated from the two examinations performed by the two different operators, the precision of an echocardiographic examination was 13 ± 12% for VTI. For LVEF, E, e’ and LVEDA, it was 8 ± 7%, 10 ± 8%, 19 ± 19% and 13 ± 11% for LVEF, E, e’ and LVEDA, respectively. In this condition also, the LSC was 19 ± 17% for VTI. It was 11 ± 10%, 14 ± 12%, 28 ± 27% and 18 ± 15% for LVEF, E, e’ and LVEDA, respectively.

**Conclusion** When an echocardiographic examination is performed by averaging three measurements, the precision of echocardiography is almost acceptable for VTI. It is better for LVEDA and LVEF. We are currently investigating the number of measurements that must be averaged within one examination to obtain a satisfactory precision for VTI.

**Competing interests** None.

#### P40 Mixed venous O_2_ saturation and fluid responsiveness

##### Najla Tilouch^1^, Houda Mater^1^, Ben Sik Ali Habiba^2^, Oussama Jaoued^1^, Rim Gharbi^1^, Mohamed Fekih Hassen^1^, Souheil Elatrous^1^

###### ^1^Réanimation Médicale, EPS Taher Sfar Mahdia, Mahdia, Tunisia; ^2^Réanimation médicale, EP taher sfar, Mahdia, Tunisia

####### **Correspondence:** Mohamed Fekih Hassen - mohamed.fekihhassen@rns.tn

*Annals of Intensive Care* 2017, **7(Suppl 1)**:P40

**Introduction** Hypovolemia is common in intensive care patients. It may reduce cardiac output and O_2_ delivery relative to tissue need. Low mixed Central venous O_2_ saturation (ScvO_2_) has been used to optimize cardiac output and O_2_ delivery relative to tissue needs. The use of ScvO_2_ to predict fluid responsiveness is unclear. Objective: To determine whether ScvO_2_ is a good indicator of fluid responsiveness.

**Patients and methods** We carried out a prospective study in the medical intensive care unit of the teaching hospital in Mahdia over a period of 48 months. All patients with circulatory failure were enrolled. At baseline (t  =  0 min), hemodynamic measurements were performed with a PICCO. After baseline measurements and blood sampling, 500 cc of fluids were given over 30 mn and hemodynamic measurement were performed at the end of fluid perfusion. Concomitant vasoactive and sedative drugs and ventilatory settings remained unchanged. Fluid responsiveness was defined as an increase in cardiac index >15%.

**Results** A total of 68 patients requiring volume expansion were included. The causes of acute circulatory failure were septic shock (*n* = 45), cardiogenic shock (*n* = 11), and dehydration (*n* = 12). Among the 68 included patients, 33 (49%) were responders. ScvO_2_ was significantly lower in no responders group (62 ± 11 vs 68 ± 12, p = 0.046). However ScvO_2_ variation was significantly higher in responders group (10 ± 8 vs 6 ± 7, p = 0.041). The area under the ROC curve for ScvO_2_ was 0. 37 (95% CI 0.23–0.5). The best cutoff value of ScvO_2_ was <55% (sensibility = 36%, specificity = 8 8%, positive predictive value = 75% and negative predictive value = 60%). The area under the ROC curve for ScvO_2_ variation was 0.65 (95% CI 0.52–0.78). The area under the ROC curve for delta ScvO_2_ was 0.5 (95% CI 0.43–0.7).

**Conclusion** In this study, we can’t recommend strongly the use of ScvO_2_ as a predictor of fluid responsiveness.

**Competing interests** None.

#### P42 Association between personality traits and life-saving interventions: a simulation-based study and a questionnaire survey

##### Pierre Pasquier^1^, Quentin Vuillemin^2^, Jean-Vivien Schaal^3^, Thibault Martinez^1^, Sandrine Duron^4^, Marion Trousselard^5^, Pierre-Eric Schwartzbrod^6^

###### ^1^Service de réanimation, Hôpital d’Instruction des Armées Percy, Clamart, France; ^2^Antenne médicale, Centre médical des armées, Besançon, France; ^3^Centre de traitement des brûlés, Hôpital d’Instruction des Armées Percy, Clamart, France; ^4^Unité de recherche, Centre d’épidémiologie et de santé publique des armées, Marseille, France; ^5^Département de neurosciences et contraintes opérationnelles, Institut de Recherche Biomédicale des Armées, Brétigny-sur-Orge, France; ^6^Antenne médicale, Centre médical des armées, Besançon, France

####### **Correspondence:** Pierre Pasquier - pasquier9606@me.com

*Annals of Intensive Care* 2017, **7(Suppl 1)**:P42

**Introduction** The application of tactical tourniquet is one of the most important lifesaving intervention (LSI) on the battlefield and in theaters of terrorist attacks. Despite its apparent simplicity, the failure rate of tactical tourniquet application remains high in real life experience. Some personality traits of the combat lifesavers could explain this high failure rate. The aim of this study was to analyze the association between different personality traits and the performance for applying the tactical tourniquet.

**Materials and methods** This observational, cross-sectional study concerned French soldiers of two theaters of operations in Africa (SANGARIS in Central African Republic and BARKHANE in Malia), between October 2015 and April 2016. The performance for application of the tactical tourniquet SOFFT^®^ was evaluated during simulation sessions and included the time for application and its effectiveness, demonstrated by the elimination of the popliteal pulse Doppler signal. A performance score included both an application time shorter than 60 s and the interruption of the Doppler signal. Personality traits were assessed using validated questionnaires: the Self-Esteem Scale of Rosenberg (SES), the HEXACO personality inventory for empathy (HEXACO), the Freiburg Mindfulness Inventory (FMI) and the Büssing Altruism Scale (BAS).

**Results** A total of 72 participants (mean age 27 ± 4) were included in the study. The effectiveness rate of tactical tourniquet use was 51%. Only 26% of the participants performed this LSI in accordance with the performance score criteria. Some of the personality traits were associated with significant differences in the performance of the SOFFT^®^ application. Thus, the most empathetic soldiers (high HEXACO levels) had weak performance scores, in comparison with those with a lower HEXACO level (p = 0.016). Participants with a high BAS level were also less performant for application of the tactical tourniquet. However, combattants with a high SES applied more quickly and more efficiently the tactical tourniquet (Fig. 16).

**Fig. 16:**
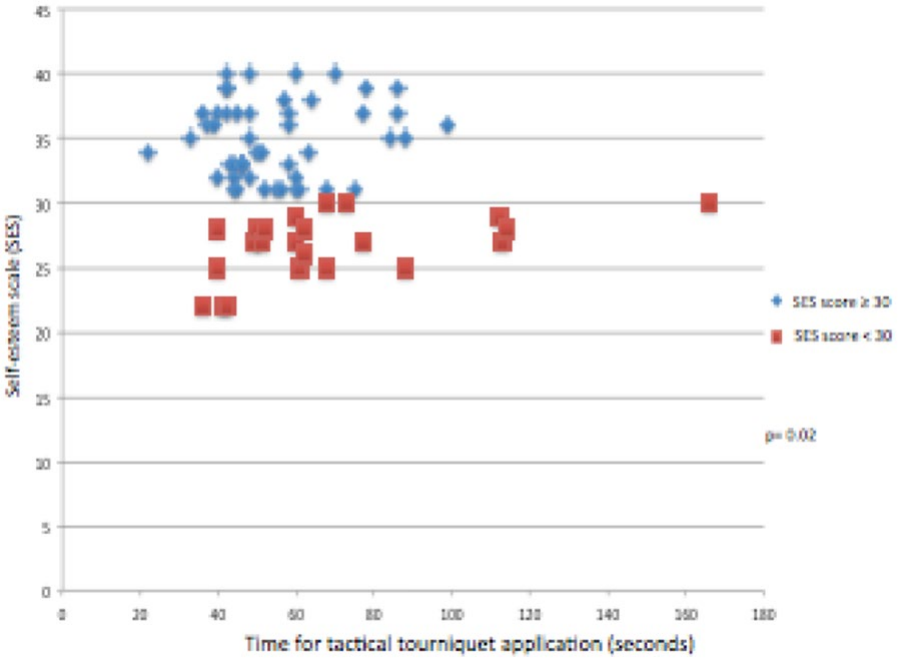
See text for description

**Conclusion** Personality traits are associated with significant differences for the performance of tactical tourniquet application, a crucial LSI in setting of both combat and terrorist attacks. Personality traits can be evaluated easily, using validated questionnaires. Finally, the “adaptive learning” could represent an adequate approach to improve the LSI training, according to the personality traits of the trainees.

**Competing interests** None.

**References**

1. Pasquier P, Dubost C, Boutonnet M, Chrisment A, Villevieille T, Batjom E, et al. Predeployment training for forward medicalisation in a combat zone: the specific policy of the French Military Health Service. Injury. 2014;45(9):1307–11.
2. Trousselard M, Steiler D, Claverie D, Canini F. Relationship between mindfulness and psychological adjustment in soldiers according to their confrontation with repeated deployments and stressors. Psychology. 2012;3(1):100–15.

#### P43 Teaching communication skills through simulation: experience with residents and fellows in paediatric intensive care and anaesthesiology

##### Thomas Baugnon^1^, Laurent Dupic^2^, Caroline Duracher Gout^1^, Laure De Saint Blanquat^2^, Sylvie Séguret^2^, Gaelle Le Ficher^1^, Gilles Orliaguet^1^, Philippe Hubert^2^, Groupe Communication et Simulation en Pédiatrie

###### ^1^Réanimation neurochirurgicale pédiatrique, Hôpital Necker - Enfants Malades, Paris, France; ^2^Réanimation et surveillance continue médico-chirurgicales, Hôpital Necker - Enfants Malades, Paris, France

####### **Correspondence:** Thomas Baugnon - thomas.baugnon@aphp.fr

*Annals of Intensive Care* 2017, **7(Suppl 1)**:P43

**Introduction** Delivering bad news regarding prognosis or care-related damages are difficult situations generating anxiety for patients but also for the medical teams who feel generally insufficiently prepared and trained. We have studied the interest of training paediatric and anaesthesiologist residents and fellows in difficult communication through simulation.

**Materials and methods** This multimodal training with multidisciplinary teachers (psychologists and doctors) included theoretical interactive sessions (behaviour and environment to inform parents in an acute situation, modalities of communication, and knowledge about the defense mechanisms…) and simulation sessions with professional actors playing child’s parents. The scenarios promoted situations after significant events involving a child in PICU or in the operative room. Each scenario was created from real case. Standardized debriefing by teachers and a psychologist was done after each scenario with actors and every participant. A pre and post-test using Lickert scale was fulfilled to evaluate the interest of this program and the confidence in this kind of meeting. The results are expressed in median [interquartile] and comparison used the U Man-Whitney test. A p value <0.05 was considered significant.

**Results** Thirty-one medical residents (17 anaesthesiologists, 14 paediatricians) and 5 fellows (1 anaesthesiologist and 4 paediatricians) have participated at this 1 day course training. 44% declared to have previously received a course on communication, and 80% had already experienced, alone, situations with the announcement of bad news.

*PreTest*

- How you consider important the communication with families?5 [5–5]
- Do you feel trained in communication with the patient or relatives? 2 [2–3]
- Do you feel anxious in situations requiring breaking bad news? 3 [2–3]

*Post Test*

- How do you evaluate this training course? 4 [4–4]
- Does this training course meet your expectations? 4 [4–4]
- Do you feel anxious in situations requiring breaking bad news? 3 [3–3]
- 97% of participants declared they recommend the training to their colleagues.

See Fig. 17

**Fig. 17:**
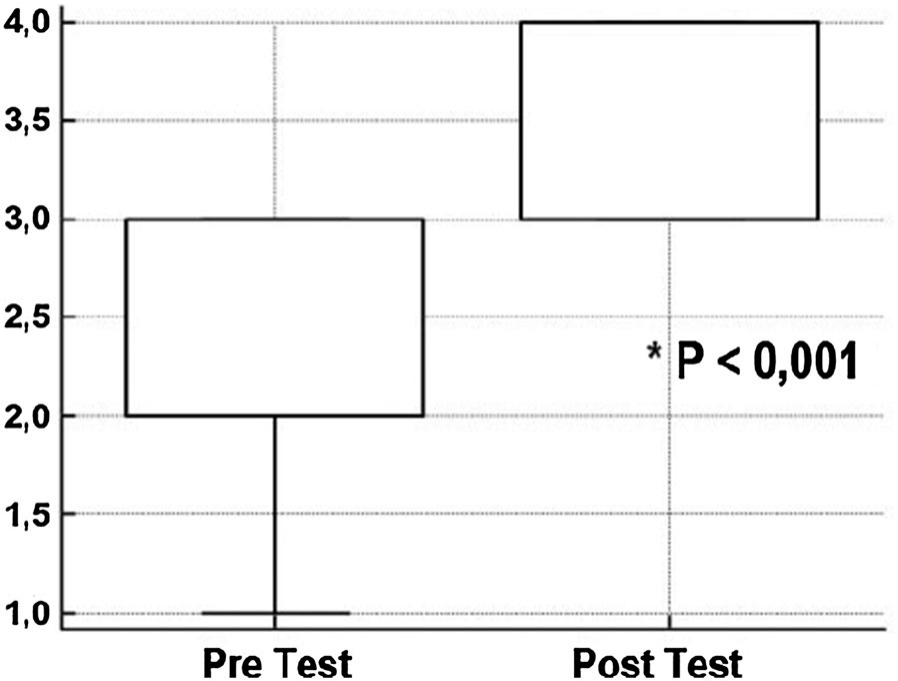
Confidence before and after training

**Discussion** The participants reported a significant lack of initial training and a lack in confidence in communication skills with relatives’ despites their strong interest for this topic. The majority has still encountered such a situation. The post test evaluation showed a significant improvement in confidence even though the level of anxiety remained high after the session. The multimodal aspect of the program combining theoretical courses and realistic simulation with professional actors in a “secured” environment was emphasized by the learners.

**Conclusion** This study confirms the feasibility, the realism and the learners’ satisfaction. It’s of major interest to pursue development of such interprofessional training with others caregivers (nurses…) and other medical and surgical specialists.

**Competing interests** None.

**Reference**

1. Baile WF. SPIKES-A six-step protocol for delivering bad news. Oncologist. 2000;5(4):302–11.

#### P44 Usefulness of a sterile in-plane needle-guidance for internal jugular and axillary vein cannulation: a randomized controlled study on an inanimate manikin

##### Gabriel Preda^1^, Naïke Bigé^1^, Vincent Dubée^1^, Jeremie Joffre^1^, Jean-Luc Baudel^1^, Guillaume Leblanc^1^, Hafid Ait-Oufella^1^, Eric Maury^1^

###### ^1^Réanimation médicale, Hôpital Saint-Antoine, Paris, France

####### **Correspondence:** Naïke Bigé - naikebige@gmail.com

*Annals of Intensive Care* 2017, **7(Suppl 1)**:P44

**Introduction** International guidelines strongly recommend ultrasound (US) guidance for central venous catheter (CVC) insertion. Compared to anatomical landmark technique, US guidance reduces the number of attempts, and time to successful cannulation of internal jugular (IJ) vein [1]. However, adequate studies to prove this benefit for subclavian/axillary vein catheterization are lacking [2]. This could be explained by anatomical issues but also by technical difficulties. In-plane (IP) technique allows seeing the whole needle’s course but needs to place the needle in the exact middle of the probe in its long axis. If not achieved, no or only part of the needle is visualized. In the out-of-plane (OOP) method, only the needle’s tip is visualized, and the course of the needle is not controlled. Needle guidance systems are aimed at improving needle visualization but data supporting their advantage over conventional US guidance are scarce in the setting of axillary vein cannulation. We conducted the present study to compare a sterile in-plane needle-guide (Infinity Pro; CIVCO Medical Solutions) to standard freehand US guidance during IJ and axillary veins cannulation.

**Materials and methods** For both sites, each operator performed venipuncture using three methods: (1) standard OOP, (2) standard IP, and (3) IP with tested guide. Procedure was stopped at 180 s when the operator failed to cannulate vein. Punctures were performed on an inanimate manikin (Blue Phantom II, CAE Healthcare St. Louis, MO) using the M-Turbo^®^ device (Sonosite, Bothewell, MA) with a 7.5 MHz linear probe equipped or not with the tested guide. The order of punctures—site (IJ or axillary) and method—was randomized using a 2-by-3 design in a 1:1:1:1:1:1 ratio. The random allocation sequence was generated using a random number table.

The number of attempts (needle passes) before success and time between first skin puncture and successful venous puncture were recorded. Qualitative and quantitative values are expressed as number (percentage), and median (range), and were compared using the Wilcoxon matched pairs test and the Fisher exact test, respectively.

**Results** Twenty physicians [median age 22 (22; 27) years, 7 senior physicians, graduated in intensive care medicine, 6 residents, and 3 medicine students] participated to this randomized controlled study. Twelve (60%) declared prior experience in US-guidance for CVC insertion, and none had previously used the tested device.

At the axillary site, the tested device significantly reduced the number of attempts compared to both standard techniques (median 1 [1; 1] attempts vs 1 [1; 2.8] for OOP standard, p = 0.021, and vs 1 [1; 2], p = 0.035 for IP standard). Time between first skin puncture and success was also significantly reduced with tested guide (median 16.5 [9.8; 39.3] s) compared to OOP standard (median 32.5 [9; 180] s, p = 0.029) and tended to be shorter than IP standard method (median 29 [9.5; 96.3] s, p = 0.079). Success rate at first attempt was 85.5% using needle guidance, whereas it was 45% with OOP standard (p = 0.0057) and 55% with IP standard (p = 0.031) methods. No significant difference was observed between needle-guidance and standard methods for IJ cannulation. Compared to IP standard, seventeen (85%) operators preferred in-plane needle guidance device for axillary vein puncture, whereas only 5 (25%) preferred tested device for IJ puncture.

**Discussion** These results have yet to be confirmed on a larger sample size and for bedside procedures on patients.

**Conclusion** Consistently with previous findings, these results suggest the interest of needle-guidance devices over freehand method for US-guided axillary but not for IJ vein puncture.

**Competing interests** None.

**References**

1. Brass P, Hellmich M, Kolodziej L, Schick G, Smith AF. Ultrasound guidance versus anatomical landmarks for internal jugular vein catheterization. Cochrane Database Syst Rev. 2015;1–190.
2. Brass P, Hellmich M, Kolodziej L, Schick G, Smith AF. Ultrasound guidance versus anatomical landmarks for subclavian or femoral vein catheterization. Cochrane Database Syst Rev. 2015;1–81.

#### P45 Medical students’ first foray into clinical research through a professional practice assessment: Could it be a win–win experience?

##### Raphael Briand^1^, Lucas Brousse^1^, Valentine Brunet^1^, Léonard Chatelain^1^, Dominique Prat^1^, Frédéric Jacobs^1^, Nadège Demars^1^, Olfa Hamzaoui^1^, Guy Moneger^2^, Anne Sylvie Dumenil^3^, Pierre Trouiller^1^, Benjamin Sztrymf^1^

###### ^1^Réanimation polyvalente, Hôpital Antoine Béclère, Clamart, France; ^2^Réanimation polyvalente, Hospital Antoine Béclère, Clamart, France; ^3^Réanimation chirurgicale, Hôpital Antoine Béclère, Clamart, France

####### **Correspondence:** Benjamin Sztrymf - benjamin.sztrymf@aphp.fr

*Annals of Intensive Care* 2017, **7(Suppl 1)**:P45

**Introduction** Though efforts have been made in the previous decades to teach medical students the basics of clinical research, their own involvement in such studies infrequently occurs before they become residents or assistants. The medical courses include lessons on experimental and clinical research methods, which may be difficult to integrate out of a concrete context. On the other hand, much has to be learned for medical students in ICU within a short training period. Involving medical students in research could increase their workload, and may affect their evaluation of the training period. We therefore aimed to have medical students making their first foray in clinical research through a practice evaluation. We asked them to evaluate the change in workload and their global satisfaction afterward.

**Patients and methods** Four medical students (4th year of medicine) agreed to participate to a clinical trial through an evaluation of medical practices. None of them had had a previous experience in this field. Gastric stress ulcer prophylaxis (SUP) was chosen by the students among other subjects. Two references were provided, in French, to give them clues on how to conduct the research. The first one was a free web access medical thesis exploring the medical practice on ulcer prophylaxis in three ICU in Toulouse. The second was a chapter of a reference textbook. Three weeks thereafter, it was decided in a meeting to have a 2 steps approach. First, they realized an evaluation of the local practices through a daily monitoring of patients admitted during a 2 weeks period. All patients admitted for an estimated period of more than 48 h were eligible. Exclusion criteria were baseline ulcer treatments and active gastrointestinal bleeding. Second, a brief survey of seniors and residents’ knowledge on the subject of matter was performed. In both steps of investigation, the recorded criteria were those described in the French health authority’s recommendations. At the end the study period students participated to statistical analysis and interpretation of results. The study was presented to the department’s staff by the students. A few weeks later, they were asked to evaluate this experience.

**Results** During the study period, 27 patients were screened and 14 patients (Mean age 57.4 ± 23.5 years, 4 female, 10 men, mean SAPS II 48 ± 25) were included and observed during a total 105 ICU days (7.5 ± 6.4 days/patient). 6 (42.9%) patients received stress ulcer prophylaxis at least once, and during 71.6 ± 32.6% of their ICU stay. According to French recommendations, SUP was not justified at least once in 5 out of the 6 patients. In 29% of SUP days, no criteria supported this prescription. 8 patients did not receive SUP. These patients were observed during 66 days. According to guidelines, SUP should have been offered in 2 patients during 15 ICU days. In this subgroup of patients no criteria for SUP was found in only 13 (19.6%) days. Among the studied criteria, a strong link between SUP and enteral nutrition was found (p < 0.0001, Fisher exact test). 8 senior intensivists and 6 residents answered the survey. The estimated prevalence of this condition was evenly distributed between less than 1% and more 10% among responders. 30% of them ignored whether guidelines were available, and 80% of all responders ignored the strength of evidence of the guidelines. All the known risk factors for stress ulcer were cited, but the two major criteria (mechanical ventilation and coagulation abnormalities) were recognized in less than 30% of responders. 86% of responders declared that only proton pump inhibitor were appropriate for SUP.

All students declared that adding this study to the global burden of the training period was manageable. They pointed out that building a study was a difficult task and declared that it was an added-value to the training course, and a good experience in their medical course. They would agree to do it again.

**Discussion** This study is limited by its single center design, and the little number of participating students and included patients.

**Conclusion** Helping medical students make their first steps in clinical research seems to be feasible in ICU. The students greatly appreciated this experience and judged it improved their training period. It could also represent a mean for patient management improvement.

**Competing interests** None.

#### P46 Does observational role allow skills acquisition in cardiopulmonary resuscitation simulation training?

##### Emilie Duburcq-Gury^1^, Léa Satre-Buisson^1^, Thibault Duburcq^1^, Julien Poissy^1^, Laurent Robriquet^1^, Merce Jourdain^1^

###### ^1^Pôle de réanimation, hôpital salengro, C.H.R.U. - Lille, Avenue Oscar Lambret, Lille, France, Lille, France

####### **Correspondence:** Emilie Duburcq-Gury - emilie.gury@outlook.fr

*Annals of Intensive Care* 2017, **7(Suppl 1)**:P46

**Introduction** High-fidelity simulation is a recognized tool for acquisition and performance improvement in advanced cardiac life support. Our study aims to assess the contribution of attending a high fidelity simulation session on acquisition of technical and non-technical performances (crisis resource management CRM).

**Materials and methods** This observational prospective study included second-year post graduate medical residents from Lille2 University, divided into an observational group (A) and an intervention group (B). At the first session, a resident from group A observed a cardiac arrest scenario managed by a resident from group B (B1). A debriefing was performed after each session. The observers and actors expressed feelings, doubts and asked questions to the trainer. Three months later, all residents (A2 and B2) played the role of leader in another cardiac arrest scenario. Their technical performance was rated over 50 points by one trainer and CRM was retrospectively evaluated via a video system by three independent reviewers according to the Ottawa Global Rating Scale (GRS) over 35 points.

**Results** Thirteen residents from group A and 14 from group B were analyzed. The performances of the group B active residents significantly improved from the first (B1) to the second session (B2), with regard to the technical score (31.6 ± 5.8 vs. 41.1 ± 4.9; p = 0.0004) as well as the Ottawa GRS (18.1 ± 3.2 vs. 22.7 ± 4.6; p = 0.0007). Spectators (A2) had better technical performances than B1 active players (41 ± 3.6 vs. 31.6 ± 5.8; p < 0.0001) and were not different from B2 active players (41.1 ± 4.9 vs. 41 ± 3.6; p = 0.93). There was no significant difference for the Ottawa GRS between A2 spectators and B1 active players (20.4 ± 3.4 vs. 18.1 ± 3.2; p = 0.11), nor between A2 spectators and B2 active players (22.7 ± 4.6 vs. 20.4 ± 3.4; p = 0.17).

**Conclusion** Our findings suggest that residents who observed a simulation session developed similar technical performances than residents playing for the second time an ACR scenario. This result was not confirmed for non technical performances and further studies are needed.

**Competing interests** None.

**Reference**

1. Kim J, Neilipovitz D, Cardinal P, Chiu M, Clinch J. A pilot study using high-fidelity simulation to formally evaluate performance in the resuscitation of critically ill patients: the University of Ottawa Critical Care Medicine, High-Fidelity Simulation, and Crisis Resource Management I Study. Crit Care Med. 2006;34(8):2167–74

#### P47 High fidelity simulation and ECMO in intensive care unit: a multi professional training program to improve skills and self efficacy

##### Thierry Sécheresse^1^, Mattéo Miquet^1^, Alexis Simond^1^, Pascal Usseglio^1^

###### ^1^73, Centre hospitalier Métropole Savoie, Chambéry, France

####### **Correspondence:** Mattéo Miquet - matteo.miquet@gmail.com

*Annals of Intensive Care* 2017, **7(Suppl 1)**:P47

**Introduction** Technical progress and development of indications may lead non-specialized ICU implanting ECMO, with an investment in equipment and appropriation of new proccess. Acquisition and retention of new skills to manage these specific situations isessential but difficult because these events are infrequent at an individual level. Full scale high-fidelity simulation conducted in multi professional team is a way to improve skills and confidence of the teams for these rare situations but with immediate vital risk.

**Materials and methods** A simulation program on emergency management in relation with ECMO has been jointly set up by members of the intensive care unit for disciplinary expertise in connection with the CEnSIM team for pedagogical expertise. Three priorities have been identified:

- In situ high fidelity simulation
- Multi-professional team simulation (1 intensivist physician, 2 nurses with at least 1 ECMO referrer, 1 caregiver) by 4 h session.
- Program proposed to the whole service to ensure support for uniformity in healthcare

These sessions were conducted in an intensive care room adapted to meet the requirements of high-fidelity simulation. Each session consisted of two simulations each followed by a debriefing. Two objectives were targeted: technical achievement and teamwork.

Beyond the reactions, the main assessment was performed by measuring the evolution of the participant self-efficacy defined by Bandura as “an individual’s belief in its ability to organize and execute the course of action required to produce desired results.” Measurements were performed before and after training using analogic visual scale of self-efficacy tailored specifically to each profession.

**Results** Two test sessions and ten training sessions were conducted over a period of 6 days, allowing the participation of all the physicians and ECMO referral nurses (total participants n = 44: 10 physicians, 4 residents, 20 nurses, 10 caregivers).

This training was appreciated by all participants, both in terms of emotional perception (overall satisfaction: M = 4.9/5; SD = 0.3) and in terms of practical reinvestment (perceived usefulness: M = 4.9/5; SD = 0.3).

The results show a beneficial effect of this program on self-efficacy for the three professional categories: Delta (pre-form–post-form) caregivers score = 2.63; p < 0.005; Delta (pre-form–post-form) nurses score = 2.6 nurses; p < 0.001; Delta (pre-form–post-form) physicians score = 3.46; p < 0.001.

**Conclusion** Inter professional full scale simulation allows the acquisition of experiential knowledge and skills essential to the management of these complex situations. Beyond teamwork and technical skills, significantly increased self-efficacy is an important factor in achieving optimal behavior in emergencies. In situ simulation performed in interprofessional teams is probably an interesting method to optimize management of ECMO patient. Implementation and perpetuation of such projects need a strong institutional commitment as part of a policy.

**Competing interests** None.

#### P48 Discrepancies in ventilatory settings: frequency, typology and severity in a Tunisian medical ICU

##### Hedia Hammed^1^, Yamina Hamdaoui^1^, Jihene Ayachi^1^, Khaoula Meddeb^1^, Nesrine Fraj^1^, Nesrine Sma^1^, Ahmed Khedher^1^, Hend Ben Lakhal^1^, Messaouda Khelfa^1^, Raja Boukadida^1^, Hajer Hafsa^1^, Imed Chouchene^1^, Mohamed Boussarsar^2^

###### ^1^Réanimation médicale, CHU Farhat Hached, Sousse, Tunisia; ^2^Réanimation médicale, CHU Farhat Hached, Research Laboratory N° LR14ES05, Faculty of Medicine, Sousse, Tunisia

####### **Correspondence:** Mohamed Boussarsar - hamadi.boussarsar@gmail.com

*Annals of Intensive Care* 2017, **7(Suppl 1)**:P48

**Introduction** Mechanical ventilation is a life supporting treatment commonly indicated in patient’s ICU [1]. However, discrepancy in ventilatory settings can lead to patient–ventilator asynchrony. This adds a burden on the respiratory system and may increase the morbidity and mortality in the critically ill.

The discrepancies in ventilatory settings may be largely underestimated because of a frequent lack of monitoring [2].

The aim of the study was to evaluate frequency, typology and severity of discrepancies in ventilatory settings in a Tunisian medical ICU, and to identify factors associated with patient-ventilator asynchrony.

**Patients and methods** An audit observational study was conducted in a 7-beds medical ICU during 1 month period (August 2016). All consecutive ICU patients requiring invasive or non invasive mechanical ventilation were included. The data collected were: patient’s characteristics, initial diagnosis, SAPSII, PaO_2_/FiO_2_ ratio, type of mechanical ventilation, ventilatory mode, prescribed ventilatory parameters and interfaces. Were analyzed, peak inspiratory pressure, plateau pressure, auto-PEEP, volume and inspiratory and expiratory flow waveforms. Patient-ventilator asynchrony was defined as: ineffective inspiratory efforts, auto-triggering, delayed cycling, double triggering and inspiratory waveform distorsion.

**Results** During the study period, were performed a total of 160 ventilatory settings observations. Mean age was 59.9 ± 16.8 years, mean SAPSII score was 36.3 ± 11.1. 157 (98.1) patients were on invasive mechanical ventilation and 3(1.9) were on non-invasive mechanical ventilation. PaO_2_/FiO_2_, 286 ± 115; mean peak inspiratory pressure, auto-PEEP and plateau pressure were respectively: 35.5 ± 11.8; 8.4 ± 4.5 and 22.0 ± 4.7 cmH_2_O.

Discrepancies in ventilatory settings were found in 55(34.4) patients. 23(42) patients had frequent patient–ventilator interactions. Ineffective efforts and double triggering were the two most common asynchronies (43.4 and 13.1% respectively). Patient–ventilator interactions were assessed as severe in 9(16.3). Patient-ventilator asynchronies were associated neither to the severity (SPAS II, PaO_2_/FiO_2_) nor to the respiratory system mechanic (peak inspiratory pressure, autoPEEP, plateau pressure and driving pressure). A simple intervention on the ventilatory settings corrected the asynchronies in all the patients.

**Conclusion** The discrepancies in ventilatory settings reveals frequent. Patient-ventilator asynchronies are the most observed discrepancies. This could be the consequence of a frequent lack of monitoring.

**Competing interests** None.

**References**

1. Murias G, Lucangelo U, Blanch L. Patient–ventilator asynchrony. Curr Opin Crit Care. 2016;22(1):53–9.
2. Dres M, Rittayamai N, Brochard L. Monitoring patient–ventilator asynchrony. Curr Opin Crit Care. 2016;22(3):246–53.

#### P49 The use of the serious game Stayingalive^®^ at school improves basic life support performed by secondary pupils: a randomized controlled study

##### Victoire Desailly^1^, David Hajage^2^, Pierre Pasquier^3^, Patrick Brun^4^, Pauline Iglesias^1^, Jérémie Huet^1^, Clémence Masseran^1^, Antoine Claudon^1^, Clément Ebeyer^1^, Thomas Truong^1^, Jonathan Messika^1^, Damien Roux^5^, Jean-Damien Ricard^6^, Didier Dreyfuss^7^, Antoine Tesnière^8^, Alexandre Mignon^9^, Stéphane Gaudry^1^

###### ^1^Service de réanimation médico-chirurgicale, CHU Louis Mourier, Colombes, Colombes, France; ^2^Département d’épidémiologie et de recherche clinique, Hôpital Louis-Mourier - APHP, Colombes, France; ^3^Service de réanimation, Hôpital d’Instruction des Armées Percy, Clamart, France; ^4^Giga la vis, Institut des Hauts de Seine, Nanterre, France; ^5^Réanimation médico-chirurgicale, Hôpital Louis-Mourier - APHP, Colombes, France; ^6^Service de Réanimation Médico-Chirurgicale, CHU Louis Mourier, Colombes, France; ^7^Inserm, iame, umr 1137, Université Paris Diderot, Sorbonne Paris Cité, Paris, France; ^8^Service de pneumologie et réanimation, Hôpital Cochin, Paris, France; ^9^Laboratoire ilumens, Universisté René Descartes - Paris V, Paris, France

####### **Correspondence:** Stéphane Gaudry - stephanegaudry@gmail.com

*Annals of Intensive Care* 2017, **7(Suppl 1)**:P49

**Introduction** First aid and cardiopulmonary resuscitation training have received widespread promotion throughout the world. France has promoted a decree establishing the mandatory teaching of basic life support to all French secondary pupils (*Décret n° 2006*-*41 du 11 janvier 2006*). Large-scale feasibility of such a training, retraining and assessment remains a big challenge. We conducted a study to evaluate the training efficacy of a serious game designed for cardiopulmonary resuscitation and applied to secondary school students.

**Materials and methods** Students of 6th and 7th grades from Paris region were randomized (1:1) to one of the 2 groups using a computerized system. Randomization was stratified by classroom. In the Control group, children received a 30-min teaching on nutritional wellbeing. In the Interventional group, children played a serious game reproducing real-life cardiac arrest situation (3D real-time simulation software, *Stayingalive* ^®^, iLUMENS-Dassault Systemes) without any adult intervention. Two months later, each student was observed and evaluated by 2 assessors blinded to the study group during a cardiopulmonary resuscitation session using mini manikins *MiniAnnePlus* ^*®*^ (Laerdal Medical). The assessment scale included 15 items divided into 4 categories (cardiac arrest recognition, call for help, chest compression and use of defibrillator). Primary endpoint was the total score obtained by each student. Secondary endpoints included performance for each item.

**Results** A total of 97 children were included in the analysis (Interventional group n = 50, Control group n = 47). Median total score was significantly higher in the Interventional group (7 [6–9] vs 6 [5–8], p = 0.02, Fig. 18). In this group, children performed better on the following items: chest compression rate [10/50 (21%) vs 2/47 (4%), p = 0.01], non-stop chest compression [8/50 (17%) vs 2/47 (4%), p = 0.04], search for a defibrillator [18/50 (38%) vs 3/47 (6%), p = 0.0001], placement of electrodes [44/50 (94%) vs 36/47 (72%), p = 0.005], and defibrillator activation [35/50 (74%) vs 27/47 (54%), p = 0.04].

**Fig. 18:**
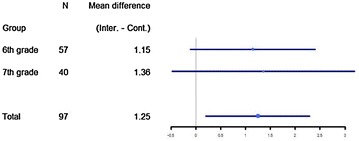
Mean difference between groups and according to class grade

**Conclusion** A 30-min experience with a cardiopulmonary resuscitation serious game without any adult intervention improves basic life support performed by secondary school students. It may be further improved by adult supervision, repeated use, and gamification using leaderboards. These results are promising to implement on a larger scale such serious games to improve knowledge and skills of children.

**Competing interests** Mini manikins were provided by Laerdal Medical.

#### P50 Management of cardiovascular manifestations in patients with Guillain–Barre syndrome associated with Zika virus infection

##### Dabor Resiere^1^, Ruddy Valentino^1^, Julien Fabre^2^, Benoit Roze^3^, Jean-Louis Ferge^1^, Cyrille Charbatier^1^, Sabia Marie^1^, Michel Scholsser^1^, Signate Aitsatou^4^, Mathieu Raad^1^, Andre Cabie^5^, Hossein Mehdaoui^1^

###### ^1^Intensive care unit, University Hospital of Martinique, Fort-de-France, France; ^2^Cardiology department, University Hospital of Martinique, Fort-de-France, France; ^3^Infectious & tropical diseases department, University Hospital of Martinique, Fort-de-France, France; ^4^Department of neurology, University Hospital of Martinique, Fort-de-France, France; ^5^Department of Infectious and Tropical Diseases, University Hospital of Martinique, Fort-de-France, France

####### **Correspondence:** Dabor Resiere - dabor.resiere@chu-fortdefrance.fr

*Annals of Intensive Care* 2017, **7(Suppl 1)**:P50

**Introduction** Although the Zika virus causes predominantly mild symptoms, this mosquito-borne disease has become the newest public health challenge. Meningoencephalitis, myelitis, Guillain-Barré syndrome (GBS) associated with deglutition disorders, arrhythmia, cardiac arrest, myocardial infarction, dysautonomia and microcephaly in new borns are among the most serious recorded complications.

The first case of Zika in Martinique, French West Indies, was diagnosed in December 2015. To date, this country of 400 000 inhabitants has recorded over 36,260 suspected cases of Zika virus infection, including 29 cases of GBS with Zika present in urine in 14 of them. Among 519 pregnant women who tested positive, only two cases of microcephaly has been identified. One patient a 76-year- old man died. Our objective was to describe severe forms of Zika-associated GBS complicated by cardiovascular disorders in patients admitted to the Intensive Care Unit (ICU) during the onset of the endemic period.

**Patients and methods** Prospective study of all Zika-associated GBS in patients admitted in our ICU from 1/12/2015 to 5/25/2016 and presenting at least one organ failure using the SOFA score. During this period, all our ICU patients were systematically tested for dengue fever, Chickungunya and Zika viruses. Cases included in this study were defined by the presence of GBS clinical signs (e.g., distal paresthesias) with biological confirmation using RT-PCR blood and urine and also CSF, and/or positive serology for IgM or IgG.

**Results** During the study period, 22 Zika-infected patients with GBS were hospitalized in the ICU. The median age was 59 years [19–84] and male/female gender ratio 1.5. The patients presented GBS symptoms (N = 22), acute renal failure (N = 8), cardiac complications (including hemodynamic disorders and arrhythmia; N = 6), bradycardia (N = 6), and ARDS (N = 7), refractory hypoxemia required ECMO VV in one patient. Respiratory failure mainly resulting from swallowing disorders required mechanical ventilation in 20 patients. Patients developed one organ failure (N = 4) and multiple organ failure (N = 18). Four patients presented cardiac arrest. Sofa score at 48 h following admission was 10 [2–18]. All the patients received intravenous polyvalent immunoglobins (0.4 g/kg/day for 5 days). The median duration of ICU stay was 15 days. One patient died.

**Conclusion** Zika infection may lead to ICU admission due to the development of GBS. Physicans particularly cardiologists should be aware of additional cardiovascular complications secondary to GBS-related dysautonomic complications. An emergent need for collaborative and multidisciplinary approach is required to mitigate the imminent Zika outbreak in the Caribbean, by providing diagnostic laboratory facilities, ICU beds availability and assistance for appropriate management of the environment. A prospective study is currently conducted to evaluate the cardiovascular complications associated with fatal cases of Zika infection virus.

**Competing interests** None.

**Reference**

1. Burakgazi AZ, Almahameed S. Cardiac involvement in peripheral neuropathies. J Clin Neuromuscul Dis. 2016;17:120–8.

#### P51 Mechanisms of sepsis-induced inhibition of malignant tumor growth in cancer mice

##### Clement Cousin^1^, Hamid Merdji^2^, Christophe Rousseau^3^, Jean-François Llitjos^4^, Fanny Alby-Laurent^5^, Julie Toubiana^5^, Nadia Belaidouni^5^, Jean-Paul Mira^4^, Jean-Daniel Chiche^4^, Frédéric Pène^4^

###### ^1^Medical Intensive Care, Hôpital Cochin, Paris, France; ^2^Departement d’anesthésie-réanimation, Hôpital Maison Blanche, Reims Cedex, France; ^3^Unité 1016, 22 rue méchain, Institut National de la Santé et de la Recherche Médicale, Paris, France; ^4^Réanimation Médicale, Hôpital Cochin, Paris, France; ^5^U1016, 22 rue méchain, Institut National de la Santé et de la Recherche Médicale, Paris, France

####### **Correspondence:** Clement Cousin - clecou@hotmail.fr

*Annals of Intensive Care* 2017, **7(Suppl 1)**:P51

**Introduction** The outcome of septic shock in cancer patients has dramatically improved over the last two decades, but the impact the acute inflammatory insult on the further prognosis of cancer is unclear. Indeed, sepsis-induced immune dysfunctions may directly impact on the prognosis of the underlying malignancy in survivors. We are currently developing a research project in order to investigate the reciprocal relationships between bacterial sepsis and malignant tumor growth. We first reported that sepsis-induced immune suppression promoted malignant tumor growth when tumor cells were inoculated in post-septic mice. Conversely, we observed that sepsis may inhibit the growth of previously established local and metastatic tumors. The present study aimed at investigating the cellular and molecular mechanisms of sepsis-induced tumor inhibition.

**Materials and methods** We used 8–12 w.o. C57BL/6 J mice. Mice were first subjected to malignant tumor inoculation by subcutaneous injection of the MCA205 fibrosarcoma cell line. Seven days after tumor inoculation, mice were subjected to polymicrobial sepsis induced by cecal ligation and puncture (CLP), without any subsequent antibiotic treatment. Controls were cancer mice subjected to sham surgery. The features of anti-tumoral immune response were compared between CLP- and sham-operated cancer mice at day 1 (early assessment) and day 7 (late assessment) following surgery. The intra-tumoral infiltration of immune cells within tumor tissues and draining lymph nodes was assessed by flow cytometry, and the intratumoral cytokine production was assessed by ELISA. We also investigated the role of bacteria and related pathogen-associated molecular patterns on growth of MCA205 cells. The expression of *Tlr2* and *Tlr4* genes was assessed by southern blot after DNA amplification.

**Results** We first confirmed that polymicrobial was able to inhibit malignant tumor growth when applied in mice previously inoculated with fibrosarcoma MCA205 cell line. Not surprisingly, the bacterial load in blood, kidneys and tumors were higher at day 1 in CLP-operated mice than in sham-operated counterparts. Of note, the bacterial contamination of tumors was not sustained at 7 days. We checked that MCA205 cells expressed functional Tlr2 and Tlr4 receptors. MCA205 cells cultured in the presence of Tlr4 and Tlr2 agonists, respectively lipopolysaccharide (LPS) and heat-killed *Staphylococcus aureus*, did not display accelerated cell growth in vitro, suggesting that the anti-tumoral effect of sepsis is mediated by the septic host’s response. The distribution of myeloid and lymphoid immune cells within tumor tissue was quite similar in sham- and CLP-operated mice, with the exception of increased proportions of inflammatory monocytes and Tgd lymphocytes in septic mice. The intra-tumoral cytokine pattern of CLP-operated mice was skewed towards an increased production of both the pro-inflammatory Tnf-a and the anti-inflammatory IL-10 cytokines at day 1.

**Conclusion** Polymicrobial sepsis applied to cancer mice inhibits the local growth of a malignant fibrosarcoma tumor. This anti-tumoral effect is not directly driven by pathogens, but rather involves an enhanced intratumoral inflammatory response. In the light of mild quantitative cell alterations, our results suggest that sepsis induces a functional modulation of immune cells resulting in potent anti-tumoral activity.

**Competing interests** None.

#### P52 Sepsis-like circulatory shock related to haematological malignancies

##### Marlène Cherruault^1^, Jérome Tamburini^2^, Julien Charpentier^1^, Didier Bouscary^2^, Jean-Paul Mira^1^, Frédéric Pène^1^

###### ^1^Réanimation médicale, Hôpital Cochin, Paris, France; ^2^Haematology, Hôpital Cochin, Paris, France

####### **Correspondence:** Marlène Cherruault - marlene.cherruault@gmail.com

*Annals of Intensive Care* 2017, **7(Suppl 1)**:P52

**Introduction** Hematological malignancies may be directly responsible for life-threatening organ failures through tumor lysis syndrome, tissue infiltration, coagulation disorders and obstruction of anatomical structures. Respiratory and renal dysfunctions are commonly encountered in this setting. In addition, some patients may develop a systemic inflammatory response syndrome, responsible for acute circulatory dysfunction, so-called sepsis-like syndrome. Besides advanced life support, the treatment of those cancer-related organ failures relies on timely administration of chemotherapy. In this study, we addressed the features and outcomes of patients with sepsis-like circulatory dysfunction related to hematological malignancies, with particular emphasis on the impact of chemotherapy on organ failures.

**Patients and methods** This was a 9-year (2007–2016) single-center retrospective observational study performed in a 24-bed medical ICU. Inclusion criteria were age ≥18 years AND presence of a hematological malignancy, either already known at the time of ICU admission or diagnosed during the ICU stay AND development of acute circulatory dysfunction requiring vasopressors without any evidence of underlying infection. Patients with hematological malignancies who received chemotherapy in the ICU were retrieved through the information systems from hospital pharmacy units involved in the delivery of cytostatic drugs, and all medical files were individually checked for inclusion criteria. Data were collected from individual files, and included the overall severity through the APACHE2 and SOFA scores computed at the time of ICU admission. The SOFA score was thereafter computed daily. Endpoints were the in-ICU and in-hospital vital status.

**Results** Over the study period, 24 patients (12 men, 12 females) fulfilled the inclusion criteria. Their mean age was 62 ± 16 years. Most of them were in good functional condition since 22 had a performance status of 0. Two patients were previously immunocompromised (severe combined immune deficiency and kidney transplant). The underlying haematological malignancies were distributed as follows: non-Hodgkin lymphoma (n = 18) including 6 patients with diffuse large B-cell lymphoma, acute myeloid leukemia (n = 4), Hodgkin’s lymphoma (n = 1) and chronic lymphocytic leukemia (n = 1). Seven patients had malignancies newly diagnosed in the ICU. Otherwise, the median time from diagnosis to ICU admission was 8 days (min 2 days; max 4357 days).

The primary reasons for ICU admission were respiratory (n = 11), renal (n = 7), neurological (n = 6) and hemodynamic (n = 5) failures. The mean admission APACHE II and SOFA scores were 18.8 ± 9.0 and 8.6 ± 3.4, respectively. The mean blood lactate level was 4.9 ± 1.4 mmol/L. Mechanisms of organ failures were related to tumor lysis syndrome (n = 10), hemophagocytic lymphohistiocytosis (n = 4), lung (n = 4) and liver (n = 2) malignant infiltration, and disseminated intravascular coagulation (n = 2). Acute circulatory failure requiring vasopressors was present prior to chemotherapy in 18 patients, and was secondarily triggered by chemotherapy in 6 patients. In the first group, administration of chemotherapy was associated with a dramatic improvement in the circulatory conditions (Fig. 19) as assessed by the trend in the hemodynamic SOFA variable. The in-ICU and in-hospital mortality rates were 75 and 79%, respectively. The main cause of death was untractable multiple organ failure (n = 14).

**Fig. 19:**
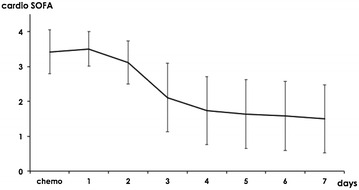
Evolution of cardiovascular SOFA of patients with hemodynamic failure before chemotherapy, from initiation of chemotherapy until 7 days after

**Discussion** The mechanism of organ failures in this setting is questionable, but is presumably linked to a massive release of pro-inflammatory cytokines and endogenous danger signals by malignant cells. Modulation of this overwhelming inflammatory response may represent a field of investigation as well as a potential therapeutic target in the future.

**Conclusion** Urgent administration of chemotherapy is associated with fast improvement in circulatory conditions in patients with sepsis-like shock related to hematological malignancies. However, the overall outcome remains very poor.

**Competing interests** None.

#### P53 PTP1B endothelial gene deletion limits glucotoxicity pathways in endotoxemia model

##### Sarah Fert^1^, Eugénie Delile^1^, Emmanuel Besnier^2^, David Coquerel^3^, Rémi Nevière^4^, Vincent Richard^3^, Fabienne Tamion^5^

###### ^1^Université de rouen, INSERM U1096, Rouen, France; ^2^Pôle Réanimations Anesthésie SAMU, Hospital Center University Rouen, Rouen, France; ^3^U1096, INSERM, Rouen, France; ^4^Inserm u995 equipe 4, Université Lille 2, Lille, France; ^5^Réanimation médicale, Hospital Center University Rouen, Rouen, France

####### **Correspondence:** Fabienne Tamion - fabienne.tamion@chu-rouen.fr

*Annals of Intensive Care* 2017, **7(Suppl 1)**:P53

**Introduction** Glucotoxicity is known to occur during hyperglycemia and glycemic variability, situations that are associated with poor outcomes in sepsis patients. Although cardiovascular dysfunction is a major cause of mortality during sepsis, the impact of glucotoxicity on the cardiovascular system during sepsis remains unknown [1]. Beneficial effects of protein tyrosine phosphatase 1B (PTP1B) deletion, a negative regulator of insulin signaling, on glucose homeostasis and cardiovascular dysfunction during endotoxemia have been reported. We hypothesized that exogenous glucose administration during inflammation increases cardiovascular dysfunctions by activating glucotoxicity pathways and that this is prevented by endothelial PTP1B gene deletion.

**Materials and methods** For this purpose, we generated an endotoxinic model with glucose administration. EndoPTP1B−/− or *wild type* (WT) mice received LPS (1 mg/kg) or saline solution followed by five injections of glucose (2 g/kg) or saline solution each hour 12 h after LPS. Endothelial deletion of PTP1B was generated by crossing LoxP-PTP1B with Tie2-Cre mice. The exploration of the cardiac function is performed 20 h after LPS injection (H_2_O) by the non-invasive echocardiography on anesthetized mice. The gene expression of PARP1(Poly (ADP-ribose) polymérase 1), iNOS, eNOS, IL1-β, TNF-α, IL-10, IL-6, PKC δ, GRP78 (78 kDa glucose-regulated protein), gp91phox, CD-45, CHOP (C/EBP Homologous protein), PTP1B, ICAM-1, VCAM-1 and Caspase 3 is evaluated by q-RT-PCR and Western-Blot.

**Results** The comparison between groups WT and WT endoPTP1B−/− receiving glucose shows an effect of endothelial deletion of PTP1B−/− on the average blood glucose for the LPS-Glc groups. The fractional shortening tended without significance to decrease in mice receiving LPS with glucose compared LPS alone. Analysis of cardiac tissue showed a significant deleterious impact of LPS and glucose association on oxidative stress (gp91phox) and inflammatory responses with increased cytokines production and expression of adhesion molecules (VCAM-1 and ICAM-1). The cardiac gene expression of CHOP and GRP78 (endoplasmic reticulum stress) in WT showed no change in the condition LPS with glucose. Moreover, expression of PARP1, sensor of DNA damages and activator of glucotoxicity pathways, is increased with glycemic variability. Despite an increased expression of PTP1B when LPS and glucose are combined in *wild type* mice, endothelial PTP1B deletion gene only reduced significantly the cardiac expression of iNOS, PARP1 and PTP1B. Finally, cardiac systolic function tended without significance to be impaired by glucose and LPS combined.

**Discussion** The absence of variation in the gene and protein expression of CHOP and GRP78 shows a lack of endoplasmic reticulum stress in our model despite cellular stresses generated by glucose.

**Conclusion** In endotoxinic model, the variability of blood glucose aggravates the effects induced by an inflammatory trigger in the cardiac tissue without significant impaired cardiac function in this model. Endothelial deletion in PTP1B, involved in the regulation of glucose homeostasis, provides improved glycemic control, with a reduction of pathological activation of iNOS, a marker of abnormal vascular function, and PARP1 marker DNA damage.

**Competing interests** None.

**Reference**

1. Brownlee M. The pathobiology of diabetic complications: a unifying mechanism. Diabetes. 2005;54:1615–25.

#### P54 Effects of low doses of esmolol on cardiac and vascular function in experimental septic shock

##### Chaojie Wei^1^, Huguette Louis^1^, Schmitt Margaux^1^, Albuisson Eliane^2^, Orlowski Sophie^3^, Bruno Levy^4^, Antoine Kimmoun^4^

###### ^1^U1116, Faculté de Médecine de Nancy, Vandœuvre-lès-Nancy, France; ^2^Unité espri-biobase, CHRU Nancy, Vandœuvre-lès-Nancy, France; ^3^Service de biochimie, CHRU de Nancy, Vandœuvre-lès-Nancy, France; ^4^Service de réanimation médicale brabois, CHRU de Nancy, Vandœuvre-lès-Nancy, France

####### **Correspondence:** Antoine Kimmoun - akimmoun@gmail.com

*Annals of Intensive Care* 2017, **7(Suppl 1)**:P54

**Introduction** Administration of a selective β1-blocker, such as Esmolol, in septic shock has demonstrated cardiovascular protective effects related to a down-regulation of inflammation. However, the administered dose systematically induced a reduction in heart rate of approximately 20%, thus limiting its prescription at bedside. The present study aimed to determine whether a non-chronotropic dose of Esmolol still maintains its protective cardiovascular and anti-inflammatory effects in experimental septic shock.

**Materials and methods** Four hours after cecal ligation and puncture (CLP), Wistar male rats were randomly allocated to the following groups (n = 8): CLP, CLP + E1 (Esmolol: 1 mg kg^−1^ h^−1^), CLP + E5 (Esmolol: 5 mg kg^−1^ h^−1^), CLP + E18 (Esmolol: 18 mg kg^−1^ h^−1^). An additional eight rats underwent Sham operation. All rats received a continuous infusion of saline, analgesic and antibiotics 4 h after the surgery. Assessment at 18 h included in vivo cardiac function by echocardiography and ex vivo vasoreactivity by myography. Circulating cytokine levels (IL-6 and IL-10) were measured by ELISA. Cardiac and vascular protein expressions of p-NF-κB/IκBα, iNOS, p-AKT/AKT and p-eNOS/eNOS were assessed by Western blotting.

**Results** CLP induced tachycardia, hypotension, cardiac output reduction, hyperlactatemia and vascular hypo-responsiveness to vasopressors. Compared to CLP animals, heart rate was unchanged in CLP + E1 and CLP + E5 but was reduced in CLP + E18. Stroke volume, cardiac output, mean arterial pressure and lactatemia were improved in CLP + E1 and CLP + E5 while vascular responsiveness to Phenylephrine was only improved in CLP + E5 and CLP + E18. Plasma IL-6 levels were decreased in all Esmolol groups. p-NF-κB was decreased in both cardiac and vascular tissues in CLP + E5 and CLP + E18.

**Conclusion** In experimental septic shock, low doses of Esmolol, still improved cardiac function and vasoreactivity. These benefits appear to be associated with a modulation of inflammatory pathways.

**Competing interests** Dr Kimmoun and Pr Levy received fees from Baxter. The remaining authors have disclosed that they do not have any potential conflicts of interest.

**References**

1. Wei C, et al. If channel inhibition with ivabradine does not improve cardiac and vascular function in experimental septic shock. 2016;46(3):297–303.
2. Kimmoun A, et al. beta1-adrenergic inhibition improves cardiac and vascular function in experimental septic shock. Crit Care Med 2015;43:e332–40.

#### P55 Is there an inflammatory rebound upon discontinuation of cisatracurium in critically ill patients?

##### Zakaria Riad^1^, Hodane Yonis^1^, Mylène Aublanc^1^, Sophie Perinel-Ragey^1^, Floriane Lissonde^1^, Aurore Louf-Durier^1^, Romain Tapponnier^1^, Jean-Christophe Richard^1^, Claude Guérin^1^

###### ^1^Réanimation médicale, Hôpital de la Croix-Rousse, Lyon, France

####### **Correspondence:** Zakaria Riad - zakaria.riad@icloud.com

*Annals of Intensive Care* 2017, **7(Suppl 1)**:P55

**Introduction** For patients treated with cisatracurium, it has been shown that proinflammatory cytokines (IL-1, IL-6, and IL-8) significantly went down during the 48 h of molecule administration as compared to placebo [1]. This anti-inflammatory effect is mediated in part by an inhibition of nicotinic acetylcholine receptor [2]. We did not find in the literature any study exploring the post-discontinuation phase of neuromuscular blocking agent apart the fact that blood oxygenation improved after interruption of cisatracurium infusion. The goal of present study was to demonstrate inflammatory rebound defined from the systemic inflammatory response syndrome (SIRS) criteria after discontinuation of cisatracurium.

**Patients and methods** It was a prospective, single-center, observational study. We included adult patients admitted to the medical intensive care unit of the Croix-Rousse hospital in Lyon, who received mechanical ventilation and cisatracurium infusion between February and August 2016. A rapid intravenous infusion of 15 mg of cisatracurium was administred followed by a continuous infusion of 37.5 mg per hour for at least 24 h. After inclusion in the study, SIRS criteria were monitored daily from cisatracurium onset until the 72th hour after molecule interruption. The SIRS was defined by the presence of at least 2 of the followings: temperature >38 °C or <36 °C, heart rate >90/min, respiratory rate >20/min or PaCO_2_ <32 mmHg and white blood cells count >12 G/L or <4 G/L or >10% immature neutrophils. Inflammatory rebound was defined as the increase of SIRS by at least 1 criterion from the baseline defined as the cisatracurium discontinuation. The primary outcome was the prevalence of inflammatory rebound 24 h after the cisatracurium discontinuation.

**Results** Thirty-nine patients were enrolled. The prevalence of inflammatory rebound 24 h after the cisatracurium discontinuation was 56.4%. No risk factor in the multivariate analysis was associated with the presence of inflammatory rebound and this rebound did not affect mortality. We also noticed an increased risk of inflammatory rebound with time after cessation of cisatracurium (Fig. 20) and this risk was not depending on the length of cisatracurium administration before its discontinuation.

**Fig. 20:**
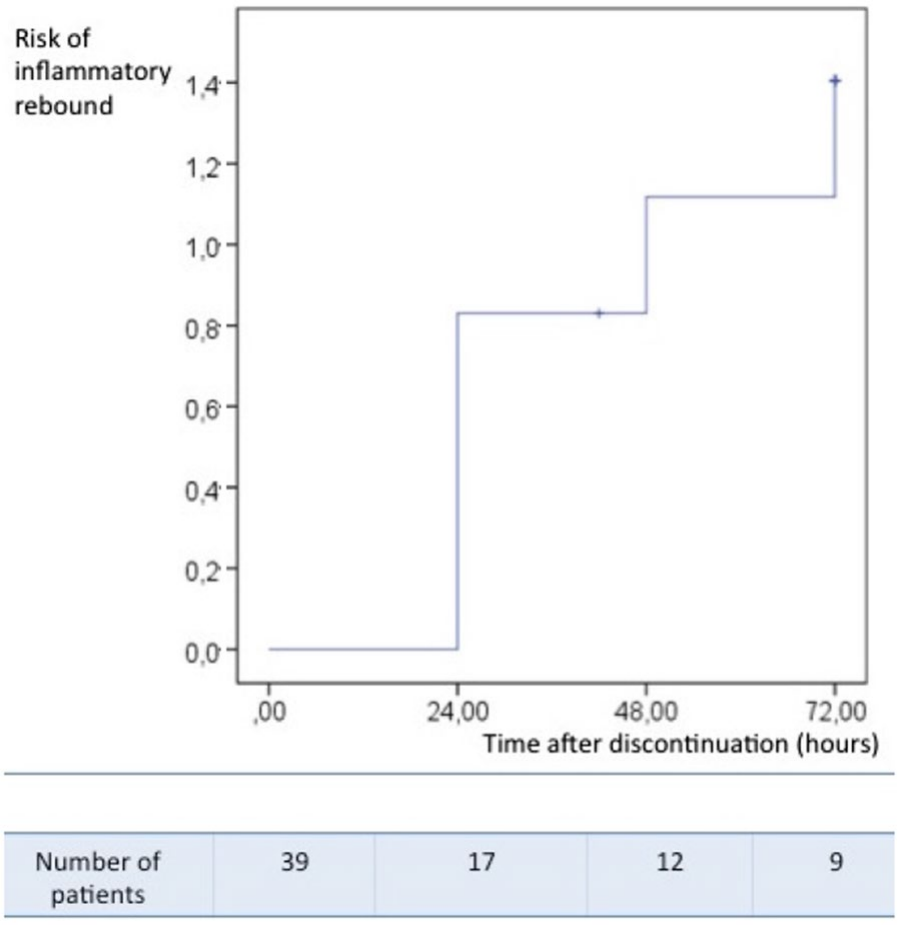
Cumulative risk of inflammatory rebound over time after cisatracurium discontinuation)

**Conclusion** This study demonstrated that inflammatory rebound occurred in more than half of patients after interruption of cisatracurium infusion and its risk of occurrence increased with time.

**Competing interests** None.

**References**

1. Forel JM, et al. Neuromuscular blocking agents decrease inflammatory response in patients presenting with acute respiratory distress syndrome. Crit Care Med. 2006;34:2749–57.
2. Fanelli V, et al. Neuromuscular blocking agent cisatracurium attenuates lung injury by inhibition of nicotinic acetylcholine receptor-α1. Anesthesiology. 2016;(1):132–40.

#### P56 Epidemiology and risk factor of femoral cannula infections after venoarterial extracorporeal membrane oxygenation in cardiac surgery

##### Marine Coroir^1^, Charles Vidal^1^, Bernard Rémy^1^, Bombled Camille^1^, Dimitri Margetis^1^, Adrien Bouglé^1^, Julien Amour^1^

###### ^1^Réanimation chirurgicale cardiovasculaire et thoracique, Groupe Hospitalier Pitié-Salpêtrière, Paris, France

####### **Correspondence:** Marine Coroir - marine.coroir@aphp.fr

*Annals of Intensive Care* 2017, **7(Suppl 1)**:P56

**Introduction** The Veno-Arterial ExtraCorporeal Life Support (VA-ECLS) is an efficient therapy in refractory cardiogenic shock. Nevertheless, VA-ECLS is associated with serious infectious complications. The objective of this study was to investigate the epidemiology and the risk factors of infection of femoral cannulation site in patients with VA-ECLS after cardiac surgery.

**Materials and methods** We investigated all patients underwent VA-ECLS after cardiac surgery between January 2013 and December 2014, all included in the SARIC database. The infection of inguinal cannulation site was defined as an inflammatory or purulent appearance of the cannulation site associated with a positive quantitative culture of the cannulation site sample. The «infected» and «non-infected» patients were compared by Wilcoxon or Fisher tests. A test of Log Rank and a Cox model was used for univariate and multivariate analysis.

**Results** At all, 142 patients were investigated. Diagnosis of cannulation infection site was made in 38 (27%) patients. The median time to infection was 10 days [8; 15] (Fig. 21). Pseudomonas aeruginosa, Enterococcus faecalis, Escherichia coli and Enterobacter cloacae were identified in 16, 12, 12 and 10% respectively. The other bacteria were klebsiella pneumoniae in 15% and staphylococcus in 10%. In univariate analysis, risk factors of cannula site infection were a multiresistant bacterial colonization, a bacteremia event and/or a bleeding event in VA-ECLS cannulation site. In multivariate analysis, only a bleeding event in VA-ECLS cannulation site was identified as a risk factor cannulation site infection.

**Fig. 21:**
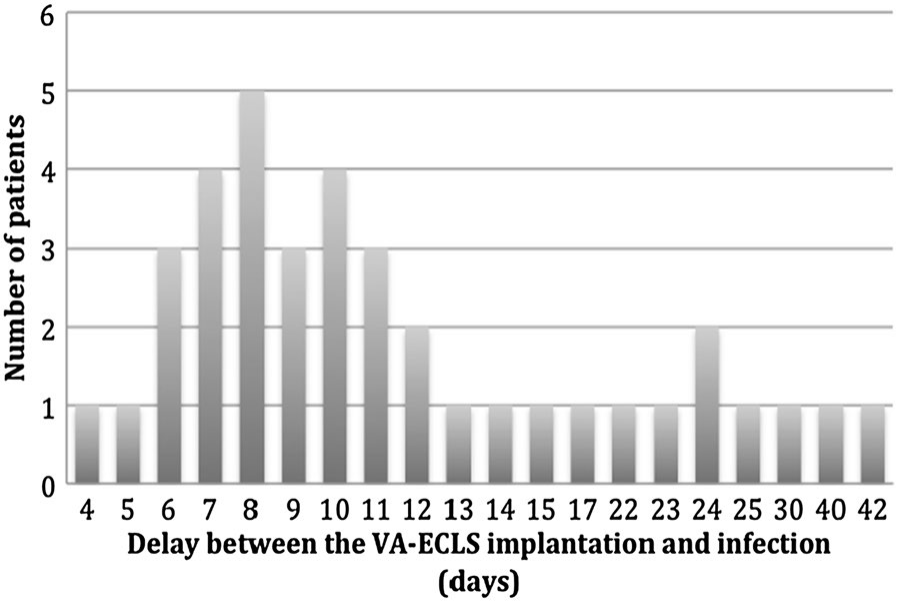
Delay between the VA-ECLS implantation and infection of the inguinal cannulation site (n = 38)

The intrahospital mortality was similar in both groups but the hospitalization length stay increased in the «infected» group, 45 vs. 26 days (p < 0.0001) respectively. Furthermore, reoperation increased significantly in the «infected» group, 71 vs. 2% of patients (p < 0.0001) respectively. After reoperation, a reconstructive surgery of the cannulation site was necessary in 45% of cases.

**Conclusion** In patients treated with VA-ECLS after cardiac surgery, inguinal cannulation site infection occurs in 27% of patients. Bleeding event is the main risk factor.

**Competing interests** None.

**Reference**

1. Schmidt M, et al. Nosocomial infections in adult cardiogenic shock patients supported by venoarterial extracorporeal membrane oxygenation. Clin Infect Dis Off Publ Infect Dis Soc Am. 2012;55:16–1641.

#### P57 Infective endocarditis in French ICUs: trends during the 1997–2014 period and impact of antibioprophylaxis guidelines change

##### Jeremie Joffre^1^, Philippe Aegerter^2^, Vincent Dubée^3^, Naïke Bigé^3^, Gabriel Preda^4^, Jean-Luc Baudel^3^, Eric Maury^5^, Hafid Ait-Oufella^3^, Bertrand Guidet^3^

###### ^1^Service de reanimation médicale, Hôpital Saint-Antoine, Paris, France; ^2^Urc, Hospital Ambroise Paré, Boulogne-Billancourt, France; ^3^Réanimation Médicale, Hôpital Saint-Antoine, Paris, France; ^4^Réanimation, Hôpital Saint-Antoine, Paris, France; ^5^Réanimation Médicale, Hôpital Saint-Antoine, AP-HP, Paris, France

####### **Correspondence:** Jeremie Joffre - jeremie.joffre@inserm.fr

*Annals of Intensive Care* 2017, **7(Suppl 1)**:P57

**Introduction** Few studies focused on patients with severe infective endocarditis (IE) in Intensive Care Unit (ICU), and despite major advances in both diagnosis and therapeutic procedures, it still carries poor prognosis. In 2009, European guidelines on antibiotic prophylaxis have changed and became more restrictive. The goal of this study is to describe changes in incidence, characteristics of patients, management, in-hospital mortality and pathogens in an overall population of patients admitted to ICU for a definite IE. Through comparison of two periods we aim to estimate the impact of the amended recommendations issued by European society of cardiology.

**Patients and methods** Retrospective study based on the CUB-Rea register, including 31 medical and/or surgical intensive care units between 1997 and 2014. Statistical analysis compared the periods 1997–2009 and 2010–2014.

**Results** We included 4757 definite IE over the two periods, 2848 in 1997–2009 and 1909 in 2010–2014. We observed a significant increase in crude annual incidence (153.0 ± 14.9 vs 217.6 ± 46.4; *P* = 0.018) and in incidence density relative to number of stays (0.79 ± 0.08 vs 1.1 ± 0.02% *P* = 0.004). Despite a trend towards increasing number of cases over the first period (1997–2009), slopes of incidence density curves clearly indicate an acceleration of the number of cases since 2009 (*P* = 0.017). Patients treated during the second period are significantly older and more severe than those treated before 2009. Surprisingly, use of invasive ventilation, renal replacement therapy, and vasopressor were significantly lower during second period. Contrariwise, resort to surgery has doubled between two periods. ICU mortality is significantly lower in the second period but in-hospital mortality remains unchanged. Concerning pathogens, we found a significant increase in incidence of *Streptococcus* spp. and *Staphylococcus* spp., and no changes concerning intracellular bacteria, *Enterococcus* spp., C*andida spp*. or Gram-negative bacilli.

**Conclusion** Despite some limitations inherent to its retrospective design and to potential diagnostic coding bias, our study highlights a quick shifting landscape in the epidemiology of infectious endocarditis in intensive care, characterized by a strong increase in the incidence and changes in bacterial epidemiology. Restrictive bend in antibiotic prophylaxis guidelines could be substantially responsible for these trends.

**Competing interests** None.

#### P58 Early identification of heparin-induced thrombocytopenia in surgical intensive care patients by using the HIT Expert Probability score: a pilot study

##### Dejan Ilic^1^, Guillaume Besch^1^, Marc Ginet^1^, Caroline Pignard^1^, Philippe Nguyen^2^, Guillaume Mourey^3^, Emmanuel Samain^1^, Sebastien Pili-Floury^1^

###### ^1^Réanimation chirurgicale, CHU Jean Minjoz, Besançon, France; ^2^Laboratoire d’hématologie, Hopital Robert Debré, Reims, France; ^3^Laboratoire d’hémostase, cytologie et plateforme de biomonitoring, EFS Bourgogne Franche-Comté, Besancon, France

####### **Correspondence:** Dejan Ilic - dejan.ilic@sfr.fr

*Annals of Intensive Care* 2017, **7(Suppl 1)**:P58

**Introduction** Heparin is widely prescribed in patients admitted in surgical intensive care units (SICU) to prevent venous thromboembolic events. Heparin-induced thrombocytopenia (HIT) is a rare but potentially life-threatening complication of heparin therapy. HIT requires the emergent discontinuation of heparin and the prescription of an alternative anticoagulant therapy that could be difficult to manage in SICU patients and enhance the risk of hemorrhagic complication. The early diagnosis of HIT in SICU patients remains a challenge. As thrombocytopenia could reveal several SICU complications, the 4T score of Warkentin is a useless tool to efficiently discriminate patients having or not HIT and the biological confirmation of HIT is delayed. The HIT Expert Probability (HEP) score has been reported to have a higher predictive value than the 4T score in non-ICU patients. The purpose of the study was to compare the HEP score to the 4T score in the early diagnosis of HIT in SICU patients.

**Materials and methods** We conducted a one-center prospective observational cohort pilot study (www.ClinicalTrials.gov Identifier NCT02790567), included all consecutive patients admitted in our SICU between October 2013 and May 2015 and suspected to have HIT. Non-inclusion criteria were pregnancy, age <18 years old and treatment with fondaparinux. The day the diagnosis of HIT was suspected, the HEP and the 4T scores were calculated and the following blood analyses were performed: the ID-PaGIA Heparin/PF4 test, the ELISA test, the heparin-induced-platelet-aggregation (HIPA), and the serotonin release assay (SRA). After completion of the study, all medical files were reviewed by a multi-disciplinary independent committee to discriminate patient having (HIT group) or not (SAFE group) a HIT. The final diagnosis was based on the medical history of the patient, on the time-variation of the platelet count while heparin was discontinued or not, and on the results of the venous Doppler of the 4 limbs. The committee was blinded from the value of the HEP and of the 4T scores, and from the results of the ID-PaGIA Heparin/PF4 test. The ROC curves of the HEP and of the 4T score were constructed. The sensibility (Se), the specificity (Sp), the positive predictive value (PPV), and the negative predictive value (NPV) of the HEP and of the 4T scores were calculated, and the areas under the ROC curves (AUC) were compared by using a Chi2 test. Data are presented as median [interquartile range] and number of patients (percentage).

**Results** From the 119 patients included, 6 (5) patients had a HIT (age: 66 [59–74] vs 70 [54–80] year, p = 0.62; male: 80 (71) vs 6 (100), p = 0.18; IGSII score value: 54 [46–68] vs 46 [38–62], p = 0.25; respectively in the SAFE and in the HIT groups). The global incidence of HIT during the study was 0.43%. The ROC curves are presented in Fig. 22. The Se, the Sp, the PPV and the NPV of a HEP score ≥5 and of a 4T score ≥6 were respectively 100 and 50%, 93 and 86%, 50 and 22%, and 100 and 96%. All patients in the HIT group had a HEP score ≥5. The AUC was significantly higher for the HEP score (AUC [95% confidence interval] 0.967 [0.922–1.000]) than for the 4T score (AUC [95% CI] 0.707 [0.449–0.965]) (p = 0.035).

**Fig. 22:**
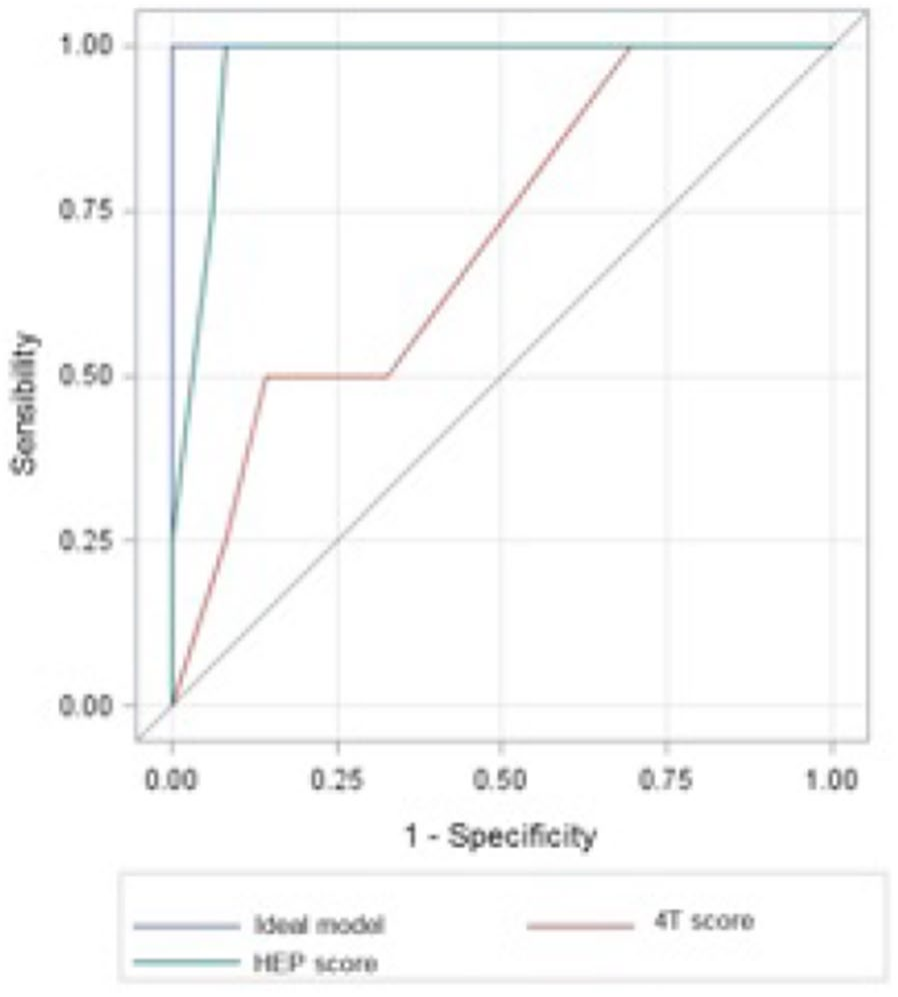
Receiver operating characteristic curves for the HEP and for the 4T scores

**Conclusion** Based on a small prospective observational pilot study, a HEP score ≥5 could have a higher predictive value than a 4T score ≥6 in the early diagnosis of HIT in SICU patients. This result needs to be confirmed in a larger multicenter study.

**Competing interests** None.

#### P59 Purifying efficiency of CVVHDF and MARS during a simulated intoxication pentobarbital

##### Romain Jouffroy^1^, Caill Nicolas^2^, Jean-Claude Alvarez^3^, Maraffi Tomasso^1^, Pascal Philippe^1^, Jean-Herlé Raphalen^4^, Lionel Lamhaut^5^, J Baud Frédéric^1^, Benoit Vivien^6^, Carli Pierre^1^, Frederic Baud^7^

###### ^1^Anesthésie Réanimation SAMU, APHP - CHRU Necker Enfants Malades, Paris, France; ^2^Service de pharmacologie toxicologie, APHP - Hopital Raymond Poincaré, Paris, France; ^3^Paris, Assistance Publique Hôpitaux de Paris, Paris, France; ^4^Service de réanimation adulte, Hôpital Necker, Assistance Publique Hôpitaux de Paris, France; ^5^Réanimation adulte, Hôpital Necker - Enfants Malades, Paris, France; ^6^Réanimation adulte - samu, Hôpital Necker - Enfants Malades, Paris, France; ^7^75014, SAMU de Paris, Réanimation polyvalente, Paris, France

####### **Correspondence:** Romain Jouffroy - romain.jouffroy@aphp.fr

*Annals of Intensive Care* 2017, **7(Suppl 1)**:P59

**Introduction** Drug poisoning is a frequent cause of hospital admission especially in intensive care unit (ICU). Despite advances in treatment, hospital mortality of severe acute poisoning admitted in ICU seems to increase. Purifying methods, continuous haemodiafiltration veinoveinous (CVVHDF) and molecular adsorbent recirculating system (MARS) were developed with promising clinical results [1]. However no analytical study has quantified their accurately purifying efficiency. It has not been assessed efficiency of the different compartments of MARS nor its advantages over other methods of dialysis and filtration. The objective of this study was to quantify the purifying efficiency of the different compartments of the CVVHDF and MARS and to compare their respective efficiency in an ex vivo model in the most favourable conditions for these methods to assess their maximum capacity purification.

**Materials and methods** We performed an ex vivo study based on a manipulation bench simulating intoxication pentobarbital at a plasma concentration of 40 mg/l injected into a central compartment (5 l) devoid of transporter proteins (200 mg of pentobarbital). The EC extraction coefficient [EC = (in concentration − out concentration)/in concentration] were calculated for each compartment of CVVHDF and MARS as well as the amounts withdrawn by the sum each compartment allows to assess the overall capacity of each technique.

**Results** At the end of 6 h simulation with CVVHDF, the remaining material in the central compartment was 3% of the total quantity injected. The cumulative amount removed in total effluent was equal to 95%. The non-recovered amount was equal to 2%. EC CVVHDF was almost constant with an average value of 14% with large variations however (range 2.5–27%). It was not observed to release at the end of manipulation.

At the end of a 6 h simulation with MARS, the remaining amount in the central compartment was undetectable. The cumulative amount removed in total effluent from the hemodiafiltration column was equal to 11.5% the amount removed by carbon column was 88.5%. The sum of the amounts removed by the effluent and the carbon column reflects the total amount injected. The EC hemodiafiltration compartments and charcoal hemoperfusion were 25 and 70% respectively. Purification of pentobarbital was complete after 3 h of a MARS session.

**Discussion** MARS is the most effective purifying method in a pentobarbital-simulated intoxication. This rapid and complete treatment is mainly due to purifying coal capacity. It was not observed the cartridge saturation for the amount of administered pentobarbital.

**Conclusion** Future studies should determine the parameters that may affect the treatment capacity by both methods.

**Competing interests** None.

#### P60 Plant poisoning: still a current intoxication

##### Hana Fredj^1^, A M’rad^1^, Messaouda Khelfa^1^, Youssef Blel^1^, Nozha Brahmi^1^

###### ^1^Department of intensive care and toxicology, Centre d’Assistance Médicale Urgente, Tunis, Tunisia

####### **Correspondence:** Youssef Blel - blelyoussef@yahoo.fr

*Annals of Intensive Care* 2017, **7(Suppl 1)**:P60

**Introduction** Plant poisoning is generally not life-threatening. Its occurrence in children is commonly accidental. In adults it results from a suicide attempt or from its use for addictive or therapeutic purposes.

Because of their specificity in clinical presentation and outcomes, we have conducted this study.

The objective of this study was to report all plant poisoning cases collected in Tunisian toxicological intensive care unit, with their epidemiological, clinical characteristics and outcomes.

**Patients and methods** A retrospective study was performed between January 2007 and December 2015.

Epidemiological data and clinical outcomes were reviewed. Data were analyzed using SPSS.

**Results** During the study period, 38 patients were included. Sex ratio was 1. Mean age was 43 years (5–65). Poisoning was accidental in 88% of cases. Most frequently incriminated plants were Datura stramonium (40%) with an anticholinergic toxidrom present in all cases, Ricinus communis (23%) with gastrointestinal manifestations (present in 88% of cases), Nerium oleander (9%) with digitalis toxicity–like symptom in 66% of cases, Hyoscyamus Niger (12%) with anticholinergic symptoms in 75% of cases and hallucinogenic effects in all cases, Atractylis gummifera (9%) with gastro-intestinal and acute liver failure symptoms, and Peganum harmala (9%) with only gastro-intestinal effects. All patients received supportive care. Mortality rate was 8, 5%, interesting children, and was secondary to multi-system organ failure due to ingestion of Atractylis gummifera.

**Conclusion** Each year 2.2% of our poison center calls report exposures to toxic plants. Most of these exposures are of minimal toxicity largely because of the fact that they involve pediatric ingestions, which are of low quantity. Public education is important to minimize these poisonings and must be oriented primarily towards children to reduce mortality.

**Competing interests** None.

#### P61 Toxic effects of rhamnus alaternus: a collective intoxication

##### Hassen Ben Ghezala^1^, Salah Snouda^2^, Chiekh Imen Ben^3^, Moez Kaddour^2^

###### ^1^Réanimation Médicale, Hôpital Henri Mondor, Avenue du Maréchal de Lattre de Tassigny, Créteil, France, Créteil, France; ^2^Réanimation Médicale, Hopital regional zaghouan, faculté de médecine de Tunis, Zaghouan, Tunisia; ^3^Teaching department of emergency and intensive care, Regional hospital of Zaghouan, Zaghouan, Tunisia

####### **Correspondence:** Hassen Ben Ghezala - hassen.ghezala@gmail.com

*Annals of Intensive Care* 2017, **7(Suppl 1)**:P61

**Introduction** Herbal remedies have been used for centuries to treat a variety of diseases. *Mediterranean Buckthorn (Rhamnus alaternus*) has been used for therapeutic purposes and no toxicity effects have been documented. Rhamnusalaternus (Rhamnaceae) is a small tree located mainly in the North of Tunisia, where it is known as ‘‘Oud El-khir”. It has traditionally been used as a diuretic, laxative, hypotensive drug and for the treatment of diabetes, hepatic and dermatologic complications. Previous phytochemical studies have shown potent antioxidant, free radical scavenging, antimutagenic and antigenotoxic activities of flavonoids and phenol isolated from Rhamnus alaternus roots and leaves.

**Patients and methods** It was a retrospective study reporting a family collective poisoning which occurred in the region of Zaghouan in Tunisia in July 2015. All the members of the family ingested accidentally a traditional preparation of a plant “Oud El-Khir” in a juice prepared for a traditional marriage.

**Results** On 1st July 2015, a family composed of seventeen members (ten men and seven women) was admitted to the teaching emergency and intensive care department of the regional hospital of Zaghouan (Tunisia). All members of the family presented dizziness, weakness, anorexia and dyspnea. They reported the ingestion 10 h before of a juice in a traditional marriage. This juice was prepared using a plant called “Oud El-Khir” because of its capacity to be a lucky charm for the new married. The mean blood pressure was 130/60 mmHg. All members of the family experienced nausea, vomiting, anuria and hematuria. On physical examination, five members of the family had myalgia without other clinical signs.

For all patients: cytological reports and sputum smear were negative (three times) for pulmonary tuberculosis. Hepatitis B and C serology were also negative. Chest x-ray was normal; Blood and urine culture were negative. In renal ultrasonography performed in five members of the family, there was a significant difference in kidney sizes and the corticomedullary differentiation was altered. Laboratory tests showed hyperglycemia and renal failure with metabolic acidosis in ten patients. Three dialysis sessions were performed.

Samples of the herbal decoction were obtained from the juice. It was a dark brown suspension with fine brown deposit and a clear supernatant. It smelled a strong penetrating odor. Samples of both Rhamnus alaternus roots and its decoction were sent to be analyzed in the laboratory of toxicology in the Center for Emergency Medical Assistance of Tunis in Tunisia.

Screening by GC–MS of both *Rhamnus alaternus* roots and infusion extracts, revealed the presence of anthraquinone glycosides such as 4,5-dihydroxy-9,10-dioxoanthracène-2-carboxylic acid (Rhein), 1,8-dihydroxy-3-(hydroxymethyl)-9,10-anthracenedione (Aloe-emodin) and 1,8-dihydroxy-3-methoxy-6-methylanthracene-9,10-dione (Physcion). The retention times were 8.95, 9.67 and 10.25 min respectively. Anthraquinone glycosides were detected in a dichloromethane extract and ethyl acetate extract at pH value = 9 and only in a dichloromethane extract at pH value = 7 by GC–MS analysis.

**Conclusion** Rhamnus alaternus can be toxic when used in an abusive way besides its strong antibacterial, antioxidants and antidiabetic activities. To our knowledge, this is the first report of cases of renal failure and rhabdomyolysis which is possibly associated with an accidental consumption of *Rhamnus alaternus* roots. We present these cases to illustrate the role of both clinical and biological investigations in handling cases of herbal poisonings. We aimed also to increase awareness among emergency physicians about patients presenting to the emergency department with unexplained symptoms (renal failure, rhabdomyolysis…) that requires prompt diagnosis so that such life-threatening complications can be avoided.

**Competing interests** None.

#### P62 Electroencephalographic patterns of lithium poisoning: a study of the effect/concentration relationships in the rat

##### Anne-Sophie Hanak^1^, Isabelle Malissin^2^, Joel Poupon^3^, Patricia Risede^1^, Lucie Chevillard^1^, Bruno Megarbane^2^

###### ^1^Inserm u1144, Paris-Descartes University, Paris, France; ^2^Department of medical and toxicological critical care, Lariboisière Hospital, Paris, France; ^3^Laboratory of toxicology, Lariboisière Hospital, Paris, France

####### **Correspondence:** Bruno Megarbane - bruno.megarbane@aphp.fr

*Annals of Intensive Care* 2017, **7(Suppl 1)**:P62

**Introduction** Lithium overdose may result in encephalopathy and electroencephalographic abnormalities. Three poisoning patterns have been identified based on the ingested dose, previous treatment duration and renal function. Whether severity of lithium-induced encephalopathy depends on the poisoning pattern is not established.

**Materials and methods** We designed a rat study to investigate lithium-induced encephalopathy and correlate its severity to plasma, erythrocyte, cerebrospinal fluid and brain lithium concentrations previously determined in rat models mimicking human poisoning patterns. Lithium-induced encephalopathy was assessed and scored using continuous electroencephalography.

**Results** We demonstrated that lithium overdose was consistently responsible for encephalopathy which severity depended on the poisoning pattern. Acutely poisoned rats developed rapid-onset encephalopathy which reached a maximal grade of 2/5 at 6 h and disappeared at 24 h post-injection. Acute-on-chronically poisoned rats developed persistent and slightly fluctuating encephalopathy which reached a maximal grade of 3/5. Chronically poisoned rats developed rapid-onset but gradually increasing life-threatening encephalopathy which reached a maximal grade of 4/5. None of the acutely, 20% of the acute-on-chronically and 57% of the chronically lithium-poisoned rats developed seizures. The relationships between encephalopathy severity and lithium concentrations fitted a sigmoidal Emax model based on cerebrospinal fluid concentrations in the acute poisoning and brain concentrations in the acute-on-chronic poisoning. In the chronic poisoning, encephalopathy worsening paralleled the increase in plasma lithium concentrations.

**Conclusion** Severity of lithium-induced encephalopathy is dependent on the poisoning pattern, previously shown to determine the lithium amount accumulated in the brain. Our data supports that electroencephalography is a sensitive tool to score lithium-related neurotoxicity.

**Competing interests** None.

#### P63 Electrocardiogram changes in amitriptyline poisoning

##### Manel Barghouth^1^, Aymen M’rad^1^, Marwa Ben Hmida^1^, Amira Ben Jazia^1^, Hafedh Thabet^2^, Youssef Blel^1^, Nozha Brahmi^1^

###### ^1^Department of intensive care and toxicology, Centre d’Assistance Médicale Urgente, Tunis, Tunisia; ^2^Department of emergency, Centre d’Assistance Médicale Urgente, Tunis, Tunisia

####### **Correspondence:** A M’rad - mrad.aymen@gmail.com

*Annals of Intensive Care* 2017, **7(Suppl 1)**:P63

**Introduction** Amitriptyline is one of the common drug poisoning that induces severe cardiovascular and neurological complications.

The incidence of electrocardiogram changes in Amitriptyline poisoning was not explored enough. Therefore, we conducted this study to determinate the incidence of electrocardiogram abnormalities in Amitriptyline poisoning.

**Patients and methods** It was a retrospective study conducted from January 2012 to July 2016 in a toxicological unit including all admitted patients for Amitriptyline poisoning. A 12-lead electrocardiogram was carried out and analyzed at admission and every 6 h.

**Results** One hundred and fifty patients aged 29 ± 12 years were included; their sex ratio was of 0.19. Among them, only 6 had a history of cardiovascular disease.

A loss of consciousness was noted in 58% of patients (n = 87) and electrocardiogram changes in 42% of patients (n = 63). The most common electrocardiogram changes were sinus tachycardia (108 ± 13 beats/min) (60%), widening of the QRS complex (16%) and right bundle ranch block (12%). A changes in die S-T was observed in 4 patients and dominant secondary R wave (R’ > 3 mm) in aVR in 3. Forty-four percent of patients with electrocardiogram changes were comatose. No patient had developed hemodynamic instability especially in the case of widening of the QRS. These electrocardiogram abnormalities took 6 to 36 h to regress without specific treatment.

**Conclusion** Although electrocardiogram changes are common in Amitriptyline poisoning particularly in a patients with loss of consciousness, it rarely induced hemodynamic instability.

**Competing interests** None.

#### P64 Tramadol-related neurotoxicity in the rat: contributions of the different neuromediators and effects of potential antidotes

##### Hao Liang^1^, Lucie Chevillard^1^, Jacques Callebert^1^, Camille Lagard^1^, Patricia Risede^1^, Bruno Megarbane^2^

###### ^1^Inserm u1144, Paris-Descartes University, Paris, France; ^2^Department of medical and toxicological critical care, Lariboisière Hospital, Paris, France

####### **Correspondence:** Bruno Megarbane - bruno.megarbane@aphp.fr

*Annals of Intensive Care* 2017, **7(Suppl 1)**:P64

**Introduction** Tramadol, an opioid analgesics used to treat moderate to severe pain, is responsible in overdose for coma, respiratory depression, seizures and serotonin syndrome. The exact role of naloxone to reverse tramadol-related effects is debated. We aimed at investigating the pathways involved in tramadol-related neurotoxicity and seizures in the rat, by using various antagonists of the different tramadol-mediated effects including naloxone, cyproheptadine, fexofenadine and diazepam and determining the turnover of brain monoamines.

**Materials and methods** Body temperature (using telemetry), respiratory effects (using plethysmography) and neurological effects (using clinical scales and EEG) were studied. Brain (frontal lobes) monoamines (serotonin, dopamine and norepinephrine) and their respective metabolites were measured using HPLC coupled to fluorimetry. For each animal and each time, we calculated the difference between the parameter value at that time and baseline and the area under the curve of its time course. Comparisons were performed using two-way ANOVA followed by post-tests using Bonferroni correction.

**Results** Tramadol induced sedation (p < 0.01), seizures (early onset and peaking at 30 min) and increase in inspiratory time (p < 0.001) as well as a non-significant trend to hypothermia,. Diazepam completely suppressed seizures Naloxone prevented tramadol-related sedation and respiratory effects while did not inhibit seizures. In contrast to cyproheptadine which exhibited no effects, fexofenadine partially reduced seizures, suggesting the involvement of a histaminergic pathway. Monoamines turnovers were significantly reduced in the presence of diazepam (p < 0.01), suggesting that diazepam-mediated prevention of tramadol-induced seizures could be related to the inhibition of monoamines metabolism in addition to its usual GABAergic effects.

**Conclusion** Tramadol-induced sedation and respiratory effects are mediated by mu-opioid receptors. Seizures involve complex mechanisms including histaminergic but not serotoninergic pathways. Diazepam-related anticonvulsive activity to prevent tramadol-induced seizures may be related to the inhibition of monoamines metabolism in addition to its GABAergic effects.

**Competing interests** None.

#### P65 Acute insulin poisoning: What about frequency?

##### Sahar Habacha^1^, Bassem Chatbri^1^, A M’rad^1^, Youssef Blel^1^, Nozha Brahmi^1^

###### ^1^Departement of intensive care and toxicology, Centre d’Assistance Médicale Urgente, Tunis, Tunisia

####### **Correspondence:** Sahar Habacha - sahar.habacha@gmail.com

*Annals of Intensive Care* 2017, **7(Suppl 1)**:P65

**Introduction** Suicide by injecting insulin is not uncommon in diabetics and non-diabetics but few series were described in literature. Our study aimed to describe insulin intoxication in a series of cases.

**Materials and methods** Our study is retrospective. It included all patients admitted for insulin intoxication in the period between 2012 and 2015. Insulin poising was withheld on medical history of insulin injection without therapeutic indication, on clinical signs of hypoglycemia (sweats, neurologic and digestive signs…), and on capillary glucose under 0.5 g/L in non-diabetics and under 0.6 g/L in diabetics, at least once.

**Results** Forty-three patients were collected, with sex-ratio at 0.72. The mean age was 31 ± 13 years. Only 32% of patients were diabetics. The median amount of injected dose was 120 UI [50; 302]. Rapid-acting insulin (RI) was found in 30%, lente insulin (LI) in 58% and mixed intoxication in 7%. Route of administration was in all cases subcutaneous. Time of onset of symptoms was 1H and 45 min with extremes of 15 min and 2 h for RI. It was 3H with extremes of 1 and 6 h for LI. The most common signs were vertigo (53.5%), sweats (42%), palpitations (23%), coma (21%), paresthesia (18%), nausea (14%), seizures (11%). The median capillary glycaemia on the first medical contact was 0.5 g/L [0.36; 0.71]. The median required dose of injected carbohydrate was 175 g [90; 200]. Central catheter was required in only one case. Outcome was favorable in 93% (n = 40) with hospital discharge in 1.5 days ± 0.68. Outcome were fatal in two cases and one patient kept chronic coma because of late care.

**Conclusion** Insulin intoxication had good prognosis if patients received adequate medical care on time.

**Competing interests** None.

#### P66 Tramadol poisoning in the intensive care unit: clinical presentation and prognostic value of plasma tramadol concentration of on admission

##### Christophe Camillerapp^1^, Laurence Labat^2^, Marion Soichot^3^, Isabelle Malissin^1^, Pierre Garçon^1^, Antoine Goury^1^, Lamia Kerdjana^1^, Sebastian Voicu^1^, Nicolas Deye^1^, Bruno Megarbane^1^

###### ^1^Department of medical and toxicological critical care, Lariboisière Hospital, Paris, France; ^2^Inserm u1144, Paris-Descartes University, Paris, France; ^3^Laboratory of toxicology, Lariboisière Hospital, Paris, France

####### **Correspondence:** Bruno Megarbane - bruno.megarbane@aphp.fr

*Annals of Intensive Care* 2017, **7(Suppl 1)**:P66

**Introduction** Tramadol poisonings are significantly increasing due to the increase in prescriptions since dextropropoxyphene banning from the European market in 2011. Tramadol-related analgesic effects are mediated by its antagonist activity on the norepinephrine and serotonin transporters in addition to the agonist activity of its major active metabolite M1 on the mu-opioid receptors. Thus, tramadol overdose may result in various toxicities including central nervous system depression, seizures and serotonin syndrome. The relative prevalence of each of these complications is debated. We aimed (1) to describe the clinical features in tramadol-poisoned patients and (2) to study the prognostic value of the plasma concentration of tramadol and its metabolites on ICU admission.

**Patients and methods** We conducted a prospective single centre observational study including all tramadol-poisoned patients admitted to the intensive care unit (ICU) from 2012 to 2016. The plasma concentrations of tramadol and its metabolites were determined using high-performance liquid chromatography coupled to mass spectrometry. Subgroup comparisons were performed using Chi-2 and Mann–Whitney tests.

**Results** Forty-two tramadol-poisoned patients (41 years [26; 55], median [25; 75 percentiles]; 30 females et 12 males; 90% with poly-intoxications; presumed ingested dose: 2000 mg [1000; 4000]; plasma tramadol concentration on admission: 1.48 mg/L [1.17; 2.34]) were included in the study. The patients presented consciousness impairment (Glasgow coma score: 13 [6; 15]), opioid syndrome (48%), serotonin syndrome (36%) and seizures (24%). Life-threatening complications occurred including pre-hospital cardiac arrest (10%), cardiovascular failure (31%), aspiration pneumonia (34%), disseminated intravascular coagulation (5%) and fatality (7%). There was a significant relationship between plasma tramadol concentration measured on admission and the risk of seizure onset (p < 0.05). Patients presenting opioid syndrome on admission significantly had lower plasma tramadol concentrations on admission than patients presenting serotonin syndrome (p < 0.03).

**Conclusion** Tramadol poisoning may result in significant morbidities requiring invasive ICU management. Onset of seizures is not related to the serotonin syndrome. Measurement of the concentrations of tramadol and its metabolites on ICU admission seems helpful to predict the kind of toxic syndromes presented and the nature of further complications.

**Competing interests** None.

#### P67 Purpura fulminans mortality factors in pediatric intensive care department: about 28 cases

##### Anwar Armel^1^, Benqqa Anas^1^, Samira Kalouch^2^, Khalid Yaqini^2^, Aziz Chlilek^2^

###### ^1^Anesthésie réanimation, CHU Ibn Rochd, Casablanca, Morocco; ^2^Service de réanimation pédiatrique, Chu Ibn Rochd, Casablanca, Morocco

####### **Correspondence:** Anwar Armel - armelanwar@gmail.com

*Annals of Intensive Care* 2017, **7(Suppl 1)**:P67

**Introduction** Purpura fulminans (PF) is one of the biggest pediatrics emergencies. It is a septic shock associated with an extensive purpura complicating most often meningococcal septicemia. With a non casual mortality despite of advances in pediatric intensive care. Our study’s aim is to study purpura fulminans mortality factors in our pediatric intensive care unit.

**Materials and methods** We led a prospective study of a series of 28 cases of purpura fulminans collected in our pediatric intensive care unit, Abderrahim Harouchi child hospital of Casablanca, over a period of 2 years from January 2014 to December 2015.

Statistical analysis used the epi-info test with significance level P ˂ 0.05.

**Results** Our series analysis underlined a slight male predominance (53.6%) and incidence peak in patients of less than 4 years (67.8%). Most of the patients were from a low socioeconomic level (65%). And earlier evolution period to hospitalization relatively long between 4 h and 7 days was noted in our series.

The main symptoms were fever (100%), purpura (28.6%), digestive disorders (42.8%) and neurological signs mainly consciousness disorders (28.6%). The shock, infectious syndrome, purpura, neurological and respiratory disorders dominated the clinical background at admission.

Hyperleukocytosis to polynuclear neutrophils was found in 67.9% of the cases, C-reactive protein was positive in 100% and the hemostatic assessement underlined disseminated intravascular coagulation in 78.9% of the cases. The causative organism was isolated only in 14 cases: meningococcus B in 11 cases (78.6%), pneumococcus in one case, Haemophilus in one case and Acinetobacter in one case.

Therapeutic schema used was based on fluid replacement and antibiotic therapy for all patients, vasoactive drugs (50%), corticosteroids (100%), mechanical ventilation (46.4%) and symptomatic treatment.

The evolution was marked by death in 42.9% of the cases, two patients showed out complications of skin necrosis and distal ischemia requiring necrosectomies. Recovery without sequelae was achieved in 50% of the survivors.

**Conclusion** Mortalities factors found in our series was the time between the first symptoms and hospitalization exceeding 48 h, presence of extensive purpura of a shock on admission, presence of seizures, the use of mechanical ventilation and the presence of disseminated intra vascular coagulation.

**Competing interests** None.

#### P68 Guillain-Barré syndrome prognostic factors in pediatric reanimation

##### Mezgui Othman^1^, S. Moumine, S. Kalouch, K. K. Yakini, A. Chlilek

###### ^1^Anesthesia-Reanimation, CHU Ibn Rochd Casa, Casablanca, Morocco

####### **Correspondence:** Mezgui Othman - o.mezgui@hotmail.fr

*Annals of Intensive Care* 2017, **7(Suppl 1)**:P68

**Introduction** Guillain-Barré syndrome (GBS) is an inflammatory disorder of the peripheral nervous system is idiopathic acute polyneuropathy, which has now become the most common cause of acute flaccid paralysis in children.

**Patients and methods** This is a retrospective study which runs from 2006 to 2015 performed in the pediatric reanimation

**Results** Guillain-Barré syndrome was diagnosed in 50 patients aged 9–15 years, the predominant involvement in sex with male enrollment ratio of 1.7 men/women

After a prodromal event, usually infectious (96%) and a free interval of 15 days on average, start motor disorders.

These are of two types:

- Consider a hypo or areflectique flaccid paralysis of the lower limbs (25%) of ascending evolution in 91.4% of cases.
- Let flaccid tetraplegia or hypo areflectique (75%)

The respiratory reached was observed in 64% of cases also other serious signs such as swallowing disorders (70%) and autonomic disturbances (14%) were also observed which justified a decision support in intensive care for all our patients.

The use of ventilation was required in 68% of cases and specific treatments based on immunoglobulins were administered in 88% of cases

The death rate is still high (22%) and the majority of complications related to hospital.

The study of these patients has identified some prognostic factors of the intensive care unit in disease as male gender, duration of administration of Ig, the occurrence of autonomic disorders such unstable blood pressure which remains the most discriminating the occurrence of nosocomial infection.

**Conclusion** Guillain-Barré syndrome therefore a pediatric emergency that requires rapid diagnosis and immediate changes in severity criteria for the establishment of appropriate treatment.

**Competing interests** None.

**References**

1. Crit Care Med. 2003;31(1):278–283.
2. Nobuhiro Y, Hans-Peter H. N Engl J Med. 2012;366:2294–304.

#### P69 Predictors of mortality during purpura fulminans in a developing country

##### Ahmed Hajji^1^, Assaad Louati^2^, Ayari Ahmed^3^, Ammar Khaldi^1^, Aida Borgi^1^, Nargess Ghali^1^, Asma Bouziri^1^, Khaled Menif^1^, Jaballah Najla Ben^1^

###### ^1^Peadiatric intensive care unit, Children Hospital of Tunis, Tunis, Tunisia; ^2^Unité de recherche ur12sp10, université tunis el manar, Children Hospital of Tunis, Peadiatric intensive care unit, Tunis, Tunisia; ^3^Réanimation polyvalente, Hospital Children, Tunis, Tunisia

####### **Correspondence:** Assaad Louati - assaadlouati@gmail.com

*Annals of Intensive Care* 2017, **7(Suppl 1)**:P69

**Introduction** Purpura fulminans (PF) is a major cause of mortality and morbidity in children. Despite therapeutic advances in the management of PF, mortality remains high up rates that exceed 50%. In Tunisia, the profile of such serious disease has not yet been described. The aim of this study was to determine the predictors of mortality in patients admitted in pediatric intensive care unit (PICU) with purpura fulminans.

**Patients and methods** Retrospective review of case sheets was done. Sixty-nine children [median age 3 years (1.6 months to 11 years and boy to girl ratio 1.15) admitted between January 2000 and May 2015 with PF in PICU of the pediatric hospital Bechir Hamza in Tunis, were included. The diagnosis of PF was made in patients with severe sepsis or septic shock with an extensive purpura. The PF is considered secondary to meningococcal infection if *Neisseria meningitidis* or soluble antigens are found at the blood culture or cerebrospinal fluid. In patients whose samples are negative, the PF is considered secondary to meningococcal infection if no other bacterial or viral origin is found to explain the purpura. For each patient enrolled, we have clarified the demographic data, the severity of clinical presentation by pediatric risk score of mortality (PRISM) and Glasgow meningococcal septicemia prognostic score (GMSPS) and therapeutic data.

**Results** A mortality rate of 52% was observed for a predicted death by the PRISM score at 24.6%. Seventy-seven percent of the deaths occurred during the first 24 h. Twenty-five (69.4%) children died of irreversible septic shock and 6 (16.6%) children died of refractory hypoxemiae. Independent predictors of mortality were the initial severity assessed by the GMSPS (p = 0.001) and use of high doses of vasoactive drugs evaluated by the vasoactive-inotropic score (p = 0.026).

**Conclusion** The mortality in our PICU is high, higher than predicted by PRISM. It occurred mainly within 24 h of admission. Early recognition and prompt initial antibiotic therapy continue to be the cornerstones of the successful management of this dramatic disease, reducing mortality.

**Competing interests** None.

#### P70 Epidemiology multiresistant bacteria in pediatric medicosurgical intensive care unit

##### Anwar Armel^1^, Rchi Abdou^1^, Samira Kalouch^2^, Khalid Yaqini^2^, Aziz Chlilek^2^

###### ^1^Anesthésie réanimation, CHU Ibn Rochd, Casablanca, Morocco; ^2^Service de réanimation pédiatrique, Chu Ibn Rochd, Casablanca, Morocco

####### **Correspondence:** Anwar Armel - armelanwar@gmail.com

*Annals of Intensive Care* 2017, **7(Suppl 1)**:P70

**Introduction** Nosocomial infections (NI) to multi-resistant bacteria (BMR) is a main public health problem worldwide. They are particularly frequent and severe in pediatric intensive care.

**Objective** To evaluate the incidence, bacteriological profile, epidemiological characteristics and resistance associated to BMR antibiotics acquired in pediatric intensive care unit.

**Materials and methods** Retrospective study including inpatients and spending more than 48 h in medical-surgical pediatric intensive care unit, at Ibn Rochd University Hospital of Casablanca, over a period of 12 months from 1 January 2015 to 31 December 2015. The BMR taken from different samples (PBDP, central catheter, hemoculture, LCR, urine culture and sampling devices).

**Results** During the study period, we collected 30 episodes of IN to BMR, the incidence rate was 7.1% and the incidence density was 20.6% per 1000 hospitalization days. Two infectious sites were preponderant: pneumonia (53.3%), and central catheter infections (40%). Gram-negative bacilli (BGN) resistant to C3G were most common (about 120 samples: 87%). Acinetobacter baumannii was the main species (60 samples: 43.5%). These BGN resistant to C3G had presented resistance exceeding 50% for imipenem, amikacin, gentamicin, tobramycin, and cotrimoxazole. Resistance to ciprofloxacin was 16.6%. They had isolated 30 strains of Pseudomonas aeruginosa and 24 klebsiella pneumoniae strains resistant to C3G. Six strains of Staphylococcus coagulase negative (SCN) were isolated 2 of which resistant to vancomycin. No strain was resistant to glycopeptides.

**Conclusion** BMR Nosocomial infections are frequent and serious in pediatric intensive care unit. Thus, continuous monitoring of resistance in nosocomial bacteria to antibiotics is a main concern.

**Competing interests** None.

#### P71 An outbreak of *Serratia marcescens* in a mixed neonatal and paediatric intensive care unit: investigation of causes and management

##### Jeanne Brochon^1^, Mihaela Dumitrescu^2^, Sarah Thévenot^2^, Jean-Pascal Saulnier^1^, Khaled Husseini^1^, Catherine Laland^2^, Julie Cremniter^3^, Anne Bousseau^2^, Olivier Castel^2^, Cassandra Brémaud-Csizmadia^1^

###### ^1^Chu de poitiers, Neonatal and paediatric intensive care, Poitiers, France; ^2^Chu de poitiers, Infection prevention team, Poitiers, France; ^3^Chu de poitiers, Laboratory of bacteriology, Poitiers, France

####### **Correspondence:** Cassandra Brémaud-Csizmadia - c.bremaud@chu-poitiers.fr

*Annals of Intensive Care* 2017, **7(Suppl 1)**:P71

**Introduction** *S. marcescens,* a gram negative bacillus, classified as an *Enterobacteriaceae*, was originally considered to be a non-pathogenic saprophytic water organism. Since several years, it is a well-recognised nosocomial pathogen involved in outbreaks in neonatal intensive care units [1].

**Patients and methods** This study describes two consecutive outbreaks of *S. marcescens* clones in a mixed neonatal and paediatric intensive care unit and discusses its investigation and control.

**Results** Between October 2015 and February 2016, seven preterm new-borns (gestational age: 25–34 weeks) were identified with *S. marcescens* in our 15 beds unit, spread over 12 rooms. Four children presented severe infections (four pneumonias, one sepsis and one conjunctivitis). *S. marcescens* was isolated in the stools for three others. From May to June 2016, three new cases (gestational age: 26–34 weeks) were detected (two pneumonias and one lung colonisation). All infected new-borns have favourable clinical outcome.

A single *S. marcescens* strain was identified during each outbreak. The two strains were genetically unrelated and have a natural phenotype of resistance.

In order to contain the outbreak, active surveillance was established to detect infected and/or colonised patients. Strict cohort nursing of those patients was implemented and admissions in the unit were limited.

Health care workers of the unit were requested to reinforce infection control measures. Audit programs performed by the infection prevention team showed the necessity to reinforce meticulous hand hygiene using hydro alcoholic solution and adequate gloves wearing.

Sixty environmental samples (water, room surfaces, medical devices…) failed to identify *S. marcescens* reservoirs.

Actually, the current strategy of empiric antibiotic treatment is being reevaluated by a multidisciplinary group composed of a medical microbiologist, an infectious disease specialist and a paediatric intensive care clinician. Furthermore, a case–control study is being performed to assess the risk factors for acquiring *S. marcescens* in a mixed neonatal and paediatric intensive care unit.

The cause analysis also highlighted that:

- due to the mixed character of our ward, patients frequently changed rooms during their hospitalisation in order to increase the admission capacity. However, this policy may increase infectious risks.
- the first outbreak was detected late when three infants with *S. marcescens* were identified. During the second event, infection control measures were applied after detection of two cases. This early intervention probably limited the spread. No new case was detected since June 2016.

**Conclusion** *S. marcescens* can cause rapidly-spreading outbreaks of severe infections in neonatal units, but with appropriate control measures these outbreaks can be contained at an early stage.

**Competing interests** None.

**Reference**

1. Voelz A. Outbreaks of *Serratia marcescens* in neonatal and pediatric intensive care units: clinical aspects, risk factors and management. Int J Hyg Environ Health. 2010;213:79–87.

#### P72 Adjustment of antimicrobial treatment in a paediatric intensive care unit

##### Margot Diss^1^, Aurélie Portefaix^2^, Julien Berthiller^3^, Etienne Javouhey^4^, Yves Gillet^2^

###### ^1^PEDIATRIE, Hôpital Femme Mère Enfant, Bron, France; ^2^Réanimation Pédiatrique, Groupement Hospitalier Est-Hôpital Femme Mère Enfant, Bron, France; ^3^Pôle information médicale evaluation recherche, equipe d’accueil 4129, hospices civils de Lyon, lyon, France; ^4^Réanimation pédiatrique hfme, Hospices civils de Lyon, Lyon, France

####### **Correspondence:** Margot Diss - margot.diss@gmail.com

*Annals of Intensive Care* 2017, **7(Suppl 1)**:P72

**Introduction** In order to limit the emergence of antimicrobial resistance, it is recommended to re-evaluate every prescription of antimicrobial treatment within 72 h and to proceed to a de-escalation whenever it is possible [1]. However, evaluations of such recommendations are scarce, especially in paediatrics. Our goal was to evaluate the proportion of modification of the antimicrobial treatment within the first 72 h in our PICU and to determinate whether it was relevant or not.

**Materials and methods** We conducted a retrospective study during 6 months in the paediatric intensive care unit at the Hôpital Femme Mere Enfant in Lyon, France. Every patient with curative antimicrobial treatment initiated in the unit or within 48 h before admission was included. Evaluation and adjustment—if any- of antimicrobial treatment before 72 h, according to bacteriological results and clinical evolution, were recorded. A paediatric infectious disease specialist reviewed the charts to determine for each patient if the modification or the remaining of initial treatment were relevant or not. An alternative choice was suggested in case of irrelevance.

**Results** We included 168 antimicrobial treatments. 87.4% were adjusted within 72 h after initiation. 77.3% of adjustments were considered as relevant. Among those that had not been adjusted, de-escalation would have been possible in 92.9% of cases. Among all those which should have needed an adjustment, 26.1% had an insufficient or non-realised de-escalation. Factors associated with absence of or irrelevant adaptation were nosocomial infection and absence of consultation of a paediatric infectious disease specialist about antibiotic modification.

**Conclusion** Antimicrobial treatment adjustments are globally well executed in our paediatric intensive care unit, but could be improved by obtaining expertise from an infectious diseases specialist more frequently. Hence antibiotic de-escalation should be improved. The results of this study constitute an adequate basis for a future antimicrobial stewardship that could be conducted in our unit while focusing on a systematic consultation with a paediatric infectious disease specialist.

**Competing interests** None.

**Reference**

1. Bretonnière C, Leone M, Milési C, et al. Strategies to reduce curative antibiotic therapy in intensive care units (adult and paediatric). Intensive Care Med. 2015;41(7):1181–96.

#### P73 The use of antibiotics in pediatric critical care in Oran, Algeria

##### Nabil Tabet Aoul^1^, Ali Douah^2^, Zakaria Addou^3^, Houari Youbi^2^, Mohamed Moussati^2^, Kamel Belhabiche^2^, Souad Mir^1^, Sanaa Abada^1^, Zerhouni Amel^4^, Nabil Aouffen^2^

###### ^1^Réanimation pédiatrique canastel, Faculté de médecine d’Oran, Oran, Algeria; ^2^Anesthésie réanimation pédiatrique, Etablissement hospitalier spécialisé en pédiatrie Canastel, Oran, Algeria; ^3^Réanimation pédiatrique de Canastel d’oran, Departement de medecine d’Oran Algerie, Oran, Algeria; ^4^Réanimation pédiatrique, EHS CANASTEL, Oran, Algeria

####### **Correspondence:** Nabil Tabet Aoul - tabetrea@yahoo.fr

*Annals of Intensive Care* 2017, **7(Suppl 1)**:P73

**Introduction** Development of an infection in a critically ill patient is a gravity element associated with increased mortality. Elements related to the patient and his environment used to target the empiric antibiotic therapy to bacterial flora community or nosocomial kind. The choice of therapy is guided by direct examination of bacteriological samples.

Our objective is to identify the use of antibiotics variations in pediatric intensive care unit in the pediatric hospital of Canastel, in Oran, Algeria.

**Patients and methods** This is a retrospective study during the period 1 January 2016 to 30 June 2016, including all patients hospitalized in pediatric critical care (10 beds) of Canastel, in Oran Algeria. We studied the age, gender, reason for hospitalization, various antibiotics used, length of stay and mode of discharge of patients.

**Results** Two hundred and sixteen patients were hospitalized during the first 6 months of 2016. The average age is 3 years (1 month to 15 years), sex ration is 1.2. The most common reason for admission was respiratory distress 27.2%, cardiopulmonary arrest in 10.7% and status epilepticus in 10.1%. The most commonly used antibiotic is cefotaxime 75% of cases, followed by Vancomycin 28.5%, Mitronidazole 22.5%, Gentamycine 17.5%, Ceftazidime 8.8%, Imipinem 7.4%. About 216 patients, 27% received at least 3 antibiotics during their hospitalization, 29.5% received only one antibiotic and 16.5% none. The average hospital stay was 6 days (±12 days).

**Conclusion** Our study shows a heterogeneous use of antibiotics, from where interest of a prescription adapted to prevent the emergence of resistant bacteria. This problem could be better controlled by optimizing antibiotic prescriptions also taking into account the economic consequences.

**Competing interests** None.

#### P74 Does apnea affects the reliability of Analgesia Nociception Index (ANI), as a monitor of nociception?

Zina Bouzit^1^, Ahmed H. Grati^1^, Gilles F. Dhonneur^1^

##### ^1^Service d’Anesthésie et des Réanimations Chirurgicales, Hôpital Henri Mondor, Créteil, France

###### **Correspondence:** Zina Bouzit - zina.bouzit@gmail.com

####### *Annals of Intensive Care* 2017, **7(Suppl 1)**:P74

**Introduction** Continuous assessment of nociception intensity during anesthesia is crucial. Analgesia nociception index (ANI: 0–100) has been proposed to clinicians to objectively measure pain in both conscious and anesthetized patient. ANI calculation results from sophisticated computing of sympathetic to parasympathetic tones balance using heart and respiration rate variability. Because we aimed to evaluate nociception during oro-tracheal intubation performed under apnea conditions, we conducted a study to test the reliability of ANI at monitoring nociception when respiratory rate was zero.

**Materials and methods** ASA physical status I healthy informed and consenting adult volunteers participated in this experimental prospective trial. Demographic parameters were recorded and non-invasive hemodynamics (HD), blood pressure (BP), heart rate (HR), pulsed oxygen saturation (SpO_2_) and ANI monitoring tools were installed. After a 5 min resting and stabilization the volunteers lying on their back were asked to perform a 30 s duration apnea starting on command at Residual Functional Capacity (RCF). After a wash-out period following this first apnea period (P1) and return to baseline parameters, the volunteers were asked again to perform a second 30 s duration apnea (P2) starting on command at RCF. Initiation of P2 coincided with 30 s ulnar nerve stimulation at the wrist (single twitch, 1 Hz, 25 mA). The different component of ANI (energy, mean, and instantaneous values: iANI), HR, BP and SpO_2_ values were recorded at predefined time points: T0 (baseline values, just before apnea), T1 (end of apnea), T2 (T1 + 30 s), for P1, and the same predefined time points (T0′, T1′, T2′) for P2. After the experiment, volunteers were asked to rate the pain intensity at the wrist during P2, using a Visual Analogue Scale (VAS: 0–100). Evolutions of measured parameters were compared between P1 and P2. Values are mean ± SD.

**Results** 21 healthy volunteers aged 34 ± 10 were included. During P1 and P2, both iANI and energy were monitored. Mean VAS during P2 was 27/100. Figure 23 illustrates HD and iANI variations at T1/T1′ and T2/T2′. During P1, there is a remarkable stability of iANI at T1 (+1%) and no significant decrease at T2 (−3%). On the opposite, a progressive decrease of iANI was evidenced during P2 (−5% at T1′), reaching a nadir 30 s after the end of nociceptive stimulation (−17% at T2′). HD and Sp02 remained stable and comparable in P1 and P2.

**Fig. 23:**
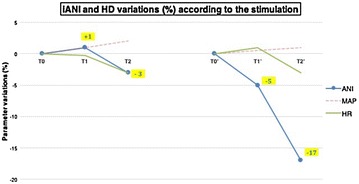
iANI and HD variations (%) according to the stimulation

**Conclusion** Based upon our results, ANI seems discriminant and efficient at measuring nociception in the conditions of a short apnea of 30 s. ANI is thus probably a reliable tool to measure nociception during tracheal intubation. Our trial shows that HD is less sensitive than ANI to detect weak intensity nociceptive stimulus.

**Competing interests** None.

**Reference**

1. Feng Y. Eur J Anaesthesiol. 2015;32(1):58–9.

#### P75 Early mobilization in a medical ICU: a first Tunisian experience

##### Nesrine Sma^1^, Ahmed Khedher^1^, Khaoula Meddeb^1^, Jihene Ayachi^1^, Nesrine Fraj^1^, Hend Ben Lakhal^1^, Messaouda Khelfa^1^, Hedia Hammed^1^, Hajer Hafsa^1^, Raja Boukadida^1^, Imed Chouchene^1^, Mohamed Boussarsar^2^

###### ^1^Réanimation médicale, CHU Farhat Hached, Sousse, Tunisia; ^2^Réanimation médicale, CHU Farhat Hached, Research Laboratory N° LR14ES05, Faculty of Medicine, Sousse, Tunisia

####### **Correspondence:** Mohamed Boussarsar - hamadi.boussarsar@gmail.com

*Annals of Intensive Care* 2017, **7(Suppl 1)**:P75

**Introduction** Critically ill patients are known to be complicated and their mobilization on the earliest days of ICU stay has shown to be feasible, well tolerated and beneficial. We aimed to assess the feasibility and tolerance of early mobilization as part of routine care in a Tunisian medical ICU.

**Patients and methods** A prospective period study conducted in a 7-bed-medical ICU with nurse/patient ratio at 1/3 over 12 months. Were studied, incidence density of four predefined activity events (sit on bed, sit in chair, stand-up and ambulate), onset delay, limitation factors and tolerance. Six activity-related adverse events were defined as fall to knees, catheters or tubes removal, systolic pressure drops more than 30 mmHg, pulse increase by 20 beats/mn or bradycardia, SpO_2_ drop <80% and extubation.

**Results** During the study period, we conducted a total of 1265 activity events in 127 patients. The mean length of stay was 11.8 ± 12.3 days and the global incidence density was 842 activities/1000 patient days. The mean age was 56.4 years ± 19.9. 44(34) patients had a BMI > 30 kg/m^2^. Mean SAPS II score was 29.9 ± 12.9 years. At ICU admission 54(42.5) patients were on invasive mechanical ventilation, 38(29.9) patients were on NIV and 27(21.3) patients had vasopressors. Onset delay to the first activity was 5.8 ± 3.8 days for patients on mechanical ventilation. 21(16.5) patients had vasopressors when they did their first activity and the onset delay for those patients was 5.6 ± 3.5 days. The first activities consisted only in “sit on bed”, 80.3% and “sit in chair”, 19.7%. Neither standing nor ambulation was done as a first activity. The activity events included 306(24.2) sit on bed, 629(49.8) sit in chair, 182(14.3) standing position and 148(11.7%) ambulation. Incidence density of activities was 604 activities/1000 patient days for patients with invasive mechanical ventilation, 1233 for those with NIV and 1004 for patients without mechanical ventilation. In patients on ambulation 6 had endotracheal tube, 19 had tracheostomy and 27 had NIV. Twenty-eight incidents occurred (2.1%). They consist in hypotension (32.1%), tachycardia (46.4%), one fall to knees, 3 feed tube removals, one urinary tube removal and only one central catheter removal was registered without harmful consequences. No accidental extubation occurred. No barrier to critically ill patient was found.

**Discussion** Our study proves that early mobilization is feasible and safe for all patients including those with mechanical ventilation. Our results are in line with literature [1]. Sricharoenchai in an observational study conducted in Baltimore ICU over 30-month period including 5267 activities showed a low incidence of early mobilization adverse effects (0.6%) [2].

**Conclusion** This study shows the feasibility and safety of an uncommon practice in a relatively poor resources and low patient-nurse ratio medical ICU.

**Competing interests** None.

**References**

1. Guérin C, Burle J-F. Early rehabilitation in ICU is possible. Réanimation. 2015;24:S371–8. doi:10.1007/s13546-014-1005-7.
2. Sricharoenchai T, Parker AM, Zanni JM, Nelliot A, Dinglas VD, Needham DM. Safety of physical therapy interventions in critically ill patients: a single center prospective evaluation of 1110 ICU admissions. J Crit Care. 2013. doi:10.1016/j.jcrc.2013.12.012.

#### P77 Analysis of interventions by ICU team outside the ICU: 11 years monocentric prospective study

##### Nicolas Lau^1^, Ilham Mezhari^1^, Nicolas Roucaud^1^, Matthieu Le Meur^1^, Rémi Paulet^1^, Jean-Michel Coudray^1^, Martial Thyrault^1^

###### ^1^Icu, C.H. des Deux Vallées - site Longjumeau, Longjumeau, France

####### **Correspondence:** Nicolas Lau - docteurlau@gmail.com

*Annals of Intensive Care* 2017, **7(Suppl 1)**:P77

**Introduction** Traditionally, in French Hospitals, the team composition is different according of the moment: the daily team for day from Monday to Friday, the watch team for night, week end, and public holiday. It appears to be important for us to evaluate the workload in the different configurations. We need to know the way our ICU team is needed outside the ICU in order to improve the quality of the team, in order to anticipate the workload during different moments and in order to avoid reducing the team size.

**Materials and methods** It is a prospective monocentric study inside a hospital of 450 beds with an ICU of 12 beds.

The ICU team is the rescue team for every patient inside the hospital. It is also the referent for antibiotherapy, emergency advice, withholding treatment consultant.

From January 1st 2005 to December 31th 2015, every intervention of the ICU team outside the ICU is recorded with day, hour, time spent, ground of appeal, outcome of the patient.

So we define 2 situations for the ICU team:

Team configuration A: the team is composed of more than one senior and more than one junior (from Monday to Friday, 9.00 am to 18.29 pm)

Team configuration B: the team is composed only of one senior and one junior. (From 18.30 pm to 8.59 am, all Saturdays, all Sundays and all public holidays)

Considering that the average time in these 11 years of each configuration are Team configuration A: 27%, Team configuration B: 73%.

We compare the percentage of all interventions using the time passed in the interventions and the numbers of interventions.

**Results** The ground of appeal of interventions was a rescue request in 8.7%. Interventions consisted of decision of withholding treatment for in 16.3%. These interventions ended with a transfer to ICU in 30.6% as direct outcome.

Inside these transfers, 64.5% were for the Hospital ICU, 35.5% for ICU of other hospital including for Neuro surgical center, immediate Interventional radiography and Neuro vascular ICU.

See Table 17.

**Table 17:** Time data

Time data	Team Config A	Team Config B
Number of interventions	2342	4287
Percentage of interventions	35.3	64.7
Total duration of all interventions (h)	1088	2575
Percentage of duration of all interventions	29.7	70.3
Average time of intervention (min)	28	36
Maximal duration (min)	285	390

**Conclusion** Clinical activity outside the ICU is not reduced when the ICU team is the smallest, it respects the time ratio of 27/73. More time is needed outside the ICU. The smallest team has to deal with a huge workload and also makes important decision. Do we have to rethink our general organization?

**Competing interests** None.

#### P78 Who are these consultants to medical emergencies in a university hospital?

##### Wahiba Imène Ghomari^1^, Reda Boumlik^2^

###### ^1^Anesthésie-Réanimatiion, CHU Abdelkader Hassani, Sidi Bel Abbès, Algeria; ^2^Urgences médico-chirurgicales, CHU Abdelkader Hassani, Sidi Bel Abbès, Algeria

####### **Correspondence:** Wahiba Imène Ghomari - ghomari_wahiba@yahoo.fr

*Annals of Intensive Care* 2017, **7(Suppl 1)**:P78

**Introduction** Besides the “real” urgent medical cases, emergency department hire people who took by themselves the decision to come, as well as those addressed by other doctors. The purpose of this study was to determine the profile of patients who arrived at the emergency for a general medical consultation.

**Patients and methods** This study carried out in an emergency department of a university hospital included all patients over 15 years, having consulted with medical emergencies over a week. The data collected were epidemiological and socio-economic, the reason for the consultation and the future of consultants. The consultations were deemed appropriate or not by the doctor on duty depending on the nature of the disease, day and time of the consultation and the age of the symptoms.

**Results** We collected 469 consultants. The average age was 43.45 years (16–104 years) for a sex ratio of 0.87. The main reasons were an oropharyngeal infection or bronchopulmonary (17.3%), asthenia (13.4%), chest pain (11.9%). More than 50% of the consultations were deemed inappropriate. These patients were younger than 35 years in 48.4% of cases, and jobless in 58.6% of cases. There was no significant difference in the number of inappropriate consultations between the two genders. The days and times of arrival did not differ between appropriate consultation and not appropriate. After review, 6% of patients were admitted to unit short hospitalization, 49.9% referred to a specialist consultation, other consultants put outgoing, equipped (39.7%) or not (4.5%) of a therapeutic prescription.CV

**Discussion** The use of emergency would be linked to the absence of prior appointment, and continuity of care. The consultants have the option of availability, some for conditions not covered by the emergency. The high proportion of younger people without profession is explained by the free care. The elderly rarely autonomous and complex diseases carriers, are fewer in the “inadequate consultation” group.

**Conclusion** Develop a care system and quality of care in basic health facilities, educate and convince patients to get there, would relieve hospital emergency services that will then take care of true emergencies.

**Competing interests** None.

**References**

1. Gentile S, Durand AC, Bongiovanni I, Rofritsch S. Les consultants des services d’urgence relevant de la médecine générale: analyse de nouveaux comportements de santé. JEUR. 2007;20(1S):138.
2. Berraho M. Les consultations non appropriées aux services des urgences: étude dans un hôpital provincial au Maroc. Prat Organ Soins. 2012; 3(43):197–204.

#### P79 Impact of the evolution of the National Health Insurance tariffs on the income of a French ICU

##### Vincent Peigne^1^, Jean-Louis Daban^2^, Mathieu Boutonnet^2^, Bernard Lenoir^3^

###### ^1^Réanimation, Centre hospitalier Métropole Savoie, Chambéry, France; ^2^Réanimation, Hôpital d’Instruction des Armées Percy, Clamart, France; ^3^Département d’anesthésie-réanimation, Hôpital d’Instruction des Armées Percy, Clamart, France

####### **Correspondence:** Vincent Peigne - vincentpeigne@yahoo.fr

*Annals of Intensive Care* 2017, **7(Suppl 1)**:P79

**Introduction** Hospital funding for ICU stays in France is made of reimbursement of a fixed amount according the diagnosis-related group (DRG) of the patients and of extra funding for each day spent in ICU if the patient fulfilled criteria of severity (SAPS2 > 15) and of treatment intensity (use of organ support). The tariffs of reimbursement for the different DRGs and for the extra funding are updated every year. We measured the impact of these updates on the income of our ICU.

**Patients and methods** DRG and length of stay of all the patients hospitalized during 2011 in our 12-bed ICU were extracted from the administrative database of our institution. We computed the reimbursement for the care of these patients with the tariffs of 2011, 2012, 2013, 2014, 2015 and 2016 (data from the Agence Technique pour l’Information Hospitalière). Inflation was taken into account according to the recommendations of the Institut national de la statistique et des études économiques.

**Results** 592 ICU stays (3224 days) have been analyzed. The patients were classified in 237 DRG. The income of the ICU decreased from 8,416,260, 14€ in 2011 to 7,816,786, 72€ (−7%).

Income decreased every year, with the most important lessenings during the first years (2012: −2.37%; 2013: −2.47%; 2014: −0.81%; 2015: −0.82%; 2016: −0.84%)

This reduction was explained by both a lowering of the tariffs of the different DRG (mean evolution −4.6%) and a diminution of the extra funding (814,32 € per day in 2011, 801,19 € in 2016, −1.6%).

**Discussion** These results are based on a small number of ICU stays but are probably representative of the global trends of ICU reimbursement because of the high number of DRG analyzed.

**Conclusion** This simulation gives an estimate of the economical pressure sustained by the French ICUs during the last 6 years. Productivity gains are necessary to cope the tariff evolution and may require reduction of costs, increase of activity and pricing optimization.

**Competing interests** None.

#### P80 Risk factors and prognosis of C sections performed in emergency for placenta previa bleeding

##### Hafiani Yassine^1^, Cheikh Chaigar Mohamed^1^, Allali Khalid^1^, Moussaid Ihssan^1^, Elyoussoufi Said^1^, Salmi Said^1^

###### ^1^Anesthesie Reanimation, CHU Ibn Rochd Casa, Casablanca, Morocco

####### **Correspondence:** Hafiani Yassine - docteurhafianiyassine@live.fr

*Annals of Intensive Care* 2017, **7(Suppl 1)**:P80

**Introduction** Hemorrhagic complications during placenta previa are unpredictable and may indicate an extremely urgent C section. Our study goal is to determine the risk factors and prognosis of this urgent situation.

**Patients and methods** This is a case–control study, prospective and analytic over the second semester of 2015. We included all patients who underwent emergency C section for placenta previa bleeding. The witness cases are represented by C section performed in emergency for another reason, at random.

**Results** We identified 42 cases for 126 witnesses. The over 30 years patients accounted for 45.23% in the case group against 22.2% in the control group (p = 0.03). The average rate for cases was 3–2 against the controls (p = 0.001). For instance, the admission was referred in 76.19% in the case group against 80.95% for controls. At least an ultrasound was performed during pregnancy in 23.8% of case group against 32.5% for control. Maternal complications in the case group are represented by a maternal anemia in 85.71% against 15.07% for controls (p = 0.002), fetal complications due to hypotrophy represent 19.04% in the case group against 11.1% for controls (p = 0.04).the perinatal mortality was 26.19% in the case group against 9.5% for controls (p = 0.03).

**Conclusion** The placenta previa is still a serious disease of pregnancy. Early ultrasound diagnosis would enable appropriate monitoring and prevention of all obstetric complications of this disease.

**Competing interests** None.

#### P81 Unusual vein thrombosis

##### Amira Ben Jazia^1^, Jaziri Fatima^2^, Skouri Wafa^2^, Bennasr Maha^2^, Ben Abdelghni Khaoula^2^, Turki Sami^2^, B Abdallah Taeib^2^

###### ^1^Medical icu, Hospital Abderrahmen Mami De Pneumo-Phtisiologie, Ariana, Tunisia; ^2^Réanimation médicale, Hôpital Abderrahmen Mami, Ariana, Tunisia

####### **Correspondence:** Amira Ben Jazia - amira26juillet@yahoo.fr

*Annals of Intensive Care* 2017, **7(Suppl 1)**:P81

**Introduction** Unusual venous thrombosis are those whose site is other than the lower limbs. They are quite rare. Clinical expressions depend on their locations (digestive, brain or upper limbs).

Their diagnosis and treatment are much less codified than the lower limbs locations. Their causes should be carefully sought for better management.

**Materials and methods** Retrospective and descriptive study of patient records who experienced deep vein thrombosis and were followed in the service during the period between 1985 and 2015. Our work aims to classify these atypical localizations in our patients, and to study the epidemiology, etiology, treatment, and finally their evolution

**Results** We collected 120 cases of deep vein thrombosis, of which 25 cases are unusual location, it is 6 inferior vena cava thrombosis, 1 superior vena cava thrombosis, 8 thrombosis of renal vein, one thrombosis of hepatic vein, 4 cerebral localizations.

4 locations at the Superior Members, 1 thrombosis of the internal jugular vein and 2 of the subclavian vein and 2 of the axillary veins.

One thrombosis of the central vein of the retina and one of thrombosis renal graft vein. There were 17 women and 8 men whose average age was 43.4 years.

The diagnostic tool was Doppler ultrasound in 60% of cases, CT or magnetic resonance imaging in 40% of cases.

The etiologic survey found that 7 patients had glomerular nephropathy with an intense nephrotic syndrome, 1 patient had lupus, 4 patients with Behçet’s disease, 4 patients had neoplasia, 1 patient had protein C and S deficiency, 1 patient had hyperhomocysteinemia and no etiology was found in 1 case.

All patients were put on anticoagulants associated with etiological treatment. The outcome was favorable in 13 patients, 2 patients had pulmonary embolism, 2 patients died, in 3 patients thrombosis recurred and 5 patients were lost to view.

**Conclusion** In the contrary to the literature, the neoplasti origin of unusual thrombosis does not prevail in our series where the causes are dominated vasculitis and renal disease which is explained by the selection bias of patients.

**Competing interests** None.

**Reference**

1. Rev Internal Med.

#### P82 Pain therapy in the emergency: satisfaction survey

##### Fatma Kaaniche Medhioub^1^, Rania Allela^2^, Najla Ben Algia^3^

###### ^1^Faculté de médecine de Sfax, Sfax, Tunisia; ^2^Hopital régional mahres, Faculté de médecine de Sfax, Sfax, Tunisia; ^3^Intensive care, hopital régional Gafsa, Sfax, Tunisia

####### **Correspondence:** Fatma Kaaniche Medhioub - fatma_kaaniche@yahoo.fr

*Annals of Intensive Care* 2017, **7(Suppl 1)**:P82

**Introduction** The management of pain is an emergency medicine’s priority. It is a criterion of quality care. We conducted a survey that assesses the degree of patient satisfaction overlooked the pain therapy.

**Patients and methods** Prospective observational study performed in the emergency department, which included all patients aged over 10 years consulting for acute pain requiring the use of an analgesic treatment. The intensity of pain was measured by visual analogue scale (VAS). Patients unable to assess pain by VAS or having a vital distress are excluded from this study

**Results** Over a period of 3 months, we included 183 patients. The average age was 41 ± 16 years. The sex ratio was 1.7. The mechanism of pain was traumatic in 25 patients (13.7%). VAS means at the entry was 5.8 ± 1.6. At the exit, it was 6.4 ± 2.3. We used the painkillers levels 1 in 145 patients (79.2%), levels 2 in 27 patients (14.8%) and levels 3 in 11 patients (6%). Only 6% of patients are not satisfied, 38.8% are dissatisfied, while 44.8% are satisfied or very satisfied (10.4%).

**Conclusion** This study shows that nearly 50% of patients are few or dissatisfied with the management of pain, efforts are still needed to improve.

**Competing interests** None.

#### P83 Transcranial Doppler in pediatric intensive care units for 152 children without traumatic brain injury

##### Virginie Rollet-Cohen^1^, Philippe Sachs^2^, Pierre-Louis Leger^3^, Zied Merchaoui^4^, Sylvain Renolleau^5^, Mehdi Oualha^6^

###### ^1^Réanimation et surveillance continue pédiatriques, Hôpital Necker, Rue de Sèvres, Paris, France; ^2^Réanimation et surveillance continue pédiatriques, CHU Robert Debré, Paris, France; ^3^Réanimation néonatale et pédiatrique, Hopital pour enfants Trousseau, Paris, France; ^4^Réanimation néonatale et pédiatrique, CHU Kremlin-bicêtre, Le Kremlin-Bicêtre, France; ^5^Réanimation pédiatrique polyvalente, Hôpital Necker - Enfants Malades, Paris, France; ^6^Réanimation et surveillance continue médico-chirurgicales, Hôpital Necker - Enfants Malades, Paris, France

####### **Correspondence:** Virginie Rollet-Cohen - virginierollet11@gmail.com

*Annals of Intensive Care* 2017, **7(Suppl 1)**:P83

**Introduction** Transcranial Doppler (TCD) is currently used at the bedside of critically ill children and can detect cerebral blood flow modifications. Use and impact of TCD in non-traumatic critically ill children is unknown. We aimed to describe and to assess the TCD and related impact in this population.

**Patients and methods** This French prospective and multicentric study included all children (0–18 years) who had TCD in four pediatric intensive care units during 1 year. A questionnaire was completed for each performed TCD by the intensivist. TCD interpretation and impact were accorded to the intensivist. Factors associated with impact, and especially diagnostic and therapeutic impacts were identified.

**Results** 152 patients were included with a median age of 7.6 months (0–206 months). TCD was performed in 6.7% of all admitted patients during the inclusion period. 61% of patients were aged between 28 days and 2 years. 67% of patients were admitted for a neurological motive. Diagnosis were: neurological (70%), hemodynamic (10%) respiratory (10%), hepatic (7%) and renal (1%), others (2%). TCD was performed for monitoring of neurological disorders in 56% of cases. Intracranial hypertension was the most frequently suspected abnormality, and most often searched by young operators (p < 0.001). Hyperperfusion was most often suspected by experienced intensivists (p < 0.05). Discrepancy between the interpretation of DTC values and published reference values was found in 116 patients (76%). TCD had an impact for 116 patients (76%), with a strong impact (diagnosis’ confirmation or exclusion, therapeutic impact) for 98 patients (64%). Confirmation of diagnosis was statistically associated with patients severity: mortality, catecholamines, intubation, PELOD score (p < 0.05). Hemodynamic dysfunction (OR 6.4; p = 0.007) and less experienced operators (OR 3.9; p = 0.012) were independent factors affecting therapeutic impact in multivariate analysis.

**Discussion** This original study is at our best of knowledge the first to describe use of TCD in heterogeneous diseases in critically ill children without traumatic brain injury. TCD was performed not only in patients with neurological disorders, highlighting the wide field of applications of this exam. This study showed the difficulty of TCD interpretation, whose reference values are not well-known by operators. Moreover, TCD values could be biased by several systemic parameters, usually not controlled in these unstable patients. TCD’s impact was highly retained, and depending on severity of patients and on intensivist’s experience.

**Conclusion** TCD is performed in heterogeneous diseases in non-traumatic critically ill children. TCD has an important impact, dependent on the operator’s experience and on patient’s severity. Regarding the difficulty of accurate interpretation of TCD’s values, improvement of TCD’s use is necessary.

**Competing interests** None.

#### P84 Recruitment of the sublingual microcirculation in children with extracorporeal membrane oxygenation

##### Maxime Eloi^1^, Jérôme Rambaud^2^, Sandrine Jean^2^, Maryne Demoulin^2^, Cécile Valentin^2^, Julia Guilbert^2^, Isabelle Guellec^2^, Hervé Walti^2^, Ricardo Carbajal^2^, Pierre-Louis Leger^2^

###### ^1^Unité u1141, INSERM-Hôpital Robert Debré, Paris, France; ^2^Réanimation néonatale et pédiatrique, Hopital pour enfants Trousseau, Paris, France

####### **Correspondence:** Pierre-Louis Leger - leger.pierrelouis@gmail.com

*Annals of Intensive Care* 2017, **7(Suppl 1)**:P84

**Introduction** The sublingual microcirculation is impaired in states of severe sepsis and septic shocks. The microcirculatory dysfunctions are prognostic markers of survival and organ failures in critically ill adult patients. In children, the sublingual microcirculation is also impaired in septic shock [1] and severe respiratory failures [2]. We hypothesized that the microcirculation impairments related to the severity of respiratory and circulatory failures could be reversible under extracorporeal membrane oxygenation (ECMO). The aims of the study are (1) assess the feasibility of the sublingual microcirculation monitoring in pediatric ECMO, (2) compare the microcirculation parameters under ECMO between J1 and J3.

**Patients and methods** Prospective observational study in the pediatric intensive care unit at Trousseau hospital, Paris. The videos have been acquired with Microscan^®^ device (Microvision, Amsterdam) in sublingual areas. The data analyses have been performed by the AVA3 software. The statistical analyses have been performed by PRISM 5 software.

**Results** We have included 10 children on ECMO. The age and weight were respectively 5 ± 14.8 months [0;47] and 4.7 ± 4 kg [2.5;16]. The sexe ratio (M/F) was 3/7. The ECMO indications were refractory acute respiratory distress syndromes (4), septic shocks (3), cardiac arrest (1), diaphragmatic hernias (3), refractory pulmonary hypertension (1). During the pre-ECMO period 9 children have been treated with Nitric Oxide for pulmonary arterial hypertension and 9 children needed catecholamine support. During ECMO 8 children have been treated with noradrenaline, 3 with adrenaline, 6 with dobutamine. The mean catecholamines duration was 6.5 ± 8.2 days [0; 25]. Five children received a arterio-venous ECMO, 2 veno-venous ECMO, and 3 multimodal ECMO (A-V and V-V). The mean ECMO duration was 9.4 ± 3.3 days [2; 13] and hospitalization duration was 29.6 ± 20 days [9;64]. Concerning the microcirculation parameters the microvascular flow index (MFI) was significantly improved in the small microvessels (2.1 ± 0.36 [1.3; 2.5] at day 1 and 2.5 ± 0.27 [2.2; 3] at day 3; p = 0.0173) and in the medium microvessels (2.6 ± 0.24 [2.2; 2.9] at day 1 and 2.8 ± 0.2 [2.5; 3] at day 3; p = 0.045). Regarding the biological markers the pH increased between day 1 and day 3 (7.3 ± 0.12 [7.1, 7.5] vs 7.4 ± 0.11 [7.3, 7, 6]; p = 0.0064). See Fig. 24.

**Fig. 24:**
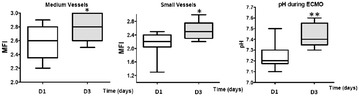
Time course of the sublingual microcirculation and pH during ECMO. *MFI* The microvascular flow index evaluates the micro vascular perfusion. *p < 0.05; **p < 0.005

**Discussion** In this preliminary study the children had severe hemodynamic failures or severe respiratory failures with pulmonary hypertension which needed of catecholamines and ECMO support. The alteration of the sublingual microcirculation was confirmed at day 1 under ECMO. The microvascular perfusion parameters were reduced compared to the normal value of healthy subjects (MFI = 3). The partial MFI restoration confirmed the microcirculation recruitment in these patients. Now, further studies are needed to confirm this microcirculation’s recruitment in a larger pediatric ECMO cohort and to investigate the impact of this recruitment on the prognosis.

**Conclusion** The microcirculation was impaired in the initial phase of ECMO in children with severe respiratory or circulatory failures and it was partially restored during the early phase of ECMO.

**Competing interests** None.

**References**

1. Top AP, Ince C, de Meij N, van Dijk M, Tibboel D. Persistent low microcirculatory vessel density in nonsurvivors of sepsis in pediatric intensive care. Crit Care Med. 2011;39(1):8–13.
2. Top AP, Ince C, Schouwenberg PH, Tibboel D. Inhaled nitric oxide improves systemic microcirculation in infants with hypoxemic respiratory failure. Pediatr Crit Care Med. 2011;12(6):e271–4.

#### P85 Impedance cardiography by PhysioFlow^®^ for non-invasive cardiac output monitoring: a comparison with trans-thoracic echocardiography in pediatric intensive care patients

##### Yasemin Karaca-Altintas^1^, Astrid Botte^1^, Julien Labreuche^2^, Elodie Drumez^2^, David Brossier^1^, Patrick Devos^2^, Franck Bour^3^, Francis Leclerc^1^, Stéphane Leteurtre^1^

###### ^1^Pediatric intensive care unit, CHU Lille, F-59000 Lille, France; ^2^Department of biostatistics, CHU Lille, F-59000 Lille, France; ^3^Physioflow, Manatec Biomedical, F-78300 Poissy, France

####### **Correspondence:** Yasemin Karaca-Altintas - millistate@gmail.com

*Annals of Intensive Care* 2017, **7(Suppl 1)**:P85

**Introduction** Impedance cardiography (IC) is a promising non-invasive, continuous cardiac output (CO) monitoring method. PhysioFlow^®^ (PF^®^) is a new IC technique never studied in pediatric intensive care unit (PICU). The aim of the study was to compare CO and cardiac index (CI) measurements in PICU patients by IC using PF^®^ with those obtained by the method of reference, trans-thoracic Doppler echocardiography (TTE), with regard to accuracy, precision of agreement and reproducibility.

**Patients and methods** In this single-center prospective method comparison study, all PICU patients aged between 28 days and 10-year-old and requiring TTE were included, except those with complex congenital heart disease or poor IC signal and/or TTE quality. Simultaneous sets of three measurements were realized by TTE and PF^®^. CO and CI measured by TTE (CO*TTE* and CI*TTE*) were compared with CO and CI obtained by PF^®^ (CO*IC* and CI*IC*). Concordance correlation coefficient (CCC) and Bland–Altman analysis were used to analyze the concordance rate and compare accuracy and percentage error (PE) between the two methods. As required, data were logarithmically transformed prior to Bland–Altman analysis. Reproducibility was evaluated with intraclass correlation coefficient (ICC) and using the calculation of the precision of the method [1]. Post-hoc signal analysis was performed to evaluate the quality of IC signal.

**Results** A total of 43 patients (median age: 13 months, interquartile range (IQR) 4–34 months) were included. Median PIM2 probability of death was 1.04 (IQR 0.34–5.11), 9.3% of patients had inotropic support and 30.2% had a mechanic ventilation. On 129 paired measurements, mean CO*TTE* was 1.74 ± 0.93 L/min whereas mean CO*IC* was 2.23 ± 1.21 L/min. Concordance for CO measurements was considered as good, since CCC was r = 0.54 (95% CI 0.43–0.63). However, mean absolute bias for CO was 0.80 L/min (40%) with an unacceptable PE of 158%. Concerning CI measurements, mean CI*TTE* was 3.74 ± 1.00 L/min/m^2^ whereas mean CI*IC* was 4.93 ± 1.95 L/min/m^2^. Concordance for CI measurements was low, since CCC was r = 0.12 (95% CI −0.08 to +0.31). Mean absolute bias for CI was 0.77 L/min/m^2^ (18%) with an unacceptable PE of 62%. Nevertheless, reproducibility of PF^®^ for CO and CI measurements was very good: ICC were r = 0.94 and r = 0.90 for CO and CI, respectively. Precision of PF^®^ for CO measurements was 6.9% (IQR 4.6–11.6%) and 7.3% for CI (IQR 4.2%-12.2%). Age, sex, weight heart rate, and hematocrit didn’t affect differences of CO and CI between the two methods. Post-hoc signal analysis revealed that only 67.4% patients had an acceptable quality for IC signal, and PF^®^’s algorithm didn’t recognize adequately the whole IC signal in 37.2% of our pediatric patients.

**Conclusion** In this first method comparison study testing CO monitoring by IC with PF^®^ in PICU, PF^®^ can’t accurately estimate CO and CI in comparison with TTE, mainly because of signal analysis and algorithm failure. Nevertheless, PF^®^ has a very good reproducibility for CO and CI measurements. Trending ability of PF^®^ should be tested, and this device could be able to monitor fluid challenge response.

**Competing interests** None.

**Reference**

1. Cecconi M, Rhodes A, Poloniecki J, Della Rocca G, Grounds RM. Bench-to-bedside review: the importance of the precision of the reference technique in method comparison studies–with specific reference to the measurement of cardiac output. Crit Care 2009;13(1):201.

#### P86 Non invasive assessment of cardiac output by echocardiography and the ultrasonic cardiac output monitor in pediatric ill patients

##### Ayari Ahmed^1^, Menif Khaled^1^, Assaad Louati^1^, Borgi Aida^1^, Khaldi Ammar^1^, Ghali Narjess^1^, Hajji Ahmed^1^, Bouziri Asma^1^, Nejla Ben Jaballah^1^

###### ^1^Unité de recherche ur12sp10, université tunis el manar, Service de réanimation pédiatrique polyvalente, Hôpital d’enfants Béchir Hamza de Tunis, Tunis, Tunisia

####### **Correspondence:** Ayari Ahmed - ahmed.alayari@gmail.com

*Annals of Intensive Care* 2017, **7(Suppl 1)**:P86

**Introduction** Accurate and reliable evaluation of cardiac index (CI) in critically ill pediatric patients can optimize their management. The ultrasonic cardiac output monitoring (USCOM, USCOM Pty Ltd, Coffs Harbour, NSW, Australia) device provides a new method of non-invasively assessing cardiac output (CO). It has been successfully used in adults, but there have been few studies in children. This device is less expensive than the echocardiography and simpler to handle. In this study, we compared non-invasive cardiac index (CI) measurements by USCOM-1A with transthoracic echocardiography (TTE).

**Patients and methods** Paired measurements of CI in pediatric critical ill patients without congenital heart disease were performed simultaneously using the USCOM-1A and echocardiography (Sonosite Turbo-M). Correlation and agreement between aortic outflow tract diameter (OTD), velocity time integral (VTI) and CO were assessed by Pearson correlation and Bland–Altman analysis.

**Results** Fifty-eight pediatric intensive care patients were enrolled (age 92 ± 277 days). The USCOM-1A and TTE measurements of CI, OTD, and VTI in all subjects were 4.18 ± 1.32 versus 2.9 ± 1.06 L/min/m^2^, 0.7 ± 0.16 versus 0.65 ± 0.19 cm and 17.23 ± 5.03 versus 12.61 ± 2.63 cm, respectively. The bias and limits of agreement of USCOM-1A compared to TTE CI were 1.29 (−0.77 to 3.32) L/min/m^2^. The percentage error in CI measurements with USCOM-1A was 49.35% relative to TTE measurements.

**Conclusion** These results suggest that the two devices can track, individually, the CO, but cannot be relied upon to provide the same values.

**Competing interests** None.

#### P87 COX-2-derived prostaglandins mediate cerebral microcirculation in a juvenile ischemic rat model

##### Pierre-Louis Leger^1^, Julien Pansiot^2^, Valérie Besson^3^, Bruno Palmier^3^, Sylvain Renolleau^4^, Olivier Baud^2^, Bruno Cauli^5^, Christiane Charriaut-Marlangue^2^

###### ^1^Réanimation néonatale et pédiatrique, Hopital pour enfants Trousseau, Paris, France; ^2^Inserm u1141, Hôpital Robert Debré, Paris, France; ^3^Pharmacologie de la circulation cérébrale, INSERM EA4475, University Paris Descartes, Faculté de Pharmacie, France, Paris, France; ^4^Réanimation pédiatrique polyvalente, Hôpital Necker - Enfants Malades, Paris, France; ^5^Inserm nps, Université UPMC, Paris, France

####### **Correspondence:** Pierre-Louis Leger - leger.pierrelouis@gmail.com

*Annals of Intensive Care* 2017, **7(Suppl 1)**:P87

**Introduction** We previously showed that the selective neuronal NO synthase inhibitor 7-nitroindazole (7-NI) increases cerebral microcirculation in a juvenile ischemic rat model [1]. We address the roles of COX-elaborated prostaglandins (Pgs) in collateral recruitment and blood supply.

**Materials and methods** Fourteen-day-old (P14) rats were subjected to ischemia–reperfusion, and treated with either PBS or 7-NI (25 mg/kg) at the reperfusion onset. Six-keto-PGF1α was measured using ELISA. COX-1 and COX-2, and prostaglandins terminal synthesizing enzymes were evaluated using reverse-transcriptase polymerase chain reaction and immunofluorescence. Microvascular blood flow indexes (artery diameter, capillaries number) were measured using sidestream dark-field videomicroscopy in PBS- and 7-NI-treated ischemic rats in the absence or presence of the COX-2 inhibitor NS-398 (5 mg/kg). Cell death was measured with the TUNEL assay and cleaved-caspase-3 immunostaining.

**Results** Six-keto-PGF1α and COX-2, associated with a PgE synthase, were significantly increased in PBS- and 7-NI-treated animals 15 min and 1 h after ischemia–reperfusion, respectively. In contrast and as compared to PBS, 7-NI significantly decreased prostacyclin synthase (PGIS) and cytosolic PgE synthase (cPGES) mRNA. Selective COX-2 inhibition significantly decreased BF indexes, and significantly reversed the effects of 7-NI including the number of TUNEL+- and cleaved-caspase-3+-nuclei. See Fig. 25 below.

**Fig. 25:**
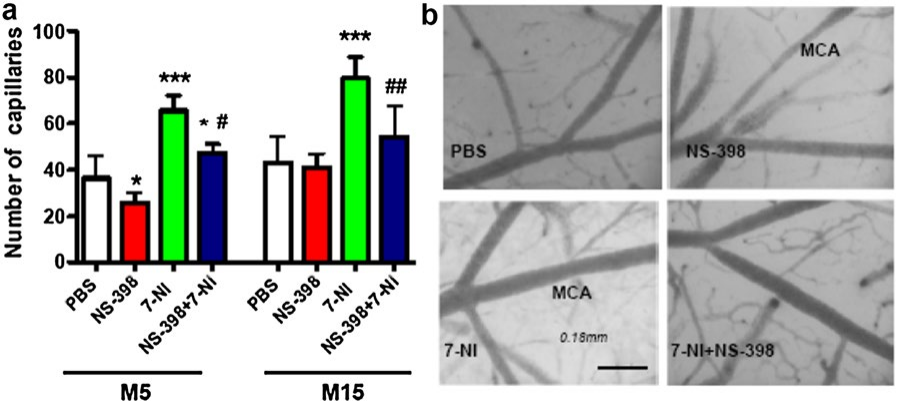
COX-2 inhibitor NS-398 reverse the 7-NI-mediated blood supply. **a** Number of capillaries measured at 5 (M5) and 15 (M15) min after re-flow in PBS-, NS-398-, 7-NI- and [7-NI+NS-398]-treated rats (n = 6 per group). **b** Digital SDF photomicrographs (0.94 × 0.75 mm) of the ipsilateral (IL) cortical circulation at 5 min after re-flow. **p < 0.01, *** p < 0.001 vs PBS. ^#^ p < 0.05; ^##^ p < 0.01; ^###^ p < 0.001 [7-NI+NS-398] vs [7-NI+NS-398] vs 7-NI. ^£^p < 0.05 [NS-398+7-NI] vs NS-398

**Discussion** We here report a role for prostaglandins (Pgs) in CBF regulation after ischemia–reperfusion in the juvenile brain. We identify specific up- (COX-2 and mPGES1) and down- (COX-1 and PGIS) regulation enzymes in the better outcome observed after nNOS inhibition. The early vascular responses of the immature and juvenile brain to ischemia are not enough investigated and well understood, however are likely to play a critical role in outcome because early cerebral perfusion inversely correlates with the extent of the lesion [2]. These data indicate (1) various Pgs biosynthesis (PgI2 in adult, PgI2, PgE2, PgF2 and/or PgD2 in neonatal-juvenile) and up- and down-regulation of specific terminal synthesis enzymes according to the developmental stage, and (2) an interaction between NO and prostaglandin synthesis via COX-2 in the juvenile P14 ischemic rat. Increasing evidences suggest a “cross talk” between NO and Pgs biosynthetic pathways but still remains to be better elucidated, namely during brain development.

**Conclusion** These results show that the juvenile rat brains mostly responds to ischemia by a COX-2-dependent prostaglandins production, and suggest that the transcriptional responses observed under 7-NI facilitate and reorient COX-2-dependent prostaglandins production.

**Competing interests** None.

**References**

1. Leger PL, Bonnin P, Moretti R, Tanaka S, Duranteau J, Renolleau S, et al. Early recruitment of cerebral microcirculation by neuronal nitric oxide synthase inhibition in a juvenile ischemic rat model. Cerebrovasc Dis. 41(1–2):40–9.
2. Bonnin P, Leger PL, Deroide N, Fau S, Baud O, Pocard M, et al. Impact of intracranial blood-flow redistribution on stroke size during ischemia–reperfusion in 7-day-old rats. J Neurosci Methods. 2015;198(1):103–9.

#### P88 Factors associated with extracorporeal membrane oxygenation treatment in congenital diaphragmatic hernia: 10 years of experience

##### Amélie Mansuy^1^, Fabrice Michel^1^, Anderson Loundou^2^, Stéphane Le Bel^3^, Julia Boubnova^4^, Fabrice Ughetto^1^, Caroline Ovaert^5^, Virginie Fouilloux^6^, Olivier Paut^1^

###### ^1^Anesthésie-réanimation pédiatrique, Hopital de la Timone, Marseille, France; ^2^Unité de recherche de santé publique, Faculté de Médecine secteur Timone (Aix-Marseille Université), Marseille, France; ^3^Anesthésie-réanimation pédiatrique, Hospital Timone, Marseille, France; ^4^Centre de référence des hernies diaphragmatiques, Hôpital de la Timone, Marseille, France; ^5^Cardiologie pédiatrique, Hôpital de la Timone, Marseille, France; ^6^Chirurgie cardiaque pédiatrique, Hôpital de la Timone, Marseille, France

####### **Correspondence:** Amélie Mansuy - amelie2602@hotmail.com

*Annals of Intensive Care* 2017, **7(Suppl 1)**:P88

**Introduction** Congenital diaphragmatic hernia is a rare anomaly that occurs in <1–5 of every 10,000 births. Pulmonary hypoplasia and pulmonary hypertension are commonly associated with congenital diaphragmatic hernia and explain its high morbidity and mortality. Despite significant advances in neonatal intensive care and protective ventilation, mortality remains as high as 50%. The development of extracorporeal membrane oxygenation (ECMO) support has improved the outcome but its use is controversial in this pathology. The main objective of this study is to determine associated factors with extracorporeal membrane oxygenation (ECMO) in congenital diaphragmatic hernia and to explore ECMO’s indications, causes of death, and evolution after discharge for survivors.

**Patients and methods** We led a retrospective study in all neonates born with congenital diaphragmatic hernia and admitted to our institution between 2003 and 2015. Antenatal data, including observed/expected foetal pulmonary volume by magnetic resonance imaging, data of the first day in paediatric intensive care unit before ECMO initiation, complications, causes of death, characteristics of ECMO group, surgical parameters and evolution of survivors after 1 month of life were recorded in the national database for congenital diaphragmatic hernia and patients’ charts.

**Results** Among the 62 cases of congenital diaphragmatic hernia, 50 infants were included with 14 (36%) requiring ECMO. Global survival rate at 1 month of life was 39/50 (78%). Lowest observed/expected lung foetal volume (p = 0.016), lowest Apgar at 1 min (p = 0.041), highest oxygenation index (p < 0.001), highest SNAP-PE 2 score (p < 0.001), left ventricle dysfunction (p = 0.046), highest lactatemia (p = 0.002), highest PaCO_2_ (p = 0.007) were significantly associated with ECMO support. Among 6 (17%) deaths in the non-ECMO group, 5 (83%) were due to absolute contraindications to ECMO. Complications occurred more frequently in the infants requiring extracorporeal membrane oxygenation support.

**Conclusion** Associated factors for the need for ECMO in congenital diaphragmatic hernia have been identified. They should be useful to determine earlier the need for ECMO and prognosis for counselling parents.

**Competing interests** None.

**References**

1. Khan AM, Lally KP. The role of extracorporeal membrane oxygenation in the management of infants with congenital diaphragmatic hernia. Semin Perinatol. 2005; 29(2):118–22.
2. Tovar JA. Congenital diaphragmatic hernia. Orphanet J Rare Dis. 2012;7:1.

#### P89 Diagnostic accuracy of abdominal compression for predicting fluid responsiveness in children

##### Matthias Jacquet-Lagrèze^1^, Nicolas Tiebergien^1^, Najib Hanna^1^, Jean-Noël Evain^2^, Florent Baudin^2^, Sonia Courtil-Teyssedre^2^, Dominique Bompard^1^, Marc Lilot^3^, Aurélie Portefaix^2^, Laurent Chardonal^1^, Etienne Javouhey^2^, Jean-Luc Fellahi^1^

###### ^1^Anesthésie réanimation, Hôpital Louis Pradel, Bron, France; ^2^Réanimation pédiatrique, Hôpital Femme Mère Enfant, Bron, France; ^3^Anesthésie, Hôpital Femme Mère Enfant, Bron, France

####### **Correspondence:** Matthias Jacquet-Lagrèze - matthias.jl@gmail.com

*Annals of Intensive Care* 2017, **7(Suppl 1)**:P89

**Introduction** Administration of fluid to increase cardiac output is a cornerstone of the hemodynamic resuscitation. The respiratory variation in aortic blood flow peak velocity (ΔVpeak) is the only variable to reliably predict fluid responsiveness in children. It requires assist control ventilation with high tidal volume (>10 ml/kg) [1]. The purpose of this study was to evaluate the clinical usefulness of assessing variation of stroke volume during a calibrated abdominal compression for the prediction of fluid responsiveness in children during acute circulatory failure.

**Patients and methods** This study was approved by local institutional review boards (CPP Lyon Sud Est II). Written informed consent was obtained from parents. Oral consent of patients old enough to understand the study was also obtained. Patient, less than 8 years old of two paediatric intensive care unit (PICU), during a 1-year period inclusion, from September 2015 to September 2016 were selected. Stroke volume index was assessed with an echocardiography at baseline, after an abdominal compression (with a calibrated pressure of 25 mm Hg), at a return to baseline, and after a volume expansion (10 ml/kg of fluid challenge over 10 min). Pulse pressure (PP) Systolic (SAP), diastolic (DAP), and mean arterial pressure (MAP); heart rate (HR), respiratory aortic blood flow velocity (ΔVpeak), left ventricular ejection fraction (LVEF), and respiratory vena cava diameter variation (VCIvar) were also recorded before volume expansion. Stroke volume index was calculated as the left ventricular outflow tract (LVOT) surface multiplied by the LVOT Velocity time integer (VTI). Patients were classified as responders to fluid loading if their stroke volume index (SVI) increased by at least 15%. R software with pROC package was used to performed descriptive and analytic statistic. Pearson correlations were performed and Receiver operative characteristic curves were built. Bootstrap technic was used to compute confidence interval (CI). p < 0.05 was considered significant.

**Results** Thirty-one children were included, 17(±22) month old and weighing 8(±5) kg. Seven were not on mechanical ventilation and 15 were in a mode allowing spontaneous breathing. 16 patients were fluid responders and 15 non-responders. All the echocardiography were performed by MJL and NT. Operators were blind from the value of the VTI for each condition. Coefficient of variation of the SVI was 8.4 (CI 5.4;11.4)% and the least significant change of five averaged SVI was 10.6 (CI 6.8–14.5)%. Changes in SVI during abdominal compression and after a fluid challenge were correlated (R^2^ = 0.796; p < 0.001). Change in SVI and respiratory vena cava diameter variation or delta Peak before abdominal compression were not significantly correlated with change in SVI during volume expansion. PP variation and MAP variation during abdominal compression were not correlated with SVI variation after fluid challenge. The ROC curve analysis showed that SVI change during abdominal compression predicts fluid responsiveness. (AUC ROC = 0.93; CI 0.82–0.99). The best threshold was 9.28% with a sensitivity 75% (CI: 0.50–0.94) and a specificity of 93% (CI 79–100). AUROC of VCIvar was 0.70 (CI 0.48–0.91). AUC ROC of ΔVpeak was 0.58 (CI 0.34–0.81).

**Discussion** Fluid responsiveness assessment, in children especially, is challenging when spontaneous breathing is authorized. It represents the vast majority of children hospitalized in PICU for acute circulatory failure. Abdominal compression is an old technic used to modify preload. Historically assessed with arterial pressure, it has never been formally evaluated so far. Our method seems quite reliable to predict fluid responsiveness, it was shown to be superior to VCIvar and ΔVpeak in our settings.

**Conclusion** SVI variation during abdominal compression was the sole reliable method to predict fluid responsiveness in a mixed population of children, with and without spontaneous breathing, suffering from acute circulatory failure.

**Competing interests** None.

**Reference**

1. Gan H. Predicting fluid responsiveness in children: a systematic review. Anesth Analg. 2013;117:1380–92.

#### P90 Epidemiology of pediatric and adult fatal anaphylaxis in France: analysis of the French national data

##### Claire Claverie^1^, Guillaume Pouessel^2^, Julien Labreuche^3^, Aimée Dorkenoo^1^, Jean-Marie Renaudin^4^, Mireille Eb^5^, Antoine Deschildre^6^, Stéphane Leteurtre^1^

###### ^1^Réanimation pédiatrique, Centre Hospitalier Régional Universitaire de Lille, Lille, France; ^2^Pédiatrie, Centre Hospitalier de Roubaix, Roubaix, France; ^3^Univ.lille, ea 2694 - santé publique : épidémiologie et qualité des soins, F-59000, Lille, C.H. Régional Universitaire de Lille (CHRU de Lille), Lille, France; ^4^Department of allergology, Emile Durkheim Hospital, Épinal, France; ^5^Department of epidemiology (cepidc), National Mortality Center, Le Kremlin-Bicêtre, France; ^6^Pediatric pulmonology and allergy department, C.H. Régional Universitaire de Lille (CHRU de Lille), Lille, France

####### **Correspondence:** Stéphane Leteurtre - stephane.leteurtre@chru-lille.fr

*Annals of Intensive Care* 2017, **7(Suppl 1)**:P90

**Introduction** Data on fatal anaphylaxis in France are limited. The aim of this study was to define the anaphylaxis mortality rate (AMR) in France between 1979 and 2011, in general and pediatric populations, and to examine the repartition by age, sex, cause and geographical regions.

**Patients and methods** All deaths in France are recorded by a physician and death certificates are collected and analyzed by the CEPIDC Unit (National Institute of Health and Medical Research, INSERM). Each death certificate records primary and secondary causes of death, demographic data including sex, age, birthday and geographic region of death. Anaphylaxis related deaths were identified by using the International Classification of Diseases (ICD) codes on the death certificates.

Databases of the French National Institute for Economical and Statistical Studies (INSEE) are available freely (www.insee.fr). Data regarding the characteristics of the French population, by region, year, sex and age were collected.

AMR were expressed per million persons and per year. All regression analyses were adjusted for age-effect and a multivariable log-linear Poisson regression model was further performed by including all of the predictors.

**Results** There were 1603 anaphylaxis related deaths in metropolitan France between 1979 and 2011, with 39 deaths in pediatric population (age less than 20 years). AMR was lower in pediatric population (0.08 per million population per annum, 95% CI 0.05–0.1) than in general population (0.83 per million population per annum, 95% CI 0.79–0.88) (p < 0.01). In general population, AMR was higher in male sex (1.08 per million population per annum, 95% CI 1–1, 16) than in female sex (0.86 per million population per annum, 95% CI 0.78–0.89) (p < 0.01). Annual percentage change for case fatality rate was −2% (95% CI −2.5 to −1.5) indicating a decrease during the study period (p < 10–4). Latrogenic cause was the most common (63%), followed by «unspecified» (23%), venom (14%) and alimentation (0.6%). AMR was the highest in persons aged ≥70 years (3.50 [95% CI 3.25–3.76]) and the lowest in children. Venom-induced mortality rate was higher in the South region (0.16) compared to the North (0.11) (p = 0.003). Only 8 food-induced fatalities were recorded (age < 32 years in 7). With the multivariate analysis, older age and male sex were associated with an increased risk for anaphylaxis death of any cause (p < 10–4).

**Conclusion** Fatal anaphylaxis decreased in France between 1979 and 2011. Higher rates of fatal anaphylaxis are observed in male sex, group aged 70 years and older and iatrogenic cause. In pediatric population, fatal anaphylaxis is low, but it’s probably under-estimated by diagnostic difficulties.

**Competing interests** None.

#### P91 The burn out in the anesthesiology department

##### Hafiani Yassine^1^, Belkadi Kamal^1^, Oboukhlik Adil^1^, Aalalam Ouafa^1^, Moussaoui Mouhamed^1^, Charkab Rachid^1^, Barrou Lahoucine^1^

###### ^1^Anesthesie Reanimation, CHU Ibn Rochd Casa, Casablanca, Morocco

####### **Correspondence:** Hafiani Yassine - docteurhafianiyassine@live.fr

*Annals of Intensive Care* 2017, **7(Suppl 1)**:P91

**Introduction** The burn out is an emotional state of exhaustion and loss of performance in response to chronic work stress. This study aims to assess the prevalence and determine the Burn out of risk factors in medical and paramedical staff in the anesthesiology department.

**Patients and methods** This was a multicenter, transversal analytic study performed within the medical and paramedical staff of the operation rooms in the central anesthesiology department in IBN ROCHD hospital in CASABLANCA. Caregivers were asked to freely and anonymously complete a questionnaire involving demographic variables, professional and an assessment of the causes and consequences of stress at work.

**Results** On a population composed of 60 caregivers, 41.7% had a score of high emotional exhaustion, 11.98% a high score of depersonalization and 47.38% a low score of professional achievement. Le burnout was found at 62.52% of our caregivers: 39.62% of them had low levels of burnout; 21.34% presented a moderate level and 1.58% had the highest level of burn out. In multivariate analysis, Doctors and Nurses Residents were most at risk of burn out. Fear of malpractice and the unsatisfactory salary multiplied the risk of burnout, respectively 2.14 and 1.83.

**Conclusion** Burn out is a threatning reality of the anesthesiology environment. The consequences could be severe on the personal and professional performance of the health institution, which involves the implementation of preventive strategies highlighting the value of work organization and the contribution of the and paramedical medical staff.

**Competing interests** None.

#### P92 Family satisfaction: impact of moving in new buildings

##### Fahmi Dachraoui^1^, Kmar Hraiech^1^, Abdelwaheb M’ghirbi^1^, Ali Adhieb^1^, Sabrine Nakkaa^1^, Islem Ouanes^1^, Saousen Ben Abdallah^1^, Hammouda Zaineb^1^, Dhouha Ben Braiek^1^, Ali Ousji^1^, Lamia Ouanes-Besbes^1^, Fekri Abroug^1^

###### ^1^Réanimation polyvalente, CHU Fatouma Bourguiba, Monastir, Tunisia

####### **Correspondence:** Fahmi Dachraoui - dachraoui.fahmi@gmail.com

*Annals of Intensive Care* 2017, **7(Suppl 1)**:P92

**Introduction** Evaluation of family satisfaction has become an essential part of the responsibility of intensivists and a major criterion for assessing the quality of care. Family satisfaction could depend on organizational and architectural characteristics of ICU. Starting from March 2016 our ICU was transferred to new buildings. The aim of the present study is to compare the level of family satisfaction before and after the change of the ICU buildings.

**Materials and methods** In a cross-sectional study we measured family members’ satisfaction in two periods (Period I: July–September 2009, old buildings; period II: August–September 2016, new buildings).We used the Critical Care Family Needs Inventory Questionnaire (CCFNI) which includes demographic characteristics of family members (age, sex, relationship, education level) and that of patients (age, sex, marital status, SAPS II, diagnosis, outcome). The 14 items of CCFNI are scored 1 point if the answer was “all the time” or “most of the time” (indicating satisfaction) and a rating of 0 if the response was “only sometimes” or “never”).

**Results** During the first and second study periods we included respectively 139 and 78 relatives of 44 and 17 patients, respectively hospitalized in our ICU for more than 48 h. The demographic characteristics of patients were similar in the two study periods: age: 59 years (IQR 19), 62% female, SAPS II = 33 (IQR 33), 78% were married, 82% of them were suffering from a chronic disease. Respiratory failure was the most frequent reason for hospitalization (73%), ICU length of stay was 14 days (IQR 19) and ICU mortality was 27%. After the transfer to new ICU, family members were more often satisfied with the information provided by the medical team (82 vs 46%; p < 0.001); only one-third of them (during both 2 periods) said they recognized the function of each member of the team (31 vs 29.4%, p = 0.4), 24.6 and 21.8% found inconsistencies in the information provided respectively in 2009 and 2016 (p = 0.38). The satisfaction of respondents on the quality of the waiting room was substantially higher in the second period than in the first period (98 vs 27.3%; p < 0.001). The average CCFNI score after the transfer of the ICU was significantly higher in the second period: 11.19 ± 2.24 vs 9.41 ± 1.64, p < 0.001(Fig 26).

**Fig. 26:**
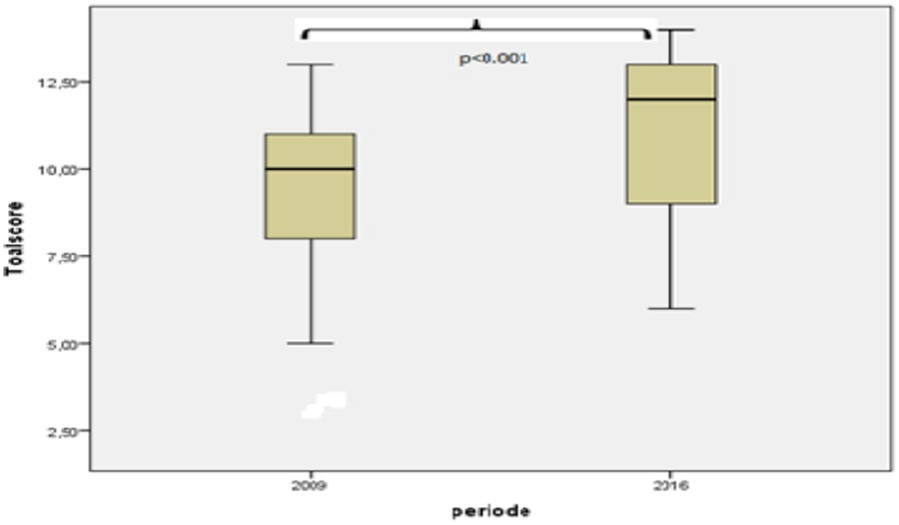
Comparison of CCFN1 scores between 2009 and 2016

**Conclusion** The transfer to a new ICU building without implementing any new policy is associated with a higher a higher satisfaction of families. A communication strategy could help improving further family satisfaction.

**Competing interests** None.

#### P93 Evaluation of professional practices: an audit and feedback intervention on antibiotic prescription in a Tunisian medical ICU

##### Dorra Mlika^1^, Hend Ben Lakhal^2^, Ahmed Khedher^2^, Khaoula Meddeb^2^, Jihene Ayachi^2^, Nesrine Sma^2^, Nesrine Fraj^2^, Messaouda Khelfa^2^, Hedia Hammed^2^, Imed Chouchene^2^, Olfa Gloulou^1^, Mohamed Boussarsar^3^

###### ^1^Pharmacy Department, CHU Farhat Hached, Sousse, Tunisia; ^2^Réanimation médicale, CHU Farhat Hached, Sousse, Tunisia; ^3^Réanimation médicale, CHU Farhat Hached, Research Laboratory N° LR14ES05. Faculty of Medicine, Sousse, Tunisia

####### **Correspondence:** Mohamed Boussarsar - hamadi.boussarsar@gmail.com

*Annals of Intensive Care* 2017, **7(Suppl 1)**:P93

**Introduction** The improvement of patient safety and quality of health care is the main goal of the Joint Commission International standards. One of these sections covers medication management and use. The standard recommends the requirement for a trained professional to review medication orders or prescriptions for appropriateness. In practice, this review process is usually performed by a clinical pharmacist. The aim of our study was to evaluate the impact of the clinical pharmacist intervention in medication orders.

**Patients and methods** This prospective study was conducted in a 7-bed medical ICU in Sousse, between November 2014 and August 2015. Patients who were admitted for at least one overnight stay were included. This study was performed in two periods, 1/an audit to identify the discrepancies of the medical prescriptions, 2/a “before/after” intervention study to evaluate the impact on medical prescriptions. The French Society of Clinical Pharmacy sheet was used to collect data.

**Results** Over a 10 months’ period, 68 patients were followed and 379 sheets were analyzed, gathering all the pharmacological classes. 148(39) discrepancies were identified, mainly related to *drug interactions* (69 (46.6)). 42% had no clinical impact, 42% had mild and none had severe impact. The most common classes involved in medication discrepancies were anti-infective, 53 (35.8); cardiovascular, 43 (29); systemic hormonal preparations, 14 (9.5) and anticoagulants, 13 (8.7). The post intervention period was addressed to the anti-infective drugs’ prescription. The discrepancies rate was significantly reduced from 53 (63) to 33 (39) respectively in a total of 84 patients.

**Conclusion** Discrepancies seem to be common in a medical ICU, involving mainly drug interactions with rather a mild clinical impact. This could be significantly reduced by the intervention of a clinical pharmacist.

**Competing interests** None.

#### P95 Impact of human factors on improving the management of the syndrome hellp

##### Setti-Aouicha Zelmat^1^, Djamila-Djahida Batouche^2^, Belkacem Chaffi^3^, Fatima Mazour^4^, Nadia Benatta^5^

###### ^1^Anesthésie réanimation chirurgicale, EHU 1er Novembre, Oran, Algeria; ^2^Réanimation pédiatrique, Centre Hospitalier et Universitaire d’Oran, Oran, Algeria; ^3^Service de gynéco-obstétrique, EHS 1er Novembre, oran, Algeria; ^4^Anesthesie -réanimation chirurgicale, EHS 1ER NOVEMBRE, oran, Algeria; ^5^cardiologie, Centre Hospitalier et Universitaire d’Oran, Oran, Algeria

####### **Correspondence:** Djamila-Djahida Batouche - khedidjabatouche@yahoo.fr

*Annals of Intensive Care* 2017, **7(Suppl 1)**:P95

**Introduction** Some aircraft accidents are linked to human error. 50% of deaths syndrome Hellp (SH) are due to late diagnosis, these accidents could be avoided by the application of particular procedures involving the human factor (HF). Yet the management of (HS), continues not consider the complications at an angle of disease severity. Human behavior, strongly influenced by the lack of training, organization, awareness is rarely implicated in the chain of events that leads to failure. Our goal is to improve the management of SH based on the (HF), largely underestimated in obstetric emergencies. While this sector requires alertness, concentration and responsiveness on the part of medical staff.

**Patients and methods** Single-center prospective study at the University Hospital Establishment OranFirst Observational from 1 January 2014 until the end of December 2014, Interventional then from 1 January 2015 until the end of December 2015 by the introduction of HF which necessitated a revision of the monitoring protocols according to the latest recommendations, with patients developing a training record of their pathology and awareness of nursing staff on shift work, the interest of the multidisciplinary management, good coordination and the establishment of an electronic registry Excel facilitating monitoring of each patient and for comparing characteristics taken into charge of two populations.

The average waiting time (AWT): Average time between the arrival of the patient and her admission.

The average time management (ATM): Average time between hospitalization and administration of the first treatment.

The mean time to blood components (MTBC): Average time between the application and administration of blood products.

The average hospital stay (AHS).

**Results** Records of these women were studied. The characteristics of women are the same for both populations, but there is a net reduction of maternal mortality 0–4 and the satisfaction rate increased from 31 to 71%. The features of management are summarized (see Table 18).

**Table 18:** Features of management

	Population 1 (N = 92 patients)	Population 2 (N = 112 patients)
AWT	30 min	15 min
ATM	49 min	24.5 min
MTBC	4 h 30	2 h 30
AHS	29 days	20 days

*AWT* average waiting time, *ATM* average time management, *MTBC* mean time to blood components, *AHS* average hospital stay

**Discussion** Our institution demand for unscheduled care and frequency of emergencies are increasing in the service of emergency obstetric gynecological similar phenomenon reported in some publications. Our study marks a reduced waiting time in half, as in a study published in France [2]. Despite the protocol established for the second population, we note a major impact for the average time of care, and the average time to obtain blood components is as long, related organizational deficits at night and on weekends, but this has allowed us to reduce the mortality rate and duration so stay on the economic cost.

**Conclusion** The human factor has a direct impact on the length of hospitalization and thus the economic cost, on maternal mortality, an indirect impact on satisfaction patient. Il is time to realize that the human factor is the success factor.

**Competing interests** None.

**References**

1. Horwitz LI, Green J, Bradley EH. US Emergency department performance on wait time and length visit. Ann Emerg Med. 2010;55:133–41.
2. Coutin AS, Vaucel E. Analyse de l’évolution des temps d’attente et durées de passage aux urgences gynécologiques et obstétricales du CHU de Nantes entre 2005 et 2012. J Gynecol Obstet Biol Reprod. 2014;43:371–8.

#### P96 Involvement of paramedical staff newly recruited in intensive care in their own training

##### Sahar Habacha^1^, Ines Fathallah^1^, Rafaa Aloui^1^, Aymen Zoubli^1^, Nadia Kouraichi^1^

###### ^1^Intensive care unit, regional hospital of Ben Arous, Tunis, Tunisia

####### **Correspondence:** Ines Fathallah - ines822004@yahoo.fr

*Annals of Intensive Care* 2017, **7(Suppl 1)**:P96

**Introduction** In the absence of specific academic training in intensive care for paramedical personnel and in order to improve skills, training is necessary especially before the opening of a new unit. The aim of our study was to evaluate the training of newly recruited in intensive care paramedical staff.

**Patients and methods** For initiation of a new intensive care unit, medical staff prepared a specific training program of resuscitation. The schedule of the training has been distributed to all relevant staff in advance.

Presentations (basics and technical data sheets) on various resuscitation themes were prepared by the staff and reviewed by doctors’ leaders. The evaluation form contained 16 tests (T). Each test consisted of five questions before the presentation and five after.

An increase in the evaluation mark obtained after the presentation is considered an improvement in knowledge.

**Results** Twenty of the staff: twelve nurses, four technicians in intensive care and four healthcare assistant participated in the training. Fifty-five percent of the staff were working in the private sector before their recruitment. The median time between graduation and recruitment was 1 year and a half. Technicians group had the highest level of knowledge (Table 19).

**Table 19:** Evaluation of pre-formative knowledge: rate score above average

	T1	T2	T3	T4	T5	T6	T7	T8	T9	T10	T11	T12	T13	T14	T15	T16	M
Nurse (%)	11	0	60	20	10	20	10	50	54	83	27	75	90	90	33	91	45
Technicien (%)	0	75	75	100	0	25	100	75	100	100	33	100	100	66	66	66	67
Healthcare assistant (%)	0	0	25	50	0	0	25	25	33	33	0	75	100	50	50	100	33

The analysis of the post-formative evaluation noted an increase in knowledge. The healthcare assistant group had the lowest rate of improvement (Table 20).

**Table 20:** Knowledge improvement percentage

	T1	T2	T3	T4	T5	T6	T7	T8	T9	T10	T11	T12	T13	T14	T15	T16	M
Nurse (%)	60	55	70	80	50	60	90	75	70	36	66	63	30	77	41	58	63
Technicien (%)	100	25	75	25	25	75	50	25	66	66	66	66	100	100	33	33	58
Healthcare assistant r (%)	75	50	75	66	0	100	66	25	25	66	100	66	0	0	25	25	47

**Conclusion** The direct involvement of paramedical staff in their own training increases their motivation without harming the quality and the main objective: knowledge improvement.

**Competing interests** None.

#### P97 Burnout in newly recruited paramedical intensive care staff: before and after theory and practical training evaluation

##### Sahar Habacha^1^, Ines Fathallah^2^, Aymen Zoubli^1^, Rafaa Aloui^1^, N. Kouraichi^1^

###### ^1^Intensive Care Unit, Regional Hospital of Ben Arous, Tunis, Tunisia; ^2^Intensive Care Unit, Regional Hospital of Ben Arous Tunisia, Tunis, Tunisia

####### **Correspondence:** Ines Fathallah - ines822004@yahoo.fr

*Annals of Intensive Care* 2017, **7(Suppl 1)**:P97

**Introduction** Burnout is a severe form of stress that affects mostly health professionals, in particular intensive care staff. Our study aims to assess the burnout prevalence in newly recruited paramedical intensive care staff before and after theory and practical training.

**Patients and methods** Before the opening of a new intensive care unit, three questionnaires were filled by the paramedical staff: at the taking up, at the end of theory training and 3 months after practical training in intensive care units.

The level of burnout was assessed using the “Maslach Burn Out Inventory” score and the degree of depression with Major Depression Inventory (MDI) test.

**Results** Twenty of the paramedical staff: twelve nurses, four technicians in intensive care and four healthcare assistants participated in the study. Only one questionnaire has not been analyzed, it belonged to a nurse presenting a serious mood disorder. Forty-five percent of the study population was unemployed before their recruitment. Eight individuals (40%) showed burnout the first day of work, 11 (55%) at the end of training and 12 (60%) after 3 months of training (Table 21).

**Table 21:** Burnout level

Level	Burnout at day 1 (N = 8)	Burnout after the training (N = 11)	Burnout after 3 months of training (N = 12)
Low	8	8	3
Moderated	0	2	6
High	0	1	3

Regarding the sub-dimensions of burnout there was an improvement of professional fulfillment (increasing from 35 to 45% after training and practicum). We also noticed a worsening of emotional distress (increasing from 0 to 25%). There was also an improvement in mood disorders (decreasing of 35–15%).

**Conclusion** Theoretical and practical courses enhanced the level of professional achievement but were not sufficient alone to prevent Burnout.

**Competing interests** None.

#### P98 Does serum procalcitonin predict the onset of toxic acute hepatitis in acetaminophen poisoning?

##### Shireen Salem^1^, Antoine Goury^1^, Isabelle Malissin^1^, Pierre Garçon^1^, Lamia Kerdjana^1^, Sebastian Voicu^1^, Nicolas Deye^1^, Marion Soichot^2^, Eric Vicaut^3^, Bruno Megarbane^1^

###### ^1^Department of medical and toxicological critical care, Lariboisière Hospital, Paris, France; ^2^Laboratory of toxicology, Lariboisière Hospital, Paris, France; ^3^Department of biostatistics, Lariboisière Hospital, Paris, France

####### **Correspondence:** Bruno Megarbane - bruno.megarbane@aphp.fr

*Annals of Intensive Care* 2017, **7(Suppl 1)**:P98

**Introduction** Procalcitonin (PCT) is a pro-hormone mainly produced by the thyroid C cells and routinely used as diagnostic biomarker of bacterial infection. PCT synthesis has never been described in the liver. To our best knowledge, no study investigated the possible predictive value of PCT in acetaminophen poisoning. Our objectives were to report the distribution of serum PCT values in acetaminophen-poisoned patients according to the onset and severity of their toxic liver injury in order to assess any possible predictive value for this biomarker.

**Patients and methods** We conducted a retrospective single centre observational study including all acetaminophen-poisoned patients (either accidentally or voluntary) admitted to the ICU from 2013 to 2016. Patients were treated with the 3-bag N-acetylcysteine protocol according to the international recommendations based on the interpretation (when possible) of the plasma acetaminophen concentration on the Rumack-Matthew nomogram (line to treat the patient starting at 150 mg/L at the 4th hour). Serum PCT was measured using an automated method (Elecsys^®^ and Cobase^®^ analyzers; range: 0.02–100 ng/mL) and plasma acetaminophen concentrations were determined using spectrophotometry. Comparisons were performed using Chi-2 and Mann–Whitney tests.

**Results** Seventy patients (50F/20M; age: 34 years [21; 53] (median [25; 75 percentiles]; poly-intoxications: 83%) were included in the study. The presumed ingested acetaminophen dose was 15.5 g [8.0; 29.0]. The delay between acetaminophen ingestion and N-acetylcysteine infusion was 4.5 h [2.9; 9.0]. Serum PCT was markedly increased above the 1 μg/L threshold in the patients who already presented or further developed significant liver cytolysis defined by serum alanine aminotransferase (ALAT) >100 UI/l (2 N) despite the treatment with N-acetylcysteine and independently of the onset of any bacterial infection, with a specificity of 97.9% and sensitivity of 69.6%.

**Conclusion** Serum PCT measurement in acetaminophen-poisoned patients admitted to the ICU is helpful to early identify patients who present significant acetaminophen-related liver toxicity already established or in progress on admission despite the administration of N-acetylcysteine according to the international recommendations.

**Competing interests** None.

#### P99 Venlafaxine poisoning in the intensive care unit: clinical presentation and role of the cytochrome P450 2D6 phenotype in the onset of cardiovascular complications

##### David Ambroise^1^, Anne-Marie Loriot^2^, Isabelle Malissin^1^, Lucie Chevillard^3^, Marion Soichot^4^, Emmanuel Bourgogne^4^, Bruno Megarbane^1^

###### ^1^Department of medical and toxicological critical care, Lariboisière Hospital, Paris, France; ^2^Laboratory of biochemistry, HEGP, Paris, France; ^3^Inserm u1144, Paris-Descartes University, Paris, France; ^4^Laboratory of toxicology, Lariboisière Hospital, Paris, France

####### **Correspondence:** Bruno Megarbane - bruno.megarbane@aphp.fr

*Annals of Intensive Care* 2017, **7(Suppl 1)**:P99

**Introduction** Venlafaxine, an antidepressant drug with properties of serotonin and norepinephrine reuptake inhibition, may be responsible in overdose for life-threatening cardiovascular complications. Venlafaxin-related toxicity additionally includes a possible interaction at elevated concentrations with the membrane sodium channels resulting in membrane stabilizing effects. To date, vulnerability factors to develop such cardiovascular complications are unknown. Based on a limited number of reported cases, poor cytochrome P450 (CYP) 2D6 metabolizers have been suggested to develop increased cardiovascular toxicity. This liver enzyme metabolizes venlafaxine to O-desmethyl-venlafaxine (or norvenlafaxine) and altered norvenlafaxine-to-venlafaxine metabolic ratio was suggested to support cardiotoxicity onset. We aimed to describe venlafaxine-related toxicity in patients admitted to the intensive care unit (ICU) and test the proposed hypothesis of vulnerability.

**Patients and methods** We conducted a prospective single centre observational study including all venlafaxine-poisoned patients admitted to the ICU from 2010 to 2016. Plasma venlafaxine and norvenlafaxine concentrations were determined using gas chromatography coupled to nitrogen–phosphorus detector (GC-NPD) after initial detection using high-performance liquid chromatography coupled to diode array detector and mass spectrometer (LC-DAD/MS). CYP2D6 genotyping was performed with the patient’s consent, allowing classifying the patients into poor, rapid, and ultra-rapid CYP2D6 metabolizers.

**Results** Fifty-two patients (60% F/40% M; age: 44 years [32; 52], median [25; 75 percentiles]) exposed to venlafaxine (presumed ingested dose: 1.9 g [1.0; 3.0]; plasma venlafaxine concentration on admission: 0.8 mg/L [0.3; 2.0] and at the peak: 0.9 mg/L [0.4; 2.7]; 98% poly-intoxications) were included. Clinical features included consciousness impairment (Glasgow coma score: 8 [4; 14]) and seizure onset (14%), requiring mechanical ventilation (56%). Nineteen patients (37%) presented cardiovascular complications and three patients (6%) died in the ICU. Based on an univariate analysis, onset of cardiovascular toxicity was significantly associated with deeper coma (p = 0.04), reduced PaO_2_/FiO_2_ ratio (p = 0.004), onset of acute renal failure (p = 0.02), requirement of mechanical ventilation (p = 0.02) and fatality (p = 0.04). No statistical relationships were found between cardiovascular toxicity and plasma venlafaxine concentration and norvenlafaxine-to-venlafaxine ratio on admission and at their respective peaks. When focusing on the patients with cardiovascular manifestations strictly attributed to venlafaxine toxicity, no statistical link was found with CYP2D6 phenotype.

**Conclusion** Venlafaxine poisoning may result in severe complications including cardiovascular toxicity and even fatality. Cardiac toxicity is responsible for increased morbi-mortality but is not related to CYP2D6 phenotype. However, inclusion of additional patients is still warranted in our possibly underpowered study before any definitive conclusion.

**Competing interests** None.

#### P100 Life threatening suicide attempts in Tunisia: an epidemiological and prognostic study

##### Hatem Ghadhoune^1^, Guissouma Jihene^1^, Insaf Trabelsi^1^, Hend Allouche^1^, Habib Brahmi^1^, Mohamed Samet^1^, Hatem El Ghord^1^

###### ^1^Réanimation médicale bizerte, Faculté de médecine de Tunis, Bizerte, Tunisia

####### **Correspondence:** Hatem Ghadhoune - ghadhoune@yahoo.fr

*Annals of Intensive Care* 2017, **7(Suppl 1)**:P100

**Introduction** Suicide attempts represent a major public health problem in Tunisia according to recent data published in 2015 by the Tunisian National Social Observatory. However, data about the incidence of life threatening suicide attempts requiring intensive care unit (ICU) admission are lacking.

The aim of this study is to describe the epidemiological profile of critically-ill patients admitted for suicide attempts and to identify factors predicting prolonged ICU stay (>3 days).

**Patients and methods** We conducted a retrospective study in the medical ICU of Bizerte city (Tunisia). From January 2009 to December 2015, 219 patients were consecutively admitted for suicide attempt. Three patients were excluded because of lacking data and 216 cases were included in the study.

**Results** The median age was 23 years [12–69]. The mean IGS2 score was 20.5 ± 14.5 and the mean APACHE2 was 8.1 ± 7. Psychotropic drugs overdose (43.1%) and pesticides ingestion (25.5%) were the two most common causes of suicide attempts. Eighty-one patients (37.5%) had a psychiatric history. Coma (32.9%) and respiratory distress (22.7%) were the two major reasons for ICU admission. Mechanical ventilation was required for 84 patients (38.9%). Median duration of mechanical ventilation was 2 days [1–9]. Overall mortality was 3.2%. The median ICU length of stay for the 209 survivors was 2j [1–19]. Factors associated with prolonged ICU stay (>3 days) were coma, hemodynamic instability, hypoxia, aspiration and mechanical ventilation.

**Conclusion** The current study is the largest study in Tunisia describing the epidemiological features of critically-ill patients admitted for suicide attempts. Identifying the underlying risk factors leading to suicide attempts and psychiatric follow-up starting upon ICU discharge are of paramount importance.

**Competing interests** None.

**Reference**

1. L’observatoire social tunisien. Rapport annuel “suicide et tentatives de suicide en Tunisie 2015″.

#### P101 Neurobehavioral effects of lithium in the rat: investigation of the effect/concentration relationships and the contribution of the poisoning pattern

##### Anne-Sophie Hanak^1^, Lucie Chevillard^1^, Rodolphe Lebeau^1^, Patricia Risede^1^, Jean-Louis Laplanche^1^, Nadia Benturquia^1^, Bruno Megarbane^2^

###### ^1^Inserm u1144, Paris-Descartes University, Paris, France; ^2^Department of medical and toxicological critical care, Lariboisière Hospital, Paris, France

####### **Correspondence:** Bruno Megarbane - bruno.megarbane@aphp.fr

*Annals of Intensive Care* 2017, **7(Suppl 1)**:P101

**Introduction** Severity of lithium poisoning depends on the ingested dose, previous treatment duration and renal function. No animal study has investigated neurobehavioral differences in relation to the lithium poisoning pattern observed in humans, while differences in lithium pharmacokinetics have been reported in lithium-pretreated rats mimicking chronic poisonings with enhanced brain accumulation in rats with renal failure. Our objectives were: (1) to investigate lithium-related effects in overdose on locomotor activity, anxiety-like behavior, spatial recognition memory and anhedonia in the rat; (2) to model the relationships between lithium-induced effects on locomotion and plasma, erythrocyte, cerebrospinal fluid and brain concentrations previously obtained according to the poisoning pattern.

**Materials and methods** Open-field, elevated plus-maze, Y-maze and sucrose consumption tests were used. We developed acute (intraperitoneal administration of 185 mg/kg Li_2_CO_3_ in naive rats), acute-on-chronic (intraperitoneal administration of 185 mg/kg Li_2_CO_3_ in rats receiving 800 mg/l Li_2_CO_3_ in water during 28 days), and chronic poisoning models (intraperitoneal administration of 74 mg/kg Li_2_CO_3_ during 5 days in rats with 15 mg/kg K_2_Cr_2_O_7_-induced renal failure).

**Results** In acutely lithium-poisoned rats, we observed horizontal (*p* < 0.001) and vertical hypolocomotion *(p* < 0.0001), increased anxiety-like behavior (*p* < 0.05) and impaired memory *(p* < 0.01) but no altered hedonic status. Horizontal (*p* < 0.01) and vertical (*p* < 0.001) hypolocomotion peaked more markedly 24 h after lithium injection and was more prolonged in acute-on-chronically versus acutely lithium-poisoned rats. Hypolocomotion in chronically lithium-poisoned rats with impaired renal function did not differ from acutely poisoned rats 24 h after the last injection. Interestingly, hypolocomotion/concentration relationships best fitted a sigmoidal Emax model in acute poisoning and a linear regression model linked to brain lithium in acute-on-chronic poisoning.

**Conclusion** Lithium overdose alters rat behaviour and consistently induces hypolocomotion which is more marked and prolonged in repeatedly lithium-treated rats. Our data suggest that differences between poisoning patterns regarding lithium-induced hypolocomotion are better explained by the duration of lithium exposure than by its brain accumulation.

**Competing interests** None.

#### P102 Acute cyproheptadine poisoning: an epidemiological study

##### Hana Fredj^1^, Youssef Blel^1^, Messaouda Khelfa^1^, A. M’rad^1^, Nozha Brahmi^1^

###### ^1^Department of intensive care and toxicology, Centre d’Assistance Médicale Urgente, Tunis, Tunisia

####### **Correspondence:** Youssef Blel - blelyoussef@yahoo.fr

*Annals of Intensive Care* 2017, **7(Suppl 1)**:P102

**Introduction** Cyproheptadine (Periactin^®^) is a histamine H1 receptor and serotonin antagonist, it has also anticholinergic effects. It is used to treat allergic type symptoms and as an appetite stimulator. Cyproheptadine poisoning has been rarely reported.

That’s why we conduct this study, to identify the epidemiological characteristics and clinical outcomes of cyproheptadine poisoning and to define the association between the ingested dose and clinical manifestations.

**Patients and methods** Retrospective study was performed between January 2011 and December 2015. Epidemiological data, clinical data and outcome were reviewed. Data were analyzed by statistical methods in SPSS.

**Results** A total of 36 patients were reviewed with a mean age of 21 [14, 27]. All patients had no psychiatric background. All cases were self-inflicted. Mean ingested dose was 72 mg [34,143]. Mean delay of symptoms onset was 2 h [1, 4]. Neurological symptoms included delirium in 17% of cases, agitation, and hallucinations. Thirty-six percent of patients had peripheral anticholinergic symptoms. Gastric lavage was performed in 8% of cases. Four patients were admitted to ICU and required mechanical ventilation (MV) for neurologic impairment. Mean duration of MV was 17 h ± 4. The outcome was favourable in all cases. Length of hospital stay was less than 24 h with an average of 22 h [20, 24]. There is no correlation between coma and assumed ingested dose.

**Conclusion** Cyproheptadin overdose causes mainly neurological effects resulting from anticholinergic syndrom. Outcome is generally favourable.

**Competing interests** None.

#### P103 Cardiogenic shock complicating acute beta blockers intoxication: epidemiology and treatment

##### A. M’rad^1^, Fatma Essafi^1^, Hend Ben Lakhal^1^, A. Benabderrahim^1^, H. Thabet^2^, Y. Blel^1^, N. Brahmi^1^

###### ^1^Department of intensive care and toxicology, Centre d’Assistance Médicale Urgente, Tunis, Tunisia; ^2^Department of emergency, Centre d’Assistance Médicale Urgente, Tunis, Tunisia

####### **Correspondence:** A. M’rad - mrad.aymen@gmail.com

*Annals of Intensive Care* 2017, **7(Suppl 1)**:P103

**Introduction** In spite of its rarity, the acute Beta blockers (BB) intoxication represents a major cause of mortality as a result of cardiovascular toxicity. (CS) is the most serious complication of this intoxication.

The objectives of this study were to determine epidemiological, clinical, management and prognosis of cardiogenic shock (CS) complicating acute BB intoxication.

**Patients and methods** This was a retrospective descriptive study conducted at the Medical Intensive Care Unit of CAMU over a 10 years period from January 2006 to December 2015. All hospitalized patients for cardiogenic shock complicating pure acute BB intoxication were included.

**Results** Twenty-five patients were included. The average age was 23 ± 7 years with a female predominance (sex ratio = 0.32). Three different molecules were involved: Acebutolol (n = 22), Atenolol (n = 2) and Propranolol (n = 1). The assumed average dose of ingested Acebutolol was 4000 [3200; 4800] mg. All patients were hospitalized in the first 3 h of taking and CS occurred within 3 h post ingestion in all cases. Bradycardia was reported in all patients who have ingested Atenolol or Propranolol. For patients who ingested Acebutolol, bradycardia was observed in 45% of cases (n = 10). Electrocardiographic signs included a ventricular atrioventricular block in 5 cases (as first degree in 3 cases and 3rd degree in 2 cases) and a membrane stabilizing effect in 10 cases. Digestive decontamination was performed in 14 patients (56%). All patients were treated using catecholamines: epinephrine in 18 patients (72%), both dobutamine and norepinephrine in 6 patients and only dobutamine in 3 patients. Glucagon was administered in 8 cases. Semi- molar bicarbonate intake as infusion of 500 ml into 30 min was prescribed to 10 patients who have membrane stabilizing effect. Eighteen patients required mechanical ventilation. The median duration of hospital stay was 72 [18; 120] h. Thirteen patients (52%) died within 24 h.

**Conclusion** Management of Cardiogenic shock complicating acute BB intoxication is largely symptomatic and based on vasopressors, positive inotropic agents. Sodium salts could be an alternative measure in cases of membrane stabilizing effect, and temporary cardiac electrical pacing if high degrees BAV. In refractory shock circulatory support should be initiated as early as possible.

**Competing interests** None.

#### P104 Purifying efficiency of CVVHDF and MARS in a simulated poisoning by verapamil

##### Romain Jouffroy^1^, Caill Nicolas^2^, Pascal Philippe^1^, Maraffi Tomasso^1^, Jean-Herlé Raphalen^3^, Jean-Claude Alvarez^4^, Benoit Vivien^5^, Lionel Lamhaut^6^, Carli Pierre^1^, J Baud Frédéric^1^

###### ^1^Anesthésie Réanimation SAMU, APHP - CHRU Necker Enfants Malades, Paris, France; ^2^Service de pharmacologie toxicologie, APHP - Hopital Raymond Poincaré, Paris, France; ^3^Service de réanimation adulte, Hôpital Necker, Assistance Publique Hôpitaux de Paris, France; ^4^Paris, Assistance Publique Hôpitaux de Paris, Paris, France; ^5^Réanimation adulte - samu, Hôpital Necker - Enfants Malades, Paris, France; ^6^Réanimation adulte, Hôpital Necker - Enfants Malades, Paris, France

####### **Correspondence:** Romain Jouffroy - romain.jouffroy@aphp.fr

*Annals of Intensive Care* 2017, **7(Suppl 1)**:P104

**Introduction** Drug poisoning is a frequent cause of hospital admission especially in intensive care unit (ICU). Despite progress, hospital mortality of severe acute poisoning admitted in ICU seems to increase. Purifying methods such as continuous haemodiafiltration veinoveinous (CVVHDF) and molecular adsorbent recirculating system (MARS), were developed with promising clinical results (1). However no analytical study has quantified accurately their purifying efficiency. It has not been assessed efficiency of the different compartments of the MARS nor its advantages over other methods of dialysis and filtration. The objective of this study was to quantify the purifying efficiency of the different compartments of the CVVHDF and MARS and to compare their respective efficiency in an ex vivo model in the most favourable conditions for these methods to assess their capability maximum treatment.

**Materials and methods** We performed a ex vivo study based on manipulation bench simulating intoxication verapamil with a theoretical plasma doses of 1, 2.5 and 5 mg/l injected into a central compartment of 5 L. Sampling and assays were carried out at various points of the circuit CVVHDF and MARS. The EC extraction coefficient [EC = (in concentration − out concentration)/in concentration] was calculated for each compartment of the CVVHDF and MARS as well as the amounts withdrawn by the sum each compartment allows assessing the overall capacity of each technique. Three manipulations were performed for each concentration.

**Results** CVVHDF EC was 22, 22 and 28% for theoretical concentrations of 1, 2.5 and 5 mg/l. For the 3 previous concentrations, EC was not constant but decreased steadily over the sessions to cancel 0 +10, +5 and +4 h for the different concentrations with even become negative after up to 14 h (end handling). At the end of handling the amount remaining in the central compartment was 6, 3 and 4% of injected dose. The amounts found in the effluent corresponded to 16, 20 and 28% of injected dose. In fact the amounts “not found” accounted for 78, 79 and 69% of injected dose. A desorption ultrasound membranes revealed that verapamil was fixed by the membrane of the CVVHDF. During all handling (12–14 h) concentrations in the circuit output “patient” for the CVVHDF have never been undetectable but remained stable.

The different compartments of MARS led instead to an undetectable concentration at the exit of “patient” circuit. Similarly the concentration of verapamil remaining in the central compartment was undetectable at the end of the handling and this for all three concentrations studied. The quantities withdrawn by the hemodiafiltration compartment MARS corresponded to 8, 18 and 12% of the injected quantity, EC varied between 10 and 20% remaining stable. The quantities withdrawn by the coal compartment activated MARS was 92, 82 and 89% of the injected amount, the EC induced by MARS cartridge was stable in the order of 70% throughout the manipulation.

**Discussion** CVVHDF and MARS are 2 effective purifying methods for removal of a simulated poisoning by verapamil. Their action and performance modes are very different. The membrane used in CVVDHF, adsorption verapamil realized 80% of the elimination of the toxic but with a residual salting out effect. A 8 h session of MARS had the effect of making them undetectable concentrations in the central compartment. The purifying effect is bound to 80% to the effect of carbon cartridge and for 10–20% to hemodiafiltration.

**Conclusion** Clearance is a simple parameter that two determinants are EC and flow in the scrubber compartment. Pharmacokinetic modeling that require constant clearance. CVVHDF is characterized by a decrease in EC resulting in a variable clearance and preventing modeling. Elimination of verapamil is mainly due to unspecific binding properties. MARS is particularly effective in poisoning by verapamil. These results must be tempered by the fact that the conditions of our manipulations, without protein in the central compartment, evaluate the maximum ex vivo treatment capacity.

**Competing interests** None.

#### P105 Acute beta blocker overdose management: Factors associated with cardiovascular mortality in a Caribbean intensive care unit

##### Dabor Resiere^1^, Ruddy Valentino^2^, Cyrille Charbatier^1^, Sabia Marie^1^, Bruno Sanchez^1^, Jean-Louis Ferge^3^, Julien Fabre^3^, Jocelyn Inamo^2^, Bruno Megarbane^4^, Hossein Mehdaoui^1^

###### ^1^France, Centre Hospitalier Universitaire de Fort de France, Fort-de-France, Martinique; ^2^France, Centre Hospitalier Universitaire de Fort de France, Fort-de-France, France; ^3^Réanimation Polyvalente, Centre Hospitalier Universitaire de Fort de France, Fort-de-France, Martinique; ^4^Service de Réanimation Médicale et Toxicologique, CHU Lariboisière, Paris, France

####### **Correspondence:** Dabor Resiere - dabor.resiere@chu-fortdefrance.fr

*Annals of Intensive Care* 2017, **7(Suppl 1)**:P105

**Introduction** Beta-adrenergic antagonists are commonly used worldwide to treat hypertension, tremor, migraines, ischemic heart disease, heart failure, arrhythmias, portal hypertension, angina and panic attack. Propranolol, a beta-adrenergic antagonist with membrane stabilizing proprieties, is the most common toxic used in suicide attempts in Martinique.

Through respiratory depression, bronchopasm, bradycardia, severe hypotension, and seizures may result from beta blocker intoxication, cardiovascular depression appears to be the most common cause of morbidity and mortality in severe acute beta blocker poisoning. Massive beta-blocker ingestions may cause prolonged QRS intervals may also be associated with refractory cardiac failure. Our objectives were to determine factors that are associated with the development of cardiovascular mortality.

**Patients and methods** We conducted a retrospective study over 10 years (January 2005 to December 2015), including all poisoned patients admitted and treated to the Emergency Department and the Intensive Care Unit. During this period, there were over 10 beta-adrenergic antagonist exposures per year reported by the medical records department. These poisonings accounted for an average of 5 deaths annually.

**Results** Three Hundred and eight patients (173 males/135 females) were admitted to the ICU for severe acute poisonings. Median age, 46.5% years (16–79); SAPS II, 120 (49–94). Among these 308 patients, 100 had ingested high doses of cardiotoxicants [class I ant-arrhythmic drugs (40%), β-blockers (15%), calcium-channel blockers (10%)]. Fifty patients (50%) survived, including 18 to prolonged cardiac arrest. Bad prognostic factors in ECLS-treated poisoned patients for beta blocker poisoning, were as follows: QRS enlargement on admission, SAPS II score on admission, Extracorporeal Life Support (ECLS) performance under massage, potential co-ingestants, arterial pH and lactate concentration (10.5 mmol/l).

**Conclusion** The most important factor associated with an increased risk of cardiovascular mortality in beta blocker poisoning is the exposure to a beta blocker with stabilizing activity. The identification of risk factors allows physicians to identify patients at greatest risk. ECLS appears to be an efficient salvage technique in case of refractory toxic cardiac failure or arrest.

**Competing interests** None.

**Reference**

1. Jeffrey NL, John MH, Toby LL, Wendy KS. Acute Beta blocker overdose: factors associated with the development of cardiovascular mortality. Clin Toxicol 2000;38:275–81.

#### P106 Accidental poisoning in children aged under 6 years old admitted in pediatric intensive care unit: For which toxic?

##### Djamila-Djahida Batouche^1^, Nadia Benatta^2^, Amel Zerhouni^3^, Kheira Tabeliouna^1^, Setti-Aouicha Zelmat^4^, Amine Negadi^1^, Zahia Mentouri^1^

###### ^1^Réanimation pédiatrique, Centre Hospitalier et Universitaire d’Oran, Oran, Algeria; ^2^cardiologie, Centre Hospitalier et Universitaire d’Oran, Oran, Algeria; ^3^Reanimation, EHS CANASTEL, Oran, Algeria; ^4^Anesthésie réanimation chirurgicale, EHU 1er Novembre, Oran, Algeria

####### **Correspondence:** Djamila-Djahida Batouche - khedidjabatouche@yahoo.fr

*Annals of Intensive Care* 2017, **7(Suppl 1)**:P106

**Introduction** Accidental acute intoxications are part of pediatric medical emergencies of our hospital experience. Children are most at risk of accidental poisoning due to characteristics of their development and their environment. Our Goal is to point to the toxic substances involved and to draw up the outcome profile in these patients.

**Patients and methods** Retrospective study, covering all poisoning cases occurred in children under 6 years old and admitted to PICU in the university hospital Oran.

We specified the characteristics of children, the nature of the toxic, symptoms on admission, the therapeutic aspect, the evolutionary mode and length of hospital stay. The duration of our study was to 4 years, from 2011 to 2015.

**Results** In those years, the ICU admitted 365 poisonings occurring in children. They represent 13% of all admissions and 71% (260 children) under 6 years old. The average age of addicts was 5.08 ± 1.36 years old with a male predominance.

All product types were involved in the poisoning. According to data, 72% caustic toxic (189 cases) followed by drug poisoning (in 28 cases) and it is basically a case of psychotropic and narcotic (01 case), and then in descending order are rodenticides poison (19 cases), organophosphorus insecticides like (11 cases), Hydrocarbon (7 cases) ingestion of plants (3 cases), making cosmetics (2 cases) and poisoning of industrial glue (01 cases). Upon arrival at the hospital, 68% of young children had gastrointestinal disorders, and 2.3% had neurological disorders (seizures, ataxia and abnormal movements), 1.1% were in respiratory distress.

After observation for most children, symptomatic treatment was introduced, 6 patients have been put under non invasive ventilation, intubation and mechanical ventilation was required in 01 patient for 16 days also put under Hemodynamic Support.

The outcome was favorable with an average hospital stay of 28 h in survivors, and 01 death occurred following an ingestion of hydrocarbons.

**Discussion** Children in pre-school age are often at risk of domestic accidents in relation to their age and environment. They ingest products wrongly deconditioned or transferred into current use containers. These by catch happen to be in the kitchen at a large household or in the bathroom. They also ingest drugs for adults and thus higher dosage: these drugs are too easily left to reach those children who are not suspected faculties to identify seize and swallow what they take to candy. A study in India positioned intoxication hydrocarbons 1st place (50.9%), followed by organophosphates (11.9%), medication and caustic identify (4.8%) cases each [1]. In Oran (Algeria) taking caustic remains at the level of poisoning in children. Overall mortality by poisoning is lower in children compared to adults. (The products in question are heterogeneous drugs, household products, plants, pesticides) We recorded 01 death secondary to acute respiratory distress syndrome following ingestion by siphoning gasoline.

**Conclusion** At the end of our study, it appears that the acute poisoning in children is a very common event. However, developments remain favorable in most cases. This should not obscure the potential severity of the poisoning, nor lose sight of the necessary prophylactic measures. This prophylaxis should include education of the population, prevention of certain tragedies related to toxins or aberrant behavior, and triggering alerts to reduce morbidity and mortality related to poisoning.

Our ACCIPED laboratory medical university Oran for a future mission secondary and tertiary prevention through continuous training for general practitioners, to respond to any request for advice on diagnosis and treatment of these accidents, assess risk through telemedicine and through a website continuously 24 h/24 h.

**Competing interests** None.

**Reference**

1. Basu K, Mondal RK, Banerjee DP. Epidemiological aspects of acute childhood poisoning among patients attending a hospital at Kolkata. Indian J Public Health. 2005;XXXXIX(1).

#### P107 Effectiveness of preoxygenation with spontaneous breathing or noninvasive positive pressure ventilation in the presence of calibrated leak: an experimental study in healthy volunteers

##### Fanny Le Gall^1^, Jean-Luc Hanouz^1^, Hervé Normand^2^, Nicolas Terzi^3^

###### ^1^Service d’anesthésie réanimation, Centre Hospitalier Universitaire de Caen, Caen, France; ^2^Service d’explorations fonctionnelles respiratoire, Centre Hospitalier Universitaire de Caen, Caen, France; ^3^Service de réanimation médicale, Clinique de Réanimation Médicale, Grenoble, France

####### **Correspondence:** Fanny Le Gall - fannylegall4@gmail.com

*Annals of Intensive Care* 2017, **7(Suppl 1)**:P107

**Introduction** Noninvasive ventilation has become the preoxygenation reference technique. In PreOx study [1], Hanouz et al. showed that the preoxygenation is faster with positive pressure ventilation with or without positive end expiratory pressure (PEEP) than spontaneous ventilation. However, during preoxygenation imperfect seal between the face mask and patient’s face can induce a leak decreasing its effectiveness. We assume that noninvasive ventilation could cancel the effect of the leak in non-obese subjects.

**Materials and methods** Experimental prospective study, randomised in cross-over of healthy volunteers from March to July 2016, in Caen university hospital. None of them were obese, smoked or had a chronic lung disease. Healthy volunteers conducted a preoxygenation with fraction of inspired oxygen at 100%: spontaneous ventilation (SV) and noninvasive ventilation (NIV, pressure support +6 cmH_2_O and PEEP +5 cmH_2_O) without and with calibrated leak (SV-leak and NIV-leak). From the volunteer mouth at respirator, subjects breathed through a mouthpiece (with a nose clip), mount catheter, antibacterial filter and Y-piece of respirator. In modes of ventilation with calibrated leaks, the leak was placed on mount catheter. The leak was characterized as a turbulent flow with the flow/pressure curve. The fraction of expired oxygen (FeO_2_), changes in respiratory rate and end-tidal of carbon dioxide were measured. A PreOx was deemed effective if FeO_2_ > 90%. The primary endpoint was the time to achieve a FeO_2_ > 90%. The secondary endpoint was the proportion of subject with FeO_2_ > 90% at the third minute.

**Results** The study included 20 healthy volunteers. The time to achieve a FeO_2_ > 90% was 85 [68–110] s, 78 [69–84] s and 84 [72–110] s in SV group, NIV and NIV-leak, respectively. The SV-leak group never reached a FeO_2_ > 90% (p < 0.001). After 3 min, 19 (95%) in SV group, 20 (100%) in NIV group, 19 (95%) in NIV-leak group and 0 (0%) in SV-leak group volunteers had a FeO_2_ > 90%. Cox proportional-hazards regression showed that NIV [hazard ratio 2.13; 95% confidence interval (95% CI) 1.09 to 4.18); p = 0.02] was associated with a shorter time to an expired O_2_ fraction of 90% than NIV-leak or SV-leak.

**Discussion** In the presence of calibrated leaks, preoxygenation is effective with noninvasive ventilation than spontaneous ventilation. The breathing patterns of every subject seems to influence the existence of inspiratory leaks. Some volunteers had more inspiratory leaks than others, and experienced a longer length of preoxygenation than others. This is probably explained by a different instantaneous inspiratory flow [2]. These results hypothesize that individual preoxygenation is needed for each subject.

**Conclusion** Noninvasive ventilation allows effective preoxygenation despite the existence of leak.

**Competing interests** None.

**References**

1. Hanouz J-L, Lammens S, Tasle M, Lesage A, Gérard J-L, Plaud B. Preoxygenation by spontaneous breathing or noninvasive positive pressure ventilation with and without positive end-expiratory pressure: a randomised controlled trial. Eur J Anaesthesiol. 2015;32(12):881–7.
2. Benchetrit G. Breathing pattern in humans: diversity and individuality. Respir Physiol. 2000;122(2–3):123–9.

#### P108 Evaluation of bag-valve-mask ventilation in manikin studies: What are the current limitations?

##### Abdo Khoury^1^, Fatimata Seydou Sall^2^, Alban De Luca^2^, Aurore Pugin^2^, Sebastien Pili-Floury^3^, Lionel Pazart^2^, Gilles Capellier^4^

###### ^1^Emergency medicine and critical care, CHU de Besançon, Besançon, France; ^2^Inserm cic 1431, CHU de Besançon, Besançon, France; ^3^Réanimation chirurgicale, CHU de Besançon, Besançon, France; ^4^Réanimation Médicale, CHU de Besançon, Besançon, France

####### **Correspondence:** Abdo Khoury - abdokhoury@hotmail.com

*Annals of Intensive Care* 2017, **7(Suppl 1)**:P108

**Introduction** Manikin-based studies for evaluation of ventilation performance show high heterogeneity in the analysis and experimental methods used as we pointed out in previous studies. In this work, we aim to evaluate these potential limitations and propose a new analysis methodology to reliably assess ventilation performance.

**Patients and methods** One hundred forty healthcare providers were selected to ventilate a manikin with two adult self-inflating bags in random order. Ventilation parameters were analysed using different published analysis methods compared to ours.

**Results** Using different methods impact the evaluation of ventilation efficiency which ranges from 0% to 45.71%. Our new method proved to be relevant and showed that all professionals tend to hyperventilate and revealed a significant relationship between professional category, grip strength of the hand keeping the mask and ventilation performance (p = 0.0049 and p = 0.0297 respectively).

**Discussion** Using adequate analysis methods is crucial to avoid many biases. Extrapolations to humans still have to be taken with caution as many factors impact the evaluation of ventilation performance. Healthcare professionals tend to hyperventilate with current devices.

**Conclusion** We believe that problems related to manual ventilation efficiency could be prevented by implementing monitoring tools in order to give a direct feedback to healthcare professionals regarding ventilation efficiency and ventilatory parameter values.

**Competing interests** None.

**References**

1. Harrison RR, Maull KI, Keenan RL, Boyan CP. Mouth-to-mask ventilation: a superior method of rescue breathing. Ann Emerg Med. 1982;11:74–6.
2. Hess D, Baran C. Ventilatory volumes using mouth-to-mouth, mouth-to-mask, and bag-valve-mask techniques. Am J Emerg Med. 1985;3:292–6.

#### P109 Manual ventilation: to intubate or not to intubate the patient, it is not the question—a manikin study

##### Fatimata Seydou Sall^1^, Alban De Luca^1^, Aurore Pugin^1^, Chrystelle Vidal^1^, Franck Leroux^1^, Lionel Pazart^1^, Gilles Capellier^2^, Abdo Khoury^3^

###### ^1^Inserm cic 1431, CHU de Besançon, Besançon, France; ^2^Réanimation Médicale, CHU de Besançon, Besançon, France; ^3^Emergency medicine and critical care, Hôpital jean Minjoz, Besançon, France

####### **Correspondence:** Abdo Khoury - abdokhoury@hotmail.com

*Annals of Intensive Care* 2017, **7(Suppl 1)**:P109

**Introduction** Basic and advanced airway management as Bag-Valve-Mask (BVM) and Endotracheal Intubation (ETI) are procedures used in prehospital resuscitation. In this work, we compare manual ventilation performance with both techniques and analyse their variability and also try to assess what factors may affect their performance.

**Patients and methods** One hundred forty healthcare providers were selected to ventilate an intubated and not-intubated manikin with two adult bags for 5 min each in a random order. Ventilation performance were analysed using a new analysis method we described in a previous study which takes into account ventilation variability.

**Results** Five hundred sixty ventilation tests (280 for ventilation using a facemask and 280 for ventilation with an Endotracheal Tube (ETT)) were performed by the 140 healthcare professionals. Results show a significant difference between ventilation performance with a mask compared to ETT (*p* < 0.05) with more ventilation efficiency when healthcare professionals ventilate with an ETT than a mask (37 vs. 21 ventilation tests). Furthermore, a significant relationship is observed between participants’ professional category, the size of the hand squeezing the bag and manual ventilation performance (*p* < 0.05).

**Discussion** Healthcare professionals have performed 560 ventilation tests on an ETT and a facemask. Among them, only 58 (10.41%) were efficient and 502 (89.64%) are inadequate (of which 11.4% insufficient and 78.2% excessive). Whatever the kind of ventilation technique used, they are still struggling to perform manual ventilation efficiently according to international guidelines.

**Conclusion** The high ventilation performance failure observed in this study shows that “to intubate or not to intubate the patient” is not the question, but we should focus on how to improve efficiency of manual ventilation performed by healthcare professionals.

**Competing interests** None.

**References**

1. Boyle M, Flavell E. Which is more effective for ventilation in the prehospital setting during cardiopulmonary resuscitation, the laryngeal mask airway or the bag-valve-mask? A review of the literature. J Emerg Prim Health Care, 2012;8:2.
2. Doerges V, Sauer C, Ocker H, Wenzel V, Schmucker P. Airway management during cardiopulmonary resuscitation—a comparative study of bag–valve–mask, laryngeal mask airway and combitube in a bench model. Resuscitation 1999;41:63–9.

#### P110 A novel and global approach of ICU ventilator ergonomics

##### Erwan L’Her^1^, Nicolas Marjanovic^2^

###### ^1^Réanimation médicale, CHRU de Brest, Brest, France; ^2^Urgences, CHU de Poitiers, Poitiers, France

####### **Correspondence:** Erwan L’Her - erwan.lher@univ-brest.fr

*Annals of Intensive Care* 2017, **7(Suppl 1)**:P110

**Introduction** Devices’ ergonomics are major determinants of task failure, especially in stressful environments. The aim of the study was to provide a global evaluation of ICU ventilator ergonomics using novel exploration tools.

**Materials and methods** *Devices and physicians* 6 new-generation ventilators were evaluated (Dräger Evita V300, Covidien PB980, Philips V680, Hamilton S1, GE Carescape R860, Maquet Servo-U) and compared to an old one (Carefusion Avea). 20 senior physicians were included, each testing up to 4 ventilators in a randomized order.

*Objective task completion* 11 specific tasks were to be completed. The test was a failure if the correct response was given ≥120 s, or in case of incorrect response or abandoned task

*User*-*friendliness evaluation* System Usability Scale (SUS) was developed to measure device’s usability. It has a range of 0–100, the highest score being the best value. Mental Workload evaluation (MWL) is an indicator for human–machine interface (HMI) comparison. 3 dimensions depending to user and 3 dimensions depending to HMI are subsequently weighed to obtain TLX result. The higher the TLX, the higher the MWL.

*Physiological measurement* Pupil diameter modifications were assessed by eye-tracking (SMI ETG 1); heart, respiratory rate and thoracic volume variations were measured with a dedicated device (Hexoskin).

**Results** No users could set inspiratory flow on Servo U, and only 18% succeeded with S1. Servo U had the worst global results (failure rate = 42%) and Avea the best (failure rate = 13%) (p = 0.12). Among all ventilators, Avea had the best SUS and TLX values, and Philips V680 the worst (p < 0.05). Eye tracking, respiratory rate and tidal volume activation differed between ventilators (p < 0.05). V300 caused the higher eye-tracking activation rate when compared to Avea (p = 0.03) and R860 (p = 0.019) (Fig. 27).

**Fig. 27:**
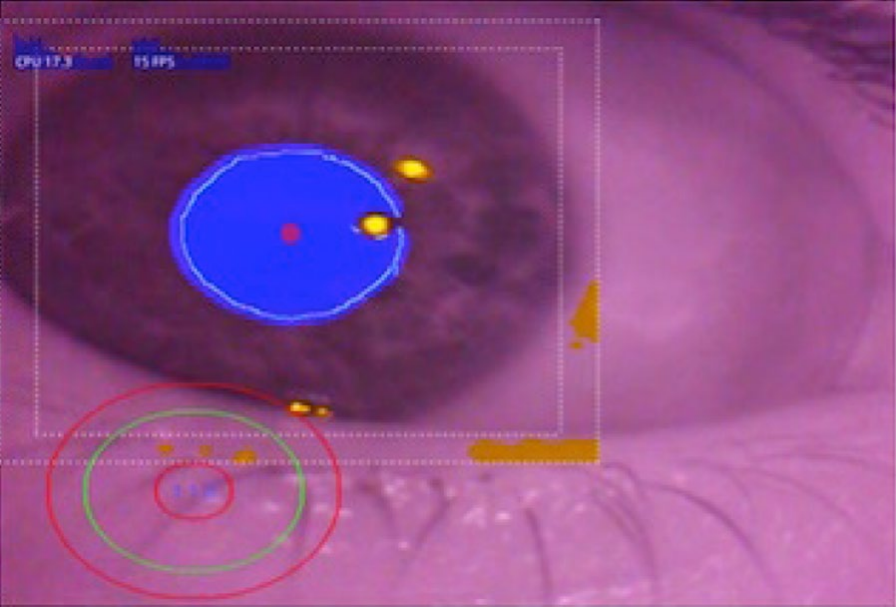
See text for description

**Conclusion** Ergonomics evaluation is mandatory when evaluating new devices in the ICU. Most ICU ventilators presented poor HMI capabilities, thus allowing the occurrence of various hazards and failure.

**Competing interests** None.

#### P111 Which of the last-generation ventilators is the most suitable for emergency transports and inter-hospital transfers?

##### Abdo Khoury^1^, Alban De Luca^2^, Thibault Desmettre^3^, Christophe Lambert^1^, Gilles Capellier^3^, Fatimata Seydou Sall^2^

###### ^1^Emergency medicine and critical care, CHU de Besançon, Besançon, France; ^2^Inserm cic 1431, CHU de Besançon, Besançon, France; ^3^Réanimation Médicale, CHU de Besançon, Besançon, France

####### **Correspondence:** Abdo Khoury - abdokhoury@hotmail.com

*Annals of Intensive Care* 2017, **7(Suppl 1)**:P111

**Introduction** Hypotension and oxygen desaturation are the most common critical events occurring in 17% of emergency transports. They are mainly related to ventilator issues and the type of ventilator might mitigate the rate of adverse events. We decided to compare the most suitable ventilators for out-of hospital transport.

**Materials and methods** This experimental bench test was designed to evaluate the technical characteristics, ergonomics, accuracy of volume and pressure delivery, triggering performance, pressurization and depressurization capacity, patient asynchrony and leak compensation of transport ventilators. We simulated many patient profiles and conditions by adjusting lung compliances (30, 100 ml cmH_2_O^−1^) and resistances (5, 20, 50 cmH_2_O L^−1^ s), inspiratory efforts (2.5, 5, 10 cmH_2_O), and leakage levels (<1, 3, 6, 12 L min^−1^). The ventilators were used in pressure control, volume control, and pressure support ventilatory modes in non-invasive and invasive settings.

**Results** Even if the technical characteristics of portable ventilators are quite similar, their ergonomics and performance are unequal. Major differences were found on tidal volume delivery, with mean relative errors ranging from +1.7 to −14.9%. Triggering delays and pressurization capacity also showed significant heterogeneity with a mean pressure time product (PTP) varying from 883 to 3018 cmH_2_O ms. The resistance of the expiratory circuit also differed across ventilators (from 0.96 to 1.82 cmH_2_O L^−1^ s), impacting the mean exhalation time and inducing air trapping and dynamic hyperinflation. Finally, while all ventilators were able to synchronize with patient’s demand at baseline (leakage <1 L min^−1^), only one showed adequate patient synchrony at high leakage level.

**Discussion** The main characteristics of last-generation ventilators are comparable, but major differences remain, especially in the conception of their basic ventilation modes. For instance, Hamilton T1 VC mode is actually a hidden pressure control mode that does not ensure tidal volume accuracy under changing lung conditions, unlike specified by international guidelines.

**Conclusion** While it is still difficult to determine which ventilator best meets clinician expectations, this study will help to select the most appropriate device in regard to his own needs and make a clear comparison between ventilator performances.

**Competing interests** Abdo Khoury reports invitations to conferences from Resmed and Air Liquide.

**References**

1. Ligtenberg JJ, Arnold LG, Stienstra Y, van der Werf TS, Meertens JH, Tulleken JE, et al. Quality of interhospital transport of critically ill patients: a prospective audit. Crit Care. 2005;9:R446.
2. Singh JM, Ferguson ND, MacDonald RD, Stewart TE, Schull MJ. Ventilation practices and critical events during transport of ventilated patients outside of hospital: a retrospective cohort study. Prehospital Emerg. Care Off. J. Natl. Assoc. EMS Phys Natl Assoc State EMS Dir. 2009;13:316–23.

#### P112 Variability of tidal volume in assisted mechanical ventilation in ARDS: a bench study

##### Sophie Perinel Ragey^1^, Loredana Baboi^1^, Claude Guérin^1^

###### ^1^Réanimation médicale, Hôpital de la Croix-Rousse, Lyon, France

####### **Correspondence:** Sophie Perinel Ragey - sophie.ragey@etu.univ-st-etienne.fr

*Annals of Intensive Care* 2017, **7(Suppl 1)**:P112

**Introduction** Even though limitingtidal volume (TV) in ARDS patients is recommended, this goal may not be achieved once spontaneous breathing comes up and assisted modes are used. Furthermore ICU ventilators offer numerous assisted ventilation modes that work differently across the brands. We underwent present bench study to systematically investigate the effect of assisted mechanical modes on a single ICU ventilator on size and variability of TV at different breathing frequencies (f), patient effort and ARDS severity.

**Materials and methods** We performed a bench study in our university laboratory on an ICU ventilator (V500 Infinity, Dräger, Germany) using ASL 5000 lung model. Compliance was set at value mimicking mild, moderate and severe ARDS as recently reported. Thirteen assisted ventilation modes were tested falling into three categories, namely volume controlled ventilation with mandatory minute ventilation (VCV-MMV), pressure-controlled ventilation (PCV) including airway pressure release ventilation (APRV) and biphasic positive airway pressure (BPAP), and pressure support ventilation (PSV). fand effort were tested each at two levels for each ARDS severity in each mode. TV was expressed as median (first-third quartiles) and compared across modes using non parametric tests. The probability for TV > 6 ml/kg ideal body weight (IBW) was assessed by binomial regression and expressed as odds ratio (OR) with 95% confidence intervals (CI). The variability of TV was measured from the coefficient of variation.

**Results** The distribution of TV over all f, effort and ARDS categories significantly differed across modes (P < 0.001, Kruskal–Wallis test). TV was significantly greater with PSV (420 mL (332- 527) than with any other mode except for the three modes accommodating a variable PS level. The risk for TV to be greater than 6 ml/kg IBW was significantly increased with spontaneous breaths assisted by PSV modes (for PSV OR 19.36; [12.37–30.65]) and significantly reduced in APRV (OR 0.44; [0.26–0.72]) and PSV with guaranteed volume mode. The risk increased with increasing effort and decreasing f. Coefficients of variation of TV were greater for low f and for VCV-MMV and PCV modes. APRV had the greatest within-mode variability.

**Conclusion** The ventilation mode had an important impact on TV in this study. The risk of TV > 6 ml/kg IBW was significantly reduced in APRV and PSV with guaranteed volume mode. APRV had the highest variability. PSV with guaranteed volume could be tested in ARDS patients.

**Competing interests** None.

#### P113 Reusable versus single use fiberscope in the ICU: a medico-economical evaluation in the ICU

##### Jean-Etienne Bazin^1^, Catherine Koffel^2^, Gilles Dhonneur^3^

###### ^1^CHU Gabriel-Montpied, Clermont-Ferrand, France; ^2^Anesthesia and intensive care medicine, Centre Hospitalier Louis Pradel, Lyon, France; ^3^Anesthesia and intensive care medicine, CHU Henri Mondor, Créteil, France

####### **Correspondence:** Gilles Dhonneur - gilles.dhonneur@aphp.fr

*Annals of Intensive Care* 2017, **7(Suppl 1)**:P113

**Introduction** Single-Use Fiberscopes (SUF) have recently become available for ICU clinical practice to overcome 3 main problems associated with Re-Usable Fiberscopes (RUF) resulting in systematic: immediate availability of a sterile device, short lag time to endoscopic treatment and removal of the cross contamination risk. However endoscopy of the upper airway and lung (EAWL) is a medical act that generates significant costs. We thus conducted a medico-economical evaluation of RUF versus SUF.

**Patients and methods** The study was conducted over 1 year (2015) in 3 French university hospitals. The medical evaluation was conducted in one of them while the cost analysis included the 3 hospitals. Medical evaluation was based upon a qualitative questionary filed by the clinician after each EAWL. Calculating the cost of RUF included the amount of money paid for the device acquisition and its equipment, and the cost related to both sanitary (systematic and post gesture) and technical maintenances. The cost of SUF was calculated based upon acquisition and the cost of disposable medical waste.

**Results** Medical evaluation performed upon 368 EAWL (60% SUF and 40 RUF) showed quality parameters either similar or better for SUF as compared to RUF except for one paramater: suction performance. Cost details of 922 EAWL were analyzed. Depending upon center organisations, cost per EAWL ranged between € 203 to € 240 for SUF, as compared to € 189 and € 269 for RUF. Saving related to SUF exclusive use would have ranged between € 2097 and € 7636 depending upon center’s settings.

**Discussion** SUF is efficient and performant for EAWL. Although costs associated with the use of SUF and RUF for ICU EAWL are close, selecting SUF induces substantial savings. The permanent and immediate availability of sterile equipment allowing to significantly shorten the lag time to treatment may have important clinical outcome for the patients.

**Conclusion** At a time when health authorities are focusing their efforts on the prevention of nosocomial infections, elimination of endoscopes-related cross-contamination risk by using SUF, especially in ICU is a major issue. Improvement of Suction capability will promote clininal superiority of SUF over RUF.

**Competing interests** Dhonneur Gilles is consultant for Ambu.

#### P114 Nociception resulting from oro-tracheal intubation: an observational study

Zina Bouzit^1^, Ahmed. H. Grati^1^, Larbi Bradai^1^, Issam Ben Ayed^1^, Fethi Aissa^1^, Hakim Haouache^1^, Nicolas Mongardon^1^, Gilles. F. Dhonneur^1^

##### ^1^Service d’Anesthésie et des Réanimations Chirurgicales, Hôpital Henri Mondor, Créteil, France

###### **Correspondence:** Zina Bouzit - zina.bouzit@gmail.com

####### *Annals of Intensive Care* 2017, **7(Suppl 1)**:P114

**Introduction** Oro-tracheal Intubation (OTI) is thought to be one of the highest nociceptive stimulation for the patient but this dogma has never been clearly proved. We tightly monitored hemodynamics (HD), nociception, and consciousness during OTI with the aim of pragmatically revisiting this dogma.

**Patients and methods** After ethic comity approval, consenting informed patients scheduled for elective cardiac or vascular surgery were included in this observational prospective study. Patients were orally premedicated (hydroxyzine) 1 h before admission into the operating room, where invasive blood pressure (BP), Bis-spectral (BIS) and Analgesia Nociception Index (ANI) monitoring were installed. The induction procedure of anesthesia with target-controlled infusion was standardized using sufentanil and propofol. Timed OTI maneuver was performed after monitored (corrugator supercillii muscle) deep neuromuscular blockade (atracurium) was installed. OTI maneuver which duration could not exceed 30 s was truncated in 3 periods (P) of predefined duration. P1 lasted 10 to 15 s and corresponded with direct laryngoscopy. P2 lasted 5 to 10 s and corresponded with tracheal tube (TT) manipulation. Finally, P3 coincided with TT cuff inflation to controlled 35 cm H_2_O lasted less than 5 s. Recorded HD, BIS and ANI data were analyzed at 6 predefined time points. T0: stable baseline values after monitors installation, T1: post induction of anesthesia and just before OTI, T2: end of P1, T3: end of P2, T4: end of P3, T5 and T6, respectively 1 and 5 min after the end of OTI. Recorded parameters evolution was compared using standard statistics.

**Results** Thirty-nine patients were included in this trial, but 4 were secondarily excluded because of OTI lasting more than 30 s. Figure 28 shows mean (SD) variations of monitored parameters: mean arterial pressure (MAP), heart rate (HR), BIS, and ANI, from T0 to T6. P1 is characterized by a remarkable stability of all measured parameters. P2 and even more P3, are associated with intense and significant HD and ANI variations. ANI variations are completely superimposed, but opposite, to those of MAP and HR. BIS variations during TI were negligible.

**Fig. 28:**
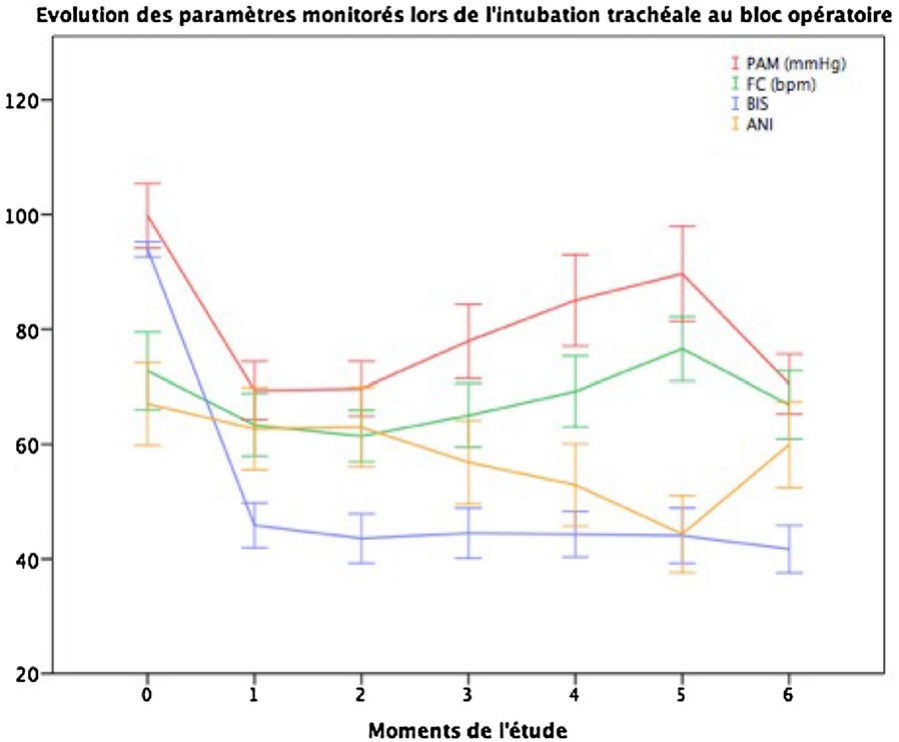
See text for description

**Conclusion** Our data does not support the dogma but rather suggests that laryngoscopy may not be the most intense nociceptive stimulus applied to patients during OTI. Both laryngeal and/or tracheal stimulation resulting from tracheal tube passage and cuff inflation seem to promote very intense nociception. Interestingly, such phenomenon should be erased by either laryngeal mucous topicalization or superior laryngeal nerve block.

**Competing interests** None.

**Reference**

1. Boselli E. Minerva Anestesiol Mars 2015;81(3):288–97.

#### P117 Comparisons and time course of mixed venous and central venous saturation are independent of severe sepsis and septic shock origin

##### Yoann Marechal^1^, Patrick Biston^1^, Michael Piagnerelli^1^

###### ^1^Reanimation polyvalente, Hôpital Civil Marie Curie, Charleroi, Belgium

####### **Correspondence:** Yoann Marechal - yoann.marechal@gmail.com

*Annals of Intensive Care* 2017, **7(Suppl 1)**:P117

**Introduction** Low (<70%) mixed venous saturation (SvO_2_) was one of the independent variable related to mortality in septic shock patients [1]. Nevertheless, values of central venous saturation (ScVO_2_) may be considered as a surrogate to SvO_2_ for early goal directed therapy in severe sepsis and septic shock. In sepsis, difference between ScVO_2_ and SVO_2_ (ΔSVO_2_) was approximately of 5% [2]. This delta SVO_2_ ignores the origin of sepsis: supra or infra-diaphragmatic where this gradient could be higher due to increased oxygen extraction by organs under diaphragm (gut, liver and kidney). We compared the correlation between ScVO_2_ and SVO_2_, the time course of delta SVO_2_ and effects of therapeutic challenge on delta SVO_2_ between patients in severe sepsis or septic shock due to supra or infra diaphragmatic infection. Enter the text.

**Patients and methods** We included patients in severe sepsis or septic shock monitored by Swan Ganz catheter and continuous ScVO_2_, hospitalized in a 24 bed medico-surgical ICU of CHU-Charleroi. Hemodynamic data and delta SVO_2_ were recorded from catheter insertion to withdrawal and before and after each therapeutic intervention (fluid challenge, dobutamine initiation or dosage modifications). Demographic data and mortality were also collected.

Graphpad prism was used for statistical analysis and graph, Bland–Altmann analysis was used and results were compared by Mann–Whitney U test when not specified. Data were expressed in median values (IQR 25–75 percentiles).

**Results** 34 patients were included which 26 presented a septic shock of upper diaphragmatic origin.

Median Apache II score was 24 (16–30). Eighteen patients (53%) out of 34 died before hospital discharge. At inclusion, mean arterial pressure, SvO_2_, cardiac index and norepinephrine infusion rate were respectively 71 (67–82) mmHg, 66 (59–72)%, 2.7 (2.2–3.4) L/min/m^2^, and 0.09 (0.00–0.50) (mcg/kg/min).

For all patients included, a total of 442 paired blood samples were obtained (321 for upper diaphragmatic group). ScvO_2_ overestimate SvO_2_ by an average of 4.96% (−6.4 to 16.3) for all paired data. No difference between upper (bias 5.24, limits −6.1 to 16.56) and under diaphragmatic (bias 4.21, limits −7.1 to 15.53) paired (ΔSvO_2_) was observed (p = 0.1).

Time course of SCVO_2_ and SVO_2_ were similar during the study period and independent of the sepsis origin.

No difference in ΔSvO_2_ was observed in relation the baseline SvO_2_ values (<50% and higher values (p = 0.11)).

Median SvO_2_ and ScVO_2_ were not statistically different before (64 [55–72] and 70 [63–78]% respectively) and after intervention (67 [56–75] and 72 [65–78]% respectively (p = 0.27 for SvO_2_ and p = 0.40 for ScvO_2_)). Therefore, ΔSvO_2_ was the same before and after interventions in the 2 groups of patients (p = 0.99) (Fig. 29).

**Fig. 29:**
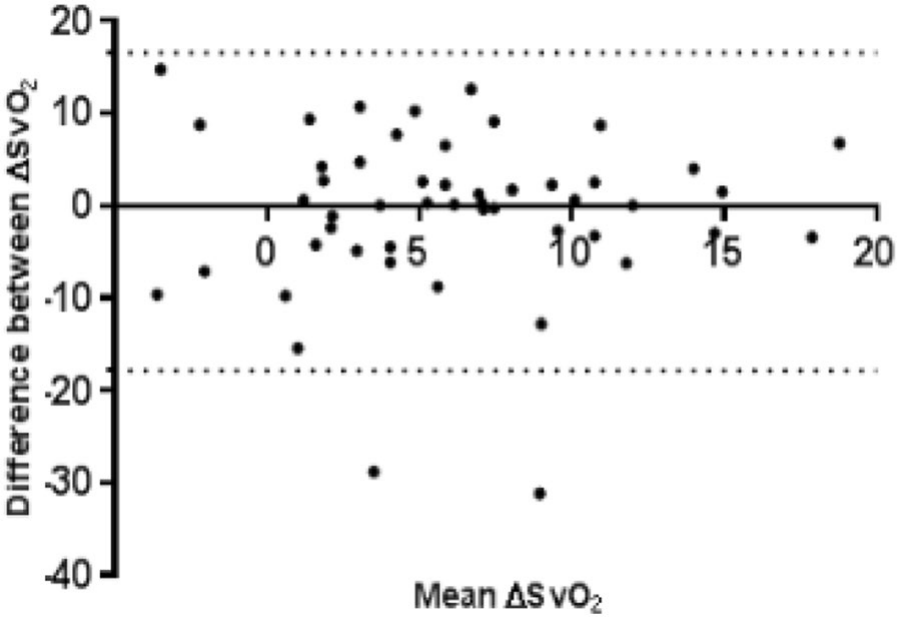
Response to therapeutic intervention Bland and Altman plot showing the agreement between ΔSvO_2_ after and before intervention (Bias −0.62%, limits of agreement from −17.83 to 16.58 (*dotted lines* on graph)

**Conclusion** As in other studies, ScvO_2_ overestimate SvO_2_ by nearly 5.0% in patients with septic shock independently of the sepsis origin. ScVO_2_ could be used as a surrogate of SVO_2_ in the management of septic patients.

**Competing interests** None.

**References**

1. Varpula M, Karlsson S, Ruokonen E, Pettilä V. Intensive Care Med. 2006 Sep; 32(9):1336–43.
2. Bloos F, Reinhart K. Intens Care Med. 2005; 31: 911–13.

#### P118 Diagnostic accuracy of the inferior vena cava collapsibility to predict fluid responsiveness in spontaneously breathing patients with cardiac arrhythmia

##### Perrine Bortolotti^1^, Delphine Colling^1^, Vincent Colas^2^, Benoit Voisin^1^, Florent Dewavrin^2^, Thierry Onimus^1^, Patrick Girardie^1^, Fabienne Saulnier^1^, Sebastien Preau^1^

###### Réanimation médicale, Centre Hospitalier Régional Universitaire de Lille, Lille, France; ^2^Réanimation Polyvalente, Centre Hospitalier De Valenciennes, Valenciennes, France

####### **Correspondence:** Perrine Bortolotti - perrine.bortolotti@gmail.com

*Annals of Intensive Care* 2017, **7(Suppl 1)**:P118

**Introduction** To investigate whether respiratory variations of the inferior vena cava diameter (cIVC) assessed during a simple standardized breathing maneuver predict fluid responsiveness in spontaneously breathing patients with septic acute circulatory failure and irregular heartbeats.

**Patients and methods** This prospective, bicentric study, was performed in the intensive care units of a general and a teaching hospital. Spontaneously breathing patients with cardiac arrhythmia considered for volume expansion for clinical signs of acute circulatory failure related to sepsis were included. Echocardiography and Doppler ultrasound were used to record the stroke volume (SV) and IVC collapsibility index (cIVC) defined as [maximum expiratory diameter (edIVC) − minimum inspiratory diameter (idIVC)]/edIVC × 100 at baseline and after a 500 ml colloid infusion. Vena cava pertinent diameters were measured 15–20 mm caudal to the hepatic vein junction and recorded by bi-dimensional imaging on a subcostal long axis view. We measured the edIVC and idIVC during standardized (st) and unstandardized (ns) breathing, and calculated cIVCst and cIVCns before fluid loading. Standardized respiratory cycles consisted of a deep standardized inspiration followed by passive exhalation. A positive response to fluid loading was defined as an increase of the SV > 10%.

**Results** Fifty-five patients were included, 29 (53%) with atrial fibrillation and 25 with atrial extrasystols >6/min. Twenty-nine (53%) patients were responders to fluid loading. A cIVCns > 19% predicts fluid responsiveness with a sensibility of 83%, a specificity of 68%, and an area under the ROC curve of 0.83 (95% CI 0.72–0.94). A cIVCst > 39% predicts fluid responsiveness with a sensibility of 93%, a specificity of 88%, and an area under the ROC curve of 0.93 [IC95: 0.86–1]. The area of uncertainty was restricted between 41 and 46%. Interestingly, idIVCst < 12 mm predicts fluid responsiveness with a sensibility of 83%, a specificity of 88%, and an area under the ROC curve of 0.93 (95% CI 0.86–1). The area of uncertainty ranged between 10 and 13 mm.

**Discussion** A simple standardized inspiratory maneuver improves the accuracy of cVCI to predict fluid responsiveness with high specificity and sensibility and a limited grey area in patients with cardiac arrhythmia. Moreover, idIVCst demonstrates very good performance to predict fluid responsiveness. This easy to acquire parameter may be more suitable for bedside patient’s management.

**Conclusion** cIVCst is a powerful index to predict fluid responsiveness in spontaneously breathing patients with sepsis and irregular heartbeats. Interestingly, idIVCst shows similar performance to predict fluid responsiveness with a much easier assessment, improving feasibility in clinical routine.

**Competing interests** None.

#### P119 Evaluation of tissue hypoperfusion parameters in acute pulmonary embolism patients admitted to the intensive care unit

##### Tomas Urbina^1^, Yann Nguyen^1^, Naïke Bigé^1^, Vincent Dubée^1^, Jeremie Joffre^1^, Gabriel Preda^2^, Jean-Luc Baudel^1^, Eric Maury^3^, Bertrand Guidet^1^, Hafid Ait-Oufella^1^

###### ^1^Réanimation médicale, Hôpital Saint-Antoine, Paris, France; ^2^Réanimation, Centre Hospitalier Général de Saint-Denis, Saint-Denis, France; ^3^Réanimation médicale, Hôpital Saint-Antoine, AP-HP, Paris, France

####### **Correspondence:** Tomas Urbina - tomasurbina75@hotmail.com

*Annals of Intensive Care* 2017, **7(Suppl 1)**:P119

**Introduction** Acute pulmonary embolism (PE) is a common condition responsible for morbidities such as chronic pulmonary hypertension and deaths. The evaluation of PE severity is mainly based on arterial blood pressure and right ventricular abnormalities. According to recent ESC guidelines, patients with high risk of death had systolic hypotension and intermediate-high risk patients had right ventricular injury (defined by biological and echocardiographic alterations). However, the predictive value of tissue perfusion remains unknown. We aimed to investigate the relationship between 28-day mortality and tissue hypoperfusion parameters on patients admitted to intensive care units (ICU) for symptomatic acute PE.

**Patients and methods** We conducted a retrospective observational study in a 18-bed ICU in a tertiary teaching hospital. From January 1993 to December 2015, all consecutive patients older than 18 years admitted for acute EP were included. Patients were identified by querying the electronic health records with the following keywords (in French): “pulmonary embolism”, “anticoagulant”, “deep venous thrombosis”, “cardiac arrest”, “heparin”, “vitamin K antagonists” and “cava filter”.

General characteristics of the patients were recorded: demographics, biological data, diagnoses, severity of illness evaluated by the SOFA score and SAPS II. Global hemodynamic and tissue perfusion (arterial lactate level, skin mottling and urinary output) parameters were collected. We investigate the relationship between these parameters and 28-day mortality.

**Results** Over a 22-year period, we identified 317 patients admitted to our ICU with a diagnosis of acute PE. Of these 120 patients were excluded because PE was not the main diagnosis of ICU admission and 85 patients because of missing data, leaving 112 patients for study. At admission, the SOFA score was 2 [1–6] and the SAPS II was 31 [17–43]. 31 patients (27%) were infused by vasopressors and 32 received mechanical ventilation support (28%). According to international guidelines, 49 patients (35%) were classified as high risk and 73 patients (65%) were classified as intermediate-high risk [1]. Thirty-nine patients (35%) received thrombolytic therapy. Day-28 mortality rate was 26%.

At admission, when compared to 28-day survivors, non-survivor patients had higher lactate level (4.2 ± 5.9 vs. 1.5 ± 2.9 mmol/L, Mann–Whitney test P < 0.001), more frequent mottling around the knee area (50 vs. 24%, Chi squared test P = 0.02) and a lower urinary output (during the first 24 h) (0.5 ± 0.6 vs. 0.9 ± 0.8 mL/kg/h, Mann–Whitney test P = 0.02). Interestingly, 28-day mortality increased with the number of tissue perfusion alterations defined as hyperlactatemia (>2 mmol/L), oliguria (<0.5 mL/kg/h) and mottling presence. The mortality at day 28 increased from 10% for no failure to 22% for 1 alteration, 32% for 2 alterations and finally to 58% for 3 tissue perfusion alterations (P = 0.02, Chi squared for a trend test).

**Discussion** Several studies in emergency and pneumology departments have investigated global hemodynamic parameters to predict outcome of PE patients. Low systolic blood pressure called “shock” and right ventricular dysfunction were identified as predictors of bad outcome. We were interested in the exploration of tissue perfusion because in septic shock several studies highlighted the discordance between global hemodynamic parameters and tissue perfusion. Moreover, during severe infections, hypoperfusion markers such as lactatemia, mottling or oliguria have been identified as predictive of ICU mortality. Recently, Vanni et al. [2] suggested lactate could be useful to identify normotensive PE patients at high risk of early death.

Here, we found that, at admission, non-survivors patients at day-28 had more severe tissue hypoperfusion than non-survivors. In addition, mortality increased with the number of tissue hypoperfusion parameters. These results have to be confirmed prospectively in a large cohort of PE patients but suggest that tissue hypoperfusion could be helpful for triage and potentially for guiding treatment.

**Conclusion** In a retrospective study, we observed an association between tissue hypoperfusion parameters and 28-day outcome in ICU patients admitted for acute symptomatic PE.

**Competing interests** None.

**References**

1. Meyer G, et al. Fibrinolysis for patients with intermediate-risk pulmonary embolism. N Engl J Med. 2014;370(15):1402–11.
2. Vanni S, et al. Short-term clinical outcome of normotensive patients with acute PE and high plasma lactate. Thorax. 2015;70(4):333–8.

#### P120 Impact of early weight gain on patient outcome in the Intensive Care Unit

##### Anne-Lise Druoton^1^, Marc Soudant^2^, Damien Barraud^1^, Jérémie Lemarie^1^, Marie Conrad^1^, Aurélie Cravoisy-Popovic^1^, Lionel Nace^1^, Sébastien Gibot^1^, Pierre-Edouard Bollaert^1^

###### ^1^Réanimation médicale, hôpital central, C.H.U. de Nancy, Nancy, France; ^2^Cic 1433 épidémiologie et clinique, C.H.U. de Nancy, Nancy, France

####### **Correspondence:** Pierre-Edouard Bollaert - pe.bollaert@chu-nancy.fr

*Annals of Intensive Care* 2017, **7(Suppl 1)**:P120

**Introduction** The administration of intravenous fluids is a key concern in the ICU. Fluids are used in many cases for renal protection, dilution of medications and as “maintenance” fluids. Moreover, they are considered as the cornerstone in resuscitation of patients with shock states [1]. Accordingly, large amounts of fluids are often administered with a resulting positive fluid balance and fluid overload.

Currently, several studies have identified a relationship between fluid overload and mortality [2]. Most of them have estimated fluid overload by calculating the cumulated fluid balance. This calculation is subject to numerous inaccuracies. Another method is daily measurement of body weight. Few studies demonstrated the link between ICU mortality and early weight gain [2].

The aim of this study was to evaluate the association between early weight gain and ICU outcome.

**Patients and methods** All patients admitted from the 1st January 2012 to 31th December 2013 in a medical ICU of a University Hospital for which a weight was recorded on admission (Day 0) and at Day 3 were included. Demographic data, comorbidities, SOFA score, cause of ICU admission, length of stay and mortality were recorded. Weight changes were calculated by subtracting body weight as measured on third day from one as measured on admission.

Values are expressed as mean or median [min–max]. Univariate and multivariate linear or logistic regression analyses were performed to evaluate factors associated with clinical outcomes.

**Results** Two hundred and thirty-eight patients (mean age 64 years; 61% male) were included. Median length of stay was 8 days [3–44]. ICU mortality rate was 12.6%. Significant differences were found between survivors and non survivors with regard to Day 0 SOFA score on admission (p < 0.005), need for mechanical ventilation (p = 0.012 and hemodialysis (p = 0.048). Median body weight gain between Day 0 and Day 3 was 1.3 kg [−5 to 10] in non-survivors and 0.8 kg [−13 to 14] in survivors (p = 0.069). Admission SOFA score (OR 1.288; 95% CI 1.103–1.503; p = 0.001), PaO_2_/FiO_2_ ratio on admission (OR 1.006; 95% CI 1.002–1.011; p = 0.002) and weight gain between Day 0 and Day 3 (OR 1.123; 95% CI 1.001–1.261; p = 0.047) were independent mortality factors in multivariate analysis. Body weight gain (>1 kg day 0 to 3) was also correlated with a longer stay in the ICU (OR 1.39; 95% CI 1.15–1.68; p = 0.0008).

**Conclusion** This study is one of the few to establish that early weight gain is an independent risk factor for mortality in ICU patients. The body weight, though subject to difficulties and possible errors in measurement is an indirect marker of fluid overload. It remains to establish if the control of fluid overload based on an early weight-gain control strategy is likely to improve patients outcome.

**Competing interests** None.

**References**

1. Malbrain M et al. Fluid overload, de-resuscitation, and outcomes in critically ill or injured patients: a systematic review with suggestions for clinical practice. Anaesthesiol Intensive Ther. 2014;46(5):361–80.
2. You JW et al. Association between weight change and clinical outcomes in critically ill patients. J Crit Care. 2013;28(6):923–7.

#### P122 PEEP sparing in prone position has a weak but significant positive effect on cardiac index in ARDS patients

##### Ruste Martin^1^, Laurent Bitker^1^, Hodane Yonis^1^, Mylène Aublanc^1^, Sophie Perinel-Ragey^1^, Aurore Louf-Durier^1^, Floriane Lissonde^1^, Claude Guérin^1^, Jean-Christophe Richard^1^

###### ^1^Réanimation médicale, Hôpital de la Croix-Rousse, Lyon, France

####### **Correspondence:** Jean-Christophe Richard - j-christophe.richard@chu-lyon.fr

*Annals of Intensive Care* 2017, **7(Suppl 1)**:P122

**Introduction** The PROSEVA study was the first randomized controlled study having shown a positive effect of prone position (PP) on ARDS mortality [1]. Unlike previous randomized controlled trial, PEEP was adjusted with a PEEP-FIO_2_ table, whose consequence was a small but significant decrease in the PEEP level. The aim of this study was to investigate the effect of PEEP variation during prone position on cardiac index in ARDS patients.

**Patients and methods** Single center retrospective observational study performed on ARDS patients hospitalized in a medical ICU between July 2012 and March 2016. Patients included were adults fulfilling the Berlin definition for ARDS, undergoing at least one prone position session, under hemodynamic monitoring by the Picco ^®^ device, with availability of hemodynamic measurements performed before (T1), at the end (T2), and after the prone position session (T3). Prone position sessions were excluded if they were performed >7 days after ARDS onset. The following variables were recorded: demographic, SAPSII, ARDS severity and risk factor, SOFA score and cumulative fluid balance at PP onset, delay between ARDS session and PP session, hemodynamic, arterial blood gas, ventilatory settings, plateau pressure, catecholamine dose and additional treatments. Statistical analyses were performed using prone position session as statistical unit and mixed models taking into account both multiple prone position sessions by patient and multiple measurements during a prone position session. Variables associated with cardiac index with a p value below 0.1 in univariate analysis were selected for inclusion in a multivariable mixed logistic regression model, using backward stepwise descending selection. p < 0.05 was chosen for statistical significance. Data are expressed as mean ± standard deviation.

**Results** 85 patients fulfilled the inclusion criteria over the study period, totalizing 149 prone position sessions (2 ± 1 sessions per patient). PEEP level decreased significantly from 9 ± 3 to 8 ± 2 cm H_2_O between T1 and T2. PEEP decreased by at least 5 cm of H_2_O in 18 (12%) of the PP sessions. Multivariate analysis identified 7 variables independently associated with cardiac index (model 1, cf. Table 22). Multivariate analysis also identified measurement time, age, sex, PEEP and pH as being independently related to global end-diastolic volume. After removing the effect of global end-diastolic volume by forcing it out of the model, PEEP became significantly related to cardiac index (model 2, cf. Table 22), with a coefficient β suggesting a marginal effect on cardiac index (−0.03 l min^−1^ m^−2^ per 1 cm H_2_O increase of PEEP).

**Table 22:** Multivariate analyses of variables associated with cardiac index

	Model 1	Model 1	Model 2	Model 2
	β	p	β	p
Age	−0.043	<0.001	−0.021	<0.05
SOFA score	0.071	<0.001	0.103	<0.001
Neuromuscular blocking agents (ref = no)	0.450	<0.001	0.336	<0.05
Global end-diastolic volume (mL m^−2^)	0.003	<0.001	–	–
Dobutamine dose (µg kg min^−1^)	−0.021	<0.01	−0.023	<0.01
PaCO_2_ (mmHg)	0.014	<0.01	0.013	<0.05
PaO_2_/FiO_2_ (mmHg)	−0.002	<0.001	−0.001	<0.05
PEEP (cm H_2_O)	NS	NS	−0.030	<0.05
Time between ARDS onset and PP session	NS	NS	−0.044	<0.05
Renal replacement therapy (ref = no)	NS	NS	−0.355	<0.05
Measurement time [ref = before PP (T1)]	NS	NS		<0.001
End of PP (T2)			0	
After PP (T3)			−0.204	

**Conclusion** PEEP decrease during prone position as a consequence of improvement of oxygenation has a marginal but significant positive effect on cardiac index, related to an increase in global end-diastolic volume and probably venous return.

**Competing interests** None.

#### P123 Impact of implementing a sodium phenyl-acetate and sodium benzoate delivery protocol on hyperammonaemia

##### David Brossier^1^, Isabelle Goyer^1^, Christopher Marquis^1^, Philippe Jouvet^1^

###### ^1^Soins intensifs pédiatriques, CHU Sainte-Justine, Montréal, Canada

####### **Correspondence:** David Brossier - david_brossier@yahoo.fr

*Annals of Intensive Care* 2017, **7(Suppl 1)**:P123

**Introduction** Hyperammonaemia is defined as ammonaemia above 80 µmol/L when child is under 1 month old and 55 µmol/L when over 1 month old. Hyperammonaemia over 200 µmol/L was proved to be an independent factor of mortality. The recommended treatment for hyperammonaemia due to inherited error of metabolism is based on the association of sodium phenyl-acetate and sodium benzoate called Ammonul^®^. In order to improve management of hyperammonaemia in pediatric intensive care unit (PICU), an Ammonul^®^ delivery protocol was implemented since the 30th August 2008. The purpose of our study was to evaluate the impact of this delivery protocol on morbidity and mortality for patient admitted in PICU for hyperammonaemia.

**Patients and methods** A retrospective study was conducted from January 1st 2000 to May 31st 2016 in the PICU of the CHU Sainte Justine. Every patient with hyperammonaemia receiving Ammonul^®^ and admitted in the PICU was included. Patients under 18 years old or for whom decision to withhold or withdraw treatment was taken, or brain dead at admission, or if treatment was started in another hospital were excluded. Patients were allocated in two groups, without or with, depending on if they were treated after or before the implementation of the protocol.

**Results** Ammonul^®^ was prescribed for 32 patients during the study period, 4 of them weren’t admitted in PICU. Among the remaining 28, 6 were excluded, especially for treatment timing reasons. Finally, 22 patients were studied, 8 in the without protocol group and 14 in the with protocol group. Patients were aged 9 month [0–452] in the without group and 238 [0–512] in the with group (p = 0.09). There was no difference between groups in term of weight (6 [3–43] vs 24 [3–67]), ammonaemia level (328 [75–2022] vs 239 [150–830]) and severity score (PIMII 18 [5–54] vs 19 [2–100]). The principal cause of hyperammonaemia was inherited error of metabolism in both groups (6 (75%) vs 9 (64%)). The median delay between diagnosis and Ammonul prescription was 100 min [75–209] in the without group and 47 min [0–81] in the with group (p < 0.001), the median delay between diagnosis and Ammonul administration was 173 min [95–339] in the without group and 173 min [73–276] in the with group (p = 0.63). The mortality during hospitalisation was 50% (4) in the without group and 35% (5) in the with group (p = 0.34). The median PICU stay was 104 [26–469] in the without group and 75.5 h [12–1305] in the with group (p = 0.73). There was no difference concerning liver transplant, ventilation and renal replacement therapy between groups.

**Discussion** This study is one the most important series of paediatric patients treated with Ammonul^®^. Our study succeeded to show improvement in delay between diagnosis and prescription, but failed to show any improvement in morbi-mortality. Further analysis must be perform. One of the explanation of this lack of improvement would be the absence of difference between groups concerning delay between diagnosis and treatment administration.

**Conclusion** Our study showed that the implementation of a sodium phenyl-acetate and sodium benzoate delivery protocol shorten the delay between diagnosis of hyperammonaemia and treatment prescription but failed to find any improvement in morbi-mortality.

**Competing interests** None.

#### P124 Patients characteristic of French pediatric intermediate care units

##### Marie Lampin^1^, Alain Duhamel^2^, Hélène Béhal^3^, Tahar Dhaoui^4^, Véronique Godeffroy^5^, Guillaume Pouessel^6^, Eve Devouge^7^, Dominique Evrard^8^, Florence Delepoulle^9^, Sylvie Racoussot^10^, Bruno Grandbastien^11^, Francis Leclerc^1^, Stéphane Leteurtre^1^

###### ^1^Réanimation pédiatrique, CHRU de Lille, Lille, France; ^2^Santé publique : épidémiologie et qualité des soins, service de biostatistiques, CHRU de Lille, Lille, France; ^3^Biostatistiques, C.H. Régional Universitaire de Lille (CHRU de Lille), Lille, France; ^4^Pédiatrie, C.H. de Cambrai, Cambrai, France; ^5^Pédiatrie, C.H. de Lens, Lens, France; ^6^Pédiatrie, C.H. de Roubaix, Roubaix, France; ^7^Pédiatrie, C.H. d ‘Arras, Arras, France; ^8^Pédiatrie, C.H. de Boulogne-sur-Mer, Boulogne-sur-Mer, France; ^9^Pédiatrie, C.H. de Dunkerque, Dunkerque, France; ^10^Pédiatrie, C.H. de Douai, Douai, France; ^11^Santé publique : épidémiologie et qualité des soins, service de gestion du risque infectieux, de, CHRU de Lille, Lille, France

####### **Correspondence:** Marie Lampin - marie-emilie.lampin@chru-lille.fr

*Annals of Intensive Care* 2017, **7(Suppl 1)**:P124

**Introduction** In France, since 2006, legislation for pediatric critical care organization approved the establishment of pediatric intermediate care units, between pediatric wards and pediatric intensive care units. In pediatric intermediate units, children require close monitoring and/or continuous monitoring due to potential organ failure, without requiring resuscitation. The aim of this study is to describe patients characteristics of French pediatric intermediate care units.

**Patients and methods** We performed a prospective observational cohort study in pediatric intermediate care units of seven regional hospitals in northern of France. All consecutive patients under 18 years, admitted in these seven pediatric intermediate care units were included (September 2012–January 2014).

**Results** Among 2909 consecutive screened patients, 2868 patients were included. Sex ratio was 1.26. Median of age was 29[5–103] months. Median of length of stay was 1 [1–3] day. Thirty-three percent had comorbidities. Seventy per cent were transferred from emergency unit. The type of stay was medical for 95%. The primary reason for admission was essentially respiratory (44%) and neurologic (22%). Infection was the main cause of respiratory failure (79%). The destination was pediatric ward in 53% and home in 36%. Three per cent were transferred in pediatric intensive care unit and one patient deceased.

**Conclusion** The patients hospitalized in pediatric intermediate care unit were young. The length of stay in these units was short and the respiratory failure was more frequent.

**Competing interests** None.

#### P125 Validation of three pediatric early warning scores in seven French pediatric intermediate care units

##### Marie Lampin^1^, Alain Duhamel^2^, Hélène Béhal^3^, Tahar Dhaoui^4^, Véronique Godeffroy^5^, Guillaume Pouessel^6^, Eve Devouge^7^, Dominique Evrard^8^, Florence Delepoulle^9^, Sylvie Racoussot^10^, Bruno Grandbastien^11^, Francis Leclerc^1^, Stéphane Leteurtre^1^

###### ^1^Réanimation pédiatrique, CHRU de Lille, Lille, France; ^2^Santé publique : épidémiologie et qualité des soins, service de biostatistiques, CHRU de Lille, Lille, France; ^3^Biostatistiques, CHRU de Lille, Lille, France; ^4^Pédiatrie, CH de Cambrai, Cambrai, France; ^5^Pédiatrie, CH de Lens, Lens, France; ^6^Pédiatrie, CH de Roubaix, Roubaix, France; ^7^Pédiatrie, C.H. d ‘Arras, Arras, France; ^8^Pédiatrie, C.H. de Boulogne-sur-Mer, Boulogne-sur-Mer, France; ^9^Pédiatrie, C.H. de Dunkerque, Dunkerque, France; ^10^Pédiatrie, C.H. de Douai, Douai, France; ^11^Santé publique : épidémiologie et qualité des soins, service de gestion du risque infectieux, de, CHRU de Lille, Lille, France

####### **Correspondence:** Marie Lampin - marie-emilie.lampin@chru-lille.fr

*Annals of Intensive Care* 2017, **7(Suppl 1)**:P125

**Introduction** Pediatric Early warning systems were created to quantify severity of illness across in hospitalized children in pediatric ward or emergency unit. Pediatric intermediate care units are alternative structures for moderate ill children. The aim of this study was to assess the validity of three pediatric early warning scores in pediatric intermediate care units.

**Patients and methods** We did a prospective, observational, multicenter cohort study in seven French regional hospitals (09/2012–01/2014). All consecutive children <18 years were included. Three scores (PAWS, PEWS, and BedPEWS) were calculated each 8 h and more if deterioration. Binary outcome criteria were “medical call”, and “admission to Pediatric intensive care Unit (PICU)”. When one or two monitoring parameters necessary to calculate the score were missing, the score was still calculated and the missing value was considered normal and these scores were called “imputed scores”. We used areas under the ROC curve (AUC) to estimate discrimination.

**Results** 2868 children were included for a total of 19,071 observations for calculating the three scores. Median scores for the three scores were respectively 2, 2 and 1. Medical call was observed in 11% (n = 2056), and admission to PICU in 0.45% (n = 85). AUCs calculated for the three scores for predicting medical call were ranged from 0.92 to 0.93. AUCs for predicting PICU admission were ranged from 0.89 to 0.92 (Table 23).

**Table 23:** Discrimination of three pediatric early warning systems for predicting medical call or admission to PICU

Scores	Medical call		Admission to PICU	
	AUC	OR [95% CI]	AUC	OR [95% CI]
Complete PAWS	0.93	1.44 [1.38–1.51]	0.90	2.37 [1.97–2.85]
Imputed PAWS	0.93	1.34 [1.28–1.39]	0.91	2.44 [2.08–2.87]
Complete PEWS	0.92	1.67 [1.57–1.78]	0.91	2.82 [2.27–3.49]
Imputed PEWS	0.93	1.55 [1.47–1.63]	0.89	2.73 [2.25–3.30]
Complete BedPEWS	0.92	1.29 [1.25–1.33]	0.92	1.84 [1.60–2.11]
Imputed BedPEWS	0.93	1.29 [1.25–1.33]	0.92	1.85 [1.66–2.06]

*AUC* area under the curve, *OR* odds ratio to increase score one point, *CI* confidence intervals

**Conclusion** The three Pediatric Early warning scores, developed from pediatric ward and emergency department, can be used to detect deterioration requiring a medical intervention or PICU admission in hospitalized children in pediatric intermediate care unit.

**Competing interests** None.

**References**

1. Monaghan A. Detecting and managing deterioration in children. Paediatr Nurs. 2005;17:32–5.
2. Egdell P, Finlay L, Pedley DK. The PAWS score: validation of an early warning scoring system for the initial assessment of children in the emergency department. Emerg Med J. 2008;25:745–9.

#### P126 Breastfeeding disruption during hospitalisation for acute bronchiolitis in children: determining factors in a French teaching hospital

##### Claire Heilbronner^1^, Emeline Roy^2^, Alexandra Masson^1^, Alice Hadchouel-Duvergé^3^, Sylvain Renolleau^4^, Virginie Rigourd^5^

###### ^1^Service de réanimation et surveillance continue médico-chirurgicale pédiatrique, Hôpital Necker - Enfants Malades, Paris, France; ^2^Service de pédiatrie générale, Hôpital Necker - Enfants Malades, Paris, France; ^3^Pneumology, Hospital Necker, Paris, France; ^4^Réanimation pédiatrique polyvalente, Hôpital Necker - Enfants Malades, Paris, France; ^5^Réanimation néonatale, Hôpital Necker - Enfants Malades, Paris, France

####### **Correspondence:** Claire Heilbronner - claire.heilbronner@aphp.fr

*Annals of Intensive Care* 2017, **7(Suppl 1)**:P126

**Introduction** No study have been published to specifically evaluate or measure breastfeeding disruption during hospitalisation for bronchiolitis although the high number of hospitalisation for bronchiolitis in children potentially breastfed makes it an important health issue. We conducted a pilot study to try to measure that risk.

**Patients and methods** This is a single center prospective observational study conducted between the 1st of October 2015 and the 15th of February 2016 in a tertiary care teaching hospital. All patients under 6 month hospitalized with acute bronchiolitis and receiving at least partial breastfeeding were considered for study. Patients discharged at home whose parents accepted to be contacted by phone were prospectively included. The primary outcome was unwanted weaning from breastfeeding. The secondary outcome was trying to identify risk factors for weaning. Data are expressed as median values (with minimum and maximum value) for continuous variables, and number and/or frequency (%) for binary or categorical data.

**Results** During the study period 144 breastfed patients under 6 month were hospitalised at our hospital for bronchiolitis, 84 patients (58%) could be included in the study. Length of hospital stay was 3 days [1; 34] and 27 patients (32%) patients spent some time in PICU. One patient (1.2%) only needed invasive ventilation for 10 days, 18 (21%) received either NIV or high flow oxygen for 3 days [1;9], 34 patients (40%) received standard oxygen for 3 days [1; 7] and 34 patients (40%) received no respiratory support. Forty-five (53%) patients received nutritional support, either enteraly (N = 38, 45%) or parenteraly (N = 5, 6%) or both (N = 2, 2.4%). Most breastfeeding mothers (96%) did not smoke and lived as a couple (98%), 2% of mothers were working at the time of hospitalisation. Sixty-five patients (77%) were exclusively breastfed before hospitalisation. The median delay for phone contact was 3 month [0.5; 6]. Forty-three mothers (51%) stated that their breastfeeding had been modified by the hospitalisation of their child: 17 stopped breastfeeding (Group 1), 12 switched from total to partial breastfeeding (Group 2) and 14 reduced breasfeeding without stopping (Group 3). Remaining mothers (Group 4) (41, 49%) stated to have kept breastfeeding as before or that their breastfeeding modification was personal choice. Patients whose breastfeeding was more impacted had a tendency to being younger with a median age of 34 days [3;166] vs 50 days [16;159] on admission, p 0.06) and to be exclusively breastfed before (81 vs 73%, NS). No difference was observed for birth weight, gestational age, daily growth before stay, length of stay, need for respiratory support, need for nutritional support, need for PICU admission between groups 1 + 2 + 3 and group 4. Mothers stated first cause of breastfeeding disturbance had been lack of support and advices (32.5%) followed with some logistics problem (difficulties to draw breast milk, room accommodation, introduction of formula milk) in 15.7% cases and severity of child’s respiratory disease in 16.8% cases.

**Discussion** This is the first study to highlight the effect of hospitalization for bronchiolitis on breastfeeding, and to question the various factors involved. Our study is subject to possible bias (single centered, long delay between hospitalisation and questionnaire) but we still achieved to reach a big number of patients (84 patients in one epidemic season). We observed a very high rate of breastfeeding disturbance during hospitalisation for bronchiolitis in our hospital (over 50%). We expected to find severity of respiratory disease as the first risk factor but only 16.8% of mothers pointed out severity of disease as a reason for unwanted weaning and respiratory distress on medical charts was also not significantly different. Lack of support from caregivers was the first factor pointed out. It is a modifiable factor for further practice, especially in younger infants for witch breastfeeding has just begun and is still precarious.

**Conclusion** Bronchiolitis is a high risk event for breastfeeding with about half of mothers in our hospital either stopping or diminishing their breastfeeding during hospitalisation. Correct advice and support could be a determining factor of breastfeeding’s continuation and further studies should focus on interventions to prevent unwanted weaning.

**Competing interests** None.

#### P127 Factors associated with severe bronchiolitis in PICU: a retrospective study

##### Elise Delacroix^1^, Isabelle Wroblewski^2^, Isabelle Pin^1^, Anne Ego^1^, Valerie Payen^1^, Thierry Debillon^2^, Anne Millet^1^

###### ^1^Isère, Hôpital couple enfant, Grenoble, France; ^2^Reanimation pediatrique, C.h.u., La Tronche, France

####### **Correspondence:** Anne Millet - amillet@chu-grenoble.fr

*Annals of Intensive Care* 2017, **7(Suppl 1)**:P127

**Introduction** The explosion of non invasive ventilation (NIV) and high frequency canula therapy (HFNCT) in bronchiolitis for the last 10 years led to a decreased rate of intubation [1]. There are lacking data concerning the clinical and biological factors associated with severe bronchiolitis defined by the need for an intensive level of NIV or for invasive ventilation (IV).

**Patients and methods** Two hundred and fifty-two patients aged of less than 6 months were included in a retrospective study conducted over a three winter period from 2013 to 2016 in a 16 bed French PICU. We aimed to compare patients hospitalized for severe bronchiolitis, defined by the need for an intensive level of NIV or for invasive ventilation (IV) to non severe patients that were treated with nasal continuous positive airway pressure (nCPAP) or HFNCT. We secondly analyzed the evolution of patients characteristics over a three winter period and factors associated with HFNCT failure. Clinical and biological factors were recorded at the admission of the patients and 4 h after (H4).

**Results** One hundred patients were included in the non severe bronchiolitis group and 152 in the severe group. Factors significantly associated with severe bronchiolitis were a young age, a small weight and the presence of apneas at the admission. The Wang severity score, the heart rate, the apH and the PaCO_2_ were significantly different between the two groups at the admission and at H4. The level of oxygenation at H4 were also higher in the non severe group. The rate of bacterial coinfection, the length of stay in PICU and of ventilation were higher in the severe group. Over the 3 years period, the rate of IV and NIV bilevel positive airway pressure significantly decreased in parallel with the increased utilization of HFNCT. Comparing patients responders or not to HFNCT, the weight, the Wang score, the respiratory rate, the apH and the PaCO_2_ at H4 were significantly different.

**Discussion** First, we found that clinical and biological factors associated with the need for a NIV bi level positive airway pressure were similar of those associated with the need for IV previously described [2]. We can hypothesize that patients at risk of severe bronchiolitis and NIV failure are actually better identified with prompt modification of ventilation parameters and a greater tolerance for permissive hypercapnia under NIV support. All of these factors lead to a reduction of the rate of intubation [1]. Secondly, patients hospitalized seemed to be less severe according to biological parameters (apH and PaCO_2_) and the decreased need for IV all along the three seasons studied. This could be in relation with an earlier recognition of critically illness patients and no delayed respiratory support treatment. This could also be related to an increased administration of HFNCT to patients usually treated with standard oxygen treatment. Finally, we reported factors associated with HFNCT failure. Even if HFNCT is not actually recommended in the first line treatment of bronchiolitis, some authors described its efficiency in this pathology, with similar results as nCPAP and superiority to standard nasal oxygen with reduced respiratory rate, respiratory work, length of stay and level of oxygenation.

**Conclusion** NIV is a useful respiratory support technique in paediatric patients with bronchiolitis. Younger and heavier patients, clinical severity score, respiratory and heart rate or biological parameters like apH and PaCO_2_ are factors associated with an intensive level of NIV.

**Competing interests** None.

**References**

1. Mayordomo-Colunga J, Medina A, Rey C, Díaz JJ, Concha A, Los Arcos M, Menéndez S. Predictive factors of non invasive ventilation failure in critically ill children: a prospective epidemiological study. Intensive Care Med. 2009;35(3):527–36.
2. Ganu SS, Gautam A, Wilkins B, Egan J. Increase in use of non-invasive ventilation for infants with severe bronchiolitis is associated with decline in intubation rates over a decade. Intensive Care Med. 2012;38(7):1177–83.

#### P128 Practice survey on prone positioning in French-speaking pediatric intensive care units (nursing part)

##### Julien Denot^1^, Véronique Berthelot^1^, Emilie Thueux^1^, Marie Reymond^1^, Alexandra De Larrard^1^, Alain Amblard^1^, Jérôme Rambaud^1^, Pierre-Louis Leger^1^

###### ^1^Réanimation néonatale et pédiatrique, Hopital pour enfants Trousseau, Paris, France

####### **Correspondence:** Pierre-Louis Leger - leger.pierrelouis@gmail.com

*Annals of Intensive Care* 2017, **7(Suppl 1)**:P128

**Introduction** The prone positioning (PP) is a strategy widely used in the treatment of severe forms of acute respiratory distress syndrome (ARDS) in adults. Its early use significantly reduces mortality [1]. However, the studies do not strongly demonstrate its prognostic impact in pediatric ARDS. The aim of this study was to describe the prone positioning practices in the French-speaking pediatric intensive care units (PICU).

**Patients and methods** This survey was conducted by email questionnaire to pediatric intensivists belonging to the French Society of Intensive Care Medicine and the French-speaking Group of Pediatric Intensive Care and Emergency Medicine. It was conducted From February to May 2016. The survey was addressed to doctors, nurses, physiotherapists practicing in PICU. It included 29 questions about indications, contraindications, techniques and medical devices used, and complications.

**Results** One hundred and three persons answered (69 doctors and 33 nurses) which work in 28 French hospitals and 1 Canadian hospital. The thoraco-abdominal support is use whatever the age (neonates, infants, children) and frequently made with non-specific material: sheets and undersheets (64%), cocoons with balls (44%), pillows and bolsters (38%). The members are placed in flexion or half-flexion (71% of interviewed persons) without systematic changes of the positions. The PP is frequently stopped during the dressings (72% of interviewed persons), less frequently to achieve a toilet (44%), radiography (43%) or physiotherapy session (29%). Seventy-six percent of interviewed persons say that the kind and location of prostheses (catheter, drains, stomy) are not contraindications to the PP. Concerning respiratory care 74% of interviewed persons frequently use a closed system for tracheal aspirations. Moreover, 45% of interviewed persons say that respiratory physiotherapy sessions are not classically realized in PP periods. Concerning the enteral nutrition 59% of persons do not change their practices during PP and only 10% reduce or stop it. Finally the mucocutaneous complications are the most frequent (54% of interviewed persons) just before the displacement of protheses (37%). The prevention of cutaneous pressure ulcers by the use of specific mattress is widespread (61% of interviewed persons) but no supplemental care in prone compared to supine positioning.

**Discussion** The survey aimed to describe the techniques and modalities of PP in children, as well as several aspects of care associated with PP. The results show that the quality of care seems unaffected by the PP. The interviewed persons do not reported technical contraindication of PP. However, a special attention should be put on hemodynamic instability, tracheostomy or umbilical venous catheter. The survey reported the same complications in children and adult [2]. Finally, we also report the absence of paramedical care protocol for the PP. Future clinical studies will assess the impact of nursing protocols to limit complications, improve quality and comfort of care in children during PP periods.

**Conclusion** The prone positioning in French-speaking pediatric intensive care units is not very well protocolized. Nevertheless, the care not seems to be significantly impacted by the prone positioning. Future research should focus on the evaluation of prone positioning nursing protocols.

**Competing interests** None.

**References**

1. Guerin C, Reignier J, Richard JC, Beuret P, Gacouin A, Boulain T, et al. Prone positioning in severe acute respiratory distress syndrome. N Engl J Med. 368(23):2159–68.
2. Koulouras V, Papathanakos G, Papathanasiou A, Nakos G. Efficacy of prone position in acute respiratory distress syndrome patients: A pathophysiology-based review. World J Crit Care Med. 5(2):121–36.

#### P129 Implementation of a weaning protocol of mechanical ventilation in a pediatric intensive care unit in Oran, Algeria

##### Nabil Tabet Aoul^1^, Ali Douah^2^, Zakaria Addou^3^, Houari Youbi^2^, Mohamed Moussati^2^, Kamel Belhabiche^2^, Souad Mir^1^, Sanaa Abada^1^, Zerhouni Amel^4^, Nabil Aouffen^2^

###### ^1^Réanimation pédiatrique canastel, Faculté de médecine d’Oran, Oran, Algeria; ^2^Anesthésie réanimation pédiatrique, Etablissement hospitalier spécialisé en pédiatrie Canastel, Oran, Algeria; ^3^Réanimation pédiatrique de Canastel d’oran, Departement de medecine d’Oran Algerie, Oran, Algeria; ^4^Réanimation pédiatrique, EHS CANASTEL, ORAN, Algeria

####### **Correspondence:** Nabil Tabet Aoul - tabetrea@yahoo.fr

*Annals of Intensive Care* 2017, **7(Suppl 1)**:P129

**Introduction** The Mechanical ventilation (MV) is the main technical motivating ICU admission and involves on average 30% of children (range 20–60%), half is extubated within 48 h. Weaning from mechanical ventilation, should be as early as possible in order to reduce the risk of iatrogenic complications, hospital stay in intensive care and costs.

Our purpose is the implementation of a written protocol of weaning from mechanical ventilation in a pediatric intensive care unit.

**Patients and methods** This is a prospective study including all children aged between 1 month and 15 years, admitted to the pediatric intensive care unit at Canastel Hospital in Oran Algeria during the period from 1 March 2015 to 31 August 2016.

The weaning protocol involves the systematic and daily search for 7 criteria for sevrabilité allowing extubation:

- Resolution of the case
- FiO_2_ < 50% or SaO_2_ > 90% and ≤5 cm H_2_O PEEP
- Dobutamine or noradrenaline ≤ 5 ≤ 0.2/kg/min
- Conscious or slightly sedated or Glasgow >12
- Cough, effective deglutition
- No surgery <12 h
- T < 38.5°C

**Results** We included 43 children. The average age is 6 years (1 month–14 years), sex ratio is 1.2. The most common reason for admission in ICU was the severe brain injury in 21.4% and the status epilepticus 19%. The patients were intubated in 70.7% of cases for an alteration of the state of consciousness. Ten patients (25.6%) had a post extubation dyspnea, 3 required reintubation within 24 h. A corticosteroids administered in 67.4% of patients in the 12 h before extubation. No deaths during the study.

**Conclusion** Weaning from mechanical ventilation is a key direction for the prognosis of ICU patients where the value of a weaning protocol with general and respiratory criteria to facilitate extubation.

**Competing interests** None.

#### P130 Severe atypical pneumonia in critically ill patients: a retrospective multicentre study

##### Valade Sandrine^1^, Lucie Biard^2^, Virginie Lemiale^1^, Laurent Argaud^3^, Frédéric Pène^4^, Laurent Papazian^5^, Fabrice Bruneel^6^, Amélie Seguin^7^, Achille Kouatchet^8^, Johanna Oziel^9^, Olivier Lesieur^10^, Florence Boissier^11^, Bruno Megarbane^12^, Naïke Bigé^13^, Noelle Brule^14^, Anne-Sophie Moreau^15^, Alexandre Lautrette^16^, Elie Azoulay^1^

###### ^1^Réanimation médicale, Hôpital Saint-Louis, Paris, France; ^2^Sbim, Assistance Publique Hôpitaux de Paris, Paris, France; ^3^Réanimation Médicale, Hospices Civils de Lyon - Groupement Hospitalier Edouard Herriot, Lyon, France; ^4^Réanimation Médicale, Hôpital Cochin, Paris, France; ^5^Service de réanimation-détresses respiratoires et infections sévères, Hôpital Nord, Marseille, France; ^6^Réanimation médico-chirurgicale, Centre Hospitalier de Versailles, Le Chesnay, France; ^7^Réanimation médicale, Centre Hospitalier Universitaire de Caen, Caen, France; ^8^Service de Réanimation médicale et Médecine hyperbare, Centre Hospitalier Universitaire d’Angers, Angers, France; ^9^Réanimation medico-chirurgicale, hopital avicenne, Bobigny, France; ^10^Réanimation, Centre Hospitalier la Rochelle, La Rochelle, France; ^11^Réanimation Médicale, Hôpital Henri Mondor, Créteil, France; ^12^Service de Réanimation Médicale et Toxicologique, CHU Lariboisière, Paris, France; ^13^réanimation médicale, Hôpital Saint-Antoine, Paris, France; ^14^Réanimation médicale, CHU, Nantes, France; ^15^Réanimation Médicale, Hôpital Saint-Louis, Assistance Publique Hôpitaux de Paris, Paris, France; ^16^ Réanimation médicale, CHU Gabriel-Montpied, Clermont-Ferrand, France

####### **Correspondence:** Virginie Lemiale - virginie.lemiale@aphp.fr

*Annals of Intensive Care* 2017, **7(Suppl 1)**:P130

**Introduction** *Chlamydia pneumoniae* (CP) and *Mycoplasma pneumoniae* (MP) are intracellular pathogens that account up to 20% of community acquired. These patients with “atypical pneumonia” may require ICU admission for acute respiratory failure.

**Patients and methods** Medical charts of adult patients hospitalized between 2000 and 2015 in the ICU of 20 French hospitals with proven atypical pneumonia (positive serology or PCR) were retrospectively reviewed. Patients with *Mycoplasma pneumoniae* infections were compared to microbiologically documented *Streptococcus pneumonia* patients (SP).

**Results** 104 patients were included (71 men, 33 women) with a median age of 56 [44–67] years, mainly admitted to the ICU for acute respiratory failure (n = 96, 92%). *Mycoplasma pneumoniae* was the causative agent in 76 patients (73%) and *Chlamydia pneumoniae* in 28 (27%). Atypical pneumonia was more frequent during autumn and winter. Superinfection was reported in 19 cases (viruses in 47%). Clinically, 33 patients (32%) had at least one extra pulmonary symptom. Chest-X-ray disclosed an involvement of 2 [1–4] quadrants, alveolar opacities (n = 61, 75%), interstitial opacities (n = 32, 40%) or pleural effusion (n = 6, 7%). During ICU stay, 75 patients (72%) required mechanical ventilation. Among them, 34 had ARDS. More than one-third of the patients (n = 41) received vasopressors. ICU length of stay was 14 days [6–21] in patients that were discharged alive and 23 [18–41] in patients who died in the ICU-days; 11 (11%) patients died in the ICU. Factors associated with mortality, in univariate analysis, were age ≥65 years (p = 0.033), signs of respiratory distress (p = 0.017) and interstitial opacities on chest X-ray (p = 0.017).

Characteristics at admission for SP and MP patients were compared (Table 24). HIV infection was more frequently associated with SP pneumonia (16 vs. 3%, p = 0.009). SP patients presented more frequently with shock in (32 vs. 8%, p = 0.0004) and acute renal failure (13 vs. 9%, p = 0.008). SP pneumonia was associated with higher ICU mortality (20 vs. 5% at 28 days, p = 0.005).

**Table 24:** Characteristics at admission for SP patients and MP patients

N (%) or median (IQR)	*Mycoplasma pneumoniae* patients (N = 76)	*Streptococcus pneumoniae* patients (N = 76)	P value
Clinical respiratory findings			
Respiratory rate	33 [27–38]	30 [26–36]	0.43
Signs of respiratory distress	33 (49%)	34 (45%)	0.74
Ronchi	9 (15%)	12 (16%)	1
Crackles	36 (61%)	44 (59%)	1
Signs of consolidation	5 (9%)	22 (30%)	0.008
Decreased vesicular breath sound	10 (17%)	28 (38%)	0.007
Clinical presentation			
Time since symptom onset (days)	6 [4–9]	3 [2–7]	0.0008
Fever	58 (83%)	54 (71%)	0.12
Shock	6 (8%)	24 (32%)	0.0004
Neurological disorders	1 (1%)	20 (26%)	<0.0001
Gastro intestinal symptoms	1 (1%)	15 (20%)	0.0003
Radiological features			
Number of quadrants			
<2	37 (49%)	66 (87%)	
>2	16 (21%)	9 (12%)	
Alveolar opacities	42 (75%)	69 (92%)	
Interstitial opacities	20 (36%)	6 (8%)	
Pleural effusion	3 (5%)	17 (23%)	

**Conclusion** Critically ill patients with severe atypical bacterial pneumonia have a 11% case fatality. This study maintains a high level of suspicion towards atypical pneumonia as compared to SP pneumonia.

**Competing interests** None.

#### P131 Severe pneumonia due to legionella pneumophilia: clinical characteristics and outcomes

##### Takoua Marhbène^1^, Salma Sellami^1^, Amira Jamoussi^1^, Samia Ayed^1^, Emna Mhiri^2^, Leila Slim^2^, Jalila Ben Khelil^1^, Mohamed Besbes^1^

###### ^1^Réanimation médicale, Hôpital Abderrahmen Mami, Ariana, Tunisia; ^2^Microbiologie laboratory, Hôpital Abderrahmen Mami, Ariana, Tunisia

####### **Correspondence:** Salma Sellami - selma_sellami@outlook.com

*Annals of Intensive Care* 2017, **7(Suppl 1)**:P131

**Introduction** Legionnaire’s disease (LD) is a severe pneumonia commonly caused by *Legionella pneumophila* serogroup 1. Despite advances in critical care management and antimicrobial thearapy its morbimortality remains still high. No epidemiologic studies had been reported in Tunisia.

**Objectives:** To study characteristics and outcomes of patients admitted in ICU with severe community acquired pneumonia due to LD over 17 years.

**Patients and methods** It was a retrospective cohort study from the 1st of January 2000 to the 15th of September 2016. We described epidemiology, clinical features, treatment and outcomes of patients admitted to ICU.

**Results** 19 patients were enrolled, the overall cumulative incidence was 0.2 episodes/1000 admissions and 0.17% of all community acquired pneumonia hospitalized during this period. Eighteen patients were males. Median age was 51 years [25–81 years]. A summer and autumnal peaks was observed. Comorbidities were observed in 9 patients. Mean IGSII score was 42 [23–79] and major criteria of ATS were present in 5 cases. At admission in ICU, all patients had acute respiratory failure; 3 gastrointestinal symptoms; 9 hyponatremia, 8 rhabdomyolysis and 12 had elevated liver enzymes. Acute respiratory distress syndrome (ARDS) was present in 16 cases (severe n = 5, mild n = 6, light n = 5). Septic shock and acute renal failure were observed respectively in 5 and 8 cases. Diagnosis was made in all cases by urinary antigen test. *Legionella pneumophila was* present also in sputum in 5 cases. Mechanical ventilation was needed in 13 cases; it was initially NIV in 46%. Fifteen patients were treated by a combination antibiotherapy: It was macrolide and quinolone in 52% (n = 10), quinolone and rifampicine in 31% (n = 5), the others were treated with macrolide or quinolones in monotheray. Median duration of ventilation was 7 days [2–29 days]. Median length of ICU stay was 13 days. Seven patients were died. Univariate analysis showed that presence of major criteria of ATS score at admission (p = 0.001), severe ARDS (p = 0.004), elevated liver enzymes (p = 0.047), needs of mechanical ventilation (p = 0.024) or intubation (p = 0.00), septic shock (p = 0.001); dialysis (p = 0.020) were associated with a higher mortality. Multivariate analysis showed that independent predictive factors of mortality were ATS major criteria OR 3.5 [95% CI (1.08–11.92)]; mechanical ventilation OR 0.5 [95% CI (0.28–0.88)]; severe ARDS OR 0.117 [95% CI (0.017–0.80)]; septic shock OR 0.09 [95% CI (0.015–0.65)] and need of dialysis OR 0.14 [95% CI (0.02–1.06)].

**Conclusion** Our study confirms that LD requiring hospitalization in ICU is associated with high morbimortality. Presence of ATS major criteria, severe ARDS, mechanical ventilation, septic shock and dialysis were the major factors for worse prognosis.

**Competing interests** None.

#### P132 Acute respiratory failure in patients treated with chronic hemodialysis: pulmonary edema or not?

##### Sylvain Chawki^1^, Aicha Hamdi^1^, Magali Ciroldi^1^, Alice Cottereau^1^, Edouard Obadia^1^, Vincent Das^1^

###### ^1^Réanimation polyvalente adulte, Centre Hospitalier Intercommunal André Grégoire, Montreuil, France

####### **Correspondence:** Sylvain Chawki - sylvain.chawki@gmail.com

*Annals of Intensive Care* 2017, **7(Suppl 1)**:P132

**Introduction** Patients on chronic hemodialysis are prone to develop overload pulmonary edema (OPE) but also infections, and have been reported to be regularly admitted to the intensive care unit (ICU) [1, 2]. Acute respiratory failure (ARF) in this specific population can be challenging for the intensivist, as OPE requires ultrafiltration which could be deleterious in other causes. The objectives of this study were (1) to describe the spectrum of ARF causes in patients on chronic hemodialysis and (2) to identify predictors of pulmonary edema diagnosis.

**Patients and methods** We performed a monocentric retrospective study in the 18-bed ICU of a non-teaching hospital with a nephrology unit. All patients on chronic hemodialysis admitted to the ICU for ARF in the period 2011–2015 were included. Patients with unknown diagnosis on ICU discharge were excluded.

We defined ARF as either use of oxygen at ≥6 l/min dioxygen to achieve a pulse oximetry measurement of ≥90% (88% in COPD patients) or partial pressure of arterial oxygen (PaO_2_) <60 mmHg on room air or need for mechanical ventilation plus of one of the following signs: a respiratory rate of >25 breaths/min, use of accessory respiratory muscles, or cyanosis associated to worsening dyspnea. The following data were collected: previous dry weight, time from last dialysis session, clinical, biological and imaging data on admission, number of dialysis sessions performed in ICU and total ultrafiltration volume required to withdraw oxygen therapy, weight on discharge, treatment and outcome, Patients were classified as having OPE (OPE group) or not (non-OPE group) based on the final diagnosis on ICU discharge. The two groups were compared with univariate analysis using non parametric tests.

**Results** 44 patients with 59 episodes of ARF were included in the final analysis.

46 (78%) episodes were due to OPE and 13 (22%) to other causes, including: 8 (61.5%) lower respiratory tract infections, 4 (30.8%) COPD exacerbation and one pulmonary embolism (7.7%). Patients admitted for OPE had higher systolic blood pressure at first medical contact (median [25–75 IQR]: 194 mmHg [176–215] vs 105 mmHg [91–119]; p < 0.0000001), lower CRP on arrival (10 mg/l [6–17] vs 53.5 mg/l [9.8–133.8]; p = 0.006), higher weight gain related to dry weight (2.1 [1–3] vs 0.5 [−0.25 to 2]; p = 0.01) and longer time since last dialysis (2 days [2–3] vs 1 [0–2.5]; p = 0.02). We did not find significant differences in temperature on arrival, white blood cells count nor NT-proBNP. Chest X-Ray interpretation was correct in 82% of cases of OPE patients but could not conclude in 46% of non-OPE episodes. Non-OPE patients had a more severe prognosis 38.5% of death (n = 5) versus 2.2% (n = 1).

**Conclusion** In this small retrospective monocentric study, ARF in patients on chronic hemodialysis is due to non-OPE cause in 22% of cases. A lower blood pressure, a higher CRP, and a shorter time from last dialysis session could prompt physician to search for a non-OPE cause and adjust cautiously ultrafiltration therapy.

**Competing interests** None.

**References**

1. Judd E, Ahmed MI, Harms JC, Terry NL, Sonavane SK, Allon M. Pneumonia in hemodialysis patients: a challenging diagnosis in the emergency room. J Nephrol. 2013;26:1128–35.
2. Halle MP, Hertig A, Kengne AP, Ashuntantang G, Rondeau E, Ridel C. Acute pulmonary oedema in chronic dialysis patients admitted into an intensive care unit. Nephrol Dial Transplant. 2012;27:603–7.

#### P133 Urgent chemotherapy for life-threatening complications related to solid neoplasms

##### Yoann Zerbib^1^, Antoine Rabbat^2^, Muriel Fartoukh^3^, Naïke Bigé^4^, Claire Andrejak^5^, Julien Mayaux^6^, Nicolas de Prost^7^, Benoit Misset^8^, Virginie Lemiale^9^, Fabrice Bruneel^10^, Julien Maizel^11^, Sylvie Ricome^12^, Frédéric Jacobs^13^, Caroline Bornstain^14^, Hervé Dupont^15^, François Baudin^16^, Elie Azoulay^9^, Frédéric Pène^1^

###### ^1^Réanimation Médicale, Hôpital Cochin, Paris, France; ^2^Réanimation pneumologique, Hôpital Cochin, Paris, France; ^3^Réanimation médico-chirurgicale, Hôpital Tenon, Paris, France; ^4^réanimation médicale, Hôpital Saint-Antoine, Paris, France; ^5^Service de Réanimation Pneumologique, CHU Amiens - Hôpital Sud, Amiens, France; ^6^Réanimation médicale, Hôpital Pitié-Salpêtrière, Paris, France; ^7^Réanimation Médicale, Hôpital Henri Mondor, Créteil, France; ^8^Réanimation polyvalente, Groupe Hospitalier Paris-Saint-Joseph, Paris, France; ^9^Réanimation médicale, Hôpital Saint-Louis, Paris, France; ^10^Réanimation médico-chirurgicale, Centre Hospitalier de Versailles, Le Chesnay, France; ^11^Réanimation médicale, Centre Hospitalier Universitaire, Amiens, France; ^12^Service de réanimation polyvalente, Centre hospitalier intercommunal Robert Ballanger, Aulnay-sous-Bois, France; ^13^Réanimation polyvalente, Hôpital Antoine Béclère, Clamart, France; ^14^Réanimation polyvalente, Groupe Hospitalier Intercommunal Le Raincy-Montfermeil, Montfermeil, France; ^15^Réanimation cardio thoracique et vasculaire, CHU Amiens-Picardie, Amiens, France; ^16^Réanimation chirurgicale, Cochin Port-Royal, Paris, France

####### **Correspondence:** Yoann Zerbib - yoanz@hotmail.com

*Annals of Intensive Care* 2017, **7(Suppl 1)**:P133

**Introduction** Both haematological and solid malignancies may be directly responsible for life-threatening organ failures including obstruction of anatomical structures, tissue infiltration, tumor lysis syndrome, or coagulation disorders. Besides advanced life support and eventual instrumental interventions, the treatment of cancer-related organ failures relies on timely administration of chemotherapy. Published data about requirements of chemotherapy in the ICU are mostly related to patients with haematological malignancies, while reports of patients with solid tumors are scarce. In this study, we addressed the features and outcomes of patients with organ failures directly related to solid neoplasms.

**Patients and methods** We performed a retrospective multicenter study within the GrrrOH research network. All adult patients who were admitted to the ICU with organ failures related to solid malignancies and treated with chemotherapy between 2000 and 2015 were enrolled into the study. Data were collected from individual files, and included the overall severity through the SOFA score computed at the time of ICU admission, the type and mechanism of organ failures, and the modalities of chemotherapy administration. Endpoints were the in-ICU and in-hospital vital status.

**Results** 136 patients were included. The most common underlying malignancy was lung cancer (n = 90), distributed between small cell lung cancer (SCLC) (n = 57) and non-small cell lung cancer (NSCLC) (n = 33). Most malignancies were newly diagnosed (n = 122, 89.7%), the diagnosis being made in the ICU for 80 patients (58.9%). The majority of patients (n = 82, 60.3%) had metastasis. The main reason for ICU admission was acute respiratory failure in 111 (81.6%) patients. Compression and tissue infiltration by tumor cells were the leading mechanisms resulting in organ involvement in 78 (57.4%) and 47 (34.6%) patients. Other indications for ICU admission relied on paraneoplastic manifestations in 11 cases. The treatment was based on combined and single chemotherapy in 120 (88.2%) and 16 (11.8%) patients, respectively. The dosing was reduced in 20 patients (20%) mostly linked to renal failure, and the sequence of drug administration was modified in 18 patients (18%). Thirty-four patients (25%) required either instrumental or surgical adjuvant procedures. Eighty-nine patients received invasive mechanical ventilation with duration of 13 (5–25) days, 39 patients required vasopressors (28.7%) and 11 required renal replacement therapy (8.1%).

The overall in-ICU, in-hospital, 6-month and 1-year mortality rates were 37, 58, 74 and 88%, respectively. In a multivariate logistic regression analysis, SCLC was identified as an independent predictor of hospital survival. However this gain in survival was not sustained since the 1-year survival rates of SCLC, NSCLC and non-lung cancer patients all dropped below 20%.

**Discussion** The prognosis of solid neoplasm-related organ failures relies on multidisciplinary management involving both intensivists and oncologists. Appropriate management of cytostatic drugs is paramount to improve their efficacy on tumor cells while minimizing their toxicity. Dosing of chemotherapy in critically ill patients represents a still unexplored area of research owing to a number of factors that may result in drug underdosing or overdosing.

**Conclusion** Acute respiratory failure related to lung cancer represents the main indication for urgent administration of chemotherapy in the ICU. Urgent chemotherapy along with aggressive management of organ failures in the ICU can be life-saving in a number of cancer patients, most especially for SCLC, although the long-term survival is hardly sustainable.

**Competing interests** None.

#### P134 Lung ultrasound for early diagnosis of pneumonia after cardiac surgery

##### Pauline Dureau^1^, Adrien Bouglé^2^, Audrey Tanguy^3^, Charlotte Arbelot^4^, Hamou, Nora Ait^2^, Hassen, Kais Ben^2^, Ahmed Charfeddine^2^, Benjamin Granger^3^, Julien Amour^1^

###### ^1^Service d’anesthésie et de réanimation, institut de cardiologie, pitié salpetrière hospital, Faculté de Médecine Pierre et Marie Curie, Paris, France; ^2^Service d’anesthésie et de réanimation, institut de cardiologie, Pitié-Salpêtrière Hospital, Paris, France; ^3^Département de biostatistiques, santé publique et information médicale, Pitié-Salpêtrière Hospital, Paris, France; ^4^Réanimation chirurgicale polyvalente, Hôpital de la Pitié-Salpêtrière, Paris, France

####### **Correspondence:** Pauline Dureau - pauline.dureau@gmail.com

*Annals of Intensive Care* 2017, **7(Suppl 1)**:P134

**Introduction** Pneumonia is a frequent and severe complication of major cardiac surgery, contributing to postoperative morbidity and death [1]. Diagnosis of pneumonia remains a challenge in ventilated patients, notably after cardiac surgery, and the diagnostic performance of Clinical Pulmonary Infection Score (CPIS) remains controversial. Lung ultrasound (LUS) has been successfully used for the diagnosis and the management of community acquired pneumonia and ventilator-associated pneumonia (VAP) [2], but LUS usefulness and reliability was never investigated in the specific ICU patients after cardiac surgery with cardiopulmonary bypass (CPB). This pilot observational study investigated the clinical relevance of lung ultrasonography (LUS) for diagnosis of pneumonia in cardiac postoperative patients with acute respiratory failure (ARF).

**Patients and methods** Adult patients were prospectively enrolled from January through May 2015 in presence of acute respiratory failure (ARF) less than 3 days after a cardiac surgery with CPB. Lung ultrasound examination was performed as follows: subpleural consolidation, lobar consolidation, dynamic and static air bronchogram, and intrapulmonary shunt. We compared this ultrasound approach to the post hoc diagnosis of pneumonia established from clinical, radiologic, and biologic data by three blinded experts. Then, we compared the diagnostic performances of CPIS, ultrasound criteria and a lung ultrasound based CPIS, LUS-CPIS, with or without radiology criteria. The study was approved by the Comité de Protection des Personnes Ile de France III; IRB 2015-A00127-42 Réf. S.C. 3275.

**Results** Fifty-one patients (age 65 ± 12 years, male sex 72.6%) with ARF were included in the study, and pneumonia was diagnosed in 26 of them. No difference was observed between patients with or without pneumonia among demographic data, type of surgery, CPB length. In case of pneumonia, the most frequently identified pathogens were Enterobacteriaceae (37%), *Pseudomonas aeruginosa* (15%) and *Haemophilus influenza* (8%). Multivaried analysis showed that intrapulmonary shunt was the best predictive criteria of pneumonia (odds-ratio 1.92; 95% CI [1.00; 5.43]). The ultrasound diagnosis was more accurate than the simplified CPIS for the diagnosis of pneumonia, area under the curve (AUC) respectively 0.75 (95% CI [0.62; 0.87] vs 0.59 (95% CI [0.47; 0.71]). Receiver operating characteristic curves analysis showed that the most accurate score for diagnosis of pneumonia was LUS-CPIS without radiology (AUC, 0.80 [0.69; 0.91]), with a sensitivity of 92% (95% CI [0.85; 0.99]) and a specificity of 68% (95% CI [0.55; 0.81]), a positive predictive value of 75% (95% CI [0.64; 0.88]) and a negative predictive value of (95% CI [0.63; 0.87]) (Fig. 30). Furthermore, our results suggest the use of ultrasound data may consistently reduce misuse of antibiotics of 70%.

**Fig. 30:**
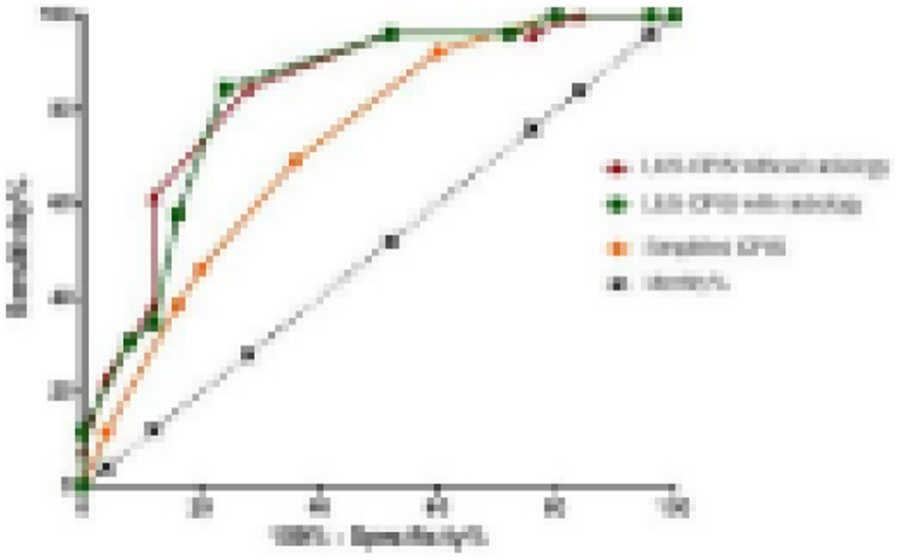
See text for description

**Discussion** A new score, combination of simplified CPIS and a simple ultrasound parameter, intra-pulmonary shunt, predicts efficiently the presence of pneumonia after cardiac surgery with CPB. Two LUS parameters, presence of a shunt and a bronchogram aeric within a consolidation, are crucial for discerning pneumonia from another etiology. This approach has the advantage to make an early diagnosis of pneumonia in ARF patients with an inexpensive, non-invasive and convenient at bedside technology.

**Conclusion** This prospective observational study is the first one showing that LUS combined with a clinical score can be a reliable tool for early diagnosis of pneumonia in a cardiac ICU population after cardiac surgery with CPB.

**Competing interests** None.

**References**

1. Hortal J, Giannella M, Pérez MJ, Barrio JM, Desco M, Bouza E, et al. Incidence and risk factors for ventilator-associated pneumonia after major heart surgery. Intensive Care Med. 2009;35(9):1518–25.
2. Mongodi S, Via G, Girard M, Rouquette I, Misset B, Braschi A, et al. Lung ultrasound for early diagnosis of ventilator-associated pneumonia. Chest. 2016;149(4):969–80.

#### P135 Trends in intensive care admission for respiratory infections attributable to the elderly in aging population

##### Lucile Laporte^1^, Coralie Hermetet^2^, Youenn Jouan^1^, Christophe Gaborit^3^, Leslie Grammatico-Guillon^4^, Antoine Guillon^1^

###### ^1^Réanimation polyvalente, CHRU Hôpitaux de Tours, Tours, France; ^2^Service d’information médicale, d’épidémiologie et d’économie de la santé, CHRU Hôpitaux de Tours, Tours, France; ^3^Service d’information médicale, d’épidémiologie et d’économie de la santé, CHU Bretonneau, Tours, France; ^4^Service d’information médicale, epidémiologie et economie de la santé, CHRU Hôpitaux de Tours, Tours, France

####### **Correspondence:** Lucile Laporte - antoine.guillon@univ-tours.fr

*Annals of Intensive Care* 2017, **7(Suppl 1)**:P135

**Introduction** Acute respiratory infection (ARI) is the most common infectious cause for admission to Intensive Care Unit (ICU) and the prevalence of infection increases with age. Changes in population demographics and comorbid illness may drive important changes in the composition of patients admitted to the ICU. Notably, how the population aging impacts on the incidence of hospitalized patients for ARI and how it increases the demand for critical care services is unknown. To address this knowledge gap, we sought to describe trends in demographics changes among elderly patients admitted to ICU for ARI on a 9-year period.

**Materials and methods** We conducted a retrospective cross-sectional study based on hospital discharge databases (HDDs) from January 1, 2006 to December 31, 2014. We selected patients over 18 years old (y-o) who were hospitalized for ARI in a French region (Centre Val de Loire region, 2.5 million inhabitants, served by one university hospital, one regional hospital and 37 general and private hospitals). Cases of ARI were extracted from the HDD with an algorithm based on ICD-10 specific diagnosis codes, taking into account the type, number and position of these codes in the hospital discharge report. We previously validated the ICD-10 case definition reviewing a sample of medical charts as the gold standard. Giving the acceptable accuracy and precision of our algorithm, the following data were extracted from the HDD: patient characteristics and comorbidities, ICU hospitalization, use of mechanical ventilation, length of stay, occurrence of death.

**Results** On the study period, the number of hospitalization for ARI has almost doubled: from 6751 cases in 2006 to 11,744 cases in 2012, and 10,167 cases in 2014. The incidence of ARI increased in all age class but was more important for patient over 85 y-o. Among these hospitalized patients, 740 were hospitalized in ICU in 2006 and nearly 2000 in 2014. Importantly, the rate of ICU-hospitalization among hospitalized patients for ARI increased on the study period: 11% in 2006 whereas it was superior to 17% since 2010. This trend for increase ICU admission was proportional with aging: 2-fold increase at 75–79 y-o, 2.5-fold increase at 80–84 y-o, threefold increase at 85–90 y-o, and 4.5-fold increase for patient over 90 y-o. The overall hospital-mortality was relatively stable and varied between 6.7 and 9% for non-ICU patients and 14.4–17.2% in ICU-patients. Regarding patients over 90 y-o hospitalized in ICU, the mortality has been drastically reduced: from 40.9 to 22.2% on the study period.

**Discussion** This large prospective study provides a contemporary view of elderly with severe ARI admitted to ICUs. We found significant increase in elderly patient with a primary diagnosis of ARI hospitalized in ICU. We highlighted that ARI is an increasing reason for death among ICU-hospitalized patient and still a major public health problem. Interestingly, our results are consistent with longitudinal changes in ICU admission also observed in this study period in the United States.

**Conclusion** We found substantial increase of ARI diagnoses leading to hospitalization between 2006 and 2014 with a growing demand for critical care service. Further studies are needed to evaluate the benefits of intensive care hospitalization for the very elderly patients. We cannot arbitrary reject the very elderly from the doors of the ICU—it is ageism—but we need information to select more accurately the patients with the highest probability of survival, and to avoid useless and aggressive cares when it is not appropriate.

**Competing interests** None.

#### P137 Pulmonary embolism in intensive care unit: incidence and impact prognosis

##### Kais Regaieg^1^, Mabrouk Bahloul^2^, Najeh Baccouch^3^, Olfa Turki^2^, Rim Khemakhem^2^, Chtara Kamilia^2^, Hedi Chelly^2^, Mounir Bouaziz^2^

###### ^1^ICU, CHU Habib Bourguiba, Sfax, Tunisia; ^2^Réanimation polyvalente, Faculté de médecine de Sfax, Sfax, Tunisia; ^3^Réanimation polyvalente, CHU Habib Bourguiba, Sfax, Tunisia

####### **Correspondence:** Kais Regaieg - kais.regaieg@gmail.com

*Annals of Intensive Care* 2017, **7(Suppl 1)**:P137

**Introduction** Pulmonary embolism is a major complication observed in critical ill patients. ICU environment patients are at high risk of deep vein thrombosis and pulmonary embolism. The objective of our study is to determine the incidence of this pathology and to analyze the prognosis of patients having a pulmonary embolism in ICU.

**Patients and methods** Prospective study included all patients with confirmed pulmonary embolism by a spiral computed tomography scan showing one or more filling defects in the pulmonary artery or in its branches, over a period of 1 year from 1 July 2014 to 30 June 2015. We studied the epidemiological, clinical, biological and radiological these patients. We compare two groups in univariate analysis: survivors and deaths.

**Results** During the study period, 705 patients were admitted in our ICU. The diagnosis of PE was confirmed in 75 patients (10.6%). The mean age was 53.2. The sex ratio (M/W) was 5.25. SAPSII was 37, SAPSIII was 43 and SOFA was 5. At admission in ICU, 90% of patients were ventilated. The mean delay of development of PE was 7.15. On the day of PE diagnosis, shock index was 0.94, clinical examination showed that 22 patients (29.3%) were hypotensive, 33 (44.4%) have SIRS, 9 (12%) have clinical manifestations of DVT and 69 (92%) have respiratory distress requiring mechanical ventilation. All patients underwent spiral computed tomography scan. PE was proximal in 58 patients (77%) and bilateral in 7 patients (9.3%). In our study, intravenous unfractionated heparin was used in 70 cases (93.3%) and low molecular weight heparins were used in 5 cases (6.66%). The mean ICU stay was 20.2 ± 25.3 days and the mean hospital stay was 26.3 ± 26 days. The mortality rate in ICU was 38.7%.

Moreover, comparison between survivors and deceased showed that factors associated with deaths were: high SAPS II score on ICU admission, high SAPS III score on ICU admission, high SOFA score, low shock index on the day of pulmonary embolism, hypoxemia with PaO_2_/FiO_2_ < 200 mmHg and the presence of shock.

**Conclusion** Pulmonary embolism (PE) is a frequent, in ICU. It is associated with a high ICU and in-hospital mortality rate. high SAPS II score on ICU admission, high SAPS III score on ICU admission, high SOFA score, low shock index on the day of pulmonary embolism, hypoxemia with PaO_2_/FiO_2_ < 200 mmHg and the presence of shock. Prevention is advised.

**Competing interests** None.

#### P138 Correlation between the transcranial color-coded duplex sonography and the optic nerve sheath diameter in the prediction of intracranial hypertension

##### Chaigar Mohammed Cheikh^1^, Hamid Mountij^1^, Kawtar Rghioui^1^, Wafae Haddad^1^, Rachid Cherkab^1^, Houcine Barrou^1^, Aitmouden Naima^2^, Othmani M. Bennani^2^

###### ^1^Anesthésie réanimation, Chu Ibn Rochd, Casablanca, Morocco; ^2^Medical informatics laboratory, Chu Ibn Rochd, Casablanca, Morocco

####### **Correspondence:** Chaigar Mohammed Cheikh - chaigarmed@gmail.com

*Annals of Intensive Care* 2017, **7(Suppl 1)**:P138

**Introduction** Intracranial hypertension is suspected on clinical, radiological and ophthalmological criteria. The measurement of intracranial pressure is the reference method. Due to infectious and hemorrhagic risks, other non-invasive techniques have been developed such as the transcranial color-coded duplex sonography (TCCDS) and the optic nerve sheath diameter (ONSD). The aim of our study was to compare TCCDS data to the ONSD measurements and to CT scanning data in predicting intracranial hypertension.

**Patients and methods** Prospective study enrolling all patients admitted to our ICU for severe traumatic brain injury between February and August 2016. The intracranial hypertension was studied by CT scanning, TCCDS and ocular ultrasonography. CT defines intracranial hypertension by a midline shift >5 mm, collapsed third ventricle, presence of hydrocephalus and erased basal cisterns with significant edema. TTCDS identifies a significant intracranial hypertension by a pulsatile index (PI) >1.2 and a diastolic velocity <20 cm/s. An ONSD > 5 mm is considered abnormal. The study of associations was done using non-parametric tests (Wilcoxon and Fisher) and data analysis was performed by epi-info software.

**Results** Sixty patients with severe traumatic brain injury were included in the study. The average age was 42 ± 17 years. The sex-ratio was 1.4. The intracranial hypertension was diagnosed by pupillary abnormalities in 36% of cases, CT scanning in 84% of cases, TTCDS in 68% of cases and ONSD in 69% of cases. There was no significant correlation between an ONSD > 5 mm and CT scanning findings (p = 0.58) nor with TCCDS (p = 0.08) in predicting intracranial hypertension.

**Conclusion** ONSD is a quick and simple method that has been widely proven to predict intracranial hypertension. A larger sample is needed to confirm our results.

**Competing interests** None.

#### P140 Traumatic brain injury caused by traffic accidents: epidemiology and prognostic factors: a multivariate analysis of 694 cases

##### Hedi Chelly^1^, Kais Regaieg^2^, Ahmed Douib^1^, Amal Samet^3^, Chtara Kamilia^1^, Mabrouk Bahloul^1^, Mounir Bouaziz^1^

###### ^1^Réanimation polyvalente, Faculté de médecine de Sfax, Sfax, Tunisia; ^2^ICU, CHU Habib Bourguiba, Sfax, Tunisia; ^3^Réanimation polyvalente, CHU Habib Bourguiba, Sfax, Tunisia

####### **Correspondence:** Kais Regaieg - kais.regaieg@gmail.com

*Annals of Intensive Care* 2017, **7(Suppl 1)**:P140

**Introduction** Head injuries are a public health problem. Studies on the epidemiology and especially the prognosis of head trauma are rare in developing countries. The objective of our study is to determine the epidemiological aspects and to identify factors correlated with short- and long-term prognosis.

**Patients and methods** It was a retrospective study during 4 years (January 2009–December 2012) included 694 patients with traumatic brain injury due to traffic accidents. We studied the factors correlated with poor prognosis in terms of death or Glasgow Outcome Scale (GOS) in univariate and multivariate analysis.

For study according to GOS, patients (n = 694) were divided in two groups: favorable GOS for patients having a good recovery or recovery with a minor handicap [GOS class 4 or5] and unfavorable GOS for patients having a severe handicap or vegetative state [GOS class 1or 2] or died [GOS1].

**Results** During the study period, 694 patients (18.7% of all patients) were admitted to our ICU with traumatic brain injury due to traffic accidents. The mean of age was 31.9. The sex ratio was 5.8. In admission, 20% of patients had hypotension, 17.4% of patients had respiratory distress and the mean GSC was 8.8. The mortality rate was 28.5%. 496 patients were survivor: 13 patients with vegetative state, 133 with severe handicap (19.3%), 185 patients with minor handicap (26.7%), and 163 patients with good recovery (23.6%). The predictive independent factors correlated to mortality in multivariate analysis were: age ≥38, hypotension, hypoxemia, SGS ≤ 8, unilateral or bilateral mydriasis, cerebral edema, Marshall Class VI, initial hemoglobin ≤11.3, Blood sugar ≥8.3 mmol/l. The predictive factors correlated with poor prognosis according GOS were: age ≥38, initial shock, SGC ≤ 8, unilateral or bilateral mydriasis, post traumatic coma duration more than 5 days, cerebral edema, initial hemoglobin ≤11.7 g/dl, blood sugar ≥8.4 mmol/l and catecholamine using.

**Conclusion** The short-term prognosis of head trauma seems recently stabilized even for the most serious patients, but the distant consequences of head trauma are fairly frequent, heavy and often undervalued. That is why, it is so important to identify these consequences and management them correctly.

**Competing interests** None.

#### P141 Benefice of rib fixation in chest trauma: a before/after study

##### Pierre-Julien Cungi^1^, Cédric Nguyen^1^, Jean Cotte^1^, Erwan D’aranda^1^, Eric Meaudre^1^, Jean-Phillipe Avaro^2^

###### ^1^Intensive Care Unit and Anesthesiology, Hôpital d’Instruction des Armées Sainte-Anne, Toulon, France; ^2^Thoracic Surgery Department, Hôpital d’Instruction des Armées Sainte-Anne, Toulon, France

####### **Correspondence:** Pierre-Julien Cungi - pjcungi@gmail.com

*Annals of Intensive Care* 2017, **7(Suppl 1)**:P141

**Introduction** Fifteen percent of the trauma has a chest trauma. Depending on the type of injury, mortality raises from 4 to 60%. There are no surgical guidelines concerning rib fracture except for flail chest needing mechanical ventilation. No trial has yet assessed the global benefit of both surgical and medical treatment.

The primary endpoint was to determine wether rib fixation on the one hand and epidural analgesia on the other hand reduces pneumonia. The secondary endpoints were to show a decrease of mechanical ventilation duration and lengths of stay in intensive care and hospital.

**Materials and methods** We performed a pre/post osteosynthesis study. We determined two groups. From 01/2011 to 08/2014, all patients admitted for chest trauma were screened by a thoracic surgeon in order to select those with a rib fixation indication. They formed the historical cohort: «not fixed». From 08/2014 to 08/2016, all patients with chest trauma and rib fixation were included. They formed the group «fixed». Were excluded patients who died within the first 48 h. The surgical treatment is defined by a rib fixation. The medical treatment is defined by epidural analgesia and noninvasive ventilation.

**Results** One hundred and twelve patients with equivalent surgical indication of rib fixation were enrolled from January 2011 to August 2016. Fifty-seven patients were not fixed and 55 patients were fixed. Eighty-two (73%) were men of 57 (20–86) years old. The mean ISS was 22 (4–66). The mean SAPS II was 25 (6–86). Eighty-five patients (80%) were hospitalized in Intensive care. 72 patients (64%) had an epidural analgesia. Fifty percent of the patients were operated on within the first 24 h. Thirty-three patients suffered from pneumonia that occurred on average on the fifth (2–10) day. Epidural analgesia significantly decreased the incidence of pneumonia (p < 0.001). Rib fixation had no impact on the incidence of pneumonia (p = 0.1). Both length of stay in hospital and in intensive care were neither reduced by epidural analgesia nor by rib fixation. Epidural analgesia reduced mechanical ventilation duration. Rib fixation didn’t reduce mechanical ventilation duration.

**Conclusion** It is the first study to assess the importance of both medical and surgical care of chest trauma. Epidural analgesia is effective in decreasing incidence of pneumonia whatever the rib fixation.

**Competing interests** None.

#### P142 Prognostic factors of severe liver injury

##### Med Aziz Bouhouri^1^, Mohamed Taoufik Slaoui^1^, A. Soufi^1^, K. Khaleq^1^, D. Hamoudi^1^, A. Nsiri^1^, R. Harrar^1^

###### ^1^Reanimation des urgences chirurgicale, chu ibn rochd, Casablanca, Morocco

####### **Correspondence:** Mohamed Taoufik Slaoui - dr.t.slaoui@gmail.com

*Annals of Intensive Care* 2017, **7(Suppl 1)**:P142

**Introduction** Serious liver injuries classified have very high mortality figures despite improved care systems and means of paraclinical investigations.

Stud Objective: The purpose of this study was to determine the prognostic factors of severe liver injury by evaluating epidemiological, diagnostic, therapeutic and evolutionary data and this to promote conservative therapeutic management.

**Patients and methods** In this retrospective clinical study over a period of 3 years (May 2013–May 2016), patients admitted to the intensive care surgical emergencies Ibn Rochd CHU Casablanca for serious liver injury were evaluated on specific criteria for inclusion and exclusion in using a pre-established operating profile including epidemiological, clinical, biological, radiological and therapeutic findings with a significance level set at 5%.

**Results** After this study, 23 patients were compiled. 19 patients were male and 4 female, mean ages was 34 ± 11.7 years with a range from 17 to 58, the etiologies were dominated by accidents of the high way followed by assaults and finally fall from heights.

An unstable hemodynamic status was initially found in 6 cases (26%) and the use of vasoconstrictor was necessary in 18 patients (78.2%).

Ultra sonography was performed in 16 patients (69.5%) and objectified isolated hem peritoneum or associated with visceral lesions in all patients.

Abdominal CT scan was performed to 20 patients (87%) objectifying in 10 cases (50%) different findings with those of ultrasound.

An emergency surgery was raised in 15 cases and delayed surgery was indicated in 3 patients.

Mortality in our study was 39.13% where hemorrhagic shock presents the leading cause. The prognosis was closely linked to the value of systolic blood pressure at admission, the SPO_2_, the Glasgow coma scale, haemoglobin value, the presence of hem peritoneum of great abundance and whether or not a use of vasoconstrictors.

**Conclusion** The development of imaging techniques in emergency, especially FAST ultrasound and computerized tomography scan, have modified diagnostic and therapeutic attitude in severe liver I jury over the last years. The yellow a conservative approach based on the stability of the hemodynamic status and the response to new resuscitation techniques, this approach requires strict supervision and involves a multidisciplinary team (intensivist, surgeon and radiologist) available in emergency for cases with complications.

**Competing interests** None.

#### P143 Acute burn care: A mythe or a reality?

##### Amel Mokline^1^, Imene Rahmani^1^, Achraf Laajili^1^, Helmi Amri^1^, Lazheri Gharsallah^1^, Bahija Gasri^1^, Sofiene Tlaili^1^, Rym Hammouda^1^, Amen Allah Messadi^1^

###### ^1^Burn Care Department, Trauma and Burn Center, Tunis, Tunisia

####### **Correspondence:** Amel Mokline - dr.amelmokline@gmail.com

*Annals of Intensive Care* 2017, **7(Suppl 1)**:P143

**Introduction** Early burn resuscitation of major burn-injured patients is the cornerstones of burn care and aims to improve outcome and decreases morbidity and mortality rates of these patients. So, initial care of severely burned patients requires participation of all physians in every discipline and in every hospital. The goal of this study was to examine characteristics of burn patients acutely transferred to our intensive burn care unit and to assess their prognosis.

**Patients and methods** A prospective study was conducted in intensive burn care center in Tunis. All consecutive adult burned patients acutely transferred to our burn center, from January 1st to September 23st, 2016 were included in the study. Demograhic, clinical and biological data of patients were recorded.

**Results** During the 9 month study period, 190 patients were admitted among which 101 patients were acutely transferred from other hospitals (53%). The mean age was 37 ± 15 years. The mean surface burned area announced was 44 ± 22 versus 36 ± 22% reevaluated at admission. Patients were transferred with a delay of 40 H after burns [H1–H264]. Burn injuries were caused by domestic accidents in 42%, self immolation in 26% and work related burns in 14%. Transfer with medical agreement was noted in 57% of cases. At admission, 12% of patients had burn shock and 43% had endotracheal intubation. A central venous cathter was placed in 51% of cases, nasogastric tubes in 11% and urinary devices in 55% of cases. Dressing were performed in 66% of cases. Fluid resuscitation was initiated in 74% of cases with crystalloid: Ringer lactate (42%) and/or normal saline (19%). Initial lactate of patients was 3.36 ± 1.7 mmol [1–8.8] with pH at 7.33 [6.6–7.51] and bicarbonates at 20 ± 5 [9–34]. We noted that patients transferred without medical agreement had more burn shock (16.2 vs 8.6%) and a higher mortality (25.5 vs 17.2%).

**Conclusion** Early critical care of severely burned patients, especially, fluid resuscitation and monitoring, coupled with appropriate early referral to a specialist, greatly help in minimizing complications and optimizing prognosis.

**Competing interests** None.

